# *Fusobacterium nucleatum* Causes Microbial Dysbiosis and Exacerbates Visceral Hypersensitivity in a Colonization-Independent Manner

**DOI:** 10.3389/fmicb.2020.01281

**Published:** 2020-06-24

**Authors:** Xiang Gu, Li-jin Song, Li-xiang Li, Tong Liu, Ming-ming Zhang, Zhen Li, Peng Wang, Ming Li, Xiu-li Zuo

**Affiliations:** ^1^Department of Gastroenterology, Qilu Hospital, Cheloo College of Medicine, Shandong University, Jinan, China; ^2^Laboratory of Translational Gastroenterology, Qilu Hospital, Cheloo College of Medicine, Shandong University, Jinan, China

**Keywords:** *Fusobacterium nucleatum*, irritable bowel syndrome, visceral hypersensitivity, intestinal microbiota, immunoglobulin A

## Abstract

**Background:** Microbial dysbiosis is closely associated with visceral hypersensitivity and is involved in the pathogenesis of irritable bowel syndrome (IBS), but the specific strains that play a key role have yet to be identified. Previous bioinformatic studies have demonstrated that *Fusobacterium* is a shared microbial feature between IBS patients and maternal separation (MS)-stressed rats. In this study, we assessed the potential role of *Fusobacterium nucleatum* (*F. nucleatum*) in the pathogenesis of IBS.

**Methods:** Fecal samples of patients with diarrhea predominant-IBS (IBS-D) and healthy controls were obtained. An MS rat model was established to receive gavage of either *F. nucleatum* or normal saline. Visceral sensitivity was evaluated through colorectal distension test, and fecal microbiota was analyzed by 16S rRNA gene sequencing. *F. nucleatum*-specific IgA levels in fecal supernatants were assessed by western blotting. The antigen reacted with the specific IgA of *F. nucleatum* was identified by mass spectrometry and the construction of a recombinant *Escherichia coli* BL21 (DE3).

**Results:** IBS-D patients showed a lower Shannon index and a higher abundance of *Fusobacterium*. The *F. nucleatum*-gavage was shown to exacerbate visceral hypersensitivity in MS rats, with both the *F. nucleatum*-gavage and MS causing a decreased Shannon index and a clear segregation of fecal microbiota. In addition, specific IgA against *F. nucleatum* was detected in fecal supernatants of both the *F. nucleatum*-gavaged rats and the IBS-D patients. The FomA protein, which is a major outer membrane protein of *F. nucleatum*, was confirmed to react with the specific IgA of *F. nucleatum* in fecal supernatants.

**Conclusion:** *Fusobacterium* increased significantly in IBS-D patients, and *F. nucleatum* was involved in the pathogenesis of IBS by causing microbial dysbiosis and exacerbating visceral hypersensitivity in a colonization-independent manner. Meanwhile, *F. nucleatum* was found to induce an increase in specific secretory IgA through FomA.

## Introduction

Irritable bowel syndrome (IBS) is a common functional gastrointestinal disorder that primarily manifests as abdominal pain and stool irregularities (Enck et al., 2016). Although IBS affects 11% of the global population (Lovell and Ford, 2012), its pathogenesis still remains unelucidated.

Due to the importance of the gut microbiota and viscera in the microbiome–gut–brain axis, both microbial dysbiosis and visceral hypersensitivity have been considered to be associated with the severity of IBS symptoms (Malinen et al., 2010; Simren et al., 2013, 2018). IBS patients often suffer from psychologically abnormal comorbidity (Bengtson et al., 2015) that can lead to the development of visceral pain sensation (Greenwood-Van Meerveld and Johnson, 2017). Moreover, fecal microbiota from IBS patients was observed to cause visceral hypersensitivity (Crouzet et al., 2013) and motility dysfunction when inoculated into germ-free mice (De Palma et al., 2017). However, a unique mechanism of microbially induced IBS has not been identified.

In a previous study, maternal separation (MS)-stressed rats were observed to form independent clusters that differed from the normal control group, and *Fusobacterium* was subsequently identified as one of the shared microbial features between IBS patients and MS-stressed rats in our previous research (Zhou et al., 2016). Thus, *Fusobacterium* may be closely related to the visceral hypersensitivity associated with IBS. *Fusobacterium nucleatum* (*F. nucleatum*), a typical strain of the genus *Fusobacterium*, is a gram-negative, obligate anaerobic bacterium that constitutively colonizes the oral mucosa (Aagaard et al., 2012; Segata et al., 2012). *F. nucleatum* has been suggested to contribute to the etiology of some gastrointestinal disorders, such as appendicitis, colon cancer, and inflammatory bowel disease (IBD) (Han, 2015). In addition, *F. nucleatum* has been shown to be responsive to stress hormones and is associated with pain and cold sensitivity in the oral cavity (Hahn et al., 1993; Massey et al., 1993; Sandrini et al., 2015). Nevertheless, an understanding of the effect of *F. nucleatum* in visceral hypersensitivity, its mechanism in the pathogenesis and/or manifestation of IBS symptoms, and the correlation between *F. nucleatum* and IBS symptoms has remained elusive. In this study, to confirm the role of *F. nucleatum* in the pathogenesis of IBS, the effects of *F. nucleatum* in the development of hypersensitivity and the presence of *F. nucleatum-*specific IgA in fecal samples was evaluated.

## Materials and Methods

### Participants

Patients with diarrhea predominant-IBS (IBS-D) (diagnosed as per the Rome IV criteria) and healthy controls (HC) aged 18–65 years were recruited from Qilu Hospital of Shandong University between August 2016 and March 2018. This study was approved by the ethics committees in Qilu Hospital of Shandong University. Patients suspected of being postinfectious IBS were excluded due to the heterogeneity in pathophysiological mechanisms. The exclusion criteria included pregnancy; lactation; previous surgeries within half a year; vaccination within 3 months; organic diseases or mental diseases (e.g., IBD, coeliac disease, anaphylactic diseases, and/or clinical psychiatric disorders); treatment with systemic corticosteroid, antipsychotics, and immunosuppressant agents; and taking antibiotics, probiotics, and laxatives within a month. The severity of symptoms was assessed by the IBS symptom severity scale (IBS-SSS) (Francis et al., 1997). The Self-rating Anxiety Scale (SAS) (Zung, 1971) and Self-rating Depression Scale (SDS) (Zung, 1965) were used to evaluate anxiety and depression. All subjects gave written informed consent.

### Animal Maintenance and Modeling

Pregnant Sprague Dawley rats were purchased from Beijing HFK Bioscience Co., Ltd, and fed in the Experimental Animal Center of Shandong University. All animals were maintained under pathogen-free conditions (22 ± 2°C, 12-h light–dark cycle). All experiments performed on animals were approved by the Ethical and Institutional Animal Care and Use Committee of Qilu Hospital of Shandong University.

**FIGURE 1:**
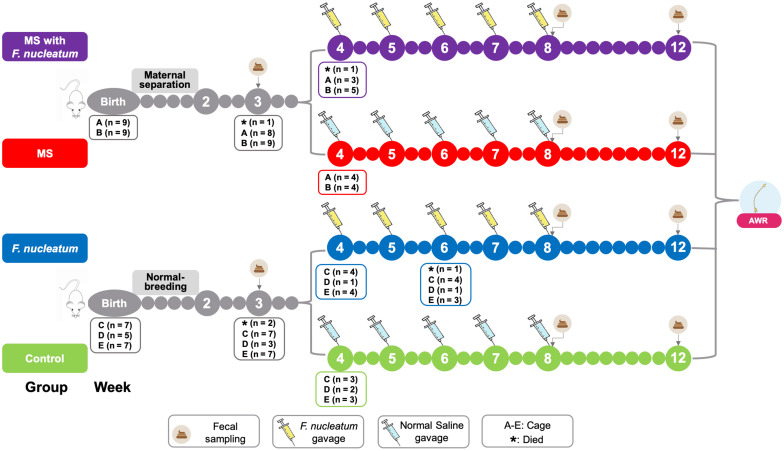
Study design. The treatment and the cohousing association of the experimental rats. MS, maternal separation; *F. nucleatum*, *Fusobacterium nucleatum*; AWR, abdominal withdraw reflex.

The experimental procedure is shown in Figure 1. The MS pups were isolated from dams for 3 h (from 9:00 to 12:00 am) between postnatal days 2 and 14 (O’Mahony et al., 2011). All pups were weaned on postnatal day 22. Male pups were preferred since female pups are subject to hormonal alterations. The pups received either *F. nucleatum* (ATCC 25586, 109 cfu, 1 ml/100 g) or normal saline (1 ml/100 g) once a week between the 4th and 8th weeks by oral gavage. Based on animal modeling, four groups were used in this study: group MS with *F. nucleatum*, the MS and *F. nucleatum*-gavage treatment group; group *F. nucleatum*, the normal-breeding and *F. nucleatum*-gavage treatment group; group MS, the MS and normal saline-gavage treatment group; and control group, the normal-breeding and normal saline-gavage treatment group. Fecal samples were collected at the end of weeks 3, 8, and 12. Visceral sensitivity was evaluated at week 12.

### Bacterial Strains and Culture Conditions

*F. nucleatum* strain ATCC 25586 was purchased from the General Microbiological Culture Collection Center (Beijing, China). *Escherichia coli* (*E. coli*) BL21 (DE3) was purchased from TransGen Biotech (Beijing, China). *F. nucleatum* was cultured anaerobically at 37°C for 18 h in Brain Heart Infusion Broth (Qingdao Haibo Biotechnology Company, Qingdao, China, HB8297-4) before harvesting, while *E. coli* BL21 (DE3) strain was cultured aerobically at 37°C for 8 h in Luria-Bertani (LB) infusion broth.

### Fecal Sample Collection, DNA Extraction, and 16S rRNA Gene Sequencing

Fecal samples collected from the IBS-D patients, HC, and rats were rapidly frozen in liquid nitrogen and stored at -80°C. These samples were then shipped to Majorbio (Shanghai, China) for high-throughput sequencing (Tang et al., 2018).

A FastDNA SPIN Kit (MP Biomedicals, Irvine, CA, United States) was used to extract stool DNA. Polymerase chain reaction (PCR) amplification of the V3–V4 regions of the bacterial 16S rRNA gene was performed with barcode-indexed primers (338F: ACTCCTACGGGAGGCAGCAG, 806R: ACTCCTACGGGAGGCAGCAG) and TransStart FastPfu DNA Polymerase (TransGen, Beijing, China) in an ABI GeneAmp instrument (ABI, United States). The amplicons were subsequently purified by gel extraction (AxyPrep DNA Gel Extraction Kit, Axygen, Union City, CA, United States) and quantified using a QuantiFluor-ST instrument (Promega, United States). The purified amplicons were pooled in equimolar concentrations, and paired-end sequencing was performed using an Illumina MiSeq System (Illumina, San Diego, CA, United States).

### Visceral Hypersensitivity Evaluation

Visceral sensitivity was evaluated by colorectal distension (CRD) test (Xu et al., 2013). A urinary catheter (4 mm in diameter) was inserted into the colon via the anus. The balloon was placed 6 cm proximal to the anus and secured at the tail. The rats took approximately 10 min to adapt. Subsequently, the balloon was filled with saline to a constant volume. Each volume was repeatedly measured 5 times, 20 s at a time with a 5-min break. The abdominal withdraw reflex (AWR) scores were recorded as per the aforementioned methods (Al-Chaer et al., 2000). The overall visceral sensitivity was determined using the visceral hypersensitivity index (VHI), which involves obtaining the sum total of the AWR score from the balloon volume (Zhou et al., 2016).

### Detection of *F. nucleatum*

*F. nucleatum* gene was detected by PCR. Total DNA was extracted from the stool of rats using TIANamp Stool DNA Kit (Tiangen, Beijing, China, DP328). The detection of *F. nucleatum* was performed using specific primers (Forward primer: 5′-CAACCATTACTTTAACTCTACCATGTTCA-3′; Reverse primer: 5′-GTTGACTTTACAGAAGGAGATTATGTAAAAATC-3′, Sangon Biotech Company, Shanghai, China) in a Mastercycler^®^ pro S instrument (Eppendorf, Germany). Positive and negative controls were standard strains of *F. nucleatum* (ATCC 25586) and *E. coli* BL21 (DE3), respectively. The V4 region of the bacterial 16S rRNA gene was amplified by primers (515F: 5′-GTGCCAGCAGCCGCGGTAA-3′ and 806R: 5′-GGACTACCAGGGTATCTAAT-3′, Sangon Biotech Company, Shanghai, China) (Zhao et al., 2017) as internal control.

### Specific IgA Detection and Antigen Identification

#### Fecal Supernatant (FSN) Collection

The fecal samples of the IBS-D patients, healthy volunteers, and rats were diluted in normal saline (1 g sample/7 ml normal saline) supplemented with 1% PMSF (Solarbio, Beijing, China, P0100) and homogenized on ice (Wang P. et al., 2016). The supernatants were collected after centrifugation (10,000 *g*, 4°C, 10 min), filtered using 0.22-μm micron syringe filters (Millex-GP, United States, SLGP033RB), and stored in a fridge at -80°C.

#### Detection of Total Protein Concentration and IgA Concentration

The total protein concentration of *F. nucleatum*, *E. coli*, and FSN was determined by BCA assay (BCA Protein Assay kit, ABP Biosciences, United States). For quantification of total IgA in FSN, the ELISA assay (IgA Rat Uncoated ELISA Kit with Plates, IgA Human Uncoated ELISA Kit with Plates, Invitrogen, United States) was performed in a 96-well flat-bottom plate. Both of the BCA and ELISA assays were performed according to the manufacturer’s instructions.

#### Specific IgA Detection via Western Blotting

The process was based on the protocols described previously (Wang H. F. et al., 2016; Donaldson et al., 2018). *F. nucleatum* or *E. coli* was collected, resuspended in normal saline, and the total protein was extracted by hypothermic ultrasonification. The *F. nucleatum* and *E. coli* proteins were denatured and separated by 10% sodium dodecyl sulfate-polyacrylamide gel electrophoresis (SDS-PAGE). The proteins were then transferred to polyvinylidene difluoride (PVDF) membranes. After blocking with 5% non-fat milk at room temperature for 5 h, the membrane strips were incubated at 4°C for 20 h with FSN from either human or rats that had been diluted to a specific concentration in normal saline. After washing, the PVDF membrane strips were incubated with either horseradish peroxidase (HRP)-labeled goat anti-human IgA alpha chain (1:5,000, Abcam, United Kingdom, ab97215) or HRP-labeled goat anti-rat IgA alpha chain (1:5,000, Abcam, United Kingdom, ab97185) for 60 min at room temperature. Immobilon Western Chemiluminescent HRP Substrate (Millipore, United States, WBKLS0100) was used to detect the bands.

#### Antigen Investigation Using Mass Spectrometry

The proteins were extracted from the gels after SDS-PAGE (10%), and the samples were treated (Qin and Zhang, 2002), cleaned, and digested using modified trypsin (Promega, United States) in the digestion buffer (100 mM ammonium bicarbonate, pH 8.5). Peptides were extracted using acetonitrile and dried down completely in a SpeedVac device (Thermo, United States). The dried samples were subsequently redissolved in 2% acetonitrile, 97.5% water, and 0.5% formic acid solution. The protein solution sample was first reduced by DL-dithiothreitol (DTT), and all cysteine residues were alkylated using iodoacetamide and cleaned either with desalting columns or by ethanol precipitation. The samples were then digested with sequencing grade modified trypsin in digestion buffer. The dissolved peptide samples were then analyzed with a NanoLC-ESI-MS/MS system, and the mass spectrometric data was used to search the NCBI NR protein database, after which the relative abundances of the proteins were calculated (Griffin et al., 2010).

#### Antigen Identification Using Recombination

The following procedures were performed to confirm that FomA (a major outer membrane protein of *F. nucleatum*) was recognized by the specific IgA (Liu et al., 2010). The sequence of the *F. nucleatum* fomA gene (GenBank Accession Number: X72582) was amplified using a forward PCR primer (5′-TTTC ATATGGAAGTTATGCCTGCACCTAC-3′) containing an *Nde*I site (Thermo Scientific, United States, FD0583) and a reverse PCR primer (5′-CGGCTCGAGTTAAGTAACTTTCATACC AG-3′) containing a *Xho*I *site* (Thermo Scientific, United States, FD0694). The primers were synthesized by the Sangon Biotech Company (Shanghai, China). The generated fragment was inserted into a pETDuet-1 vector that was subsequently transferred into *E. coli* BL21 (DE3) and plated onto LB agar medium containing ampicillin (50 μg/ml). Single colonies were selected and cultured in LB broth at 37°C for 8 h with shaking. The culture was diluted (1:100) with LB broth and incubated at 37°C, and isopropyl-β-D-thiogalactoside (IPTG) (Solarbio, Beijing, China, I8070) was added at a final concentration of 1 mM when the optical density at 620 nm reached 0.5. After induction, bacteria were harvested by centrifugation (6,000 × *g*, 4°C, 5 min) and then washed twice with normal saline. The total protein was extracted by hypothermic ultrasonification, after which SDS-PAGE was used to assess FomA expression, and then the reaction between the recombinant FomA and specific IgA of *F. nucleatum* in FNS was detected by western blotting as described above.

### Statistics and Taxonomy Quantification

The Mann–Whitney non-parametric test, Kruskal–Wallis non-parametric test and Spearman correlation were used to assess differences among the groups and correlations between variables. SPSS (version 24.0, IBM, United States) was used to analyze data. A 95% confidence level, i.e., *P* < 0.05 was used.

The raw sequences were processed using Usearch version 8.1.1861_win32. The raw reads were dereplicated, and the singletons or chimeras were removed. The sequences were then clustered into operational taxonomic units (OTUs) based on a 97% similarity and analyzed using the RDP classifier Bayes algorithm to calculate the community composition at each taxonomic level. The OTU tables were imported into Mothur v 1.38.1 (Schloss et al., 2009) for the rarefaction curves and diversity (Shannon index) analyses. The specific taxa were identified by the linear discriminant analysis effect size (LEfSe) analysis with the value of the Kruskal–Wallis rank-sum test set to 0.05 and an LDA cutoff value of 2.5. The non-metric multi-dimensional scaling (NMDS) analysis and redundancy analysis (RDA) were performed in Mothur and plotted in R v 3.2.3 (R Core team, 2015).

## Results

### *Fusobacterium* Is a Microbial Biomarker of IBS-D

A total of 71 IBS-D patients and 39 HC were recruited. Demographics between IBS-D patients and HC were well balanced (Table 1).

**TABLE 1:** Characteristics of IBS-D patients and healthy controls.

	IBS-D patients (*n* = 71)	Healthy controls (*n* = 39)
Age [mean (SD)]	42.06 (10.94)	39.62 (12.82)
**Sex**		
Female, *n* (%)	27 (38.03)	16 (41.03)
Male, *n* (%)	44 (61.97)	23 (58.97)
BMI kg/m^2^ [mean (SD)]	23.63 (3.56)	23.93 (3.83)
IBS-SSS [mean (SD)]	289.58 (46.46)	–
SAS [mean (SD)]	50.38 (9.61)	–
SDS [mean (SD)]	48.79 (9.84)	–

*IBS-D, diarrhea predominant-irritable bowel syndrome; SD, standard deviation; BMI, body mass index; IBS-SSS, irritable bowel syndrome symptom severity scale; SAS, Self-rating Anxiety Scale; SDS, Self-rating Depression Scale.*

A total of 110 fecal samples were analyzed using a MiSeq PE 300, with 5,530,955 valid reads obtained. In the analysis, 4,657,712 reads were used after noise reduction and removal of low-quality reads and chimeras. Subsequently, the reads were analyzed using Usearch and the RDP database, resulting in the identification of 2,425 OTUs.

**FIGURE 2:**
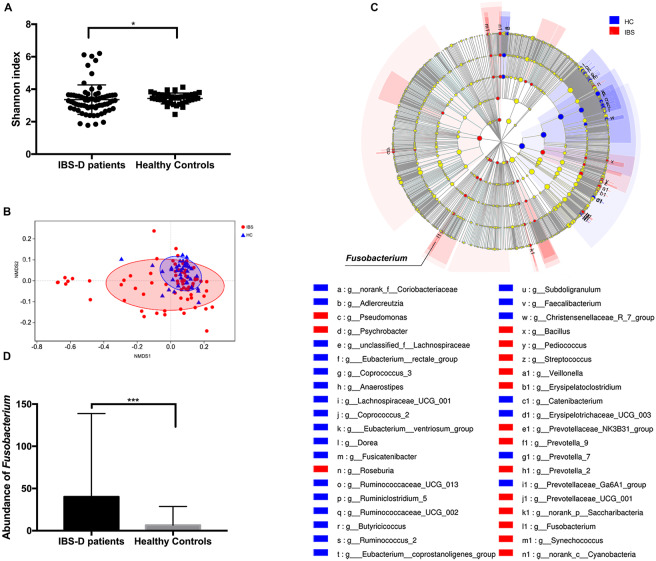
Intestinal microbiota characteristics in patients with diarrhea predominant-irritable bowel syndrome (IBS-D) and healthy controls (HC). **(A)** Shannon index between IBS-D patients and HC. **(B)** The non-metric multi-dimensional scaling (NMDS) analysis of IBS-D patients and HC. **(C)** The linear discriminant analysis effect size analysis with the value of the Kruskal–Wallis sum-rank test set to 0.05 and an LDA cutoff value of 2.5. **(D)** Abundance of *F. nucleatum* between IBS-D patients and HC. *P < 0.05, ***P < 0.001. IBS, IBS-D patients; HC, healthy controls.

The microbial diversity was compared by calculating the Shannon index. IBS-D patients showed a significantly lower Shannon index (*P* = 0.047, Mann–Whitney test) (Figure 2A). Furthermore, the microbial community structure was evaluated by NMDS analysis of beta diversity. IBS-D patients showed different microbial structures from healthy controls (*P* = 0.002, Adonis) (Figure 2B). Based on the observed OTU distributions, possible microbial biomarkers associated with IBS-D were determined by LEfSe analysis with the value of the Kruskal–Wallis rank-sum test set to 0.05 and an LDA cutoff value of 2.5 (Figure 2C, Supplementary Table 1 and Figure 1). The genus *Faecalibacterium*, genus *Ruminococcus*, genus *Subdoligranulum*, genus *Dorea*, etc., were highly enriched in HC, and in IBS-D patients, the genus *Prevotella*, genus *Streptococcus*, genus *Roseburia*, genus *Fusobacterium*, etc., were significantly enriched (Supplementary Table 1 and Figure 1). From these, all of the phylum *Fusobacteria*, class *Fusobacteria*, order *Fusobacteriales*, family *Fusobacteriaceae*, and genus *Fusobacterium* were shown to be possible microbial biomarkers of IBS-D patients (Figure 2C, Supplementary Table 1 and Figure 1). The abundance of *Fusobacterium* in the IBS-D patients was shown to remarkably higher than that observed in HC (*P* < 0.001, Mann–Whitney test) (Figure 2D). In addition, the abundance of *Fusobacterium* in IBS-D patients was positively correlated with SDS values (*P* = 0.034, rho = 0.252, Spearman correlation) (Supplementary Figure 2).

### *F. nucleatum* Causes the Intestinal Microbial Dysbiosis in Rats

A total of 96 fecal samples were analyzed using MiSeq PE 300, with 3,645,585 valid reads obtained. After noise reduction and removal of low-quality reads and chimeras, 3,008,922 reads were used. Subsequently, the reads were analyzed using the Usearch and the RDP database, with 1,239 OTUs obtained.

#### Microbial Diversity and NMDS Analysis

**FIGURE 3:**
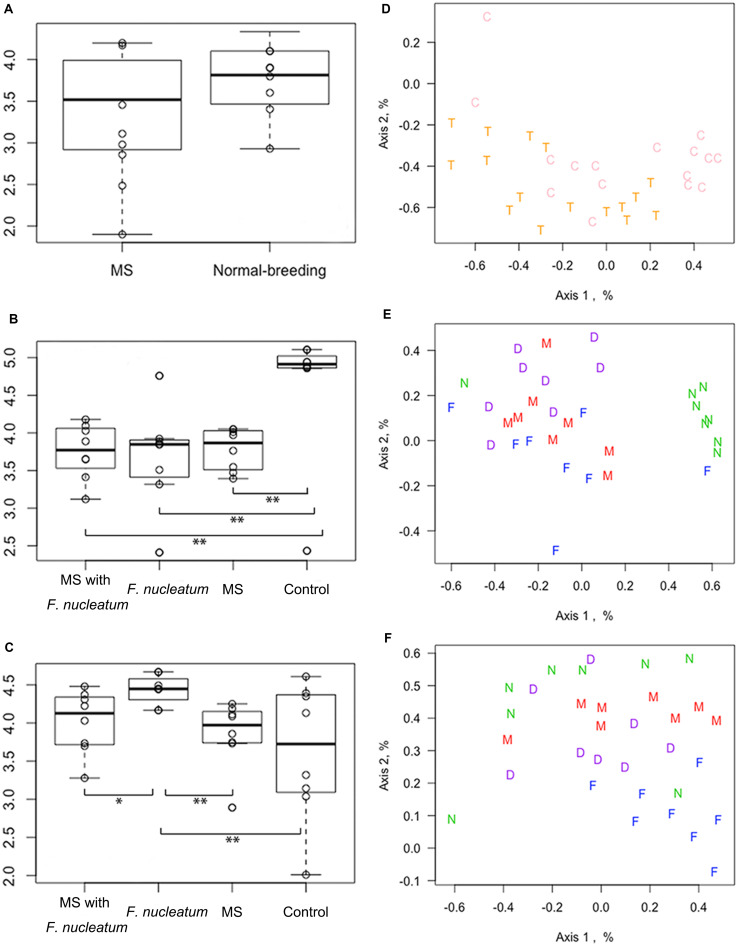
Microbial diversity and non-metric multi-dimensional scaling (NMDS) analysis. The diversity was analyzed by Shannon index at weeks 3 **(A)**, 8 **(B)**, and 12 **(C)**. The NMDS was analyzed at week 3 **(D)**, 8 **(E)**, and 12 **(F)**. *P < 0.05, **P < 0.01. MS, maternal separation; C, normal-breeding rats; T, MS rats; D, group MS with *F. nucleatum*; M, group MS; F, group *F. nucleatum*; N, control group.

The microbial diversity was compared using the Shannon index, Sobs value, and Chao_1 value. Results showed that at week 3, there was no significant difference of Shannon index between the MS rats and normal-breeding rats (χ^2^ = 2.27, *df* = 1, *P* = 0.13, KW) (Figure 3A). The Shannon index of the control group was higher than that of the other three groups at week 8 (χ^2^ = 10.2926, *df* = 3, *P* = 0.02, KW) (Figure 3B), while group *F. nucleatum* had the highest Shannon index at week 12 (χ^2^ = 10.1278, *df* = 3, *P* = 0.02, KW) (Figure 3C). Moreover, MS rats had a lower Sobs value than normal-breeding rats at week 3 (χ^2^ = 5.114, *df* = 1, *P* = 0.024, KW) (Supplementary Figure 3A). The control group showed a significantly higher Sobs value than the other three groups at week 8 (χ^2^ = 11.747, *df* = 3, *P* = 0.008, KW) (Supplementary Figure 3B), while there was no clear difference among the four groups at week 12 (χ^2^ = 0.054, *df* = 3, *P* = 0.997, KW) (Supplementary Figure 3C). For the Chao_1 value, no difference was found at week 3 (χ^2^ = 2.876, *df* = 1, *P* = 0.090, KW) (Supplementary Figure 3D), and all the three treatment groups had a lower Chao_1 value than the control group at week 8 (χ^2^ = 12.847, *df* = 3, *P* = 0.005, KW) (Supplementary Figure 3E), whereas at week 12, the difference among groups was unapparent (χ^2^ = 0.114, *df* = 3, *P* = 0.990, KW) (Supplementary Figure 3F). These results demonstrated that both the MS and *F. nucleatum*-gavage could decrease the diversity of intestinal microbiota, with the *F. nucleatum*-gavage having a particularly lasting effect.

The OTU data was clustered through an NMDS analysis. At week 3, no visible distinction was observed (Figure 3D). However, the control group had notably separated from the other three groups at week 8 (Figure 3E), although this separation was not obvious at week 12 (Figure 3F). Microbial dysbiosis was further evaluated by assessing the effect of treatments and cohousing using the Adonis and Anosim test (Supplementary Table 2). At week 3, the fecal samples of the MS rats were distinct from that of normal-breeding rats (*P* < 0.01). A significant difference also existed across the four groups at both weeks 8 and 12 (*P* < 0.001). No significant difference in cohousing was observed.

#### Reduced Colonization of Dominant OTUs

**FIGURE 4:**
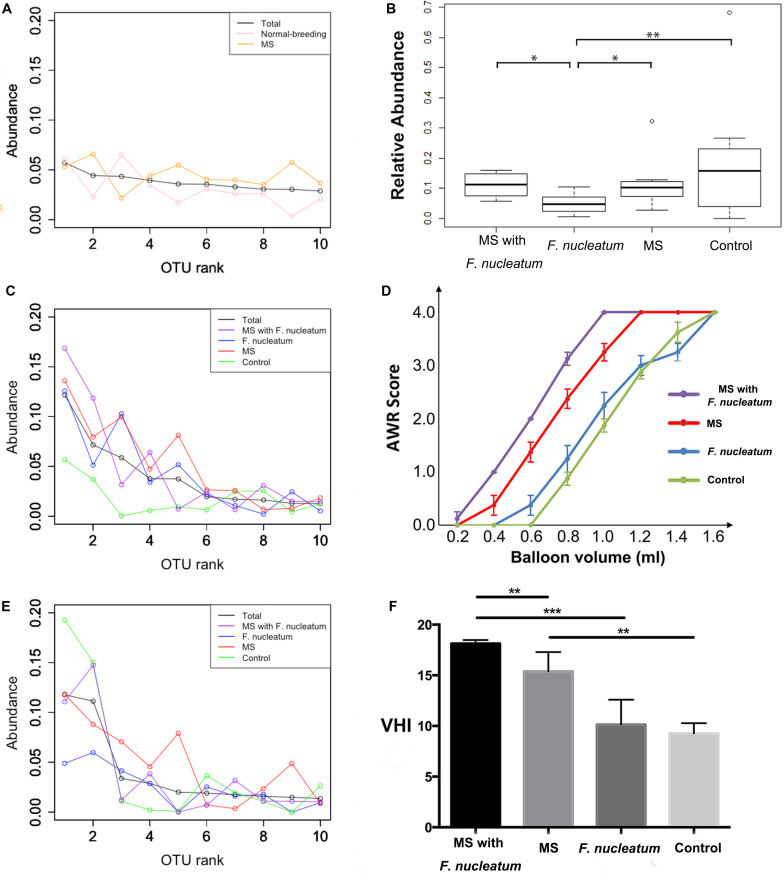
Rank-abundance curve and visceral sensitivity evaluation. For rank-abundance curves, the OTU ranks at the X-axis were ordered by the mean abundance of total samples, and the curve of each group was referred to the same ranks of total samples. Curves were plotted at weeks 3 **(A)**, 8 **(B)**, and 12 **(C)**. **(D)** The relative amount of the most abundant OTU at week 12. **(E)** Abdominal withdraw reflex (AWR) score. **(F)** Visceral hypersensitive index (VHI). *P < 0.05, **P < 0.01, ***P < 0.001. MS, maternal separation.

A reduced colonization of dominant OTUs was observed from the rank abundance curve. No notable differences were observed at week 3 (Figure 4A), whereas at week 8, the curve of the control group was distinct from those of the other groups (Figure 4B). Notably, the first two ranks of the group *F. nucleatum* were less than those of the other three groups at week 12 (Figure 4C). The relative amounts of the most abundant OTU at week 12 were then compared, showing that that of group *F. nucleatum* was lesser than those of the other groups (*P* = 0.01; *P* = 0.03; *P* = 0.008, Mann–Whitney test) (Figure 4D). This result indicated that the species in group *F. nucleatum* were more evenly distributed, consistent with the result that the diversity of group *F. nucleatum* was the highest at week 12 as described above.

### *F. nucleatum* Exacerbates Visceral Hypersensitivity in MS Rats

The visceral sensitivity of each group was evaluated by determining AWR scores based on graded CRD (Figure 4E). The VHI (Zhou et al., 2016) from the sum of the AWR scores at 0.4, 0.6, 0.8, 1.0, 1.2, and 1.4 ml were calculated (Figure 4F). Significant differences in the VHI values were observed among four groups (χ^2^ = 20.313, *df* = 8, *P* = 0.009, KW). Group MS with *F. nucleatum* and group MS had higher VHI values than group *F. nucleatum* and the control group (*P* < 0.001 and *P* = 0.001, respectively, Mann–Whitney test). The VHI of group MS with *F. nucleatum* was higher than that of group MS (*P* = 0.003, Mann–Whitney test). These results showed that *F. nucleatum*-gavage exacerbated visceral hypersensitivity in MS rats.

### *F. nucleatum* Does Not Colonize but Imprints the Immune System

#### Detection of Specific IgA Against *F. nucleatum* in Rat FSN and RDA

**FIGURE 5:**
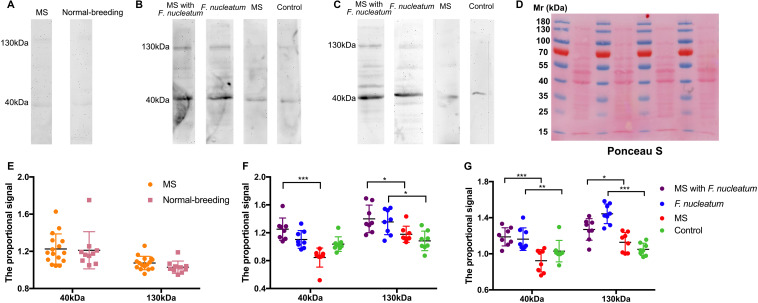
Detection of specific IgA against *F. nucleatum* in fecal supernatants (FSN) of rats. The western blotting was used to detect the IgA against *F. nucleatum* at weeks 3 **(A)**, 8 **(B)**, and 12 **(C)**. **(D)**, Ponceau S staining was used as loading control. The quantified proportional signals from reactive bands at 40 and 130 kDa were calculated at weeks 3 **(E)**, 8 **(F)**, and 12 **(G)**. *P < 0.05, **P < 0.01, ***P < 0.001. MS, maternal separation.

*F. nucleatum* was not detected from 16s rRNA gene sequencing or PCR (Supplementary Figure 4). Microbial antigens were previously shown to induce mucosal immunity where microbial neutralization was achieved by secretory IgA (Macpherson and Uhr, 2004; Konrad et al., 2006). No significant difference was tested in the total protein concentration of FSN among groups at week 3, week 8, or week 12 (Supplementary Figure 5), and for the IgA concentration in FSN, there was no significant difference among groups by time points (Supplementary Figure 6). Thus, *F. nucleatum*-specific IgA in the FSNs of rats was detected by western blotting. Ponceau S staining was used as loading control (Figure 5D). No obvious reactive band was observed at week 3 (Figure 5A), whereas two strong bands with molecular masses of 40 and 130 kDa were detected at week 8 in most samples of group MS with *F. nucleatum* and group *F. nucleatum*. In contrast, two weak bands at the same positions were detected in some of the samples from group MS and the control group (Figure 5B). These two bands were also observed in most samples from group MS with *F. nucleatum* and group *F. nucleatum* at week 12 but were almost invisible in group MS and the control group (Figure 5C). Additionally, the results of a parallel experiment showed that the bands observed using *E. coli* BL21 (DE3) occurred at different positions, confirming the specificity of IgA toward *F. nucleatum* (Figure 7H). The proportional signals of these two reactive bands reflected the levels of *F. nucleatum*-specific IgA in FSN. After quantifying and comparing the proportional signals of these two bands (Donaldson et al., 2018), no differences were observed in either the 130- or 40-kDa bands at week 3 (Figure 5E). At week 8, there were significant differences among groups at both 130 kDa (χ^2^ = 13.943, *df* = 3, *P* = 0.003, KW) and 40 kDa (χ^2^ = 22.259, *df* = 3, *P* < 0.001, KW) (Figure 5F), at week 12 these differences were still significantly different at both 130 kDa (χ^2^ = 21.838, *df* = 3, *P* < 0.001, KW) and 40 kDa (χ^2^ = 18.219, *df* = 3, *P* < 0.001, KW) (Figure 5G). Thus, *F. nucleatum* protein was shown to stimulate the production of specific IgA in *F. nucleatum*-gavaged rats, and this effect was relatively persistent.

**FIGURE 6:**
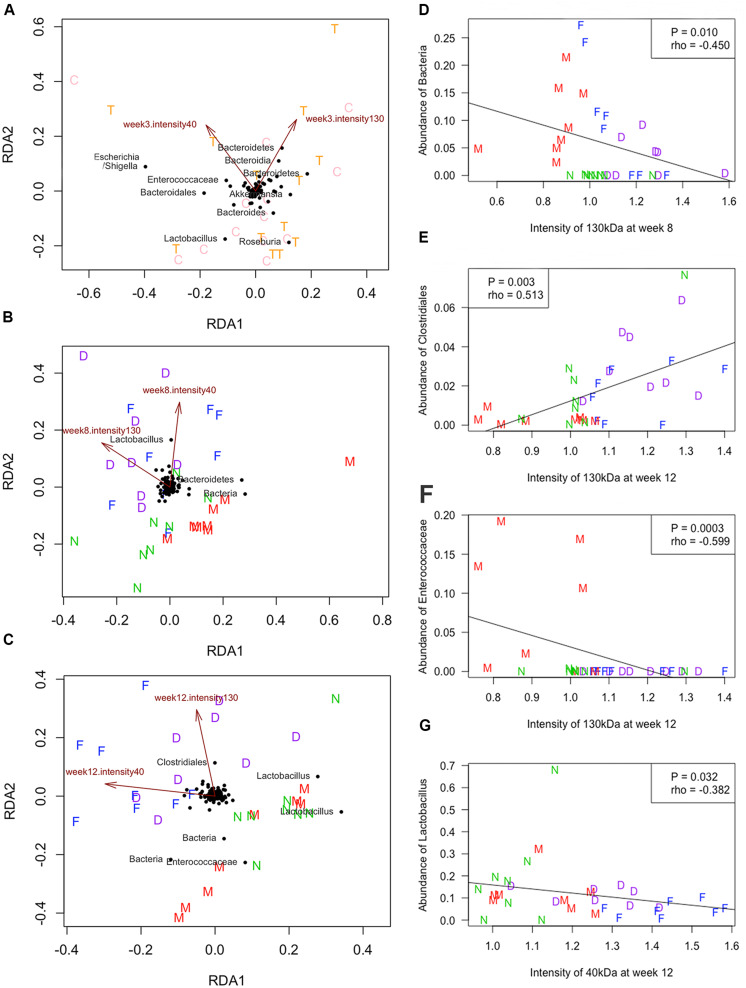
Redundancy analysis (RDA) and Spearman correlation. The RDA was performed at weeks 3 **(A)**, 8 **(B)**, and 12 **(C)**. The quantified proportional signals from reactive bands at 130 and 40 kDa were used as environmental variables. The Spearman correlation was calculated between the quantified proportional signals at 130 kDa and abundance of an unclassified OTU at week 8 **(D)**, the signals of 130 kDa and abundance of the OTU belonged to *Clostridiales* **(E)** or *Enterococcaceae* **(F)** at week 12, and signals of 40 kDa and abundance of the OTU belonged to *Lactobacillus* at week 12 **(G)**. C, normal-breeding rats; T, maternal separation (MS) rats; D, group MS with *F. nucleatum*; M, group MS; F, group *F. nucleatum*; N, control group.

The association between the levels of *F. nucleatum*-specific IgA in FSN and the microbial community was analyzed by RDA. As shown in Figure 6, the mean intensity showed the proportional signals, which at 130 and 40 kDa were crucial attributes that influenced the bacterial community. In particular, the mean intensity of the 130 and 40 kDa at weeks 8 and 12 was positively correlated with the microbial communities of group MS with *F. nucleatum* and group *F. nucleatum*, both of which received the *F. nucleatum*-gavage, whereas group MS and the control group exhibited a negative correlation (Figures 6B,C). The relationship between the levels of *F. nucleatum*-specific IgA in FSN and the abundance of specific bacterial taxa was analyzed. According to the Spearman correlation analysis, the mean intensity of the 130-kDa band at week 8 negatively correlated with the abundance of an unclassified OTU (*P* = 0.010, rho = -0.450, Figure 6D). The mean intensity of the 130-kDa band at week 12 correlated positively with the abundance of an OTU belonged to *Clostridiales* (*P* = 0.003, rho = 0.513, Figure 6E), but negatively correlated with the abundance of an OTU belonging to *Enterococcaceae* (*P* = 0.0003, rho = -0.599, Figure 6F). Additionally, the mean intensity of the 40-kDa band at week 12 negatively correlated with the abundance of an OTU belonging to *Lactobacillus* (*P* = 0.032, rho = -0.382, Figure 6G).

#### Detection of *F. nucleatum*-Specific IgA in Human FSN

To further confirm the association of *F. nucleatum*-specific IgA with IBS, the FSNs of seven IBS-D patients and five HC were investigated because of their sufficient fecal samples and complete clinical data. The patient characteristics are shown in Supplementary Table 3. No significant differences in age (*P* = 0.530, Mann–Whitney test) and body mass index (BMI) (*P* = 0.639, Mann–Whitney test) were observed. The IBS-D patients showed more notable anxiety and depression based on SAS (*P* = 0.003, Mann–Whitney test) and SDS (*P* = 0.003, Mann–Whitney test). The severity of intestinal symptoms was assessed using the IBS-SSS, with all IBS-D patients exhibiting severe IBS (total score >300).

**FIGURE 7:**
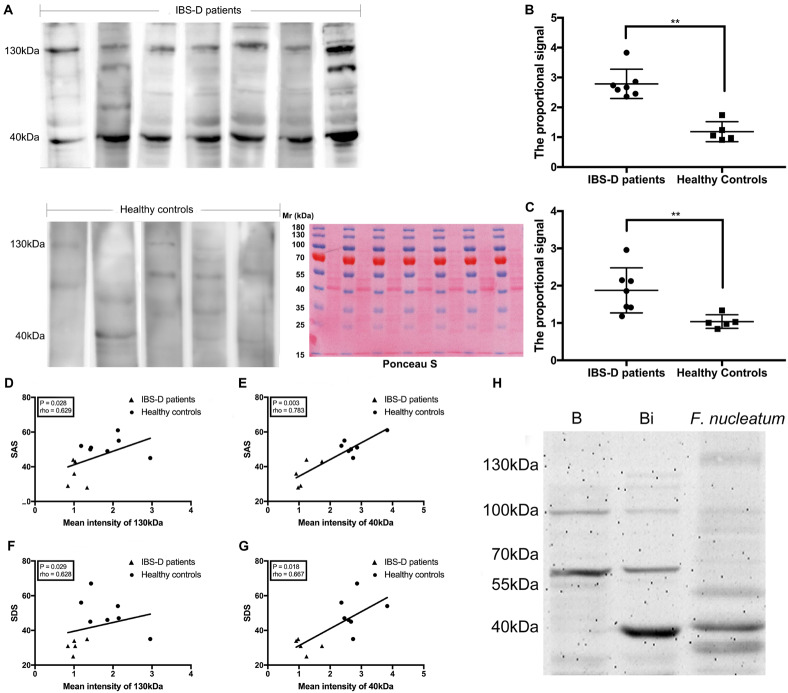
Detection of specific IgA against *F. nucleatum* in fecal supernatants (FSN) of patients and identification of FomA. The IgA against *F. nucleatum* was detected by western blotting, and Ponceau S staining was used as loading control **(A)**. The quantified proportional signals from reactive bands were evaluated at 40 **(B)** and 130 kDa **(C)**. The Spearman correlation was calculated between the Self-rating Anxiety Scale (SAS) and the quantified proportional signals from band at 130 **(D)** and 40 kDa **(E)**, Self-rating Depression Scale (SDS), and signals from band at 130 **(F)** and 40 kDa **(G)**. **(H)** The specific IgA in the FSN against *E. coli* BL21 (DE3) with or without expression of FomA gene. **P < 0.01. B: *E. coli* BL21 (DE3) without expression of FomA gene; Bi: *E. coli* BL21 (DE3) with expression of the FomA gene.

There was no significant difference between IBS-D patients and HC in neither FSN total protein concentration nor FSN IgA concentration (Supplementary Figure 7). The detection of *F. nucleatum*-specific IgA was performed by western blotting. Ponceau S staining was used as loading control (Figure 7A). Two strong reactive bands at 130 and 40 kDa were observed using the FSN of IBS-D patients, whereas the use of the FSN from HC produced bands with relatively weak intensities (Figure 7A). This result was similar to those observed using rats. The quantified proportional signals from reactive bands were significantly higher in IBS-D patients at both 40 kDa (*P* = 0.003, Mann–Whitney test) (Figure 7B) and 130 kDa (*P* = 0.005, Mann–Whitney test) (Figure 7C). Furthermore, SAS was positively correlated with the quantified proportional signal for both the 130-kDa (*P* = 0.028, rho = 0.629, Spearman correlation) (Figure 7D) and 40-kDa bands (*P* = 0.003, rho = 0.783, Spearman correlation) (Figure 7E). Similarly, SDS exhibited a positive correlation with both the signals of the 130-kDa (*P* = 0.029, rho = 0.628, Spearman correlation) (Figure 7F) and 40-kDa bands (*P* = 0.018, rho = 0.667, Spearman correlation) (Figure 7G). Therefore, the specific IgA against *F. nucleatum* was suggested as a symptom-associated antibody in IBS-D patients.

#### Identification of a *F. nucleatum*-Specific Antigen

A proteomic analysis of *F. nucleatum* was performed by combining the protein molecular weight and functional category annotated in eggNOG. By filtering the results, 57 proteins in the M category (cell wall/membrane/envelope biogenesis) (Singer and Nicolson, 1972; Hedin et al., 2011) with an approximate molecular mass of 40 kDa and none at 130 kDa were filtered out. Next, the two strongly reactive bands at 40 and 130 kDa were cut out of the gels and sent for mass spectrometry. The sequence coverage of all the proteins identified by mass spectrometry is shown in the Supplementary Table 4. Also, six major proteins were identified. Of these, three belonged to the band at 40 kDa and the other three belonged to that at 130 kDa (Table 2), which were identified by NanoLC-ESI-MS/MS. Among these proteins, FomA, which is a major outer membrane protein of *F. nucleatum*, appeared in both and belonged to the proteins in the M category selected above. Thus, FomA was identified as a possible key protein eliciting the immune response.

**TABLE 2:** Protein identified of interest proteins.

Band	Protein information			Protein mass^(B)^	Gene	No. of peptide^(C)^	Relative abundance (%)	Organism
	Rank	Name	Accession no.^(A)^					
130 kDa	1	Acyl-CoA dehydrogenase C-terminal domain protein	EFG95074.1	41898.5	bcd2	50	10.8	*Fusobacterium nucleatum*
	2	Porin	CAA51172.1	42346.27	fomA	69	9.2	*Fusobacterium nucleatum*
	3	Pyruvate-flavodoxin oxidoreductase	AAL95366.1	132250.47	FN1170	92	8.5	*Fusobacterium nucleatum*
40 kDa	1	Porin	CAA51172.1	42346.27	fomA	368	17.5	*Fusobacterium nucleatum*
	2	Acyl-CoA dehydrogenase C-terminal domain protein	EFG95074.1	41898.5	bcd2	218	11.6	*Fusobacterium nucleatum*
	3	Electron transf flavoprotein alpha-submit	AAL94981.1	42298.47	FN0785	177	9.4	*Fusobacterium nucleatum*

*^(A)^Protein accession number on NCBI. ^(B)^The calculated molecular weight of each identified protein based on its amino acid sequence present in the current NR database. ^(C)^The number of peptides sequenced by LC-MS/MS in each identified protein.*

To test this hypothesis, the fomA gene was recombined into an *E. coli* strain harboring the expression vector. A strong reactive band with a molecular mass of 40 kDa was observed in the western blotting test using the recombinant FomA and the *F. nucleatum*-reactive FSN, whereas no band was observed at 40 kDa using the FomA-negative *E. coli* (Figure 7H). The strong band at 40 kDa was then cut and sent for mass spectrometry, the results of which further confirmed that FomA was expressed at 40 kDa of recombinant *E. coli* proteins (Table 3). Other details of mass spectrometry are shown in the Supplementary Table 4. Thus, these results collectively suggested that FomA was the antigen recognized by the symptom-associated antibody.

**TABLE 3:** Protein identified of recombinant proteins.

Band	Protein information			Protein mass^(B)^	Gene	No. of peptide^(C)^	Relative abundance (%)	Organism
	Rank	Name	Accession no.^(A)^					
*E. coli*	1	Porin	CAA51172.1	42346.27	fomA	490	37.56	*Fusobacterium nucleatum*
BL21(DE3)	2	Porin Gram-negative type	ACT29688.1	39309.08	ECBD_2666	168	15.59	*Escherichia coli* (strain B/BL21-DE3)
FomA	3	Phosphoglycerate kinase	CQR82365.1	41263.73	pgk	77	6.63	*Escherichia coli* (strain B/BL21-DE3)

*^(A)^Protein accession number on NCBI. ^(B)^The calculated molecular weight of each identified protein based on its amino acid sequence present in the current NR database. ^(C)^The number of peptides sequenced by LC-MS/MS in each identified protein.*

## Discussion

According to our findings, the genus *Fusobacterium* remarkably increased in IBS-D. *F. nucleatum* could cause intestinal microbial dysbiosis and exacerbate visceral hypersensitivity in MS rats. The oral administration of *F. nucleatum* activated the secretion of specific secretory IgA that acted against it, while *F. nucleatum*-specific IgA was also detected in the FSN of IBS-D patients. FomA was further identified as the target antigen that bound to the *F. nucleatum*-specific IgA.

Microbial dysbiosis is closely associated with the occurrence and development of IBS (Ghoshal et al., 2012; Bennet et al., 2015). Some characteristics, such as an increased ratio of *Firmicutes* to *Bacteroides* and decreased bacterial diversity, have been observed in IBS patients (Simren et al., 2013; Jalanka-Tuovinen et al., 2014). However, there are still controversies regarding the characteristic bacteria in the intestinal microbiota of IBS patients. In this study, it was first revealed that the abundance of *Fusobacterium* was observed to be significantly higher and be positively correlated with SDS in IBS-D patients. Therefore, *Fusobacterium* was shown to be involved in the pathophysiology of IBS, especially with respect to mental and sensory abnormalities.

Because *Fusobacterium* was obviously enriched in IBS-D, *F. nucleatum* was chosen to administer to rats. Both MS and gavage-introduced *F. nucleatum* were observed to reduce microbial diversity. The results from the NMDS analysis also indicated the separation between the control and the treatment rats. In addition, gavage-introduced *F. nucleatum* was observed to exacerbate the visceral hypersensitivity of MS rats. Visceral hypersensitivity is considered to be an important feature of IBS (Farzaei et al., 2016; Simren et al., 2017). However, the relationship between intestinal microbiota and visceral hypersensitivity still remains unclear. As a member of the genus *Fusobacterium*, *F. nucleatum* has been demonstrated to promote the release of intestinal inflammatory factors and destroy the intestinal barrier function (Liu et al., 2007; Han, 2015; Tang et al., 2016) and is associated with oral pain and cold sensitivity (Hahn et al., 1993; Massey et al., 1993). After the gavage of *F. nucleatum*, the decrease in the genus *Lactobacillus* was detected. *Lactobacillus* is a type of probiotic that is used to regulate the intestinal microbiota of IBS (Niedzielin et al., 2001; Ducrotte et al., 2012). Probiotic treatment with *Lactobacillus* was suggested to prevent visceral hypersensitivity to colonic inflammation and an acute psychological stress (Darbaky et al., 2017). The phylum *Firmicutes* and the class *Clostridia* were also shown be present at higher levels after the gavage of *F. nucleatum*, and an increase in *Firmicutes* and some *Clostridium* spp. has been noted in IBS patients (Matto et al., 2005; Krogius-Kurikka et al., 2009). Therefore, the intestinal microbial dysbiosis caused by *F. nucleatum* might be related to visceral hypersensitivity.

Although *F. nucleatum* supposedly caused intestinal microbiota dysbiosis and exacerbated visceral hypersensitivity in rats, the strain was not detected in fecal samples of rats by 16s rRNA sequencing or PCR. To identify the mechanism of the colonization-independent process, the specific IgA against *F. nucleatum* in the FSN was evaluated. At weeks 8 and 12, the levels of *F. nucleatum*-specific IgA in FSN of rats increased; similarly, that in the FSN of IBS-D patients was also increased. Moreover, the levels of *F. nucleatum*-specific IgA in the FSN of the IBS-D patients and HC were found to be positively correlated with the SAS and SDS. When microorganisms enter the intestine, mucosal immunity is shown to be activated by microbial antigens and induce secretory IgA release for neutralization (Macpherson and Uhr, 2004; Konrad et al., 2006). Although *F. nucleatum* was not colonized in rats, its specific IgA was observed to be a crucial factor influencing the bacterial community by RDA analysis. These results suggest that the production of *F. nucleatum*-specific IgA may play a role in the pathogenesis of IBS. Moreover, FomA (Nakagaki et al., 2010), encoded by the fomA gene (Toussi et al., 2012), was identified as the molecular antigen stimulating the production of specific IgA in the intestine. FomA is a major outer membrane protein of *F. nucleatum* (Nakagaki et al., 2010). Membrane proteins have been shown to play crucial roles in bioenergetics, transport, and signaling (Singer and Nicolson, 1972; Hedin et al., 2011), and more than half of the known drugs target membrane proteins (Sojo et al., 2016). Furthermore, FomA is a Toll-like receptor 2 agonist with an adjuvant immune activity that stimulates the production of IgA and IgG (Toussi et al., 2012; Ma et al., 2013), while it is also capable of recruiting oral bacteria (Liu et al., 2010). Interestingly, the oral administration of recombinant FomA to mice was shown to increase IgG in serum and IgA in saliva and reduce the risk of periodontal infection of *F. nucleatum* (Ma et al., 2013). Therefore, these results suggest that *F. nucleatum* can activate intestinal immunity via FomA to promote the secretion of high quantities of IgA that prevent its colonization.

Despite the useful findings, there are some limitations to this study. First, whether or not other strains of the *Fusobacterium* would have a similar effect was not determined. Second, although *F. nucleatum* was observed to be capable of inducing the secretion of IgA, its specific immune mechanism was not explored and requires further research.

In conclusion, *Fusobacterium* was observed to be an increased genus with significantly higher abundance in IBS-D patients. Moreover, the mechanism of *F. nucleatum* in the pathogenesis of IBS through causing microbial dysbiosis and exacerbating visceral hypersensitivity in a colonization-independent manner was confirmed. Furthermore, FomA was identified to be the antigen stimulating the production of the symptom-associated antibody.

## Data Availability Statement

The datasets generated for this study can be found in the National Center for Biotechnology Information (NCBI) database with accession code PRJNA511737, National Center for Biotechnology Information (NCBI) database with accession code PRJNA511738.

## Ethics Statement

The studies involving human participants were reviewed and approved by the ethics committees in Qilu Hospital, Shandong University. The patients/participants provided their written informed consent to participate in this study. The animal study was reviewed and approved by Ethical and Institutional Animal Care and Use Committee of Qilu Hospital, Shandong University.

## Author Contributions

XG, ML, and XZ designed the project. ML conducted the bioinformatic data mining. XG, LS, TL, and MZ conducted key experiments. XG and ML performed statistical analysis. LL, ZL, and PW provided vital expertise and advice. XG and LS wrote the manuscript. All authors read the manuscript and approved the final draft that was submitted.

## Conflict of Interest

The authors declare that the research was conducted in the absence of any commercial or financial relationships that could be construed as a potential conflict of interest.
