# Supplementary material for: Fusobacterium nucleatum Causes Microbial Dysbiosis and Exacerbates Visceral Hypersensitivity in a Colonization-Independent Manner
Source: Front Microbiol. 2020 Jun 24;11:1281. doi: 10.3389/fmicb.2020.01281 (PMC7358639; doi:10.3389/fmicb.2020.01281)
Supplement: Supplementary file 1 [file Data_Sheet_1.pdf]

Supplementary Material

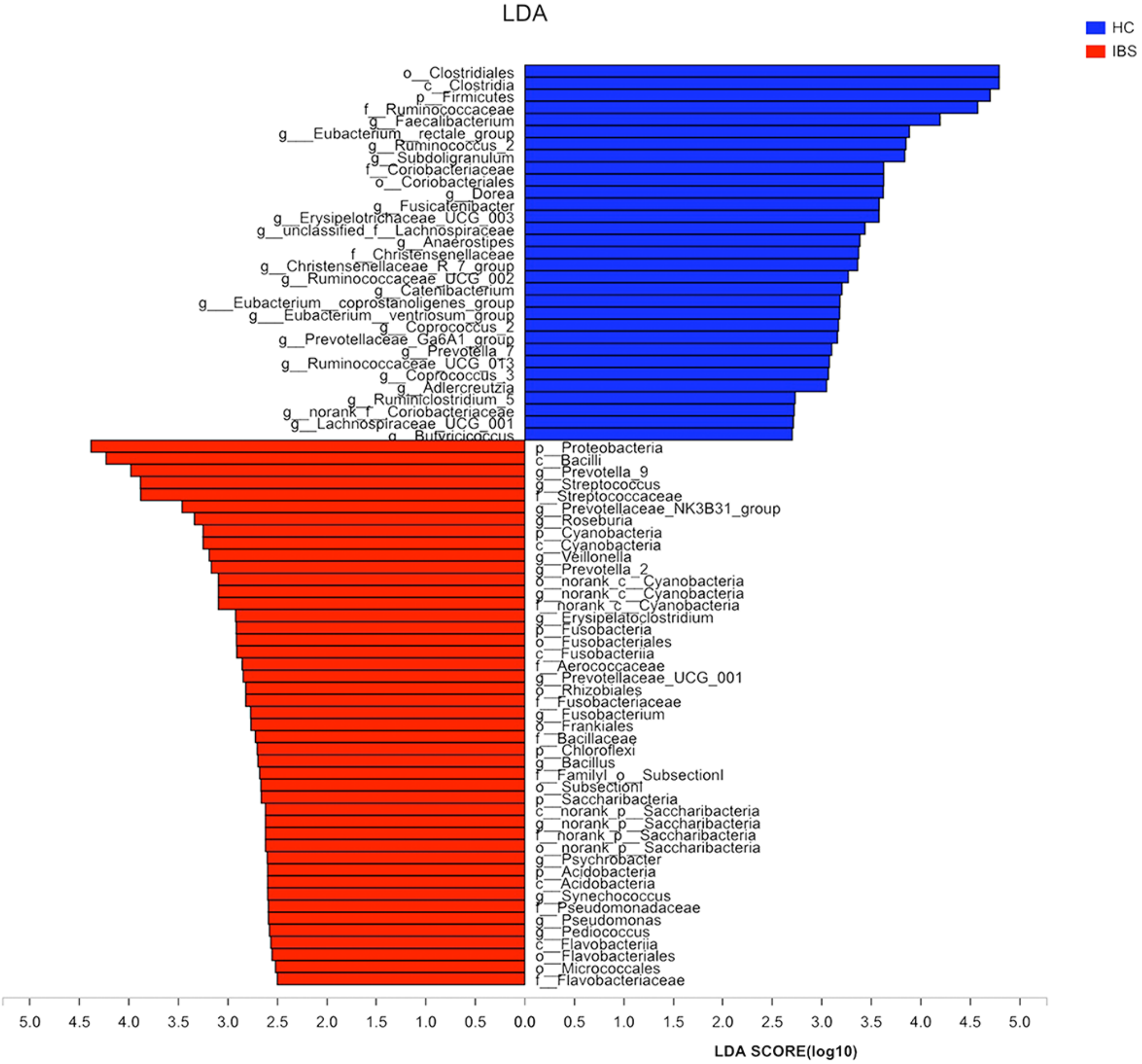

Supplementary Figure 1. Taxonomic difference based on LDA**.** IBS, IBS-D patients; HC, healthy controls

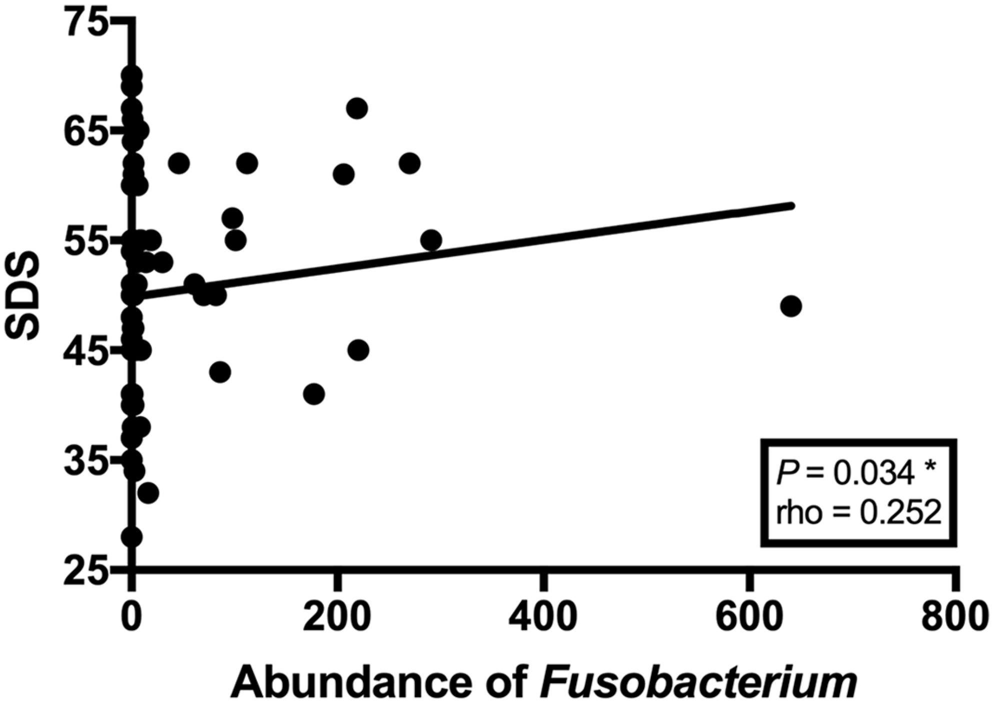

Supplementary Figure 2. The Spearman correlation between abundance of *Fusobacterium* and Self-rating Depression Scale (SDS) in IBS-D patients.

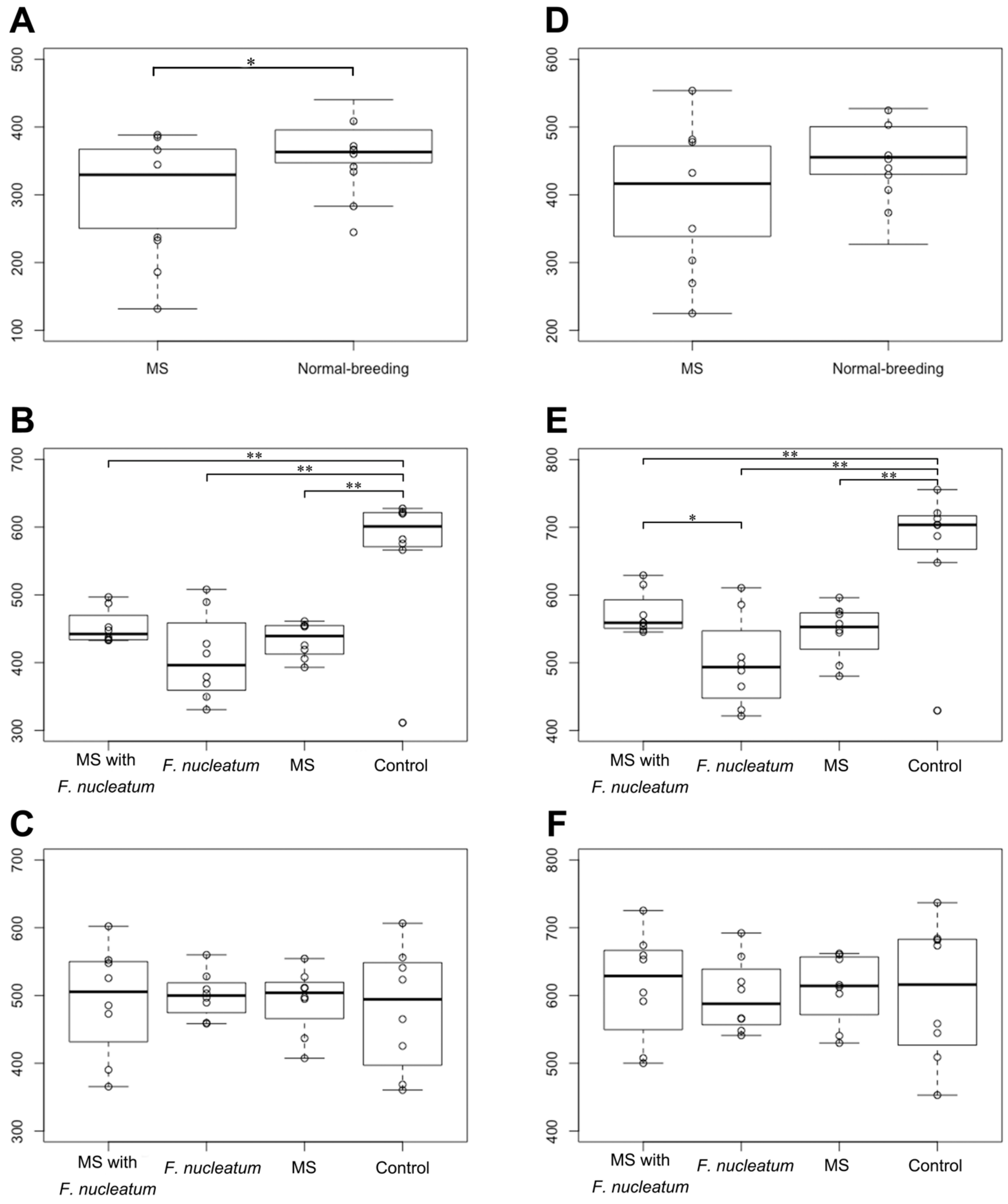

Supplementary Figure 3. The Sobs value at at week3 (**A**), 8 (**B**), and 12 (**C**), and the Chao_1 value at week3 (**D**), 8 (**E**), and 12 (**F**). *: $P < 0.05$, **: $P < 0.01$. MS: maternal separation.

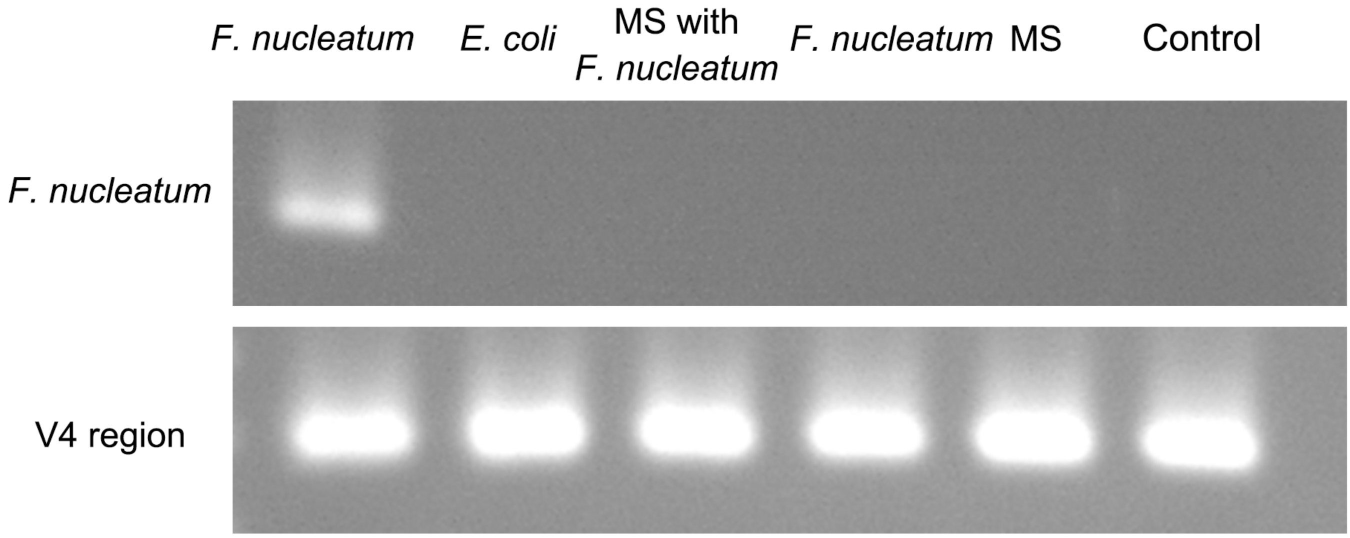

Supplementary Figure 4. Detection of *F. nucleatum*. *F. nucleatum* was detected by polymerase chain reaction in stool of rats in 4 groups, and *F. nucleatum* (ATCC25586) and *E. coli* BL21 (DE3) were used as positive and negative controls, respectively. The V4 region of the bacterial 16S rRNA gene was amplified as internal control. MS: maternal separation.

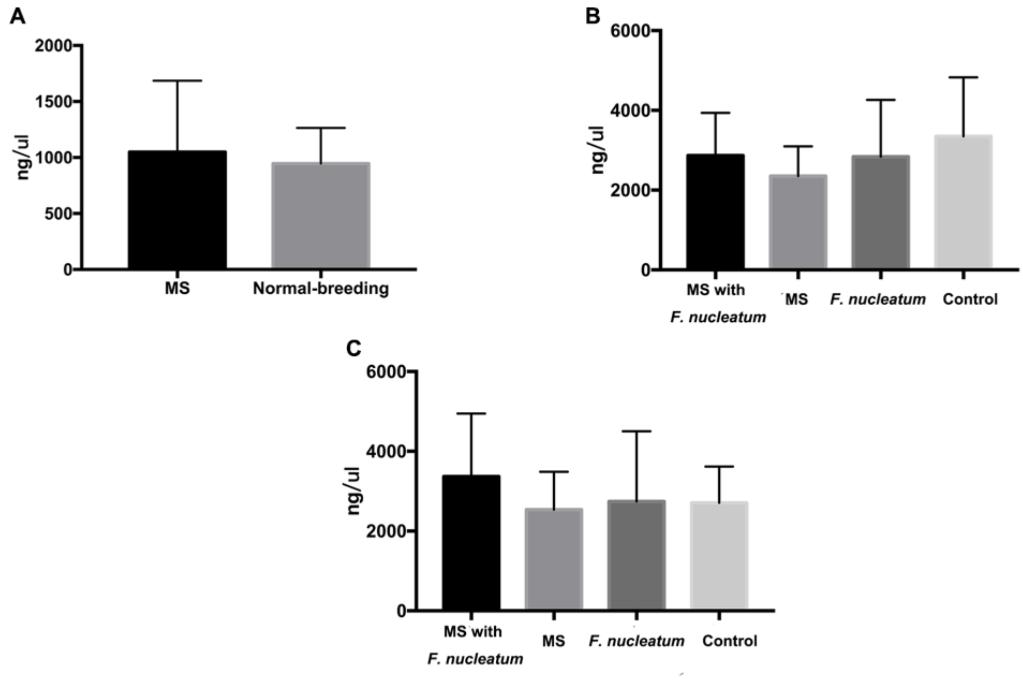

Supplementary Figure 5. The total protein concentration of rats' FSN was tested by BCA assay at week 3 (**A**), week 8 (**B**), week 12 (**C**). MS: maternal separation.

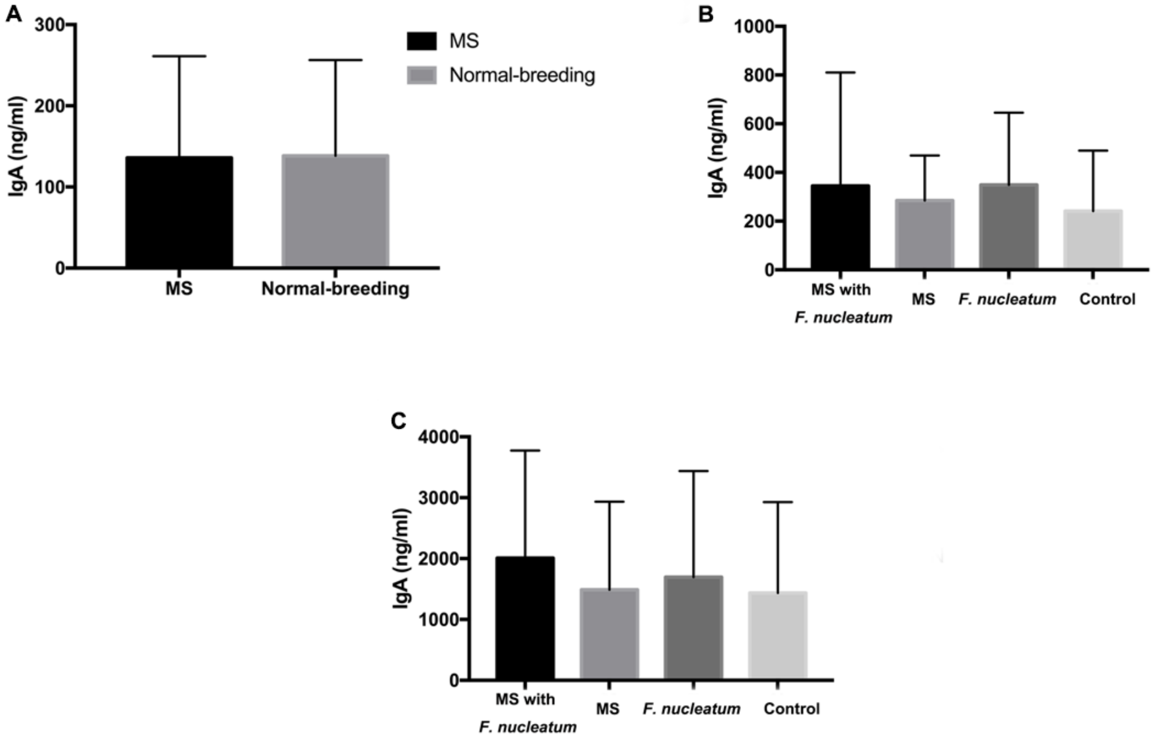

Supplementary Figure 6. The concentration of IgA in rats' FSN was tested by ELISA kits at week 3 (**A**), week 8 (**B**), week 12 (**C**). MS: maternal separation.

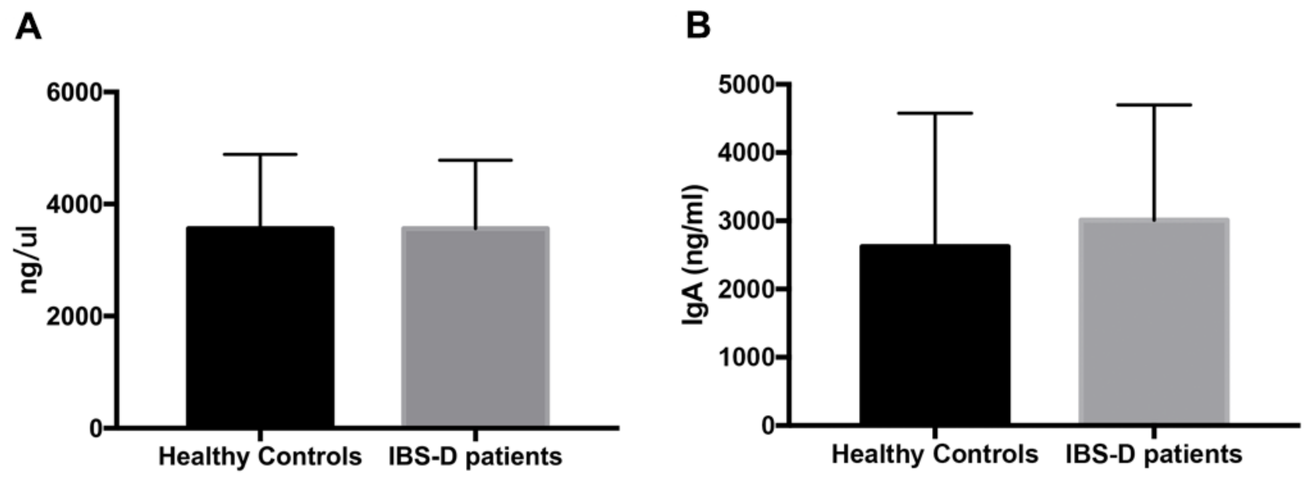

Supplementary Figure 7. The total protein concentration (**A**) and IgA concentration (**B**) in FSN of IBS-D patients and healthy controls.

Supplementary Table 1. Taxonomic difference

Taxonomy	number	group	LDA	*P* value
p__Acidobacteria		IBS-D	2.5996	0.0003
p__Acidobacteria.c__Acidobacteria		IBS-D	2.5995	0.0003
p__Actinobacteria.c__Actinobacteria.o__Frankiales		IBS-D	2.7683	0.0348
p__Actinobacteria.c__Actinobacteria.o__Micrococcales		IBS-D	2.5205	0.0032
p__Bacteroidetes.c__Bacteroidia.o__Bacteroidales.f__Prevotellaceae.g__Prevotella_2		IBS-D	3.1689	0.0203
p__Bacteroidetes.c__Bacteroidia.o__Bacteroidales.f__Prevotellaceae.g__Prevotella_9		IBS-D	3.9821	0.0163
p__Bacteroidetes.c__Bacteroidia.o__Bacteroidales.f__Prevotellaceae.g__Prevotellaceae_NK3B31_group	n = 45	IBS-D	3.4647	0.0002
p__Bacteroidetes.c__Bacteroidia.o__Bacteroidales.f__Prevotellaceae.g__Prevotellaceae_UCG_001		IBS-D	2.8477	0.0117
p__Bacteroidetes.c__Flavobacteriia		IBS-D	2.5679	0.0071
p__Bacteroidetes.c__Flavobacteriia.o__Flavobacteriales		IBS-D	2.5553	0.0071
p__Bacteroidetes.c__Flavobacteriia.o__Flavobacteriales.f__Flavobacteriaceae		IBS-D	2.5020	0.0039
p__Chloroflexi		IBS-D	2.7055	0.0003
p__Cyanobacteria		IBS-D	3.2524	0.0023
p__Cyanobacteria.c__Cyanobacteria		IBS-D	3.2524	0.0023

p__Cyanobacteria.c__Cyanobacteria.o__norank_c__Cyanobacteria	IBS-D	3.0970	0.0036
p__Cyanobacteria.c__Cyanobacteria.o__norank_c__Cyanobacteria.f__norank_c__Cyanobacteria	IBS-D	3.0970	0.0036
p__Cyanobacteria.c__Cyanobacteria.o__norank_c__Cyanobacteria.f__norank_c__Cyanobacteria.g__norank_c__Cyanobacteria	IBS-D	3.0970	0.0036
p__Cyanobacteria.c__Cyanobacteria.o__SubsectionI	IBS-D	2.6677	0.0086
p__Cyanobacteria.c__Cyanobacteria.o__SubsectionI.f__FamilyI_o__SubsectionI	IBS-D	2.6807	0.0086
p__Cyanobacteria.c__Cyanobacteria.o__SubsectionI.f__FamilyI_o__SubsectionI.g__Synechococcus	IBS-D	2.5994	0.0119
p__Firmicutes.c__Bacilli	IBS-D	4.2329	0.0487
p__Firmicutes.c__Bacilli.o__Bacillales.f__Bacillaceae	IBS-D	2.7241	0.0104
p__Firmicutes.c__Bacilli.o__Bacillales.f__Bacillaceae.g__Bacillus	IBS-D	2.6981	0.0049
p__Firmicutes.c__Bacilli.o__Lactobacillales.f__Aerococcaceae	IBS-D	2.8571	0.0187
p__Firmicutes.c__Bacilli.o__Lactobacillales.f__Lactobacillaceae.g__Pediococcus	IBS-D	2.5819	0.0006
p__Firmicutes.c__Bacilli.o__Lactobacillales.f__Streptococcaceae	IBS-D	3.8833	0.0452
p__Firmicutes.c__Bacilli.o__Lactobacillales.f__Streptococcaceae.g__Streptococcus	IBS-D	3.8856	0.0171
p__Firmicutes.c__Clostridia.o__Clostridiales.f__Lachnospiraceae.g__Roseburia	IBS-D	3.3389	0.0255
p__Firmicutes.c__Erysipelotrichia.o__Erysipelotrichales.f__Erysipelotrichaceae.g__Erysipelatoclostridium	IBS-D	2.9235	0.0129

p__Firmicutes.c__Negativicutes.o__Selenomonadales.f__Veillonellaceae.g__Veillonella	IBS-D	3.1906	0.0388
p__Fusobacteria	IBS-D	2.9168	0.0001
p__Fusobacteria.c__Fusobacteriia	IBS-D	2.9115	0.0001
p__Fusobacteria.c__Fusobacteriia.o__Fusobacteriales	IBS-D	2.9156	0.0001
p__Fusobacteria.c__Fusobacteriia.o__Fusobacteriales.f__Fusobacteriaceae	IBS-D	2.8204	0.0001
p__Fusobacteria.c__Fusobacteriia.o__Fusobacteriales.f__Fusobacteriaceae.g__Fusobacterium	IBS-D	2.7721	0.0001
p__Proteobacteria	IBS-D	4.3849	0.0377
p__Proteobacteria.c__Alphaproteobacteria.o__Rhizobiales	IBS-D	2.8208	0.0258
p__Proteobacteria.c__Gammaproteobacteria.o__Pseudomonadales.f__Moraxellaceae.g__Psychrobacter	IBS-D	2.6042	0.0454
p__Proteobacteria.c__Gammaproteobacteria.o__Pseudomonadales.f__Pseudomonadaceae	IBS-D	2.5929	0.0012
p__Proteobacteria.c__Gammaproteobacteria.o__Pseudomonadales.f__Pseudomonadaceae.g__Pseudomonas	IBS-D	2.5927	0.0012
p__Saccharibacteria	IBS-D	2.6644	0.0229
p__Saccharibacteria.c__norank_p__Saccharibacteria	IBS-D	2.6222	0.0045
p__Saccharibacteria.c__norank_p__Saccharibacteria.o__norank_p__Saccharibacteria	IBS-D	2.6222	0.0045
p__Saccharibacteria.c__norank_p__Saccharibacteria.o__norank_p__Saccharibacteria.f__norank_p__Saccharibacteria	IBS-D	2.6222	0.0045

p__Saccharibacteria.c__norank_p__Saccharibacteria.o__norank_p__Saccharibacteria.f__norank_p__Saccharibacteria.g__norank_p__Saccharibacteria		IBS-D	2.6222	0.0045
p__Actinobacteria.c__Actinobacteria.o__Coriobacteriales		HC	3.6264	0.0196
p__Actinobacteria.c__Actinobacteria.o__Coriobacteriales.f__Coriobacteriaceae		HC	3.6264	0.0196
p__Actinobacteria.c__Actinobacteria.o__Coriobacteriales.f__Coriobacteriaceae.g__Adlercreutzia		HC	3.0496	0.0000
p__Actinobacteria.c__Actinobacteria.o__Coriobacteriales.f__Coriobacteriaceae.g__norank_f__Coriobacteriaceae		HC	2.7223	0.0005
p__Bacteroidetes.c__Bacteroidia.o__Bacteroidales.f__Prevotellaceae.g__Prevotella_7		HC	3.1017	0.0324
p__Bacteroidetes.c__Bacteroidia.o__Bacteroidales.f__Prevotellaceae.g__Prevotellaceae_Ga6A1_group		HC	3.1598	0.0158
p__Firmicutes	n = 31	HC	4.7026	0.0113
p__Firmicutes.c__Clostridia		HC	4.7931	0.0044
p__Firmicutes.c__Clostridia.o__Clostridiales		HC	4.7939	0.0044
p__Firmicutes.c__Clostridia.o__Clostridiales.f__Christensenellaceae		HC	3.3738	0.0005
p__Firmicutes.c__Clostridia.o__Clostridiales.f__Christensenellaceae.g__Christensenellaceae_R_7_group		HC	3.3640	0.0024
p__Firmicutes.c__Clostridia.o__Clostridiales.f__Lachnospiraceae.g___Eubacterium__rectale_group		HC	3.8865	0.0323
p__Firmicutes.c__Clostridia.o__Clostridiales.f__Lachnospiraceae.g___Eubacterium__ventriosum_group		HC	3.1814	0.0031

p__Firmicutes.c__Clostridia.o__Clostridiales.f__Lachnospiraceae.g__Anaerostipes	HC	3.3871	0.0121
p__Firmicutes.c__Clostridia.o__Clostridiales.f__Lachnospiraceae.g__Coprococcus_2	HC	3.1698	0.0449
p__Firmicutes.c__Clostridia.o__Clostridiales.f__Lachnospiraceae.g__Coprococcus_3	HC	3.0676	0.0141
p__Firmicutes.c__Clostridia.o__Clostridiales.f__Lachnospiraceae.g__Dorea	HC	3.6227	0.0057
p__Firmicutes.c__Clostridia.o__Clostridiales.f__Lachnospiraceae.g__Fusicatenibacter	HC	3.5809	0.0005
p__Firmicutes.c__Clostridia.o__Clostridiales.f__Lachnospiraceae.g__Lachnospiraceae_UCG_001	HC	2.7129	0.0094
p__Firmicutes.c__Clostridia.o__Clostridiales.f__Lachnospiraceae.g__unclassified_f__Lachnospiraceae	HC	3.4379	0.0054
p__Firmicutes.c__Clostridia.o__Clostridiales.f__Ruminococcaceae	HC	4.5771	0.0002
p__Firmicutes.c__Clostridia.o__Clostridiales.f__Ruminococcaceae.g___Eubacterium__coprostanoligenes_group	HC	3.1856	0.0081
p__Firmicutes.c__Clostridia.o__Clostridiales.f__Ruminococcaceae.g__Butyricicoccus	HC	2.7032	0.0142
p__Firmicutes.c__Clostridia.o__Clostridiales.f__Ruminococcaceae.g__Faecalibacterium	HC	4.1954	0.0015
p__Firmicutes.c__Clostridia.o__Clostridiales.f__Ruminococcaceae.g__Ruminiclostridium_5	HC	2.7324	0.0239
p__Firmicutes.c__Clostridia.o__Clostridiales.f__Ruminococcaceae.g__Ruminococcaceae_UCG_002	HC	3.2691	0.0222
p__Firmicutes.c__Clostridia.o__Clostridiales.f__Ruminococcaceae.g__Ruminococcaceae_UCG_013	HC	3.0788	0.0008
p__Firmicutes.c__Clostridia.o__Clostridiales.f__Ruminococcaceae.g__Ruminococcus_2	HC	3.8519	0.0120

p__Firmicutes.c__Clostridia.o__Clostridiales.f__Ruminococcaceae.g__Subdoligranulum	HC	3.839<br>7	0.0097
p__Firmicutes.c__Erysipelotrichia.o__Erysipelotrichales.f__Erysipelotrichaceae.g__Catenibacterium	HC	3.204<br>7	0.0364
p__Firmicutes.c__Erysipelotrichia.o__Erysipelotrichales.f__Erysipelotrichaceae.g__Erysipelotrichaceae_UCG_003	HC	3.578<br>7	0.0267

IBS-D, diarrhea predominant-irritable bowel syndrome; HC, healthy controls.

Supplementary Table 2. Adonis and Anosim test on animal treatments and co-housing effect

**Time**	**Methods**	**Across**	**Co-housing**			
**Week 3**		**MS: Normal**	**MS (A9: B9)**		**Normal (C7: D5: E7)**	
	Anosim	$P = 0.002$(**)	$P = 0.961$		$P = 0.297$	
	Adonis	$P = 0.003$(**)	$P = 0.905$		$P = 0.293$	
**Week 8**		**D: M: F: N**	**D (A3: A5)**	**M (A4: B4)**	**F (C1: D1: E4)**	**N (C3: D2: E4)**
	**Anosim**	$P = 0.001$(***)	$P = 0.655$	$P = 0.336$	$P = 0.239$	$P = 0.577$
	**Adonis**	$P = 0.001$(***)	$P = 0.626$	$P = 0.402$	$P = 0.214$	$P = 0.825$
**Week 12**		**D: M: F: N**	**D (A3: A5)**	**M (A4: B4)**	**F (C1: D1: E3)**	**N (C3: D2: E4)**
	**Anosim**	$P = 0.001$(***)	P=0.221	$P = 0.116$	$P = 0.498$	$P = 0.075$
	**Adonis**	$P = 0.001$(***)	P=0.27	$P = 0.136$	$P = 0.69$	$P = 0.138$

** $P < 0.01$, *** $P < 0.001$. MS: maternal separation; Normal: normal-breeding; D: maternal separation with *Fusobacterium nucleatum* gavage; M: maternal separation with normal-saline gavage; F: normal breeding during lactation with *Fusobacterium nucleatum* gavage; N: normal control group with normal-saline gavage.

Supplementary Table 3. Patient characteristics for detection of *F. nucleatum* specific IgA

	**IBS-D patients (n = 7)**	**Healthy controls (n = 5)**
Age [mean (SD)]	41.43 (2.48)	36.20 (6.06)
BMI kg/m^2 [mean (SD)]	22.66 (1.77)	21.55 (1.18)
SAS [mean (SD)] **	51.86 (5.05)	36.00 (7.52)
SDS [mean (SD)] **	50.00 (10.13)	31.20 (3.90)

IBS-D, diarrhea predominant-irritable bowel syndrome; SD, standard deviation; BMI, Body Mass Index; SAS, Self-rating Anxiety Scale; SDS, Self-rating Depression Scale; **: $P < 0.01$.
