# Supplementary material for: Fusobacterium nucleatum Causes Microbial Dysbiosis and Exacerbates Visceral Hypersensitivity in a Colonization-Independent Manner
Source: Front Microbiol. 2020 Jun 24;11:1281. doi: 10.3389/fmicb.2020.01281 (PMC7358639; doi:10.3389/fmicb.2020.01281)
Supplement: Supplementary file 2 [file Data_Sheet_2.PDF]

Supplementary Table 4. Mass spectrometry

F. nucleatum 40 kDa	Coverage (Percent)	Probability (Percent)	Sequences and Coverage
1	71.20%	99.0%	>tr|Q47903|Q47903_FUSNC Porin OS=Fusobacterium nucleatum subsp. nucleatum OX=76856 GN=fomA PE=4 SV=1
			M K K L A L V L G S L L V V G S V A S A K E V M P A P T P A
			P K K V V E Y V E K P V I V Y R D R E V A P A W R P N G S V
			D V Q Y R W Y G N V E N R T P K K E D P A S P W L G D N V N
			A G R L Q T L T K V N F T E K Q T L E I R T R N Y H T L M N
			P K D S Q A A D D Q V R V R H F Y K F G K L G S S K I D V T
			S R L E Y K K N N G D A G R K Q A E A S V L F D F A D Y I Y
			S N N F F K A D K F G F R L G Y Q H K W A G H N S G V V G Q
			P F N K G T Q D N Y F I N F E S E Y T L P W G F S A E L N A
			Y N Y Y N V H N K K F A T Y N K G N K K S Q F Y G E I E A Y
			L Y Q H T P L Y K T N N V E L S F D F E G G Y D P Y T W H Q
			Y K V V S A K D S N K Y E V Y M L P T L Q V S Y K P T D F V
			K L Y A A A G A E Y R N W A V T A E S K A K N W R W Q P T A
			W A G M K V T F
			1 5 10 15 20 25 30
2	59.84%	99.0%	>tr|D5RDU9|D5RDU9_FUSN2 Acyl-CoA dehydrogenase, C-terminal domain protein OS=Fusobacterium nucleatum subsp. nucleatum (strain ATCC 23726 / VPI 4351) OX=525283 GN=bcd2 PE=3 SV=1
			M E F N V P K T H E L F R Q M I R E F V E K E V K P I A A E
			V D E N E R F P V E T V E K M A K I G I M G I P I P K Q Y G
			G A G G D N L M Y A M A V E E L S K A C A T T G V I V S A H
			T S L G T W P I L K F G N E K Q K Q K Y L P K M A S G E W I
			G A F G L T E P N A G T D A A G Q Q T M A V Q D P E T G E W
			I L N G A K I F I T N A G Y A H V Y V V F A M T D K S K G L
			K G I S A F I V E A N T P G F S I G K K E M K L G I R G S A
			T C E L I F E N C R I P K E N L L G D K G K G F K I A M M T
			L D G G R I G I A S Q A L G I A A G A L D E A I N Y A K E R
			K Q F G R S L A Q F Q N T Q F Q I A N L D V K V E A A R L L
			V Y K A A W R E S N N L P Y S L D A A R A K L F A A E T A M
			E V T T K A V Q I F G G Y G Y T R E Y P V E R M M R D A K I
			T E I Y E G T S E V Q R M V I A A N I I K
			1 5 10 15 20 25 30
3	49.87%	99.0%	>tr|Q8RFC6|Q8RFC6_FUSNN Electron transfer flavoprotein alpha-subunit OS=Fusobacterium nucleatum subsp. nucleatum (strain ATCC 25586 / CIP 101130 / JCM 8532 / LMG 13131) OX=190304 GN=FN0785 PE=4 SV=1
			M N L N D Y K G I L V F A E Q R D G I L Q N V G L E L L G K
			A T E L A Y E I N K Q I A L K D A G D E L A D F A G K K E A
			A I K S V D V A A A T L E E D D Q E L K N K V A D I K K N N
			P D A A K V T A L L V G H N I K N L A Q D L I K A G A D K V
			L V V D K P E L E V Y D T E A Y A Q V L T A T I N D Q K P E
			I V L F G A T T L G R D L A P R V S S R I A T G L T A D C T
			K L E L L K D K E R Q L G M T R P A F G G N L M A T I V S P
			D H R P Q M A T V R P G V M K K L P K S D D R T G E V V E F
			P V T L D T S K M K V K L L K V V K E G G N K V D I S E A K
			I L V S G G R G V G S K Q N F E L L E D L A T E I G G I V S
			S S R A Q V D A G N M P H D R Q V G Q T G K T V R P E V Y F
			A C G I S G A I Q H V A G M E E S E F I I A I N K D R F A P
			I F S V A D L G I V G D L H K I L P I L T E E I K K Y K A T
			K
			1 5 10 15 20 25 30
4	70.05%	99.0%	>sp|Q8R603|EFTU_FUSNN Elongation factor Tu OS=Fusobacterium nucleatum subsp. nucleatum (strain ATCC 25586 / CIP 101130 / JCM 8532 / LMG 13131) OX=190304 GN=tuf PE=3 SV=1
			M A K E K Y E R S K P H V N I G T I G H V D H G K T T T T A
			A I S K V L S D K G W A S K V D F D Q I D A A P E E K E R G
			I T I N T A H I E Y E T E K R H Y A H V D C P G H A D Y V K
			N M I T G A A Q M D G A I L V V S A A D G P M P Q T R E H I
			L L S R Q V G V P Y I V V Y L N K S D M V E D E E L L E L V
			E M E V R E L L T E Y G F P G D D I P V I R G S S L G A L N
			G E E K W V E K I L E L M E A V D N Y I P T P E R A V D Q P
			F L M P I E D V F T I T G R G T V V T G R V E R G V I K V G
			E E I E I V G I K P T T K T T C T G V E M F R K L L D Q G Q
			A G D N I G V L L R G T K K E E V E R G Q V L A K P G S I H
			P H T N F K G E V Y V L T K D E G G R H T P F F T G Y R P Q
			F Y F R T T D I T G A V T L P D G V E M V M P G D N I T M T
			V E L I H P I A M E Q G L R F A I R E G G R T V A S G V V S
			E I T K
			1 5 10 15 20 25 30
5	76.37%	99.0%	>tr|D5RE94|D5RE94_FUSN2 Acetyl-CoA C-acetyltransferase OS=Fusobacterium nucleatum subsp. nucleatum (strain ATCC 23726 / VPI 4351) OX=525283 GN=atoB PE=3 SV=1
			M S K V Y V V A A K R S A I G S F L G T L S P L K P G D L G
			A Q I V K N I L E E T K V D P A N I D E V I V G N V L S A G
			Q A Q G V G R Q V A I R A G I P Y E V P A Y S V N I I C G S
			G M K S V I T A F S N I K A G E A D L V I A G G T E S M S G
			A G F I L P G A I R G G H K M A D L T M K D H M I L D A L T
			D A Y H N I H M G I T A E N I A E R Y G I T R E E Q D A F A
			L D S Q L K A I A A V D S G R F K D E I A P V V I P N K K G
			D I I F D T D E Y P N R K T D A E K L A K L K P A F K K D G
			S V T A G N A S G L N D G A S F L M L A S E E A V K K Y N L
			K P L V E I V A T G T G G V D P L V M G M G P V P A I R K A
			F N K T D L K L K D M E L I E L N E A F A A Q S L G V I K E
			L C K E H G V T P E W I K E R T N V N G G A I A L G H P V G
			A S G N R I T V T L I Y E M K K R G V E Y G L A S L C I G G
			G M G T A L I L K N V K
			1 5 10 15 20 25 30
6	48.15%	99.0%	>tr|Q8RIB8|Q8RIB8_FUSNN N-acetylneuraminate synthase OS=Fusobacterium nucleatum subsp. nucleatum (strain ATCC 25586 / CIP 101130 / JCM 8532 / LMG 13131) OX=190304 GN=FN1684 PE=4 SV=1
			M K I G N K E I L K K G K P F F I A D I A A N H D G D L E R
			A Y K L I E L A K E A G A D V A K F Q N F N A K T I V S K K
			G F E T L G K Q L S H Q A N W K K S V Y E V Y K D A S I N P
			D W T E L L K R K C D E V G I E Y M T S P Y D Y A S V D L V
			D P Y V N A Y K I G S G D I T W T E I L K Y I L S K N K P I
			L L A T G A S S M E D V D R A M D I L K E N Q N I V L M Q C
			N T N Y T A S T E N F K Y I N L N V L K K Y E E K Y P N A I
			L G L S D H T K G F A T V L G A I A L G A V V I E K H F T D
			D N D R E G P D H K F A M N P K T W R E M V D A S L E L Y Y
			S L G D G I K R I E K N E E D T A M V Q R R G I Y I T K S L
			K A G H I L S R E D L I A L R P I K I D G I Q P Y E I D L L
			L G K K L T K N I E S D S Y L T W K D V E
			1 5 10 15 20 25 30
7	73.37%	99.0%	>sp|Q8RFN7|PGK_FUSNN Phosphoglycerate kinase OS=Fusobacterium nucleatum subsp. nucleatum (strain ATCC 25586 / CIP 101130 / JCM 8532 / LMG 13131) OX=190304 GN=pgk PE=3 SV=1
			M K K I I T D L D L N N K K V L M R V D F N V P M K D G K I
			T D E N R I V Q A L P T I K Y V L E H N A K L I L F S H L G
			K V K I E E D K A T K S L K A V A E K L S E L L G K N V T F
			I P E T R G E K L E S A I N N L K S G E V L M F E N T R F E
			D L D G K K E S K N D S E L G K Y W A S L G D V F V N D A F
			G T A H R A H A S N V G I A E N I G N G N S A V G F L V E K
			E L K F I G E A V N N P K R P L I A I L G G A K V S D K I G
			V I E N L L T K A D K I L I G G A M M F T F L K A E G K N I
			G T S L V E D D K L D L A K D L L A K S N G K I V L P V D T
			V I A S E F K N D I E F S T V D V D N I P N N K M G L D I G
			E K T V T L F D S Y I K T A K T V V W N G P M G V F E M S N
			F A K G T I G V C E S I A N L T D A V T I I G G G D S A A A
			A I S L G Y A D K F T H I S T G G G A S L E F L E G K V L P
			G V E A I S N K
			1 5 10 15 20 25 30
8	58.35%	99.0%	>sp|Q8RFG1|HUTI_FUSNN Imidazolonepropionase OS=Fusobacterium nucleatum subsp. nucleatum (strain ATCC 25586 / CIP 101130 / JCM 8532 / LMG 13131) OX=190304 GN=hutI PE=3 SV=1
			M Q A D L V L Y N I G Q L V T S R E L D K T K K M D N I E V
			I E N N G Y I V I E K D K I V A V G S G E V L K E Y L T P A
			T E M V D L S G K L V T P G L I D S H T H L V H G G S R E N
			E F A M K I A G V P Y L E I L E K G G G I L S T L K S T R N
			A S E Q E L I E K T L K S L R H M L E L G V T T V E A K S G
			Y G L N L E D E L K Q L E V T K I L G Y L Q P V T L V S T F
			M A A H A T P P E Y K D N K E G Y V Q E V I K M L P I V K E
			R N L A E F C D I F C E D K V F S V D E S R R I L T V A K E
			L G Y K L K I H A D E I V S L G G V E L A A E L G A T S A E
			H L M K I T D S G I N A L A N S N V I A D L L P A T S F N L
			M E H Y A P A R K M I E A G I Q I A L S T D Y N P G S C P S
			E N L Q F V M Q I G A A H L K M T P K E V F K S V T I N A A
			K A I D K Q D T I G S I E V G K K A D I T V F D A P S M A Y
			F L Y H F G I N H T D S V Y K N G K L V F K K
			1 5 10 15 20 25 30
9	52.17%	99.0%	>sp|Q8RHX3|KDD_FUSNN L-erythro-3,5-diaminohexanoate dehydrogenase OS=Fusobacterium nucleatum subsp. nucleatum (strain ATCC 25586 / CIP 101130 / JCM 8532 / LMG 13131) OX=190304 GN=kdd PE=1 SV=1
			M K K G C K Y G T H R V I E P A G V L P Q P A K K I S N D M
			E I F S N E I L I D V I A L N I D S A S F T Q I E E E A G H
			D V E K V K A K I K E I V A E R G K M Q N P V T G S G G M L

			I G T V E K I G D D L V G K T D L K V G D K I A T L V S L S
			L T P L R I D E I I N I K P E I D R V E I K G K A I L F E S
			G I Y A V L P K D M P E N L A L A A L D V A G A P A Q V A K
			L V K P C Q S V A I L G S A G K S G M L C A Y E A V K R V G
			P T G K V I G V V R N D K E K A L L Q R V S D K V K I V I A
			D A T K P M D V L H A V L E A N D A K E V D V A I N C V N V
			P N T E M S T I L P V K E F G I A Y F F S M A T G F S K A A
			L G A E G V G K D I T M I V G N G Y T V D H A A I T L E E L
			R E S A V L R E I F N E I Y L
			1 5 10 15 20 25 30
10	44.24%	99.0%	>tr|U7TXB8|U7TXB8_FUSNU Glutamate formiminotransferase OS=Fusobacterium nucleatum CTI-6 OX=1316587 GN=HMPREF1767_00531 PE=4 SV=1
			M A K I V E C I P N Y S E G K D L A K I E R I V A P Y K N N
			P K V K L L S V E P D A N Y N R T V V T V L G D P E E V K K
			A V I E S I G I A T K E I D M N V H K G E H K R M G A T D V
			V P F L P I Q E M T T E E C N E I S R E V G K A V W E R F Q
			L P V F L Y E S T A T A P N R V S L P D I R K G E Y E G M A
			E K L K Q P E W A P D F G E R A P H P T A G V T A I G C R M
			P L I A F N I N L A T T D M D I P K E I A K A I R F S S G G
			F R F I Q A G P A E I L D K G F V Q V T M N I K D Y T K N P
			I Y R I M E T V K M E A K R W G V K V T G C E I I G A T P F
			A S L T D S L K Y Y L A C D G I K D D V D A M S M E K V V E
			L M V K Y L G L T D F D V K K V L E A N I
			1 5 10 15 20 25 30
11	54.55%	99.0%	>tr|Q8RGI5|Q8RGI5_FUSNN Iron(III)-binding protein OS=Fusobacterium nucleatum subsp. nucleatum (strain ATCC 25586 / CIP 101130 / JCM 8532 / LMG 13131) OX=190304 GN=FN0308 PE=4 SV=1
			M K K K F F W L V L S L M T L F L V A C G G G E K K E E T T
			D S A N A N T E I S G K I V I Y T S M Y E D I I D N V S E K
			L K K E F P N L E V E F F Q G G T G T L Q S K I I A E L Q A
			N K L G C D M L M V A E P S Y S L E L K E K G I L H A Y L S
			K N A E N L A L D Y D K E G Y W Y P V R L L N M V L A Y N P
			D K Y K K E D L A L T F E D F A K R E D L A G K I S I P D P
			L K S G T A L A A V S A L S D K Y G E E Y F K N L A N L K V
			V V E S G S V A V T K L E T G E A A E I M I L E E S I L K K
			R E E E N S T L E V I Y P E D G I I S I P S T I M T V K E D
			M S A N K N I K A A E A L T D W F L S P A G Q E A I V E G W
			M H S V L K N P E K A P Y D A K A T D E I L K A S M P I N W
			E K T Y K D R E E L R K M F E K F I T K A N
			1 5 10 15 20 25 30
12	49.25%	99.0%	>sp|Q8RED7|ACKA_FUSNN Acetate kinase OS=Fusobacterium nucleatum subsp. nucleatum (strain ATCC 25586 / CIP 101130 / JCM 8532 / LMG 13131) OX=190304 GN=ackA PE=3 SV=1
			M K I L V I N C G S S S L K Y Q L I N P E T E E V F A K G L
			C E R I G I D G S K L E Y E V V A K D F E K K L E T P M P S
			H K E A L E L V I S H L T D K E I G V I A S V D E V D A I G
			H R V V H G G E E F A Q S V L I N D A V L K A I E A N N D L
			A P L H N P A N L M G I R T C M E L M P G K K N V A V F D T
			A F H Q T M K P E A F M Y P L P Y E D Y K E L K V R K Y G F
			H G T S H L Y V S G I M R E I M G N P E H S K I I V C H L G
			N G A S I T A V K D G K S V D T S M G L T P L Q G L M M G T
			R C G D I D P A A V L F V K N K R G L T D A Q M D D R M N K
			K S G I L G L F G K S S D C R D L E N A V V E G D E R A I L
			A E S V S M H R L R S Y I G A Y A A I M G G V D A I C F T G
			G I G E N S S M T R E K A L E G L E F L G V E L D K E I N S
			V R K K G N V K L S K D S S K V L I Y K I P T N E E L V I A
			R D T F R L A K
			1 5 10 15 20 25 30
13	34.33%	99.0%	>tr|Q8RFN9|Q8RFN9_FUSNN Glyceraldehyde-3-phosphate dehydrogenase OS=Fusobacterium nucleatum subsp. nucleatum (strain ATCC 25586 / CIP 101130 / JCM 8532 / LMG 13131) OX=190304 GN=FN0652 PE=3 SV=1
			M A V K V A I N G F G R I G R L A L R V M S K N K D F D V V
			A I N D L T D A K T L A H L F K Y D S A Q G R F D G T I E V
			T D D G F V V N G D S I K V F A K A N P E E L P W K D L G V
			D V V L E C T G F F T S K E K A E A H I K A G A K K V V I S
			A P A T G D L K T V V Y N V N D N I L D G S E T V I S G A S
			C T T N C L A P M A K V L N D K F G I V E G L M T T I H A Y
			T N D Q N T L D A P H K K G D L R R A R A A A E N I V P N T
			T G A A K A I G L V I P E L K G K L D G A A Q R V P V I T G
			S I T E L V T V L G K E V T V E E I N A A M K A A S N E S F
			G Y T E E P L V S S D I I G I S F G S L F D A T Q T K V L T
			V D G K Q L V K T V A W Y D N E M S Y T S Q L I R T L K K F
			V E I S K
			1 5 10 15 20 25 30
14	32.28%	99.0%	>tr|D5REL3|D5REL3_FUSN2 Acyl-CoA dehydrogenase, C-terminal domain protein OS=Fusobacterium nucleatum subsp. nucleatum (strain ATCC 23726 / VPI 4351) OX=525283 GN=acdA PE=3 SV=1
			M A Y L I S E E A Q D L L K D V K K F C D N E V R E Q C K E
			Y D K S G E W P K E I Y D K A I E Q G Y Q A L E V P E K Y G
			G P G L K R V D I A A L I E E M A I A D A G F A T T I S A S
			G L A M K P V F I S G S E E Q K Q R M C D L V L D G G F G A
			F C L T E P G A G S D A S A G R T T A V K D G D E Y V L N G
			R K C F I T N G A V A S F Y C I T A I T D K E K G L K G I S
			M F F V E K G T K G L S T G N H E D K M G I R T S N T C D V
			V L E D C R I P A S A L V G K E G E G F A I A M K T L D Q A
			R S W I G C I A V G I A Q R G I Q E A I A Y G K E R I Q F G
			K P I I K N Q A L Q F K I A D M E I K T E T A R Q M V A H A
			L T K M D L G L P Y G K E S A I A K C Y A G D I A M E V S S
			E A I Q M F G G Y G Y S R E Y P V E K L I R D S K I F Q I F
			E G T N E I Q R I V I A N N V I G R
			1 5 10 15 20 25 30
15	48.82%	99.0%	>tr|D5RE86|D5RE86_FUSN2 4-phosphoerythronate dehydrogenase OS=Fusobacterium nucleatum subsp. nucleatum (strain ATCC 23726 / VPI 4351) OX=525283 GN=pdxB PE=3 SV=1
			M K V L F Y G V R E V E I P L F H E L N K K E G F G Y E L E
			L I P D Y L N S K E T A E K A K G F E C V V L R G N C F A T
			K E V L D M Y K E Y G V K Y L L T R T V G T N H I D V K Y A
			K E L G F K L A Y V P F Y S P N A I A E L A V S L A M S L L
			R H L P Y T A E K F K N R N F T V D A Q M F S K E V R N C T
			V G V I G L G R I G F T A A K L F K G L G A N V I G Y D M F
			P K T G V E D I V T Q V P M D E L I K K S D I I T L H A P F
			I K E N G K I V T K E F L N N M K E N S I L I N T A R G E L
			M D L E A V V E A L E S G H L A A A G I D T I E G E V N Y F
			F K N F S D K Q A E F R A D Y P L Y N R L L D L Y P R V L V
			T P H V G S Y T D E A A S N M I E T S F E N L K E Y L D T G
			A C K N D I K A
			1 5 10 15 20 25 30
16	50.72%	99.0%	>tr|A0A0X3Y1L1|A0A0X3Y1L1_FUSNC Uncharacterized protein OS=Fusobacterium nucleatum subsp. nucleatum OX=76856 GN=RO03_04420 PE=4 SV=1
			M K K L L L L T A I L S S G L Y A A S I D H I Q T Y S P D Y
			L A N Q A Q T G M I N G V S P Y Y N P A A L G R L E K G K Y
			L H A G F Q F A H G H E K M S Y K G K E H K A N L N Q A I P
			N I A L T F V D D K G A T F F N F G G L A G G G K L R Y D G
			V S G V D V L T D L A Q F K P L G I Y D K G S S L T G S N K
			Y E Q I T L G R A F N I D D K L S F S V A A R V V H G T R R
			L N G N L N I G A N P T A Q Y K Q E K A Q Q V A Q E V S K A
			V D A A T Q G K G L S A A Q I A A I K T K K T N E A L A K L
			Q Q R V N D L S Q N G L S G D I D S K R E A W G Y G F Q L G
			V N Y K V N D K L N L A A R Y D S R I K M N F K A K G H E H
			Q L E T T D I L K Q T I G L S T F Y P Q Y T I N S K I R R D
			L P A I L S V G A S Y K V A D N Y L V S T T A N Y Y F N H Q
			A K M D R V T T F G E H E H G R D Y K N G W E I A V G N E Y
			K L N D K F T L I G S L N Y A N T G A K T A S Y N D T E Y A
			L N S F T L G G G I R Y Q Y D E S L A I T A S V A H F I Y Q
			G A E G N F K E K Y G V T E N Q K Y K K E I T A V G L S V T
			K K F
			1 5 10 15 20 25 30
17	28.47%	99.0%	>tr|D5RE87|D5RE87_FUSN2 Glutamate dehydrogenase OS=Fusobacterium nucleatum subsp. nucleatum (strain ATCC 23726 / VPI 4351) OX=525283 GN=gluD PE=3 SV=1
			M S K E T L N P L E S G Q K Q V K K A C D A L G L D P A V Y
			E L L K E P Q R I I E I T I P V K M D D G S I K T F K G Y R
			A A H N D A V G P F K G G I R F H Q N V N S D E V K A L S L
			W M S I K C Q V T G I P Y G G G K G G I T V D P S E L S Q R
			E L E Q L S R G W V R G M W K Y L G E K V D V P A P D V N T
			N G Q I M A W M Q D E Y N K L S G E Q T I G V F T G K P L S
			Y G G S Q G R N E A T G F G V A V T M R E A F K A L G K N L
			K G A T V A V Q G F G N V G K F T V K N I M K L G G K V V A
			V A E F E K G K G A Y A I Y K D S G F T F E E L E A A K A A
			G S L T K V A G A K E L S M D E F W A L N V E A I A P C A L
			E N A I T N H E A E L I K A G I V C E G A N G P I T P E A D
			E V L Y K K G I V V T P D V L T N A G G V T V S Y F E W V Q
			N I Y G Y Y W T E K E V E E K E E R A M V D A F T P I W A L
			K K E F D G K G Q P I S F R Q A T Y M K S I K R I A E A M K
			I R G W Y
			1 5 10 15 20 25 30

18	36.72%	99.0%	>tr|D5RDX3|D5RDX3_FUSN2 Cell shape determining protein, MreB/Mrl family OS=Fusobacterium nucleatum subsp. nucleatum (strain ATCC 23726 / VPI 4351) OX=525283 GN=ppx PE=4 SV=1																														
				M	M	R	C	K	V	M	K	K	F	M	G	K	L	L	G	I	F	S	D	D	L	G	I	D	L	G	T	S	N
				T	L	I	C	M	K	N	K	G	I	I	L	R	E	P	S	V	V	A	I	S	T	K	T	K	E	I	F	E	V
				G	E	K	A	K	H	M	I	G	R	T	P	S	T	Y	E	T	I	R	P	L	R	N	G	V	I	A	D	Y	E
				V	T	E	K	M	L	R	S	F	Y	K	R	I	K	S	G	T	L	L	N	K	P	R	V	I	I	C	V	P	A
				G	I	T	Q	V	E	K	R	A	V	M	E	V	T	R	E	A	G	A	R	E	A	Y	L	I	E	E	P	M	A
				A	A	I	G	V	G	I	N	I	F	E	P	E	G	S	M	V	V	D	I	G	G	G	T	S	E	L	A	V	V
				S	L	G	G	V	V	K	K	S	S	F	R	V	A	G	D	R	F	D	T	A	I	V	D	Y	V	R	Q	K	H
				N	L	L	I	G	E	K	S	A	E	D	I	K	I	K	I	G	T	V	S	P	E	E	E	D	M	E	I	E	V
				S	G	K	Y	V	L	N	G	L	P	K	D	I	T	L	T	S	S	E	L	I	D	T	L	S	T	L	V	Q	E
				I	I	E	E	I	R	V	V	F	E	K	T	P	P	E	L	A	A	D	I	K	K	R	G	I	Y	I	S	G	G
				G	A	L	L	R	G	I	D	K	K	I	A	A	G	L	N	L	K	V	T	I	S	E	D	P	L	N	A	V	I
				N	G	I	G	V	L	L	N	N	F	S	L	Y	S	K	V	L	V	S	T	E	T	E	Y						
				1				5					10					15					20					25					30
19	35.81%	99.0%	>sp|Q8R5X7|CH60_FUSNN 60 kDa chaperonin OS=Fusobacterium nucleatum subsp. nucleatum (strain ATCC 25586 / CIP 101130 / JCM 8532 / LMG 13131) OX=190304 GN=groL PE=3 SV=1																														
				M	A	K	I	I	N	F	N	D	E	A	R	K	K	L	E	I	G	V	N	T	L	A	D	A	V	K	V	T	L
				G	P	R	G	R	N	V	V	L	E	K	S	Y	G	A	P	L	I	T	N	D	G	V	T	I	A	K	E	I	E
				L	E	D	P	F	E	N	M	G	A	A	L	V	K	E	V	A	I	K	S	N	D	V	A	G	D	G	T	T	T
				A	T	I	L	A	Q	A	I	V	K	E	G	L	K	M	L	S	A	G	A	N	P	I	F	L	K	K	G	I	E
				L	A	A	K	E	A	V	E	V	L	K	D	K	A	K	K	I	E	S	N	E	E	I	S	Q	V	A	S	I	S
				A	G	D	E	E	I	G	K	L	I	A	Q	A	M	A	K	V	G	E	T	G	V	I	T	V	E	E	A	K	S
				L	E	T	T	L	E	I	V	E	G	M	Q	F	D	K	G	Y	V	S	P	Y	M	V	T	D	S	E	R	M	T
				A	E	L	D	N	P	L	I	L	L	T	D	K	K	I	S	S	M	K	E	L	L	P	L	L	E	Q	T	V	Q
				M	S	K	P	V	L	I	V	A	D	D	I	E	G	E	A	L	T	T	L	V	I	N	K	L	R	G	T	L	N
				V	V	A	V	K	A	P	A	F	G	D	R	R	K	A	I	L	E	D	I	A	I	L	T	G	G	E	V	I	S
				E	E	K	G	M	K	L	E	E	A	T	I	E	Q	L	G	K	A	K	T	V	K	V	T	K	D	L	T	V	I
				V	D	G	G	G	Q	Q	K	D	I	S	A	R	V	N	S	I	K	A	Q	I	A	E	T	T	S	D	Y	D	K
				E	K	L	Q	E	R	L	A	K	L	S	G	G	V	A	V	I	K	V	G	A	A	T	E	V	E	M	K	D	K
				K	L	R	I	E	D	A	L	N	A	T	R	A	A	V	E	E	G	I	V	A	G	G	G	T	I	L	L	D	I
				I	E	S	M	K	D	F	N	E	T	D	E	I	A	M	G	I	E	I	V	K	R	A	L	E	A	P	I	K	Q
				I	A	E	N	C	G	L	N	G	G	V	V	L	E	K	V	R	M	S	P	K	G	F	G	F	D	A	K	N	E
				K	Y	V	N	M	I	E	S	G	I	I	D	P	A	K	V	T	R	A	A	I	Q	N	S	T	S	V	A	S	L
				L	L	T	T	E	V	V	I	A	N	K	K	E	E	E	K	A	P	M	G	A	G	G	M	M	P	G	M	M	
				1				5					10					15					20					25					30
20	44.11%	99.0%	>sp|Q8R600|EFTS_FUSNN Elongation factor Ts OS=Fusobacterium nucleatum subsp. nucleatum (strain ATCC 25586 / CIP 101130 / JCM 8532 / LMG 13131) OX=190304 GN=tsf PE=3 SV=2																														
				M	A	T	V	T	A	A	L	V	K	E	L	R	E	R	T	G	A	G	M	L	D	C	K	K	A	L	E	S	H
				D	G	D	I	E	K	S	I	D	Y	L	R	E	K	G	I	A	K	A	V	K	K	A	G	R	I	A	A	E	G
				L	I	F	D	E	A	T	P	D	H	K	K	A	V	I	L	E	F	N	S	E	T	D	F	V	A	K	N	E	E
				F	K	E	F	G	R	K	L	V	K	I	A	L	E	R	N	V	H	Q	L	E	E	L	N	E	A	Q	V	E	G
				D	K	K	V	S	E	A	L	T	D	L	I	A	K	I	G	E	N	M	S	L	R	R	L	A	V	V	V	A	K
				D	G	F	V	Q	T	Y	S	H	L	G	G	K	L	G	V	I	V	E	M	S	G	E	P	T	E	A	N	L	E
				K	A	K	N	I	A	M	H	V	A	A	M	D	P	K	Y	L	S	E	E	E	V	T	A	S	D	L	E	H	E
				K	E	I	A	R	K	Q	L	E	E	E	G	K	P	A	N	I	I	E	K	I	L	T	G	K	M	H	K	F	Y
				E	E	N	C	L	V	D	Q	I	Y	V	R	A	E	N	K	E	T	V	K	Q	Y	A	G	D	I	K	V	L	S
				F	E	R	F	K	V	G	D	G	I	E	K	R	E	E	D	F	A	A	E	V	A	A	Q	I	N	G			
				1				5					10					15					20					25					30
21	33.25%	99.0%	>tr|D5RC19|D5RC19_FUSN2 2-hydroxyglutaryl-CoA dehydratase D-component OS=Fusobacterium nucleatum subsp. nucleatum (strain ATCC 23726 / VPI 4351) OX=525283 GN=hgdB PE=4 SV=1																														
				M	A	E	I	K	E	L	L	E	Q	F	K	Y	Y	A	E	N	P	R	K	Q	L	D	K	Y	L	A	E	G	K
				K	A	V	G	I	F	P	Y	Y	A	P	E	E	I	V	Y	A	G	G	M	V	P	F	G	V	W	G	G	Q	G
				P	I	E	K	A	K	D	Y	F	P	T	F	Y	Y	S	L	A	L	R	C	L	E	M	A	L	D	G	T	L	D
				G	L	S	A	S	M	V	T	T	L	D	D	T	L	R	P	F	S	Q	N	Y	K	V	S	A	G	R	K	I	P
				M	V	F	L	N	H	G	Q	H	R	K	E	E	F	G	K	K	Y	N	A	K	I	F	N	N	A	K	E	E	L
				E	K	I	C	D	V	K	I	T	D	E	N	L	K	K	A	F	K	V	Y	N	E	N	R	E	E	K	R	K	F
				I	K	L	A	A	K	H	P	Q	S	I	K	A	S	D	R	S	N	V	L	K	S	S	Y	F	M	L	K	D	E
				H	T	D	L	L	R	Q	L	N	Q	K	L	E	A	L	P	E	E	Q	W	D	G	V	R	V	V	T	S	G	I
				I	T	D	N	P	G	L	L	E	V	F	D	N	Y	K	V	C	V	V	A	D	D	V	A	H	E	S	R	A	L
				K	V	D	I	D	L	S	I	E	D	P	M	L	A	L	A	D	Q	F	A	R	M	D	E	D	P	L	L	Y	D
				P	D	I	I	K	R	P	K	Y	V	L	D	L	V	K	E	N	N	A	D	G	C	L	L	F	M	M	N	F	N
				D	T	E	E	M	E	Y	P	S	L	K	Q	A	F	D	E	A	K	V	P	L	I	K	M	G	Y	D	Q	Q	M
				V	D	F	G	Q	V	K	T	Q	L	E	T	F	N	E	L	V	Q	L	S	R	F								
				1				5					10					15					20					25					30
22	37.70%	99.0%	>tr|D5RAS2|D5RAS2_FUSN2 Oxidoreductase, 2-nitropropane dioxygenase family protein OS=Fusobacterium nucleatum subsp. nucleatum (strain ATCC 23726 / VPI 4351) OX=525283 GN=HMPREF0397_0307 PE=4 SV=1																														
				M	K	E	L	K	G	I	K	I	G	K	Y	Y	I	E	K	P	I	V	Q	G	G	M	G	V	G	V	S	W	D
				Q	L	A	G	T	V	S	K	N	G	G	L	G	T	I	S	G	I	C	T	G	Y	Y	D	N	L	K	Y	C	K
				K	V	V	N	G	R	P	I	G	A	D	A	L	N	S	R	E	A	M	I	E	L	F	K	N	A	R	K	I	C
				G	D	K	P	L	A	C	N	I	L	H	A	L	N	D	Y	S	K	I	V	E	Y	A	L	E	A	G	A	N	I
				I	V	T	G	A	G	L	P	L	E	L	P	K	L	V	E	S	Y	P	D	V	A	I	V	P	I	V	S	S	G
				R	A	L	K	I	I	C	K	K	W	Q	A	A	G	R	L	P	D	A	V	I	V	E	G	P	K	S	G	G	H
				Q	G	V	K	A	D	D	L	F	I	P	E	H	Q	L	E	S	I	V	P	E	V	K	E	E	R	D	K	W	G
				D	F	P	I	I	A	A	G	G	I	W	D	N	D	D	I	Q	K	I	M	A	L	G	A	D	A	V	Q	L	G
				T	R	F	I	G	T	Y	E	C	D	A	S	E	E	F	K	N	I	L	I	N	A	K	K	E	D	I	V	I	V
				K	S	P	V	G	Y	P	G	R	A	V	K	T	N	L	I	K	N	L	T	H	D	T	P	T	I	K	C	Y	S
				N	C	V	T	P	C	N	L	G	E	E	A	R	K	V	G	F	C	I	A	N	C	L	S	D	S	Y	N	G	K
				V	D	T	G	L	F	F	S	G	E	N	G	Y	R	I	E	K	L	V	S	V	E	D	L	I	N	E	L	M	T
				S	T	K	N	T	I	L	A	K	V	N	S	E	N	I	I	E	N	T	V	N	F								
				1				5					10					15					20					25					30
23	38.46%	99.0%	>tr|A0A0M3UX89|A0A0M3UX89_FUSNC Uncharacterized protein OS=Fusobacterium nucleatum subsp. nucleatum OX=76856 GN=RO03_10820 PE=4 SV=1																														
				M	K	K	F	F	L	T	I	L	F	C	I	L	S	I	F	T	F	A	N	D	W	E	F	G	S	E	G	E	H
				I	I	P	L	K	G	S	N	M	S	I	K	K	E	K	I	T	L	K	L	T	P	D	G	M	L	V	N	V	K
				F	T	F	D	N	P	T	A	E	N	K	I	I	G	F	V	T	P	E	S	G	S	G	G	E	G	E	G	K	G
				N	R	K	P	E	P	L	N	I	K	S	F	K	T	T	V	N	G	K	E	V	K	S	N	V	E	L	L	S	K
				L	L	S	K	G	V	L	D	N	N	V	I	K	E	Y	T	E	K	E	K	N	F	Y	N	Y	V	Y	Y	F	N
				A	D	F	K	Q	G	E	N	V	V	E	H	N	Y	F	Y	T	G	S	Y	G	V	Y	E	R	D	F	S	Y	V
				V	T	T	I	S	K	W	K	N	K	T	V	E	D	F	E	I	E	V	Q	P	G	N	Y	F	V	K	L	P	Y
				S	F	W	K	N	N	N	K	I	N	W	E	I	V	G	K	G	K	M	V	T	I	A	P	T	K	K	P	N	D
				E	D	A	N	G	I	E	K	F	G	V	I	Y	L	K	L	D	N	G	F	V	R	Y	K	A	K	N	F	S	P
				T	D	D	F	Y	M	V	R	M	D	N	I	L	G	F	G	Y	E	F	P	E	G	K	V	Q	G	Y	K	F	K
				D	K	Y	F	D	I	A	A	R	G	N	S	Y	G	D	T	G	F	I	K	E	Y	Q	Q	D	L	N	D	K	D
				L	D	I	I	R	N	Y	P	Y	A	L	A	G	Y	D	F	D	K	K	D	L	K	D	Y	F	S	Q	F	I	W
				Y	S	P	V	S	K	N	V	K	I	D	S	D	S	N	N	I	I	K	A	V	D	E	I	N	T	K	R	K	K
				1				5					10					15					20					25					30
24	40.80%	99.0%	>sp|Q8RE44|RPOA_FUSNN DNA-directed RNA polymerase subunit alpha OS=Fusobacterium nucleatum subsp. nucleatum (strain ATCC 25586 / CIP 101130 / JCM 8532 / LMG 13131) OX=190304 GN=rpoA PE=3 SV=2																														
				M	L	K	I	E	K	Q	A	K	A	I	K	I	T	E	V	K	E	S	N	Y	K	G	Q	F	I	V	E	P	L
				Y	R	G	Y	G	N	T	L	G	N	A	L	R	R	V	L	L	S	S	I	P	G	A	A	I	K	G	M	R	I
				E	G	V	L	S	E	F	T	V	M	D	G	V	K	E	A	V	T	E	I	I	L	N	V	K	E	I	V	V	K
				A	E	S	S	G	E	R	R	M	S	L	S	I	K	G	P	K	V	V	K	A	A	D	I	V	A	D	I	G	L
				E	I	V	N	P	E	Q	V	I	C	T	V	T	T	D	R	T	L	D	I	E	F	I	V	D	T	G	E	G	F
				V	V	S	E	E	I	D	K	K	D	W	P	V	D	Y	I	A	V	D	A	I	Y	T	P	I	R	K	V	S	Y
				E	I	Q	D	T	M	F	G	R	M	T	D	F	D	K	L	T	L	N	V	E	T	D	G	S	I	E	I	R	D
				A	L	S	Y	A	V	E	L	L	K	L	H	L	D	P	F	L	E	I	G	N	K	M	E	N	L	R	D	D	I
				E	E	M	I	E	E	P	M	D	I	Q	V	I	D	D	K	S	H	D	M	K	I	E	E	L	D	L	T	V	R
				S	F	N	C	L	K	K	A	G	I	E	E	V	S	Q	L	A	S	L	S	L	N	E	L	L	K	I	K	N	L
				G	K	K	S	L	D	E	I	L	E	K	M	K	D	L	G	Y	D	L	E	K	N	G	S	P	E				
				1				5					10					15					20					25					30
25	42.31%	99.0%	>tr|Q8RFS0|Q8RFS0_FUSNN DNA polymerase III, beta chain OS=Fusobacterium nucleatum subsp. nucleatum (strain ATCC 25586 / CIP 101130 / JCM 8532 / LMG 13131) OX=190304 GN=FN0617 PE=3 SV=1																														
				M	K	F	S	I	N	R	Q	K	T	I	E	I	I	G	E	Y	S	N	I	L	K	D	N	P	V	K	P	S	L
				A	G	L	F	I	Q	A	K	N	N	Q	V	V	F	K	G	A	N	T	E	I	E	L	I	R	Y	A	N	C	E
				I	E	S	E	G	Q	V	L	I	K	P	A	L	L	L	E	Y	I	K	L	L	E	G	E	N	I	N	F	E	K
				K	D	G	Y	L	I	V	N	N	A	E	F	S	I	L	D	D	N	T	Y	P	E	L	T	E	I	I	P	I	V
				I	A	S	E	N	T	V	K	F	T	M	S	L	E	K	V	K	F	L	T	N	S	S	A	S	T	D	T	L	F
				N	S	I	K	M	I	F	K	D	N	V	L	E	L	V	S	T	D	S	F	R	L	I	Y	M	K	K	E	L	N
				N	M	I	N	K	D	V	L	V	P	G	D	S	I	A	V	L	Y	K	I	F	K	D	L	D	E	E	F	S	L
				A	A	S	D	D	K	L	I	V	T	W	K	D	A	Y	F	T	C	K	L	L	S	L	S	F	P	D	F	R	P
				L	I	N	N	T	N	H	D	K	R	F	E	F	N	R	D	E	L	N	L	A	L	K	K	V	I	S	V	T	K
				N	S	S	D	S	K	N	V	A	T	F	N	F	K	G	N	Q	L	L	I	S	G	V	S	A	N	A	K	I	N
				Q	K	V	N	M	I	K	T	G	E	D	L	K	L	G	M	N	C	K	Y	I	K	D	F	I	D	N	V	D	K
				N	I	I	I	D	A	T	N	S	S	S	M	L	R	F	M	E	E	G	N	E	N	Y	I	Y	L	I	M	P	V
				N	I	R	V																										
				1				5					10					15					20					25					30
26	43.79%	99.0%	>tr|A0A117MVW7|A0A117MVW7_FUSNC Phenylalanine--tRNA ligase alpha subunit OS=Fusobacterium nucleatum subsp. nucleatum OX=76856 GN=pheS PE=3 SV=1																														
				M	K	E	E	I	L	K	V	K	E	E	I	Q	N	Y	I	K	E	S	K	T	L	Q	R	L	E	E	I	R	V
				N	Y	M	G	K	K	G	I	F	T	E	L	S	K	K	M	K	D	L	S	V	E	E	R	P	K	I	G	Q	I
				I	N	E	V	K	E	K	I	N	S	L	L	D	E	R	N	K	A	L	K	E	K	E	L	N	E	R	L	E	S
				E	I	I	D	I	S	L	P	G	T	K	Y	N	Y	G	T	I	H	P	I	N	E	T	M	E	L	M	K	N	I
				F	S	K	M	G	F	D	I	V	D	G	P	E	I	E	T	V	E	Y	N	F	D	A	L	N	I	P	K	T	H
				P	S	R	D	L	T	D	T	F	Y	L	N	D	S	I	V	L	R	T	Q	T	S	P	V	Q	I	R	Y	M	L
				E	H	G	T	P	F	R	M	I	C	P	G	K	V	Y	R	P	D	Y	D	I	S	H	T	P	M	F	H	Q	M
				E	G	L	V	V	G	K	D	I	S	F	A	D	L	K	G	I	L	T	H	F	V	K	E	V	F	G	D	R	K
				V	R	F	R	P	H	F	F	P	F	T	E	P	S	A	E	M	D	V	E	C	M	I	C	H	G	D	G	C	R
				L	C	K	E	S	G	W	I	E	I	M	G	C	G	M	V	D	P	E	V	L	K	Y	V	G	L	N	P	D	E
				V	N	G	F	A	F	G	V	G	I	E	R	V	T	M	L	R	H	G	I	G	D	L	R	A	F	F	E	N	D
				M	R	F	L	K	Q	F	K																						

				1				5					10					15					20					25					30
27	39.01%	99.0%	>tr|D5RDA1|D5RDA1_FUSN2 Metallo-beta-lactamase domain protein OS=Fusobacterium nucleatum subsp. nucleatum (strain ATCC 23726 / VPI 4351) OX=525283 GN=HMPREF0397_1186 PE=3 SV=1																														
				M	H	N	V	R	K	I	T	E	D	L	Y	W	I	G	A	N	D	R	R	L	A	L	F	E	N	I	H	P	I
				P	E	G	V	S	Y	N	S	Y	M	L	L	D	K	E	T	V	V	F	D	T	V	D	W	S	V	T	R	Q	Y
				I	E	N	I	E	Y	L	L	N	G	R	E	L	D	Y	L	V	V	H	H	M	E	P	D	H	C	G	S	I	E
				E	L	A	L	R	Y	P	K	M	K	I	I	S	S	E	K	G	F	M	F	M	R	Q	F	G	Y	K	S	I	N
				G	H	Q	L	I	E	A	K	E	G	D	K	F	K	F	G	K	H	E	I	V	F	L	E	A	P	M	V	H	W
				P	E	V	L	V	S	F	D	T	T	N	G	A	L	F	S	A	D	A	F	G	S	F	K	S	L	D	G	R	L
				F	N	D	E	V	N	W	D	R	D	W	L	D	E	G	R	R	Y	L	T	N	I	V	G	K	Y	G	P	H	I
				Q	H	L	L	K	K	A	G	P	I	V	D	K	I	K	F	I	C	P	L	H	G	V	V	W	R	N	D	F	G
				Y	L	I	D	K	Y	D	K	W	S	R	Y	E	P	E	E	K	G	I	L	I	A	Y	A	S	M	Y	G	N	T
				E	N	A	V	E	I	L	A	A	K	L	A	E	K	G	I	T	N	I	K	M	F	D	V	S	N	T	H	V	S
				Y	L	I	S	N	V	F	K	Y	S	H	I	V	I	A	S	P	T	Y	N	L	G	I	Y	P	V	I	H	N	F
				V	M	D	M	K	A	L	N	L	Q	N	R	T	V	A	I	V	E	N	G	S	W	A	R	K	S	G	D	L	L
				Q	E	F	F	E	T	Q	I	K	D	I	T	V	L	N	E	R	V	G	L	T	S	S	A	N	N	V	N	L	D
				E	M	D	T	L	V	D	A	L	V	E	S	L	N	K															
				1				5					10					15					20					25					30
28	15.24%	99.0%	>tr|D5RD18|D5RD18_FUSN2 Pyruvate synthase OS=Fusobacterium nucleatum subsp. nucleatum (strain ATCC 23726 / VPI 4351) OX=525283 GN=nifJ PE=3 SV=1																														
				M	A	K	K	M	Q	T	M	D	G	N	Q	A	A	A	Y	A	S	Y	A	F	T	E	V	A	G	I	Y	P	I
				T	P	S	S	P	M	A	E	Y	T	D	E	W	A	A	K	G	M	K	N	I	F	G	V	P	V	K	L	V	E
				M	Q	S	E	G	G	A	A	G	T	V	H	G	S	L	Q	A	G	A	L	T	T	T	Y	T	A	S	Q	G	L
				L	L	K	I	P	N	M	Y	K	I	A	G	E	L	L	P	G	V	I	H	V	S	A	R	S	L	S	A	Q	A
				L	S	I	F	G	D	H	Q	D	I	Y	A	A	R	Q	T	G	F	A	M	L	A	T	N	S	V	Q	E	V	M
				D	L	A	G	V	A	H	L	S	A	L	K	S	R	V	P	F	L	H	F	F	D	G	F	R	T	S	H	E	I
				Q	K	V	E	V	M	D	Y	E	D	L	K	K	L	V	D	W	K	A	L	E	G	F	R	K	R	A	L	N	P
				E	H	P	V	T	R	G	T	A	Q	N	D	D	I	Y	F	Q	A	R	E	V	Q	N	K	F	Y	D	A	V	P
				D	I	V	A	D	Y	M	K	E	I	S	K	I	T	G	R	E	Y	K	P	F	N	Y	Y	G	A	P	D	A	E
				R	V	I	I	A	M	G	S	V	C	E	A	A	Q	E	V	I	D	Y	L	V	E	K	G	E	K	V	G	L	I
				S	V	H	L	F	R	P	F	S	A	K	Y	F	F	D	V	L	P	K	T	A	K	R	I	S	V	L	D	R	T
				K	E	P	G	S	L	G	E	P	L	L	L	D	I	K	A	L	F	Y	N	K	E	N	A	P	L	I	V	G	G
				R	Y	G	L	S	S	K	D	T	T	P	A	Q	I	K	A	V	F	D	N	L	A	K	D	T	P	K	D	A	F
				T	V	G	I	V	D	D	V	T	H	T	S	L	E	V	G	P	A	M	A	L	A	D	P	T	T	K	A	C	L
				F	Y	G	L	G	A	D	G	T	V	G	A	N	K	N	S	I	K	I	I	G	D	K	T	D	L	Y	A	Q	G
				Y	F	A	Y	D	S	K	K	S	G	G	V	T	R	S	H	L	R	F	G	K	K	P	I	R	S	T	Y	L	V
				S	R	P	T	F	V	A	C	S	V	P	A	Y	L	N	Q	Y	D	M	T	S	G	L	K	E	G	G	K	F	L
				L	N	C	V	W	T	K	E	E	A	L	E	N	I	P	N	N	V	K	R	D	L	A	K	N	K	A	R	L	F
				I	I	N	A	T	A	L	A	Q	E	I	G	L	G	Q	R	T	N	T	I	M	Q	A	A	F	F	K	L	A	E
				I	I	P	F	E	E	A	Q	Q	Y	M	K	D	Y	A	K	K	S	Y	A	K	K	G	D	E	I	V	Q	L	N
				Y	N	A	I	D	R	G	A	N	D	I	I	E	I	E	V	D	P	A	W	A	N	L	E	V	T	P	L	N	E
				P	K	E	T	T	G	C	A	Y	C	Q	P	D	T	E	F	V	K	K	I	V	R	P	V	N	A	I	K	G	Y
				D	L	P	V	S	A	F	L	G	Y	E	D	G	T	F	E	N	G	T	S	A	F	E	K	R	G	V	A	V	D
				V	P	I	W	N	V	D	K	C	I	Q	C	N	Q	C	S	Y	V	C	P	H	A	A	I	R	P	F	L	I	N
				E	D	E	L	K	A	A	P	N	S	F	A	T	K	K	A	T	G	K	G	L	D	E	L	V	Y	R	I	Q	V
				S	A	L	D	C	V	G	C	G	S	C	A	N	V	C	P	A	N	A	L	D	M	R	P	I	A	E	S	L	E
				T	H	E	D	I	N	T	N	Y	L	Y	N	N	V	E	Y	R	S	D	L	M	P	L	D	T	V	K	G	S	Q
				F	S	Q	P	L	F	E	F	H	G	A	C	P	G	C	G	E	T	P	Y	I	K	L	I	T	Q	L	Y	G	D
				R	M	M	V	A	N	A	T	G	C	S	S	I	Y	S	G	S	A	P	A	T	P	Y	T	T	N	K	N	G	E
				G	P	S	W	G	S	S	L	F	E	D	N	A	E	Y	G	F	G	M	H	V	G	V	E	A	L	R	F	R	I
				Q	H	T	M	E	E	N	M	D	K	V	D	E	D	I	A	T	L	F	K	D	W	I	A	N	R	Q	Y	S	V
				R	T	R	E	V	R	D	I	L	V	P	K	L	E	A	L	G	T	D	F	A	K	E	I	L	D	L	K	Q	Y
				L	V	K	K	S	Q	W	I	I	G	G	D	G	W	A	Y	D	I	G	Y	G	G	L	D	H	V	L	A	S	N
				E	D	I	N	V	L	V	M	D	T	E	V	Y	S	N	T	G	G	Q	A	S	K	A	T	P	T	G	S	V	A
				K	F	A	A	A	G	K	P	V	K	K	K	D	L	A	A	I	A	M	S	Y	G	H	I	Y	V	A	Q	V	S
				M	G	A	N	Q	Q	Q	F	I	K	A	V	K	E	A	E	A	H	Q	G	P	S	I	I	I	A	Y	S	P	C
				I	N	H	G	I	K	K	G	M	S	K	S	Q	T	E	M	K	L	A	T	E	C	G	Y	W	P	I	F	R	Y
				N	P	S	L	E	K	I	G	K	N	P	L	Q	I	D	S	K	E	P	K	W	E	K	Y	E	E	Y	L	S	G
				E	V	R	Y	Q	T	L	A	K	S	N	P	E	E	A	K	D	L	F	E	K	N	K	K	D	A	Q	K	R	W
				R	Q	Y	K	R	M	A	A	L	D	Y	S	E	E	K	E	A	E												
				1				5					10					15					20					25					30
29	44.58%	99.0%	>sp|Q8RIA5|HLDD_FUSNN ADP-L-glycero-D-manno-heptose-6-epimerase OS=Fusobacterium nucleatum subsp. nucleatum (strain ATCC 25586 / CIP 101130 / JCM 8532 / LMG 13131) OX=190304 GN=hldD PE=3 SV=1																														
				M	I	I	V	T	G	G	A	G	M	I	G	S	A	F	V	W	K	L	N	E	M	G	I	K	D	I	L	I	V
				D	K	L	R	K	E	D	K	W	L	N	I	R	K	R	E	Y	Y	D	W	I	D	K	D	N	L	K	E	W	L
				V	C	K	E	N	A	D	N	I	E	A	V	I	H	M	G	A	C	S	A	T	T	E	T	D	A	D	F	L	M
				D	N	N	F	G	Y	T	K	F	L	W	N	F	C	A	E	K	N	I	K	Y	I	Y	A	S	S	A	A	T	Y
				G	M	G	E	L	G	Y	N	D	D	V	S	P	E	E	L	Q	K	L	M	P	L	N	K	Y	G	Y	S	K	K
				F	F	D	D	W	A	F	K	Q	K	N	Q	P	K	Q	W	N	G	L	K	F	F	N	V	Y	G	P	Q	E	Y
				H	K	G	R	M	A	S	M	V	F	H	T	Y	N	Q	Y	K	E	N	G	Y	V	K	L	F	K	S	Y	K	E
				G	F	K	D	G	E	Q	L	R	D	F	V	Y	V	K	D	V	V	D	I	M	Y	F	M	L	V	N	D	V	K
				S	G	I	Y	N	I	G	T	G	K	A	R	S	F	M	D	L	S	M	A	T	M	R	A	A	S	H	N	D	N
				L	D	K	N	E	V	V	K	L	I	E	M	P	E	D	L	Q	G	R	Y	Q	Y	F	T	E	A	K	I	N	K
				L	R	E	I	G	Y	T	K	E	M	H	S	L	E	E	G	V	K	D	Y	V	Q	N	Y	L	A	K	E	D	S
				Y	L																												
				1				5					10					15					20					25					30
30	38.25%	99.0%	>sp|Q8RI55|ENO_FUSNN Enolase OS=Fusobacterium nucleatum subsp. nucleatum (strain ATCC 25586 / CIP 101130 / JCM 8532 / LMG 13131) OX=190304 GN=eno PE=3 SV=1																														
				M	T	G	I	V	E	V	I	G	R	E	I	L	D	S	R	G	N	P	T	V	E	V	D	V	I	L	E	C	G
				A	K	G	R	A	A	V	P	S	G	A	S	T	G	S	H	E	A	V	E	L	R	D	E	D	K	G	R	Y	L
				G	K	G	V	L	K	A	V	N	N	V	N	T	E	I	R	E	A	L	L	G	M	D	A	L	N	Q	V	E	I
				D	K	V	M	L	E	L	D	R	T	P	N	K	G	R	L	G	A	N	A	I	L	G	V	S	L	A	V	A	K
				A	A	A	E	A	L	G	Q	P	L	Y	K	Y	L	G	G	V	N	S	K	E	L	P	L	P	M	M	N	I	L
				N	G	G	A	H	A	D	S	A	V	D	L	Q	E	F	M	I	Q	P	V	G	A	K	S	F	R	E	A	M	Q
				M	G	A	E	V	F	H	H	L	G	K	I	L	K	A	N	G	D	S	T	N	V	G	N	E	G	G	Y	A	P
				S	K	I	Q	G	T	E	G	A	L	A	L	I	S	E	A	V	K	A	A	G	Y	E	L	G	K	D	I	T	F
				A	L	D	A	A	S	S	E	F	C	K	E	V	N	G	K	Y	E	Y	H	F	K	R	E	G	G	V	V	R	T
				T	D	E	M	I	K	W	Y	E	E	L	I	N	K	Y	P	I	V	S	I	E	D	G	L	G	E	D	D	W	D
				G	W	V	K	L	T	K	A	I	G	D	R	V	Q	I	V	G	D	D	L	F	V	T	N	T	E	R	L	K	K
				G	I	E	L	G	A	G	N	S	I	L	I	K	L	N	Q	I	G	S	L	T	E	T	L	D	A	I	E	M	A
				K	R	A	G	Y	T	A	V	V	S	H	R	S	G	E	T	E	D	A	T	I	A	D	V	A	V	A	T	N	A
				G	Q	I	K	T	G	S	T	S	R	T	D	R	M	A	K	Y	N	Q	L	L	R	I	E	E	E	L	G	S	V
				A	Q	Y	N	G	R	D	V	F	Y	N	I	K	K																
				1				5					10					15					20					25					30
31	26.00%	89.9%	>tr|Q7M144|Q7M144_FUSNU Major outer membrane protein (Fragment) OS=Fusobacterium nucleatum OX=851 PE=1 SV=1																														
				K	E	V	M	P	A	P	T	P	A	P	E	K	V	V	E	Y	V	E	K	P	V	I	V	Y	A	X	E	V	A
				P	A	W	R	P	N	G	S	V	D	V	Q	Y	K	W	Y	G	E	V	E										
				1				5					10					15					20					25					30
32	32.17%	99.0%	>tr|Q8RIC2|Q8RIC2_FUSNN LPS biosynthesis protein WbpG OS=Fusobacterium nucleatum subsp. nucleatum (strain ATCC 25586 / CIP 101130 / JCM 8532 / LMG 13131) OX=190304 GN=FN1679 PE=4 SV=1																														
				M	K	Y	C	K	N	C	L	Q	P	D	T	R	P	G	I	K	F	N	E	D	G	V	C	Y	A	C	L	Y	E
				K	E	K	E	K	I	D	W	K	S	R	E	K	E	L	K	E	I	A	E	W	A	K	K	T	S	K	G	P	Y
				D	C	V	I	G	V	S	G	G	K	D	S	T	F	Q	A	I	Y	A	K	E	K	L	G	L	H	V	L	L	V
				N	G	E	P	D	Q	I	T	E	I	G	R	K	N	I	E	N	L	I	Q	K	G	F	D	I	I	K	L	R	P
				N	P	K	I	T	K	Q	L	V	K	T	A	F	L	K	Y	G	N	P	M	K	S	T	E	Y	P	L	W	T	S
				A	Y	I	I	A	D	K	F	D	I	P	L	I	I	Q	G	E	N	A	A	L	T	L	G	V	V	N	T	K	L
				G	V	D	G	N	A	M	N	V	D	G	G	N	T	L	N	G	C	N	A	S	D	W	V	E	D	G	L	P	L
				E	D	L	Y	M	Y	Q	F	P	D	K	E	K	M	A	K	K	G	I	K	A	I	W	L	Q	Y	Y	A	K	E
				W	S	Q	V	Y	N	A	D	F	S	I	A	R	G	L	L	G	R	T	V	E	D	L	H	D	L	G	R	Y	R
				R	Y	S	S	L	D	S	N	L	H	I	V	N	Q	M	L	K	Y	Y	K	F	G	F	G	F	A	T	D	E	A
				C	Y	D	I	R	E	G	R	L	T	R	D	D	A	I	W	L	I	N	E	Y	D	G	K	C	G	K	Q	Y	I
				D	E	F	C	K	Y	I	E	I	T	E	E	E	F	W	N	T	V	D	Q	Y	V	N	K	E	L	F	E	K	K
				D	G	K	W	I	P	K	F	K	V	G	K	N																	
				1				5					10					15					20					25					30
33	24.22%	99.0%	>sp|Q8RH05|DNAK_FUSNN Chaperone protein DnaK OS=Fusobacterium nucleatum subsp. nucleatum (strain ATCC 25586 / CIP 101130 / JCM 8532 / LMG 13131) OX=190304 GN=dnaK PE=3 SV=1																														
				M	S	K	I	I	G	I	D	L	G	T	T	N	S	C	V	A	V	M	E	G	G	S	A	T	I	I	P	N	S
				E	G	A	R	T	T	P	S	V	V	N	I	K	D	N	G	E	V	V	V	G	E	I	A	K	R	Q	A	V	T
				N	P	T	S	T	V	S	S	I	K	T	H	M	G	S	D	Y	K	V	E	I	F	G	K	K	Y	T	P	Q	E
				I	S	A	K	T	L	Q	K	L	K	K	D	A	E	A	Y	L	G	E	E	V	K	E	A	V	I	T	V	P	A
				Y	F	T	D	S	Q	R	Q	A	T	K	D	A	G	T	I	A	G	L	D	V	K	R	I	I	N	E	P	T	A
				A	A	L	A	Y	G	L	E	K	K	K	E	E	K	V	L	V	F	D	L	G	G	G	T	F	D	V	S	V	L
				E	I	S	D	G	V	I	E	V	I	S	T	A	G	N	N	H	L	G	G	D	D	F	D	N	E	I	I	N	W
				L	V	A	E	F	K	K	E	T	G	I	D	L	S	N	D	K	M	A	Y	Q	R	L	K	D	A	A	E	K	A
				K	K	E	L	S	T	L	M	E	T	S	I	S	L	P	F	I	T	M	D	A	T	G	P	K	H	L	E	M	K
				L	T	R	A	K	F	N	D	L	T	K	H	L	V	E	A	T	Q	G	P	T	K	T	A	L	K	D	A	S	L
				E	A	N	Q	I	D	E	I	L	L	V	G	G	S	T	R	I	P	A	V	Q	E	W	V	E	N	F	F	G	K
				K	P	N	K	G	I	N	P	D	E	V	V	A	A	G	A	A	I	Q	G	G	V	L	M	G	D	V	K	D	V
				L	L	L	D	V	T	P	L	S	L	G	I	E	T	L	G	G	V	F	T	K	M	I	E	K	N	T	T	I	P
				V	K	K	S	Q	V	Y	S	T	A	V	D	N	Q	P	A	V	T	I	N	V	L	Q	G	E	R	S	R	A	T
				D	N	H	K	L	G	E	F	N	L	E	G	I	P	A	A	P	R	G	V	P	Q	I	E	V	T	F	D	I	D
				A	N	G	I	V	H	V	S	A	K	D	L	G	T	G	K	E	N	K	V	T	I	S	G	S	S	N	L	S	K
				E	E	I	E	R	M	T	K	E	A	E	A	H	A	E	E	D	K	K	F	Q	E	L	V	E	A	R	N	K	A
				D	Q	L	I	S	A	T	E	K	T	L	K	E	N	P	D	K	V	S	E	E	D	K	K	N	I	E	A	A	I
				E	E	L	K	K	V	K	D	G	D	D	K	S	A	I	D	S	A	M	E	K	L	S	Q	A	S	H	K	F	A
				E	E	L	Y	K	E	V	Q	A	Q	A	Q	A	Q	Q	Q	A	G	A	N	A	G	S	D	K	K	D	E	D	V
				A	E	A	E	V	V	D																							
				1				5					10					15					20					25					30
34	22.81%	99.0%	>sp|Q8RFY7|GSA_FUSNN Glutamate-1-semialdehyde 21-aminomutase OS=Fusobacterium nucleatum subsp. nucleatum (strain ATCC 25586 / CIP 101130 / JCM 8532 / LMG 13131) OX=190304 GN=hemL PE=3 SV=1																														

				M	V	F	K	N	S	I	D	L	Y	K	K	A	V	E	L	I	P	G	G	V	N	S	P	V	R	A	F	K	S
				V	N	R	E	A	P	I	F	I	K	K	G	Q	G	A	K	I	W	D	E	D	D	N	E	Y	I	D	Y	I	C
				S	W	G	P	L	I	L	G	H	N	H	P	K	V	I	E	E	V	K	K	I	I	E	N	G	S	S	Y	G	L
				P	T	K	Y	E	V	D	L	A	E	L	I	V	D	I	V	P	S	I	E	K	V	R	L	T	T	S	G	T	E
				A	T	M	S	A	V	R	L	A	R	A	Y	T	Q	R	N	K	I	L	K	F	E	G	C	Y	H	G	H	S	D
				A	L	L	V	K	S	G	S	G	L	L	T	E	G	Y	Q	D	S	N	G	I	T	D	G	V	L	K	D	T	L
				T	L	P	F	G	D	I	E	K	V	K	E	I	L	K	N	K	D	V	A	C	V	I	V	E	P	I	P	A	N
				M	G	L	I	E	T	H	K	E	F	L	Q	G	L	G	K	V	T	E	K	T	G	T	I	L	I	F	D	E	V
				I	S	G	F	R	L	A	L	G	G	A	Q	E	F	F	G	I	T	P	D	L	T	T	L	G	K	I	I	G	G
				G	Y	P	V	G	A	F	G	G	K	K	E	I	M	D	L	V	A	P	V	G	R	V	Y	H	A	G	T	L	S
				G	N	P	I	A	S	K	A	G	F	A	T	I	S	Y	L	K	E	N	P	N	I	Y	K	E	L	E	E	K	T
				N	Y	L	I	D	N	I	E	I	L	A	K	K	Y	S	V	N	V	C	V	N	S	M	G	S	L	F	T	I	F
				F	V	D	I	D	K	V	E	N	L	E	D	S	L	K	S	N	T	E	N	F	S	I	Y	F	N	T	M	L	E
				N	G	I	V	I	P	P	S	Q	F	E	A	H	F	L	S	M	A	H	T	K	K	E	L	N	R	T	L	E	V
				I	E	M	A	F	K	K	I	G	E	K	S	G	K																
				1				5					10					15					20					25					30
35	30.25%	99.0%	>tr|D5RDJ3|D5RDJ3_FUSN2 Transcription termination/antitermination protein NusA OS=Fusobacterium nucleatum subsp. nucleatum (strain ATCC 23726 / VPI 4351) OX=525283 GN=nusA PE=3 SV=1																														
				M	K	A	K	D	S	K	N	F	L	E	A	L	D	E	L	E	R	E	K	G	I	S	K	E	S	V	L	E	A
				I	E	L	A	L	L	A	A	Y	K	K	N	Y	G	E	D	E	N	V	E	V	I	V	D	R	E	N	G	D	I
				K	V	F	A	S	K	I	I	V	N	A	D	D	L	L	D	P	N	K	E	I	S	L	E	D	A	K	K	I	K
				K	R	V	K	V	G	D	T	L	K	F	E	V	N	C	E	D	F	R	R	N	A	V	Q	N	G	K	Q	I	V
				I	Q	K	V	R	E	A	E	R	E	H	I	F	N	K	F	K	E	R	E	D	S	I	V	T	G	I	I	R	R
				I	D	N	R	K	N	I	F	I	E	I	D	G	I	E	L	I	L	P	P	A	E	Q	S	I	S	D	V	Y	R
				V	G	E	R	I	K	V	Y	I	L	S	V	E	K	T	N	K	F	P	K	I	L	I	S	R	K	N	E	G	L
				L	K	K	L	F	E	I	E	I	P	E	I	T	S	G	I	I	E	I	K	S	V	A	R	E	A	G	S	R	A
				K	V	A	V	Y	S	E	V	P	N	I	D	T	V	G	A	C	I	G	Q	R	G	A	R	I	K	N	I	V	D
				E	L	N	G	E	R	I	D	I	V	E	W	K	P	V	I	E	E	F	V	S	A	V	L	S	P	A	V	V	S
				N	V	T	I	L	E	D	G	T	A	R	V	L	V	E	P	S	Q	L	S	L	A	I	G	K	N	G	Q	N	A
				R	L	A	A	R	L	T	G	M	R	V	D	I	K	V	I	D	S	E	A	L	K	E	E	E	N	E			
				1				5					10					15					20					25					30
36	38.33%	99.0%	>tr|D5RBA6|D5RBA6_FUSN2 Cell division protein FtsZ OS=Fusobacterium nucleatum subsp. nucleatum (strain ATCC 23726 / VPI 4351) OX=525283 GN=ftsZ PE=3 SV=1																														
				M	T	D	A	I	K	D	L	V	K	I	K	V	I	G	V	G	G	G	G	G	N	A	I	N	D	M	L	Y	S
				G	V	T	G	V	E	Y	I	A	A	N	T	D	K	Q	D	L	E	K	S	L	A	D	V	K	L	Q	I	G	E
				K	L	T	K	G	Q	G	A	G	A	S	P	E	T	G	R	L	A	A	E	E	D	I	E	K	I	Q	E	L	L
				K	G	T	D	M	L	F	I	T	A	G	M	G	G	G	T	G	T	G	A	A	P	V	I	A	K	A	A	K	E
				L	D	V	L	T	V	A	V	V	T	K	P	F	N	F	E	G	E	R	R	K	N	N	A	E	S	G	I	E	L
				L	R	Q	N	V	D	S	L	V	I	I	P	N	D	K	L	F	D	L	P	D	K	S	I	T	L	Q	N	A	F
				K	E	A	N	N	I	L	R	I	G	I	K	A	V	V	D	L	V	L	G	Q	G	F	I	N	L	D	F	A	D
				I	K	S	V	L	K	D	S	D	I	A	V	L	G	F	G	D	G	E	G	E	N	R	A	M	K	A	A	E	K
				A	L	Q	S	P	L	L	E	K	S	I	Q	G	A	D	K	I	L	I	N	L	M	T	S	Q	D	V	G	L	S
				E	S	Q	T	V	T	D	V	I	R	Q	A	A	G	K	K	I	E	D	V	M	F	G	V	T	I	V	P	E	F
				T	D	R	I	E	I	T	I	I	A	N	N	F	K	E	G	V	D	S	N	T	D	S	P	I	R	M	D	S	A
				K	P	A	E	P	L	K	E	T	E	R	K	K	E	P	E	E	E	I	D	I	P	P	W	M	R	N	N	K	R
				1				5					10					15					20					25					30
37	38.38%	99.0%	>tr|D5REY0|D5REY0_FUSN2 Glycerophosphodiester phosphodiesterase family protein OS=Fusobacterium nucleatum subsp. nucleatum (strain ATCC 23726 / VPI 4351) OX=525283 GN=glpQ PE=4 SV=1																														
				M	K	L	K	S	C	L	V	L	L	G	I	L	S	S	T	A	L	F	A	A	H	N	G	K	I	I	I	A	H
				R	G	A	S	G	Y	L	P	E	H	T	L	E	S	K	A	L	A	F	A	Q	Q	A	D	Y	L	E	Q	D	L
				A	M	S	K	D	G	K	L	I	V	I	H	D	H	F	L	D	G	L	T	D	V	A	K	K	F	P	N	R	K
				R	A	D	G	R	Y	Y	V	I	D	F	T	W	P	E	L	Q	T	L	E	M	T	E	N	F	T	T	K	D	G
				K	Q	T	A	V	Y	P	N	R	F	P	L	W	K	S	D	F	K	L	H	T	F	E	E	E	I	E	F	I	Q
				G	L	E	K	S	T	G	K	K	I	G	I	Y	P	E	I	K	A	P	W	F	H	H	Q	N	G	K	D	I	A
				K	A	T	L	E	V	L	K	K	Y	G	Y	T	K	K	S	D	M	V	Y	L	Q	T	F	D	Y	N	E	L	K
				R	I	K	T	E	L	M	P	K	M	G	M	D	L	K	L	V	Q	L	I	A	Y	N	D	W	H	E	T	E	E
				K	D	K	N	G	K	W	V	N	Y	D	Y	D	W	M	F	K	E	G	A	M	K	E	I	A	K	Y	A	D	G
				V	G	P	G	W	Y	M	L	I	D	D	K	N	S	K	V	G	N	I	V	Y	T	P	M	V	K	D	I	A	T
				T	K	M	E	L	H	P	Y	T	V	R	K	D	A	L	P	E	F	F	T	D	V	N	Q	M	Y	D	A	L	L
				N	K	A	G	A	T	G	V	F	T	D	F	P	D	L	G	V	Q	F	L	E	N	Q	K	N	K	K			
				1				5					10					15					20					25					30
38	19.38%	99.0%	>tr|Q8RGM9|Q8RGM9_FUSNN Formate acetyltransferase OS=Fusobacterium nucleatum subsp. nucleatum (strain ATCC 25586 / CIP 101130 / JCM 8532 / LMG 13131) OX=190304 GN=FN0262 PE=4 SV=1																														
				M	E	A	W	R	G	F	K	S	G	E	W	Q	N	S	I	N	V	S	D	F	I	K	H	N	Y	T	E	Y	L
				G	D	E	S	F	L	E	G	P	T	E	N	T	K	K	L	W	D	I	L	S	G	M	L	K	I	E	R	E	K
				G	I	Y	D	A	E	T	K	T	P	S	K	I	D	A	Y	G	A	G	Y	I	S	K	D	L	E	T	I	V	G
				L	Q	T	D	A	P	L	K	R	A	I	F	P	N	G	G	L	R	M	V	E	S	S	L	E	A	F	G	Y	K
				L	D	P	T	T	K	E	I	Y	E	K	Y	R	K	S	H	N	A	G	V	F	S	A	Y	T	P	A	I	K	A
				A	R	H	T	G	V	I	T	G	L	P	D	A	Y	G	R	G	R	I	I	G	D	Y	R	R	V	A	L	Y	G
				V	D	R	L	I	E	E	R	K	R	E	F	D	A	Y	D	P	E	E	M	T	E	D	V	I	R	D	R	E	E
				M	F	E	Q	L	E	A	L	K	A	L	K	R	M	A	A	A	Y	G	F	D	I	G	R	P	A	E	T	A	Q
				E	A	I	Q	W	T	Y	F	G	Y	L	G	A	I	K	D	Q	N	G	A	A	M	S	L	G	K	T	A	G	F
				L	D	V	Y	I	E	R	D	L	K	E	G	R	I	T	E	K	Q	A	Q	E	F	I	D	H	F	I	M	K	L
				R	I	V	R	F	L	R	T	P	E	Y	D	Q	L	F	S	G	D	P	V	W	V	T	E	S	I	G	G	M	N
				N	D	G	K	S	W	V	T	K	N	A	Y	R	Y	L	N	T	L	Y	N	L	G	T	A	P	E	P	N	L	T
				I	L	W	S	E	R	L	P	E	N	W	K	K	F	C	S	K	V	S	I	D	T	S	S	L	Q	Y	E	N	D
				D	I	M	R	P	Q	F	G	E	D	Y	G	I	A	C	C	V	S	P	M	A	I	G	K	Q	M	Q	F	F	G
				A	R	A	N	L	P	K	A	L	L	Y	A	I	N	G	G	K	D	E	L	K	K	D	Q	V	T	P	A	G	Q
				F	E	R	I	T	S	D	Y	L	D	F	D	E	V	W	E	K	Y	D	K	M	L	T	W	L	A	S	T	Y	I
				K	A	L	N	I	I	H	Y	M	H	D	K	Y	S	Y	E	A	L	E	M	A	L	H	S	L	D	I	K	R	T
				E	A	C	G	I	A	G	L	S	I	V	A	D	S	L	A	A	I	K	Y	G	K	V	R	V	I	R	D	E	A
				G	D	A	V	D	Y	V	V	E	Q	P	Y	V	P	F	G	N	N	D	D	R	T	D	E	L	A	V	K	V	V
				R	T	F	M	N	K	I	R	S	H	K	M	Y	R	D	A	E	P	T	Q	S	V	L	T	I	T	S	N	V	V
				Y	G	K	K	T	G	N	T	P	D	G	R	R	A	G	A	P	F	G	P	G	A	N	P	M	H	G	R	D	T
				K	G	A	V	A	S	L	A	S	V	A	K	L	P	F	E	D	A	N	D	G	I	S	Y	T	F	A	I	T	P
				E	T	L	G	K	T	D	D	E	K	K	N	N	L	V	G	L	L	D	G	Y	F	K	Q	T	G	H	H	L	N
				V	N	V	F	G	R	E	L	L	E	D	A	M	E	H	P	E	N	Y	P	Q	L	T	I	R	V	S	G	Y	A
				V	N	F	I	K	L	T	R	E	Q	Q	L	D	V	I	N	R	T	I	S	N	K	M							
				1				5					10					15					20					25					30
39	28.72%	99.0%	>sp|Q8RGE5|METK_FUSNN S-adenosylmethionine synthase OS=Fusobacterium nucleatum subsp. nucleatum (strain ATCC 25586 / CIP 101130 / JCM 8532 / LMG 13131) OX=190304 GN=metK PE=3 SV=1																														
				M	K	K	F	T	Y	F	T	S	E	F	V	S	P	G	H	P	D	K	I	S	D	Q	I	S	D	A	I	L	D
				A	C	L	K	D	D	P	N	S	R	V	A	C	E	V	F	C	T	T	G	L	V	V	V	G	G	E	I	T	T
				S	T	Y	I	D	V	Q	D	I	V	R	K	K	I	D	E	I	G	Y	R	P	G	M	G	F	D	S	N	C	G
				T	L	S	C	I	H	A	Q	S	P	D	I	A	M	G	V	D	I	G	G	A	G	D	Q	G	I	M	F	G	G
				A	V	R	E	T	E	E	L	M	P	L	A	L	V	L	S	R	E	I	L	V	K	L	T	N	M	M	K	S	N
				E	I	E	W	A	R	P	D	Q	K	S	Q	V	T	L	A	Y	D	E	N	G	K	V	D	H	V	D	S	I	V
				V	S	V	Q	H	D	E	D	T	T	H	D	E	I	E	K	I	V	I	E	K	V	V	K	P	V	L	E	K	Y
				N	L	S	S	D	N	I	K	Y	Y	I	N	P	T	G	R	F	V	I	G	G	P	H	G	D	T	G	V	T	G
				R	K	I	I	V	D	T	Y	G	G	Y	F	K	H	G	G	G	A	F	S	G	K	D	P	S	K	V	D	R	S
				A	A	Y	A	A	R	W	V	A	K	N	I	V	A	A	E	L	A	D	K	C	E	I	Q	L	S	Y	A	I	G
				V	P	K	P	V	S	V	K	V	D	T	F	G	T	S	K	V	D	E	D	K	I	S	E	A	V	S	K	V	F
				D	L	S	P	R	G	I	E	K	A	L	E	L	R	E	G	N	F	K	Y	Q	D	L	A	A	F	G	H	I	G
				R	T	D	I	D	T	P	W	E	R	L	N	K	V	D	E	L	K	K	A	I	E	L							
				1				5					10					15					20					25					30
40	33.53%	99.0%	>tr|Q8RED6|Q8RED6_FUSNN Phosphate acetyltransferase OS=Fusobacterium nucleatum subsp. nucleatum (strain ATCC 25586 / CIP 101130 / JCM 8532 / LMG 13131) OX=190304 GN=FN1172 PE=4 SV=1																														
				M	I	I	M	S	F	L	G	Q	V	R	K	K	A	L	Q	A	N	R	R	I	V	L	P	E	S	S	D	E	R
				I	I	R	A	T	S	Q	I	L	K	E	G	L	A	Q	V	V	L	V	G	N	Q	E	A	I	M	H	S	A	K
				A	Y	E	V	S	L	S	G	A	K	I	V	D	P	Y	N	F	E	R	L	N	D	Y	I	N	K	L	V	E	L
				R	A	K	K	G	M	T	P	E	E	A	K	K	I	L	L	N	D	P	T	F	F	G	A	M	L	V	R	M	G
				D	A	D	G	M	V	S	G	S	A	S	P	T	A	N	V	L	R	A	A	I	Q	V	I	G	T	Q	P	G	V
				K	T	V	S	S	V	F	I	M	E	L	S	Q	F	K	D	L	F	G	S	I	L	V	F	G	D	C	S	V	I
				P	F	P	T	S	E	Q	L	A	D	I	A	T	S	A	A	E	T	A	I	K	I	A	G	I	N	P	R	V	A
				L	M	T	F	S	T	K	G	S	A	K	H	E	C	V	D	R	V	I	E	A	G	H	I	L	R	E	R	K	V
				Q	F	R	F	D	A	E	L	Q	A	D	A	A	L	V	K	S	I	G	E	I	K	A	P	L	S	D	V	S	G
				N	A	N	V	L	I	F	P	T	L	S	A	G	N	I	G	Y	K	L	V	Q	R	L	A	G	A	N	A	Y	G
				P	I	I	Q	G	L	N	A	P	V	N	D	L	S	R	G	C	S	V	E	D	I	V	V	L	T	A	I	T	S
				A	Q	A	C	I	D	C																							
				1				5					10					15					20					25					30
41	26.70%	99.0%	>tr|Q8RGC9|Q8RGC9_FUSNN Iron(III)-binding protein OS=Fusobacterium nucleatum subsp. nucleatum (strain ATCC 25586 / CIP 101130 / JCM 8532 / LMG 13131) OX=190304 GN=FN0375 PE=4 SV=1																														
				M	K	K	F	I	K	F	L	L	M	S	M	G	M	I	F	M	L	V	A	C	G	G	D	K	E	K	T	E	A
				T	P	E	T	Q	G	S	N	E	L	V	I	Y	S	P	N	A	D	D	E	V	N	K	I	I	P	A	F	E	E
				A	T	G	I	K	V	I	L	Q	S	M	G	S	G	D	V	L	A	R	I	S	A	E	K	E	N	P	Q	A	D
				I	N	W	G	A	I	S	M	G	V	L	A	T	T	P	D	L	W	E	S	Y	T	S	E	N	E	K	N	V	P
				D	A	Y	K	N	T	T	G	F	F	T	N	Y	K	L	D	G	S	A	A	L	L	V	N	K	D	V	F	K	K
				L	G	L	D	P	E	K	F	N	G	Y	K	D	L	L	W	P	E	L	K	G	K	I	A	M	G	D	P	T	A
				S	S	S	A	I	A	E	L	T	N	M	L	L	V	M	G	E	K	P	Y	D	E	K	A	W	E	F	I	E	K
				F	I	G	Q	L	D	G	T	I	L	S	S	S	S	Q	I	Y	K	A	T	A	D	G	E	Y	A	V	G	V	T
				Y	E	N	P	A	V	T	L	L	Q	D	G	A	T	N	L	K	L	V	Y	P	E	E	G	S	V	W	L	P	G
				A	A	A	I	V	K	N	A	P	H	M	E	N	A	K	K	F	I	D	F	L	I	S	D	E	G	Q	K	V	V
				A	E	T	S	T	R	P	V	N	T	S	I	K	N	T	S	E	F	I	R	P	F	D	E	I	K	V	A	Y	E
				D	I	P	Y	C	A	E	H	R	K	E	W	Q	E	R	W	T	N	I	L	T	K								
				1				5					10					15					20					25					30
42	32.15%	99.0%	>sp|Q8RDT4|MEGL_FUSNN L-methionine gamma-lyase OS=Fusobacterium nucleatum subsp. nucleatum (strain ATCC 25586 / CIP 101130 / JCM 8532 / LMG 13131) OX=190304 GN=FN1419 PE=1 SV=1																														
				M	E	M	K	K	S	G	L	G	T	T	A	I	H	A	G	T	L	K	N	L	Y	G	T	L	A	M	P	I	Y
				Q	T	S	T	F	I	F	D	S	A	E	Q	G	G	R	R	F	A	L	E	E	A	G	Y	I	Y	T	R	L	G
				N	P	T	T	T	V	L	E	N	K	I	A	A	L	E	E	G	E	A	G	I	A	M	S	S	G	M	G	A	I

			S S T L W T V L K A G D H V V T D K T L Y G C T F A L M N H
			G L T R F G V E V T F V D T S N L E E V K N A M K K N T R V
			V Y L E T P A N P N L K I V D L E A L S K I A H T N P N T L
			V I V D N T F A T P Y M Q K P L K L G V D I V V H S A T K Y
			L N G H G D V I A G L V V T R Q E L A D Q I R F V G L K D M
			T G A V L G P Q E A Y Y I I R G L K T F E I R M E R H C K N
			A R T I V D F L N K H P K V E K V Y Y P G L E T H P G Y E I
			A K K Q M K D F G A M I S F E L K G G F E A G K T L L N N L
			K L C S L A V S L G D T E T L I Q H P A S M T H S P Y T K E
			E R E V A G I T D G L V R L S V G L E N V E D I I A D L E Q
			G L E K I
			1 5 10 15 20 25 30
43	26.95%	99.0%	>tr|Q8R6C5|Q8R6C5_FUSNN Cysteine desulfurase OS=Fusobacterium nucleatum subsp. nucleatum (strain ATCC 25586 / CIP 101130 / JCM 8532 / LMG 13131) OX=190304 GN=FN0058 PE=3 SV=1
			M K V Y L D N N A T T K V D E E V V K A M L P Y F S D Y Y G
			N P F S L H L F G A E T G K A V T E A R Q T I A D I L K A K
			P N E I I F T A S G S E A D N L A I R G I A K A Y K H R G K
			H I I T S T I E H P A V K N T F M D L I E D G F E V T M V P
			V D E N G V M I I D E F K K V L R E D T I L V S V M H A N N
			E V G S F Q P I E E I A K I T K E R K I I L H V D A V Q T M
			G K V E I Y P E R M G I D L L S F S G H K F H A P K G I G V
			L Y K R D G V R L A R I I T G G N Q E G K R R P G T S N V P
			Y I V G L A K A L E I S V A N M K E E W N R E E T L R N Y F
			E D E V S K R I P E I K I N G K E A R R L P G T S S I T F K
			Y L E G E S M L L N L S L K G I A V S S G S A C S S D S L Q
			P S H V L L A M G I P A E F A H G T L R F S L S K Y T T K E
			E I D Y T I E S L V E I I G K L R E L S P L W K I F K D N K
			L T N E A S F
			1 5 10 15 20 25 30
44	21.56%	99.0%	>tr|Q8RIJ9|Q8RIJ9_FUSNN RNFB-related protein OS=Fusobacterium nucleatum subsp. nucleatum (strain ATCC 25586 / CIP 101130 / JCM 8532 / LMG 13131) OX=190304 GN=FN1591 PE=4 SV=1
			M E A I M M P V A V L G I T G V L M G L F L A Y A S K K F E
			V E V D P K V E A I L A I L P G A N C G A C G F P G C A G Y
			A S G V A L E G A K M T L C A P G G P K V I E K I G E I M G
			V A V E I P V K K K P A K K P V E K K E A P K A Q T G E P I
			S A S Q E F I E K N K R M L M K F K E A F D A G D K E G F E
			K L E N L A K M A K K D E L L K Y Y E E I K A G K I V P D G
			S A P V A A G T T N A N A I S A S K E F V E K N K R M L M K
			F K E A F D A G D K E G F E K L E N L A K M A K K D E L L K
			F Y E E I K A G K I V P D P A T M T D A V A A V K A E V I S
			A T K E F V E K N K R M L M K F K E A F D A G D K E G F E K
			L E N L A K M A K K D E L L K F Y E E I K A G K T V P D P A
			T M T D T P A A K Q E A P K V E D T K K Q E A S Y C S V L G
			D G L C V P E Q N E K V K E N L H Q E I D K E V K
			1 5 10 15 20 25 30
45	20.29%	99.0%	>sp|Q8RGJ5|SYH_FUSNN Histidine--tRNA ligase OS=Fusobacterium nucleatum subsp. nucleatum (strain ATCC 25586 / CIP 101130 / JCM 8532 / LMG 13131) OX=190304 GN=hisS PE=3 SV=1
			M K L I K A V R G T K D I I G E E A K K Y I Y I S N V A Q K
			M F E N Y G Y N F V K T P I F E E T E L F K R G I G E A T D
			V V E K E M Y T F K D R G D R S I T L R P E N T A S L V R C
			Y L E N A I Y A K E E I S R F Y Y N G S M F R Y E R P Q A G
			R Q R E F N Q I G L E V F G E K S P K V D A E V I A I G Y K
			F L E K L G I T D L E V K I N S V G S K A S R T V Y R E K L
			I E H F K S H L D D M C E D C R D R I N R N P L R L L D C K
			V D G E K D F Y K S A P S I I D Y L F E D E R K H Y D D V K
			K Y L D I F G I K Y T E D P T L V R G L D Y Y S S T V F E I
			V T N K L G S Q G T V L G G G R Y D N L L K E L G D K D I P
			A V G F A T G V E R I M M L L E E N Y P K N T P D V Y I A W
			L G E N T S E T A L K I A E S L R D N D I K V Y I D Y S E K
			G M K S H M K K A D K L E T R Y C V I L G E D E F N K G I V
			L L K D F S T R E Q K E I K I E E I I N Y I K
			1 5 10 15 20 25 30
46	18.32%	99.0%	>tr|Q8RGS8|Q8RGS8_FUSNN Glutaconyl-CoA decarboxylase A subunit OS=Fusobacterium nucleatum subsp. nucleatum (strain ATCC 25586 / CIP 101130 / JCM 8532 / LMG 13131) OX=190304 GN=FN0204 PE=4 SV=1
			M N Y S M P K Y F Q N M P Q V G K A L A N I D E A N E N A V
			K E I E A A I A D S V A A M Q D S G T S D E K I H D K G Q M
			T A L E R V A E L V D E G T W Y P L N T L Y N P E N F E T G
			T G I V K G L G R I G G K W A V V V A S D N K K I V G A W V
			P G Q A D N L L R A S D T A K C L G I P L V Y V L N C S G V
			K L D E Q E K V Y A N R R G G G T P F F R N A E L Q Q L G I
			P V I V G I Y G T N P A G G G Y H S I S P T I L I A H K D A
			N M A V G G A G I V G G M N P K G Y I D M E G A I Q I A E A
			T M A A K K V E V P G T I H V H Y D K T G F F R E V Y D D E
			I G V I D G I K K Y M D Y L P A Y D L E F F R V D E P T E P
			A L D P N D L Y S I I P M N Q K K I Y N I Y D V I G R L F D
			N S E F S E Y K K G Y G P E V V T G I A K V D G L L V G V I
			A N A Q G L L M N Y P E Y R E K A V G I G G K L Y R Q G L I
			K M S E F V T L C S R D R L P I V W L Q D T S G I D V G N P
			A E E A E L L G L G Q S L I Y S I E N S H V P Q I E I T I R
			K G S A A A H Y V L G G P Q G N N T N A F S L G T A A T E V
			Y V M N G E T A A S A M Y S R R L A K D Y K A G K D L Q P T
			I D K M N Q L I N E Y T A K S R P A Y C A K T G M V D E I V
			P L Y D L R G Y I S A F A N A V Y Q N P K S I C A F H Q M I
			L P R A I R E F E T Y T K K
			1 5 10 15 20 25 30
47	24.27%	99.0%	>tr|Q8RGB0|Q8RGB0_FUSNN Dipeptide-binding protein OS=Fusobacterium nucleatum subsp. nucleatum (strain ATCC 25586 / CIP 101130 / JCM 8532 / LMG 13131) OX=190304 GN=FN0396 PE=4 SV=1
			M K K K F W L L M A M V L S V L F L V A C G G D P D K K S D
			A G A G G K R D T L V I G N G A D A K S L D P H A S N D N P
			S S N V R V Q I F D T L M D L D D D G I P Q P M L A E S W E
			R P D D K T I I F H L R K G V K F H N G D E M K A S D V K F
			S L E R A L K S P E V S H I L A G I N G V E V L D D Y T V K
			V T T E K P M A A I L N N L S H T T I A I L S E K A T T E A
			G D K F G Q N P I G T G P Y K F V S W Q S G D R I T L E A F
			P D Y W R G E T P V K N I V F R N I V E E T N R T I G L E T
			G E L D I A Y D I K G L D K N K L K E D E R F T L L E G P Q
			V S I T Y L G F N L R K A P Y D N L K V R E A I S Y A I D Q
			K P I I D T V F L G A G E P A N S I I G P N I W G Y Y D V E
			K Y T Q D I E K A K A L L V E A G Y P N G F K A K I W V N D
			N P V R R D T A V I L Q D Q L K Q I G I D L T I E T V E W G
			A F L D G T A R G D H E M Y L L G W G T V T R D P D Y G M F
			E L I S S S T M G A A G N R S F Y S N P E V D K L L E A G K
			T E L D P E K R K D I Y K Q I Q E I V R R D I P M Y M I V Y
			P L Q N V I T K K D V K N F K L D A A Q T Y R L Y G V S I E
			E
			1 5 10 15 20 25 30
48	18.84%	99.0%	>tr|Q8RGG7|Q8RGG7_FUSNN Uncharacterized protein OS=Fusobacterium nucleatum subsp. nucleatum (strain ATCC 25586 / CIP 101130 / JCM 8532 / LMG 13131) OX=190304 GN=FN0331 PE=4 SV=1
			M Q E Q E F S L S Q E N N S G D K V E K E K E I I A L I N S
			K G T F T S E I T S E N K S L L V A N A E L I A Y I D C A T
			N E L H K T K T K I E W L V T S E E S K K N F I Y N I E K Y
			H I Y H L K V K E V D T D N F L L I D V L E R D L E N E L L
			K E T L K E C E Q K A A I V I E E P D L G K F I L D K N L K
			A F L S Q I E W L N P K K Q I G I S L N I G E N T R I K A L
			E K V G A F F V T L E K L L T N K K E W D K K L K V Y A A E
			N L V D L A N E L R K N L K G M F K F I K I W K W R F I G K
			I E L I S L A I N P N G E F V A T F D D K K L F V G H K I V
			V N G N I N G E L L N S A I V E N F N I E D Y K K V E L A L
			P A S E E K N E N T L D G I P A V D N N M N D K E E S K E
			1 5 10 15 20 25 30
49	11.20%	99.0%	>sp|Q8RG65|GLMS_FUSNN Glutamine--fructose-6-phosphate aminotransferase [isomerizing] OS=Fusobacterium nucleatum subsp. nucleatum (strain ATCC 25586 / CIP 101130 / JCM 8532 / LMG 13131) OX=190304 GN=glmS PE=3 SV=3
			M C G I I G Y S G S K A N A V E V L L E G L E K V E Y R G Y
			D S A G I A F V T D S G I Q I E K K E G K L E N L K N H M K
			N F E V L S C T G I G H T R W A T H G I P T D R N A H P H Y
			S E S K D V A L I H N G I I E N Y V E I K K E L L E Q G V K
			F S S D T D T E V V A Q L F S K L Y D G D L Y S T L K K V L
			K R I R G T Y A F A I I H K D F P D K M I C C R N H S P L I
			V G L G E H Q N F I A S D V S A I L K Y T R D I I Y L E D G
			D V V L V T K D N V T V Y D K D E K E V K R E V K K V E W N
			F E Q A S K G G Y A H F M I K E I E E Q P E I I E K T L N V
			Y T D K E K N V K F D E Q L E G I N F H D I D R I Y I V A C
			G T A Y Y A G L Q G Q Y F M K K L L G I D V F T D I A S E F
			R Y N D P V I T N K T L A I F V S Q S G E T I D T L M S M K
			Y A K E K G A R T L A I S N V L G S T I T R E A D N V I Y T
			L A G P E I S V A S T K A Y S S Q V L V M Y L L S L Y M G A
			K L G K I E E K D Y Q K Y I S D I S L L K E N V V K L I S E
			K E K I H D I A K K I K D I K N G F Y L G R G I D E K V A R
			E G S L K M K E I N Y I H T E A L P A G E L K H G S I A L I
			E K G V L V V A I S T N L E M D E K V V S N I K E V K A R G

				A	Y	V	V	G	A	C	K	E	G	S	L	V	P	E	V	V	D	D	V	I	Q	V	K	D	S	G	E	L	L
				T	P	V	L	T	V	V	G	L	Q	Y	L	A	Y	Y	T	S	L	E	K	G	Y	D	V	D	K	P	R	N	L
				A	K	S	V	T	V	E																							
				1				5					10					15					20					25					30
50	16.16%	99.0%	>sp|Q8R602|EFG_FUSNN Elongation factor G OS=Fusobacterium nucleatum subsp. nucleatum (strain ATCC 25586 / CIP 101130 / JCM 8532 / LMG 13131) OX=190304 GN=fusA PE=3 SV=1																														
				M	A	R	K	I	S	L	D	M	T	R	N	V	G	I	M	A	H	I	D	A	G	K	T	T	T	T	E	R	I
				L	F	Y	T	G	V	E	R	K	L	K	E	V	H	E	G	Q	A	T	M	D	W	M	E	Q	E	Q	E	R	G
				I	T	I	T	S	A	A	T	T	C	F	W	K	G	H	R	I	N	I	I	D	T	P	G	H	V	D	F	T	V
				E	V	E	R	S	L	R	V	L	D	G	A	V	A	V	F	S	A	V	D	G	V	Q	P	Q	S	E	T	V	W
				R	Q	A	D	K	Y	K	V	P	R	L	A	F	F	N	K	M	D	R	I	G	A	N	F	D	M	C	V	S	D
				I	K	E	K	L	G	S	N	P	V	P	I	Q	I	P	I	G	A	E	D	Q	F	E	G	V	V	D	L	I	E
				M	K	E	I	V	W	P	V	D	S	D	Q	G	Q	H	F	D	V	K	D	I	R	A	E	L	K	E	K	A	E
				E	T	R	Q	Y	M	L	E	S	I	V	E	T	D	D	A	L	M	E	K	F	F	G	G	E	E	I	T	K	E
				E	I	I	K	G	L	R	K	A	T	I	D	N	T	I	V	P	V	V	C	G	T	A	F	K	N	K	G	I	Q
				A	L	L	D	A	I	V	N	Y	M	P	A	P	T	D	V	A	M	V	E	G	R	D	P	K	N	P	D	V	L
				I	D	R	E	M	S	D	D	A	P	F	A	A	L	A	F	K	V	M	T	D	P	F	V	G	R	L	T	F	F
				R	V	Y	S	G	F	V	E	K	G	A	T	V	L	N	S	T	K	G	K	K	E	R	M	G	R	I	L	Q	M
				H	A	N	N	R	E	E	I	E	H	V	Y	C	G	D	I	A	A	A	V	G	L	K	D	T	A	T	G	D	T
				L	C	A	E	N	A	P	I	V	L	E	Q	M	E	F	P	E	P	V	I	S	V	A	V	E	P	K	T	K	N
				D	Q	E	K	M	G	I	A	L	S	K	L	A	E	E	D	P	T	F	K	V	R	T	D	E	E	T	G	Q	T
				I	I	S	G	M	G	E	L	H	L	E	I	I	V	D	R	M	K	R	E	F	K	V	E	S	N	V	G	K	P
				Q	V	A	Y	R	E	T	I	T	Q	S	C	D	Q	E	V	K	Y	A	K	Q	S	G	G	R	G	Q	Y	G	H
				V	K	I	I	L	E	P	N	P	G	K	E	F	E	F	V	N	K	I	T	G	G	V	I	P	R	E	Y	I	P
				A	V	E	K	G	C	K	E	A	L	E	S	G	V	I	A	G	Y	P	L	V	D	V	K	V	T	L	Y	D	G
				S	Y	H	E	V	D	S	S	E	M	A	F	K	I	A	G	S	M	A	L	K	Q	A	A	T	K	A	K	P	V
				I	L	E	P	V	F	K	V	E	V	T	T	P	E	E	Y	M	G	D	I	I	G	D	L	N	S	R	R	G	M
				V	S	G	M	I	D	R	N	G	A	K	I	I	T	A	K	V	P	L	S	E	M	F	G	Y	A	T	D	L	R
				S	K	S	Q	G	R	A	T	Y	S	W	E	F	S	E	Y	L	Q	V	P	A	S	I	Q	K	Q	I	Q	E	E
				R	G	K																											
				1				5					10					15					20					25					30
51	22.10%	99.0%	>sp|Q8RGC8|FBPC_FUSNN Fe(3+) ions import ATP-binding protein FbpC OS=Fusobacterium nucleatum subsp. nucleatum (strain ATCC 25586 / CIP 101130 / JCM 8532 / LMG 13131) OX=190304 GN=fbpC PE=3 SV=1																														
				M	S	V	N	I	K	I	E	N	A	Q	K	R	Y	G	D	N	I	I	I	E	N	L	S	L	D	I	K	Q	G
				E	F	F	T	L	L	G	P	S	G	C	G	K	T	T	L	L	R	M	I	A	G	F	N	S	I	E	K	G	N
				F	Y	F	N	E	K	R	I	N	D	L	D	P	A	K	R	N	I	G	M	V	F	Q	N	Y	A	I	F	P	H
				L	T	V	E	Q	N	V	E	F	G	L	K	N	R	K	V	S	K	E	E	M	K	I	E	I	D	K	F	L	K
				L	M	Q	I	D	E	Y	K	D	R	M	P	E	R	L	S	G	G	Q	Q	Q	R	V	A	L	A	R	A	L	V
				I	K	P	D	V	L	L	M	D	E	P	L	S	N	L	D	A	K	L	R	V	E	M	R	T	A	I	K	E	I
				Q	N	S	I	G	I	T	T	V	Y	V	T	H	D	Q	E	E	A	M	A	V	S	D	R	I	A	V	M	K	D
				G	E	I	Q	H	L	G	Q	P	K	D	I	Y	Q	R	P	A	N	L	F	V	A	T	F	I	G	K	T	N	V
				L	K	G	N	L	N	G	S	I	L	K	V	A	G	K	Y	E	V	S	L	N	N	I	K	D	K	N	I	K	G
				N	V	V	I	S	I	R	P	E	E	F	V	I	D	E	N	Q	T	K	D	G	M	R	A	F	I	D	S	S	V
				F	L	G	L	N	T	H	Y	F	A	H	L	E	S	G	E	K	I	E	I	V	Q	E	S	K	I	D	S	I	I
				P	K	G	A	E	V	Y	L	K	V	K	Q	D	K	I	N	V	F	T	E	D	G	S	R	N	I	L	E	G	V
				N	N	D	A	I	G	V	A	Y	V	K																			
				1				5					10					15					20					25					30
52	22.93%	99.0%	>sp|Q8RIQ1|MURA_FUSNN UDP-N-acetylglucosamine 1-carboxyvinyltransferase OS=Fusobacterium nucleatum subsp. nucleatum (strain ATCC 25586 / CIP 101130 / JCM 8532 / LMG 13131) OX=190304 GN=murA PE=3 SV=1																														
				M	V	E	A	F	K	I	I	G	G	K	K	I	A	G	E	L	K	V	D	G	S	K	N	S	T	L	P	I	M
				I	A	T	L	V	E	K	G	T	Y	V	L	R	N	V	P	D	L	R	D	I	R	T	L	V	A	L	L	E	S
				L	G	L	E	V	E	K	L	D	A	N	S	Y	K	I	I	N	N	G	L	S	G	A	E	A	S	Y	D	L	V
				K	K	M	R	A	S	F	L	V	M	G	G	M	L	A	I	E	K	R	G	K	V	A	L	P	G	G	C	A	I
				G	A	R	P	V	D	L	H	L	K	G	F	E	A	L	G	A	K	I	N	I	E	H	G	Y	V	E	A	T	T
				E	N	G	L	I	G	G	N	I	V	L	D	F	P	S	V	G	A	T	E	N	I	I	M	A	A	V	K	A	K
				G	K	T	V	L	E	N	A	A	K	E	P	E	I	E	D	L	C	N	F	L	I	K	M	G	A	K	I	S	G
				V	G	T	S	R	L	E	I	D	G	V	D	K	L	T	A	C	E	Y	T	I	I	P	D	R	I	V	A	G	T
				Y	I	I	A	S	I	L	F	D	G	S	I	K	V	S	G	I	V	P	E	H	L	S	S	F	L	L	K	L	E
				E	M	G	T	K	F	K	I	E	G	D	K	L	E	V	L	T	K	L	S	D	L	K	P	A	K	V	T	T	M
				P	H	P	G	F	P	T	D	L	Q	S	P	M	M	T	L	M	C	L	V	N	G	T	S	E	I	K	E	T	I
				F	E	N	R	F	M	H	V	P	E	L	N	R	M	G	A	K	I	E	I	D	S	S	T	A	K	I	T	G	V
				E	N	F	S	S	A	E	V	M	A	S	D	L	R	A	G	A	S	L	I	L	A	A	L	K	A	N	G	E	S
				L	V	N	R	I	Y	H	V	D	R	G	Y	E	N	F	E	E	K	F	K	A	L	G	A	N	I	E	R	I	K
				T	E	A																											
				1				5					10					15					20					25					30
53	26.90%	99.0%	>tr|D5RAM6|D5RAM6_FUSN2 ABC transportersolute-binding protein OS=Fusobacterium nucleatum subsp. nucleatum (strain ATCC 23726 / VPI 4351) OX=525283 GN=potD PE=4 SV=1																														
				M	K	K	I	F	L	L	F	L	V	T	I	I	L	V	S	C	G	D	S	K	D	E	N	T	L	Y	V	Y	S
				W	A	D	Y	I	P	Q	F	V	Y	S	D	F	E	K	E	T	G	I	R	V	I	E	D	I	Y	S	S	N	E
				E	M	Y	T	K	I	K	A	G	G	E	G	Y	D	I	I	M	P	S	S	D	Y	Y	E	I	M	M	K	E	D
				M	L	A	K	L	D	K	S	Q	L	E	N	V	K	N	I	D	D	S	Y	M	A	K	L	R	E	F	D	P	E
				N	D	Y	G	V	P	Y	M	R	G	I	T	C	I	A	V	N	K	K	F	V	K	D	Y	P	R	D	Y	T	I
				Y	N	R	E	D	L	A	G	R	M	T	L	L	D	D	M	R	E	V	F	V	P	A	L	A	L	N	G	Y	K
				Q	D	A	D	S	T	E	A	M	E	K	A	K	S	T	I	L	N	W	K	K	N	I	A	K	F	D	A	E	S
				Y	G	K	G	F	A	N	G	D	F	W	V	V	Q	G	Y	P	D	N	I	Y	R	E	L	S	E	E	D	R	E
				N	V	D	F	I	V	P	P	G	E	Q	G	Y	S	S	I	D	S	F	V	V	L	K	D	S	K	H	L	E	N
				A	M	K	F	I	N	Y	I	H	R	P	D	V	Y	A	K	I	S	D	F	I	E	I	P	S	I	N	T	E	A
				D	K	L	I	T	K	K	P	L	Y	D	V	E	K	T	K	N	A	Q	L	L	I	D	I	G	D	K	L	N	I
				Q	N	K	Y	W	Q	E	I	L	I	A	N																		
				1				5					10					15					20					25					30
54	10.85%	99.0%	>sp|Q8RFC2|HUTH1_FUSNN Histidine ammonia-lyase 1 OS=Fusobacterium nucleatum subsp. nucleatum (strain ATCC 25586 / CIP 101130 / JCM 8532 / LMG 13131) OX=190304 GN=hutH1 PE=3 SV=1																														
				M	E	V	F	I	L	E	L	V	L	G	S	K	N	I	T	L	E	D	L	I	N	V	T	R	K	G	Y	K	V
				S	I	S	E	E	A	Y	E	K	I	D	K	A	R	A	L	V	D	K	Y	V	E	E	G	K	V	S	Y	G	I
				T	T	G	F	G	K	F	A	E	V	S	I	S	K	E	Q	T	G	Q	L	Q	K	N	I	V	M	S	H	S	C
				N	V	G	N	P	L	P	I	D	I	A	K	G	I	V	L	L	R	A	V	N	L	A	K	G	Y	S	G	A	R
				R	I	V	I	E	K	L	V	E	L	L	N	K	D	V	T	P	W	I	P	E	K	G	S	V	G	S	S	G	D
				L	S	P	L	A	H	M	S	L	V	L	I	G	L	G	K	A	Y	Y	K	G	E	L	L	E	A	K	D	A	L
				A	K	A	D	I	E	P	I	P	A	L	S	S	K	E	G	L	A	L	T	N	G	T	Q	A	L	T	S	T	G
				A	H	V	L	Y	D	A	I	N	L	S	K	H	L	D	I	A	A	S	L	T	M	E	G	L	H	G	I	I	D
				A	Y	D	P	R	I	G	E	V	R	G	H	L	G	Q	I	N	T	A	K	N	M	R	N	I	L	A	G	S	K
				N	V	T	K	Q	G	V	E	R	V	Q	D	S	Y	V	L	R	C	I	P	Q	I	H	G	A	S	K	D	T	L
				E	Y	V	K	Q	K	V	E	L	E	L	N	A	V	T	D	N	P	I	I	F	V	D	T	D	E	V	I	S	G
				G	N	F	H	G	Q	P	M	A	L	P	F	D	F	L	G	I	A	L	S	E	M	A	N	V	S	E	R	R	I
				E	K	M	V	N	P	A	I	N	N	G	L	P	A	F	L	V	E	K	G	G	L	N	S	G	F	M	I	V	Q
				Y	S	A	A	S	L	V	S	E	N	K	V	L	A	H	P	A	S	V	D	S	I	P	T	S	A	N	Q	E	D
				H	V	S	M	G	S	V	A	A	K	K	S	K	D	I	F	E	N	V	R	K	V	I	G	M	E	L	I	T	A
				C	Q	A	I	D	L	K	E	A	K	D	K	L	S	P	A	T	K	V	A	Y	D	E	V	R	K	I	I	S	Y
				V	S	E	D	R	P	M	Y	I	D	I	H	A	A	E	D	L	I	K	T	N	K	I	V	E	N	V	E	K	A
				I	G	K	L	E	F																								
				1				5					10					15					20					25					30
55	14.71%	99.0%	>tr|Q8RE65|Q8RE65_FUSNN C4-dicarboxylate-binding protein OS=Fusobacterium nucleatum subsp. nucleatum (strain ATCC 25586 / CIP 101130 / JCM 8532 / LMG 13131) OX=190304 GN=FN1258 PE=1 SV=1																														
				M	A	L	V	A	C	G	G	K	K	E	E	A	T	K	E	S	G	D	A	K	Q	E	A	R	V	I	K	V	T
				T	K	F	V	D	D	E	Q	T	A	K	S	L	V	K	V	V	E	A	I	N	Q	R	S	N	G	T	L	E	L
				Q	L	F	T	S	G	T	L	P	I	G	K	D	G	M	E	Q	V	A	N	G	S	D	W	I	L	V	D	G	V
				N	F	L	G	D	Y	V	P	D	Y	N	A	V	T	G	P	M	L	Y	Q	S	F	E	E	Y	L	R	M	V	K
				T	P	L	V	Q	D	L	N	A	Q	A	L	E	K	G	I	K	V	L	S	L	D	W	L	F	G	F	R	N	I
				E	A	K	K	P	I	K	T	P	E	D	M	K	G	L	K	L	R	V	P	T	S	Q	L	Y	T	F	T	I	E
				A	M	G	G	N	P	V	A	M	P	Y	P	D	T	Y	A	A	L	Q	Q	G	V	I	D	G	L	E	G	S	I
				L	S	F	Y	G	T	K	Q	Y	E	N	V	K	E	Y	S	L	T	R	H	L	L	G	V	S	A	V	C	I	S
				K	K	C	W	D	S	L	T	D	E	Q	R	T	I	I	Q	E	E	F	D	K	G	A	L	D	N	L	T	E	T
				E	K	L	E	D	E	Y	A	Q	K	L	K	D	N	G	V	T	F	H	E	V	D	A	E	A	F	N	K	A	V
				A	P	V	Y	D	K	F	P	K	W	T	P	G	I	Y	D	K	I	M	E	N	L	T	Q	I	R	E	D	I	K
				N	G	K																											
				1				5					10					15					20					25					30
56	14.52%	99.0%	>sp|Q8RHF4|FTHS_FUSNN Formate--tetrahydrofolate ligase OS=Fusobacterium nucleatum subsp. nucleatum (strain ATCC 25586 / CIP 101130 / JCM 8532 / LMG 13131) OX=190304 GN=fhs PE=3 SV=1																														
				M	T	D	I	Q	I	A	Q	A	A	K	K	E	N	I	V	E	I	A	K	K	L	G	L	T	E	D	D	I	E
				Q	Y	G	K	Y	K	A	K	V	N	L	D	V	L	Q	K	N	K	R	P	N	G	K	L	I	L	V	T	A	I
				T	P	T	P	A	G	E	G	K	S	T	V	T	I	G	L	T	Q	A	L	N	K	M	G	K	L	S	A	A	A
				I	R	E	P	S	L	G	P	V	F	G	M	K	G	G	A	A	G	G	G	Y	A	Q	V	V	P	M	E	D	I
				N	L	H	F	T	G	D	M	H	A	I	G	I	A	H	N	L	I	S	A	C	I	D	N	H	I	N	S	G	N
				A	L	G	I	D	V	T	K	I	T	W	K	R	V	V	D	M	N	D	R	A	L	R	N	I	V	I	G	L	G
				G	K	A	N	G	Y	P	R	Q	D	S	F	Q	I	T	V	G	S	E	I	M	A	I	L	C	L	S	N	S	I
				T	E	L	K	E	K	I	K	N	I	V	I	G	T	S	V	T	G	K	L	I	K	V	G	D	F	H	I	E	G
				A	V	A	A	L	L	K	D	A	I	K	P	N	L	V	Q	T	L	E	N	T	P	V	F	I	H	G	G	P	F
				A	N	I	A	H	G	C	N	S	I	L	A	T	K	M	A	L	K	L	T	D	Y	V	V	T	E	A	G	F	A
				A	D	L	G	A	E	K	F	I	D	I	K	C	R	L	G	G	L	K	P	D	C	A	V	I	V	A	T	V	R
				A	L	E	H	H	G	K	G	D	L	K	A	G	L	E	N	L	D	K	H	I	D	N	I	K	N	K	Y	K	L
				P	L	V	V	A	I	N	K	F	I	T	D	T	D	E	Q	I	N	M	I	E	K	F	C	N	E	R	G	A	E
				V	S	L	C	E	V	W	A	K	G	G	E	G	G	I	D	L	A	E	K	V	L	K	A	I	D	N	N	K	T
				E	F	D	Y	F	Y	D	I	N	L	T	I	K	E	K	I	E	K	I	C	K	E	I	Y	G	A	D	G	V	I
				F	A	P	A	T	K	K	V	F	D	V	I	E	A	E	G	L	N	K	L	P	V	C	M	S	K	T	Q	K	S
				I	S	D	N	P	A	L	L	G	K	P	T	G	F	K	V	T	I	N	D	L	R	L	A	V	G	A	G	F	V
				I	A	M	A	G	D	I	I	D	M	P	G	L	P	K	K	P	S	A	E	V	I	D	I	D	E	N	G	V	I
				S	G	L	F																										
				1				5					10					15					20					25					30
57	34.04%	99.0%	>tr|Q8R6D3|Q8R6D3_FUSNN Alkyl hydroperoxide reductase C OS=Fusobacterium nucleatum subsp. nucleatum (strain ATCC 25586 / CIP 101130 / JCM 8532 / LMG 13131) OX=190304 GN=FN1983 PE=3 SV=1																														

				M	S	L	I	G	R	K	V	P	E	F	K	A	T	A	F	K	K	G	E	K	D	F	V	T	V	T	D	K	D
				L	L	G	K	W	S	V	F	V	F	Y	P	A	D	F	T	F	V	C	P	T	E	L	E	D	L	Q	D	N	Y
				E	A	F	K	K	E	G	A	E	V	Y	S	V	S	C	D	T	A	F	V	H	K	A	W	A	D	H	S	E	R
				I	K	K	V	T	Y	P	M	V	A	D	P	T	G	F	L	A	R	A	F	E	V	M	I	E	E	E	G	L	A
				L	R	G	S	F	V	I	N	P	E	G	K	I	V	A	Y	E	V	H	D	N	G	I	G	R	E	A	K	E	L
				L	R	K	L	Q	G	A	K	F	V	A	E	H	G	E	V	C	P	A	K	W	Q	P	G	S	E	T	L	K	P
				S	L	D	L	I	G	E	L																						
				1				5					10					15					20					25					30
58	25.90%	99.0%	>sp|Q8R6D9|LPXD_FUSNN UDP-3-O-acylglucosamine N-acyltransferase OS=Fusobacterium nucleatum subsp. nucleatum (strain ATCC 25586 / CIP 101130 / JCM 8532 / LMG 13131) OX=190304 GN=lpxD PE=3 SV=1																														
				M	E	Y	R	V	T	D	I	I	T	L	L	N	A	E	Y	K	G	E	V	V	E	K	V	S	K	L	S	P	F
				F	H	S	D	E	K	S	L	T	F	A	A	D	E	K	F	L	K	N	L	S	Q	T	K	A	K	V	I	I	V
				P	D	I	D	L	P	L	I	E	G	K	G	Y	I	V	V	K	D	S	P	R	V	I	M	P	K	L	L	H	F
				F	S	R	N	L	K	K	I	E	K	M	R	E	D	S	A	K	I	G	E	N	V	D	I	A	P	N	V	Y	I
				G	H	D	V	V	I	G	N	N	V	K	I	F	P	N	V	T	I	G	E	G	S	I	I	G	D	G	T	V	I
				Y	S	N	V	S	I	R	E	F	V	E	I	G	K	N	C	V	I	Q	P	G	A	V	I	G	S	D	G	F	G
				F	V	K	V	N	G	N	N	T	K	I	D	Q	I	G	T	V	I	V	E	D	E	V	E	I	G	A	N	T	T
				I	D	R	G	A	I	G	D	T	I	I	K	K	Y	T	K	I	D	N	L	V	Q	I	A	H	N	D	I	I	G
				E	N	C	L	I	I	S	Q	V	G	I	A	G	S	T	T	I	G	N	N	V	T	L	A	G	Q	V	G	V	A
				G	H	L	E	I	G	D	N	T	M	I	G	A	Q	S	G	V	P	G	N	V	E	A	N	K	I	L	S	G	H
				P	L	V	D	H	R	E	D	M	K	I	R	V	A	M	K	K	L	P	E	L	L	K	R	V	K	A	L	E	E
				K	K																												
				1				5					10					15					20					25					30
59	1.79%	99.0%	>tr|A0A101K6J5|A0A101K6J5_FUSNC Uncharacterized protein OS=Fusobacterium nucleatum subsp. nucleatum OX=76856 GN=RO03_07960 PE=4 SV=1																														
				M	K	D	Y	N	K	V	E	S	C	L	K	S	F	L	K	N	N	K	R	L	S	Y	S	M	A	L	L	I	T
				F	L	I	N	G	G	F	S	Y	A	D	E	A	V	Q	V	P	L	R	T	E	I	K	T	R	I	E	K	E	Q
				E	N	I	S	Q	M	L	K	E	A	D	E	S	M	K	D	I	E	L	K	I	K	K	L	T	Q	R	G	E	F
				W	V	K	P	L	E	K	S	Y	Q	G	F	I	F	A	N	W	G	N	Y	S	K	N	K	N	K	T	E	S	N
				F	N	G	P	E	Y	S	A	S	Y	G	R	N	M	G	Y	G	Q	F	S	N	G	K	Y	Y	G	E	Y	G	I
				V	K	N	P	L	E	F	V	D	K	I	D	F	G	A	N	I	T	P	K	A	V	I	E	K	T	I	I	E	K
				T	V	I	K	K	D	I	T	A	P	S	V	T	P	P	T	V	E	V	G	E	I	T	L	T	A	P	E	E	V
				A	I	G	E	M	T	P	P	N	E	P	N	P	S	V	T	T	P	S	T	V	P	A	L	Q	G	I	T	V	A
				A	V	S	E	V	N	V	T	P	S	T	P	E	V	A	A	A	P	T	I	N	A	P	G	V	Q	P	P	A	T
				P	A	G	F	T	P	R	L	I	T	P	P	E	A	P	E	D	I	V	I	T	P	P	G	E	V	Q	A	N	L
				T	G	S	G	A	N	P	T	T	Y	Y	Y	W	W	N	G	N	K	G	A	I	S	Q	I	S	L	T	A	G	T
				F	N	I	E	S	G	N	T	I	N	V	N	G	Y	Q	A	K	A	F	T	G	T	T	P	Q	G	T	K	P	S
				D	G	D	Y	V	I	N	Q	Q	F	F	H	T	L	L	N	V	A	Y	S	E	Y	S	N	G	V	V	I	N	N
				N	R	A	G	F	T	L	I	N	L	E	T	E	G	Q	V	S	G	N	L	A	D	R	V	S	D	A	S	I	T
				A	D	K	K	T	T	L	K	G	Y	Q	T	Y	S	E	I	K	G	Q	N	N	T	E	L	L	F	I	N	K	G
				T	V	N	L	N	A	D	N	T	T	Y	L	F	T	T	S	H	N	D	G	N	L	R	T	N	Y	L	D	N	E
				G	T	I	S	A	K	G	K	N	S	I	I	I	K	H	S	P	D	T	N	Q	A	K	A	W	I	Y	S	N	N
				G	I	M	K	A	E	G	K	G	S	I	V	Y	G	A	A	Y	S	Y	L	G	S	G	R	A	A	F	V	N	D
				G	T	I	E	V	S	G	E	E	A	I	G	V	I	L	P	R	K	T	G	N	S	E	L	A	N	G	H	L	V
				W	L	K	K	P	I	S	L	K	G	K	K	S	M	G	F	V	A	Q	N	T	N	I	S	N	E	K	N	L	V
				K	F	N	I	D	A	E	K	A	I	G	I	L	Q	D	V	E	G	A	G	T	A	K	T	A	G	K	I	V	I
				E	G	N	S	S	G	S	M	G	I	Y	A	K	Q	G	T	L	E	L	I	K	P	H	A	D	S	G	E	T	E
				S	S	I	E	L	K	A	G	S	N	N	I	G	I	F	A	K	G	A	S	T	V	N	F	N	G	I	T	K	I
				T	G	G	E	R	Q	K	I	A	L	A	T	E	G	S	T	I	N	L	K	N	T	V	T	A	G	D	K	I	T
				S	N	F	V	K	D	A	V	P	L	Y	A	T	G	T	G	S	K	I	N	V	V	T	P	D	S	L	K	F	Y
				L	G	G	N	S	T	A	A	Y	A	K	D	G	G	I	I	N	M	N	R	T	N	L	P	A	D	P	S	I	Y
				I	K	G	E	N	G	K	G	I	G	L	F	A	K	D	G	G	V	I	N	A	Q	K	H	Y	I	K	V	E	N
				G	S	V	A	L	S	S	V	G	A	N	A	T	N	K	S	N	I	D	F	T	G	G	K	L	E	Y	T	G	N
				G	Y	A	V	Y	S	D	G	T	G	T	V	N	L	S	D	A	E	L	N	L	Y	G	S	S	T	A	F	D	V
				D	F	N	A	T	T	L	P	T	I	L	N	A	N	T	R	I	H	A	N	S	D	D	V	I	A	F	N	L	K
				N	A	S	G	L	T	T	V	G	G	I	E	T	S	I	K	S	K	I	E	T	K	L	G	L	G	S	G	S	L
				N	N	L	F	T	G	S	A	S	T	K	Y	K	V	A	A	V	D	G	G	E	I	T	V	G	N	L	D	K	S
				G	T	K	D	D	T	D	Q	A	K	R	D	G	Y	Q	Y	F	N	R	F	L	A	Q	R	L	K	A	T	A	N
				G	S	T	I	K	A	V	L	N	S	N	F	A	N	S	N	F	N	G	Q	V	V	G	F	E	M	N	S	S	K
				N	A	T	S	V	N	E	T	A	I	N	L	V	D	S	K	I	I	A	D	R	T	D	A	G	T	G	A	I	G
				A	F	I	N	Y	G	E	V	N	I	D	A	T	S	K	I	E	V	E	K	E	N	N	V	V	N	K	Q	A	V
				G	V	Y	A	V	N	G	S	K	V	D	N	K	G	T	I	D	V	G	G	D	Q	S	V	G	I	L	G	M	A
				Y	R	E	G	S	S	N	N	P	I	V	N	E	F	G	G	K	S	G	E	G	T	V	N	I	T	N	E	K	D
				I	K	M	S	G	K	D	A	I	G	I	Y	A	M	N	N	N	P	D	K	T	V	T	S	H	L	V	I	N	K
				G	T	V	E	V	G	D	S	A	E	K	T	A	V	G	I	Y	A	K	G	V	D	V	K	P	E	S	G	K	I
				K	I	G	K	K	A	V	G	I	Y	A	E	D	S	N	V	G	E	V	N	K	D	L	G	T	I	D	F	N	G
				D	D	G	V	G	I	Y	L	K	G	S	G	S	N	L	L	G	N	K	V	T	L	T	Q	S	K	D	S	K	N
				K	V	G	I	L	A	D	R	G	T	S	S	I	I	K	T	E	V	A	V	G	A	L	N	N	V	I	A	Y	Y
				S	K	G	N	H	E	F	N	V	Q	S	N	V	T	L	N	E	N	S	I	G	I	S	G	E	G	D	L	Q	Y
				G	D	G	T	N	P	Y	I	M	K	L	G	K	G	S	T	G	L	F	G	T	K	N	I	V	L	K	D	K	T
				N	I	E	L	N	G	E	N	S	V	G	A	Y	A	S	G	A	S	G	V	I	T	S	E	G	K	I	K	F	L
				K	E	K	S	I	G	L	Y	G	A	N	G	A	T	V	K	D	K	T	T	M	D	F	S	N	A	N	A	K	N
				N	I	G	V	Y	L	A	G	A	K	W	G	I	D	R	A	L	T	F	D	S	A	H	E	K	G	N	I	Y	L
				F	A	Q	G	G	S	T	A	T	L	K	N	G	F	D	I	T	P	L	T	A	P	T	G	N	N	R	T	I	G
				M	Y	L	D	T	A	V	K	G	G	A	T	T	A	D	N	T	V	D	M	S	D	G	N	A	K	I	S	V	T
				K	K	A	I	G	I	Y	A	K	N	V	D	N	S	K	N	N	I	I	N	K	I	K	V	L	S	D	G	Q	G
				T	V	G	V	F	T	D	G	N	L	K	L	S	G	N	G	G	L	I	E	A	Q	N	A	G	I	G	L	Y	G
				N	K	G	T	V	T	V	E	G	T	H	K	V	E	V	T	S	A	G	T	G	M	Y	L	T	K	G	S	H	L
				S	G	G	K	L	E	L	E	N	K	T	A	G	T	S	A	A	G	I	Y	Y	E	G	T	N	N	E	V	D	H
				N	T	D	I	E	V	T	A	G	E	N	L	L	A	I	Y	A	N	G	L	K	L	N	N	N	K	E	I	L	I
				K	K	G	K	N	N	V	A	A	Y	I	T	G	N	S	T	F	K	N	K	G	K	I	Q	L	G	H	P	T	Q
				N	D	F	E	S	G	I	G	I	Y	V	V	D	G	E	A	I	N	E	S	G	K	T	I	D	I	Y	D	F	E
				N	T	A	S	G	G	S	L	S	V	G	M	L	A	K	A	G	A	G	K	T	A	K	V	T	N	K	G	T	I
				N	A	N	G	E	V	I	G	M	V	V	E	D	N	S	E	G	L	N	D	T	G	A	E	I	V	A	K	D	K
				E	D	I	N	A	K	A	I	G	A	Y	V	K	G	A	N	A	K	F	E	N	K	G	K	I	S	A	E	N	I
				A	L	A	L	Q	G	T	G	A	N	K	I	L	N	S	G	T	L	N	L	T	K	T	G	A	I	G	V	Y	A
				K	D	S	V	V	D	F	N	I	A	P	T	V	A	G	A	N	K	T	V	A	L	Y	A	S	G	T	T	K	I
				K	S	Q	I	T	S	A	S	G	K	A	H	I	G	V	Y	A	E	G	D	A	E	F	L	S	G	S	K	V	T
				V	G	N	G	D	G	N	D	Y	G	I	G	V	Y	T	K	S	G	Y	N	K	T	V	N	T	D	I	Q	L	G
				G	E	K	T	I	G	F	Y	L	G	A	T	G	G	T	G	S	T	V	T	H	T	G	T	I	D	V	G	S	G
				I	G	T	Y	I	P	E	H	S	K	F	I	A	Q	N	T	T	F	N	V	G	D	N	G	T	A	V	Y	L	K
				G	G	E	V	D	L	G	K	T	G	T	A	N	I	N	F	N	G	T	N	G	R	A	I	Y	Q	D	G	G	T
				I	T	T	G	T	G	L	H	I	Q	G	S	G	S	F	L	T	L	K	N	A	N	S	S	I	N	S	L	V	E
				V	G	A	S	G	I	G	I	N	G	I	Y	D	M	A	G	K	D	Y	T	L	T	L	E	S	P	N	G	H	I
				K	L	G	G	N	K	A	T	G	I	A	A	V	A	K	S	T	V	G	P	N	K	V	N	V	I	N	K	G	T
				I	E	T	T	S	G	E	K	T	T	G	I	Y	G	K	G	A	N	I	E	N	A	T	G	A	K	I	N	I	G
				A	K	G	V	G	I	Y	T	T	N	D	N	S	L	E	N	T	T	L	N	N	A	G	E	I	N	L	T	G	D
				E	A	T	G	L	V	A	V	K	A	K	T	N	Q	D	F	I	V	G	K	I	S	G	T	K	D	K	L	V	G
				A	Y	F	K	D	S	Q	A	V	T	K	V	K	D	F	N	I	S	L	G	T	N	A	K	G	L	V	F	D	G
				G	K	D	F	T	V	T	S	S	S	T	N	K	V	K	I	G	N	T	T	G	N	S	R	G	I	G	I	A	A
				L	G	V	N	G	N	I	S	K	T	D	V	V	V	G	K	G	S	L	G	L	Y	A	K	D	K	K	L	T	F
				D	L	A	S	G	K	L	E	S	S	D	A	N	R	S	S	I	L	A	Y	A	D	G	N	T	S	E	V	A	L
				N	G	G	G	T	L	K	V	G	A	N	G	I	A	L	G	T	K	G	G	K	V	S	A	N	A	T	T	T	V
				E	V	D	G	V	K	G	L	G	A	Y	V	E	N	G	G	S	I	D	N	N	F	D	I	K	V	K	S	A	E
				G	I	G	M	Y	A	K	G	G	A	L	T	S	V	A	K	V	S	E	I	K	G	N	K	S	I	G	Y	V	F
				E	N	I	T	S	A	I	T	M	P	N	S	V	Q	L	T	D	I	N	A	T	G	Q	V	G	V	V	A	Q	G
				T	G	N	G	L	T	V	A	G	V	S	V	V	G	S	G	N	T	G	V	Y	S	S	T	G	K	A	V	I	N
				N	G	T	L	T	V	G	D	S	T	G	K	S	S	I	G	I	Y	S	K	G	G	A	V	T	S	T	G	S	A
				T	I	G	K	N	S	I	A	I	Y	G	K	D	T	A	A	T	L	N	G	N	L	T	I	G	E	K	G	I	G
				L	Y	V	D	N	T	A	T	S	K	G	D	T	A	V	N	G	N	I	T	V	G	A	N	G	A	I	G	I	Q
				T	T	N	S	K	V	N	L	T	G	D	L	S	V	A	S	G	D	S	K	G	I	F	S	M	G	A	G	N	V
				E	T	T	G	N	I	T	V	G	S	N	S	V	G	I	Y	K	N	G	S	G	E	V	K	T	A	A	G	K	T
				L	T	V	A	D	S	A	Y	G	I	F	S	K	G	A	K	L	I	N	N	M	N	V	T	V	G	V	D	A	I
				G	A	Y	V	D	G	N	D	L	T	S	T	G	T	V	T	V	G	D	K	G	V	G	L	L	V	K	G	T	G
				K	T	L	T	S	T	G	N	I	T	V	G	S	N	N	S	V	G	L	Y	A	G	D	N	A	N	I	A	Q	S
				G	N	I	T	V	A	N	N	N	G	I	G	V	Y	S	K	G	N	G	N	V	S	T	I	G	A	I	T	V	G
				K	D	S	I	G	V	Y	K	D	G	K	G	T	M	N	I	N	A	S	S	P	I	Q	T	M	T	I	A	E	K
				G	Y	G	L	Y	Y	K	G	N	S	R	A	D	S	I	I	N	S	N	M	N	M	T	L	G	K	E	A	V	G
				I	Y	A	K	N	T	T	V	N	H	V	G	D	I	T	V	G	E	T	N	I	G	S	S	G	F	T	T	P	S
				D	N	K	N	S	I	G	I	F	G	D	N	S	N	I	N	F	K	G	N	M	L	V	D	K	P	L	S	V	G
				I	Y	G	S	N	G	G	S	I	T	V	K	S	G	S	T	I	T	V	K	N	G	A	T	G	I	M	T	G	S
				K	V	E	S	I	T	L	E	S	G	S	T	L	N	V	D	G	K	V	D	T	S	V	Y	T	N	A	T	K	S
				N	V	S	F	G	I	A	A	Y	S	G	A	I	N	N	Q	G	T	I	N	V	K	N	G	A	T	G	I	Y	L
				A	G	T	A	S	L	V	N	Q	G	T	I	T	V	D	A	I	S	K	Q	I	G	R	P	D	T	K	A	S	A
				E	L	G	G	I	K	V	T	D	K	G	E	V	T	I	N	N	K	V	I	N	G	G	T	V	N	I	K	G	D
				L	N	M	A	G	M	G	L	D	V	S	T	G	K	T	I	V	D	A	R	S	I	S	G	V	A	E	V	L	P
				N	F	S	K	G	N	S	E	Q	K	V	T	I	K	D	V	F	R	T	G	A	V	G	A	F	S	G	D	V	K
				S	K	S	V	S	W	I	A	K	I	S	K	E	P	G	S	S	T	T	T	K	D	I	T	M	V	R	I	P	Y
				N	S	L	I	S	G	E	K	Y	K	N	L	A	K	G	L	E	D	I	R	S	K	I	G	K	D	S	S	S	P
				I	F	K	S	L	D	N	I	S	S	H	R	D	F	A	R	A	I	A	N	I	R	G	D	I	Y	S	N	V	Q
				E	R	M	K	T	V	E	N	A	F	D	K	S	Y	N	E	L	L	S	S	Y	N	K	T	R	N	V	N	K	F
				G	I	I	Y	T	G	G	E	H	K	D	S	T	L	G	V	S	G	Y	K	Y	K	S	T	G	V	L	Y	L	N
				D	R	E	A	F	T	Y	G	G	K	Y	G	W	S	A	G	I	V	G	S	N	F	E	F	N	G	D	T	N	K
				G	S	K	E	R	V	V	S	G	K	L	G	L	H	Y	Q	A	P	L	N	K	E	D	D	N	A	K	L	K	W
				L	T	R	G	E	V	T	V	N	N	H	R	T	N	R	Y	S	Q	V	G	K	D	T	Y	Q	N	K	A	S	F
				Y	S	T	E	L	S	W	K	N	I	I	S	Y	D	Y	D	I	N	T	N	W	M	V	K	P	Y	T	G	I	D
				M	S	Y	G	H	I	F	N	I	K	E	K	N	E	G	L	P	L	E	V	K	G	K	D	Y	F	V	I	T	P
				N	V	G	V	E	T	K	Y	V	L	P	G	A	T	H	Q	V	F	A	K	A	D	T	E	F	S	Y	D	V
				A	K	L	Y	H	G	V	N	Q	A	K	M	K	N	A	S	S	G	Y	Y	D	L	S	K	P	E	R	R	R	A
				R	V	A	V	G	A	E	L	G	L	E	K	E	N	A	Y	G	I	T	F	R	A	E	Y	Q	G	Y	K	K	S
				Q	L	N	Y	G	V	R	L	N	Y	K	F																		

			1 5 10 15 20 25 30
60	23.88%	99.0%	>tr|D5RA84|D5RA84_FUSN2 ABC transporter, ATP-binding protein OS=Fusobacterium nucleatum subsp. nucleatum (strain ATCC 23726 / VPI 4351) OX=525283 GN=HMPREF0397_0119 PE=3 SV=1
			M A S V T I T G V T K S F G N V T V L Q E F N Q K F E D G E
			F I T L L G P S G C G K T T M L R L I A G F E K P S S G E I
			Y I G D K L V S S E K E F L P P E K R G I G M V F Q S Y A V
			W P H M N V F D N I A Y P L K I Q K I N K N E I E E R V K Q
			V L K I V H L E Q Y K D R F P S E L S G G Q Q Q R V A L G R
			A L V A Q P E I L L L D E P L S N L D A K L R E E M R Y E I
			K E I T K K L K I T V I Y V T H D Q I E A M T M S D R I V L
			I N K G E V Q Q V A P P Q E I Y S K P K N M F V A N F V G K
			V D F I T G K V E G S K I L L D N S N N Q I L S N T S S F K
			G K V V V A I R P E N A I L S D D G E I T G K V Y S K F Y L
			G D C N D L R V E I G N G N I L R I I A R A S T Y N T L N E
			G D E V K I K I L D Y F V F E D D G K D Q I K I M T
			1 5 10 15 20 25 30
61	16.50%	99.0%	>tr|D5RC10|D5RC10_FUSN2 Uncharacterized protein OS=Fusobacterium nucleatum subsp. nucleatum (strain ATCC 23726 / VPI 4351) OX=525283 GN=HMPREF0397_0745 PE=4 SV=1
			M N K K K I L L F M L M I A M S F N I N A A Q T A S F A E T
			I N N E N I E V I A T Y D N E L P Q E I K K I Y K P K H T G
			E G V S Y F D Y I F I K A R V S N L R E K P D P D S Q I V G
			K Y T Y D S K L K L L E K I K Y Q G N L W Y L A Q D Q N G V
			K G Y I A A S Q T E K R N F R F Q M A L D K I H D L E D F I
			N K S L N E G A T L M S V N T Y A P N P S N I N P K R Q K D
			K Y G T S L D Q N L L G I S Q K G E Q I I I P D R S V V K I
			I E N R G D K A L V K A L S I P E T L E V S K A K L S T F P
			S I K K G F R K V I A I D I E N Q N F M V F E K S K Q T N E
			W E L I S Y V Y T K T G I D S E L G Y E T P K G F F T V P V
			V K Y V M P Y T D E T G Q K A G T A K F A I R F C G G G Y L
			H G T P I N V Q E E V N K E F F L R Q K E F T L G T Y T G T
			R K C V R T S E G H A K F L F D W L V G S A N K D S N D Q R
			L S E D A Y F I V F
			1 5 10 15 20 25 30
62	23.46%	99.0%	>sp|Q8RFZ9|RLMN_FUSNN Probable dual-specificity RNA methyltransferase RlmN OS=Fusobacterium nucleatum subsp. nucleatum (strain ATCC 25586 / CIP 101130 / JCM 8532 / LMG 13131) OX=190304 GN=rlmN PE=3 SV=1
			M K N E K I N I L N L T Q E E L T E L L V S L G L K K F Y G
			K E V F I W L H K K I T R S F D E M T N L S L K D R E I L K
			E K T Y I P F F N L L K Y Q V S K I D K T E K F L F E L E D
			G G T I E T V L L R H K D S K N K E I R N T L C V S S Q V G
			C P V K C S F C A T G Q S G Y M R N L S V S E I L N Q I Y T
			V E R R L R K K G E N L N N L V F M G M G E P L L N I D N L
			S K A L S I I S N E N G I N I S K R K I T I S T S G V V S G
			I E K I L L D K I P I E L A I S L H S A I N E K R D K I I P
			I N K N F P L E D L S A V L I E Y Q K Q T K R R I T F E Y I
			L I D N F N I S E T D A N A L A D F I H Q F D H V V N L I P
			Y N E V E G A E H T R P S V K K I N K F Y I Y L K N V R K V
			N V T L R Q E K G S D I D G A C G Q L R Q R N K K G D N
			1 5 10 15 20 25 30
63	13.09%	99.0%	>tr|Q8RG28|Q8RG28_FUSNN Alanine racemase OS=Fusobacterium nucleatum subsp. nucleatum (strain ATCC 25586 / CIP 101130 / JCM 8532 / LMG 13131) OX=190304 GN=FN0491 PE=3 SV=1
			M N T S F Y V S L D K N A L Y H N I E Y L R E Y K Q K E L L
			P V I K A N A Y G H N Y L L I A K A L Y D F N I K T W A A A
			R F S E A I T I T E Y M M N N F S I N D F K I L V F E S L D
			D D Y S F L E K Y P Q I C P T I N S I K D L K N A L A N N I
			P I D R L S L K I D F G F G R N G I K T E E V D E L K N L I
			K F N S L K F L G I F S H L F S S T Y T D G L E V I R K F T
			E V V N K L G K D N F E M V H L Q N A A G I Y N Y N V E V V
			T H I R T G M L T Y G L Q E A G F Y D H D L K P V F T G L I
			G Y V D S V R Y V N E L D Y V A Y E D L T S I S P K T K K I
			A K I K I G Y G D G F P K A N N K T T C L I K K K E Y V I S
			Q V T M D N T F I E V D D R V N V G D K V H L Y H R P N E M
			K T R T G L S M L E A L I A L S P L R V K R I F E G E E N
			1 5 10 15 20 25 30
64	23.77%	99.0%	>tr|Q8RHP5|Q8RHP5_FUSNN Tetratricopeptide repeat family protein OS=Fusobacterium nucleatum subsp. nucleatum (strain ATCC 25586 / CIP 101130 / JCM 8532 / LMG 13131) OX=190304 GN=FN1965 PE=4 SV=1
			M D E K F W E K I N T F G E N G E F D K I V K E I K K L P E
			D K L D I E I I N V L G R A Y M N L G D Y E N A L D T Y L S
			Y I G K A K E D V T N V D I W L Y S E C G W L C N E V G D Y
			E Q G L K Y L L E A E K L G R D D E W L N T E M G Q C L G R
			L E R A E E G L E R L K K S L K L I E T E A P E N I N E K I
			F I N S E I G Y L Y G F L E N S E E A L K Y F H I A K D L G
			R K D D W I Y M H L W F N L E R S K G K E E A L K Y F E N E
			A K T D D K N A I V W A S L G Q I Y M N F F R N Y E E A E K
			V F K K A F G L S G D G L Y L Y N R G M V L R I L G R Y K E
			A V E V L L Q S R K I S V Q E G D V T D G E D L D L V R C Y
			I A L K D K E N A K K Y L E F A K E G M E N I P E E H V D E
			F E D A L K E L E D L I A K I
			1 5 10 15 20 25 30
65	55.56%	99.0%	>tr|Q8REM0|Q8REM0_FUSNN Neutrophil-activating protein A OS=Fusobacterium nucleatum subsp. nucleatum (strain ATCC 25586 / CIP 101130 / JCM 8532 / LMG 13131) OX=190304 GN=FN1079 PE=3 SV=1
			M K N K E N L N K Y L S N L G I L I T K T H N L H W N V V G
			A R F K A I H E Y T E S L Y D Y Y F E K F D E V A E A F K M
			K G E F P L V K V A D Y L K H A T V K E L E A K D F T I P E
			V V T S I K E D I E L M L A D A R K I R E V A N E E D D F L
			V A N M M E D Q I E Y F V K Q L W F I S A M A K
			1 5 10 15 20 25 30
66	15.84%	99.0%	>tr|A0A0M1VWV6|A0A0M1VWV6_FUSNV (R)-2-hydroxyglutaryl-CoA dehydratase subunit alpha OS=Fusobacterium nucleatum subsp. vincentii 4_1_13 OX=469606 GN=FSCG_01612 PE=4 SV=1
			M A G K M E K L P N K K P R P I E G H K P A A A I L R G V V
			D K V Y A N A W E A K K R G E L V G W S S S K F P I E L A K
			A F D L N V V Y P E N H A A S T A A K K D G L R L C Q A A E
			D M G Y D N D I C G Y A R I S L A Y A A G E P T D S R R M P
			Q P D F L L C C N N I C N M M T K W Y E N I A R I H N I P L
			I M I D I P F S N T V D T P E E K V D Y L I G Q F D H A I K
			Q L E E L T G K K F D E K K F E D A C A R A N R T A A A W L
			K S C K Y M G Y K P S P L S G F D L F N H M A D I V A A R C
			D E E A A I G F E L L A D E F E Q S I K E G T S T W E Y P E
			E H R I L F E G I P C W P G L K P L F E P L K D N G V N V T
			A V V Y A P A F G F R Y N N V R E M A A A Y C K A P C S V C
			I E T G V E W R E T M A K E N G I S G A L V N Y N R S C K P
			W S G A M P E I E R R W K E D L G I P V V H F D G D Q A D E
			R N F S T E Q Y N T R V Q G L V E I M Q E R K E E K L A K G
			E E V Y T N F E N T K E T D W S K E T I K H
			1 5 10 15 20 25 30
67	11.92%	99.0%	>tr|R9RCC7|R9RCC7_FUSNU Uncharacterized protein OS=Fusobacterium nucleatum subsp. animalis 4_8 OX=469607 GN=HMPREF0409_00425 PE=4 SV=1
			M E K L F I F N K K L L T K H N K K L Y I V L Y M K I Y N I
			K Q K I I I L L N F L G G Y L Y E K I G M L L G S I I L A S
			A L V A C G E K K E E A K T E A P A S E K L S I G L T A Y K
			F D D N F I A L F R K A F E A E A A A K A D T V E V T A I D
			S Q N S V A T E K E Q I E A V L E K G V K A F A I N L V D A
			S A A D G I I N L L K E K N V P V V F Y N R K P S D E A I A
			S Y D K L Y Y V G I D P N A Q G V A Q G E L I E K L W K E N
			P D L D L N K D G V I Q Y V M L T G E P G H P D A V A R T K
			Y S I Q T L N D H G I K T E E L H K D T A M W D T A T A K D
			K M D A W L S G P N G S K I E V V I C N N D G M A L G A I E
			S M K A A G K V L P T F G V D A L P E A L V K I E A G E M A
			G T V L N D A K G Q A S A T F N M V V N L A Q G K E P T E G
			T D L K L D N K V I L I P S I G I D K S N V A D F K
			1 5 10 15 20 25 30
68	18.67%	99.0%	>sp|Q8RGX8|PLSX_FUSNN Phosphate acyltransferase OS=Fusobacterium nucleatum subsp. nucleatum (strain ATCC 25586 / CIP 101130 / JCM 8532 / LMG 13131) OX=190304 GN=plsX PE=3 SV=1
			M K I A L D A M S G D F A P I S T V K G A I E A L E E I E G
			L Q V I L V G K E G I I K E E L K K Y K Y D T N R I E I K N
			A D E V I V M T D D P V K A V R E K K D S S M N V C I D L V
			K E K L A Q A S V S C G N T G A L L A S S Q L K L K R I K G
			V L R P A I A V L F P N K K D S G T L F L D L G A N S D S K
			P E F L N Q F A T M G S K Y M E I F S G K K N P K V A L L N
			I G E E E T K G N E L T R E T Y A L L K E N K D I D F Y G N
			I E S T K I M E G D V D V V V T D G Y T G N I L L K T S E G
			I G K F I F H I V K E S I M E S W I S K L G A L L V K G A M
			K K V K K K T E A S E Y G G A I F L G L S E L S L K A H G N
			S D S R A I K N A L K V A S K F I E L N F I E E L R K T M E
			V E
			1 5 10 15 20 25 30
69	14.11%	99.0%	>tr|Q8RDT9|Q8RDT9_FUSNN Threonine dehydratase OS=Fusobacterium nucleatum subsp. nucleatum (strain ATCC 25586 / CIP 101130 / JCM 8532 / LMG 13131) OX=190304 GN=FN1411 PE=4 SV=1
			M A K L E A F I K A K E K L S K V L L E T Q L I Y S P I F S
			K E S G N K V F I K P E N L Q K T G S F K I R G A Y N K I S
			N L T D A E K K R G V I A S S A G N H A Q G V A Y G A K E S

				G	I	K	A	V	I	V	M	P	K	S	T	P	L	I	K	V	E	S	T	K	Q	Y	G	A	E	V	I	L	H
				G	D	V	Y	D	D	A	F	K	K	A	K	E	L	E	E	K	E	G	Y	V	F	V	H	P	F	N	D	E	D
				V	L	D	G	Q	G	T	I	A	L	E	I	L	E	E	L	P	E	T	D	I	I	L	V	P	I	G	G	G	G
				L	I	S	G	I	A	C	A	A	K	I	L	K	P	E	I	K	I	I	G	V	E	P	E	G	A	A	S	A	Y
				E	A	I	K	E	N	K	V	V	E	L	K	E	A	N	T	I	A	D	G	T	A	V	K	K	I	G	D	L	N
				F	E	Y	I	K	K	Y	V	D	E	I	I	T	V	S	D	Y	E	L	M	E	A	F	L	L	L	V	E	K	H
				K	I	I	A	E	N	S	G	I	L	S	I	A	A	T	K	K	L	K	E	K	N	K	K	V	V	S	V	I	S
				G	G	N	I	D	V	L	M	I	S	S	M	I	N	K	G	L	I	R	R	D	R	I	F	N	F	T	V	S	I
				P	D	K	P	G	E	L	A	K	V	V	D	L	I	A	E	Q	G	A	N	V	I	K	L	E	H	N	Q	F	K
				N	L	S	R	F	K	D	I	E	L	Q	I	T	V	E	T	N	G	S	E	H	V	Q	N	L	T	Q	A	F	E
				E	K	G	Y	E	I	V	R	I	K	S	K	I	N																
				1				5					10					15					20					25					30
70	26.15%	99.0%	>sp|Q8RE43|RS4_FUSNN 30S ribosomal protein S4 OS=Fusobacterium nucleatum subsp. nucleatum (strain ATCC 25586 / CIP 101130 / JCM 8532 / LMG 13131) OX=190304 GN=rpsD PE=3 SV=1																														
				M	A	R	N	R	Q	P	V	L	K	K	C	R	A	L	G	I	D	P	V	I	L	G	V	K	K	S	S	N	R
				Q	I	R	P	N	A	N	K	K	P	T	E	Y	A	T	Q	L	R	E	K	Q	K	A	K	F	I	Y	N	V	M
				E	K	Q	F	R	K	I	Y	E	E	A	A	R	K	L	G	V	T	G	L	T	L	I	E	Y	L	E	R	R	L
				E	N	V	V	Y	R	L	G	F	A	K	T	R	R	Q	A	R	Q	I	V	S	H	G	H	I	A	V	N	G	R
				R	V	N	I	A	S	F	R	V	K	V	G	D	I	V	S	V	I	E	N	S	K	N	V	E	L	I	K	L	A
				V	E	D	A	T	P	P	A	W	L	E	L	D	R	A	A	F	S	G	K	V	L	Q	N	P	T	K	D	D	L
				D	F	D	L	N	E	S	L	I	V	E	F	Y	S	R															
				1				5					10					15					20					25					30
71	20.00%	99.0%	>tr|D5REA7|D5REA7_FUSN2 4-phosphoerythronate dehydrogenase OS=Fusobacterium nucleatum subsp. nucleatum (strain ATCC 23726 / VPI 4351) OX=525283 GN=pdxB PE=3 SV=1																														
				M	Q	K	T	K	I	I	F	F	D	I	K	D	Y	D	K	E	F	F	K	K	Y	G	A	D	Y	N	F	E	M
				T	F	L	K	V	R	L	T	E	E	T	A	N	L	T	K	G	Y	D	V	V	C	G	F	A	N	D	N	I	N
				K	E	T	I	D	I	M	A	E	N	G	I	K	L	L	A	M	R	C	A	G	F	N	N	V	S	L	K	D	V
				N	E	K	F	K	V	V	R	V	P	A	Y	S	P	H	A	I	A	E	Y	T	V	G	L	I	L	A	V	N	R
				K	I	N	K	A	Y	V	R	T	R	E	G	N	F	S	I	N	G	L	M	G	I	D	L	Y	E	K	T	A	G
				I	I	G	T	G	K	I	G	Q	I	L	I	K	I	L	R	G	F	D	M	K	V	I	A	Y	D	L	F	P	N
				Q	K	V	A	D	E	L	G	F	E	Y	V	S	L	D	E	L	Y	A	N	S	D	I	I	S	L	N	C	P	L
				T	K	D	T	K	Y	M	I	N	R	R	S	M	L	K	M	K	D	G	V	I	L	V	N	T	G	R	G	M	L
				I	D	S	A	D	L	V	E	A	L	K	D	K	K	I	G	A	V	A	L	D	V	Y	E	E	E	E	N	Y	F
				F	E	D	K	S	T	Q	V	I	E	D	D	I	L	G	R	L	L	S	F	Y	N	V	L	I	T	S	H	Q	A
				Y	F	T	K	E	A	V	G	A	I	T	V	T	T	L	N	N	I	K	D	F	V	E	G	R	P	L	V	N	E
				V	P	Q	N	Q																									
				1				5					10					15					20					25					30
72	16.07%	99.0%	>tr|A0A117MWD9|A0A117MWD9_FUSNC UDP-N-acetylglucosamine 46-dehydratase (Inverting) OS=Fusobacterium nucleatum subsp. nucleatum OX=76856 GN=RO03_08765 PE=4 SV=1																														
				M	L	N	N	S	T	I	L	I	T	G	G	T	G	S	F	G	N	R	F	I	E	R	I	L	S	E	Y	N	P
				K	K	I	I	I	Y	S	R	D	E	F	K	Q	D	L	M	K	K	N	F	V	M	K	Y	G	V	E	K	T	K
				K	L	R	F	F	I	G	D	V	R	D	K	E	R	L	Y	R	A	F	D	G	V	D	Y	V	I	H	A	A	A
				M	K	Q	V	P	A	C	E	Y	N	P	F	E	A	I	K	T	N	I	H	G	A	E	N	V	I	E	A	A	I
				D	R	G	V	K	K	V	V	A	L	S	T	D	K	A	V	N	P	I	N	L	Y	G	G	T	K	L	V	S	D
				K	L	F	I	S	A	N	A	Y	S	G	E	K	G	T	I	F	S	V	V	R	Y	G	N	V	A	G	S	R	G
				S	V	I	P	F	F	K	Q	L	L	S	Q	G	K	T	E	L	P	I	T	D	I	H	M	T	R	F	W	M	V
				L	D	D	A	V	N	L	V	L	K	A	L	K	E	S	K	G	G	E	T	F	V	F	K	N	P	S	F	L	I
				T	E	L	A	K	A	L	N	P	N	G	I	I	K	E	V	G	I	R	E	G	E	K	I	H	E	V	M	I	T
				R	D	D	A	K	Y	T	Y	D	Y	E	D	Y	Y	V	I	Y	P	N	F	E	W	W	N	K	E	K	I	K	S
				G	G	K	L	I	P	E	N	W	D	Y	N	S	G	T	N	D	W	W	L	D	A	K	E	L	E	K	R	I	A
				K	L	D	I	K	Y																								
				1				5					10					15					20					25					30
73	19.18%	99.0%	>sp|Q8RIG1|RS3_FUSNN 30S ribosomal protein S3 OS=Fusobacterium nucleatum subsp. nucleatum (strain ATCC 25586 / CIP 101130 / JCM 8532 / LMG 13131) OX=190304 GN=rpsC PE=3 SV=1																														
				M	G	Q	K	V	D	P	R	G	L	R	L	G	I	T	R	A	W	D	S	N	W	Y	A	D	K	K	E	Y	V
				K	Y	F	H	E	D	V	Q	I	K	E	F	I	K	K	N	Y	F	H	T	G	I	S	K	V	R	I	E	R	T
				S	P	S	Q	V	V	V	H	I	H	T	G	K	A	G	L	I	I	G	R	K	G	A	E	I	D	A	L	R	A
				K	L	E	K	L	T	G	K	K	V	T	V	K	V	Q	E	I	K	D	L	N	G	D	A	V	L	V	A	E	S
				I	A	A	Q	I	E	K	R	I	A	Y	K	K	A	M	T	Q	A	I	S	R	S	M	K	S	P	E	V	K	G
				I	K	V	M	I	S	G	R	L	N	G	A	E	I	A	R	S	E	W	A	V	E	G	K	V	P	L	H	T	L
				R	A	D	I	D	Y	A	V	A	T	A	H	T	T	Y	G	A	L	G	I	K	V	W	I	F	H	G	E	V	L
				P	S	K	K	E	G	G	E	A																					
				1				5					10					15					20					25					30
74	12.38%	99.0%	>tr|Q8R6E8|Q8R6E8_FUSNN ADP-heptose synthase OS=Fusobacterium nucleatum subsp. nucleatum (strain ATCC 25586 / CIP 101130 / JCM 8532 / LMG 13131) OX=190304 GN=FN1786 PE=4 SV=1																														
				M	I	M	I	S	K	L	I	E	N	F	K	N	I	K	I	A	V	I	G	D	L	M	L	D	E	Y	I	M	G
				K	V	D	R	I	S	P	E	A	P	V	P	V	V	K	V	T	E	E	K	F	V	L	G	G	A	A	N	V	I
				N	N	L	A	A	L	G	A	N	V	Y	C	G	G	L	V	G	K	D	K	N	A	E	K	L	I	N	A	F	P
				K	N	V	D	C	N	L	I	L	K	V	E	N	R	P	T	I	V	K	K	R	V	I	A	G	H	Q	Q	L	L
				R	L	D	W	E	E	E	F	Y	I	N	E	D	E	E	N	I	I	I	E	N	L	K	N	H	I	K	N	L	N
				A	V	I	L	S	D	Y	N	K	G	L	L	T	K	S	L	S	Q	K	I	I	N	L	C	R	E	N	N	V	I
				V	T	V	D	P	K	P	K	N	I	S	N	F	M	G	A	S	S	I	T	P	N	K	K	E	A	Y	T	A	V
				E	A	N	P	S	E	N	I	D	I	V	G	E	K	L	K	E	K	Y	N	L	D	T	V	L	V	T	R	S	E
				E	G	M	T	L	Y	D	K	E	I	H	N	I	P	T	Y	A	K	E	V	Y	D	V	T	G	A	G	D	T	V
				I	S	V	F	T	L	A	K	A	A	G	A	T	W	E	E	A	A	K	I	A	N	A	A	G	G	I	V	V	G
				K	I	G	T	S	T	V	S	E	M	E	L	I	E	T	Y	N	S	I	Y	S	N	N							
				1				5					10					15					20					25					30
75	12.84%	99.0%	>sp|P58824|ASNA_FUSNN Aspartate--ammonia ligase OS=Fusobacterium nucleatum subsp. nucleatum (strain ATCC 25586 / CIP 101130 / JCM 8532 / LMG 13131) OX=190304 GN=asnA PE=3 SV=1																														
				M	A	Y	I	S	S	L	D	I	L	E	T	E	I	A	I	K	K	V	K	D	F	F	E	S	H	L	S	K	E
				L	D	L	L	R	V	S	A	P	L	F	V	I	P	E	S	G	L	N	D	N	L	N	G	T	E	R	P	V	S
				F	D	T	K	S	G	E	R	V	E	I	V	H	S	L	A	K	W	K	R	M	A	L	Y	R	Y	N	I	E	N
				D	K	G	I	Y	T	D	M	N	A	I	R	R	D	E	D	T	D	F	I	H	S	Y	Y	V	D	Q	W	D	W
				E	K	I	I	S	K	E	D	R	N	E	E	Y	L	K	D	V	V	R	K	I	Y	S	V	F	K	K	T	E	E
				Y	I	T	T	E	Y	P	K	L	T	K	K	L	P	E	E	I	T	F	I	T	A	Q	E	L	E	N	K	Y	P
				N	L	T	P	K	N	R	E	H	A	A	A	K	E	Y	G	A	I	F	L	M	K	I	G	G	K	L	S	S	G
				E	K	H	D	G	R	A	P	D	Y	D	D	W	D	L	N	G	D	I	I	F	N	Y	P	L	L	G	I	G	L
				E	L	S	S	M	G	I	R	V	D	E	K	S	L	D	E	Q	L	K	I	A	N	C	E	D	R	R	S	L	P
				Y	H	Q	M	I	L	N	K	V	L	P	Y	T	I	G	G	G	I	G	Q	S	R	I	C	M	F	F	L	D	K
				L	H	I	G	E	V	Q	A	S	I	W	S	Q	E	V	H	E	I	C	R	Q	M	N	I	K	L	L			
				1				5					10					15					20					25					30
76	6.05%	99.0%	>tr|Q8RHT4|Q8RHT4_FUSNN Outer membrane protein OS=Fusobacterium nucleatum subsp. nucleatum (strain ATCC 25586 / CIP 101130 / JCM 8532 / LMG 13131) OX=190304 GN=FN1911 PE=4 SV=1																														
				M	V	N	L	P	I	K	S	V	E	V	V	N	N	Q	Q	V	P	A	S	L	I	K	N	T	L	K	L	K	E
				G	A	K	F	S	T	E	A	L	L	A	D	F	N	A	L	K	E	T	G	Y	F	E	D	V	I	L	Q	P	V
				S	Y	D	G	G	V	R	I	V	V	D	V	V	E	K	E	N	V	V	D	L	L	K	E	K	G	V	A	I	N
				T	L	R	E	D	T	D	K	S	I	V	L	S	S	V	K	F	T	G	N	K	R	V	T	T	S	E	L	L	D
				I	T	Q	L	K	A	G	E	Y	F	S	R	S	R	V	E	D	A	Q	R	R	L	L	A	T	G	K	F	S	E
				V	R	P	D	A	Q	V	A	N	G	K	M	A	L	S	F	E	V	V	E	N	P	I	V	K	S	V	I	I	T
				G	N	N	T	I	P	T	S	T	I	M	S	E	L	T	T	K	P	G	S	V	Q	N	Y	N	N	L	R	E	D
				R	D	K	I	L	G	L	Y	Q	A	Q	G	Y	T	L	V	N	I	T	D	M	S	T	D	E	N	G	T	L	H
				I	S	I	V	E	G	I	V	R	R	I	E	V	K	K	M	V	T	K	Q	K	G	N	R	R	T	P	N	D	D
				V	L	K	T	K	D	Y	V	I	D	R	E	I	E	I	Q	P	G	K	I	F	N	V	K	E	Y	D	A	T	V
				D	N	L	M	R	L	G	I	F	K	N	V	K	Y	E	A	R	S	I	P	G	D	P	E	G	I	D	L	I	L
				L	I	D	E	D	R	T	A	E	L	Q	G	G	V	A	Y	G	S	E	T	G	F	L	G	T	L	S	L	K	D
				S	N	W	R	G	K	N	Q	Q	F	G	F	T	F	E	K	S	N	K	N	Y	T	G	F	A	L	D	F	Y	D
				P	W	I	K	D	T	D	R	V	S	W	G	W	G	A	Y	R	T	S	Y	G	D	E	D	S	I	L	F	H	E
				I	D	T	I	G	F	R	T	N	I	G	K	G	L	G	K	N	F	T	L	S	L	G	T	K	V	E	Y	I	K
				E	K	H	E	D	G	K	L	R	Q	A	N	N	G	K	W	Y	Y	K	E	K	N	K	W	R	E	I	E	G	V
				D	D	K	Y	W	L	W	S	I	Y	P	Y	I	S	Y	D	T	R	N	N	Y	L	N	P	T	S	G	F	Y	G
				K	F	Q	V	E	A	G	H	A	G	G	Y	K	S	G	N	F	G	N	A	T	L	E	L	R	T	Y	H	K	G
				L	F	K	N	N	I	F	A	Y	K	V	V	G	G	V	A	T	N	N	T	K	E	S	Q	K	F	W	V	G	G
				G	N	S	L	R	G	Y	D	G	G	F	F	K	G	S	Q	K	L	V	A	T	I	E	N	R	T	Q	L	N	D
				I	I	G	L	V	V	F	A	D	A	G	R	A	W	K	Q	N	G	R	D	P	S	Y	T	R	D	N	S	R	F
				G	H	N	I	G	T	T	A	G	V	G	I	R	L	N	T	P	I	G	P	L	R	F	D	F	G	W	P	V	G
				N	K	M	D	D	D	G	M	K	F	Y	F	N	M	G	Q	S	F												
				1				5					10					15					20					25					30
77	7.55%	98.7%	>tr|Q8R5V8|Q8R5V8_FUSNN ACYL-COA dehydrogenaseshort-chain specific OS=Fusobacterium nucleatum subsp. nucleatum (strain ATCC 25586 / CIP 101130 / JCM 8532 / LMG 13131) OX=190304 GN=FN1424 PE=3 SV=1																														
				M	L	F	K	T	T	E	E	H	E	A	L	R	M	Q	V	R	E	F	V	E	T	E	V	K	P	I	A	A	I
				L	D	K	E	N	K	F	P	H	E	A	I	K	K	F	G	Q	M	G	F	M	G	L	P	Y	P	K	E	Y	G
				G	A	G	K	D	I	L	S	Y	A	I	A	V	E	E	L	S	R	V	D	G	G	T	G	V	I	L	S	A	H
				V	S	L	G	S	Y	P	I	F	A	F	G	T	E	E	Q	K	K	K	Y	L	T	P	L	A	K	G	E	K	L
				G	A	F	G	L	T	E	P	N	A	G	S	D	A	G	G	T	E	T	T	A	V	K	E	G	D	Y	Y	I	L
				N	G	E	K	I	F	I	T	N	A	D	V	A	E	T	Y	V	V	F	A	V	T	T	P	D	I	G	T	K	G
				I	S	A	F	I	V	E	K	G	W	E	G	F	T	F	G	D	H	Y	D	K	L	G	I	R	S	S	S	T	C
				Q	L	L	F	N	N	V	K	V	P	K	E	N	L	L	G	K	E	G	D	G	F	K	I	A	M	S	T	L	D
				G	G	R	I	G	I	A	A	Q	A	L	G	I	A	Q	G	A	F	E	H	A	L	E	Y	A	K	E	R	E	Q
				F	G	K	P	I	A	F	Q	Q	A	I	S	F	K	L	A	D	M	A	T	K	L	R	T	A	R	F	L	I	Y
				S	A	A	E	L	K	E	H	H	E	P	Y	G	M	E	S	A	M	A	K	Q	Y	A	S	D	I	A	L	E	V
				V	N	D	A	V	Q	I	F	G	G	S	G	Y	L	K	G	M	E	V	E	R	A	Y	R	D	A	K	I	T	T
				I	Y	E	G	T	N	E	I	Q	R	V	V	I	A	A	H	L	I	G	K	A	P	K	S	D	A	I	A	V	A
				K	K	K	K	G	P	V	T	G	P	R	K	N	I	I	F	K	D	G	S	T	K	E	K	V	A	A	L	V	A
				A	L	K	A	D	G	Y	D	F	T	V	G	I	P	L	D	T	P	I	G	K	S	E	R	V	V	S	A	G	K
				G	I	G	D	K	K	N	M	K	L	I	E	N	L	A	K	Q	A	G	A	S	V	G	C	S	R	P	V	A	E
				T	L	Q	Y	L	P	L	D	R	Y	V	G	M	S	G	Q	K	F	V	G	N	L	Y	I	A	C	G	I	S	G
				A	L	Q	H	L	K	G	I	K	D	A	T	T	I	V	A	I	N	T	N	A	N	A	P	I	F	K	N	A	D
				Y	G	I	V	G	D	V	A	E	I	L	P	L	L	T	K	E	L	D	N	G	E	A	K	K	D	A	P	P	M
				K	K	M	K	R	V	I	P	K	V	V	Y	S	P	H	V	Y	V	C	S	G	C	G	H	E	Y	N	P	E	I
				G	D	E	D	S	D	I	K	P	G	T	R	F	K	D	L	P	E	D	W	T	C	P	D	C	G	D	P	K	S
				G	Y	I	D	A	K																								

				1				5					10					15					20					25					30
78	8.02%	99.0%	>tr|Q8RFC1|Q8RFC1_FUSNN Urocanate hydratase OS=Fusobacterium nucleatum subsp. nucleatum (strain ATCC 25586 / CIP 101130 / JCM 8532 / LMG 13131) OX=190304 GN=hutU PE=3 SV=1																														
				M	L	N	N	K	N	I	Y	D	A	M	T	I	K	L	T	A	E	D	I	P	M	E	I	P	K	L	D	P	S
				I	R	R	A	P	K	R	I	V	K	L	S	D	H	D	I	E	L	A	L	R	N	A	L	R	Y	I	P	E	E
				F	H	E	M	L	A	P	E	F	L	K	E	L	E	D	R	G	R	I	Y	G	Y	R	F	R	P	E	G	N	I
				Y	G	R	P	I	D	E	Y	K	G	K	C	T	E	A	K	A	M	Q	V	M	I	D	N	N	L	D	F	D	I
				A	L	Y	P	Y	E	L	V	T	Y	G	E	T	G	Q	V	C	Q	N	W	M	Q	Y	R	L	I	K	K	Y	L
				E	N	M	T	Q	D	Q	T	L	V	V	A	S	G	H	P	T	G	L	F	R	S	N	P	Y	A	P	R	A	I
				I	T	N	G	L	M	V	G	L	F	D	N	Y	D	D	W	A	R	G	A	A	I	G	V	A	N	Y	G	Q	M
				T	A	G	G	W	M	Y	I	G	P	Q	G	I	V	H	G	T	Y	S	T	I	L	N	A	G	R	L	F	C	G
				V	P	A	D	G	D	L	R	G	K	L	F	I	T	S	G	L	G	G	M	S	G	A	Q	G	K	A	C	E	I
				A	K	G	V	A	I	V	A	E	V	D	L	S	R	I	N	T	R	L	E	Q	G	W	V	N	V	I	A	N	T
				P	E	E	A	F	K	I	A	E	E	K	M	A	S	K	T	P	Y	A	I	A	Y	H	G	N	I	V	E	I	L
				E	Y	A	I	E	H	N	K	H	I	D	L	L	S	D	Q	T	S	C	H	A	V	Y	D	G	G	Y	C	P	V
				G	T	S	F	E	E	R	T	K	L	L	G	T	D	R	A	K	F	R	E	L	V	N	E	G	L	K	R	H	Y
				K	A	I	K	T	L	H	D	R	G	V	Y	F	F	D	Y	G	N	S	F	L	K	S	I	Y	D	V	G	V	T
				E	I	S	K	N	G	K	D	D	K	E	G	F	I	F	P	S	Y	V	E	D	I	L	G	P	E	L	F	D	Y
				G	Y	G	P	F	R	W	V	C	L	S	R	K	K	E	D	L	L	K	T	D	K	A	A	L	E	L	V	D	P
				N	R	R	Y	Q	D	R	D	N	Y	V	W	I	Q	D	A	D	K	N	G	L	V	V	G	T	Q	A	R	I	F
				Y	Q	D	A	M	S	R	T	R	I	A	L	K	F	N	E	M	V	R	N	G	E	I	G	P	V	M	L	G	R
				D	H	H	D	V	S	G	T	D	S	P	F	R	E	T	S	N	I	K	D	G	S	N	I	M	A	D	M	A	T
				Q	C	F	A	G	N	A	A	R	G	M	T	M	I	A	L	H	N	G	G	G	V	G	I	G	K	S	I	N	G
				G	F	G	M	V	L	D	G	S	K	R	V	D	E	I	L	W	Q	A	M	P	W	D	V	M	G	G	V	A	R
				R	A	W	A	R	N	P	H	S	I	E	T	V	V	E	Y	N	L	D	N	K	G	T	D	H	I	T	L	P	Y
				I	V	N	D	E	L	V	K	K	V	L	K	K																	
				1				5					10					15					20					25					30
79	5.79%	99.0%	>tr|R9RCA8|R9RCA8_FUSNU Exodeoxyribonuclease 7 large subunit OS=Fusobacterium nucleatum subsp. animalis 4_8 OX=469607 GN=xseA PE=3 SV=1																														
				M	E	K	I	Y	S	V	S	E	F	N	R	M	V	K	S	Y	I	D	D	I	D	D	F	Q	E	F	F	I	E
				G	E	I	S	N	I	T	Y	Y	K	S	G	H	L	Y	F	S	I	K	D	S	K	S	Q	I	K	C	A	A	F
				N	Y	K	L	K	R	I	P	E	D	L	K	E	G	D	L	I	K	L	F	G	D	V	G	F	Y	E	V	R	G
				E	F	Q	V	L	V	R	Y	V	E	K	Q	N	T	L	G	S	L	Y	A	K	L	E	K	V	K	E	K	L	S
				E	L	G	Y	F	D	E	K	H	K	R	D	L	P	K	F	P	K	N	I	G	V	V	T	A	L	T	G	A	A
				L	Q	D	I	I	K	T	T	R	K	R	F	N	S	I	N	I	Y	I	Y	P	A	K	V	Q	G	I	G	A	E
				Q	E	I	I	K	G	I	E	T	L	N	K	I	E	E	I	D	F	I	I	A	G	R	G	G	G	S	I	E	D
				L	W	A	F	N	E	E	E	V	A	M	A	F	F	N	S	K	K	P	I	I	S	A	V	G	H	E	I	D	F
				L	L	S	D	L	T	A	D	K	R	A	A	T	P	T	Q	A	I	E	L	S	V	P	E	K	E	N	L	L	Q
				D	L	K	A	R	E	I	Y	I	T	K	L	L	K	S	Y	V	D	N	M	K	R	E	L	L	L	R	V	E	N
				Y	H	L	K	N	F	P	N	T	I	N	N	L	R	E	S	I	V	E	K	E	I	Q	L	K	E	I	M	E	S
				F	L	A	Q	K	R	N	I	F	E	T	K	I	D	K	I	S	V	L	N	P	I	N	T	L	K	R	G	Y	T
				V	S	Q	I	K	N	K	R	I	D	V	L	D	D	I	E	I	N	D	E	M	V	T	I	L	K	D	G	K	V
				I	S	I	V	K	E	K	I	Y	E	K	I	L	I	S	L	F	L	I	I	S	V	I	G	L	S	E	S	E	R
				E	T	G	I	T	A	E	R	N	E	T	P	V	T	Q	T	E	T	S	T	S	T	E	S	G	T	D	D	G	G
				E	T	V	E	N	P	E	Q	Q	K	T	T	A	G	F	Y	E	Y	R	P	Q	S	L	I	Q	L	D	E	Q	M
				K	S	A	G	R	G	S	V	G	Q	L	N	A	R	Y	E	H	E	L	N	A	Y	L	E	M	Y	S	Y	D	S
				D	R	I	F	Y	L	A	N	E	Y	M	L	L	N	N	Y	S	R	A	N	K	I	F	L	K	D	N	K	D	I
				K	N	V	F	G	A	A	T	T	Y	R	F	M	G	Q	N	E	N	A	I	Q	K	Y	T	Q	A	I	S	M	N
				P	N	F	A	E	S	Y	L	G	R	G	L	A	N	R	N	L	D	N	Y	D	S	A	V	N	D	L	Q	T	Y
				I	S	K	T	G	A	H	D	G	Y	V	A	L	A	D	V	Y	F	K	M	G	K	N	K	E	A	Y	N	I	A
				S	Q	G	L	V	K	Y	R	D	S	R	I	L	R	V	L	A	N	N	I	L	K	N	K	I	D				
				1				5					10					15					20					25					30
80	12.65%	99.0%	>tr|A0A0M1VVT3|A0A0M1VVT3_FUSNV Heat-inducible transcription repressor HrcA OS=Fusobacterium nucleatum subsp. vincentii 4_1_13 OX=469606 GN=hrcA PE=3 SV=1																														
				M	R	I	S	E	R	E	K	L	V	L	N	A	I	V	D	Y	Y	L	T	V	G	D	T	I	G	S	R	T	L
				V	K	K	Y	G	I	E	L	S	S	A	T	I	R	N	V	M	A	D	L	E	D	M	G	F	I	E	K	T	H
				T	S	S	G	R	I	P	T	D	M	G	Y	K	Y	Y	L	T	E	L	L	K	V	E	K	I	T	Q	E	E	I
				E	N	I	S	N	V	Y	D	R	R	V	D	E	L	E	N	I	L	K	K	T	S	T	L	L	S	K	L	T	N
				Y	A	G	I	A	V	E	P	K	P	D	N	K	K	V	S	R	V	E	L	V	Y	I	D	E	Y	L	I	M	A
				I	I	V	M	D	D	R	R	V	K	T	K	N	I	H	L	P	Y	P	I	S	K	E	E	V	E	K	K	V	D
				E	L	N	A	K	I	K	N	N	E	I	A	I	N	D	I	E	K	F	F	T	E	S	T	D	I	V	Y	E	Y
				D	D	E	D	E	L	T	K	Y	F	I	N	N	L	P	S	M	L	K	N	E	N	I	A	E	V	T	D	V	I
				E	F	F	N	E	R	K	D	I	R	E	L	F	E	K	L	I	E	Q	K	A	K	E	N	S	N	S	N	V	N
				V	I	L	G	D	E	L	G	I	K	E	L	E	D	F	S	F	V	Y	S	I	Y	D	I	G	G	A	Q	G	I
				I	G	V	M	G	P	K	R	M	A	Y	S	K	T	M	G	L	I	N	H	V	S	R	E	V	N	K	L	I	N
				S	M	E	K	D	K	N	K	K	V																				
				1				5					10					15					20					25					30
81	10.29%	99.0%	>tr|Q8RFA6|Q8RFA6_FUSNN Low-specificity threonine aldolase OS=Fusobacterium nucleatum subsp. nucleatum (strain ATCC 25586 / CIP 101130 / JCM 8532 / LMG 13131) OX=190304 GN=FN0810 PE=4 SV=1																														
				M	I	N	F	K	N	D	Y	S	E	G	A	C	S	E	V	L	E	A	L	I	K	T	N	Y	E	Q	T	V	G
				Y	G	E	D	E	Y	C	E	K	A	K	N	L	I	K	E	N	I	N	Y	P	N	A	N	I	Y	F	L	V	G
				G	T	Q	A	N	T	T	V	I	S	H	S	L	R	P	Y	E	A	V	I	A	C	K	T	G	H	I	S	I	H
				E	T	G	A	I	E	A	T	G	H	K	I	I	E	V	D	G	I	D	G	K	L	T	P	D	L	I	L	N	E
				L	R	K	H	E	D	H	H	M	V	K	P	K	M	V	Y	I	S	N	T	T	E	I	G	T	V	Y	T	K	K
				E	L	E	N	I	S	K	V	C	K	D	N	N	L	Y	L	Y	L	D	G	A	R	L	A	S	A	L	T	S	E
				K	C	D	I	N	L	E	D	Y	P	R	Y	C	D	V	F	Y	I	G	G	T	K	C	G	L	L	F	G	E	A
				V	V	I	I	N	D	E	I	K	K	E	F	N	F	S	V	K	Q	K	G	G	L	F	A	K	G	R	L	L	G
				I	Q	F	A	T	L	F	K	D	D	L	Y	Y	R	I	G	V	H	S	N	K	M	A	L	K	I	K	N	A	F
				I	E	K	G	I	K	L	A	T	D	S	Y	T	N	Q	V	F	V	D	L	S	Q	E	Q	I	K	K	L	E	K
				D	V	I	F	S	V	E	F	F	G	I	G	E	S	Q	S	S	R	F	V	T	S	W	A	T	K	E	E	D	I
				D	K	L	V	E	L	I	K	S	L																				
				1				5					10					15					20					25					30
82	13.91%	99.0%	>tr|Q8RHB8|Q8RHB8_FUSNN Hypothetical exported 24-amino acid repeat protein OS=Fusobacterium nucleatum subsp. nucleatum (strain ATCC 25586 / CIP 101130 / JCM 8532 / LMG 13131) OX=190304 GN=FN2119 PE=4 SV=1																														
				M	K	K	L	L	L	A	L	F	L	I	F	S	V	L	L	F	S	A	E	R	K	T	P	V	E	Q	T	F	K
				D	E	N	S	G	K	V	Y	V	Q	G	E	K	T	P	Y	T	G	I	V	E	G	K	Y	S	N	G	K	V	K
				I	L	V	S	Y	K	N	G	I	L	N	G	K	V	L	Q	Y	Y	E	N	G	K	I	K	S	E	D	T	F	M
				N	E	A	L	N	G	V	S	K	G	Y	Y	E	N	G	K	V	E	Y	E	I	S	Y	K	N	D	K	K	D	G
				I	E	K	R	Y	S	N	T	G	I	L	V	V	E	F	S	Y	K	N	D	E	L	N	G	I	I	K	K	Y	N
				E	N	T	G	K	L	E	M	E	A	L	Y	K	N	D	K	I	E	G	I	A	K	S	Y	Y	P	S	G	K	L
				E	R	E	Q	T	Y	K	N	N	I	R	D	G	I	S	K	E	Y	Y	E	N	G	K	L	Q	M	Q	T	N	Y
				K	N	G	Q	L	E	G	I	N	K	R	Y	S	Q	K	G	V	L	E	E	E	V	E	Y	K	N	G	K	M	E
				G	S	S	K	L	F	Y	P	S	G	K	L	K	S	I	A	T	F	K	N	G	L	Q	V	G	V	Q	K	D	Y
				Y	E	D	G	K	L	K	L	E	T	L	Y	K	D	G	N	K	N	G	I	T	K	S	Y	Y	P	N	G	K	V
				Q	V	E	V	N	F	K	N	G	Q	L	D	G	V	L	K	Q	F	D	E	N	G	R	L	I	N	Q	E	T	Y
				Q	N	G	N	K	I	N	N																						
				1				5					10					15					20					25					30
83	5.33%	99.0%	>tr|A0A0M1VU74|A0A0M1VU74_FUSNV Peptidylprolyl isomerase OS=Fusobacterium nucleatum subsp. vincentii 4_1_13 OX=469606 GN=FSCG_00886 PE=4 SV=1																														
				M	S	I	R	K	F	R	K	Q	M	K	P	F	I	I	I	L	T	V	V	F	I	L	S	L	A	Y	G	G	Y
				E	S	Y	K	T	S	R	A	N	K	K	A	Q	E	A	M	L	L	N	K	D	Y	I	Q	K	I	E	I	E	R
				A	K	Q	D	L	S	R	T	Y	A	D	R	V	D	K	D	I	V	D	I	L	A	F	N	D	V	I	D	K	N
				L	T	L	H	I	A	K	D	L	K	V	K	V	P	S	S	E	V	D	K	Q	Y	E	E	L	E	S	S	M	G
				D	K	E	Q	F	R	R	M	L	Q	V	R	G	L	T	K	D	S	L	K	N	Q	I	E	E	N	L	L	M	Q
				Q	T	R	E	E	F	A	K	N	I	N	P	T	D	E	E	I	N	A	Y	M	A	L	Y	S	I	P	A	D	K
				K	E	E	A	V	N	L	Y	K	S	E	K	G	A	E	A	F	R	E	A	L	L	K	A	R	K	E	M	Q	I
				K	D	L	A	P	E	Y	E	N	L	I	E	K	T	A	Y	E	E	E	G	F	T	I	T	N	L	D	L	A	R
				A	T	A	N	V	M	L	G	Q	K	I	S	K	E	D	A	E	K	Q	A	K	E	L	I	S	R	Q	I	K	M
				V	K	I	A	K	E	K	G	V	K	V	N	E	N	L	D	T	I	S	Q	F	Q	D	Y	Y	V	G	L	A	E
				K	V	R	D	E	V	K	P	T	D	E	D	L	L	K	F	F	N	E	N	K	S	K	Y	S	I	P	A	T	A
				D	A	K	L	V	F	I	S	V	K	S	A	K	E	D	D	N	L	A	K	E	K	A	E	K	L	L	S	E	L
				T	P	E	N	F	T	E	K	G	K	S	L	S	N	N	Q	D	I	I	Y	Q	D	L	G	T	F	G	T	T	A
				M	V	K	E	F	E	E	A	L	K	D	V	P	S	N	T	V	V	N	K	V	I	K	T	K	F	G	Y	H	V
				A	Y	V	K	K	N	D	N	N	R	Q	W	A	V	E	H	I	L	I	V	P	Y	P	S	E	K	T	M	T	E
				K	L	E	K	L	N	K	V	K	A	D	I	E	A	G	T	L	T	L	N	D	K	I	D	E	D	V	I	Q	S
				F	D	A	K	G	I	T	P	D	G	I	I	P	N	F	I	Y	D	P	E	I	A	K	A	V	F	G	T	E	L
				N	K	V	G	I	I	N	P	N	K	A	T	I	V	V	F	Q	K	T	K	E	V	K	A	E	D	A	N	F	N
				K	S	K	E	Q	V	K	S	D	Y	I	N	N	K	V	A	E	Y	M	S	K	L	F							
				1				5					10					15					20					25					30
84	10.85%	99.0%	>sp|Q8RFY0|RECA_FUSNN Protein RecA OS=Fusobacterium nucleatum subsp. nucleatum (strain ATCC 25586 / CIP 101130 / JCM 8532 / LMG 13131) OX=190304 GN=recA PE=3 SV=2																														
				M	A	A	K	K	D	K	S	V	P	D	S	K	I	T	D	K	E	G	K	E	K	A	V	K	D	A	M	A	A
				I	T	K	G	F	G	S	G	L	I	M	K	L	G	E	K	S	S	M	N	V	E	S	I	P	T	G	S	I	N
				L	D	I	A	L	G	I	G	G	V	P	K	G	R	I	I	E	I	Y	G	A	E	S	S	G	K	T	T	L	A
				L	H	V	I	A	E	A	Q	K	Q	G	G	T	V	A	F	I	D	A	E	H	A	L	D	P	V	Y	A	K	A
				L	G	V	D	I	D	E	L	L	I	S	Q	P	D	Y	G	E	Q	A	L	E	I	A	D	T	L	V	R	S	G
				A	I	D	L	I	V	I	D	S	V	A	A	L	V	P	K	A	E	I	D	G	E	M	S	D	Q	Q	M	G	L
				Q	A	R	L	M	S	K	G	L	R	K	L	T	G	N	L	N	K	Y	K	T	T	M	I	F	I	N	Q	I	R
				E	K	I	G	V	T	Y	G	P	T	T	T	T	T	G	G	K	A	L	K	F	Y	S	S	V	R	M	E	V	K
				K	M	G	T	V	K	Q	G	D	D	P	I	G	S	E	V	I	V	K	V	T	K	N	K	V	A	P	P	F	K
				E	A	A	F	E	I	L	Y	G	K	G	I	S	K	V	G	E	I	I	D	A	A	V	A	K	D	V	I	V	K
				A	G	S	W	F	S	F	R	D	Q	S	I	G	Q	G	K	E	K	V	R	A	E	L	E	I	N	P	E	L	L
				A	Q	V	E	K	D	L	K	E	A	I	A	K	G	P	V	D	K	K	K	K	K	S	K	K	E	A	S	S	D
				D	T	D	D	E	N	L	E	I	D	D	A	I	D	E	N	N	D												
				1				5					10					15					20					25					30
85	8.20%	99.0%	>tr|A0A0M1VTX9|A0A0M1VTX9_FUSNV Inosine-5'-monophosphate dehydrogenase OS=Fusobacterium nucleatum subsp. vincentii 4_1_13 OX=469606 GN=guaB PE=3 SV=1																														
				M	M	N	G	K	I	V	K	E	G	I	T	F	D	D	V	L	L	I	P	A	K	S	D	V	L	P	N	E	V
				S	L	Q	T	R	L	T	K	K	I	T	L	N	L	P	I	L	S	A	A	M	D	T	V	T	E	S	D	L	A
				I	A	L	A	R	Q	G	G	I	G	F	I	H	K	N	M	S	I	E	E	Q	A	A	E	V	D	R	V	K	R

S	E	S	G	M	I	T	N	P	I	T	L	N	K	D	S	R	V	Y	Q	A	E	E	L	M	S	R	Y	K	I
S	G	L	P	V	I	E	D	D	G	K	L	I	G	I	I	T	N	R	D	I	K	Y	R	K	E	L	D	Q	P
V	G	D	I	M	T	S	K	G	L	I	T	A	P	V	G	T	T	L	E	Q	A	K	E	I	L	L	A	N	R
I	E	K	L	P	I	T	D	Q	N	G	Y	L	K	G	L	I	T	I	K	D	I	D	N	I	I	Q	Y	P	N
A	C	K	D	K	L	G	K	L	R	C	G	A	A	V	G	I	G	H	D	T	I	E	R	V	G	A	L	V	K
A	G	V	D	I	I	T	V	D	S	A	H	G	H	S	Q	G	V	I	N	M	I	K	E	I	K	K	N	F	P
D	L	D	V	I	G	G	N	I	V	T	A	E	A	A	K	E	L	I	E	A	G	V	S	A	V	K	V	G	I
G	P	G	S	I	C	T	T	R	V	V	A	G	V	G	V	P	Q	L	T	A	V	N	D	V	Y	E	Y	C	K
D	K	N	I	G	V	I	A	D	G	G	I	K	L	S	G	D	I	V	K	A	L	A	A	G	G	D	C	V	M
L	G	G	L	L	A	G	T	K	E	A	P	G	E	E	I	I	L	E	G	R	R	F	K	I	Y	V	G	M	G
S	I	A	A	M	K	R	G	S	K	D	R	Y	F	Q	A	E	E	R	D	N	S	K	L	V	P	E	G	I	E
G	R	I	A	Y	K	G	S	V	K	D	V	I	F	Q	L	A	G	G	I	R	A	G	M	G	Y	C	G	T	K
T	I	K	D	L	Q	I	N	G	K	F	V	K	I	T	G	A	G	L	I	E	S	H	P	H	D	I	T	I	T
K	E	A	P	N	Y	S	K																						
1				5					10					15					20					25					30

86 9.52% 99.0% >tr|Q8RIR1|Q8RIR1_FUSNN Riboflavin biosynthesis protein RibBA OS=Fusobacterium nucleatum subsp. nucleatum (strain ATCC 25586 / CIP 101130 / JCM 8532 / LMG 13131) OX=190304 GN=ribBA PE=3 SV=1

M	I	Y	K	I	E	D	V	L	E	D	I	K	N	G	I	P	L	I	I	V	D	D	E	N	R	E	N	E	G
D	L	F	V	A	A	E	K	A	T	Y	E	S	I	N	L	M	A	T	F	A	R	G	L	T	C	T	P	M	T
S	E	Y	A	I	R	L	G	L	D	P	M	T	A	R	N	T	D	A	K	C	T	A	F	T	V	S	V	D	A
K	E	G	T	T	T	G	I	S	I	A	D	R	L	T	T	I	K	K	L	A	N	K	N	S	V	P	S	D	F
T	K	P	G	H	I	F	P	L	I	A	K	D	K	G	V	L	E	R	E	G	H	T	E	A	T	V	D	L	C
K	I	C	G	L	T	P	V	S	V	I	C	E	I	L	K	D	D	G	T	M	A	R	M	D	D	L	E	I	F
A	K	E	H	N	L	K	I	I	T	I	A	D	L	I	K	Y	R	K	K	T	E	Q	L	M	K	I	D	V	V
A	N	M	P	T	D	S	G	T	F	K	I	V	G	F	D	N	L	I	D	G	K	E	H	I	A	L	V	K	G
D	V	A	G	K	E	N	V	T	V	R	I	H	S	E	C	F	T	G	D	I	L	G	S	L	R	C	D	C	G
S	Q	L	K	T	A	M	R	R	I	D	K	L	G	E	G	I	I	L	Y	L	R	Q	E	G	R	G	I	G	L
L	N	K	L	R	A	Y	N	L	Q	E	E	G	M	D	T	L	D	A	N	L	H	L	G	F	G	A	D	M	R
D	Y	A	V	A	A	Q	M	L	K	A	L	G	V	K	S	I	K	L	L	T	N	N	P	L	K	I	N	G	L
E	E	Y	G	I	P	V	V	K	R	E	E	I	E	I	E	A	N	K	I	N	K	V	Y	L	K	T	K	K	E
R	M	G	H	L	L	K	I	K																					
1				5					10					15					20					25					30

87 11.94% 99.0% >tr|D5RFX9|D5RFX9_FUSN2 Uncharacterized protein OS=Fusobacterium nucleatum subsp. nucleatum (strain ATCC 23726 / VPI 4351) OX=525283 GN=HMPREF0397_2114 PE=4 SV=1

M	K	K	L	L	N	K	I	V	L	F	L	I	L	S	L	T	A	F	S	Y	N	F	P	I	D	D	P	Y	S
A	T	I	I	G	S	A	T	M	M	T	P	G	V	S	E	N	I	P	L	K	V	Y	K	I	Q	I	K	D	K
K	E	T	P	D	V	F	W	Y	A	S	K	F	K	F	S	F	S	K	Q	K	N	K	K	A	P	L	I	F	V
L	A	G	T	G	S	D	Y	S	T	T	R	V	K	F	M	Q	R	I	F	H	D	A	G	Y	H	T	I	A	I
S	S	Q	M	S	Q	Q	F	M	I	S	A	S	S	N	S	V	P	G	L	L	L	E	D	N	K	D	I	Y	K
A	M	K	L	A	Y	N	K	I	K	D	Q	V	D	V	T	D	F	Y	I	M	G	Y	S	L	G	G	T	N	A
A	V	L	S	Y	I	D	E	K	E	K	A	F	N	F	K	R	V	F	M	V	N	P	P	V	E	L	Y	D	S
A	V	K	L	D	K	Y	L	D	D	Y	T	G	G	K	T	K	G	I	E	K	L	L	N	T	T	L	A	R	V
K	G	G	L	T	N	E	Y	A	N	I	G	A	E	T	I	Y	E	I	I	K	G	D	I	L	S	E	A	E	K
K	A	Y	I	G	L	A	F	R	L	T	S	N	D	L	N	F	I	S	D	F	I	T	K	S	H	V	Y	T	K
N	P	E	K	V	N	K	F	T	N	M	K	E	Y	L	K	A	V	N	F	A	T	F	E	D	Y	V	N	K	V
G	F	P	Y	Y	K	K	Y	N	K	D	F	T	I	E	D	L	K	K	E	A	S	L	R	V	I	E	D	Y	L
R	T	S	P	K	I	A	A	V	T	N	A	D	E	L	I	L	N	E	K	D	I	D	Y	L	K	D	V	F	K
D	R	L	V	I	Y	P	K	G	G	H	C	G	N	M	F	Y	K	E	N	V	D	V	M	L	K	F	V	N	E
G	V	L	K	Y	E	N																							
1				5					10					15					20					25					30

88 1.56% 96.8% >tr|H1HEX8|H1HEX8_FUSNU Uncharacterized protein OS=Fusobacterium nucleatum subsp. animalis F0419 OX=999414 GN=HMPREF9942_01029 PE=4 SV=1

M	S	N	Y	F	C	N	I	S	N	S	R	S	Q	K	E	T	G	V	L	A	T	E	H	Y	E	Y	I	S	R
T	G	K	Y	S	P	E	E	E	K	E	N	Q	A	A	F	A	H	L	E	Y	V	Q	R	M	N	N	F	S	K
K	V	E	Y	E	D	L	V	Y	S	E	N	I	N	M	P	S	W	A	E	Q	N	P	N	S	F	W	I	A	S
E	T	Y	E	R	A	N	G	R	T	Y	S	E	F	L	F	S	L	P	H	E	L	T	D	E	E	N	K	E	I
V	D	R	F	C	N	K	T	F	G	K	D	F	V	Y	S	Y	G	I	H	S	K	P	S	S	E	E	G	V	Q
N	I	H	V	H	I	M	F	C	E	R	R	L	D	G	I	D	R	P	V	E	Q	F	F	K	R	Y	N	S	N
Y	P	E	R	G	G	A	K	K	D	R	Y	W	N	N	S	K	M	F	F	H	V	R	K	E	M	E	K	C	I
N	E	K	L	E	E	K	G	L	E	K	V	S	C	E	T	L	K	A	Q	R	L	V	A	L	S	A	G	D	I
L	K	A	E	F	L	D	R	P	P	V	N	C	P	G	S	I	L	M	K	I	N	K	Y	G	I	E	S	L	T
E	N	E	K	E	Q	Y	N	L	Y	L	T	T	K	E	I	R	K	I	S	L	E	E	Y	Q	R	K	L	Q	E
K	N	S	K	E	F	V	V	P	T	E	K	E	A	R	D	F	I	N	N	T	K	E	I	I	L	D	R	A	K
F	A	V	F	E	S	R	K	K	L	E	N	I	N	E	F	N	S	F	E	K	N	R	E	A	Y	L	E	E	Y
L	G	M	G	V	L	I	S	E	E	K	N	L	Q	L	K	Y	N	A	T	L	A	F	E	N	Y	T	Q	Q	N
L	N	K	V	Y	E	K	K	H	Q	E	N	F	S	S	L	K	H	N	E	F	N	F	S	N	R	Y	Y	Y	L
L	E	K	V	S	A	N	L	S	N	E	K	L	L	L	E	K	N	K	E	Q	L	L	N	N	Y	K	A	D	I
Y	S	Y	D	K	E	N	N	K	K	I	L	Q	Q	L	L	K	A	A	Y	N	D	S	R	K	E	S	I	E	T
I	L	N	K	Y	K	E	D	E	E	I	E	R	N	L	S	K	L	N	K	N	T	F	I	S	K	I	F	S	N
K	S	Q	E	I	E	I	T	K	L	N	I	E	K	E	K	I	K	E	E	I	D	K	L	F	T	S	T	F	Y
N	D	S	K	L	Q	T	T	F	I	L	K	K	L	E	L	L	D	K	E	K	N	T	D	T	Y	K	E	T	E
N	I	F	K	D	F	Y	K	K	Y	E	D	I	K	E	K	L	S	V	L	E	V	S	I	Q	Y	T	D	E	K
I	K	E	F	S	P	F	I	Q	E	Q	K	K	K	L	K	E	I	N	E	K	T	T	I	E	K	D	F	I	S
P	G	V	A	S	N	N	Y	I	D	T	T	V	E	N	I	R	E	Q	L	L	N	S	S	K	N	R	E	E	T
L	E	D	I	I	I	K	N	N	K	M	Y	S	E	L	K	A	I	E	N	K	I	L	S	F	E	K	I	L	K
D	E	K	K	I	K	E	I	S	I	K	K	N	L	Q	P	E	K	I	I	A	G	Y	Q	K	S	L	E	K	N
K	E	K	I	F	D	M	I	K	E	Y	K	N	E	R	R	K	I	Q	S	S	L	A	T	K	N	I	F	E	K
N	K	I	Y	E	T	I	L	Q	T	L	E	N	S	R	K	R	L	F	S	E	L	D	N	L	K	S	K	N	E
K	T	N	I	V	F	D	E	I	K	N	N	S	W	E	I	E	N	F	K	K	L	F	N	K	N	I	E	K	D
K	L	K	E	D	I	N	P	G	K	I	K	M	E	K	V	L	D	N	T	D	V	K	I	S	E	N	I	V	S
K	E	E	K	E	I	S	S	E	I	K	T	V	N	S	S	K	K	S	I	N	N	N	D	K	K	I	R	G	N
Y	I	N	I	I	P	K	K	K	K	R	E	S	L	V	D	E	D	D	L	N	I	K	E	Y	G	D	D		
1				5					10					15					20					25					30

89 5.61% 98.6% >sp|Q8RHM3|GLMU_FUSNN Bifunctional protein GlmU OS=Fusobacterium nucleatum subsp. nucleatum (strain ATCC 25586 / CIP 101130 / JCM 8532 / LMG 13131) OX=190304 GN=glmU PE=3 SV=1

M	K	S	I	I	M	A	A	G	K	G	T	R	M	K	S	D	L	P	K	V	V	H	L	A	H	G	K	P	M
I	V	R	I	I	D	A	L	N	T	L	D	V	E	E	N	I	L	I	L	G	H	K	R	E	K	V	L	E	V
L	G	N	D	V	S	Y	V	V	Q	E	E	Q	L	G	T	G	H	A	V	K	Q	A	I	P	K	I	K	D	Y
D	G	D	V	L	I	I	N	G	D	I	P	L	I	R	K	Q	T	L	I	D	F	Y	N	L	Y	K	N	E	N
A	D	G	I	I	L	S	A	I	F	E	N	P	F	S	Y	G	R	V	I	K	D	G	N	K	V	L	R	I	V
E	E	K	E	A	N	E	E	Q	K	K	V	K	E	I	N	A	G	V	Y	I	F	K	A	Q	A	L	V	K	A
L	E	K	I	N	N	N	N	E	K	G	E	Y	Y	I	T	D	V	I	E	I	L	S	N	D	K	K	V	I	S
Y	S	L	E	D	S	M	E	I	Q	G	V	N	S	K	V	E	L	A	L	V	S	K	V	L	R	E	R	K	N
T	A	L	M	E	D	G	V	I	L	I	D	P	A	T	T	Y	I	D	D	E	V	K	I	G	R	D	T	T	I
Y	P	N	V	T	L	Q	G	N	T	E	I	G	E	N	S	E	I	L	S	G	T	R	I	I	D	S	K	I	Y
D	N	V	R	I	E	S	S	V	I	E	E	S	I	V	E	N	G	V	T	I	G	P	Y	A	H	L	R	P	K
S	H	L	K	E	N	V	H	I	G	N	F	V	E	T	K	K	S	T	L	E	K	G	V	K	A	G	H	L	T
Y	L	G	D	A	H	I	G	E	K	T	N	I	G	A	G	T	I	T	C	N	Y	D	G	K	N	K	F	K	T
E	I	G	K	D	V	F	I	G	S	D	T	M	L	V	A	P	V	N	I	G	D	N	S	L	I	G	A	G	S
V	I	T	K	D	V	P	S	D	S	L	S	V	E	R	S	K	Q	I	I	K	E	G	W	K	K				
1				5					10					15					20					25					30

90 7.46% 98.2% >tr|D5RBV0|D5RBV0_FUSN2 ABC transporterATP-binding protein OS=Fusobacterium nucleatum subsp. nucleatum (strain ATCC 23726 / VPI 4351) OX=525283 GN=oppD2 PE=3 SV=1

M	E	N	K	N	L	L	E	I	R	D	L	E	I	Q	Y	V	K	D	D	E	T	V	H	A	V	N	G	I	S
V	D	I	A	E	G	E	T	L	G	L	V	G	E	T	G	A	G	K	T	T	T	A	L	G	I	M	R	L	I
T	G	P	T	G	K	I	K	S	G	A	I	K	F	N	G	K	S	I	L	E	I	P	E	E	E	M	R	K	I
R	G	N	D	I	S	M	I	F	Q	D	P	M	T	S	L	N	P	V	M	T	V	G	E	Q	I	A	E	V	I
E	I	H	E	H	I	G	K	E	E	A	M	N	K	A	A	E	M	L	E	L	V	G	I	P	G	A	R	K	N
D	F	P	H	Q	F	S	G	G	M	K	Q	R	V	V	I	A	I	A	L	A	C	N	P	K	L	L	I	A	D
E	P	T	T	A	L	D	V	T	I	Q	A	Q	V	L	D	L	M	T	D	L	K	N	K	F	K	T	S	M	L
L	I	T	H	D	L	G	V	V	A	Q	V	C	D	K	V	A	I	M	Y	A	G	E	I	V	E	Y	G	T	L
E	D	V	F	E	N	P	K	H	P	Y	T	L	G	L	F	G	S	I	P	S	L	D	E	E	K	T	R	L	V
P	I	K	G	L	M	P	D	P	T	N	L	P	S	G	C	K	F	N	P	R	C	P	H	A	V	E	L	C	S
Q	R	A	P	V	V	T	E	V	S	K	G	H	K	V	Q	C	L	I	A	E	G	L	V	K	F	K	E	N	W
E	E	E	N	E																									
1				5					10					15					20					25					30

91 5.27% 99.0% >tr|R9RAN7|R9RAN7_FUSNU AbgT transporter OS=Fusobacterium nucleatum subsp. animalis 4_8 OX=469607 GN=HMPREF0409_00202 PE=4 SV=1

M	E	K	E	K	K	K	G	I	Q	R	F	L	D	F	V	E	R	G	G	N	K	L	P	H	P	L	T	L	F
W	I	F	C	I	I	I	A	I	I	S	A	I	A	A	A	S	G	A	S	V	T	Y	E	G	F	D	K	K	E
G	I	I	K	E	T	T	L	T	I	Q	S	L	L	N	A	D	G	I	R	Y	I	F	S	S	M	V	K	N	F
T	G	F	A	P	L	G	T	V	L	V	A	L	I	G	I	G	V	A	E	G	S	G	L	M	G	A	T	M	K
K	V	V	T	A	T	P	K	R	A	L	T	A	I	V	V	L	A	G	V	M	S	N	I	A	S	D	A	G	Y
V	V	L	I	P	L	G	A	V	I	F	L	S	F	G	R	H	P	I	A	G	L	A	A	A	F	A	G	V	S
G	G	F	S	A	N	L	L	L	S	T	T	D	P	L	L	S	G	I	T	T	E	A	A	R	I	L	D	P	N
Y	F	V	N	P	A	S	N	Y	Y	F	M	F	A	S	T	I	L	I	M	I	I	G	T	F	I	T	E	K	I
V	E	P	R	L	G	E	Y	K	G	E	V	L	V	D	H	N	E	L	T	A	Q	E	R	K	A	L	R	C	A
G	I	S	V	I	I	F	C	I	V	I	G	I	L	T	I	P	A	N	A	I	L	R	V	D	G	A	L	K	Q
W	T	H	D	G	L	V	P	T	L	M	M	F	F	L	V	P	G	I	V	Y	G	K	V	A	G	T	I	K	N
D	K	D	V	A	K	M	M	G	S	S	L	A	T	M	G	G	Y	L	A	L	A	F	A	A	S	Q	F	V	A
Y	F	S	Y	T	H	L	G	T	Y	V	A	V	K	G	A	D	F	L	Q	S	I	G	L	T	G	L	P	L	I
V	I	F	V	F	V	A	A	F	I	N	L	F	M	G	S	A	S	A	K	W	A	I	M	A	P	I	F	V	P
M	L	M	R	L	G	Y	T	P	E	F	T	Q	L	A	Y	R	I	G	D	S	S	T	N	I	I	T	P	L	M
T	Y	F	A	M	I	V	A	F	M	Q	K	Y	D	K	D	A	G	M	G	T	L	I	S	I	M	L	P	Y	S
I	C	F	L	I	G	W	T	I	F	L	M	I	W	F	V	T	G	L	P	I	G	V	E	G	A	I	H	L	A
G	M																												
1				5					10					15					20					25					30

92 10.34% 97.4% >tr|U7TP25|U7TP25_FUSNU Ion-translocating oxidoreductase complex subunit C OS=Fusobacterium nucleatum CTI-6 OX=1316587 GN=rnfC PE=3 SV=1

M	T	F	F	G	F	R	G	G	V	H	P	P	E	N	K	I	Q	T	E	H	L	P	I	E	K	L	E	S	P
D	E	I	F	V	P	L	L	Q	H	I	G	A	P	L	N	P	L	V	N	V	G	D	R	V	L	K	G	Q	K

				IADAEGLAVPVHSPVSGTVTKIESRVFPLT
				GKVMTVFIENDKKEEWAELSKIENWEEADK
				KALLDIIREKGIVGIGGATFPTHVKLNPPP
				NTKLDSLILNGAECEPYLNSDNRLMLENPK
				SIVEGIKIIKKILNVSDVYVGIEDNKPEAI
				ETMRKATEGTDIKIVPLKTKYPQGGEKQLI
				KSILDRQVPSGQLPSAVGVVVQNTGTAAAI
				YEAVVNGKPLIEKVVTVSGKAIKNPKNVKV
				AIGTPFSYILDHCGVNREEMARLVMGGPMM
				GLAQMTEDATVIKGTSGLLALTNEEMRPYK
				TKACISCSKCVSACPMGLAPLMFDRLAAAK
				EYEEMAVHNLMDCIECGSCAYICPANRPLA
				EAIKTGKAKLRAKKK
				1 5 10 15 20 25 30
93	6.27%	98.9%	>tr|Q8RGG4|Q8RGG4_FUSNN Aminotransferase OS=Fusobacterium nucleatum subsp. nucleatum (strain ATCC 25586 / CIP 101130 / JCM 8532 / LMG 13131) OX=190304 GN=FN0334 PE=3 SV=1	
				MLAKRYTGKKLVDNIFATSKKAKQAIVKFG
				KENVINATIGSLYNEDEKLAVYDVVESVYR
				NLPPEDLYAYATNVIGEDDYLEEVIKAVFF
				DDYKEALKELHIASIATTGGTGAISNTVKN
				YMDTGDKVLLPNWMWGTYKNIVIENGGKIE
				TYQLFNENGDFNFEDFKNKVLELAKIQKNV
				VLILNEPSHNPTGFRMTYEEWVNLLDFFKS
				IKDTNIIVIRDVAYFEYDDRGEEETKKLRK
				LLIGMPKNILFMYAFSLSKSLSIYGMRIGA
				QIAVSSDEEVIQEFKDALPFSCRTTWSNIP
				KGGMKLFATIMKNPELKANFLKEKQGYMNL
				LNERANIFLKEAKEENLKVLPYKSGFFVTV
				PVGETVDKIIEDLESKNIFVIKFDTGIRIG
				LCSVPKRKIKGLAKKIKDSINKFKN
				1 5 10 15 20 25 30
94	8.10%	99.0%	>tr|D5RC25|D5RC25_FUSN2 Glutaconate CoA-transferase subunit A OS=Fusobacterium nucleatum subsp. nucleatum (strain ATCC 23726 / VPI 4351) OX=525283 GN=gctA PE=4 SV=1	
				MSKIMSLHDAIAKYVESGDSLCFGGFTTNR
				KPYAAVYEIIRQGQTDFIGYSGPAGGDWDM
				LIGCGRIKAFINCYIANSGYTNVCRRFRDA
				VEKKHNLLLEDYSQDVIMLMLHASSLGLPY
				LPVKLMEGSDLEYKWGISAEIRKTIPKLPD
				KKLERIPNPFKEGEVIAVPVPRLDTAIIS
				VQKASINGTCSIEGDEFHDIDIAIAAKKVI
				VIAEEIVTEEEIRRDPSKNSIPQFCVDAVV
				HVPYGTHPSQLYNYYDYDADFYKMYDKVTK
				TDEDFEQFIKEWVIDVKDHEGYLAKLGLPR
				VSKLKVVPGFQYAAKLVKDGE
				1 5 10 15 20 25 30
95	7.44%	99.0%	>tr|A0A0M1VT82|A0A0M1VT82_FUSNV Uncharacterized protein OS=Fusobacterium nucleatum subsp. vincentii 4_1_13 OX=469606 GN=FSCG_00561 PE=4 SV=1	
				MGQEKMKYLENLVGKTPMLELIFDYKGEER
				RIFVKNESYNLTGSIKDRMVFYTLKKAYEK
				GEIKKGAPIVEATSGNTGIAFSAMGAILGH
				PVIIYMPDWMSEERKSLIRSLGANIVLVSR
				EEGGFLGSIEKTKEFAKNNPGTYLPSQFSN
				SYNSEAHYYGVGLEIVNEMKGLNLNIDGFV
				AGVGTGGTVMGIGQRIKENFPNAKISPLEP
				LNSPTLSTGYKVAKHRIEGISDEFIPDLVK
				LDKLDKVVSVDDGDAIIMAQKLAKCGLGVG
				ISSGANFIGALMLQNKLGKDSVIVTVFPDD
				NKKYLSTDLMREEKIKEHFLSKDITLKGIK
				SVFRVI
				1 5 10 15 20 25 30
96	8.74%	99.0%	>tr|Q8RHG1|Q8RHG1_FUSNN Uncharacterized protein OS=Fusobacterium nucleatum subsp. nucleatum (strain ATCC 25586 / CIP 101130 / JCM 8532 / LMG 13131) OX=190304 GN=FN2075 PE=4 SV=1	
				MRNNNLTMEMLETFSGGVTVDFINNDFFDE
				TNEIYPVFSQLKRKLGSNEKVASIISIKSE
				SGSSSSAIQRNSFNKLINNTQVFYNNSCYI
				INKNNYKDFYIRKVADGNIGNDKWEGFLYI
				SIRGGQQDTLESHKGNITVFNPACEFATSD
				ISLDLDLVLAYFAMKSINLYDLSNCDKSNY
				FSLIRELETCLQNLKYNNLDFQGDLLSYCK
				NHPSLKMAEGKLYDPIQVEEINIRDFKEDK
				IIDLTHNEAVNLAKYYWDHEKKCILTPARP
				TNVFWSKHLSNMMQQDFTLEEYFKREEEIV
				RKRKELLGK
				1 5 10 15 20 25 30
97	11.45%	99.0%	>tr|X7S2Z5|X7S2Z5_FUSNU Uncharacterized protein OS=Fusobacterium nucleatum 13_3C OX=1357398 GN=HMPREF2085_00805 PE=4 SV=1	
				MRIVVCIKQVPDTTEVKIDPVKGTIIRDGV
				PSIMNPDDKGGLEEALKLKDLYGAEVIVIT
				MGPPQAEAILREAYAMGADRAILITDRKFG
				GADTLATSNTIAAAIRKIEDIDLIVAGRQA
				IDGDTAQVGPQIAEHLGLPQVSYVKEMEYK
				EDSKSFVIKRATEDGYFLLELPTPGLVTVL
				AEANQPRYMNVGAIVDVFERPIETWTFDDI
				EIDPAKIGLAGSPTKVNKSFTKGVKEPGVL
				HEVDAKEAANIILEKLKEKFII
				1 5 10 15 20 25 30
98	12.77%	99.0%	>sp|Q8RHI3|RL1_FUSNN 50S ribosomal protein L1 OS=Fusobacterium nucleatum subsp. nucleatum (strain ATCC 25586 / CIP 101130 / JCM 8532 / LMG 13131) OX=190304 GN=rplA PE=3 SV=1	
				MGKHRGKKYLEVAKLVEIGKLYDIREALEL
				VQKTKTAKFTETVEVALRLGVDPRHADQQI
				RGTVVLPHGTGKTVKILAITSGENIEKALA
				AGADYAGAEEYINQIQQGWLDFDLVIATPD
				MMPKIGRLGKILGTKGLMPNPKSGTVTPDI
				AAAVSEFKKGKLAFRVDKLGSIHAPIGKVD
				FDLDKIEENFKAFMDQIIRLKPASSKGQYL
				RTVAVSLTMGPGVKMDPAIVGKIVG
				1 5 10 15 20 25 30
99	6.28%	97.9%	>tr|A0A0M1VRV2|A0A0M1VRV2_FUSNV Uncharacterized protein OS=Fusobacterium nucleatum subsp. vincentii 4_1_13 OX=469606 GN=FSCG_00073 PE=4 SV=1	
				MLKKLSILVVAVILVGCANVDNLLGKKSGG
				PIREIGGEPQIPGETKIENNIVDDGKVINK
				EANNQNIKEYLSIVKGNLKGSIKKVEDSVE
				NNYTVGLGETLIFPLDNEKAIKLTASPRNT
				NTKINLSNGKVSFRSVYQGQYILSTYVDGS
				LNRKIAIAVIAKYDFSEKEVYDVIMQDAEN
				KSKDLENAVTLYKMMFPAGRYAKEVNYLFL
				KYAYDIKNTSLMNEALAGVKTDFSSYSDSE
				KATILRVAKLTNKDIFVPAETYNTSNSELK
				SALQEYIGNKSSLDKTDKVFIEKTKKETPE
				TANEVVRDKIKAVIDGAVSTKVGSSSGSKA
				ETKGESYYEKAMKNLNSNPRVAIENFKKSL
				STEKIQDKKPEIYYNIASSYAKLGNKVEVT
				KYLRLLKQEFPNSEWAKRSEALTK
				1 5 10 15 20 25 30
100	13.27%	98.7%	>sp|Q8RIF5|RL3_FUSNN 50S ribosomal protein L3 OS=Fusobacterium nucleatum subsp. nucleatum (strain ATCC 25586 / CIP 101130 / JCM 8532 / LMG 13131) OX=190304 GN=rplC PE=3 SV=1	
				MSGILGKKIGMTQIFEDGKFVPVTVVEAGP
				NFVLQKKTEEKDGYVALQLGFDEKKEKNTT
				KPLMGIFNKAGVKPQRFVKELEVESVDGYE
				LGQEIKVDVLTEVGYVDITGTSKGKGTSGV
				MKKHGFSGNRASHGVSRNHRLGGSIGMSSW
				PGKVLKGKKMAGQHGNATVTVQNLKVVKVD
				AEHNLLLIKGAVPGAKNSYLVIKPAVKKVI
				G
				1 5 10 15 20 25 30
101	5.46%	98.8%	>tr|D5RFV7|D5RFV7_FUSN2 Metallo-beta-lactamase domain protein OS=Fusobacterium nucleatum subsp. nucleatum (strain ATCC 23726 / VPI 4351) OX=525283 GN=fprA PE=3 SV=1	
				MYCCTKINDDIIWIGINDRKTERFENYIPL
				DNGVTYNSYLIMDEKICIVDGVEEGENGNF
				LSKIEAMIGNSPVDYIIVNHVEPDHSGSIK
				NMLKIYPELKVVGNAKTIMMLKLLGLDLPD
				ERVVIVKEKDILDLGKHKLTFYLMPMVHWP
				ESMATYDITDKVLFSNDAFGSFGALDGAIF
				DDEVNTDFFTDEMRRYYSNIVGKFGAPVNA
				ILKKLSSVEISCICPSHGLIWRKYIKEIIE
				RYQKWANMEPTKEGVVIVYGSMYGNTAEMA
				EILGRELGNRGIKDVIIYDSSKTDHSYIFS
				TIWKYKGLMLGSCAHNNDIYPKMEPLLHKL
				ENYGLKNRYLGIFGNMMWSGGGVKRIKEFA
				NTLTGLEQVGEPIEIKGHVTSAERDRLIEL
				ANLMADKLIANRK

				1				5					10					15					20					25					30
102	16.03%	98.8%	>sp|Q8RIM0|RS7_FUSNN 30S ribosomal protein S7 OS=Fusobacterium nucleatum subsp. nucleatum (strain ATCC 25586 / CIP 101130 / JCM 8532 / LMG 13131) OX=190304 GN=rpsG PE=3 SV=1																														
				M	S	R	R	R	A	A	V	K	R	D	V	L	P	D	S	R	Y	S	D	K	V	V	T	K	V	I	N	S	I
				M	L	D	G	K	K	**S**	**I**	**A**	**E**	**G**	**I**	**F**	**Y**	**S**	**A**	**M**	**D**	**L**	**I**	**K**	E	K	**T**	**G**	**Q**	**E**	**G**	**Y**	**D**
				**I**	**F**	**K**	Q	A	L	E	N	I	K	P	Q	I	E	V	R	S	R	R	I	G	G	A	T	Y	Q	V	P	V	E
				V	K	A	D	R	Q	Q	T	L	A	I	R	W	L	T	T	Y	T	R	A	R	K	E	Y	G	M	I	E	K	L
				A	A	E	L	I	A	A	A	N	N	E	G	A	T	I	K	K	K	E	D	T	Y	K	M	A	E	A	N	R	A
				F	A	H	Y	R	V																								
				1				5					10					15					20					25					30
103	6.48%	96.2%	>tr|F7L194|F7L194_FUSNU Uncharacterized protein OS=Fusobacterium nucleatum subsp. animalis 11_3_2 OX=457403 GN=HMPREF0401_01615 PE=4 SV=1																														
				M	M	L	Y	Q	Y	Q	K	D	L	L	D	K	S	L	K	N	Y	I	Y	P	L	G	T	G	T	G	K	T	I
				L	S	I	H	H	F	Y	K	H	A	Q	D	K	R	L	I	I	I	A	P	A	Q	K	V	K	E	G	G	W	D
				R	**E**	**V**	**N**	**N**	**F**	**N**	**K**	Y	Y	G	T	N	I	D	Y	E	V	V	S	Y	G	R	L	K	H	V	T	G	D
				K	N	T	Y	L	I	F	D	E	C	H	Y	I	K	N	Y	K	K	S	Q	R	S	K	L	A	L	K	L	C	K
				A	C	Y	G	Y	C	L	L	S	A	T	P	A	S	N	G	Y	Q	D	L	G	N	Y	M	A	I	F	G	I	Y
				A	S	G	Y	S	Y	E	K	A	N	A	I	K	K	M	N	Y	M	G	F	Y	E	I	V	A	W	K	N	T	E
				Y	I	D	K	C	W	K	A	I	S	S	V	A	L	N	K	N	D	C	I	D	L	P	D	L	V	F	E	E	K
				Y	F	D	A	G	E	E	Y	I	T	I	K	K	D	R	V	L	G	D	E	L	Y	D	S	S	P	K	F	I	A
				G	L	R	Q	Y	A	G	F	N	E	K	L	E	Y	L	K	E	F	R	E	S	T	D	S	N	I	L	I	F	Y
				N	F	K	K	E	A	E	A	I	K	E	L	I	K	V	D	Y	E	V	S	G	S	M	S	R	I	P	N	F	E
				D	F	K	N	L	K	N	K	T	T	L	V	Q	I	Q	A	G	G	A	G	I	E	L	Q	Y	N	S	E	V	I
				F	F	S	P	T	W	S	Y	Q	D	Y	E	Q	A	I	G	R	A	Y	R	I	G	Q	K	N	K	**V**	**T**	**V**	**Y**
				**K**	**Y**	**I**	**G**	**L**	**G**	**T**	**I**	**E**	**E**	**K**	**V**	**Y**	**T**	**R**	L	D	D	K	K	D	F	V	D	K	L	L	S	L	E
				D	L	G	G	Y	E	W	N	K	K	N																			
				1				5					10					15					20					25					30
104	7.24%	97.2%	>tr|Q8RFC3|Q8RFC3_FUSNN Xylose repressor OS=Fusobacterium nucleatum subsp. nucleatum (strain ATCC 25586 / CIP 101130 / JCM 8532 / LMG 13131) OX=190304 GN=FN0790 PE=4 SV=1																														
				M	Y	K	K	F	L	S	K	L	Y	K	R	G	F	K	M	Y	Q	K	E	I	K	Q	S	N	E	N	I	V	F
				H	S	I	Y	F	T	E	N	S	F	S	I	P	D	L	T	K	I	T	N	M	T	F	P	T	I	K	R	V	L
				N	E	F	L	K	K	D	I	I	K	E	W	T	L	S	T	G	G	V	G	R	R	A	V	K	Y	K	Y	N	P
				D	F	C	Y	S	I	G	V	S	I	D	E	E	K	I	R	F	I	V	I	N	T	I	G	K	I	L	Q	S	K
				E	I	E	T	T	D	E	N	F	L	I	F	F	E	R	N	L	K	Y	F	I	E	E	I	D	P	K	Y	L	A
				K	**V**	**I**	**G**	**V**	**G**	**I**	**S**	**I**	**P**	**G**	**I**	**Y**	**N**	**K**	E	N	H	F	L	E	F	N	N	I	D	R	Y	E	S
				S	I	I	K	K	L	E	E	N	I	K	L	P	I	W	V	E	N	E	A	N	M	S	I	L	A	E	A	I	I
				G	K	H	K	D	L	A	D	F	T	V	I	S	I	N	N	K	V	T	C	S	T	F	Y	K	F	G	N	K	S
				E	D	Y	F	F	K	A	S	R	V	H	H	M	I	V	D	Y	E	N	K	K	K	V	G	D	C	I	S	F	K
				I	L	K	D	K	I	L	E	A	F	P	N	I	K	N	L	D	E	F	F	S	N	K	K	Y	K	E	S	K	T
				G	K	K	**I**	**L**	**D**	**E**	**Y**	**L**	**T**	**Y**	**M**	**G**	**I**	**I**	**L**	**K**	N	L	L	F	T	Y	N	P	K	K	L	I	I
				S	G	E	L	S	Q	Y	G	N	Y	L	L	D	D	I	L	N	I	V	Y	E	K	N	H	I	F	Y	R	G	R
				E	T	I	S	F	S	N	F	K	G	S	S	S	I	I	G	A	A	L	F	P	I	V	D	N	L	M			
				1				5					10					15					20					25					30
105	5.40%	97.5%	>sp|Q8RHD0|GAL1_FUSNN Galactokinase OS=Fusobacterium nucleatum subsp. nucleatum (strain ATCC 25586 / CIP 101130 / JCM 8532 / LMG 13131) OX=190304 GN=galK PE=3 SV=1																														
				M	L	E	N	L	I	K	D	F	K	E	I	F	K	Y	S	G	E	V	E	R	F	F	S	P	G	R	V	N	L
				I	G	E	H	T	D	Y	N	G	G	F	V	F	P	C	A	L	D	F	G	T	Y	A	V	V	K	K	R	E	D
				K	T	F	K	M	Y	S	K	N	F	E	N	L	G	I	I	E	F	N	L	D	N	L	I	Y	D	K	K	D	D
				W	A	N	Y	P	K	G	V	I	K	T	F	L	D	R	N	Y	K	I	D	S	G	F	D	V	L	F	F	G	N
				I	P	N	G	A	G	L	S	S	S	A	S	I	E	V	L	T	A	V	I	L	K	D	L	F	K	L	D	V	D
				I	I	E	M	V	K	M	C	Q	V	A	E	N	K	F	I	G	V	N	S	G	I	M	D	Q	F	A	V	G	M
				G	K	K	D	N	A	I	L	L	D	C	N	T	L	K	Y	E	Y	V	P	V	K	L	V	N	M	S	I	V	I
				A	N	T	N	K	K	R	G	L	A	D	S	K	Y	N	E	R	R	T	S	C	E	E	A	V	K	**V**	**L**	**N**	**N**
				**N**	**E**	**V**	**N**	**I**	**K**	Y	L	G	E	L	T	V	T	E	F	E	K	V	K	**H**	**Y**	**I**	**T**	**D**	**E**	**E**	**Q**	**L**	**K**
				**R**	A	T	H	A	V	T	E	N	E	R	A	K	I	A	V	E	F	L	K	K	D	D	I	A	E	F	G	K	L
				M	N	K	S	H	T	S	L	R	D	D	Y	E	V	T	G	L	E	L	D	S	L	V	E	A	A	W	E	E	K
				G	T	V	G	S	R	M	T	G	A	G	F	G	G	C	T	V	S	I	V	E	N	D	Y	V	D	S	F	I	K
				N	V	G	K	K	Y	K	E	K	T	G	L	E	A	S	F	Y	I	A	N	I	G	D	G	A	G	K	V	K	
				1				5					10					15					20					25					30
106	5.48%	98.1%	>sp|Q8R5W0|RF1_FUSNN Peptide chain release factor 1 OS=Fusobacterium nucleatum subsp. nucleatum (strain ATCC 25586 / CIP 101130 / JCM 8532 / LMG 13131) OX=190304 GN=prfA PE=3 SV=1																														
				M	I	N	L	R	G	V	K	M	F	D	K	L	E	E	V	V	A	R	Y	D	E	L	N	K	M	L	V	S	P
				E	V	L	A	D	S	K	K	M	I	E	C	N	K	**A**	**I**	**N**	**E**	**I**	**T**	**E**	**I**	**V**	**E**	**K**	Y	K	E	Y	K
				K	Y	V	D	D	I	E	F	I	K	E	S	F	K	T	E	K	D	S	D	M	K	E	M	L	N	E	E	L	K
				E	A	E	E	K	L	P	K	L	E	E	E	L	K	I	L	L	L	P	K	D	K	N	D	D	K	N	V	I	V
				E	I	R	G	G	A	G	G	D	E	A	A	L	F	A	A	D	L	F	R	M	Y	S	R	Y	A	E	R	K	K
				W	K	I	E	I	I	E	K	**Q**	**D**	**G**	**E**	**L**	**N**	**G**	**L**	**K**	E	I	A	F	T	I	I	G	L	G	A	Y	S
				R	L	K	F	E	S	G	V	H	R	V	Q	R	V	P	K	T	E	A	S	G	R	I	H	T	S	T	A	T	V
				A	V	L	P	E	V	E	D	I	Q	E	V	T	V	D	P	K	D	L	K	I	D	T	Y	R	S	G	G	A	G
				G	Q	H	V	N	M	T	D	S	A	V	R	I	T	H	L	P	T	G	I	V	V	Q	C	Q	D	E	R	S	Q
				L	K	N	R	E	K	A	M	K	H	L	L	T	K	L	Y	E	M	E	Q	E	K	Q	R	S	E	V	E	S	E
				R	R	L	Q	V	G	T	G	D	R	A	E	K	I	R	T	Y	N	F	P	D	G	R	I	T	D	H	R	I	K
				L	T	V	H	Q	L	E	A	F	L	D	G	D	I	D	E	M	I	D	A	L	I	T	F	H	Q	A	E	L	L
				S	A	S	E	Q																									
				1				5					10					15					20					25					30
107	3.92%	96.1%	>sp|Q8R635|MURE_FUSNN UDP-N-acetylmuramoyl-L-alanyl-D-glutamate--26-diaminopimelate ligase OS=Fusobacterium nucleatum subsp. nucleatum (strain ATCC 25586 / CIP 101130 / JCM 8532 / LMG 13131) OX=190304 GN=murE PE=3 SV=1																														
				M	N	I	F	S	G	I	E	Y	K	V	L	K	D	V	N	L	D	R	K	Y	D	G	I	E	Y	D	S	R	K
				I	K	E	N	Y	I	F	V	A	F	E	G	A	N	V	D	G	H	N	Y	I	D	S	A	V	K	N	G	A	T
				C	I	I	V	S	K	K	V	E	M	K	**H**	**N**	**V**	**S**	**Y**	**I**	**L**	**I**	**D**	**D**	**I**	**R**	H	K	L	G	Y	I	A
				S	N	F	Y	E	W	P	Q	R	K	L	K	I	I	G	V	T	G	T	N	G	K	T	S	S	T	Y	M	I	E
				K	L	M	G	D	I	P	I	T	R	I	G	T	I	E	Y	K	I	G	D	K	V	F	E	A	V	N	T	T	P
				E	S	L	D	L	I	K	I	F	D	K	T	L	K	K	K	I	E	Y	V	I	M	E	V	S	S	H	S	L	E
				I	G	R	V	E	V	L	D	F	D	Y	A	L	F	T	N	L	T	Q	D	H	L	D	Y	H	L	T	M	E	N
				Y	F	Q	A	K	R	K	L	F	L	K	L	K	D	I	N	N	S	V	I	N	V	D	D	E	Y	G	K	R	L
				Y	D	E	F	I	V	D	N	P	E	I	I	S	Y	G	I	E	N	G	D	L	E	G	D	Y	S	D	D	G	Y
				I	D	V	K	Y	K	**N**	**Q**	**I**	**E**	**K**	**V**	**K**	F	S	L	L	G	D	F	N	L	Y	N	T	L	G	A	I	G
				I	A	L	K	I	G	I	S	M	E	E	I	L	K	R	V	S	N	I	K	A	A	P	G	R	F	E	A	L	D
				C	G	Q	D	Y	K	V	I	V	D	Y	A	H	T	P	D	A	L	V	N	V	I	V	A	A	R	N	I	K	N
				G	S	R	I	I	T	I	F	G	C	G	G	D	R	D	R	T	K	R	P	I	M	A	K	V	A	E	D	L	S
				D	V	V	I	L	T	S	D	N	P	R	T	E	S	P	E	Q	I	F	D	D	V	K	K	G	F	I	K	S	D
				D	Y	F	F	E	P	D	R	E	K	A	I	K	L	A	I	N	M	A	E	K	N	D	I	I	L	I	T	G	K
				G	H	E	T	Y	H	I	I	G	T	K	K	W	H	F	D	D	K	E	I	A	R	R	E	I	V	R	R	K	M
				V	E	N	V	N																									
				1				5					10					15					20					25					30
108	7.75%	99.0%	>tr|Q8RGX4|Q8RGX4_FUSNN 3-oxoacyl-[acyl-carrier-protein] synthase 2 OS=Fusobacterium nucleatum subsp. nucleatum (strain ATCC 25586 / CIP 101130 / JCM 8532 / LMG 13131) OX=190304 GN=FN0151 PE=3 SV=1																														
				M	K	R	**V**	**V**	**V**	**T**	**G**	**L**	**G**	**L**	**I**	**S**	**S**	**L**	**G**	**I**	**G**	**L**	**E**	**E**	**S**	**W**	**K**	**K**	L	I	D	G	E
				T	G	I	D	L	I	T	S	Y	D	T	T	D	Q	P	V	R	I	A	G	E	V	K	G	F	E	P	T	D	Y
				G	I	E	K	K	E	V	K	K	L	A	R	**N**	**T**	**Q**	**F**	**A**	**L**	**I**	**A**	**T**	**K**	M	A	L	E	D	A	N	F
				K	I	D	E	T	N	A	D	D	V	G	V	L	V	S	A	G	V	G	G	I	E	I	M	E	E	Q	Y	K	N
				M	L	E	K	G	P	K	R	I	S	P	F	T	I	P	A	M	I	E	N	M	A	A	G	N	I	A	I	Y	Y
				G	A	K	G	P	N	K	S	I	V	T	A	C	A	S	G	T	H	S	I	G	D	G	F	D	L	I	R	H	G
				R	A	K	A	M	I	V	G	G	T	E	A	S	V	T	K	F	C	I	N	S	F	A	N	M	K	A	L	S	T
				R	N	E	T	P	K	T	A	S	R	P	F	S	K	D	R	D	G	F	V	M	G	E	G	A	G	I	L	I	L
				E	E	L	E	S	A	L	A	R	G	A	K	I	Y	A	E	M	V	G	Y	G	E	T	C	D	A	N	H	I	T
				A	P	I	E	T	G	E	G	A	T	K	A	M	R	A	A	L	K	D	A	N	I	P	L	E	D	V	T	Y	I
				N	A	H	G	T	S	T	P	T	N	D	V	V	E	T	R	A	I	K	A	L	F	G	D	K	A	K	D	L	Y
				I	S	S	T	K	G	A	T	G	H	G	L	G	A	A	G	G	I	E	G	V	I	I	A	K	A	I	A	D	G
				I	I	P	P	T	I	N	L	H	E	T	E	E	E	C	D	L	N	Y	V	P	N	K	A	I	K	T	D	V	K
				V	A	M	S	N	S	L	G	F	G	G	H	N	S	V	I	V	M	K	K	F	E	K							
				1				5					10					15					20					25					30
109	3.23%	94.0%	>tr|R9RC13|R9RC13_FUSNU Uncharacterized protein OS=Fusobacterium nucleatum subsp. animalis 4_8 OX=469607 GN=HMPREF0409_01284 PE=4 SV=1																														
				M	K	K	F	L	G	I	L	L	L	F	I	V	V	V	V	T	V	L	Y	F	A	R	D	F	L	L	K	E	Y
				L	E	R	K	M	S	K	A	N	N	A	P	V	T	I	G	S	V	D	L	D	Y	F	E	S	Y	V	T	L	K
				D	I	K	I	M	S	N	L	H	K	D	E	I	F	I	S	I	D	E	L	K	S	Y	Y	E	I	D	F	S	K
				K	V	I	T	F	D	D	T	E	I	E	G	I	A	F	F	K	D	A	N	Y	E	N	S	E	R	E	G	V	V
				T	F	E	N	K	V	T	E	A	E	E	K	T	K	R	D	K	V	L	M	E	L	K	T	L	Y	L	N	K	I
				E	E	N	H	L	N	L	N	E	I	L	S	R	N	L	N	D	K	S	H	M	S	E	L	E	K	I	K	Q	S
				I	K	N	I	K	E	S	S	E	K	**N**	**L**	**N**	**I**	**S**	**D**	**V**	**V**	**G**	**E**	**I**	**S**	**N**	**I**	**G**	**K**	S	T	K	K
				L	G	K	D	L	K	L	D	N	L	N	K	T	E	E	D	I	K	E	G	L	T	L	E	E	S	L	D	R	V
				V	R	N	F	L	D	R	N	K	L	V	L	F	D	L	D	G	Y	I	N	M	Y	L	N	L	V	Y	E	Q	K
				I	Y	N	L	S	L	K	Y	R	D	I	L	D	E	I	R	L	R	K	E	E	D	S	K	L	S	D	G	D	I
				W	E	L	F	F	N	S	I	S	V	T	S	N	V	Y	G	I	S	F	N	G	E	V	K	N	F	S	T	R	L
				S	K	N	K	G	D	T	V	F	K	L	F	G	E	K	G	N	T	I	G	E	F	K	G	F	I	N	F	N	T
				E	L	T	E	S	T	L	N	I	P	E	A	D	L	K	D	L	G	S	D	L	L	K	G	G	E	G	V	L	F
				Q	S	L	S	T	N	G	Y	H	L	A	I	N	G	S	I	H	L	K	N	M	K	L	D	I	D	K	V	I	E
				S	M	K	I	E	D	E	V	I	K	E	I	I	A	P	L	L	R	E	L	N	T	G	E	I	Y	Y	S	Y	D
				T	D	T	R	I	L	T	I	K	T	N	I	V	E	V	F	D	D	I	L	N	G	E	N	S	S	L	K	T	K
				I	R	E	K	I	K	D	D	F	L	K	K	V	A	G															
				1				5					10					15					20					25					30
110	2.89%	94.4%	>tr|D5RFT5|D5RFT5_FUSN2 DNA polymerase IIIbeta subunit OS=Fusobacterium nucleatum subsp. nucleatum (strain ATCC 23726 / VPI 4351) OX=525283 GN=dnaN PE=3 SV=1																														
				M	H	I	K	V	N	R	Q	N	F	L	S	A	I	R	I	V	E	K	S	V	K	E	N	K	I	K	P	I	L
				S	C	I	Y	A	K	V	K	G	N	K	I	Y	F	T	G	T	N	L	D	T	T	I	K	T	S	I	D	V	N
				E	V	I	R	E	G	E	V	A	F	Y	Y	S	I	I	D	E	Y	L	K	E	I	K	D	E	F	V	V	L	R
				V	E	N	G	N	I	L	F	I	E	T	E	D	S	T	T	E	Y	D	V	F	S	A	E	D	Y	P	N	T	F
				E	N	I	V	L	N	E	N	N	F	K	F	E	M	P	S	Q	E	L	V	N	I	F	E	K	V	L	F	S	A
				D	T	P	D	N	I	A	M	N	C	I	R	I	E	S	I	L	K	H	L	H	F	V	S	T	N	T	Y	R	L

				T	F	L	K	K	N	I	D	K	D	I	S	D	F	S	V	S	V	P	A	D	T	I	S	S	I	I	K	I	I
				K	G	L	D	N	E	V	I	K	I	Y	K	E	D	A	H	L	Y	F	Q	Y	K	D	T	M	I	I	T	K	L
				I	E	L	R	F	P	N	Y	A	E	I	L	S	N	I	S	Y	D	K	K	L	H	M	N	N	D	K	L	T	N
				L	L	K	R	I	L	I	F	S	R	S	N	S	E	S	K	Y	S	S	T	Y	E	F	K	H	N	E	E	N	K
				N	K	M	A	I	S	A	L	N	E	I	A	R	I	N	E	E	L	D	V	N	F	E	G	E	D	L	K	I	S
				L	N	S	K	Y	L	L	E	F	I	Q	N	I	P	K	E	K	E	L	V	L	E	F	M	Y	S	N	S	A	V
				K	V	Y	E	K	D	N	D	E	Y	I	Y	I	L	M	P	L	A	L	R	E									
				1				5					10					15					20					25					30
111	2.89%	90.8%	>tr|Q8RF69|Q8RF69_FUSNN 8-amino-7-oxononanoate synthase OS=Fusobacterium nucleatum subsp. nucleatum (strain ATCC 25586 / CIP 101130 / JCM 8532 / LMG 13131) OX=190304 GN=FN0849 PE=4 SV=1																														
				M	R	N	V	M	Q	K	E	K	I	I	Q	E	L	Q	E	L	K	N	D	N	R	F	R	T	V	K	T	N	D
				K	S	L	Y	N	F	S	S	N	D	Y	L	S	L	A	H	D	K	D	L	L	Q	K	F	Y	Q	N	Y	N	F
				D	N	Y	K	L	S	S	S	S	S	R	L	I	D	G	S	Y	L	T	V	M	R	L	E	K	K	V	E	E	I
				Y	G	K	P	C	L	V	F	N	S	G	F	D	A	N	S	S	V	I	E	T	F	F	D	K	K	S	L	I	I
				T	D	R	L	N	H	A	S	I	Y	E	G	C	I	N	S	R	A	K	I	L	R	Y	K	H	L	D	V	S	A
				L	E	K	L	L	K	K	Y	S	E	N	Y	N	D	I	L	V	V	T	E	T	V	Y	S	M	D	G	D	C	A
				E	I	K	Q	I	C	D	L	K	E	K	Y	N	F	N	L	M	V	D	E	A	H	S	Y	G	A	Y	G	Y	G
				I	A	Y	N	E	K	L	V	N	K	I	D	F	L	V	I	P	L	G	K	A	G	A	S	V	G	A	Y	V	I
				C	D	E	I	Y	K	N	Y	L	I	N	K	S	K	K	F	I	Y	S	T	A	L	P	P	V	N	N	L	W	N
				L	F	V	L	E	N	L	V	N	F	Q	D	R	I	E	K	F	Q	E	L	V	T	F	S	L	N	T	L	K	K
				L	N	L	K	T	K	S	T	S	H	I	I	S	I	I	I	G	D	N	L	N	A	V	N	L	S	N	N	L	K
				E	L	G	Y	L	A	Y	A	I	K	E	P	T	V	P	K	D	T	A	R	L	R	I	S	L	T	A	D	M	K
				K	E	D	I	E	T	F	F	K	T	L	K	A	E	M	K	K	I	G	V	I									
				1				5					10					15					20					25					30
112	3.66%	95.1%	>tr|A0A0M1VWU3|A0A0M1VWU3_FUSNV Uncharacterized protein OS=Fusobacterium nucleatum subsp. vincentii 4_1_13 OX=469606 GN=FSCG_01875 PE=4 SV=1																														
				M	N	Y	K	K	Y	L	I	L	F	L	S	I	F	I	L	I	A	C	K	K	L	P	V	D	D	V	Q	E	S
				I	N	K	K	N	Y	N	G	A	L	Y	L	M	N	E	K	F	E	K	S	V	P	N	K	E	E	L	E	R	Y
				Q	K	I	Y	N	D	G	K	K	Y	F	L	S	K	N	D	L	K	K	A	Y	R	M	D	E	L	Y	L	K	L
				P	S	N	I	R	E	K	L	P	K	I	Q	V	D	I	N	K	H	N	E	D	A	K	T	I	G	K	N	Q	Y
				E	K	A	K	S	M	K	E	N	T	F	R	E	R	V	A	K	Y	I	E	Y	S	K	V	L	C	Y	D	E	S
				I	R	D	T	I	L	T	D	R	K	K	I	E	D	K	I	E	K	T	M	D	Y	K	I	V	G	G	N	D	E
				L	L	N	S	K	I	A	E	N	Y	R	G	Y	I	E	Y	N	P	F	I	N	T	E	D	N	P	D	T	I	L
				E	I	T	T	K	L	I	E	Y	T	P	N	K	V	K	E	K	K	Y	L	N	D	F	D	E	I	Y	T	D	E
				N	G	K	E	A	T	N	T	V	K	Y	L	G	K	H	F	F	K	T	S	N	M	K	V	E	V	K	Y	K	L
				T	S	T	L	T	G	E	V	I	L	Q	G	S	K	T	L	D	Y	E	E	Y	T	Y	W	D	T	Y	T	V	I
				S	G	T	L	K	D	R	S	Q	F	Y	Q	D	G	R	E	E	T	L	S	D	K	E	R	F	F	R	E	M	A
				I	K	V	L	R	A	I	N	Q	E	L	K	K	L	P	D	Y	D	F	L	K	I	Y	I	S					
				1				5					10					15					20					25					30
113	3.39%	95.4%	>sp|Q8RFS6|SYT_FUSNN Threonine--tRNA ligase OS=Fusobacterium nucleatum subsp. nucleatum (strain ATCC 25586 / CIP 101130 / JCM 8532 / LMG 13131) OX=190304 GN=thrS PE=3 SV=1																														
				M	F	E	I	A	K	G	I	S	N	S	L	A	K	K	S	V	G	A	K	V	D	G	K	N	V	D	M	S	Y
				I	L	D	H	D	A	E	V	E	F	I	D	I	D	S	P	E	G	E	D	I	V	R	H	S	T	A	H	L	M
				A	Q	A	V	L	R	L	Y	P	D	T	K	V	T	I	G	P	V	I	E	N	G	F	Y	Y	D	F	D	P	V
				E	Q	F	T	E	E	D	L	E	K	I	E	A	E	M	K	R	I	V	K	E	N	I	K	L	E	K	Y	V	L
				P	R	D	E	A	I	E	Y	F	R	D	V	D	K	N	K	Y	K	V	E	V	V	E	G	I	P	Q	G	E	Q
				V	S	F	Y	K	Q	G	D	F	T	D	L	C	R	G	T	H	V	P	S	T	G	Y	L	K	A	F	K	L	R
				T	V	A	G	A	Y	W	R	G	N	S	K	N	K	M	L	Q	R	I	Y	G	Y	S	F	S	N	E	E	R	L
				K	H	H	L	K	L	M	E	E	A	E	K	R	D	H	R	K	L	G	K	E	L	E	L	F	F	L	S	E	Y
				G	P	G	F	P	F	F	L	P	K	G	M	I	V	R	N	V	L	I	D	L	W	R	R	E	H	E	K	A	G
				Y	Q	Q	L	E	T	P	I	M	L	N	K	E	L	W	E	I	S	G	H	W	F	N	Y	R	E	N	M	Y	T
				S	E	I	D	E	L	E	F	A	I	K	P	M	N	C	P	G	G	V	L	A	F	K	H	Q	L	H	S	Y	K
				D	L	P	A	R	L	A	E	L	G	R	V	H	R	H	E	F	S	G	A	L	H	G	L	M	R	V	R	S	F
				T	Q	D	D	S	H	I	F	M	T	P	D	Q	V	Q	D	E	I	I	G	V	V	N	L	I	D	K	F	Y	S
				K	L	F	G	F	E	Y	E	I	E	L	S	T	K	P	E	K	A	I	G	S	Q	E	I	W	D	M	A	E	A
				A	L	A	G	A	L	D	K	L	G	R	K	Y	K	I	N	P	G	D	G	A	F	Y	G	P	K	L	D	F	K
				I	K	D	A	I	G	R	M	W	Q	C	G	T	I	Q	L	D	F	N	L	P	E	R	F	D	V	T	Y	I	G
				E	D	G	E	K	H	R	P	V	M	L	H	R	V	I	Y	G	S	I	E	R	F	I	G	I	L	I	E	H	Y
				A	G	A	F	P	M	W	L	A	P	V	Q	V	K	V	L	T	L	N	D	E	C	I	P	Y	A	K	E	I	M
				N	K	L	E	E	L	G	I	R	A	E	L	D	D	R	N	E	T	I	G	Y	K	I	R	E	A	N	G	K	Y
				K	I	P	M	Q	L	I	I	G	K	N	E	V	E	N	K	E	V	N	I	R	R	F	G	S	K	D	Q	F	S
				K	L	L	D	E	F	Y	T	Y	V	V	D	E	A	T	I	K	F	D	K										
				1				5					10					15					20					25					30
114	7.04%	90.3%	>sp|Q8RH07|GRPE_FUSNN Protein GrpE OS=Fusobacterium nucleatum subsp. nucleatum (strain ATCC 25586 / CIP 101130 / JCM 8532 / LMG 13131) OX=190304 GN=grpE PE=3 SV=1																														
				M	K	D	K	E	I	K	E	E	V	L	K	E	E	I	N	K	E	V	N	E	K	K	K	C	E	C	E	E	G
				K	E	E	A	H	E	H	K	N	D	E	H	A	C	C	G	K	H	N	H	K	E	E	I	E	K	L	K	A	E
				I	E	E	W	K	N	S	F	L	R	K	Q	A	D	F	Q	N	F	T	K	R	K	E	K	E	V	D	E	L	K
				K	F	A	S	E	K	I	I	T	Q	F	L	G	S	L	D	N	F	E	R	A	I	E	S	S	S	E	S	K	D
				F	D	S	L	L	Q	G	V	E	M	I	V	R	N	L	K	D	I	M	S	S	E	D	V	E	E	I	P	T	E
				G	A	F	N	P	E	Y	H	H	A	V	G	V	E	T	S	E	D	K	K	E	D	E	I	V	K	V	L	Q	K
				G	Y	M	M	K	G	K	V	I	R	P	A	M	V	I	V	C	K	K											
				1				5					10					15					20					25					30
115	3.05%	94.9%	>tr|R9RBQ7|R9RBQ7_FUSNU Translation elongation factor G OS=Fusobacterium nucleatum subsp. animalis 4_8 OX=469607 GN=HMPREF0409_01714 PE=4 SV=1																														
				M	K	V	F	T	T	E	N	I	R	N	I	S	L	L	G	H	R	G	S	G	K	T	T	L	V	E	S	I	L
				Y	V	K	G	Y	I	K	R	K	G	D	V	E	N	G	T	T	V	S	D	F	D	K	E	E	I	R	R	I	F
				S	I	N	T	S	L	I	S	V	E	Q	N	D	V	K	L	N	F	L	D	T	P	G	Y	F	D	F	V	G	E
				V	V	S	A	L	R	V	S	A	S	A	V	L	V	L	D	A	T	A	G	V	E	V	G	T	E	K	A	W	K
				L	L	E	E	R	K	L	P	R	I	I	F	V	N	K	M	D	K	G	Y	V	N	Y	P	K	L	L	A	E	L
				K	E	K	F	G	K	K	I	A	P	F	C	I	P	I	G	E	K	E	E	F	K	G	F	V	N	V	V	D	M
				V	G	R	V	F	D	G	K	E	C	V	D	T	P	I	P	K	D	I	D	V	S	E	V	R	N	L	L	F	E
				A	I	A	E	T	D	E	V	L	M	D	K	Y	F	A	G	E	E	F	T	Q	E	E	I	V	K	G	L	H	K
				G	V	V	N	G	D	I	V	P	V	M	V	G	S	A	Q	Q	N	I	G	I	H	T	L	L	N	Y	L	E	L
				Y	M	P	C	P	T	E	L	F	S	G	Q	R	I	G	E	D	P	T	T	Q	Q	E	K	V	V	K	I	S	S
				E	N	P	F	S	A	I	V	F	K	T	L	V	D	P	F	I	G	K	I	S	F	F	K	V	N	S	G	V	I
				K	K	E	T	E	V	F	N	P	K	K	N	K	K	E	R	I	A	Q	I	I	T	M	Q	G	N	K	Q	I	E
				L	E	E	L	H	A	G	D	I	G	A	T	T	K	L	Q	F	T	Q	T	G	D	T	L	C	D	K	N	F	P
				V	V	F	N	K	I	R	F	P	K	P	N	I	Y	S	G	V	V	P	A	N	K	N	D	D	E	K	L	S	T
				A	L	Q	R	V	M	E	E	D	P	T	F	V	M	S	R	N	Y	E	T	K	Q	L	L	I	G	G	Q	G	E
				K	H	L	Y	I	I	L	C	K	I	K	N	K	F	G	V	H	A	E	L	Q	D	V	I	V	S	Y	R	E	T
				I	L	G	K	A	E	V	E	G	K	H	K	K	Q	S	G	G	A	G	Q	Y	G	D	V	F	I	R	F	E	H
				S	D	K	D	F	E	F	V	D	E	I	K	G	G	V	V	P	R	N	Y	I	P	A	V	E	K	G	L	I	E
				A	K	E	K	G	V	L	A	G	Y	P	V	I	N	F	K	A	T	L	Y	D	G	S	Y	H	P	V	D	S	N
				D	L	S	F	K	L	A	A	I	L	A	F	K	A	G	M	E	K	A	K	P	V	L	L	E	P	F	V	R	M
				E	I	R	I	P	E	E	Y	M	G	D	V	M	G	D	L	N	K	R	R	G	R	V	L	G	M	D	H	T	E
				T	G	E	Q	L	L	L	A	E	V	P	E	A	E	I	L	K	Y	S	I	D	L	R	A	L	T	Q	G	R	G
				E	F	E	Y	E	F	V	R	Y	E	E	V	P	E	N	I	S	K	R	I	I	E	E	R	N	K	D	K		
				1				5					10					15					20					25					30
116	4.95%	95.6%	>tr|Q8RFI5|Q8RFI5_FUSNN Coenzyme A biosynthesis bifunctional protein CoaBC OS=Fusobacterium nucleatum subsp. nucleatum (strain ATCC 25586 / CIP 101130 / JCM 8532 / LMG 13131) OX=190304 GN=FN0711 PE=3 SV=1																														
				M	K	N	I	L	L	G	V	T	G	G	I	A	A	F	K	S	A	S	I	V	S	L	L	K	K	K	G	Y	N
				V	K	V	V	M	T	K	N	A	T	N	I	I	G	P	L	T	L	E	T	L	S	R	N	R	I	Y	V	D	M
				W	D	T	N	P	H	Y	E	V	E	H	I	S	L	A	D	W	A	D	M	V	L	I	A	P	A	T	Y	N	I
				I	G	K	V	A	N	G	I	A	D	D	M	L	T	T	I	L	S	A	I	S	V	R	K	P	V	F	F	A	L
				A	M	N	V	N	M	Y	E	N	P	I	L	K	E	N	V	D	K	L	K	S	Y	G	Y	R	F	I	E	A	E
				E	G	L	L	A	C	N	Y	V	A	K	G	R	M	S	E	P	K	D	I	V	E	E	I	E	R	Y	N	I	Y
				S	K	I	E	N	C	D	T	T	L	K	G	K	K	L	L	I	T	S	G	R	T	K	E	N	I	D	P	I	R
				Y	L	S	N	N	S	S	G	K	M	G	Y	C	L	V	Q	A	A	V	D	L	G	A	E	V	T	L	I	S	G
				P	T	N	L	D	I	P	K	G	L	K	N	F	I	S	V	E	S	A	I	Q	M	Y	E	K	V	D	E	Y	F
				K	D	T	D	I	F	I	A	C	A	A	V	A	D	Y	R	P	K	E	Y	K	K	E	K	I	K	K	S	D	S
				D	L	T	I	E	L	V	R	N	P	D	I	L	A	E	M	G	K	K	K	D	K	Q	L	L	V	G	F	A	A
				E	T	N	D	I	K	K	N	A	L	K	K	L	E	K	K	N	L	D	I	I	V	A	N	N	A	S	T	M	G
				T	D	S	N	T	I	E	V	I	K	R	D	K	S	S	V	E	I	K	Q	K	N	K	I	E	L	A	Y	D	I
				L	L	E	V	I	L	E	L	K	K	D	K	N	E																
				1				5					10					15					20					25					30
117	8.54%	95.2%	>sp|Q8RIH3|RS5_FUSNN 30S ribosomal protein S5 OS=Fusobacterium nucleatum subsp. nucleatum (strain ATCC 25586 / CIP 101130 / JCM 8532 / LMG 13131) OX=190304 GN=rpsE PE=3 SV=1																														
				M	L	N	R	E	D	N	Q	Y	Q	E	K	L	L	K	I	S	R	V	S	K	T	T	K	G	G	R	T	I	S
				F	S	V	L	A	A	V	G	D	G	E	G	K	I	G	L	G	L	G	K	A	N	G	V	P	D	A	I	R	K
				A	I	A	A	A	K	K	N	I	V	K	I	S	L	K	N	N	T	I	P	H	E	I	A	G	K	W	G	A	T
				T	L	W	M	A	P	A	Y	E	G	T	G	V	I	A	G	S	A	S	R	E	I	L	E	L	V	G	V	H	D
				I	L	T	K	I	K	G	S	R	N	K	H	N	V	A	R	A	T	V	E	A	L	K	L	L	R	T	A	E	Q
				I	A	A	L	R	G	L	E	V	K	D	I	L	S																
				1				5					10					15					20					25					30
118	3.41%	95.8%	>tr|R9RD28|R9RD28_FUSNU Protein translocase subunit SecD OS=Fusobacterium nucleatum subsp. animalis 4_8 OX=469607 GN=secD PE=3 SV=1																														
				M	N	K	N	L	F	L	R	L	L	L	V	I	A	I	F	V	V	A	L	Y	Y	S	L	S	K	P	I	K	L
				G	L	D	L	K	G	G	A	Y	V	V	L	E	A	V	K	D	Q	N	K	N	V	K	I	D	N	N	A	M	N
				R	L	I	E	V	L	N	R	R	V	N	G	I	G	V	A	E	S	T	I	Q	K	A	G	D	N	R	V	I	I
				E	L	P	G	L	Q	N	T	E	D	A	I	K	L	I	G	K	T	A	L	M	E	F	K	L	M	N	D	D	G
				S	L	G	E	T	L	L	T	G	S	A	L	E	K	A	E	V	S	Y	D	N	L	G	R	P	Q	I	S	F	K
				M	T	T	E	G	A	Q	V	F	A	K	I	T	R	E	N	I	G	R	Q	L	A	I	T	L	D	G	V	V	Q
				T	A	P	K	I	N	T	E	I	P	N	G	N	G	V	I	T	G	D	Y	T	V	E	Q	A	K	G	T	A	A
				L	L	N	A	G	A	L	P	I	K	A	E	I	V	E	T	R	T	V	G	A	T	L	G	D	E	S	I	A	Q
				S	K	N	A	G	I	V	A	I	I	L	I	W	V	F	M	I	I	F	Y	R	L	P	G	I	I	A	D	L	A
				I	I	I	F	G	F	I	T	F	A	C	L	N	F	I	D	A	T	L	T	L	P	G	I	A	G	F	I	L	S
				L	G	M	A	V	D	A	N	V	I	I	F	E	R	I	K	E	E	L	R	F	G	N	S	I	R	N	S	I	D
				S	G	F	N	K	G	F	I	A	I	F	D	S	N	L	T	T	L	I	I	T	T	I	L	F	V	F	G	T	G
				P	I	K	G	F	A	V	T	L	A	L	G	T	L	A	S	M	F	T	A	I	T	A	T	K	V	L	L	L	T

				F	V	N	M	F	G	F	R	S	P	K	L	F	G	V	T	V	E	E	A	K									
				1				5					10					15					20					25					30
119	19.28%	89.6%	>sp|Q8RIG4|RS17_FUSNN 30S ribosomal protein S17 OS=Fusobacterium nucleatum subsp. nucleatum (strain ATCC 25586 / CIP 101130 / JCM 8532 / LMG 13131) OX=190304 GN=rpsQ PE=3 SV=1																														
				M	R	N	E	R	K	V	R	E	G	I	V	V	S	D	K	M	Q	K	T	I	V	V	A	I	E	T	M	I	L
				H	P	I	Y	K	K	R	V	K	R	T	T	K	F	K	A	H	D	E	E	N	V	A	Q	V	G	D	K	V	K
				I	M	E	T	R	R	L	S	K	D	K	N	W	R	L	V	E	I	I	E	K	A	R							
				1				5					10					15					20					25					30
120	2.39%	95.3%	>tr|R9RDP7|R9RDP7_FUSNU Thioredoxin-disulfide reductase OS=Fusobacterium nucleatum subsp. animalis 4_8 OX=469607 GN=HMPREF0409_01824 PE=4 SV=1																														
				M	E	R	I	Y	D	M	I	V	I	G	G	G	P	A	G	L	S	A	G	I	Y	G	G	R	A	K	L	D	V
				L	V	I	E	K	E	N	K	G	G	Q	I	S	L	T	S	E	V	V	N	Y	P	G	I	L	E	I	S	G	S
				E	F	M	T	Q	T	K	K	Q	A	Q	G	F	G	V	N	F	V	Q	E	E	V	V	D	M	N	F	T	Q	K
				I	K	T	V	K	T	N	K	A	E	Y	K	T	L	S	V	V	I	A	T	G	A	A	P	R	K	L	G	F	P
				G	E	Q	E	F	T	G	R	G	V	A	Y	C	A	T	C	D	G	E	F	F	T	G	M	D	I	F	V	I	G
				A	G	F	A	A	A	E	E	A	M	F	L	T	K	Y	G	K	S	V	T	I	I	A	R	E	P	D	F	T	C
				A	K	S	I	G	D	K	V	K	A	H	P	K	I	T	V	K	F	N	T	E	L	I	E	L	T	G	D	V	K
				P	T	A	A	K	F	K	N	N	V	T	G	E	I	T	E	Y	K	A	K	V	G	E	T	F	G	V	F	V	F
				V	G	Y	A	P	S	S	Q	I	F	K	G	H	I	E	I	D	G	A	G	F	I	P	T	N	E	D	L	M	T
				N	V	D	G	V	F	A	V	G	D	I	R	P	K	R	L	R	Q	V	V	T	A	V	A	D	G	A	I	A	A
				T	S	I	E	K	Y	V	H	D	L	R	D	E	L	G	I	K	K	E	E	K	E	E	E	K	T	T	S	V	T
				S	E	K	E	H	F	L	D	D	E	L	R	Q	Q	L	V	A	V	V	D	R	F	E	N	P	V	E	I	V	V
				F	K	N	P	N	N	E	E	S	L	N	I	E	N	A	V	K	D	I	A	S	I	S	P	E	K	L	K	F	S
				S	Y	N	E	G	E	N	K	E	L	E	T	K	V	K	V	T	R	T	P	T	I	A	I	L	D	K	D	G	N
				F	A	G	L	K	Y	S	S	L	P	S	G	H	E	L	N	S	F	I	L	G	L	Y	N	V	A	G	P	G	Q
				K	V	A	T	E	S	L	E	K	I	E	K	I	N	K	P	I	N	I	K	I	G	V	S	L	S	C	T	K	C
				P	K	T	V	Q	A	T	Q	R	I	A	T	L	N	K	N	V	E	M	E	M	I	N	I	F	T	F	Q	D	F
				K	N	R	Y	D	I	M	S	V	P	A	I	I	V	D	D	Q	H	I	Y	F	G	E	K	T	V	E	D	M	L
				E	I	I	N	K																									
				1				5					10					15					20					25					30
121	5.42%	85.9%	>sp|Q8RGR0|PDXA2_FUSNN Putative D-threonate 4-phosphate dehydrogenase OS=Fusobacterium nucleatum subsp. nucleatum (strain ATCC 25586 / CIP 101130 / JCM 8532 / LMG 13131) OX=190304 GN=FN0226 PE=3 SV=1																														
				M	R	T	K	I	A	I	P	M	G	D	P	A	G	V	G	P	E	I	V	V	K	T	A	V	S	K	E	I	L
				D	L	C	D	L	V	V	I	G	D	K	K	V	L	E	K	A	T	K	I	C	Q	V	D	L	K	I	H	T	I
				K	N	I	E	E	G	K	Y	E	K	G	I	L	N	V	I	D	L	E	N	V	D	M	N	T	L	E	Y	G	Q
				V	Q	G	M	C	G	K	A	A	F	E	Y	I	K	K	C	V	E	L	A	M	E	Y	K	V	D	A	I	A	T
				T	P	I	N	K	E	S	L	R	A	G	N	V	N	Y	I	G	H	T	E	I	L	G	D	L	S	N	S	R	D
				P	L	T	M	F	E	V	D	N	M	R	V	F	F	L	T	R	H	M	S	L	R	K	A	C	D	A	I	T	K
				E	R	V	L	E	Y	I	E	R	C	T	K	A	L	K	Q	L	G	V	T	G	K	M	A	V	A	G	L	N	P
				H	S	G	E	H	G	L	F	G	N	E	E	V	K	E	I	T	P	A	I	E	E	A	Q	K	L	G	Y	D	V
				V	G	P	I	G	A	D	S	V	F	H	Q	A	L	Q	G	R	Y	V	A	V	L	S	L	Y	H	D	Q	G	H
				I	A	T	K	T	Y	D	F	E	R	T	I	A	I	T	L	D	M	P	F	L	R	T	S	V	D	H	G	T	A
				F	D	I	A	G	K	G	I	V	S	A	I	S	M	I	E	A	V	K	L	A	A	K	Y	A	P	N	F	K	N
				I	K																												
				1				5					10					15					20					25					30
122	5.37%	89.6%	>tr|Q8RFY1|Q8RFY1_FUSNN Lipopolysaccharide core biosynthesis protein rfaQ OS=Fusobacterium nucleatum subsp. nucleatum (strain ATCC 25586 / CIP 101130 / JCM 8532 / LMG 13131) OX=190304 GN=FN0546 PE=4 SV=1																														
				M	E	N	H	K	R	I	L	V	I	R	L	S	S	I	G	D	I	I	L	T	T	A	V	L	G	A	F	K	E
				K	Y	P	N	Y	I	I	D	F	L	V	I	D	K	F	K	D	A	I	S	F	S	P	Y	V	D	N	L	L	I
				Y	D	K	K	K	N	D	G	L	F	N	L	I	K	F	S	K	E	L	S	K	N	D	Y	D	Y	V	F	D	L
				H	S	K	F	R	S	K	I	I	S	F	V	L	S	K	F	Y	G	V	K	A	Y	T	Y	K	K	R	A	F	W
				K	S	I	L	V	N	L	K	L	I	K	Y	K	V	D	N	T	I	I	K	N	Y	F	L	A	F	K	D	F	D
				L	E	Y	Q	G	E	K	L	N	F	S	F	E	P	N	L	K	E	K	F	K	E	Y	K	D	Y	I	A	F	A
				V	G	A	S	K	E	T	K	K	W	T	V	E	G	F	G	K	L	A	K	K	L	Y	E	I	Y	G	K	K	V
				I	L	V	G	G	K	E	D	C	E	R	C	D	T	I	E	K	I	S	E	N	S	V	I	N	L	A	G	K	L
				T	L	K	E	T	G	A	L	L	S	Q	T	R	F	L	L	T	N	D	S	G	P	F	H	I	A	R	G	V	G
				C	K	T	F	V	I	F	G	P	T	S	P	E	M	F	D	F	G	E	N	D	V	L	V	Y	N	K	I	D	C
				S	P	C	S	L	H	G	D	K	V	C	P	K	K	H	F	K	C	M	K	E	L	S	Y	E	K	V	F	K	I
				I	E	N	K	E																									
				1				5					10					15					20					25					30
123	4.39%	89.4%	>sp|Q8R622|DXR_FUSNN 1-deoxy-D-xylulose 5-phosphate reductoisomerase OS=Fusobacterium nucleatum subsp. nucleatum (strain ATCC 25586 / CIP 101130 / JCM 8532 / LMG 13131) OX=190304 GN=dxr PE=3 SV=2																														
				M	K	K	I	L	I	L	G	S	T	G	S	I	G	T	N	A	L	E	L	I	R	N	N	R	E	Q	Y	Q	V
				V	G	I	S	G	N	K	N	I	D	L	L	K	K	Q	I	E	E	F	K	P	I	S	I	Y	V	G	S	E	Q
				D	A	I	Y	L	K	K	E	Y	S	F	I	K	E	V	Y	F	G	E	N	G	L	A	E	L	S	K	N	S	D
				Y	D	I	I	L	T	A	V	S	G	A	I	G	I	D	A	T	V	E	A	I	K	R	G	K	R	I	A	L	A
				N	K	E	T	M	V	S	A	G	V	Y	I	N	K	L	L	K	E	Y	P	K	A	E	I	V	P	V	D	S	E
				H	S	A	L	F	Q	S	L	Q	G	F	K	K	E	N	L	K	K	L	I	I	T	A	S	G	G	T	F	R	G
				K	D	L	A	Y	L	E	N	V	T	V	E	Q	A	L	K	H	P	N	W	S	M	G	K	K	I	T	I	D	S
				S	T	L	V	N	K	G	L	E	V	I	E	A	H	E	L	F	N	V	D	Y	D	D	I	E	V	V	I	H	P
				Q	S	V	I	H	S	M	V	E	Y	V	D	G	S	I	I	A	Q	M	G	V	A	N	M	K	T	P	I	L	Y
				A	F	T	Y	P	E	K	E	Y	N	S	S	I	N	F	L	D	L	I	K	N	N	N	L	T	F	E	E	A	D
				R	K	V	L	K	G	I	D	L	A	Y	R	A	G	R	T	G	D	T	M	P	T	V	F	N	A	S	N	E	I
				A	V	E	L	F	M	K	K	Q	I	K	F	L	D	I	Y	R	I	I	E	E	A	M	N	S	H	Q	V	L	T
				L	N	T	D	N	A	L	N	V	I	K	E	V	D	R	E	I	R	K	K	V	R	E	Q	W	E	K			
				1				5					10					15					20					25					30
124	3.06%	88.7%	>tr|A0A0M3UX56|A0A0M3UX56_FUSNC Short-chain fatty acid transporter OS=Fusobacterium nucleatum subsp. nucleatum OX=76856 GN=RO03_10175 PE=4 SV=1																														
				M	E	S	V	K	E	R	K	G	I	F	K	R	F	T	S	M	C	V	R	V	M	E	R	W	L	P	D	P	F
				I	F	C	A	L	L	T	F	I	V	F	I	G	A	I	F	L	T	K	A	T	P	L	Q	V	V	G	F	W	A
				D	G	F	W	S	L	L	S	F	S	M	Q	M	A	L	V	L	V	T	G	H	T	L	A	S	S	R	L	F	K
				K	M	L	S	T	F	A	S	G	I	K	G	P	K	Q	A	I	L	I	V	S	I	V	S	G	I	A	C	A	L
				N	W	G	F	G	L	V	I	G	A	L	F	A	K	E	I	A	K	K	V	K	G	V	D	Y	R	L	L	I	A
				S	A	Y	T	G	F	L	V	W	H	G	G	L	S	G	S	I	P	L	Q	L	A	S	G	G	E	G	L	A	K
				Q	T	A	G	V	V	T	E	A	I	P	T	S	Q	T	L	F	S	P	M	N	I	F	I	I	V	G	L	L	I
				I	V	P	L	L	N	M	A	M	F	P	S	K	D	E	I	V	E	V	D	P	K	L	L	V	E	P	E	E	V
				V	M	D	T	S	K	M	T	P	A	E	R	V	E	N	S	S	A	V	S	I	L	L	S	I	M	G	F	V	Y
				I	G	Y	Y	L	K	T	K	G	F	A	L	N	L	N	L	V	N	F	I	F	L	F	L	G	I	L	L	H	G
				T	P	R	R	Y	L	N	A	L	A	E	A	T	K	G	A	G	G	I	L	L	Q	F	P	F	Y	A	G	I	M
				G	I	M	V	G	A	D	A	D	G	M	S	L	A	K	L	M	S	N	F	F	V	S	I	S	T	E	K	T	F
				P	V	F	S	F	I	S	A	G	V	V	N	F	F	V	P	S	G	G	G	Q	W	A	V	Q	A	P	I	V	M
				P	A	G	Q	A	I	G	V	S	A	A	K	S	A	M	A	I	A	W	G	D	A	W	T	N	M	I	Q	P	F
				W	A	L	P	A	L	G	I	A	G	L	G	A	K	D	I	M	G	Y	C	L	I	V	T	V	V	S	G	I	F
				I	C	T	G	F	L	L	F																						
				1				5					10					15					20					25					30
125	1.92%	94.2%	>tr|A0A140PTH9|A0A140PTH9_FUSNU PTS systemFru familyIIC component OS=Fusobacterium nucleatum subsp. animalis 7_1 OX=457405 GN=FSDG_01232 PE=4 SV=1																														
				M	E	I	K	D	L	L	K	K	E	L	M	I	M	D	L	K	A	S	T	K	M	E	A	I	D	E	M	V	A
				K	L	K	E	K	N	I	I	S	D	E	V	V	F	K	D	L	I	L	K	R	E	E	K	S	S	T	G	L	G
				E	G	I	A	M	P	H	A	K	T	S	V	V	N	T	P	A	V	L	F	A	R	S	N	K	G	I	D	Y	D
				A	L	D	G	E	P	V	Y	I	F	F	M	I	A	A	S	E	G	A	H	D	L	H	I	D	T	L	A	K	L
				S	K	M	L	L	N	D	D	F	T	Q	G	L	K	T	C	G	T	P	D	E	V	Y	A	L	V	D	K	Y	S
				E	K	P	Q	E	T	I	N	K	E	V	K	E	T	Q	S	T	N	K	K	I	L	A	V	T	A	C	P	T	G
				I	A	H	T	Y	M	A	E	A	A	L	K	E	A	G	E	K	L	G	V	D	V	K	V	E	T	N	G	A	D
				G	I	K	N	N	L	T	V	N	D	I	N	E	A	V	G	I	I	V	A	A	D	K	K	V	E	T	A	R	F
				N	D	R	K	V	I	V	T	S	T	A	D	A	I	K	N	A	E	T	L	I	K	R	V	L	N	N	E	A	P
				I	F	K	A	E	T	E	N	N	P	E	E	D	I	Q	E	E	T	N	S	I	G	R	I	I	Y	K	S	I	M
				S	G	V	S	N	M	L	P	F	V	I	G	G	G	I	L	L	A	L	S	F	V	V	E	R	F	M	G	E	N
				Q	L	F	K	L	L	F	N	V	G	A	G	A	F	H	F	L	I	P	I	L	A	G	F	I	A	M	S	I	A
				D	K	P	G	F	M	P	G	A	V	A	G	Y	M	A	S	Q	G	A	G	F	L	G	G	L	I	G	G	F	I
				A	G	Y	S	I	I	F	L	K	K	M	T	K	N	M	S	K	Q	F	D	G	M	K	S	M	V	I	Y	P	I
				F	S	L	V	I	T	G	V	L	M	Y	F	I	I	V	P	I	F	T	K	V	N	L	I	V	A	N	W	L	N
				N	M	G	T	V	N	A	V	V	L	G	A	I	L	G	G	M	M	S	V	D	M	G	G	P	I	N	K	A	A
				Y	T	F	S	I	G	V	F	T	D	T	G	N	G	A	F	M	A	A	V	M	A	G	G	M	V	P	P	L	A
				I	A	L	A	M	T	L	F	K	N	N	F	D	E	K	E	K	Q	S	T	I	S	N	F	I	L	G	L	S	F
				I	T	E	G	A	I	P	F	A	A	K	E	P	V	K	V	I	S	S	C	V	I	G	A	A	I	A	G	G	L
				T	Q	F	W	S	V	S	A	P	A	P	H	G	G	I	F	V	I	P	A	M	P	S	V	H	S	A	I	F	F
				I	V	S	I	A	I	G	A	I	I	S	G	V	I	F	G	I	L	R	R	N	R	R	N						
				1				5					10					15					20					25					30
126	2.40%	91.5%	>tr|F7KX65|F7KX65_FUSNU Pyridine nucleotide-disulfide oxidoreductase family protein OS=Fusobacterium nucleatum subsp. animalis 11_3_2 OX=457403 GN=HMPREF0401_00186 PE=3 SV=1																														
				M	E	K	I	Y	D	L	L	V	I	G	W	G	K	A	G	K	T	L	S	A	K	L	G	T	K	G	K	K	V
				A	I	I	E	E	N	P	K	M	Y	G	G	T	C	I	N	V	G	C	L	P	T	K	S	L	V	H	S	A	K
				I	L	A	E	V	K	K	Y	G	I	D	G	D	Y	S	F	K	N	N	F	F	K	E	A	M	K	K	K	E	E
				M	T	T	K	L	R	N	K	N	F	G	I	L	D	T	N	E	N	V	D	I	Y	N	G	R	A	S	F	I	S
				N	N	E	V	K	V	T	S	S	D	N	K	E	I	I	L	K	A	D	K	I	V	I	N	T	G	S	V	S	R
				T	L	N	I	D	G	I	N	N	K	N	I	M	T	S	E	G	I	L	E	L	K	E	L	P	K	K	L	L	I
				I	G	A	G	Y	I	G	L	E	F	A	S	Y	F	A	N	F	G	S	E	V	S	V	F	Q	F	D	D	S	F
				L	V	R	E	D	E	D	E	A	K	I	V	K	E	I	L	E	K	K	G	I	K	F	F	F	N	T	S	V	K
				K	F	E	D	L	E	N	S	V	K	A	I	C	V	Q	D	G	K	E	F	I	E	E	F	D	K	V	L	V	A
				V	G	R	K	P	N	T	D	N	L	G	L	E	N	T	S	I	Q	L	G	K	F	G	E	I	L	V	D	D	Y
				L	K	T	N	A	P	N	V	W	A	V	G	D	V	K	G	G	P	Q	F	T	Y	V	S	L	D	D	F	R	I
				V	F	P	Q	I	L	G	E	N	N	G	R	K	L	S	D	R	V	L	I	P	T	S	T	F	I	D	P	P	Y
				S	R	V	G	I	N	E	K	E	A	Q	R	L	G	I	K	Y	T	K	K	F	A	L	T	N	T	I	P	K	A
				H	V	I	N	E	I	E	G	F	T	K	I	L	I	N	E	N	D	E	I	I	G	A	S	I	C	H	Y	E	S
				H	E	M	I	N	L	L	S	L	A	I	N	Q	K	I	K	S	K	V	L	K	D	F	I	Y	T	H	P	I	F
				T	E	S	L	N	D	I	L																						
				1				5					10					15					20					25					30

127	2.53%	88.3%	>tr|R9RAW2|R9RAW2_FUSNU Amino acid carrier protein OS=Fusobacterium nucleatum subsp. animalis 4_8 OX=469607 GN=HMPREF0409_00094 PE=3 SV=1																														
				M	D	F	F	N	Y	I	I	G	Q	I	N	T	F	L	W	S	Y	V	L	I	V	L	L	L	L	S	G	L	F
				Y	T	L	R	T	G	F	A	Q	G	R	L	L	G	D	M	V	A	L	I	T	G	K	L	S	S	L	R	D	G
				E	K	K	I	A	G	Q	V	T	G	F	Q	A	F	C	I	A	V	A	S	H	V	G	T	G	N	L	A	G	V
				A	I	A	V	S	I	G	G	P	G	A	L	F	W	M	W	I	I	A	L	L	G	A	A	T	S	M	I	E	N
				T	L	A	Q	T	Y	K	V	K	D	G	K	G	G	F	R	G	G	P	S	Y	Y	M	E	K	A	L	G	Q	K
				T	L	G	Y	I	F	S	V	I	V	I	L	T	F	A	F	I	F	N	T	V	Q	A	N	T	I	A	K	A	F
				E	T	S	F	A	S	F	G	M	N	S	Y	I	A	A	I	I	L	A	A	L	T	A	L	V	I	F	G	G	L
				H	R	I	A	N	V	V	S	L	M	V	P	I	M	A	I	A	Y	V	V	V	A	V	I	V	L	A	L	N	I
				T	H	I	P	R	L	F	L	S	I	I	E	A	A	F	G	V	K	Q	A	V	G	G	A	I	G	V	A	M	L
				Q	G	I	K	R	G	L	Y	S	N	E	A	G	M	G	S	A	P	N	A	A	A	T	S	N	V	S	H	P	V
				K	Q	G	L	L	Q	A	F	G	V	F	V	D	T	I	L	I	C	S	A	T	G	F	I	V	L	L	Y	P	E
				Y	N	A	G	T	D	T	G	I	Q	L	T	Q	F	A	L	S	Y	S	V	G	A	W	G	A	H	F	I	T	L
				C	I	F	L	F	A	F	S	S	L	I	G	N	Y	Y	Y	G	E	A	N	L	E	F	L	T	K	S	K	S	S
				M	F	I	F	R	V	L	T	V	V	F	V	F	L	G	S	I	A	S	L	G	L	V	W	D	M	A	D	V	F
				M	G	I	M	A	L	M	N	I	I	V	I	A	I	L	S	P	K	A	V	A	I	I	K	D	Y	I	K	Q	R
				K	E	G	K	N	P	V	F	R	A	K	D	I	P	G	L	E	N	T	E	C	W	D	D						
				1				5					10					15					20					25					30
128	4.56%	94.2%	>sp|Q8RGH8|TRPB_FUSNN Tryptophan synthase beta chain OS=Fusobacterium nucleatum subsp. nucleatum (strain ATCC 25586 / CIP 101130 / JCM 8532 / LMG 13131) OX=190304 GN=trpB PE=3 SV=1																														
				M	T	T	E	N	K	K	G	Y	F	G	E	F	G	G	S	Y	V	P	E	V	V	Q	K	A	L	D	K	L	E
				E	A	Y	N	K	Y	K	D	D	E	E	F	L	K	E	Y	H	H	Y	L	K	N	Y	S	G	R	E	T	P	L
				Y	F	A	E	S	L	T	N	Y	L	G	G	A	K	I	Y	L	K	R	E	D	L	N	H	L	G	A	H	K	L
				N	N	V	I	G	Q	I	L	L	A	K	R	M	G	K	K	K	V	I	A	E	T	G	A	G	Q	H	G	V	A
				T	A	A	A	A	A	K	F	G	M	Q	C	D	I	Y	M	G	A	L	D	V	E	R	Q	R	L	N	V	F	R
				M	E	I	L	G	A	T	V	H	A	V	E	K	G	E	R	T	L	K	E	A	V	D	A	A	F	K	A	W	I
				N	N	I	N	D	T	F	Y	V	L	G	S	A	V	G	P	H	P	Y	P	S	M	V	K	D	F	Q	R	V	I
				S	Q	E	A	R	R	Q	I	L	E	K	E	N	R	L	P	D	M	I	I	A	C	V	G	G	G	S	N	A	I
				G	A	F	A	E	F	I	P	D	K	E	V	K	L	I	G	V	E	A	A	G	K	G	L	N	T	D	R	H	A
				A	T	L	T	L	G	T	V	D	V	L	D	G	M	K	T	Y	A	L	F	N	E	D	G	S	V	K	P	V	Y
				S	I	S	P	G	L	D	Y	P	G	I	G	P	E	H	A	F	L	R	D	S	K	R	A	E	Y	V	S	A	T
				D	D	E	A	V	N	A	L	L	L	L	T	K	K	E	G	I	I	P	A	I	E	S	S	H	A	L	A	E	V
				I	K	R	A	P	K	L	N	K	N	K	I	I	I	V	N	I	S	G	R	G	D	K	D	V	A	A	I	A	E
				Y	L	K	N	K																									
				1				5					10					15					20					25					30
129	6.61%	94.0%	>tr|D5RAH3|D5RAH3_FUSN2 Thiamine biosynthesis protein OS=Fusobacterium nucleatum subsp. nucleatum (strain ATCC 23726 / VPI 4351) OX=525283 GN=HMPREF0397_0208 PE=4 SV=1																														
				M	E	K	I	K	A	L	A	L	F	S	G	G	L	D	S	A	L	A	I	K	V	V	Q	E	Q	G	I	E	V
				I	A	L	N	F	V	S	H	F	F	G	G	K	N	E	K	A	E	S	M	A	K	Q	L	G	I	R	L	E	Y
				I	D	F	K	K	R	H	I	L	V	V	E	D	P	V	Y	G	R	G	K	N	M	N	P	C	I	D	C	H	S
				L	M	F	K	I	A	G	E	L	L	E	E	Y	G	A	S	F	V	I	S	G	E	V	L	G	Q	R	P	M	S
				Q	N	S	Q	A	L	E	K	V	K	K	L	S	G	M	E	D	L	V	L	R	P	L	S	A	K	L	L	P	P
				S	K	A	E	I	E	G	W	V	D	R	E	K	L	L	D	I	N	G	R	S	R	Q	R	Q	M	E	L	M	E
				F	Y	G	L	V	E	Y	P	S	P	G	G	G	C	L	L	T	D	P	G	Y	S	S	R	L	K	V	L	E	D
				D	G	L	L	K	D	D	H	Y	W	L	F	K	L	I	K	E	A	R	F	F	R	F	S	Q	A	R	Y	L	F
				V	G	R	N	K	E	S	N	D	K	I	D	E	F	R	K	E	K	N	L	D	F	Y	I	Q	S	S	E	V	P
				G	P	H	I	I	A	N	T	N	L	T	D	E	E	I	D	F	A	K	K	L	F	S	R	Y	S	K	V	K	G
				N	E	K	V	I	L	N	N	S	G	N	L	E	E	V	D	V	L	D	L	K	K	L	D	E	E	I	K	K	Y
				Q	Q	L																											
				1				5					10					15					20					25					30
		86.9%																															
130	3.04%		>sp|Q8REF9|Y1147_FUSNN UPF0597 protein FN1147 OS=Fusobacterium nucleatum subsp. nucleatum (strain ATCC 25586 / CIP 101130 / JCM 8532 / LMG 13131) OX=190304 GN=FN1147 PE=3 SV=2																														
				M	E	T	K	I	E	K	V	L	K	I	L	E	E	E	I	V	A	A	E	G	C	T	E	P	I	A	L	S	Y
				A	A	A	K	A	R	R	I	L	G	T	V	P	N	K	V	D	V	F	L	S	G	N	I	I	K	N	V	K	S
				V	T	I	P	N	S	E	G	M	I	G	I	E	P	A	I	A	M	G	L	I	A	G	D	D	K	K	E	L	M
				V	I	S	D	T	T	H	E	Q	V	Q	E	V	R	D	F	L	D	K	K	L	I	K	T	H	V	Y	P	G	D
				I	K	L	Y	I	R	L	E	I	S	N	G	E	N	N	V	L	L	E	I	K	H	T	H	T	N	I	T	R	I
				L	K	N	D	K	V	L	L	S	Q	I	C	N	D	G	D	F	N	S	S	L	T	D	R	K	V	L	S	V	K
				Y	I	Y	D	L	A	K	T	I	D	I	D	L	I	K	P	I	F	Q	K	V	I	R	Y	N	S	A	I	A	D
				E	G	L	K	G	K	Y	G	V	N	I	G	K	M	I	L	D	N	I	E	K	G	I	Y	G	N	D	V	R	N
				K	A	A	S	Y	A	S	A	G	S	D	A	R	M	S	G	C	A	L	P	V	M	T	T	S	G	S	G	N	Q
				G	M	T	A	S	L	P	V	I	K	F	A	A	E	K	N	L	S	E	E	E	L	I	R	G	L	F	V	S	H
				L	I	T	I	H	V	K	T	N	V	G	R	L	S	A	Y	C	G	A	I	C	A	A	S	G	V	A	A	A	L
				T	F	L	H	G	G	S	F	E	M	V	C	D	A	I	T	N	I	L	G	N	L	S	G	V	I	C	D	G	A
				K	A	S	C	A	M	K	I	S	S	G	I	Y	S	A	F	D	A	T	M	L	A	L	N	K	D	V	L	K	S
				G	D	G	I	V	G	V	D	I	E	E	T	I	R	N	V	G	E	L	A	Q	C	G	M	K	G	T	D	E	T
				I	L	G	I	M	T	K																							
				1				5					10					15					20					25					30
131	2.79%	86.7%	>tr|A0A140PMR8|A0A140PMR8_FUSNU Uncharacterized protein OS=Fusobacterium nucleatum subsp. animalis 7_1 OX=457405 GN=FSDG_00120 PE=4 SV=1																														
				M	K	T	Y	E	R	L	T	K	N	R	Q	D	F	L	E	A	L	V	R	V	V	N	K	S	F	E	E	Q	K
				E	F	K	D	W	G	N	E	D	I	R	P	V	Q	R	I	F	I	N	A	P	W	G	M	G	K	T	L	F	A
				D	A	I	K	E	Y	L	S	E	N	Y	E	E	I	N	T	L	Y	V	N	S	W	K	M	D	F	Y	D	E	P
				M	K	A	L	I	A	E	M	S	E	D	S	I	I	T	V	E	S	T	E	K	A	K	K	F	L	K	N	C	G
				K	I	F	F	G	K	I	L	K	N	F	L	L	K	R	F	N	L	N	D	K	D	I	E	E	M	K	S	F	F
				N	G	L	D	T	S	E	L	E	D	Y	K	N	Y	K	K	L	L	E	E	F	K	D	T	L	S	K	E	E	R
				P	K	I	I	I	I	D	E	L	D	R	C	R	P	D	Y	A	I	Q	L	L	E	I	I	K	H	I	F	D	V
				K	N	I	I	F	L	F	L	I	N	R	K	Q	L	E	S	I	V	S	T	I	Y	M	N	S	N	L	S	I	K
				Y	F	E	K	F	Y	D	I	E	L	N	L	P	E	A	N	Y	K	E	L	N	E	P	E	F	Q	V	V	N	S
				F	K	E	Y	K	V	D	K	N	N	Y	S	E	N	R	D	L	V	I	Q	K	I	F	L	D	M	I	F	V	V
				N	D	S	F	S	K	I	S	I	R	S	I	K	K	L	L	K	K	F	N	I	L	K	D	S	L	I	E	K	E
				K	E	Q	H	I	L	I	L	L	L	I	E	Y	F	L	K	K	E	L	N	L	K	Y	D	T	I	I	T	K	Y
				N	K	H	R	L	I	E	A	Y	F	I	K	I	N	M	D	D	T	R	K	N	L	I	L	K	V	L	E	N	G
				K	I	N	R	D	D	V	N	F	L	D	D	G	K	D	F	Y	K	N	E	E	Y	K	K	N	N	Y	K	I	T
				I	F	R	N	N	I	Y	F	Q	N	F	K	S	A	V	C	S	N	L	K	N	T	L	Y	L	S	V	D	I	E
				E	T	D	I	L	L	W	L	E	R	K	Y	N	F	I	K														
				1				5					10					15					20					25					30
132	4.27%	92.0%	>tr|Q8RFR3|Q8RFR3_FUSNN Aspartate aminotransferase OS=Fusobacterium nucleatum subsp. nucleatum (strain ATCC 25586 / CIP 101130 / JCM 8532 / LMG 13131) OX=190304 GN=FN0625 PE=4 SV=1																														
				M	E	K	E	K	F	L	K	E	Y	L	V	E	R	K	E	T	N	S	L	K	W	D	A	L	D	K	R	F	G
				N	P	D	L	I	S	M	W	V	A	D	M	E	I	K	T	P	K	E	I	V	E	A	L	K	E	R	I	E	H
				G	A	F	G	Y	S	Y	V	S	D	D	Y	Y	N	A	V	I	K	W	H	K	E	K	H	N	Y	E	I	K	K
				E	W	L	R	F	S	T	G	V	V	T	A	I	Y	W	F	I	N	I	F	T	K	V	N	D	S	V	L	I	L
				T	P	V	Y	Y	P	F	H	N	A	V	K	D	N	N	R	K	L	I	T	C	D	L	K	N	T	N	G	Y	F
				T	I	D	Y	E	E	V	E	K	K	I	V	E	N	N	V	K	L	F	I	Q	C	S	P	H	N	P	A	G	R
				V	W	K	E	E	E	L	S	K	I	L	E	I	C	K	K	H	N	V	L	V	I	S	D	E	I	H	Q	D	I
				V	M	K	G	Y	K	H	I	P	S	A	I	V	E	S	G	K	Y	A	D	N	L	I	T	I	S	A	A	S	K
				T	F	N	L	A	G	L	I	H	S	N	I	I	I	S	N	D	K	L	R	K	K	Y	D	E	E	I	K	K	I
				N	Q	T	E	C	N	I	L	G	M	L	A	T	Q	V	G	Y	E	K	G	E	Y	W	L	E	N	V	K	E	L
				I	E	D	N	F	N	Y	L	K	S	E	L	N	K	N	I	P	E	I	I	I	T	N	L	E	G	T	Y	L	V
				F	L	D	L	R	K	I	I	P	I	D	K	V	K	E	F	I	Q	D	K	C	N	L	A	I	D	F	G	E	W
				F	G	E	N	F	K	G	F	I	R	I	N	L	A	T	D	P	Q	I	V	K	K	V	V	E	N	I	I	S	E
				Y	K	K	L	R	S	D	R																						
				1				5					10					15					20					25					30
133	3.96%	93.6%	>tr|R9RBU5|R9RBU5_FUSNU Dihydroxyacetone kinaseDhaK subunit OS=Fusobacterium nucleatum subsp. animalis 4_8 OX=469607 GN=HMPREF0409_01363 PE=4 SV=1																														
				M	K	K	L	I	N	D	K	N	N	I	V	E	E	V	V	Q	G	M	I	K	A	F	P	D	K	V	S	R	V
				E	N	E	P	I	I	I	R	K	N	K	K	V	N	K	V	A	L	I	S	G	G	G	S	G	H	E	P	A	H
				A	G	Y	V	G	Y	G	M	L	D	A	A	V	C	G	E	I	F	T	S	P	G	A	D	K	V	Y	N	A	I
				K	A	V	D	A	G	K	G	V	L	L	I	I	K	N	Y	S	G	D	V	M	N	F	E	M	A	G	E	M	A
				Q	A	E	G	I	T	V	K	Q	V	V	V	D	D	D	I	A	V	E	N	S	T	Y	T	V	G	R	R	G	I
				A	G	T	I	F	V	H	K	I	L	G	A	A	A	E	K	G	Y	D	L	D	K	L	V	E	L	G	N	K	V
				V	K	N	L	K	T	M	G	M	S	L	K	P	C	T	V	F	T	T	G	K	E	S	F	E	I	A	D	D	E
				V	E	I	G	L	G	I	H	G	E	P	G	T	H	R	E	K	M	T	T	A	N	E	F	T	E	K	L	F	E
				K	V	Y	A	E	S	N	A	Q	K	G	D	R	F	A	V	L	V	N	G	L	G	E	T	T	L	I	E	L	F
				I	I	N	N	H	L	Q	D	L	L	K	G	K	E	V	E	V	A	K	T	L	V	G	N	Y	M	T	S	L	D
				M	G	G	F	S	I	T	L	L	K	L	D	K	E	I	E	E	L	L	N	A	E	E	D	T	I	A	F		
				1				5					10					15					20					25					30
134	7.60%	90.9%	>tr|Q8RIL1|Q8RIL1_FUSNN Rod shape-determining protein mreB OS=Fusobacterium nucleatum subsp. nucleatum (strain ATCC 25586 / CIP 101130 / JCM 8532 / LMG 13131) OX=190304 GN=FN1577 PE=4 SV=1																														
				M	G	L	F	N	F	R	A	N	R	S	I	G	I	D	L	G	T	A	N	T	L	V	Y	S	K	K	H	K	K
				I	V	L	N	E	P	S	V	V	A	V	E	R	E	T	K	R	V	L	A	V	G	N	E	A	K	E	M	L	G
				K	T	P	D	T	I	V	A	V	R	P	L	S	E	G	V	I	A	D	Y	D	I	T	E	A	M	I	K	Y	F
				I	K	K	I	F	G	S	Y	S	F	F	M	P	E	I	M	I	C	V	P	I	D	V	T	G	V	E	K	R	A
				V	L	E	A	A	I	S	A	G	A	K	K	A	Y	L	I	E	E	A	R	A	A	A	L	G	S	G	M	D	I
				A	V	P	E	G	N	M	I	I	D	I	G	G	G	S	T	D	V	A	I	I	S	L	G	G	T	V	V	S	K
				T	I	R	V	A	G	N	N	F	D	S	D	I	I	K	Y	V	K	K	T	Y	N	L	L	I	G	D	R	T	A
				E	E	I	K	M	K	I	G	T	A	L	P	L	E	E	E	E	T	M	E	V	K	G	R	D	L	L	M	G	L
				P	K	V	V	T	I	T	S	E	E	V	R	E	A	I	K	D	S	L	D	Q	I	L	Q	C	I	R	T	V	L
				E	K	T	P	P	E	L	A	S	D	I	V	D	K	G	M	I	M	T	G	G	G	S	L	I	R	N	F	P	E
				M	L	T	K	Y	T	N	L	K	V	T	L	A	D	N	P	L	E	S	V	V	I	G	A	G	L	A	L	D	Q
				I	D	Y	L	R	K	I	E	K	A	E	R																		
				1				5					10					15					20					25					30
135	4.99%	94.0%	>tr|A0A0M1VSD1|A0A0M1VSD1_FUSNV Coenzyme A biosynthesis bifunctional protein CoaBC OS=Fusobacterium nucleatum subsp. vincentii 4_1_13 OX=469606 GN=FSCG_00255 PE=3 SV=1																														
				M	K	N	I	L	L	G	V	T	G	G	I	A	A	F	K	S	A	S	I	V	S	L	L	K	K	K	G	Y	N
				V	K	V	V	M	T	K	N	A	T	N	I	I	G	P	L	T	L	E	T	L	S	R	N	R	I	Y	V	D	M
				W	D	T	N	P	H	Y	E	V	E	H	I	S	L	A	D	W	A	D	M	V	L	I	A	P	A	T	Y	N	I
				I	G	K	V	A	N	G	I	A	D	D	M	L	T	T	I	I	S	A	V	S	I	R	K	P	V	F	F	A	L
				A	M	N	V	N	M	Y	E	N	P	I	L	K	E	N	I	N	K	L	K	S	Y	G	Y	R	F	I	E	A	E
				E	G	L	L	A	C	N	Y	V	A	K	G	R	M	S	E	P	E	D	I	V	A	E	I	E	R	Y	S	I	Y

			S K I E N Y H T V L K G K K I L I T S G R T K E N I D P I R
			Y L S N N S S G K M G Y S L A Q A A V D L G A E V T L I S G
			P T N L R I P K R L R N F I S V E S A L E M Y E K V D E Y F
			E D I D I F I A C A A I A D Y R P K E Y K K E K I K K S D S
			D L I L E L V R N P D I L F E M G K K K D K Q L L V G F A A
			E T N D I K E N A L K K L E K K N L D I I V A N N A S T M G
			T D S N T V E I I K K D K S S V E I K Q K N K M E L A Y D I
			L S E V I S V L K K G
			1 5 10 15 20 25 30
136	2.77%	93.7%	>tr|D5RE27|D5RE27_FUSN2 Flavocytochrome c OS=Fusobacterium nucleatum subsp. nucleatum (strain ATCC 23726 / VPI 4351) OX=525283 GN=HMPREF0397_1462 PE=4 SV=1
			M E E T M R K N F F G K L F G L T L L M F T L L F S G A S A
			E V Y E G T G L G Y D K N G I I L D V E I T N N K I V D I K
			V K R A K E S D F A T P A I Q E I A K K V I A T Q S L D V D
			G I S G A S L T S E G T K E A I E E A V S K S G V T L T A V
			A V Q N T K A V E L P K E A D V V V I G A G G A G L T S A I
			A A H E K G A K V I L I E K T E L L G G N T N Y A T A G L N
			A A G T K I Q E K L G E K D S P E L F Y E D T M K G G K N K
			N N K E L V K V L A N N S S A I V D W L I E R G A D L S E L
			T S T G G Q S A K R T H R P T G G S A V G P N I I S A L S K
			T A E N E K I D I R K G T K A I A L V K G K N R I V G V K V
			K E A D G K E Y T I K A K A V I V A T G G F G A N A K M V E
			K Y N P K L K G F G S T N S P A I V G D G I V M V E K V G G
			A L V D M N E I Q T H P T V V Y K K T N M I T E A V R G E G
			A I L V N K D G K R F I D E L E T R D V V S K A I L S Q N G
			K S A F L I F D E G I R T K L K A A D G Y V K K G F A V E G
			T L E E I A A K I G T D A K T L E A T L N K Y N E A V K N K
			V D S E F N K K N L P K E L T G T K Y Y A I E I S P A V H H
			T M G G V R I N T N A E V L G K N G R P I K G L Y A A G E V
			T G G I H G A N R I G G N A V A D I T I F G K I A G E N A A
			T Y S K S V K
			1 5 10 15 20 25 30
***F. nucleatum*_130 kDa**	**overage (Percen**	**obability (Percer**	**Sequences and Coverage**
1	44.28%	99.0%	>tr|Q8R643|Q8R643_FUSNN Pyruvate-flavodoxin oxidoreductase OS=Fusobacterium nucleatum subsp. nucleatum (strain ATCC 25586 / CIP 101130 / JCM 8532 / LMG 13131) OX=190304 GN=FN1170 PE=3 SV=1
			M A K K M Q T M D G N Q A A A Y A S Y A F T E V A G I Y P I
			T P S S P M A E Y T D E W A A K G M K N I F G V P V K L V E
			M Q S E G G A A G T V H G S L Q A G A L T T T Y T A S Q G L
			L L K I P N M Y K I A G E L L P G V I H V S A R S L S A Q A
			L S I F G D H Q D I Y A A R Q T G F A M L A T N S V Q E V M
			D L A G V A H L S A L K S R V P F L H F F D G F R T S H E I
			Q K V E V M D Y E D L K K L V D W K A L E G F R K R A L N P
			E H P V T R G T A Q N D D I Y F Q A R E V Q N K F Y D A V P
			D I V A D Y M K E I S K I T G R E Y K P F N Y Y G A P D A E
			R V I I A M G S V C E A A Q E V I D Y L V E K G E K V G L I
			S V H L F R P F S A K Y F F D I L P K T A K R I S V L D R T
			K E P G S L G E P L L L D I K A L F Y N K E N A P L I V G G
			R Y G L S S K D T T P A Q I K A V F D N L A K D T P K D A F
			T V G I V D D V T H T S L E V G P A M A L A D P T T K A C L
			F Y G L G A D G T V G A N K N S I K I I G D K T D L Y A Q G
			Y F A Y D S K K S G G V T R S H L R F G K K P I R S T Y L V
			S R P T F V A C S V P A Y L N Q Y D M T S G L K E G G K F L
			L N C V W T K E E A I A N I P N N V K R D L A K N K A R L F
			I I N A T A L A Q E I G L G Q R T N T I M Q A A F F K L A E
			I I P F E E A Q Q Y M K D Y A K K S Y A K K G D E I V Q L N
			Y N A I D R G A N D I I E I E V D P A W A N L E V T P L N E
			P K E T T G C A Y C Q P D T E F V K K I V R P V N A I K G Y
			D L P V S A F L G Y E D G T F E N G T S A F E K R G V A V D
			V P I W N V D K C I Q C N Q C S Y V C P H A A I R P F L I N
			E D E L K A A P N S F A T K K A T G K G L D E L V Y R I Q V
			S A L D C V G C G S C A N V C P A N A L D M R P I A E S L E
			T H E D I N T N Y L Y N N V E Y R S N L M P L D T V K G S Q
			F S Q P L F E F H G A C P G C G E T P Y I K L I T Q L Y G D
			R M M V A N A T G C S S I Y S G S A P A T P Y T T N K N G E
			G P S W G S S L F E D N A E Y G F G M H V G V E A L R F R I
			Q H T M E E N M D K V D E D I A T L F K D W I A N R Q Y S V
			R T R E V R D I L V P K L E A L G T D F A K E I L D L K Q Y
			L V K K S Q W I I G G D G W A Y D I G Y G G L D H V L A S N
			E D I N V L V M D T E V Y S N T G G Q A S K A T P T G S V A
			K F A A A G K P V K K K D L A A I A M S Y G H I Y V A Q V S
			M G A N Q Q Q F I K A V K E A E A H Q G P S I I I A Y S P C
			I N H G I K K G M S K S Q T E M K L A T E C G Y W P I F R Y
			N P S L E K I G K N P L Q I D S K E P K W E K Y E E Y L T G
			E V R Y Q T L A K S N P E E A K N L F E T N K K D A Q K R W
			R Q Y K R M A A L D Y S E E K E A E
			1 5 10 15 20 25 30
2	56.52%	99.0%	>tr|Q47903|Q47903_FUSNC Porin OS=Fusobacterium nucleatum subsp. nucleatum OX=76856 GN=fomA PE=4 SV=1
			M K K L A L V L G S L L V V G S V A S A K E V M P A P T P A
			P K K V V E Y V E K P V I V Y R D R E V A P A W R P N G S V
			D V Q Y R W Y G N V E N R T P K K E D P A S P W L G D N V N
			A G R L Q T L T K V N F T E K Q T L E I R T R N Y H T L M N
			P K D S Q A A D D Q V R V R H F Y K F G K L G S S K I D V T
			S R L E Y K K N N G D A G R K Q A E A S V L F D F A D Y I Y
			S N N F F K A D K F G F R L G Y Q H K W A G H N S G V V G Q
			P F N K G T Q D N Y F I N F E S E Y T L P W G F S A E L N A
			Y N Y Y N V H N K K F A T Y N K G N K K S Q F Y G E I E A Y
			L Y Q H T P L Y K T N N V E L S F D F E G G Y D P Y T W H Q
			Y K V V S A K D S N K Y E V Y M L P T L Q V S Y K P T D F V
			K L Y A A A G A E Y R N W A V T A E S K A K N W R W Q P T A
			W A G M K V T F
			1 5 10 15 20 25 30
3	58.53%	99.0%	>tr|D5RDU9|D5RDU9_FUSN2 Acyl-CoA dehydrogenase, C-terminal domain protein OS=Fusobacterium nucleatum subsp. nucleatum (strain ATCC 23726 / VPI 4351) OX=525283 GN=bcd2 PE=3 SV=1
			M E F N V P K T H E L F R Q M I R E F V E K E V K P I A A E
			V D E N E R F P V E T V E K M A K I G I M G I P I P K Q Y G
			G A G G D N L M Y A M A V E E L S K A C A T T G V I V S A H
			T S L G T W P I L K F G N E K Q K Q K Y L P K M A S G E W I
			G A F G L T E P N A G T D A A G Q Q T M A V Q D P E T G E W
			I L N G A K I F I T N A G Y A H V Y V V F A M T D K S K G L
			K G I S A F I V E A N T P G F S I G K K E M K L G I R G S A
			T C E L I F E N C R I P K E N L L G D K G K G F K I A M M T
			L D G G R I G I A S Q A L G I A A G A L D E A I N Y A K E R
			K Q F G R S L A Q F Q N T Q F Q I A N L D V K V E A A R L L
			V Y K A A W R E S N N L P Y S L D A A R A K L F A A E T A M
			E V T T K A V Q I F G G Y G Y T R E Y P V E R M M R D A K I
			T E I Y E G T S E V Q R M V I A A N I I K
			1 5 10 15 20 25 30
4	39.50%	99.0%	>sp|Q8RHI7|RPOC_FUSNN DNA-directed RNA polymerase subunit beta' OS=Fusobacterium nucleatum subsp. nucleatum (strain ATCC 25586 / CIP 101130 / JCM 8532 / LMG 13131) OX=190304 GN=rpoC PE=3 SV=1
			M G I R N F E K I R I K L A S P E K I L E W S H G E V T K P
			E T I N Y R T L N P E R D G L F C E I I F G P T K D W E C S
			C G K Y K R M R Y K G L V C E K C G V E V T R A K V R R E R
			M G H I T L A S P V S H I W Y S K G S P N K M S L I I G I S
			S K E L E S V L Y F A R Y I V T S S Q E S T V E V G K I L T
			E K E Y K L L K Q L Y G N K F E A Y M G A D G I L K L L T A
			I D L E K L R D E L E N E L A E A N S A Q K R K K L V K R L
			K I V R D F I A S G N R P E W M I L T N V P V I P A E L R P
			M V Q L D G G R F A T S D L N D L Y R R V I N R N N R L K K
			L L E I R A P E I V V K N E K R M L Q E A V D A L I D N G R
			R G K P V V A Q N N R E L K S L S D M L K G K Q G R F R Q N
			L L G K R V D Y S A R S V I V V G P S L K M N Q C G I P K K
			M A L E L Y K P F I M R E L V R R E L A N N I K M A K K L V
			E E S D D K V W A V I E D V I A D H P V L L N R A P T L H R
			L S I Q A F Q P V L I E G K A I R L H P L V C S A F N A D F
			D G D Q M A V H L T L S P E S M M E A K L L M F A P N N I I
			S P S S G E P I A V P S Q D M V M G C F Y M T K E R P G E K
			G E G K L F S N I E Q V I T A Y Q N D K V G T H A L I K V R
			M N G E L I E T T P G R V L F N E I L P E I D R N Y H K T Y
			G K K E I K S L I K S L Y E A H G F T E T A E L I N R V K N
			F G Y H Y G T F A G V S V G I E D L E V P P K K K S L L N Q
			A D K E V A Q I D K D Y K S G K I I N E E R Y R K T I E V W
			S R T T E A V T D A M M K N L D E F N P V Y M M A T S G A R
			G N V S Q M R Q L A G M R G N M A D T Q G R T I E V P I K A
			N F R E G L T V L E F F M S S H G A R K G L A D T A L R T A
			D S G Y L T R R L V D I S H E V I V N E E D C H T H E G I E
			V E A L V G A N G K I I E K L S E R I N G R V L A E D L V H
			K G K K I A K R N T M I H K D L L D K I E E L G I K K V K I
			R S P L T C A L E K G V C Q K C Y G M D L S N Y N E I L L G

				E	A	V	G	V	V	A	A	Q	S	I	G	E	P	G	T	Q	L	T	M	R	T	F	H	T	G	G	V	A	G
				A	A	T	V	V	N	S	K	K	A	E	N	D	G	E	V	S	F	R	D	I	K	T	I	E	I	N	G	E	D
				V	V	V	S	Q	G	G	K	I	I	I	A	D	N	E	H	E	V	D	S	G	S	V	I	R	V	T	E	G	Q
				K	V	K	E	G	D	V	L	V	T	F	D	P	Y	H	I	P	I	I	S	S	H	D	G	K	V	Q	Y	R	H
				F	T	P	K	N	I	R	D	E	K	Y	D	V	H	E	Y	L	V	V	R	S	V	D	S	V	D	S	E	P	R
				V	H	I	L	D	K	K	N	E	K	L	A	T	Y	N	I	P	Y	G	A	Y	M	M	V	R	D	G	A	K	V
				K	K	G	D	I	I	A	K	I	I	K	L	G	E	G	T	K	D	I	T	G	G	L	P	R	V	Q	E	L	F
				E	A	R	N	P	K	G	K	A	T	L	A	E	I	D	G	R	I	E	I	L	T	T	K	K	K	Q	M	R	V
				V	N	V	R	S	L	E	N	P	E	E	F	K	E	Y	L	I	P	M	G	E	R	L	V	V	T	D	G	L	K
				I	K	A	G	D	K	I	T	E	G	A	I	S	P	Y	D	V	L	N	I	K	G	L	V	A	A	E	Q	F	I
				L	E	S	V	Q	Q	V	Y	R	D	Q	G	V	T	V	N	D	K	H	I	E	I	I	V	K	Q	M	F	R	K
				V	R	I	I	D	S	G	A	S	L	F	L	E	D	E	V	I	E	K	R	V	V	D	L	E	N	K	K	L	E
				E	Q	G	K	A	L	I	K	Y	E	P	V	I	Q	G	I	T	K	A	A	V	N	T	G	S	F	I	S	A	A
				S	F	Q	E	T	T	K	V	L	S	N	A	A	I	E	G	K	V	D	Y	L	E	G	L	K	E	N	V	I	L
				G	K	K	I	P	A	G	T	G	F	N	K	Y	K	S	I	K	V	R	Y	N	T	D	D	K	P	E	E	E	
				1				5					10					15					20					25					30
5	35.81%	99.0%	>sp|Q8RHI6|RPOB_FUSNN DNA-directed RNA polymerase subunit beta OS=Fusobacterium nucleatum subsp. nucleatum (strain ATCC 25586 / CIP 101130 / JCM 8532 / LMG 13131) OX=190304 GN=rpoB PE=3 SV=1																														
				M	Q	K	L	I	E	R	L	D	F	G	K	I	K	A	R	G	E	M	P	H	F	L	E	F	Q	L	N	S	Y
				E	D	F	L	Q	T	N	M	S	P	N	K	R	E	E	K	G	F	E	L	A	F	K	E	I	F	P	I	E	S
				S	N	G	D	V	R	L	E	Y	I	G	Y	E	L	H	E	A	E	A	P	L	N	D	E	L	E	C	K	K	R
				G	K	T	Y	S	N	S	L	K	V	R	L	R	L	I	N	K	K	M	G	N	E	I	Q	E	S	L	V	Y	F
				G	E	V	P	K	M	T	D	R	A	T	F	I	I	N	G	A	E	R	V	V	V	S	Q	L	H	R	S	P	G
				V	S	F	S	K	E	V	N	T	Q	T	G	K	D	L	F	S	G	K	I	I	P	Y	K	G	T	W	L	E	F
				E	T	D	K	N	D	F	L	S	V	K	I	D	R	K	K	K	V	L	A	T	V	F	L	K	A	V	D	F	F
				K	D	N	N	E	I	R	D	Y	F	L	E	V	K	E	L	K	L	K	A	L	Y	K	K	Y	S	K	E	P	E
				E	L	L	N	V	L	K	Q	E	L	D	G	S	I	V	K	E	D	I	L	D	E	E	T	G	E	F	I	A	E
				A	E	A	F	I	N	E	E	V	I	N	K	L	I	E	N	K	V	D	K	I	S	Y	W	Y	V	G	P	E	S
				K	L	V	A	N	T	L	M	N	D	T	T	L	T	E	D	E	A	V	V	E	V	F	K	K	L	R	P	G	D
				Q	V	T	V	D	S	A	R	S	L	I	R	Q	M	F	F	N	P	Q	R	Y	D	L	E	P	V	G	R	Y	K
				M	N	K	R	L	K	L	D	V	P	E	E	Q	I	S	L	T	K	E	D	V	L	G	T	I	K	Y	V	I	E
				L	N	N	G	E	Q	N	V	H	T	D	D	I	D	N	L	S	N	R	R	I	R	G	V	G	E	L	L	L	M
				Q	I	K	T	G	L	A	K	M	N	K	M	V	R	E	K	M	T	T	Q	D	I	E	T	V	T	P	Q	S	L
				L	N	T	R	P	L	N	A	L	I	Q	D	F	F	G	S	G	Q	L	S	Q	F	M	D	Q	S	N	P	L	A
				E	L	T	H	K	R	R	I	S	A	L	G	P	G	G	L	S	R	E	R	A	G	F	E	V	R	D	V	H	D
				S	H	Y	G	R	I	C	P	I	E	T	P	E	G	P	N	I	G	L	I	G	S	L	A	T	Y	A	K	I	N
				K	Y	G	F	I	E	T	P	Y	V	K	V	E	N	G	V	A	L	V	D	D	V	R	Y	L	A	A	D	E	E
				D	G	L	F	I	A	Q	A	D	T	K	L	D	K	N	N	K	L	Q	G	L	V	V	C	R	Y	G	H	E	I
				V	E	I	E	P	E	R	V	N	Y	M	D	V	S	P	K	Q	V	V	S	V	S	A	G	L	I	P	F	L	E
				H	D	D	A	N	R	A	L	M	G	S	N	M	Q	R	Q	A	V	P	L	L	K	S	E	A	P	F	I	G	T
				G	L	E	R	K	V	A	V	D	S	G	A	V	V	T	T	K	V	S	G	K	V	T	Y	V	D	G	K	K	I
				I	I	E	D	K	D	K	K	E	H	I	Y	R	L	L	N	Y	E	R	S	N	Q	S	M	C	L	H	Q	T	P
				L	V	D	L	G	D	K	V	K	A	G	D	I	I	A	D	G	P	A	T	K	L	G	D	L	S	L	G	R	N
				I	L	M	G	F	M	P	W	E	G	Y	N	Y	E	D	A	I	L	I	S	D	R	L	R	K	D	D	V	F	T
				S	I	H	I	E	E	Y	E	I	D	A	R	T	T	K	L	G	D	E	E	I	T	R	E	I	P	N	V	S	E
				S	A	L	R	N	L	D	E	N	G	V	I	M	I	G	S	E	V	G	P	G	D	I	L	V	G	K	T	A	P
				K	G	E	T	E	P	P	A	E	E	K	L	L	R	A	I	F	G	E	K	A	R	D	V	R	D	T	S	L	T
				M	P	H	G	S	K	G	V	V	V	D	I	L	E	L	S	R	E	N	G	D	E	L	K	A	G	V	N	K	S
				I	R	V	L	V	A	E	K	R	K	I	T	V	G	D	K	M	S	G	R	H	G	N	K	G	V	V	S	R	V
				L	P	A	E	D	M	P	F	L	E	D	G	T	H	L	D	V	V	L	N	P	L	G	V	P	S	R	M	N	I
				G	Q	V	L	E	V	H	L	G	M	A	M	R	T	L	N	G	G	T	C	I	A	T	P	V	F	D	G	A	T
				E	E	Q	V	K	D	Y	L	E	K	Q	G	Y	P	R	T	G	K	V	T	L	Y	D	G	R	T	G	E	K	F
				D	N	K	V	T	V	G	I	M	Y	M	L	K	L	H	H	L	V	E	D	K	M	H	A	R	A	I	G	P	Y
				S	L	V	T	Q	Q	P	L	G	G	K	A	Q	F	G	G	Q	R	L	G	E	M	E	V	W	A	L	E	A	Y
				G	A	S	N	I	L	Q	E	M	L	T	V	K	S	D	D	I	T	G	R	T	K	T	Y	E	A	I	I	K	G
				E	A	M	P	E	S	D	L	P	E	S	F	K	V	L	L	K	E	F	Q	A	L	A	L	D	I	E	L	C	D
				E	E	D	N	V	I	N	V	D	E	E	I	G	I	E	E	T	P	T	E	Y	S	P	Q	Y	E	I	E	M	T
				G	L	H	E	I	D	E	D	A	E	D	F	E	E																
				1				5					10					15					20					25					30
6	38.10%	99.0%	>tr|Q8RFS7|Q8RFS7_FUSNN Uncharacterized protein OS=Fusobacterium nucleatum subsp. nucleatum (strain ATCC 25586 / CIP 101130 / JCM 8532 / LMG 13131) OX=190304 GN=FN0610 PE=4 SV=1																														
				M	I	I	N	N	R	A	Y	S	E	N	A	T	A	D	S	D	E	V	V	I	D	L	N	D	N	T	M	T	A
				E	R	G	V	V	V	T	N	G	N	M	K	G	L	F	Y	R	F	Q	R	D	P	V	T	G	E	I	T	F	T
				N	N	A	L	M	N	I	S	Q	P	T	G	S	I	K	I	E	T	E	G	G	K	V	S	Q	K	D	E	K	G
				E	F	Y	N	S	F	A	Y	I	N	V	A	K	M	T	G	A	E	A	P	N	D	K	I	Y	F	G	S	P	Y
				I	K	Y	E	D	E	K	I	Y	A	K	D	A	W	V	T	T	D	F	N	I	V	N	F	Q	K	E	P	Q	K
				A	G	Y	H	I	F	S	S	D	V	I	V	E	P	D	K	Q	I	T	L	K	N	S	D	L	F	I	G	E	K
				D	V	M	P	F	T	F	P	W	F	R	A	N	I	R	S	G	S	T	V	P	L	F	I	T	I	Q	S	D	D
				D	Y	G	A	A	T	S	M	G	F	L	Y	G	N	R	R	D	K	F	R	G	G	F	A	P	K	F	A	D	K
				M	G	I	L	V	G	R	W	E	N	W	Y	R	F	D	N	V	G	E	T	R	L	N	I	D	D	W	L	I	Y
				A	K	E	K	S	E	P	K	N	T	N	E	L	P	E	Y	E	K	R	H	K	R	Y	K	V	E	L	T	H	D
				Y	D	G	E	N	G	S	F	H	F	I	S	Q	N	S	T	R	S	M	V	G	N	L	E	E	L	M	D	K	Y
				D	N	N	N	V	Y	S	S	L	G	L	K	R	F	K	F	D	K	N	I	G	F	Y	N	L	N	A	N	L	F
				N	L	G	E	A	K	D	L	S	F	T	G	K	M	S	L	V	S	D	K	K	A	Y	G	L	L	V	Y	D	K
				I	D	D	I	S	Y	G	S	T	I	D	H	D	L	Y	T	N	L	S	L	T	K	D	N	D	K	F	K	L	N
				A	R	Y	D	Y	L	Y	D	M	D	P	G	S	T	A	K	D	L	M	S	R	N	E	R	I	G	A	D	F	L
				L	K	E	N	G	L	S	I	S	Y	D	K	R	R	G	D	D	Y	R	N	F	S	F	W	E	E	D	I	N	T
				S	A	R	K	R	N	V	L	G	I	D	F	S	Y	T	P	T	T	V	A	K	Y	E	F	N	N	F	E	N	I
				K	A	S	L	G	N	Y	K	V	G	N	Y	T	F	T	P	S	V	S	Y	N	F	L	D	R	K	L	D	T	A
				K	D	T	Y	R	T	T	V	L	G	S	N	R	L	A	E	F	N	R	F	E	N	I	V	Y	N	N	T	L	E
				R	R	A	D	L	N	L	S	N	D	N	E	T	Y	R	V	G	F	G	K	T	T	S	E	I	W	S	R	E	G
				L	F	D	G	T	Y	R	K	Y	E	N	K	S	K	F	Y	E	V	Q	L	G	R	Q	N	L	P	L	G	S	I
				G	T	F	G	I	D	G	M	F	R	Q	D	K	F	D	G	S	S	D	T	T	N	L	I	N	L	K	L	G	N
				D	L	Y	L	Y	K	T	E	N	L	D	V	T	N	K	F	K	A	E	I	Q	K	Y	S	F	S	G	N	K	N
				N	E	E	V	R	L	I	T	K	S	D	Y	I	K	F	D	N	S	L	V	F	D	G	K	S	T	V	T	T	Y
				D	I	G	Y	K	N	S	K	N	P	Y	G	V	K	N	K	N	G	E	Q	F	T	T	G	L	G	I	K	F	D
				E	N	T	N	L	S	L	K	Y	T	D	D	K	R	F	T	S	K	T	K	S	G	K	N	V	N	D	L	S	M
				K	Q	Y	S	V	N	F	E	T	K	K	Y	D	L	G	F	S	N	T	D	I	D	F	V	G	D	D	F	S	T
				T	T	D	F	R	E	D	I	N	E	H	R	I	R	A	G	Y	K	F	D	N	S	K	V	S	L	S	Y	A	E
				G	K	D	K	L	K	T	D	D	G	R	Y	L	D	R	K	N	R	M	Y	S	V	A	Y	N	I	Y	G	D	V
				E	Q	D	F	I	G	A	F	K	T	Y	R	Y	G	N	N	R	I	E	D	D	I	R	N	T	D	V	Y	S	F
				S	Y	A	Y	R	D	K	R	F	E	Q	E	E	L	M	R	Y	A	T	L	E	Y	E	K	P	K	D	Q	I	T
				N	D	E	I	E	Q	I	R	A	I	L	D	R	K	N	S	F	Y	N	Q	F	E	L	T	R	I	Q	D	E	T
				F	R	I	G	N	Y	K	K	T	L	S	A	Y	V	N	F	E	K	N	K	K	R	Y	S	Q	T	G	N	L	K
				D	S	L	S	N	F	S	G	G	L	T	V	S	Y	N	R	L	G	V	G	Y	T	F	T	Q	K	A	S	W	K
				N	S	G	G	S	Y	K	W	S	K	D	T	K	E	H	E	L	S	V	Y	A	K	I	G	K	P	S	Q	G	W
				K	I	K	T	Y	A	M	F	Y	D	N	K	N	D	P	T	S	S	K	N	R	K	R	S	V	D	S	I	G	V
				E	I	G	K	E	M	G	Y	Y	E	W	A	I	S	Y	E	N	R	Y	K	T	S	S	R	D	Y	E	W	R	V
				G	V	H	F	T	L	L	T	F	P	N	N	S	L	F	G	I	G	A	K	N	R	G	G	T	T	S	T	K	P
				D	G	Y	L	L	D	R	P	S	Q	L	K	N	S	Y															
				1				5					10					15					20					25					30
7	59.64%	99.0%	>sp|Q8R603|EFTU_FUSNN Elongation factor Tu OS=Fusobacterium nucleatum subsp. nucleatum (strain ATCC 25586 / CIP 101130 / JCM 8532 / LMG 13131) OX=190304 GN=tuf PE=3 SV=1																														
				M	A	K	E	K	Y	E	R	S	K	P	H	V	N	I	G	T	I	G	H	V	D	H	G	K	T	T	T	T	A
				A	I	S	K	V	L	S	D	K	G	W	A	S	K	V	D	F	D	Q	I	D	A	A	P	E	E	K	E	R	G
				I	T	I	N	T	A	H	I	E	Y	E	T	E	K	R	H	Y	A	H	V	D	C	P	G	H	A	D	Y	V	K
				N	M	I	T	G	A	A	Q	M	D	G	A	I	L	V	V	S	A	A	D	G	P	M	P	Q	T	R	E	H	I
				L	L	S	R	Q	V	G	V	P	Y	I	V	V	Y	L	N	K	S	D	M	V	E	D	E	E	L	L	E	L	V
				E	M	E	V	R	E	L	L	T	E	Y	G	F	P	G	D	D	I	P	V	I	R	G	S	S	L	G	A	L	N
				G	E	E	K	W	V	E	K	I	L	E	L	M	E	A	V	D	N	Y	I	P	T	P	E	R	A	V	D	Q	P
				F	L	M	P	I	E	D	V	F	T	I	T	G	R	G	T	V	V	T	G	R	V	E	R	G	V	I	K	V	G
				E	E	I	E	I	V	G	I	K	P	T	T	K	T	T	C	T	G	V	E	M	F	R	K	L	L	D	Q	G	Q
				A	G	D	N	I	G	V	L	L	R	G	T	K	K	E	E	V	E	R	G	Q	V	L	A	K	P	G	S	I	H
				P	H	T	N	F	K	G	E	V	Y	V	L	T	K	D	E	G	G	R	H	T	P	F	F	T	G	Y	R	P	Q
				F	Y	F	R	T	T	D	I	T	G	A	V	T	L	P	D	G	V	E	M	V	M	P	G	D	N	I	T	M	T
				V	E	L	I	H	P	I	A	M	E	Q	G	L	R	F	A	I	R	E	G	G	R	T	V	A	S	G	V	V	S
				E	I	T	K																										
				1				5					10					15					20					25					30
8	53.30%	99.0%	>tr|Q8RGM9|Q8RGM9_FUSNN Formate acetyltransferase OS=Fusobacterium nucleatum subsp. nucleatum (strain ATCC 25586 / CIP 101130 / JCM 8532 / LMG 13131) OX=190304 GN=FN0262 PE=4 SV=1																														
				M	E	A	W	R	G	F	K	S	G	E	W	Q	N	S	I	N	V	S	D	F	I	K	H	N	Y	T	E	Y	L
				G	D	E	S	F	L	E	G	P	T	E	N	T	K	K	L	W	D	I	L	S	G	M	L	K	I	E	R	E	K
				G	I	Y	D	A	E	T	K	T	P	S	K	I	D	A	Y	G	A	G	Y	I	S	K	D	L	E	T	I	V	G
				L	Q	T	D	A	P	L	K	R	A	I	F	P	N	G	G	L	R	M	V	E	S	S	L	E	A	F	G	Y	K
				L	D	P	T	T	K	E	I	Y	E	K	Y	R	K	S	H	N	A	G	V	F	S	A	Y	T	P	A	I	K	A
				A	R	H	T	G	V	I	T	G	L	P	D	A	Y	G	R	G	R	I	I	G	D	Y	R	R	V	A	L	Y	G
				V	D	R	L	I	E	E	R	K	R	E	F	D	A	Y	D	P	E	E	M	T	E	D	V	I	R	D	R	E	E
				M	F	E	Q	L	E	A	L	K	A	L	K	R	M	A	A	A	Y	G	F	D	I	G	R	P	A	E	T	A	Q
				E	A	I	Q	W	T	Y	F	G	Y	L	G	A	I	K	D	Q	N	G	A	A	M	S	L	G	K	T	A	G	F
				L	D	V	Y	I	E	R	D	L	K	E	G	R	I	T	E	K	Q	A	Q	E	F	I	D	H	F	I	M	K	L
				R	I	V	R	F	L	R	T	P	E	Y	D	Q	L	F	S	G	D	P	V	W	V	T	E	S	I	G	G	M	N
				N	D	G	K	S	W	V	T	K	N	A	Y	R	Y	L	N	T	L	Y	N	L	G	T	A	P	E	P	N	L	T
				I	L	W	S	E	R	L	P	E	N	W	K	K	F	C	S	K	V	S	I	D	T	S	S	L	Q	Y	E	N	D
				D	I	M	R	P	Q	F	G	E	D	Y	G	I	A	C	C	V	S	P	M	A	I	G	K	Q	M	Q	F	F	G
				A	R	A	N	L	P	K	A	L	L	Y	A	I	N	G	G	K	D	E	L	K	K	D	Q	V	T	P	A	G	Q
				F	E	R	I	T	S	D	Y	L	D	F	D	E	V	W	E	K	Y	D	K	M	L	T	W	L	A	S	T	Y	I
				K	A	L	N	I	I	H	Y	M	H	D	K	Y	S	Y	E	A	L	E	M	A	L	H	S	L	D	I	K	R	T
				E	A	C	G	I	A	G	L	S	I	V	A	D	S	L	A	A	I	K	Y	G	K	V	R	V	I	R	D	E	A
				G	D	A	V	D	Y	V	V	E	Q	P	Y	V	P	F	G	N	N	D	D	R	T	D	E	L	A	V	K	V	V
				R	T	F	M	N	K	I	R	S	H	K	M	Y	R	D	A	E	P	T	Q	S	V	L	T	I	T	S	N	V	V
				Y	G	K	K	T	G	N	T	P	D	G	R	R	A	G	A	P	F	G	P	G	A	N	P	M	H	G	R	D	T

K	G	A	V	A	S	L	A	S	V	A	K	L	P	F	E	D	A	N	D	G	I	S	Y	T	F	A	I	T	P
E	T	L	G	K	T	D	D	E	K	K	N	N	L	V	G	L	L	D	G	Y	F	K	Q	T	G	H	H	L	N
V	N	V	F	G	R	E	L	L	E	D	A	M	E	H	P	E	N	Y	P	Q	L	T	I	R	V	S	G	Y	A
V	N	F	I	K	L	T	R	E	Q	Q	L	D	V	I	N	R	T	I	S	N	K	M							
1				5					10					15					20					25					30

9 38.82% 99.0% >tr|D5RE87|D5RE87_FUSN2 Glutamate dehydrogenase OS=Fusobacterium nucleatum subsp. nucleatum (strain ATCC 23726 / VPI 4351) OX=525283 GN=gluD PE=3 SV=1

M	S	K	E	T	L	N	P	L	E	S	G	Q	K	Q	V	K	K	A	C	D	A	L	G	L	D	P	A	V	Y
E	L	L	K	E	P	Q	R	I	I	E	I	T	I	P	V	K	M	D	D	G	S	I	K	T	F	K	G	Y	R
A	A	H	N	D	A	V	G	P	F	K	G	G	I	R	F	H	Q	N	V	N	S	D	E	V	K	A	L	S	L
W	M	S	I	K	C	Q	V	T	G	I	P	Y	G	G	G	K	G	G	I	T	V	D	P	S	E	L	S	Q	R
E	L	E	Q	L	S	R	G	W	V	R	G	M	W	K	Y	L	G	E	K	V	D	V	P	A	P	D	V	N	T
N	G	Q	I	M	A	W	M	Q	D	E	Y	N	K	L	S	G	E	Q	T	I	G	V	F	T	G	K	P	L	S
Y	G	G	S	Q	G	R	N	E	A	T	G	F	G	V	A	V	T	M	R	E	A	F	K	A	L	G	K	N	L
K	G	A	T	V	A	V	Q	G	F	G	N	V	G	K	F	T	V	K	N	I	M	K	L	G	G	K	V	V	A
V	A	E	F	E	K	G	K	G	A	Y	A	I	Y	K	D	S	G	F	T	F	E	E	L	E	A	A	K	A	A
G	S	L	T	K	V	A	G	A	K	E	L	S	M	D	E	F	W	A	L	N	V	E	A	I	A	P	C	A	L
E	N	A	I	T	N	H	E	A	E	L	I	K	A	G	I	V	C	E	G	A	N	G	P	I	T	P	E	A	D
E	V	L	Y	K	K	G	I	V	V	T	P	D	V	L	T	N	A	G	G	V	T	V	S	Y	F	E	W	V	Q
N	I	Y	G	Y	Y	W	T	E	K	E	V	E	E	K	E	E	R	A	M	V	D	A	F	T	P	I	W	A	L
K	K	E	F	D	G	K	G	Q	P	I	S	F	R	Q	A	T	Y	M	K	S	I	K	R	I	A	E	A	M	K
I	R	G	W	Y																									
1				5					10					15					20					25					30

10 41.69% 99.0% >tr|Q8RFC6|Q8RFC6_FUSNN Electron transfer flavoprotein alpha-subunit OS=Fusobacterium nucleatum subsp. nucleatum (strain ATCC 25586 / CIP 101130 / JCM 8532 / LMG 13131) OX=190304 GN=FN0785 PE=4 SV=1

M	N	L	N	D	Y	K	G	I	L	V	F	A	E	Q	R	D	G	I	L	Q	N	V	G	L	E	L	L	G	K
A	T	E	L	A	Y	E	I	N	K	Q	I	A	L	K	D	A	G	D	E	L	A	D	F	A	G	K	K	E	A
A	I	K	S	V	D	V	A	A	A	T	L	E	E	D	D	Q	E	L	K	N	K	V	A	D	I	K	K	N	N
P	D	A	A	K	V	T	A	L	L	V	G	H	N	I	K	N	L	A	Q	D	L	I	K	A	G	A	D	K	V
L	V	V	D	K	P	E	L	E	V	Y	D	T	E	A	Y	A	Q	V	L	T	A	T	I	N	D	Q	K	P	E
I	V	L	F	G	A	T	T	L	G	R	D	L	A	P	R	V	S	S	R	I	A	T	G	L	T	A	D	C	T
K	L	E	L	L	K	D	K	E	R	Q	L	G	M	T	R	P	A	F	G	G	N	L	M	A	T	I	V	S	P
D	H	R	P	Q	M	A	T	V	R	P	G	V	M	K	K	L	P	K	S	D	D	R	T	G	E	V	V	E	F
P	V	T	L	D	T	S	K	M	K	V	K	L	L	K	V	V	K	E	G	G	N	K	V	D	I	S	E	A	K
I	L	V	S	G	G	R	G	V	G	S	K	Q	N	F	E	L	L	E	D	L	A	T	E	I	G	G	I	V	S
S	S	R	A	Q	V	D	A	G	N	M	P	H	D	R	Q	V	G	Q	T	G	K	T	V	R	P	E	V	Y	F
A	C	G	I	S	G	A	I	Q	H	V	A	G	M	E	E	S	E	F	I	I	A	I	N	K	D	R	F	A	P
I	F	S	V	A	D	L	G	I	V	G	D	L	H	K	I	L	P	I	L	T	E	E	I	K	K	Y	K	A	T
K																													
1				5					10					15					20					25					30

11 26.02% 99.0% >tr|Q8REU9|Q8REU9_FUSNN Phosphoribosylformylglycinamidine synthase OS=Fusobacterium nucleatum subsp. nucleatum (strain ATCC 25586 / CIP 101130 / JCM 8532 / LMG 13131) OX=190304 GN=FN0990 PE=4 SV=1

M	E	E	R	M	S	N	L	R	F	F	V	E	K	K	K	G	F	D	L	D	A	K	R	L	E	K	Q	F	R
E	E	L	G	V	N	V	R	D	L	R	L	I	N	C	Y	D	I	F	N	L	N	D	N	K	E	D	I	E	K
I	K	K	M	I	L	S	E	P	V	T	D	T	I	T	T	E	L	D	L	K	G	K	K	Y	F	A	V	E	F
L	P	G	Q	F	D	Q	R	A	D	S	A	L	Q	C	I	D	I	V	S	S	E	K	Q	N	A	D	I	L	T
S	R	I	I	I	L	N	N	E	L	S	D	E	E	L	N	K	I	K	K	F	Y	I	N	P	I	E	M	R	E
K	D	L	S	V	L	K	K	E	E	I	L	F	N	S	E	V	I	T	Y	D	N	F	I	L	F	D	D	V	D
L	E	K	I	R	T	D	L	G	L	S	M	S	F	E	D	L	K	F	I	Q	G	H	Y	K	E	I	G	R	N
P	T	E	T	E	I	K	V	L	D	T	Y	W	S	D	H	C	R	H	T	T	F	E	T	K	I	N	K	V	T
F	P	D	S	E	F	G	K	Q	M	E	K	E	F	N	D	Y	L	K	L	K	E	D	V	S	K	K	R	D	V
S	L	M	D	M	A	T	I	V	A	K	Y	L	K	K	E	G	K	L	D	N	L	E	V	S	E	E	N	N	A
C	S	V	Y	V	D	V	E	V	E	D	F	E	G	K	K	A	I	E	K	W	L	L	M	F	K	N	E	T	H
N	H	P	T	E	I	E	P	F	G	G	A	S	T	C	L	G	G	A	I	R	D	P	L	S	G	R	A	Y	V
Y	Q	A	I	R	V	T	G	S	G	N	P	L	E	T	V	E	E	T	L	K	G	K	L	F	Q	K	K	I	T
T	G	A	A	S	G	Y	A	S	Y	G	N	Q	I	G	I	A	T	S	L	V	S	E	I	Y	H	D	G	Y	K
A	K	R	M	E	V	G	A	V	V	A	A	A	P	V	E	N	V	V	R	K	S	P	V	P	T	D	S	I	I
I	I	G	G	K	T	G	R	D	G	C	G	G	A	T	G	S	S	K	E	H	N	D	K	S	L	L	L	C	G
A	E	V	Q	K	G	N	A	P	E	E	R	K	I	Q	R	L	F	R	N	P	N	A	T	K	L	I	K	K	C
N	D	F	G	A	G	G	V	S	V	A	I	G	E	L	A	D	G	V	E	V	N	L	D	L	V	P	V	K	Y
E	G	L	N	G	T	E	L	A	I	S	E	S	Q	E	R	M	A	V	I	V	S	K	E	D	T	E	K	F	L
K	Y	V	D	E	E	N	L	L	G	T	V	V	G	Y	V	T	D	K	N	R	L	T	L	N	W	K	G	K	A
I	V	D	I	S	R	D	F	L	N	T	N	G	V	Q	Q	N	I	D	I	E	V	R	D	Y	E	N	E	N	V
F	E	K	F	K	T	S	D	S	S	L	E	K	K	W	L	H	N	I	K	K	L	N	V	V	S	Q	K	G	L
V	E	M	F	D	S	S	V	G	A	G	T	I	L	A	P	F	G	G	K	Y	Q	M	T	P	T	D	V	S	I
M	K	F	P	V	L	N	K	N	T	N	T	A	S	A	I	T	W	G	F	N	P	Y	I	S	E	W	S	T	Y
H	G	A	I	Y	A	V	V	E	S	L	A	K	L	V	A	A	G	V	D	Y	K	T	A	R	L	S	F	Q	E
Y	F	E	K	L	G	K	D	S	Y	K	W	S	K	P	F	L	A	L	L	G	A	M	K	V	Q	K	D	F	D
V	A	A	I	G	G	K	D	S	M	S	G	T	F	N	D	I	S	V	P	P	T	L	I	S	F	A	V	S	T
V	N	V	T	D	V	I	S	T	E	F	K	K	A	K	N	K	I	Y	L	V	E	N	K	I	N	E	K	D	F
L	F	D	S	Q	E	L	K	E	N	F	H	F	I	L	K	N	I	K	D	K	K	I	V	S	A	M	V	V	K
I	G	G	L	A	E	V	L	S	K	M	S	F	G	N	R	L	G	F	E	I	N	N	K	N	V	D	L	F	S
L	K	P	A	S	I	L	I	E	T	T	E	E	L	S	Y	K	N	A	I	Y	L	G	E	V	T	D	K	F	E
G	K	V	N	G	E	N	I	N	L	E	E	V	E	A	T	W	L	N	K	L	K	P	I	F	P	Y	K	L	E
E	K	I	E	T	Y	D	I	K	N	K	I	S	E	K	K	I	Y	K	S	S	I	T	I	A	K	P	R	V	V
I	A	A	F	P	G	T	N	S	E	Y	D	M	Y	N	R	F	N	E	N	G	G	E	A	K	I	T	L	L	R
N	L	T	Q	K	H	L	T	E	S	V	D	E	M	C	K	D	L	R	N	S	Q	I	F	V	L	P	G	G	F
S	A	G	D	E	P	D	G	S	G	K	F	M	A	A	V	L	Q	N	P	K	L	M	N	E	I	K	A	F	L
D	R	D	G	L	I	L	G	V	C	N	G	F	Q	A	L	V	K	S	G	L	L	P	Y	G	E	I	G	N	V
H	E	N	S	P	T	L	T	F	N	K	I	G	R	H	I	S	Q	I	V	K	T	K	I	V	T	N	N	S	P
W	L	S	S	F	E	I	G	E	T	F	D	I	P	V	S	H	G	E	G	R	F	Y	A	S	D	G	V	L	K
Q	L	F	E	N	G	Q	I	A	T	Q	Y	V	D	F	D	L	E	A	T	N	E	F	R	F	N	P	N	G	S
S	F	A	I	E	G	I	I	S	P	D	G	K	I	F	G	K	M	G	H	S	E	R	Y	S	K	D	T	F	K
N	I	D	G	N	K	N	Q	N	L	I	L	N	G	I	K	Y	F	K											
1				5					10					15					20					25					30

12 61.94% 99.0% >tr|D5RE94|D5RE94_FUSN2 Acetyl-CoA C-acetyltransferase OS=Fusobacterium nucleatum subsp. nucleatum (strain ATCC 23726 / VPI 4351) OX=525283 GN=atoB PE=3 SV=1

M	S	K	V	Y	V	V	A	A	K	R	S	A	I	G	S	F	L	G	T	L	S	P	L	K	P	G	D	L	G
A	Q	I	V	K	N	I	L	E	E	T	K	V	D	P	A	N	I	D	E	V	I	V	G	N	V	L	S	A	G
Q	A	Q	G	V	G	R	Q	V	A	I	R	A	G	I	P	Y	E	V	P	A	Y	S	V	N	I	I	C	G	S
G	M	K	S	V	I	T	A	F	S	N	I	K	A	G	E	A	D	L	V	I	A	G	G	T	E	S	M	S	G
A	G	F	I	L	P	G	A	I	R	G	G	H	K	M	A	D	L	T	M	K	D	H	M	I	L	D	A	L	T
D	A	Y	H	N	I	H	M	G	I	T	A	E	N	I	A	E	R	Y	G	I	T	R	E	E	Q	D	A	F	A
L	D	S	Q	L	K	A	I	A	A	V	D	S	G	R	F	K	D	E	I	A	P	V	V	I	P	N	K	K	G
D	I	I	F	D	T	D	E	Y	P	N	R	K	T	D	A	E	K	L	A	K	L	K	P	A	F	K	K	D	G
S	V	T	A	G	N	A	S	G	L	N	D	G	A	S	F	L	M	L	A	S	E	E	A	V	K	K	Y	N	L
K	P	L	V	E	I	V	A	T	G	T	G	G	V	D	P	L	V	M	G	M	G	P	V	P	A	I	R	K	A
F	N	K	T	D	L	K	L	K	D	M	E	L	I	E	L	N	E	A	F	A	A	Q	S	L	G	V	I	K	E
L	C	K	E	H	G	V	T	P	E	W	I	K	E	R	T	N	V	N	G	G	A	I	A	L	G	H	P	V	G
A	S	G	N	R	I	T	V	T	L	I	Y	E	M	K	K	R	G	V	E	Y	G	L	A	S	L	C	I	G	G
G	M	G	T	A	L	I	L	K	N	V	K																		
1				5					10					15					20					25					30

13 33.44% 99.0% >sp|Q8RG65|GLMS_FUSNN Glutamine--fructose-6-phosphate aminotransferase [isomerizing] OS=Fusobacterium nucleatum subsp. nucleatum (strain ATCC 25586 / CIP 101130 / JCM 8532 / LMG 13131) OX=190304 GN=glmS PE=3 SV=3

M	C	G	I	I	G	Y	S	G	S	K	A	N	A	V	E	V	L	L	E	G	L	E	K	V	E	Y	R	G	Y
D	S	A	G	I	A	F	V	T	D	S	G	I	Q	I	E	K	K	E	G	K	L	E	N	L	K	N	H	M	K
N	F	E	V	L	S	C	T	G	I	G	H	T	R	W	A	T	H	G	I	P	T	D	R	N	A	H	P	H	Y
S	E	S	K	D	V	A	L	I	H	N	G	I	I	E	N	Y	V	E	I	K	K	E	L	L	E	Q	G	V	K
F	S	S	D	T	D	T	E	V	V	A	Q	L	F	S	K	L	Y	D	G	D	L	Y	S	T	L	K	K	V	L
K	R	I	R	G	T	Y	A	F	A	I	I	H	K	D	F	P	D	K	M	I	C	C	R	N	H	S	P	L	I
V	G	L	G	E	H	Q	N	F	I	A	S	D	V	S	A	I	L	K	Y	T	R	D	I	I	Y	L	E	D	G
D	V	V	L	V	T	K	D	N	V	T	V	Y	D	K	D	E	K	E	V	K	R	E	V	K	K	V	E	W	N
F	E	Q	A	S	K	G	G	Y	A	H	F	M	I	K	E	I	E	E	Q	P	E	I	I	E	K	T	L	N	V
Y	T	D	K	E	K	N	V	K	F	D	E	Q	L	E	G	I	N	F	H	D	I	D	R	I	Y	I	V	A	C
G	T	A	Y	Y	A	G	L	Q	G	Q	Y	F	M	K	K	L	L	G	I	D	V	F	T	D	I	A	S	E	F
R	Y	N	D	P	V	I	T	N	K	T	L	A	I	F	V	S	Q	S	G	E	T	I	D	T	L	M	S	M	K
Y	A	K	E	K	G	A	R	T	L	A	I	S	N	V	L	G	S	T	I	T	R	E	A	D	N	V	I	Y	T
L	A	G	P	E	I	S	V	A	S	T	K	A	Y	S	S	Q	V	L	V	M	Y	L	L	S	L	Y	M	G	A
K	L	G	K	I	E	E	K	D	Y	Q	K	Y	I	S	D	I	S	L	L	K	E	N	V	V	K	L	I	S	E
K	E	K	I	H	D	I	A	K	K	I	K	D	I	K	N	G	F	Y	L	G	R	G	I	D	E	K	V	A	R
E	G	S	L	K	M	K	E	I	N	Y	I	H	T	E	A	L	P	A	G	E	L	K	H	G	S	I	A	L	I
E	K	G	V	L	V	V	A	I	S	T	N	L	E	M	D	E	K	V	V	S	N	I	K	E	V	K	A	R	G
A	Y	V	V	G	A	C	K	E	G	S	L	V	P	E	V	V	D	D	V	I	Q	V	K	D	S	G	E	L	L
T	P	V	L	T	V	V	G	L	Q	Y	L	A	Y	Y	T	S	L	E	K	G	Y	D	V	D	K	P	R	N	L
A	K	S	V	T	V	E																							
1				5					10					15					20					25					30

14 30.16% 99.0% >tr|Q8RFC1|Q8RFC1_FUSNN Urocanate hydratase OS=Fusobacterium nucleatum subsp. nucleatum (strain ATCC 25586 / CIP 101130 / JCM 8532 / LMG 13131) OX=190304 GN=hutU PE=3 SV=1

M	L	N	N	K	N	I	Y	D	A	M	T	I	K	L	T	A	E	D	I	P	M	E	I	P	K	L	D	P	S
I	R	R	A	P	K	R	I	V	K	L	S	D	H	D	I	E	L	A	L	R	N	A	L	R	Y	I	P	E	E
F	H	E	M	L	A	P	E	F	L	K	E	L	E	D	R	G	R	I	Y	G	Y	R	F	R	P	E	G	N	I
Y	G	R	P	I	D	E	Y	K	G	K	C	T	E	A	K	A	M	Q	V	M	I	D	N	N	L	D	F	D	I
A	L	Y	P	Y	E	L	V	T	Y	G	E	T	G	Q	V	C	Q	N	W	M	Q	Y	R	L	I	K	K	Y	L
E	N	M	T	Q	D	Q	T	L	V	V	A	S	G	H	P	T	G	L	F	R	S	N	P	Y	A	P	R	A	I
I	T	N	G	L	M	V	G	L	F	D	N	Y	D	D	W	A	R	G	A	A	I	G	V	A	N	Y	G	Q	M
T	A	G	G	W	M	Y	I	G	P	Q	G	I	V	H	G	T	Y	S	T	I	L	N	A	G	R	L	F	C	G
V	P	A	D	G	D	L	R	G	K	L	F	I	T	S	G	L	G	G	M	S	G	A	Q	G	K	A	C	E	I
A	K	G	V	A	I	V	A	E	V	D	L	S	R	I	N	T	R	L	E	Q	G	W	V	N	V	I	A	N	T
P	E	E	A	F	K	I	A	E	E	K	M	A	S	K	T	P	Y	A	I	A	Y	H	G	N	I	V	E	I	L
E	Y	A	I	E	H	N	K	H	I	D	L	L	S	D	Q	T	S	C	H	A	V	Y	D	G	G	Y	C	P	V

			G T S F E E R T K L L G T D R A K F R E L V N E G L K R H Y
			K A I K T L H D R G V Y F F D Y G N S F L K S I Y D V G V T
			E I S K N G K D D K E G F I F P S Y V E D I L G P E L F D Y
			G Y G P F R W V C L S R K K E D L L K T D K A A L E L V D P
			N R R Y Q D R D N Y V W I Q D A D K N G L V V G T Q A R I F
			Y Q D A M S R T R I A L K F N E M V R N G E I G P V M L G R
			D H H D V S G T D S P F R E T S N I K D G S N I M A D M A T
			Q C F A G N A A R G M T M I A L H N G G G V G I G K S I N G
			G F G M V L D G S K R V D E I L W Q A M P W D V M G G V A R
			R A W A R N P H S I E T V V E Y N L D N K G T D H I T L P Y
			I V N D E L V K K V L K K
			1 5 10 15 20 25 30
15	38.94%	99.0%	>sp|Q8RFN7|PGK_FUSNN Phosphoglycerate kinase OS=Fusobacterium nucleatum subsp. nucleatum (strain ATCC 25586 / CIP 101130 / JCM 8532 / LMG 13131) OX=190304 GN=pgk PE=3 SV=1
			M K K I I T D L D L N N K K V L M R V D F N V P M K D G K I
			T D E N R I V Q A L P T I K Y V L E H N A K L I L F S H L G
			K V K I E E D K A T K S L K A V A E K L S E L L G K N V T F
			I P E T R G E K L E S A I N N L K S G E V L M F E N T R F E
			D L D G K K E S K N D S E L G K Y W A S L G D V F V N D A F
			G T A H R A H A S N V G I A E N I G N G N S A V G F L V E K
			E L K F I G E A V N N P K R P L I A I L G G A K V S D K I G
			V I E N L L T K A D K I L I G G A M M F T F L K A E G K N I
			G T S L V E D D K L D L A K D L L A K S N G K I V L P V D T
			V I A S E F K N D I E F S T V D V D N I P N N K M G L D I G
			E K T V T L F D S Y I K T A K T V V W N G P M G V F E M S N
			F A K G T I G V C E S I A N L T D A V T I I G G G D S A A A
			A I S L G Y A D K F T H I S T G G G A S L E F L E G K V L P
			G V E A I S N K
			1 5 10 15 20 25 30
16	26.65%	99.0%	>sp|Q8RHF4|FTHS_FUSNN Formate--tetrahydrofolate ligase OS=Fusobacterium nucleatum subsp. nucleatum (strain ATCC 25586 / CIP 101130 / JCM 8532 / LMG 13131) OX=190304 GN=fhs PE=3 SV=1
			M T D I Q I A Q A A K K E N I V E I A K K L G L T E D D I E
			Q Y G K Y K A K V N L D V L Q K N K R P N G K L I L V T A I
			T P T P A G E G K S T V T I G L T Q A L N K M G K L S A A A
			I R E P S L G P V F G M K G G A A G G G Y A Q V V P M E D I
			N L H F T G D M H A I G I A H N L I S A C I D N H I N S G N
			A L G I D V T K I T W K R V V D M N D R A L R N I V I G L G
			G K A N G Y P R Q D S F Q I T V G S E I M A I L C L S N S I
			T E L K E K I K N I V I G T S V T G K L I K V G D F H I E G
			A V A A L L K D A I K P N L V Q T L E N T P V F I H G G P F
			A N I A H G C N S I L A T K M A L K L T D Y V V T E A G F A
			A D L G A E K F I D I K C R L G G L K P D C A V I V A T V R
			A L E H H G K G D L K A G L E N L D K H I D N I K N K Y K L
			P L V V A I N K F I T D T D E Q I N M I E K F C N E R G A E
			V S L C E V W A K G G E G G I D L A E K V L K A I D N N K T
			E F D Y F Y D I N L T I K E K I E K I C K E I Y G A D G V I
			F A P A T K K V F D V I E A E G L N K L P V C M S K T Q K S
			I S D N P A L L G K P T G F K V T I N D L R L A V G A G F V
			I A M A G D I I D M P G L P K K P S A E V I D I D E N G V I
			S G L F
			1 5 10 15 20 25 30
17	25.25%	99.0%	>sp|Q8R602|EFG_FUSNN Elongation factor G OS=Fusobacterium nucleatum subsp. nucleatum (strain ATCC 25586 / CIP 101130 / JCM 8532 / LMG 13131) OX=190304 GN=fusA PE=3 SV=1
			M A R K I S L D M T R N V G I M A H I D A G K T T T T E R I
			L F Y T G V E R K L K E V H E G Q A T M D W M E Q E Q E R G
			I T I T S A A T T C F W K G H R I N I I D T P G H V D F T V
			E V E R S L R V L D G A V A V F S A V D G V Q P Q S E T V W
			R Q A D K Y K V P R L A F F N K M D R I G A N F D M C V S D
			I K E K L G S N P V P I Q I P I G A E D Q F E G V V D L I E
			M K E I V W P V D S D Q G Q H F D V K D I R A E L K E K A E
			E T R Q Y M L E S I V E T D D A L M E K F F G G E E I T K E
			E I I K G L R K A T I D N T I V P V V C G T A F K N K G I Q
			A L L D A I V N Y M P A P T D V A M V E G R D P K N P D V L
			I D R E M S D D A P F A A L A F K V M T D P F V G R L T F F
			R V Y S G F V E K G A T V L N S T K G K K E R M G R I L Q M
			H A N N R E E I E H V Y C G D I A A A V G L K D T A T G D T
			L C A E N A P I V L E Q M E F P E P V I S V A V E P K T K N
			D Q E K M G I A L S K L A E E D P T F K V R T D E E T G Q T
			I I S G M G E L H L E I I V D R M K R E F K V E S N V G K P
			Q V A Y R E T I T Q S C D Q E V K Y A K Q S G G R G Q Y G H
			V K I I L E P N P G K E F E F V N K I T G G V I P R E Y I P
			A V E K G C K E A L E S G V I A G Y P L V D V K V T L Y D G
			S Y H E V D S S E M A F K I A G S M A L K Q A A T K A K P V
			I L E P V F K V E V T T P E E Y M G D I I G D L N S R R G M
			V S G M I D R N G A K I I T A K V P L S E M F G Y A T D L R
			S K S Q G R A T Y S W E F S E Y L Q V P A S I Q K Q I Q E E
			R G K
			1 5 10 15 20 25 30
18	37.01%	99.0%	>tr|Q8RFN9|Q8RFN9_FUSNN Glyceraldehyde-3-phosphate dehydrogenase OS=Fusobacterium nucleatum subsp. nucleatum (strain ATCC 25586 / CIP 101130 / JCM 8532 / LMG 13131) OX=190304 GN=FN0652 PE=3 SV=1
			M A V K V A I N G F G R I G R L A L R V M S K N K D F D V V
			A I N D L T D A K T L A H L F K Y D S A Q G R F D G T I E V
			T D D G F V V N G D S I K V F A K A N P E E L P W K D L G V
			D V V L E C T G F F T S K E K A E A H I K A G A K K V V I S
			A P A T G D L K T V V Y N V N D N I L D G S E T V I S G A S
			C T T N C L A P M A K V L N D K F G I V E G L M T T I H A Y
			T N D Q N T L D A P H K K G D L R R A R A A A E N I V P N T
			T G A A K A I G L V I P E L K G K L D G A A Q R V P V I T G
			S I T E L V T V L G K E V T V E E I N A A M K A A S N E S F
			G Y T E E P L V S S D I I G I S F G S L F D A T Q T K V L T
			V D G K Q L V K T V A W Y D N E M S Y T S Q L I R T L K K F
			V E I S K
			1 5 10 15 20 25 30
19	34.92%	99.0%	>sp|Q8RED7|ACKA_FUSNN Acetate kinase OS=Fusobacterium nucleatum subsp. nucleatum (strain ATCC 25586 / CIP 101130 / JCM 8532 / LMG 13131) OX=190304 GN=ackA PE=3 SV=1
			M K I L V I N C G S S S L K Y Q L I N P E T E E V F A K G L
			C E R I G I D G S K L E Y E V V A K D F E K K L E T P M P S
			H K E A L E L V I S H L T D K E I G V I A S V D E V D A I G
			H R V V H G G E E F A Q S V L I N D A V L K A I E A N N D L
			A P L H N P A N L M G I R T C M E L M P G K K N V A V F D T
			A F H Q T M K P E A F M Y P L P Y E D Y K E L K V R K Y G F
			H G T S H L Y V S G I M R E I M G N P E H S K I I V C H L G
			N G A S I T A V K D G K S V D T S M G L T P L Q G L M M G T
			R C G D I D P A A V L F V K N K R G L T D A Q M D D R M N K
			K S G I L G L F G K S S D C R D L E N A V V E G D E R A I L
			A E S V S M H R L R S Y I G A Y A A I M G G V D A I C F T G
			G I G E N S S M T R E K A L E G L E F L G V E L D K E I N S
			V R K K G N V K L S K D S S K V L I Y K I P T N E E L V I A
			R D T F R L A K
			1 5 10 15 20 25 30
20	33.96%	99.0%	>tr|U7TXB8|U7TXB8_FUSNU Glutamate formiminotransferase OS=Fusobacterium nucleatum CTI-6 OX=1316587 GN=HMPREF1767_00531 PE=4 SV=1
			M A K I V E C I P N Y S E G K D L A K I E R I V A P Y K N N
			P K V K L L S V E P D A N Y N R T V V T V L G D P E E V K K
			A V I E S I G I A T K E I D M N V H K G E H K R M G A T D V
			V P F L P I Q E M T T E E C N E I S R E V G K A V W E R F Q
			L P V F L Y E S T A T A P N R V S L P D I R K G E Y E G M A
			E K L K Q P E W A P D F G E R A P H P T A G V T A I G C R M
			P L I A F N I N L A T T D M D I P K E I A K A I R F S S G G
			F R F I Q A G P A E I L D K G F V Q V T M N I K D Y T K N P
			I Y R I M E T V K M E A K R W G V K V T G C E I I G A T P F
			A S L T D S L K Y Y L A C D G I K D D V D A M S M E K V V E
			L M V K Y L G L T D F D V K K V L E A N I
			1 5 10 15 20 25 30
21	39.35%	99.0%	>tr|D5RE86|D5RE86_FUSN2 4-phosphoerythronate dehydrogenase OS=Fusobacterium nucleatum subsp. nucleatum (strain ATCC 23726 / VPI 4351) OX=525283 GN=pdxB PE=3 SV=1
			M K V L F Y G V R E V E I P L F H E L N K K E G F G Y E L E
			L I P D Y L N S K E T A E K A K G F E C V V L R G N C F A T
			K E V L D M Y K E Y G V K Y L L T R T V G T N H I D V K Y A
			K E L G F K L A Y V P F Y S P N A I A E L A V S L A M S L L
			R H L P Y T A E K F K N R N F T V D A Q M F S K E V R N C T
			V G V I G L G R I G F T A A K L F K G L G A N V I G Y D M F
			P K T G V E D I V T Q V P M D E L I K K S D I I T L H A P F
			I K E N G K I V T K E F L N N M K E N S I L I N T A R G E L
			M D L E A V V E A L E S G H L A A A G I D T I E G E V N Y F
			F K N F S D K Q A E F R A D Y P L Y N R L L D L Y P R V L V
			T P H V G S Y T D E A A S N M I E T S F E N L K E Y L D T G
			A C K N D I K A
			1 5 10 15 20 25 30
22	20.92%	99.0%	>sp|Q8RH05|DNAK_FUSNN Chaperone protein DnaK OS=Fusobacterium nucleatum subsp. nucleatum (strain ATCC 25586 / CIP 101130 / JCM 8532 / LMG 13131) OX=190304 GN=dnaK PE=3 SV=1

				M	S	K	I	I	G	I	D	L	G	T	T	N	S	C	V	A	V	M	E	G	G	S	A	T	I	I	P	N	S
				E	G	A	R	T	T	P	S	V	V	N	I	K	D	N	G	E	V	V	V	G	E	I	A	K	R	Q	A	V	T
				N	P	T	S	T	V	S	S	I	K	T	H	M	G	S	D	Y	K	V	E	I	F	G	K	K	Y	T	P	Q	E
				I	S	A	K	T	L	Q	K	L	K	K	D	A	E	A	Y	L	G	E	E	V	K	E	A	V	I	T	V	P	A
				Y	F	T	D	S	Q	R	Q	A	T	K	D	A	G	T	I	A	G	L	D	V	K	R	I	I	N	E	P	T	A
				A	A	L	A	Y	G	L	E	K	K	K	E	E	K	V	L	V	F	D	L	G	G	G	T	F	D	V	S	V	L
				E	I	S	D	G	V	I	E	V	I	S	T	A	G	N	N	H	L	G	G	D	D	F	D	N	E	I	I	N	W
				L	V	A	E	F	K	K	E	T	G	I	D	L	S	N	D	K	M	A	Y	Q	R	L	K	D	A	A	E	K	A
				K	K	E	L	S	T	L	M	E	T	S	I	S	L	P	F	I	T	M	D	A	T	G	P	K	H	L	E	M	K
				L	T	R	A	K	F	N	D	L	T	K	H	L	V	E	A	T	Q	G	P	T	K	T	A	L	K	D	A	S	L
				E	A	N	Q	I	D	E	I	L	L	V	G	G	S	T	R	I	P	A	V	Q	E	W	V	E	N	F	F	G	K
				K	P	N	K	G	I	N	P	D	E	V	V	A	A	G	A	A	I	Q	G	G	V	L	M	G	D	V	K	D	V
				L	L	L	D	V	T	P	L	S	L	G	I	E	T	L	G	G	V	F	T	K	M	I	E	K	N	T	T	I	P
				V	K	K	S	Q	V	Y	S	T	A	V	D	N	Q	P	A	V	T	I	N	V	L	Q	G	E	R	S	R	A	T
				D	N	H	K	L	G	E	F	N	L	E	G	I	P	A	A	P	R	G	V	P	Q	I	E	V	T	F	D	I	D
				A	N	G	I	V	H	V	S	A	K	D	L	G	T	G	K	E	N	K	V	T	I	S	G	S	S	N	L	S	K
				E	E	I	E	R	M	T	K	E	A	E	A	H	A	E	E	D	K	K	F	Q	E	L	V	E	A	R	N	K	A
				D	Q	L	I	S	A	T	E	K	T	L	K	E	N	P	D	K	V	S	E	E	D	K	K	N	I	E	A	A	I
				E	E	L	K	K	V	K	D	G	D	D	K	S	A	I	D	S	A	M	E	K	L	S	Q	A	S	H	K	F	A
				E	E	L	Y	K	E	V	Q	A	Q	A	Q	A	Q	Q	Q	A	G	A	N	A	G	S	D	K	K	D	E	D	V
				A	E	A	E	V	V	D																							
				1				5					10					15					20					25					30
23	19.09%	99.0%	>tr|Q8RIB8|Q8RIB8_FUSNN N-acetylneuraminate synthase OS=Fusobacterium nucleatum subsp. nucleatum (strain ATCC 25586 / CIP 101130 / JCM 8532 / LMG 13131) OX=190304 GN=FN1684 PE=4 SV=1																														
				M	K	I	G	N	K	E	I	L	K	K	G	K	P	F	F	I	A	D	I	A	A	N	H	D	G	D	L	E	R
				A	Y	K	L	I	E	L	A	K	E	A	G	A	D	V	A	K	F	Q	N	F	N	A	K	T	I	V	S	K	K
				G	F	E	T	L	G	K	Q	L	S	H	Q	A	N	W	K	K	S	V	Y	E	V	Y	K	D	A	S	I	N	P
				D	W	T	E	L	L	K	R	K	C	D	E	V	G	I	E	Y	M	T	S	P	Y	D	Y	A	S	V	D	L	V
				D	P	Y	V	N	A	Y	K	I	G	S	G	D	I	T	W	T	E	I	L	K	Y	I	L	S	K	N	K	P	I
				L	L	A	T	G	A	S	S	M	E	D	V	D	R	A	M	D	I	L	K	E	N	Q	N	I	V	L	M	Q	C
				N	T	N	Y	T	A	S	T	E	N	F	K	Y	I	N	L	N	V	L	K	K	Y	E	E	K	Y	P	N	A	I
				L	G	L	S	D	H	T	K	G	F	A	T	V	L	G	A	I	A	L	G	A	V	V	I	E	K	H	F	T	D
				D	N	D	R	E	G	P	D	H	K	F	A	M	N	P	K	T	W	R	E	M	V	D	A	S	L	E	L	Y	Y
				S	L	G	D	G	I	K	R	I	E	K	N	E	E	D	T	A	M	V	Q	R	R	G	I	Y	I	T	K	S	L
				K	A	G	H	I	L	S	R	E	D	L	I	A	L	R	P	I	K	I	D	G	I	Q	P	Y	E	I	D	L	L
				L	G	K	K	L	T	K	N	I	E	S	D	S	Y	L	T	W	K	D	V	E									
				1				5					10					15					20					25					30
24	49.47%	99.0%	>tr|J8VLZ0|J8VLZ0_9FUSO Alkyl hydroperoxide reductase C OS=Fusobacterium hwasookii ChDC F128 OX=1216362 GN=B437_04001 PE=3 SV=1																														
				M	S	L	I	G	K	K	V	P	E	F	K	A	T	A	F	K	K	G	E	K	D	F	V	T	V	T	D	K	D
				L	L	G	K	W	S	V	F	V	F	Y	P	A	D	F	T	F	V	C	P	T	E	L	E	D	L	Q	D	N	Y
				E	A	F	K	K	E	G	A	E	V	Y	S	V	S	C	D	T	A	F	V	H	K	A	W	A	D	H	S	E	R
				I	S	K	V	T	Y	P	M	V	A	D	P	T	G	F	L	A	R	A	F	E	V	M	I	E	E	E	G	L	A
				L	R	G	S	F	V	I	N	P	E	G	K	I	V	A	Y	E	V	H	D	N	G	I	G	R	E	A	K	E	L
				L	R	K	L	Q	G	A	K	F	V	A	E	H	G	E	V	C	P	A	K	W	Q	P	G	S	E	T	L	K	P
				S	L	D	L	I	G	E	L																						
				1				5					10					15					20					25					30
25	29.57%	99.0%	>sp|Q8RHX3|KDD_FUSNN L-erythro-3,5-diaminohexanoate dehydrogenase OS=Fusobacterium nucleatum subsp. nucleatum (strain ATCC 25586 / CIP 101130 / JCM 8532 / LMG 13131) OX=190304 GN=kdd PE=1 SV=1																														
				M	K	K	G	C	K	Y	G	T	H	R	V	I	E	P	A	G	V	L	P	Q	P	A	K	K	I	S	N	D	M
				E	I	F	S	N	E	I	L	I	D	V	I	A	L	N	I	D	S	A	S	F	T	Q	I	E	E	E	A	G	H
				D	V	E	K	V	K	A	K	I	K	E	I	V	A	E	R	G	K	M	Q	N	P	V	T	G	S	G	G	M	L
				I	G	T	V	E	K	I	G	D	D	L	V	G	K	T	D	L	K	V	G	D	K	I	A	T	L	V	S	L	S
				L	T	P	L	R	I	D	E	I	I	N	I	K	P	E	I	D	R	V	E	I	K	G	K	A	I	L	F	E	S
				G	I	Y	A	V	L	P	K	D	M	P	E	N	L	A	L	A	A	L	D	V	A	G	A	P	A	Q	V	A	K
				L	V	K	P	C	Q	S	V	A	I	L	G	S	A	G	K	S	G	M	L	C	A	Y	E	A	V	K	R	V	G
				P	T	G	K	V	I	G	V	V	R	N	D	K	E	K	A	L	L	Q	R	V	S	D	K	V	K	I	V	I	A
				D	A	T	K	P	M	D	V	L	H	A	V	L	E	A	N	D	A	K	E	V	D	V	A	I	N	C	V	N	V
				P	N	T	E	M	S	T	I	L	P	V	K	E	F	G	I	A	Y	F	F	S	M	A	T	G	F	S	K	A	A
				L	G	A	E	G	V	G	K	D	I	T	M	I	V	G	N	G	Y	T	V	D	H	A	A	I	T	L	E	E	L
				R	E	S	A	V	L	R	E	I	F	N	E	I	Y	L															
				1				5					10					15					20					25					30
26	7.70%	99.0%	>tr|Q8RFV3|Q8RFV3_FUSNN Hypothetical cytosolic protein OS=Fusobacterium nucleatum subsp. nucleatum (strain ATCC 25586 / CIP 101130 / JCM 8532 / LMG 13131) OX=190304 GN=FN0579 PE=4 SV=1																														
				M	F	I	L	S	I	L	A	I	A	F	V	A	C	K	K	D	K	Q	E	Q	Q	N	D	T	G	Q	S	E	T
				S	Q	G	Y	D	G	Y	Q	Q	V	L	Y	V	N	N	A	K	F	N	I	S	G	D	I	V	V	L	F	S	E
				E	L	D	K	N	Q	D	F	K	K	L	V	E	V	E	G	L	D	G	D	I	T	I	M	P	F	I	N	K	L
				I	I	K	G	N	F	K	K	D	N	P	Y	N	V	K	V	S	K	D	I	K	G	I	S	G	T	S	L	T	N
				D	Y	I	K	Y	N	L	Y	L	G	K	K	E	P	S	L	S	F	V	D	S	G	N	V	L	P	S	I	N	N
				K	K	I	N	F	N	S	V	N	I	S	K	V	K	L	E	V	V	K	V	Y	T	N	N	I	T	Q	Y	L	K
				L	Y	S	N	E	Y	G	V	N	E	W	E	L	K	D	D	L	G	D	I	V	Y	T	K	E	Y	E	I	D	S
				K	E	D	Q	V	V	K	N	S	I	D	L	S	N	T	I	D	T	K	G	I	Y	Y	V	K	I	S	A	I	G
				E	D	S	I	D	Y	D	I	A	K	Y	G	E	P	N	S	F	G	Y	D	G	D	V	I	Y	A	R	A	E	K
				T	I	I	L	S	D	I	G	I	V	A	N	S	N	N	S	K	L	D	L	K	L	L	N	L	N	T	L	N	P
				V	P	N	A	R	L	E	F	I	N	S	K	N	Q	T	L	E	E	G	T	T	N	S	N	G	E	Y	K	S	K
				T	N	L	D	K	V	F	Y	V	L	V	K	S	G	N	E	F	N	V	L	Y	L	A	G	S	K	I	N	Y	S
				D	F	D	I	G	G	S	L	D	G	S	D	L	K	L	Y	T	Y	T	D	K	G	Y	Y	R	P	G	D	E	I
				N	V	S	L	I	A	R	S	K	E	K	M	N	D	N	Q	P	F	E	Y	S	F	T	G	P	D	G	S	T	K
				I	N	N	E	I	V	K	E	S	K	N	G	F	Y	T	F	K	I	K	T	D	M	N	D	L	T	G	A	W	T
				L	S	I	K	F	G	G	K	E	I	T	Q	K	V	F	I	E	S	K	I	A	N	T	I	A	I	D	A	D	E
				D	K	I	Y	T	K	A	D	V	K	D	K	A	I	D	F	K	F	A	F	K	Y	L	S	G	A	N	V	D	K
				G	T	I	V	N	F	D	Y	A	V	I	E	K	D	T	Q	S	K	K	Y	R	E	Y	N	F	S	N	P	S	N
				Y	R	Y	Q	F	R	N	F	A	E	T	T	L	D	D	G	S	G	E	V	N	L	K	L	D	M	P	D	T	L
				Q	S	K	N	L	Y	L	S	T	I	V	N	V	S	D	A	N	G	R	Y	S	T	E	H	K	V	F	K	I	I
				N	R	E	N	S	V	G	V	Q	K	V	S	Q	N	D	N	E	T	S	V	K	Y	I	L	L	N	E	K	T	D
				S	L	V	A	G	K	K	L	K	Y	R	I	Y	N	K	E	Y	N	W	W	Y	D	Y	Y	Y	N	D	G	E	K
				S	F	K	E	N	I	E	T	V	L	L	E	E	G	E	I	T	S	G	S	S	P	E	L	L	K	V	T	K	L
				G	D	G	T	N	F	I	E	I	E	D	E	E	T	G	H	S	S	G	V	F	V	Y	K	F	H	Y	G	D	K
				R	H	G	T	I	E	N	L	N	I	T	A	D	K	E	K	Y	N	I	G	D	I	A	K	I	K	Y	S	G	A
				V	G	S	K	A	L	V	T	I	E	K	D	G	K	I	I	K	E	Y	W	K	T	L	T	V	K	D	N	E	E
				T	I	V	I	E	K	D	F	F	P	N	A	Y	V	S	I	S	V	F	Q	K	Y	V	D	K	Q	N	D	R	P
				L	R	L	Y	G	S	V	P	L	M	V	D	D	K	S	R	M	L	T	L	Q	V	D	T	K	T	E	V	L	P
				G	G	D	L	K	I	K	L	S	N	K	E	N	K	K	M	Y	Y	E	V	F	L	V	D	E	G	V	L	R	M
				T	D	Y	K	K	P	D	P	Y	K	F	F	Y	E	K	R	A	K	L	V	Q	S	Y	D	N	F	S	N	I	I
				E	R	Y	S	D	K	V	A	N	R	L	K	T	G	G	G	D	A	E	E	D	A	L	D	S	P	M	A	A	E
				V	A	Y	K	K	E	D	M	Q	L	F	G	D	A	Q	R	F	A	N	L	T	I	F	R	G	V	A	E	S	D
				E	N	G	N	A	V	V	D	V	K	L	P	N	F	F	G	R	M	R	L	F	V	V	A	V	S	D	E	S	Y
				G	S	A	E	K	S	I	S	V	K	A	P	V	I	V	E	T	S	A	P	R	V	L	K	V	G	D	K	F	T
				V	P	I	T	L	F	P	I	E	K	A	I	G	N	S	E	I	T	L	T	Y	N	G	K	T	Y	N	K	K	V
				N	V	K	D	G	K	S	E	K	V	L	F	E	L	E	A	P	A	T	V	G	T	T	K	I	D	I	D	F	K
				S	S	K	Y	S	Y	K	D	T	I	N	L	N	V	D	T	N	Y	P	Y	Q	Y	N	E	K	S	I	V	L	E
				P	N	Q	E	F	T	L	S	A	S	E	Y	K	D	F	I	N	G	S	V	K	S	K	L	V	L	S	S	Y	Q
				K	L	G	I	E	K	L	I	K	S	L	L	D	Y	P	Y	I	C	L	E	Q	I	S	S	K	G	M	A	M	L
				Y	I	D	N	L	T	T	D	P	I	E	K	N	D	A	K	N	E	I	N	T	I	I	G	K	L	N	N	N	Y
				Q	L	R	N	G	A	F	A	Y	W	P	G	S	Q	E	E	G	I	S	T	V	Y	A	I	R	F	L	I	E	A
				K	E	K	G	Y	Y	V	P	E	T	M	F	E	K	S	K	E	Y	L	N	S	I	A	M	R	T	D	V	P	K
				I	E	V	L	Y	L	L	A	A	I	G	D	P	N	V	S	E	M	N	I	V	F	D	R	Y	Y	K	N	I	S
				V	V	E	K	W	R	L	L	A	A	Y	S	K	I	G	E	K	D	F	A	R	K	E	A	D	K	L	P	R	K
				A	E	R	K	D	G	S	Y	Y	A	D	D	N	A	E	I	L	K	Y	Y	T	V	I	Y	G	T	A	D	A	E
				L	Y	N	S	V	L	A	I	A	K	S	D	A	W	L	T	T	Y	E	K	A	N	I	V	Q	A	L	A	G	D
				G	K	V	S	P	E	K	K	N	I	S	F	K	L	I	V	D	G	K	E	Q	N	L	E	L	K	D	G	E	Y
				T	F	R	N	L	G	T	K	E	N	V	K	K	I	V	I	K	N	T	S	S	S	K	L	Y	V	N	S	F	Y
				K	G	K	P	V	K	Y	D	E	K	D	E	S	K	N	I	T	I	T	R	K	F	V	D	M	A	G	K	E	I
				D	V	K	S	L	K	A	G	T	R	F	K	M	I	L	T	S	K	L	A	N	A	D	S	P	D	I	S	L	L
				Q	I	L	P	S	G	W	E	F	D	N	T	Q	T	N	I	Q	S	A	G	G	D	M	I	P	V	P	V	D	V
				E	E	V	D	N	E	E	Y	G	E	S	S	S	S	N	S	V	N	Y	V	D	I	K	D	D	R	V	A	Y	F
				Y	P	L	Y	S	G	E	D	K	V	I	E	I	N	L	I	A	V	T	P	G	T	Y	R	L	P	G	T	K	V
				E	S	M	Y	N	N	N	Y	R	A	Y	L	K	G	F	E	V	K	V	K	E									
				1				5					10					15					20					25					30
27	16.84%	99.0%	>sp|Q8RHJ2|HPPA_FUSNN Putative K(+)-stimulated pyrophosphate-energized sodium pump OS=Fusobacterium nucleatum subsp. nucleatum (strain ATCC 25586 / CIP 101130 / JCM 8532 / LMG 13131) OX=190304 GN=hppA PE=3 SV=1																														
				M	D	L	L	T	Q	V	M	Y	I	G	I	A	V	G	I	I	S	L	L	A	A	F	Y	Y	A	K	K	V	E
				H	Y	Q	I	N	I	P	K	V	E	E	I	T	A	A	I	R	E	G	A	M	A	F	L	A	A	E	Y	K	I
				L	I	V	F	V	I	V	V	A	V	A	L	G	I	F	I	S	V	P	T	A	G	A	F	I	L	G	A	I	T
				S	A	I	A	G	N	A	G	M	R	I	A	T	K	A	N	G	R	T	A	I	A	A	K	E	G	G	L	A	K
				A	L	N	V	A	F	S	G	G	A	V	M	G	L	T	V	V	G	L	G	M	F	M	L	S	L	I	L	L	V
				S	R	T	V	G	I	S	V	N	D	V	T	G	F	G	M	G	A	S	S	I	A	L	F	A	R	V	G	G	G
				I	Y	T	K	A	A	D	V	G	A	D	L	V	G	K	V	E	A	G	I	P	E	D	D	P	R	N	P	A	T
				I	A	D	N	V	G	D	N	V	G	D	V	A	G	M	G	A	D	L	F	E	S	Y	V	G	S	I	I	A	T
				I	T	L	A	F	L	L	P	V	D	D	A	T	P	Y	V	A	A	P	L	L	I	S	A	F	G	I	I	S	S
				I	I	A	T	L	T	V	K	T	D	D	G	S	K	V	H	A	K	L	E	M	G	T	R	I	A	G	I	L	T
				I	I	A	S	F	G	I	I	K	Y	L	G	L	D	M	G	I	F	Y	A	I	V	A	G	L	V	A	G	L	V
				I	A	Y	F	T	G	V	Y	T	D	T	G	R	R	A	V	N	R	V	S	D	A	A	G	T	G	A	A	T	A
				I	I	E	G	L	A	I	G	M	E	S	T	V	A	P	L	I	V	I	A	I	A	I	I	I	S	F	K	T	G
				G	L	Y	G	I	S	I	A	A	V	G	M	L	A	T	T	G	M	V	V	A	V	D	A	Y	G	P	V	A	D
				N	A	G	G	I	A	E	M	S	E	L	P	P	E	V	R	E	T	T	D	K	L	D	A	V	G	N	S	T	A
				A	V	G	K	G	F	A	I	G	S	A	A	L	T	A	L	S	L	F	A	A	Y	K	E	A	V	D	K	L	T
				S	E	A	L	V	I	D	V	T	D	P	E	V	I	A	G	L	F	I	G	G	M	L	T	F	L	F	S	A	L
				T	M	T	A	V	G	K	A	A	I	E	M	V	E	E	V	R	R	Q	F	R	E	F	P	G	I	M	D	R	T
				Q	K	P	D	Y	K	R	C	V	E	I	S	T	H	S	S	L	K	Q	M	I	L	P	G	V	L	A	I	I	V

			P V I I G L W S V K A L G G L L A G A L V T G V L M A I M M
			A N A G G A W D N G K K Q I E G G Y K G D K K G S D R H K A
			A V V G D T V G D P F K D T S G P S L N I L I K L M S I V S
			L V L V P L F V K F M
			1 5 10 15 20 25 30
28	25.79%	99.0%	>sp|Q8R5X7|CH60_FUSNN 60 kDa chaperonin OS=Fusobacterium nucleatum subsp. nucleatum (strain ATCC 25586 / CIP 101130 / JCM 8532 / LMG 13131) OX=190304 GN=groL PE=3 SV=1
			M A K I I N F N D E A R K K L E I G V N T L A D A V K V T L
			G P R G R N V V L E K S Y G A P L I T N D G V T I A K E I E
			L E D P F E N M G A A L V K E V A I K S N D V A G D G T T T
			A T I L A Q A I V K E G L K M L S A G A N P I F L K K G I E
			L A A K E A V E V L K D K A K K I E S N E E I S Q V A S I S
			A G D E E I G K L I A Q A M A K V G E T G V I T V E E A K S
			L E T T L E I V E G M Q F D K G Y V S P Y M V T D S E R M T
			A E L D N P L I L L T D K K I S S M K E L L P L L E Q T V Q
			M S K P V L I V A D D I E G E A L T T L V I N K L R G T L N
			V V A V K A P A F G D R R K A I L E D I A I L T G G E V I S
			E E K G M K L E E A T I E Q L G K A K T V K V T K D L T V I
			V D G G G Q Q K D I S A R V N S I K A Q I A E T T S D Y D K
			E K L Q E R L A K L S G G V A V I K V G A A T E V E M K D K
			K L R I E D A L N A T R A A V E E G I V A G G G T I L L D I
			I E S M K D F N E T D E I A M G I E I V K R A L E A P I K Q
			I A E N C G L N G G V V L E K V R M S P K G F G F D A K N E
			K Y V N M I E S G I I D P A K V T R A A I Q N S T S V A S L
			L L T T E V V I A N K K E E E K A P M G A G G M M P G M M
			1 5 10 15 20 25 30
29	15.31%	99.0%	>sp|Q8RFC2|HUTH1_FUSNN Histidine ammonia-lyase 1 OS=Fusobacterium nucleatum subsp. nucleatum (strain ATCC 25586 / CIP 101130 / JCM 8532 / LMG 13131) OX=190304 GN=hutH1 PE=3 SV=1
			M E V F I L E L V L G S K N I T L E D L I N V T R K G Y K V
			S I S E E A Y E K I D K A R A L V D K Y V E E G K V S Y G I
			T T G F G K F A E V S I S K E Q T G Q L Q K N I V M S H S C
			N V G N P L P I D I A K G I V L L R A V N L A K G Y S G A R
			R I V I E K L V E L L N K D V T P W I P E K G S V G S S G D
			L S P L A H M S L V L I G L G K A Y Y K G E L L E A K D A L
			A K A D I E P I P A L S S K E G L A L T N G T Q A L T S T G
			A H V L Y D A I N L S K H L D I A A S L T M E G L H G I I D
			A Y D P R I G E V R G H L G Q I N T A K N M R N I L A G S K
			N V T K Q G V E R V Q D S Y V L R C I P Q I H G A S K D T L
			E Y V K Q K V E L E L N A V T D N P I I F V D T D E V I S G
			G N F H G Q P M A L P F D F L G I A L S E M A N V S E R R I
			E K M V N P A I N N G L P A F L V E K G G L N S G F M I V Q
			Y S A A S L V S E N K V L A H P A S V D S I P T S A N Q E D
			H V S M G S V A A K K S K D I F E N V R K V I G M E L I T A
			C Q A I D L K E A K D K L S P A T K V A Y D E V R K I I S Y
			V S E D R P M Y I D I H A A E D L I K T N K I V E N V E K A
			I G K L E F
			1 5 10 15 20 25 30
30	23.63%	99.0%	>tr|Q8RGS8|Q8RGS8_FUSNN Glutaconyl-CoA decarboxylase A subunit OS=Fusobacterium nucleatum subsp. nucleatum (strain ATCC 25586 / CIP 101130 / JCM 8532 / LMG 13131) OX=190304 GN=FN0204 PE=4 SV=1
			M N Y S M P K Y F Q N M P Q V G K A L A N I D E A N E N A V
			K E I E A A I A D S V A A M Q D S G T S D E K I H D K G Q M
			T A L E R V A E L V D E G T W Y P L N T L Y N P E N F E T G
			T G I V K G L G R I G G K W A V V V A S D N K K I V G A W V
			P G Q A D N L L R A S D T A K C L G I P L V Y V L N C S G V
			K L D E Q E K V Y A N R R G G G T P F F R N A E L Q Q L G I
			P V I V G I Y G T N P A G G G Y H S I S P T I L I A H K D A
			N M A V G G A G I V G G M N P K G Y I D M E G A I Q I A E A
			T M A A K K V E V P G T I H V H Y D K T G F F R E V Y D D E
			I G V I D G I K K Y M D Y L P A Y D L E F F R V D E P T E P
			A L D P N D L Y S I I P M N Q K K I Y N I Y D V I G R L F D
			N S E F S E Y K K G Y G P E V V T G I A K V D G L L V G V I
			A N A Q G L L M N Y P E Y R E K A V G I G G K L Y R Q G L I
			K M S E F V T L C S R D R L P I V W L Q D T S G I D V G N P
			A E E A E L L G L G Q S L I Y S I E N S H V P Q I E I T I R
			K G S A A A H Y V L G G P Q G N N T N A F S L G T A A T E V
			Y V M N G E T A A S A M Y S R R L A K D Y K A G K D L Q P T
			I D K M N Q L I N E Y T A K S R P A Y C A K T G M V D E I V
			P L Y D L R G Y I S A F A N A V Y Q N P K S I C A F H Q M I
			L P R A I R E F E T Y T K K
			1 5 10 15 20 25 30
31	16.52%	99.0%	>tr|Q8RHT4|Q8RHT4_FUSNN Outer membrane protein OS=Fusobacterium nucleatum subsp. nucleatum (strain ATCC 25586 / CIP 101130 / JCM 8532 / LMG 13131) OX=190304 GN=FN1911 PE=4 SV=1
			M V N L P I K S V E V V N N Q Q V P A S L I K N T L K L K E
			G A K F S T E A L L A D F N A L K E T G Y F E D V I L Q P V
			S Y D G G V R I V V D V V E K E N V V D L L K E K G V A I N
			T L R E D T D K S I V L S S V K F T G N K R V T T S E L L D
			I T Q L K A G E Y F S R S R V E D A Q R R L L A T G K F S E
			V R P D A Q V A N G K M A L S F E V V E N P I V K S V I I T
			G N N T I P T S T I M S E L T T K P G S V Q N Y N N L R E D
			R D K I L G L Y Q A Q G Y T L V N I T D M S T D E N G T L H
			I S I V E G I V R R I E V K K M V T K Q K G N R R T P N D D
			V L K T K D Y V I D R E I E I Q P G K I F N V K E Y D A T V
			D N L M R L G I F K N V K Y E A R S I P G D P E G I D L I L
			L I D E D R T A E L Q G G V A Y G S E T G F L G T L S L K D
			S N W R G K N Q Q F G F T F E K S N K N Y T G F A L D F Y D
			P W I K D T D R V S W G W G A Y R T S Y G D E D S I L F H E
			I D T I G F R T N I G K G L G K N F T L S L G T K V E Y I K
			E K H E D G K L R Q A N N G K W Y Y K E K N K W R E I E G V
			D D K Y W L W S I Y P Y I S Y D T R N N Y L N P T S G F Y G
			K F Q V E A G H A G G Y K S G N F G N A T L E L R T Y H K G
			L F K N N I F A Y K V V G G V A T N N T K E S Q K F W V G G
			G N S L R G Y D G G F F K G S Q K L V A T I E N R T Q L N D
			I I G L V V F A D A G R A W K Q N G R D P S Y T R D N S R F
			G H N I G T T A G V G I R L N T P I G P L R F D F G W P V G
			N K M D D D G M K F Y F N M G Q S F
			1 5 10 15 20 25 30
32	27.77%	99.0%	>tr|A5TTP6|A5TTP6_FUSNP Acetoacetate metabolism regulator AtoC OS=Fusobacterium nucleatum subsp. polymorphum ATCC 10953 OX=393480 GN=atoC PE=4 SV=1
			M K N A I L A I S E K K E I L K Q I R K E L A E K Y E V I T
			F N N L L D A I D M V R E S D F D L I L L D N A L E G I S V
			G E A K K K L T S I G K D F I T V A L V D E I N E A E T K E
			L E K F G I F A Y L L K P I K I E D L D A I I L P S L N G L
			E L I K E N K R L E E K L S I L E E D T D I I G Q S A K I K
			D V R N L I E K I A D S D L P V L I V G E T G T G K D I I A
			K E I H R K S D R N K G K Y A Q V S C A L Y P G E L I E R E
			L F G Y E R G A F L G A N A S K K G L L E E I D G G T V Y I
			E D I A K M D I K V Q S R F L K A I E Y G E F K R V G G T K
			V R K S N V R F L V G T D I D L K Q E T E K G K F R K D L Y
			H R L T A L T I E V P P L R E R K E D I P V L A N Y F L N K
			I V R I L H K E T P V I S G E A M K F L M E Y Y Y P G N I M
			E L K N L I E R M A L L S K D K I L D V E Q L P L E I K T K
			S D I V E N K T V V G V G P L K E I L E Q E I Y S L E E V E
			R V V I A I A L Q K T R W N K Q E T S K I L G I G R T T L Y
			E K I R K Y G L D T K
			1 5 10 15 20 25 30
33	25.89%	99.0%	>tr|Q8RIS0|Q8RIS0_FUSNN Cell surface protein OS=Fusobacterium nucleatum subsp. nucleatum (strain ATCC 25586 / CIP 101130 / JCM 8532 / LMG 13131) OX=190304 GN=FN1499 PE=4 SV=1
			M K K F V S L K L I V F S F I L V A G S V S Y S A A P V I K
			A G T A T D S T E A G V D N V A N G V K S S A F G Y D N K A
			I E K E S S A F G T G N R A T G E F S S A F G F H N I A S K
			I H S S A F G S N N A A D G V N S S A F G F K N T V S G F N
			S S A F G S Q Y Q V T G N F S G A F G M G E F N G Q Y Q Y K
			N E G N N S Y M I G N K N K I A S G S D D N F I L G N N V H
			I G G G I N N S V A L G N N S T V S A S N T V S V G S S T L
			K R K I V N V G D G A I S A N S S D A V T G R Q L Y S G N G
			I D T A A W Q N K L N V T R K N D Y K D A N D I D V N K W K
			A K L G V G S G G G G G A P V D A Y T K S E A D N K F A N K
			T D L N D Y T K K D D Y K D A N G I D V D K W K A K L G T G
			A G T A D I E N L R N E V N E K I D D V K D E V R T V G S L
			S A A L A G L H P M Q Y D P K A P V Q V M A A L G H Y R D K
			Q S V A V G A S Y Y F N D R F M M S T G I A L S G E K R T K
			T M A N V G F T L K L G K G S G V T Y D E T P Q Y V V Q N E
			V K R L T V E N Q E L K E R V R N L E E K L N M L L K N K
			1 5 10 15 20 25 30
34	41.54%	99.0%	>sp|Q8RE43|RS4_FUSNN 30S ribosomal protein S4 OS=Fusobacterium nucleatum subsp. nucleatum (strain ATCC 25586 / CIP 101130 / JCM 8532 / LMG 13131) OX=190304 GN=rpsD PE=3 SV=1
			M A R N R Q P V L K K C R A L G I D P V I L G V K K S S N R
			Q I R P N A N K K P T E Y A T Q L R E K Q K A K F I Y N V M
			E K Q F R K I Y E E A A R K L G V T G L T L I E Y L E R R L
			E N V V Y R L G F A K T R R Q A R Q I V S H G H I A V N G R
			R V N I A S F R V K V G D I V S V I E N S K N V E L I K L A

				V	E	D	A	T	P	P	A	W	L	E	L	D	R	A	A	F	S	G	K	V	L	Q	N	P	T	K	D	D	L
				D	F	D	L	N	E	S	L	I	V	E	F	Y	S	R															
				1				5					10					15					20					25					30
35	27.60%	99.0%	>tr|Q8RED6|Q8RED6_FUSNN Phosphate acetyltransferase OS=Fusobacterium nucleatum subsp. nucleatum (strain ATCC 25586 / CIP 101130 / JCM 8532 / LMG 13131) OX=190304 GN=FN1172 PE=4 SV=1																														
				M	I	I	M	S	F	L	G	Q	V	R	K	K	A	L	Q	A	N	R	R	I	V	L	P	E	S	S	D	E	R
				I	I	R	A	T	S	Q	I	L	K	E	G	L	A	Q	V	V	L	V	G	N	Q	E	A	I	M	H	S	A	K
				A	Y	E	V	S	L	S	G	A	K	I	V	D	P	Y	N	F	E	R	L	N	D	Y	I	N	K	L	V	E	L
				R	A	K	K	G	M	T	P	E	E	A	K	K	I	L	L	N	D	P	T	F	F	G	A	M	L	V	R	M	G
				D	A	D	G	M	V	S	G	S	A	S	P	T	A	N	V	L	R	A	A	I	Q	V	I	G	T	Q	P	G	V
				K	T	V	S	S	V	F	I	M	E	L	S	Q	F	K	D	L	F	G	S	I	L	V	F	G	D	C	S	V	I
				P	F	P	T	S	E	Q	L	A	D	I	A	T	S	A	A	E	T	A	I	K	I	A	G	I	N	P	R	V	A
				L	M	T	F	S	T	K	G	S	A	K	H	E	C	V	D	R	V	I	E	A	G	H	I	L	R	E	R	K	V
				Q	F	R	F	D	A	E	L	Q	A	D	A	A	L	V	K	S	I	G	E	I	K	A	P	L	S	D	V	S	G
				N	A	N	V	L	I	F	P	T	L	S	A	G	N	I	G	Y	K	L	V	Q	R	L	A	G	A	N	A	Y	G
				P	I	I	Q	G	L	N	A	P	V	N	D	L	S	R	G	C	S	V	E	D	I	V	V	L	T	A	I	T	S
				A	Q	A	C	I	D	C																							
				1				5					10					15					20					25					30
36	9.55%	99.0%	>sp|Q8RI52|ISPH_FUSNN 4-hydroxy-3-methylbut-2-enyl diphosphate reductase OS=Fusobacterium nucleatum subsp. nucleatum (strain ATCC 25586 / CIP 101130 / JCM 8532 / LMG 13131) OX=190304 GN=ispH PE=3 SV=1																														
				M	E	I	I	R	A	K	H	M	G	F	C	F	G	V	L	E	A	I	N	V	C	N	S	L	V	E	E	K	G
				R	K	Y	I	L	G	M	L	V	H	N	K	Q	V	V	E	D	M	E	R	K	G	F	K	L	V	T	E	D	E
				L	L	N	D	M	D	E	L	K	E	D	D	I	V	V	I	R	A	H	G	T	S	K	S	V	H	E	K	L	K
				E	R	K	V	K	V	F	D	A	T	C	I	F	V	N	K	I	R	Q	E	I	E	I	A	N	E	N	G	Y	S
				I	L	F	M	G	D	K	N	H	P	E	V	K	G	V	I	S	F	A	D	D	I	Q	I	F	E	S	F	E	E
				A	K	K	L	K	I	D	L	D	K	T	Y	L	L	S	T	Q	T	T	L	N	K	K	K	F	E	E	I	K	K
				Y	F	K	E	N	Y	K	N	V	V	I	F	D	K	I	C	G	A	T	A	V	R	Q	K	A	V	E	D	L	A
				V	K	V	E	V	M	I	I	V	G	D	T	K	S	S	N	T	K	K	L	Y	E	I	S	K	K	L	N	D	N
				S	Y	L	V	E	N	E	E	Q	L	D	L	S	I	F	R	G	K	E	V	V	G	I	T	A	G	A	S	T	P
				E	E	T	I	M	N	I	E	K	K	V	R	G	I	Y	K	M	S	N	V	N	E	N	Q	N	E	F	S	L	M
				L	E	E	F	L	P	N	Q	E	K	R	V	E	G	V	I	E	S	M	D	Q	N	F	S	Y	L	D	V	P	G
				E	R	T	A	V	R	V	R	T	D	E	L	K	D	Y	K	V	G	D	T	V	E	V	L	I	T	G	L	S	E
				E	D	D	D	Q	E	Y	I	T	A	S	R	R	K	I	E	V	E	K	N	W	E	K	I	E	D	S	F	K	N
				K	T	I	L	D	A	K	V	T	K	K	I	K	G	G	Y	L	V	E	A	F	L	Y	P	G	F	L	P	N	S
				L	S	E	I	S	D	S	E	E	K	V	N	G	K	K	I	Q	V	I	V	K	D	I	K	M	D	P	K	D	K
				K	N	R	K	I	T	Y	S	V	K	D	I	R	L	A	E	Q	E	K	E	F	A	G	L	A	V	G	Q	I	V
				D	C	V	V	T	E	V	L	D	F	G	L	A	V	D	I	N	T	L	K	G	F	I	H	I	S	E	V	S	W
				K	R	L	D	K	L	S	D	N	Y	K	V	G	D	K	I	K	A	V	V	V	S	L	D	E	A	K	R	N	V
				K	L	S	I	K	K	L	E	E	D	P	W	A	T	V	A	N	E	F	K	V	D	D	E	I	E	G	I	V	T
				K	V	L	P	Y	G	A	F	V	E	I	K	P	G	V	E	G	L	V	H	I	S	D	F	S	W	T	K	K	K
				V	N	V	A	D	Y	V	K	E	R	E	K	I	K	V	R	I	T	D	L	H	P	E	D	R	K	L	K	L	G
				I	K	Q	L	V	A	N	P	W	E	T	A	E	K	D	F	A	I	D	T	V	I	K	G	K	V	V	E	V	K
				P	F	G	I	F	V	E	I	A	D	G	I	D	A	F	V	H	S	S	D	Y	N	W	V	G	E	E	I	P	K
				F	E	I	G	N	E	V	E	L	K	I	T	E	L	D	L	N	N	K	K	I	K	G	S	L	K	A	L	R	K
				S	P	W	E	H	A	M	E	E	Y	K	V	G	T	T	V	E	K	K	I	K	T	V	A	D	F	G	L	F	I
				E	L	I	K	G	I	D	G	F	I	P	T	Q	F	A	S	K	E	F	I	K	N	I	R	D	K	F	S	E	G
				D	V	V	K	A	Q	V	V	E	V	N	K	E	T	Q	K	I	K	L	S	I	K	K	I	E	I	E	E	E	K
				R	E	E	R	E	Q	I	E	K	Y	S	T	S	S	S	E	E													
				1				5					10					15					20					25					30
37	18.59%	99.0%	>tr|Q8RGB0|Q8RGB0_FUSNN Dipeptide-binding protein OS=Fusobacterium nucleatum subsp. nucleatum (strain ATCC 25586 / CIP 101130 / JCM 8532 / LMG 13131) OX=190304 GN=FN0396 PE=4 SV=1																														
				M	K	K	K	F	W	L	L	M	A	M	V	L	S	V	L	F	L	V	A	C	G	G	D	P	D	K	K	S	D
				A	G	A	G	G	K	R	D	T	L	V	I	G	N	G	A	D	A	K	S	L	D	P	H	A	S	N	D	N	P
				S	S	N	V	R	V	Q	I	F	D	T	L	M	D	L	D	D	D	G	I	P	Q	P	M	L	A	E	S	W	E
				R	P	D	D	K	T	I	I	F	H	L	R	K	G	V	K	F	H	N	G	D	E	M	K	A	S	D	V	K	F
				S	L	E	R	A	L	K	S	P	E	V	S	H	I	L	A	G	I	N	G	V	E	V	L	D	D	Y	T	V	K
				V	T	T	E	K	P	M	A	A	I	L	N	N	L	S	H	T	T	I	A	I	L	S	E	K	A	T	T	E	A
				G	D	K	F	G	Q	N	P	I	G	T	G	P	Y	K	F	V	S	W	Q	S	G	D	R	I	T	L	E	A	F
				P	D	Y	W	R	G	E	T	P	V	K	N	I	V	F	R	N	I	V	E	E	T	N	R	T	I	G	L	E	T
				G	E	L	D	I	A	Y	D	I	K	G	L	D	K	N	K	L	K	E	D	E	R	F	T	L	L	E	G	P	Q
				V	S	I	T	Y	L	G	F	N	L	R	K	A	P	Y	D	N	L	K	V	R	E	A	I	S	Y	A	I	D	Q
				K	P	I	I	D	T	V	F	L	G	A	G	E	P	A	N	S	I	I	G	P	N	I	W	G	Y	Y	D	V	E
				K	Y	T	Q	D	I	E	K	A	K	A	L	L	V	E	A	G	Y	P	N	G	F	K	A	K	I	W	V	N	D
				N	P	V	R	R	D	T	A	V	I	L	Q	D	Q	L	K	Q	I	G	I	D	L	T	I	E	T	V	E	W	G
				A	F	L	D	G	T	A	R	G	D	H	E	M	Y	L	L	G	W	G	T	V	T	R	D	P	D	Y	G	M	F
				E	L	I	S	S	S	T	M	G	A	A	G	N	R	S	F	Y	S	N	P	E	V	D	K	L	L	E	A	G	K
				T	E	L	D	P	E	K	R	K	D	I	Y	K	Q	I	Q	E	I	V	R	R	D	I	P	M	Y	M	I	V	Y
				P	L	Q	N	V	I	T	K	K	D	V	K	N	F	K	L	D	A	A	Q	T	Y	R	L	Y	G	V	S	I	E
				E																													
				1				5					10					15					20					25					30
38	3.00%	99.0%	>tr|D5REK4|D5REK4_FUSN2 Outer membrane autotransporter barrel domain protein OS=Fusobacterium nucleatum subsp. nucleatum (strain ATCC 23726 / VPI 4351) OX=525283 GN=HMPREF0397_1639 PE=4 SV=1																														
				M	K	D	Y	N	K	V	E	S	C	L	K	S	F	L	K	N	N	K	R	L	S	Y	S	M	A	L	L	I	T
				F	L	I	N	G	G	F	S	Y	A	D	E	A	V	Q	V	P	L	R	T	E	I	K	T	R	I	E	K	E	Q
				E	N	I	S	Q	M	L	K	E	A	D	E	S	M	K	D	I	E	L	K	I	K	K	L	T	Q	R	G	E	F
				W	V	K	P	L	E	K	S	Y	Q	G	F	I	F	A	N	W	G	N	Y	S	K	N	K	N	K	T	E	S	N
				F	N	G	P	E	Y	S	A	S	Y	G	R	N	M	G	Y	G	Q	F	S	N	G	K	Y	Y	G	E	Y	G	I
				V	K	N	P	L	E	F	V	D	K	I	D	F	G	A	N	I	T	P	K	A	V	I	E	K	T	I	I	E	K
				T	V	I	K	K	D	I	T	A	P	S	V	T	P	P	T	V	E	V	G	E	I	T	L	T	A	P	E	E	V
				A	I	D	E	M	T	P	P	N	E	P	N	P	S	V	T	T	P	S	T	V	P	A	L	Q	G	I	T	V	A
				A	V	S	E	V	N	V	T	P	S	T	P	E	V	A	A	A	P	T	I	N	A	P	G	V	Q	P	P	A	T
				P	A	G	F	T	P	R	L	I	T	P	P	E	A	P	E	D	I	V	I	T	P	P	G	E	V	Q	A	N	L
				T	G	S	G	A	N	P	T	T	Y	Y	Y	W	W	N	G	N	K	G	A	I	S	Q	I	S	L	T	A	G	T
				F	N	I	E	S	G	N	T	I	N	V	N	G	Y	Q	A	K	A	F	T	G	T	T	P	Q	G	T	K	P	S
				D	G	D	Y	V	I	N	Q	Q	F	F	H	T	L	L	N	V	A	Y	S	E	Y	S	N	G	V	V	I	N	N
				N	R	A	G	F	T	L	I	N	L	E	T	E	G	Q	V	S	G	N	L	D	N	R	V	G	T	Y	I	D	S
				A	K	R	D	K	L	R	S	Y	Q	M	Y	S	G	I	K	G	Q	D	N	T	E	L	L	F	I	N	K	G	T
				V	N	L	N	A	D	N	T	T	Y	L	F	T	T	S	H	N	D	G	N	L	R	T	N	Y	L	D	N	E	G
				T	I	S	A	K	G	K	N	S	I	I	I	K	H	S	P	D	T	N	Q	A	K	A	W	I	Y	S	N	N	G
				I	M	K	A	E	G	K	G	S	I	V	Y	G	A	A	Y	S	Y	L	G	S	G	R	A	A	F	V	N	D	G
				T	I	E	V	S	G	E	E	A	I	G	V	I	L	P	R	K	T	G	N	S	E	L	A	N	G	H	L	V	W
				L	K	K	P	I	S	L	K	G	K	K	S	M	G	F	V	A	Q	N	T	N	I	S	N	E	K	N	L	V	K
				F	N	I	D	A	E	K	A	I	G	I	L	Q	D	V	E	G	A	G	T	A	K	T	A	G	K	I	V	I	E
				G	N	S	S	G	S	M	G	I	Y	A	K	Q	G	I	L	E	L	I	K	P	H	A	D	S	G	E	T	E	S
				S	I	E	L	K	A	G	S	N	N	I	G	I	F	A	K	G	A	S	T	V	N	F	N	G	I	T	K	I	T
				G	G	E	R	Q	K	I	A	L	A	T	E	G	S	T	I	N	L	K	N	T	V	T	A	G	D	K	I	T	S
				N	F	V	K	D	A	V	P	L	Y	A	T	G	T	G	S	K	I	N	V	V	T	P	D	S	L	K	F	Y	L
				G	G	N	S	T	A	A	Y	A	K	D	G	G	I	I	N	M	N	R	T	N	L	P	A	D	P	S	I	Y	I
				K	G	E	N	G	K	G	I	G	L	F	A	K	D	G	G	V	I	N	A	Q	K	H	Y	I	K	V	E	N	G
				S	V	A	L	S	S	V	G	A	N	A	T	N	K	S	N	I	D	F	T	G	G	K	L	E	Y	T	G	N	G
				Y	A	V	Y	S	D	G	T	G	T	V	N	L	S	D	A	E	L	N	L	Y	G	S	S	T	A	F	D	V	D
				F	N	A	T	T	L	P	T	I	L	N	A	N	T	R	I	H	A	N	S	D	D	V	I	A	F	N	L	K	N
				A	S	G	L	T	T	V	G	G	I	E	T	S	I	K	S	K	I	E	T	K	L	G	L	G	S	G	S	L	N
				N	L	F	T	G	S	T	S	T	K	Y	K	V	A	A	V	D	G	G	E	I	T	V	G	N	L	D	K	S	G
				T	K	D	D	T	D	Q	A	K	R	D	G	Y	Q	Y	F	N	R	F	L	A	Q	R	L	K	A	T	A	T	G
				S	T	I	K	A	V	L	N	S	N	F	A	N	S	N	F	N	G	Q	V	V	G	F	E	M	N	S	S	K	N
				A	T	S	V	N	E	T	A	I	N	L	V	D	S	K	I	I	A	D	R	T	D	A	G	T	G	A	I	G	A
				F	I	N	Y	G	E	V	N	I	A	A	T	S	K	I	E	V	E	K	E	N	N	V	V	N	K	Q	A	V	G
				V	Y	A	V	N	G	S	K	V	D	N	K	G	T	I	D	V	G	G	D	Q	S	V	G	I	L	G	M	A	Y
				R	E	G	S	S	N	N	P	I	V	N	E	F	G	G	K	S	G	E	G	T	V	N	I	T	N	E	K	D	I
				K	M	S	G	K	D	A	I	G	I	Y	A	M	N	N	N	P	D	K	T	V	T	S	H	L	V	I	N	K	G
				T	V	E	V	G	D	S	A	E	K	T	A	V	G	I	Y	A	K	G	V	D	V	K	P	E	S	G	K	I	K
				I	G	K	K	A	V	G	I	Y	A	E	D	S	N	V	G	E	V	N	K	D	L	G	T	I	D	F	N	G	D
				D	G	V	G	I	Y	L	K	G	S	G	S	N	L	L	G	N	K	V	T	L	T	Q	S	K	D	S	K	N	K
				V	G	I	L	A	D	R	G	T	S	S	I	I	K	T	E	V	A	V	G	A	L	N	N	V	I	A	Y	Y	S
				K	G	N	H	E	F	N	V	Q	S	N	V	T	L	N	E	N	S	I	G	I	S	G	E	G	D	L	Q	Y	G
				D	G	T	N	P	Y	I	M	K	L	G	K	G	S	T	G	L	F	G	T	K	N	I	V	L	K	D	K	T	N
				I	E	L	N	G	E	N	S	V	G	A	Y	A	S	G	A	S	G	V	I	T	S	E	G	K	I	K	F	L	K
				E	K	S	I	G	L	Y	G	A	N	G	A	T	V	K	D	K	T	T	M	D	F	S	N	A	N	A	K	N	N
				I	G	V	Y	L	A	G	A	K	W	D	I	D	R	A	L	T	F	D	S	A	H	E	K	G	N	I	Y	L	F
				A	Q	G	G	S	T	A	T	L	K	N	G	F	D	I	T	P	L	T	A	P	T	G	N	N	R	T	I	G	M
				Y	L	D	T	A	V	K	G	G	A	T	T	A	D	N	T	V	D	M	S	D	G	N	A	K	I	S	V	T	K
				K	A	I	G	I	Y	A	K	N	V	D	N	S	K	N	N	I	I	N	K	I	K	V	L	S	D	G	Q	G	T
				V	G	V	F	T	D	G	N	L	K	L	S	G	N	G	G	L	I	E	A	Q	N	A	G	I	G	L	Y	G	N
				K	G	T	V	T	V	E	G	T	H	K	V	E	V	T	S	A	G	T	G	M	Y	L	T	K	G	S	H	L	S
				G	G	K	L	E	L	E	N	K	T	A	G	T	S	A	A	G	I	Y	Y	E	G	T	N	N	E	V	D	H	N
				T	D	I	E	V	T	A	G	E	N	L	L	A	I	Y	A	N	G	L	K	L	N	N	N	K	E	I	L	I	K
				K	G	K	N	N	V	A	A	Y	I	T	G	N	S	T	F	K	N	K	G	K	I	Q	L	G	H	P	T	Q	N
				D	F	E	S	G	I	G	I	Y	V	V	D	G	E	A	I	N	E	S	G	K	T	I	D	I	Y	D	F	E	N
				T	A	S	G	G	S	L	S	V	G	M	L	A	K	A	G	A	G	K	T	A	K	V	T	N	K	G	T	I	N
				A	N	G	E	V	I	G	M	V	V	E	D	N	S	E	G	L	N	D	T	G	A	E	I	V	A	K	D	K	E
				D	I	N	A	K	A	I	G	A	Y	V	K	G	A	N	A	K	F	E	N	K	G	K	I	S	A	E	N	I	A
				L	A	L	Q	G	T	G	A	N	K	I	L	N	S	G	T	L	N	L	T	K	T	G	A	I	G	V	Y	A	K
				D	S	V	V	D	F	N	I	A	P	T	V	A	G	A	N	K	T	V	A	L	Y	A	S	G	T	T	K	I	K
				S	Q	I	T	S	A	S	G	K	A	H	I	G	V	Y	A	E	G	D	A	E	F	L	S	G	S	K	V	T	V
				G	N	G	D	G	N	D	Y	G	I	G	V	Y	T	K	S	G	Y	N	K	T	V	N	T	D	I	Q	L	G	G
				E	K	T	I	G	F	Y	L	G	A	T	G	G	T	G	S	T	V	T	H	T	G	T	I	D	V	G	S	G	I
				G	T	Y	I	P	E	H	S	K	F	I	A	Q	N	T	T	F	N	V	G	D	N	G	T	A	V	Y	L	K	G
				G	E	V	D	L	G	K	T	G	T	A	N	I	N	F	N	G	T	N	G	R	A	I	Y	Q	D	G	G	T	I
				T	T	G	T	G	L	H	I	Q	G	S	G	S	F	L	T	L	K	N	A	N	S	S	I	N	S	L	V	E	V
				G	A	S	G	I	G	I	N	G	I	Y	D	M	A	G	K	D	Y	T	L	T	L	E	S	P	N	G	H	I	K

				L	G	G	N	K	A	T	G	I	A	A	V	A	K	S	T	V	G	P	N	K	V	N	V	I	N	K	G	T	I
				E	T	T	S	G	E	K	T	T	G	I	Y	G	K	G	A	N	I	E	N	A	T	G	A	K	I	N	I	G	A
				K	G	V	G	I	Y	T	T	N	D	N	S	L	E	N	T	T	L	N	N	A	G	E	I	N	L	T	G	D	E
				A	T	G	L	V	A	V	K	A	K	T	N	Q	D	F	I	V	G	K	I	S	G	T	K	D	K	L	V	G	A
				Y	F	K	D	S	Q	A	V	T	K	V	K	D	F	N	I	S	L	G	T	N	A	K	G	L	V	F	D	G	G
				K	D	F	T	V	T	S	S	S	T	N	K	V	K	I	G	N	T	T	G	N	S	R	G	I	G	I	A	A	L
				G	V	N	G	N	I	S	K	T	D	V	V	V	G	K	G	S	L	G	L	Y	A	K	D	K	K	L	T	F	D
				L	A	S	G	K	L	E	S	S	D	A	N	R	S	S	I	L	A	Y	A	D	G	N	T	S	E	V	A	L	N
				G	G	G	T	L	K	V	G	A	N	G	I	A	L	G	T	K	G	G	K	V	S	A	N	A	T	T	T	V	E
				V	D	G	V	K	G	L	G	A	Y	V	E	N	G	G	S	I	D	N	N	F	D	I	K	V	K	S	A	E	G
				I	G	M	Y	A	K	G	G	A	L	T	S	V	A	K	V	S	E	I	K	G	N	K	S	I	G	Y	V	F	E
				N	I	T	S	A	I	T	M	P	N	S	V	Q	L	T	D	I	N	A	T	G	Q	V	G	V	V	A	Q	G	T
				G	N	G	L	T	V	A	G	V	S	V	V	G	S	G	N	T	G	V	Y	S	S	T	G	K	A	V	I	N	N
				G	T	L	T	V	G	D	S	T	G	K	S	S	I	G	I	Y	S	K	G	G	A	V	T	S	T	G	S	A	T
				I	G	K	N	S	I	A	I	Y	G	K	D	T	A	A	T	L	N	G	N	L	T	I	G	E	K	G	I	G	L
				Y	V	D	N	T	A	T	S	K	G	D	T	A	V	N	G	N	I	T	V	G	A	N	G	A	I	G	I	Q	T
				T	N	S	K	V	N	L	T	G	D	L	S	V	A	S	G	D	S	K	G	I	F	S	M	G	A	G	N	V	E
				T	T	G	N	I	T	V	G	S	N	S	V	G	I	Y	K	N	G	S	G	E	V	K	T	A	A	G	K	T	L
				T	V	A	D	S	A	Y	G	I	F	S	K	G	A	K	L	I	N	N	M	N	V	T	V	G	V	D	A	I	G
				A	Y	V	D	G	N	D	L	T	S	T	G	T	V	T	V	G	D	K	G	V	G	L	F	V	K	G	T	G	K
				T	L	T	S	T	G	N	I	T	V	G	S	N	N	S	V	G	L	Y	A	G	D	N	A	N	I	A	Q	S	G
				N	I	T	V	A	N	N	N	G	I	G	V	Y	S	K	G	N	G	N	V	S	T	I	G	A	I	T	V	G	K
				D	S	I	G	V	Y	K	D	G	K	G	T	M	N	I	N	A	S	S	P	I	Q	T	M	T	I	A	E	K	G
				Y	G	L	Y	Y	K	G	N	S	R	A	D	S	I	I	N	S	N	M	N	M	T	L	G	K	E	A	V	G	I
				Y	A	K	N	T	T	V	N	H	V	G	D	I	T	V	G	E	T	N	I	G	S	S	G	F	T	T	P	S	D
				N	K	N	S	I	G	I	F	G	D	N	S	N	I	N	F	K	G	N	M	L	V	D	K	P	L	S	V	G	I
				Y	G	S	N	G	G	S	I	T	V	K	S	G	S	T	I	T	V	K	N	G	A	T	G	I	M	T	G	S	K
				V	E	S	I	T	L	E	S	G	S	T	L	N	V	D	G	K	V	D	T	S	V	Y	T	N	A	T	K	S	N
				I	S	F	G	I	A	A	Y	S	G	A	I	N	N	Q	G	T	I	N	V	T	N	G	A	T	G	I	Y	L	A
				G	T	A	S	L	V	N	Q	G	T	I	T	V	D	A	I	S	K	Q	I	G	R	P	D	T	K	A	S	A	E
				L	G	G	I	K	V	T	D	K	G	E	V	T	I	N	N	K	V	I	N	G	G	T	V	N	I	K	G	D	L
				N	M	A	G	M	G	L	D	V	S	T	G	K	T	I	V	D	A	R	S	I	S	G	V	A	E	V	L	P	N
				F	S	K	G	N	S	E	Q	K	V	T	I	K	D	V	F	R	T	G	A	V	G	A	F	S	G	D	V	K	S
				K	S	V	S	W	I	A	K	I	S	K	E	P	G	S	S	T	T	T	K	D	I	T	M	V	R	I	P	Y	N
				S	L	I	S	G	E	R	Y	K	N	L	A	S	G	L	E	D	I	R	S	K	I	G	K	D	S	S	S	P	I
				F	K	S	L	D	N	I	S	S	H	R	D	F	A	R	A	I	A	N	I	R	G	D	I	Y	S	N	V	Q	E
				R	M	K	T	V	E	N	A	F	D	K	S	Y	N	E	L	L	S	S	Y	N	K	T	R	N	V	N	K	F	G
				I	I	Y	T	G	G	E	H	K	D	S	T	L	G	V	S	G	Y	K	Y	K	S	T	G	V	L	Y	L	N	D
				R	E	A	F	T	Y	G	G	K	Y	G	W	S	A	G	I	V	G	S	N	F	E	F	N	G	D	T	N	K	G
				S	K	E	R	V	V	S	G	K	L	G	L	H	Y	Q	A	P	L	N	K	E	D	D	N	A	K	L	K	W	L
				T	R	G	E	V	T	V	N	N	H	R	T	N	R	Y	S	Q	V	G	K	D	T	Y	Q	N	K	A	S	F	Y
				S	T	E	L	S	W	K	N	I	I	S	Y	D	Y	D	I	N	T	N	W	M	V	K	P	Y	T	G	I	D	M
				S	Y	G	H	I	F	N	I	K	E	K	N	E	G	L	P	L	E	V	K	G	K	D	Y	F	V	I	T	P	N
				V	G	V	E	T	K	Y	V	L	P	L	G	V	T	H	Q	V	F	A	K	A	D	T	E	F	S	Y	D	V	A
				K	L	Y	H	G	V	N	Q	A	K	M	K	N	A	S	S	G	Y	Y	D	L	S	K	P	E	R	R	R	A	R
				V	A	V	G	A	E	L	G	L	E	K	E	N	A	Y	G	V	T	F	R	A	E	Y	Q	G	Y	K	K	S	Q
				L	N	Y	G	V	R	L	N	Y	K	F																			
				1				5					10					15					20					25					30
39	22.76%	99.0%	>sp|Q8RFG1|HUTI_FUSNN Imidazolonepropionase OS=Fusobacterium nucleatum subsp. nucleatum (strain ATCC 25586 / CIP 101130 / JCM 8532 / LMG 13131) OX=190304 GN=hutI PE=3 SV=1																														
				M	Q	A	D	L	V	L	Y	N	I	G	Q	L	V	T	S	R	E	L	D	K	T	K	K	M	D	N	I	E	V
				I	E	N	N	G	Y	I	V	I	E	K	D	K	I	V	A	V	G	S	G	E	V	L	K	E	Y	L	T	P	A
				T	E	M	V	D	L	S	G	K	L	V	T	P	G	L	I	D	S	H	T	H	L	V	H	G	G	S	R	E	N
				E	F	A	M	K	I	A	G	V	P	Y	L	E	I	L	E	K	G	G	G	I	L	S	T	L	K	S	T	R	N
				A	S	E	Q	E	L	I	E	K	T	L	K	S	L	R	H	M	L	E	L	G	V	T	T	V	E	A	K	S	G
				Y	G	L	N	L	E	D	E	L	K	Q	L	E	V	T	K	I	L	G	Y	L	Q	P	V	T	L	V	S	T	F
				M	A	A	H	A	T	P	P	E	Y	K	D	N	K	E	G	Y	V	Q	E	V	I	K	M	L	P	I	V	K	E
				R	N	L	A	E	F	C	D	I	F	C	E	D	K	V	F	S	V	D	E	S	R	R	I	L	T	V	A	K	E
				L	G	Y	K	L	K	I	H	A	D	E	I	V	S	L	G	G	V	E	L	A	A	E	L	G	A	T	S	A	E
				H	L	M	K	I	T	D	S	G	I	N	A	L	A	N	S	N	V	I	A	D	L	L	P	A	T	S	F	N	L
				M	E	H	Y	A	P	A	R	K	M	I	E	A	G	I	Q	I	A	L	S	T	D	Y	N	P	G	S	C	P	S
				E	N	L	Q	F	V	M	Q	I	G	A	A	H	L	K	M	T	P	K	E	V	F	K	S	V	T	I	N	A	A
				K	A	I	D	K	Q	D	T	I	G	S	I	E	V	G	K	K	A	D	I	T	V	F	D	A	P	S	M	A	Y
				F	L	Y	H	F	G	I	N	H	T	D	S	V	Y	K	N	G	K	L	V	F	K	K							
				1				5					10					15					20					25					30
40	11.37%	99.0%	>sp|Q8RH44|SYGB_FUSNN Glycine--tRNA ligase beta subunit OS=Fusobacterium nucleatum subsp. nucleatum (strain ATCC 25586 / CIP 101130 / JCM 8532 / LMG 13131) OX=190304 GN=glyS PE=3 SV=1																														
				M	E	L	L	F	E	I	G	M	E	E	I	P	A	R	F	L	N	Q	A	L	E	D	L	K	S	N	F	E	K
				K	L	K	N	N	R	I	K	F	D	G	V	R	T	Y	G	T	P	R	R	L	V	L	V	A	D	E	V	A	G
				M	Q	E	D	L	D	E	L	S	I	G	P	S	R	E	R	A	Y	K	D	G	A	L	T	K	A	G	E	G	F
				L	K	A	H	R	I	E	E	E	Q	I	E	I	I	K	N	D	K	G	E	Y	I	A	F	K	R	F	L	K	G
				K	P	T	E	A	I	L	P	E	I	L	K	A	L	V	L	E	E	T	F	Q	K	S	M	R	W	S	T	K	T
				I	R	F	A	R	P	I	E	W	F	L	A	L	Y	G	G	K	V	I	D	F	E	I	E	G	I	K	S	S	N
				K	S	K	G	H	R	F	F	G	K	E	F	E	V	S	S	V	E	N	Y	L	K	K	I	R	E	N	N	V	I
				I	D	I	S	E	R	R	K	M	I	E	E	M	I	N	N	S	L	L	E	D	E	K	A	D	V	D	K	A	L
				L	D	E	V	T	N	L	V	E	H	P	F	A	I	V	G	T	F	S	E	E	F	L	E	V	P	Q	E	V	L
				I	I	S	M	K	V	H	Q	R	Y	F	P	I	L	N	K	K	G	K	L	L	P	K	F	I	V	I	R	N	G
				I	D	F	S	E	N	V	K	K	G	N	E	K	V	L	S	A	R	L	A	D	A	R	F	F	Y	Y	E	D	L
				K	I	P	L	D	N	N	V	E	K	L	K	T	V	V	F	Q	K	D	L	G	T	I	Y	N	K	V	K	R	C
				E	K	I	A	E	F	L	V	G	K	L	K	Y	N	Y	M	K	E	D	I	L	R	T	V	K	L	A	K	A	D
				L	V	S	N	M	I	N	E	K	E	F	T	K	L	Q	G	F	M	G	E	N	Y	A	L	K	A	G	E	E	I
				G	V	A	L	G	I	K	E	H	Y	Y	P	R	F	Q	G	D	L	L	P	S	G	I	E	G	I	I	A	G	I
				S	D	R	I	D	T	L	V	G	C	F	G	V	G	V	I	P	T	G	S	K	D	P	F	A	L	R	R	T	A
				L	G	I	V	N	I	I	I	N	A	N	L	D	I	S	L	K	D	L	V	K	V	S	L	D	A	L	E	E	D
				K	V	L	K	T	D	R	A	K	V	E	T	D	V	L	D	F	L	K	Q	R	I	I	N	V	F	T	D	M	K
				Y	R	K	D	V	I	L	A	V	L	D	K	D	S	D	N	I	T	T	A	L	E	I	V	K	V	I	T	E	K
				L	S	K	D	K	M	Q	A	L	L	Q	V	V	K	R	V	S	N	I	M	K	G	N	K	D	V	T	I	K	E
				K	L	F	K	T	D	I	E	K	T	L	Y	A	E	S	K	K	V	E	S	E	V	E	Q	A	I	K	E	K	E
				Y	A	D	Y	F	E	K	L	F	T	L	V	P	T	I	D	K	Y	F	E	T	V	I	V	M	D	E	D	K	N
				V	R	D	N	R	I	G	Q	L	T	Y	I	M	N	L	F	E	K	I	A	Y	L	N	K	L	D				
				1				5					10					15					20					25					30
41	9.16%	99.0%	>sp|Q8RH47|SYI_FUSNN Isoleucine--tRNA ligase OS=Fusobacterium nucleatum subsp. nucleatum (strain ATCC 25586 / CIP 101130 / JCM 8532 / LMG 13131) OX=190304 GN=ileS PE=3 SV=1																														
				M	S	D	K	E	Y	T	S	T	L	H	L	P	K	T	D	F	Q	M	K	A	N	L	P	N	K	E	P	K	Y
				I	K	K	W	T	E	E	K	I	Y	E	K	G	L	E	K	N	K	N	G	E	T	F	I	L	H	D	G	P	P
				Y	A	N	G	N	T	H	I	G	H	A	L	N	K	I	L	K	D	I	I	L	K	Y	K	T	F	R	G	F	R
				S	P	Y	V	P	G	W	D	T	H	G	L	P	I	E	L	Q	V	V	K	E	V	G	V	G	K	A	R	E	M
				S	A	L	E	I	R	K	L	C	E	K	Y	A	K	K	W	V	G	I	Q	K	E	Q	F	I	R	L	G	V	L
				G	D	W	D	N	P	Y	L	T	L	D	P	R	F	E	A	K	Q	L	E	L	F	G	E	I	Y	V	N	G	Y
				I	F	K	G	L	K	P	V	Y	W	S	P	A	T	E	T	A	L	A	E	A	E	I	E	Y	Y	D	H	V	S
				P	S	I	Y	V	R	M	Q	A	N	K	D	L	L	D	K	I	G	F	N	E	D	V	Y	V	L	I	W	T	T
				T	P	W	T	L	P	A	N	V	A	I	C	L	N	E	N	F	D	Y	G	L	Y	K	T	E	K	G	N	L	I
				L	A	K	D	L	A	E	S	A	F	K	D	I	G	I	E	N	F	E	L	I	K	E	F	K	G	K	D	L	E
				Y	T	T	Y	K	H	P	F	L	E	R	T	G	L	I	I	L	G	D	H	V	T	A	D	A	G	T	G	A	V
				H	T	A	P	G	H	G	Q	D	D	Y	V	V	G	L	A	Y	K	L	P	V	I	S	P	I	D	H	R	G	C
				L	T	E	E	A	G	D	L	F	K	G	L	V	Y	S	E	A	N	K	A	I	I	K	H	L	T	E	T	G	H
				I	L	K	M	Q	E	I	S	H	S	Y	P	H	D	W	R	S	K	T	P	V	I	F	R	A	T	E	Q	W	F
				I	R	M	E	G	G	D	L	R	E	K	T	L	K	V	I	D	K	I	N	F	I	P	S	W	G	K	N	R	I
				G	S	M	M	E	T	R	P	D	W	C	I	S	R	Q	R	V	W	G	V	P	I	P	I	F	Y	N	D	E	T
				N	E	E	I	F	H	K	E	I	L	D	R	I	C	D	L	V	R	E	H	G	S	N	I	W	V	E	K	S	P
				E	E	L	I	G	E	E	L	L	V	K	Y	N	L	K	G	L	K	L	R	K	E	T	N	I	M	D	V	W	F
				D	S	G	S	S	H	R	G	V	L	E	V	W	E	G	L	H	R	P	C	D	L	Y	L	E	G	S	D	Q	H
				R	G	W	F	H	T	S	L	L	T	S	V	A	S	T	G	D	S	P	Y	K	S	V	L	T	H	G	F	V	N
				D	G	E	G	K	K	M	S	K	S	L	G	N	T	V	S	P	E	D	V	I	K	V	Y	G	A	D	I	L	R
				L	W	C	G	S	V	D	Y	R	D	D	V	R	I	S	D	N	I	V	K	Q	M	S	E	A	Y	R	R	I	R
				N	T	A	R	Y	I	L	G	N	S	Y	D	F	N	P	K	T	D	K	V	A	Y	K	D	M	L	E	I	D	K
				W	A	L	N	K	L	E	V	L	K	R	S	V	T	E	S	Y	D	K	Y	E	F	Y	N	L	F	Q	G	I	H
				Y	F	A	A	I	D	M	S	A	F	Y	L	D	I	I	K	D	R	L	Y	T	E	K	K	D	S	V	A	R	R
				A	A	Q	T	V	M	Y	E	V	L	M	T	L	T	K	M	V	A	P	I	L	S	F	T	A	E	E	I	W	E
				S	L	P	A	E	T	R	E	S	E	S	I	F	L	A	D	W	Y	V	N	N	D	E	Y	L	K	P	E	L	D
				E	K	W	Q	Q	I	I	K	L	R	K	E	V	N	K	K	L	E	K	A	R	Q	G	E	N	K	I	I	G	N
				S	L	D	A	K	V	S	L	Y	T	E	D	N	T	L	K	E	F	I	K	E	N	L	E	L	L	K	I	V	F
				I	V	S	D	L	E	V	V	D	S	A	D	G	N	Y	T	D	A	E	E	I	E	K	L	K	I	K	I	A	H
				A	D	G	E	K	C	E	R	C	W	K	Y	D	E	L	G	T	D	S	E	H	P	T	L	C	P	R	C	A	A
				V	L	K																											
				1				5					10					15					20					25					30
42	25.13%	99.0%	>tr|D5RC19|D5RC19_FUSN2 2-hydroxyglutaryl-CoA dehydratase D-component OS=Fusobacterium nucleatum subsp. nucleatum (strain ATCC 23726 / VPI 4351) OX=525283 GN=hgdB PE=4 SV=1																														
				M	A	E	I	K	E	L	L	E	Q	F	K	Y	Y	A	E	N	P	R	K	Q	L	D	K	Y	L	A	E	G	K
				K	A	V	G	I	F	P	Y	Y	A	P	E	E	I	V	Y	A	G	G	M	V	P	F	G	V	W	G	G	Q	G
				P	I	E	K	A	K	D	Y	F	P	T	F	Y	Y	S	L	A	L	R	C	L	E	M	A	L	D	G	T	L	D
				G	L	S	A	S	M	V	T	T	L	D	D	T	L	R	P	F	S	Q	N	Y	K	V	S	A	G	R	K	I	P
				M	V	F	L	N	H	G	Q	H	R	K	E	E	F	G	K	K	Y	N	A	K	I	F	N	N	A	K	E	E	L
				E	K	I	C	D	V	K	I	T	D	E	N	L	K	K	A	F	K	V	Y	N	E	N	R	E	E	K	R	K	F
				I	K	L	A	A	K	H	P	Q	S	I	K	A	S	D	R	S	N	V	L	K	S	S	Y	F	M	L	K	D	E
				H	T	D	L	L	R	Q	L	N	Q	K	L	E	A	L	P	E	E	Q	W	D	G	V	R	V	V	T	S	G	I
				I	T	D	N	P	G	L	L	E	V	F	D	N	Y	K	V	C	V	V	A	D	D	V	A	H	E	S	R	A	L
				K	V	D	I	D	L	S	I	E	D	P	M	L	A	L	A	D	Q	F	A	R	M	D	E	D	P	L	L	Y	D
				P	D	I	I	K	R	P	K	Y	V	L	D	L	V	K	E	N	N	A	D	G	C	L	L	F	M	M	N	F	N
				D	T	E	E	M	E	Y	P	S	L	K	Q	A	F	D	E	A	K	V	P	L	I	K	M	G	Y	D	Q	Q	M
				V	D	F	G	Q	V	K	T	Q	L	E	T	F	N	E	L	V	Q	L	S	R	F								

				1				5					10					15					20					25					30
43	13.39%	99.0%	>tr|Q8RHN0|Q8RHN0_FUSNN Thioredoxin reductase OS=Fusobacterium nucleatum subsp. nucleatum (strain ATCC 25586 / CIP 101130 / JCM 8532 / LMG 13131) OX=190304 GN=FN1984 PE=4 SV=1																														
				M	E	R	I	Y	D	M	I	V	I	G	G	G	P	A	G	L	S	A	G	I	Y	G	G	R	A	K	L	D	V
				L	I	I	E	K	E	N	K	G	G	Q	I	S	L	T	S	E	V	V	N	Y	P	G	I	L	E	I	S	G	S
				E	F	M	T	Q	T	K	K	Q	A	Q	G	F	G	V	N	F	V	Q	E	E	V	L	D	M	D	F	T	E	K
				I	K	T	I	K	T	N	N	A	E	Y	K	T	L	S	V	V	I	A	T	G	A	A	P	R	K	L	G	F	P
				G	E	Q	E	F	T	G	R	G	V	A	Y	C	A	T	C	D	G	E	F	F	T	G	M	D	I	F	V	I	G
				A	G	F	A	A	A	E	E	A	M	F	L	T	K	Y	G	K	S	V	T	I	I	A	R	E	P	D	F	T	C
				A	K	L	I	G	D	K	V	K	A	H	P	K	I	T	V	K	F	N	T	E	L	T	E	L	T	G	D	V	K
				P	T	A	A	K	F	K	N	N	V	T	G	E	I	T	E	Y	K	A	K	V	G	E	T	F	G	V	F	V	F
				V	G	Y	A	P	S	S	Q	I	F	K	G	H	I	E	I	D	K	A	G	F	I	P	T	N	E	D	L	M	T
				N	V	D	G	V	F	A	V	G	D	I	R	P	K	R	L	R	Q	V	V	T	A	V	A	D	G	A	I	A	A
				T	S	I	E	K	Y	V	H	D	L	R	E	E	L	G	I	K	K	E	E	K	K	E	E	K	T	T	S	V	T
				T	E	K	E	H	F	L	D	N	E	L	R	Q	Q	L	V	A	V	V	D	R	F	E	N	P	V	E	I	V	V
				F	K	D	P	N	N	E	E	S	V	N	I	E	N	A	V	K	D	I	A	S	I	S	P	E	K	L	K	F	S
				S	Y	N	E	G	E	N	K	E	L	E	T	K	V	K	V	T	R	T	P	T	I	A	I	L	D	K	D	G	N
				Y	T	G	L	K	Y	S	S	L	P	S	G	H	E	L	N	S	F	I	L	G	L	Y	N	V	A	G	P	G	Q
				K	V	A	T	E	S	L	E	K	I	E	K	I	N	K	P	V	N	I	K	I	G	I	S	L	S	C	T	K	C
				P	K	T	V	Q	A	T	Q	R	I	A	T	L	N	K	N	V	E	M	E	M	I	N	I	F	T	F	Q	D	F
				K	N	R	Y	D	I	M	S	V	P	A	I	I	V	D	D	Q	H	I	Y	F	G	E	K	T	V	E	D	M	L
				E	I	I	N	K																									
				1				5					10					15					20					25					30
44	15.32%	99.0%	>sp|Q8RFS6|SYT_FUSNN Threonine--tRNA ligase OS=Fusobacterium nucleatum subsp. nucleatum (strain ATCC 25586 / CIP 101130 / JCM 8532 / LMG 13131) OX=190304 GN=thrS PE=3 SV=1																														
				M	F	E	I	A	K	G	I	S	N	S	L	A	K	K	S	V	G	A	K	V	D	G	K	N	V	D	M	S	Y
				I	L	D	H	D	A	E	V	E	F	I	D	I	D	S	P	E	G	E	D	I	V	R	H	S	T	A	H	L	M
				A	Q	A	V	L	R	L	Y	P	D	T	K	V	T	I	G	P	V	I	E	N	G	F	Y	Y	D	F	D	P	V
				E	Q	F	T	E	E	D	L	E	K	I	E	A	E	M	K	R	I	V	K	E	N	I	K	L	E	K	Y	V	L
				P	R	D	E	A	I	E	Y	F	R	D	V	D	K	N	K	Y	K	V	E	V	V	E	G	I	P	Q	G	E	Q
				V	S	F	Y	K	Q	G	D	F	T	D	L	C	R	G	T	H	V	P	S	T	G	Y	L	K	A	F	K	L	R
				T	V	A	G	A	Y	W	R	G	N	S	K	N	K	M	L	Q	R	I	Y	G	Y	S	F	S	N	E	E	R	L
				K	H	H	L	K	L	M	E	E	A	E	K	R	D	H	R	K	L	G	K	E	L	E	L	F	F	L	S	E	Y
				G	P	G	F	P	F	F	L	P	K	G	M	I	V	R	N	V	L	I	D	L	W	R	R	E	H	E	K	A	G
				Y	Q	Q	L	E	T	P	I	M	L	N	K	E	L	W	E	I	S	G	H	W	F	N	Y	R	E	N	M	Y	T
				S	E	I	D	E	L	E	F	A	I	K	P	M	N	C	P	G	G	V	L	A	F	K	H	Q	L	H	S	Y	K
				D	L	P	A	R	L	A	E	L	G	R	V	H	R	H	E	F	S	G	A	L	H	G	L	M	R	V	R	S	F
				T	Q	D	D	S	H	I	F	M	T	P	D	Q	V	Q	D	E	I	I	G	V	V	N	L	I	D	K	F	Y	S
				K	L	F	G	F	E	Y	E	I	E	L	S	T	K	P	E	K	A	I	G	S	Q	E	I	W	D	M	A	E	A
				A	L	A	G	A	L	D	K	L	G	R	K	Y	K	I	N	P	G	D	G	A	F	Y	G	P	K	L	D	F	K
				I	K	D	A	I	G	R	M	W	Q	C	G	T	I	Q	L	D	F	N	L	P	E	R	F	D	V	T	Y	I	G
				E	D	G	E	K	H	R	P	V	M	L	H	R	V	I	Y	G	S	I	E	R	F	I	G	I	L	I	E	H	Y
				A	G	A	F	P	M	W	L	A	P	V	Q	V	K	V	L	T	L	N	D	E	C	I	P	Y	A	K	E	I	M
				N	K	L	E	E	L	G	I	R	A	E	L	D	D	R	N	E	T	I	G	Y	K	I	R	E	A	N	G	K	Y
				K	I	P	M	Q	L	I	I	G	K	N	E	V	E	N	K	E	V	N	I	R	R	F	G	S	K	D	Q	F	S
				K	L	L	D	E	F	Y	T	Y	V	V	D	E	A	T	I	K	F	D	K										
				1				5					10					15					20					25					30
45	17.87%	99.0%	>tr|J8S5F9|J8S5F9_9FUSO (R)-2-hydroxyglutaryl-CoA dehydratase alpha-subunit OS=Fusobacterium hwasookii ChDC F128 OX=1216362 GN=B437_02391 PE=4 SV=1																														
				M	A	G	K	M	E	K	L	P	N	K	T	P	R	P	I	E	G	H	K	P	A	A	A	I	L	R	G	V	V
				D	K	V	Y	A	N	A	W	E	A	K	K	R	G	E	L	V	G	W	S	S	S	K	F	P	I	E	L	A	K
				A	F	D	L	N	V	V	Y	P	E	N	H	A	A	S	T	A	A	K	K	D	G	L	R	L	C	Q	A	A	E
				D	M	G	Y	D	N	D	I	C	G	Y	A	R	I	S	L	A	Y	A	A	G	E	P	T	D	A	R	R	M	P
				Q	P	D	F	L	L	C	C	N	N	I	C	N	M	M	T	K	W	Y	E	N	I	A	R	M	H	N	I	P	L
				I	M	I	D	I	P	F	S	N	T	V	D	T	P	E	E	K	I	D	Y	L	V	G	Q	F	D	H	A	I	K
				Q	L	E	E	L	T	G	K	K	F	D	E	K	K	F	E	D	A	C	A	R	A	N	R	T	A	A	A	W	L
				K	S	C	K	Y	M	G	Y	K	P	S	P	L	S	G	F	D	L	F	N	H	M	A	D	I	V	A	A	R	C
				D	E	E	A	A	M	G	F	E	L	L	A	E	E	F	E	Q	S	I	K	E	G	T	S	T	W	E	Y	P	E
				E	H	R	I	L	F	E	G	I	P	C	W	P	G	L	K	P	L	F	E	P	L	K	D	N	G	V	N	V	T
				A	V	V	Y	A	P	A	F	G	F	R	Y	E	N	V	R	E	M	A	A	A	Y	C	K	A	P	C	S	V	C
				I	E	T	G	V	E	W	R	E	T	M	A	K	E	N	G	I	S	G	A	L	V	N	Y	N	R	S	C	K	P
				W	S	G	A	M	P	E	I	E	R	R	W	K	E	D	L	G	I	P	V	V	H	F	D	G	D	Q	A	D	E
				R	N	F	S	T	E	Q	Y	N	T	R	V	Q	G	L	V	E	I	M	Q	E	R	K	E	E	K	L	A	K	G
				E	E	V	Y	T	N	F	E	N	T	K	E	T	D	W	S	K	S	T	L	K	D								
				1				5					10					15					20					25					30
46	15.49%	99.0%	>tr|R9RBP6|R9RBP6_FUSNU Uncharacterized protein OS=Fusobacterium nucleatum subsp. animalis 4_8 OX=469607 GN=HMPREF0409_01699 PE=3 SV=1																														
				M	R	E	Q	C	K	E	Y	D	K	S	G	E	W	P	K	E	I	Y	D	K	A	I	E	Q	G	Y	Q	A	L
				E	V	P	E	K	Y	G	G	P	G	L	K	R	V	D	I	A	A	L	I	E	E	M	A	I	A	D	A	G	F
				A	T	T	I	S	A	S	G	L	A	M	K	P	V	F	I	S	G	N	E	E	Q	K	Q	R	M	C	D	L	V
				L	A	G	G	L	G	A	F	C	L	T	E	P	G	A	G	S	D	A	S	A	G	R	T	T	A	V	K	D	G
				N	E	Y	V	L	N	G	R	K	C	F	I	T	N	G	A	V	A	S	F	Y	C	I	T	A	I	T	D	K	E
				K	G	L	K	G	I	S	M	F	F	V	E	K	G	T	K	G	L	S	T	G	N	H	E	D	K	M	G	I	R
				T	S	N	T	C	D	V	V	L	E	D	C	R	I	P	A	S	A	L	V	G	K	E	G	E	G	F	A	I	A
				M	K	T	L	D	Q	A	R	S	W	I	G	C	I	A	V	G	I	A	Q	R	G	I	Q	E	A	I	A	Y	G
				K	E	R	I	Q	F	G	K	P	I	I	K	N	Q	A	L	Q	F	K	I	A	D	M	E	I	K	T	E	T	A
				R	Q	M	V	A	H	A	L	T	K	M	D	L	G	L	P	Y	G	K	E	S	A	I	A	K	C	Y	A	G	D
				I	A	M	Q	V	S	S	E	A	I	Q	M	F	G	G	Y	G	Y	S	R	E	Y	P	V	E	K	L	L	R	D
				S	K	I	F	Q	I	F	E	G	T	N	E	I	Q	R	I	V	I	A	N	N	V	I	G	R					
				1				5					10					15					20					25					30
47	7.69%	99.0%	>tr|Q8RGI2|Q8RGI2_FUSNN Anaerobic ribonucleoside-triphosphate reductase OS=Fusobacterium nucleatum subsp. nucleatum (strain ATCC 25586 / CIP 101130 / JCM 8532 / LMG 13131) OX=190304 GN=FN0311 PE=4 SV=1																														
				M	K	R	V	I	K	R	D	G	S	V	V	E	F	D	R	S	R	I	I	N	A	I	K	K	T	F	E	Q	A
				S	R	E	P	N	M	K	L	I	E	K	I	A	S	Q	V	E	D	L	P	D	K	V	L	A	V	E	Q	I	Q
				D	I	V	V	K	K	L	M	G	S	S	E	K	D	I	A	M	S	Y	Q	S	Y	R	T	L	K	A	E	I	R
				E	K	E	K	G	I	Y	R	Q	I	G	E	L	V	D	A	S	N	E	K	L	L	S	E	N	A	N	K	D	A
				K	T	I	S	V	Q	R	D	L	L	A	G	I	S	S	R	D	Y	Y	L	N	K	I	V	P	E	H	I	K	L
				A	H	I	K	G	E	I	H	L	H	D	L	D	Y	L	L	F	R	E	T	N	C	E	L	V	N	I	E	A	M
				L	K	G	G	C	N	I	G	N	A	K	M	L	E	P	N	S	V	D	V	A	V	G	H	I	V	Q	I	I	A
				S	V	S	S	N	T	Y	G	G	C	S	I	P	Y	L	D	R	A	L	V	R	Y	I	K	K	T	F	K	K	H
				F	L	R	G	A	K	Y	I	D	D	L	N	E	E	Q	I	E	E	L	K	K	E	N	L	E	Y	S	N	E	I
				I	K	N	K	Y	P	K	T	Y	E	Y	S	V	D	M	T	E	E	S	V	K	Q	A	M	Q	G	L	E	Y	E
				I	N	S	L	S	T	V	N	G	Q	T	P	F	T	T	V	G	I	G	T	E	T	S	W	E	G	R	L	V	Q
				K	Y	V	L	K	T	R	M	A	G	F	G	A	K	K	E	T	A	I	F	P	K	I	V	Y	A	M	C	E	G
				L	N	L	N	E	G	D	P	N	W	D	I	S	Q	L	A	F	E	C	M	T	K	S	I	Y	P	D	I	L	F
				I	T	P	E	Q	L	K	N	E	T	V	V	Y	P	M	G	C	R	A	F	L	S	P	W	K	D	K	N	G	K
				E	K	Y	A	G	R	F	N	I	G	A	T	S	I	N	L	P	R	I	A	I	K	N	R	G	D	E	E	G	F
				Y	K	E	L	D	R	I	L	E	I	C	K	D	N	C	L	F	R	A	K	Y	L	E	N	T	V	A	E	M	A
				P	I	L	W	M	S	G	A	L	A	E	K	N	Q	K	D	T	I	K	D	L	I	W	G	G	Y	S	T	V	S
				I	G	Y	I	G	L	S	E	V	S	Q	L	L	Y	G	K	D	F	S	E	S	E	E	V	Y	E	K	T	F	N
				I	L	K	Y	I	A	D	K	V	L	E	Y	K	Q	K	Y	N	L	G	F	A	L	Y	G	T	P	S	E	S	L
				C	D	R	F	A	R	V	D	K	Q	E	F	G	D	I	K	G	I	T	D	K	G	Y	Y	D	N	S	F	H	V
				S	S	R	I	N	M	S	P	F	E	K	L	R	L	E	A	L	G	H	K	Y	S	A	G	G	H	I	S	Y	I
				E	T	D	S	L	T	K	N	L	D	A	I	P	D	I	L	R	Y	A	K	M	V	G	I	H	Y	M	G	I	N
				Q	P	V	D	K	C	H	I	C	G	Y	K	G	E	F	T	A	T	K	E	G	F	T	C	P	Q	C	G	N	H
				D	S	N	E	M	S	V	I	R	R	V	C	G	Y	L	S	Q	P	N	A	R	P	F	N	K	G	K	Q	E	E
				I	M	H	R	V	K	H	S																						
				1				5					10					15					20					25					30
48	25.57%	99.0%	>tr|X7S2Z5|X7S2Z5_FUSNU Uncharacterized protein OS=Fusobacterium nucleatum 13_3C OX=1357398 GN=HMPREF2085_00805 PE=4 SV=1																														
				M	R	I	V	V	C	I	K	Q	V	P	D	T	T	E	V	K	I	D	P	V	K	G	T	I	I	R	D	G	V
				P	S	I	M	N	P	D	D	K	G	G	L	E	E	A	L	K	L	K	D	L	Y	G	A	E	V	I	V	I	T
				M	G	P	P	Q	A	E	A	I	L	R	E	A	Y	A	M	G	A	D	R	A	I	L	I	T	D	R	K	F	G
				G	A	D	T	L	A	T	S	N	T	I	A	A	A	I	R	K	I	E	D	I	D	L	I	V	A	G	R	Q	A
				I	D	G	D	T	A	Q	V	G	P	Q	I	A	E	H	L	G	L	P	Q	V	S	Y	V	K	E	M	E	Y	K
				E	D	S	K	S	F	V	I	K	R	A	T	E	D	G	Y	F	L	L	E	L	P	T	P	G	L	V	T	V	L
				A	E	A	N	Q	P	R	Y	M	N	V	G	A	I	V	D	V	F	E	R	P	I	E	T	W	T	F	D	D	I
				E	I	D	P	A	K	I	G	L	A	G	S	P	T	K	V	N	K	S	F	T	K	G	V	K	E	P	G	V	L
				H	E	V	D	A	K	E	A	A	N	I	I	L	E	K	L	K	E	K	F	I	I								
				1				5					10					15					20					25					30
49	19.82%	99.0%	>tr|Q8RHY4|Q8RHY4_FUSNN Butyrate-acetoacetate CoA-transferase subunit B OS=Fusobacterium nucleatum subsp. nucleatum (strain ATCC 25586 / CIP 101130 / JCM 8532 / LMG 13131) OX=190304 GN=FN1856 PE=4 SV=1																														
				M	E	M	D	K	N	L	V	R	E	V	I	A	K	R	V	A	Q	E	F	H	D	G	Y	V	V	N	L	G	I
				G	L	P	T	L	V	A	N	Y	V	G	D	M	D	V	I	F	Q	S	E	N	G	C	I	G	V	G	P	A	P
				E	K	G	K	E	D	P	Y	L	V	N	A	G	A	G	F	I	T	A	A	K	G	A	M	F	F	D	S	A	Y
				S	F	G	I	I	R	G	G	H	V	D	A	T	V	L	G	A	L	E	V	D	E	K	G	N	L	A	N	W	M
				I	P	G	K	K	V	P	G	M	G	G	A	M	D	L	V	V	G	A	K	K	V	I	V	A	M	E	H	T	S
				N	G	A	I	K	I	L	K	E	C	K	L	P	L	T	A	V	G	V	V	D	L	I	I	T	E	K	A	V	F
				E	V	T	D	K	G	L	V	L	K	E	I	T	P	Y	S	S	L	E	D	I	K	A	T	T	A	A	D	F	I
				I	A	D	D	L	K	K																							
				1				5					10					15					20					25					30
50	12.11%	99.0%	>tr|Q8R5V8|Q8R5V8_FUSNN ACYL-COA dehydrogenase, short-chain specific OS=Fusobacterium nucleatum subsp. nucleatum (strain ATCC 25586 / CIP 101130 / JCM 8532 / LMG 13131) OX=190304 GN=FN1424 PE=3 SV=1																														
				M	L	F	K	T	T	E	E	H	E	A	L	R	M	Q	V	R	E	F	V	E	T	E	V	K	P	I	A	A	I
				L	D	K	E	N	K	F	P	H	E	A	I	K	K	F	G	Q	M	G	F	M	G	L	P	Y	P	K	E	Y	G
				G	A	G	K	D	I	L	S	Y	A	I	A	V	E	E	L	S	R	V	D	G	G	T	G	V	I	L	S	A	H
				V	S	L	G	S	Y	P	I	F	A	F	G	T	E	E	Q	K	K	K	Y	L	T	P	L	A	K	G	E	K	L
				G	A	F	G	L	T	E	P	N	A	G	S	D	A	G	G	T	E	T	T	A	V	K	E	G	D	Y	Y	I	L
				N	G	E	K	I	F	I	T	N	A	D	V	A	E	T	Y	V	V	F	A	V	T	T	P	D	I	G	T	K	G
				I	S	A	F	I	V	E	K	G	W	E	G	F	T	F	G	D	H	Y	D	K	L	G	I	R	S	S	S	T	C

				Q	L	L	F	N	N	V	K	V	P	K	E	N	L	L	G	K	E	G	D	G	F	K	I	A	M	S	T	L	D
				G	G	R	I	G	I	A	A	Q	A	L	G	I	A	Q	G	A	F	E	H	A	L	E	Y	A	K	E	R	E	Q
				F	G	K	P	I	A	F	Q	Q	A	I	S	F	K	L	A	D	M	A	T	K	L	R	T	A	R	F	L	I	Y
				S	A	A	E	L	K	E	H	H	E	P	Y	G	M	E	S	A	M	A	K	Q	Y	A	S	D	I	A	L	E	V
				V	N	D	A	V	Q	I	F	G	G	S	G	Y	L	K	G	M	E	V	E	R	A	Y	R	D	A	K	I	T	T
				I	Y	E	G	T	N	E	I	Q	R	V	V	I	A	A	H	L	I	G	K	A	P	K	S	D	A	I	A	V	A
				K	K	K	K	G	P	V	T	G	P	R	K	N	I	I	F	K	D	G	S	T	K	E	K	V	A	A	L	V	A
				A	L	K	A	D	G	Y	D	F	T	V	G	I	P	L	D	T	P	I	G	K	S	E	R	V	V	S	A	G	K
				G	I	G	D	K	K	N	M	K	L	I	E	N	L	A	K	Q	A	G	A	S	V	G	C	S	R	P	V	A	E
				T	L	Q	Y	L	P	L	D	R	Y	V	G	M	S	G	Q	K	F	V	G	N	L	Y	I	A	C	G	I	S	G
				A	L	Q	H	L	K	G	I	K	D	A	T	T	I	V	A	I	N	T	N	A	N	A	P	I	F	K	N	A	D
				Y	G	I	V	G	D	V	A	E	I	L	P	L	L	T	K	E	L	D	N	G	E	A	K	K	D	A	P	P	M
				K	K	M	K	R	V	I	P	K	V	V	Y	S	P	H	V	Y	V	C	S	G	C	G	H	E	Y	N	P	E	I
				G	D	E	D	S	D	I	K	P	G	T	R	F	K	D	L	P	E	D	W	T	C	P	D	C	G	D	P	K	S
				G	Y	I	D	A	K																								
				1				5					10					15					20					25					30
51	23.29%	99.0%	>sp|Q8RIG1|RS3_FUSNN 30S ribosomal protein S3 OS=Fusobacterium nucleatum subsp. nucleatum (strain ATCC 25586 / CIP 101130 / JCM 8532 / LMG 13131) OX=190304 GN=rpsC PE=3 SV=1																														
				M	G	Q	K	V	D	P	R	G	L	R	L	G	I	T	R	A	W	D	S	N	W	Y	A	D	K	K	E	Y	V
				K	Y	F	H	E	D	V	Q	I	K	E	F	I	K	K	N	Y	F	H	T	G	I	S	K	V	R	I	E	R	T
				S	P	S	Q	V	V	V	H	I	H	T	G	K	A	G	L	I	I	G	R	K	G	A	E	I	D	A	L	R	A
				K	L	E	K	L	T	G	K	K	V	T	V	K	V	Q	E	I	K	D	L	N	G	D	A	V	L	V	A	E	S
				I	A	A	Q	I	E	K	R	I	A	Y	K	K	A	M	T	Q	A	I	S	R	S	M	K	S	P	E	V	K	G
				I	K	V	M	I	S	G	R	L	N	G	A	E	I	A	R	S	E	W	A	V	E	G	K	V	P	L	H	T	L
				R	A	D	I	D	Y	A	V	A	T	A	H	T	T	Y	G	A	L	G	I	K	V	W	I	F	H	G	E	V	L
				P	S	K	K	E	G	G	E	A																					
				1				5					10					15					20					25					30
52	23.04%	99.0%	>tr|D5RAS2|D5RAS2_FUSN2 Oxidoreductase, 2-nitropropane dioxygenase family protein OS=Fusobacterium nucleatum subsp. nucleatum (strain ATCC 23726 / VPI 4351) OX=525283 GN=HMPREF0397_0307 PE=4 SV=1																														
				M	K	E	L	K	G	I	K	I	G	K	Y	Y	I	E	K	P	I	V	Q	G	G	M	G	V	G	V	S	W	D
				Q	L	A	G	T	V	S	K	N	G	G	L	G	T	I	S	G	I	C	T	G	Y	Y	D	N	L	K	Y	C	K
				K	V	V	N	G	R	P	I	G	A	D	A	L	N	S	R	E	A	M	I	E	L	F	K	N	A	R	K	I	C
				G	D	K	P	L	A	C	N	I	L	H	A	L	N	D	Y	S	K	I	V	E	Y	A	L	E	A	G	A	N	I
				I	V	T	G	A	G	L	P	L	E	L	P	K	L	V	E	S	Y	P	D	V	A	I	V	P	I	V	S	S	G
				R	A	L	K	I	I	C	K	K	W	Q	A	A	G	R	L	P	D	A	V	I	V	E	G	P	K	S	G	G	H
				Q	G	V	K	A	D	D	L	F	I	P	E	H	Q	L	E	S	I	V	P	E	V	K	E	E	R	D	K	W	G
				D	F	P	I	I	A	A	G	G	I	W	D	N	D	D	I	Q	K	I	M	A	L	G	A	D	A	V	Q	L	G
				T	R	F	I	G	T	Y	E	C	D	A	S	E	E	F	K	N	I	L	I	N	A	K	K	E	D	I	V	I	V
				K	S	P	V	G	Y	P	G	R	A	V	K	T	N	L	I	K	N	L	T	H	D	T	P	T	I	K	C	Y	S
				N	C	V	T	P	C	N	L	G	E	E	A	R	K	V	G	F	C	I	A	N	C	L	S	D	S	Y	N	G	K
				V	D	T	G	L	F	F	S	G	E	N	G	Y	R	I	E	K	L	V	S	V	E	D	L	I	N	E	L	M	T
				S	T	K	N	T	I	L	A	K	V	N	S	E	N	I	I	E	N	T	V	N	F								
				1				5					10					15					20					25					30
53	20.68%	99.0%	>sp|Q8RGH3|ALF1_FUSNN Fructose-bisphosphate aldolase class 1 OS=Fusobacterium nucleatum subsp. nucleatum (strain ATCC 25586 / CIP 101130 / JCM 8532 / LMG 13131) OX=190304 GN=fda PE=3 SV=1																														
				M	N	E	K	L	E	K	M	R	N	G	K	G	F	I	A	A	L	D	Q	S	G	G	S	T	P	K	A	L	K
				L	Y	G	V	N	E	N	E	Y	S	N	D	K	E	M	F	D	L	I	H	K	M	R	T	R	I	I	K	S	P
				A	F	N	E	S	K	I	L	G	A	I	L	F	E	Q	T	M	D	S	K	I	D	G	K	Y	T	A	D	F	L
				W	E	E	K	K	V	L	P	F	L	K	I	D	K	G	L	N	D	L	D	A	D	G	V	Q	T	M	K	P	N
				P	T	L	A	D	L	L	K	R	A	N	E	R	H	I	F	G	T	K	M	R	S	V	I	K	K	A	S	P	A
				G	I	A	R	V	V	E	Q	Q	F	E	V	A	A	Q	V	V	A	A	G	L	I	P	I	I	E	P	E	V	D
				I	N	N	V	D	K	V	Q	C	E	E	I	L	R	D	E	I	R	K	H	L	N	A	L	P	E	T	S	N	V
				M	L	K	L	T	L	P	T	V	E	N	L	Y	E	E	F	T	K	H	P	R	V	V	R	V	V	A	L	S	G
				G	Y	S	R	E	K	A	N	D	I	L	S	K	N	K	G	V	I	A	S	F	S	R	A	L	T	E	G	L	S
				A	Q	Q	T	D	E	E	F	N	K	T	L	A	A	S	I	D	G	I	Y	E	A	S	V	K					
				1				5					10					15					20					25					30
54	38.89%	99.0%	>tr|Q8REM0|Q8REM0_FUSNN Neutrophil-activating protein A OS=Fusobacterium nucleatum subsp. nucleatum (strain ATCC 25586 / CIP 101130 / JCM 8532 / LMG 13131) OX=190304 GN=FN1079 PE=3 SV=1																														
				M	K	N	K	E	N	L	N	K	Y	L	S	N	L	G	I	L	I	T	K	T	H	N	L	H	W	N	V	V	G
				A	R	F	K	A	I	H	E	Y	T	E	S	L	Y	D	Y	Y	F	E	K	F	D	E	V	A	E	A	F	K	M
				K	G	E	F	P	L	V	K	V	A	D	Y	L	K	H	A	T	V	K	E	L	E	A	K	D	F	T	I	P	E
				V	V	T	S	I	K	E	D	I	E	L	M	L	A	D	A	R	K	I	R	E	V	A	N	E	E	D	D	F	L
				V	A	N	M	M	E	D	Q	I	E	Y	F	V	K	Q	L	W	F	I	S	A	M	A	K						
				1				5					10					15					20					25					30
55	9.15%	99.0%	>tr|J8V1Z4|J8V1Z4_9FUSO PTS system, fructose-specific IIABC component OS=Fusobacterium hwasookii ChDC F128 OX=1216362 GN=B437_10762 PE=4 SV=1																														
				M	E	I	K	D	L	L	K	K	D	L	M	I	M	D	L	K	A	S	T	K	M	E	A	I	D	E	M	V	A
				K	L	K	E	K	N	I	I	S	D	E	A	V	F	K	D	L	I	L	K	R	E	E	R	S	S	T	G	L	G
				E	G	I	A	M	P	H	A	K	T	S	V	V	N	T	P	S	V	L	F	A	R	S	N	K	G	I	D	Y	D
				A	L	D	D	E	P	V	Y	I	F	F	M	I	A	A	S	E	G	A	H	D	L	H	I	E	T	L	A	K	L
				S	K	M	L	L	N	D	D	F	T	Q	G	L	K	T	C	G	S	P	D	E	V	Y	A	L	V	D	K	Y	S
				E	K	P	Q	E	A	V	K	E	E	V	K	E	V	Q	N	T	N	K	K	R	I	L	A	V	T	A	C	P	T
				G	I	A	H	T	Y	M	A	E	A	A	L	K	E	A	G	E	K	L	G	V	D	V	K	V	E	T	N	G	A
				D	G	I	K	N	N	L	T	T	N	D	I	N	E	A	V	G	I	I	V	A	A	D	K	K	V	E	T	A	R
				F	N	D	R	K	V	I	V	T	S	T	A	D	A	I	K	N	A	E	T	L	I	K	K	V	L	N	N	E	A
				P	I	F	K	A	E	A	S	D	K	T	E	E	D	T	Q	E	N	D	S	I	G	R	I	I	Y	K	S	I	M
				S	G	V	S	N	M	L	P	F	V	I	G	G	G	I	L	L	A	I	S	F	I	V	E	R	F	M	G	Q	N
				Q	L	F	K	L	L	F	D	V	G	A	G	A	F	H	F	L	I	P	V	L	A	G	F	I	A	M	S	I	A
				D	K	P	G	F	M	P	G	A	V	A	G	Y	M	A	S	Q	G	A	G	F	L	G	G	L	I	G	G	F	I
				A	G	Y	S	V	I	F	L	K	K	M	T	K	N	M	S	K	Q	F	D	G	M	K	S	M	V	I	Y	P	I
				F	S	L	L	I	T	G	I	L	M	Y	F	I	I	G	P	I	F	T	K	V	N	L	I	V	A	N	W	L	N
				N	M	G	T	A	N	A	V	L	L	G	A	I	L	G	G	M	M	S	V	D	M	G	G	P	I	N	K	A	A
				Y	A	F	S	I	G	V	F	T	D	T	G	N	G	A	F	M	A	A	V	M	A	G	G	M	V	P	P	L	A
				I	A	L	A	M	T	L	F	K	N	N	F	D	E	K	E	K	Q	S	T	I	S	N	F	I	L	G	L	S	F
				I	T	E	G	A	I	P	F	A	A	K	E	P	V	K	V	I	G	S	C	I	V	G	A	A	I	A	G	G	L
				T	Q	F	W	S	V	S	A	P	A	P	H	G	G	I	F	V	I	P	A	M	P	S	V	H	S	A	I	F	F
				I	V	S	I	A	I	G	A	I	V	S	G	V	I	F	G	I	L	R	G	K	K	K							
				1				5					10					15					20					25					30
56	6.69%	99.0%	>sp|Q8RFJ8|SYA_FUSNN Alanine--tRNA ligase OS=Fusobacterium nucleatum subsp. nucleatum (strain ATCC 25586 / CIP 101130 / JCM 8532 / LMG 13131) OX=190304 GN=alaS PE=3 SV=1																														
				M	L	T	G	N	E	I	R	E	K	F	I	E	F	F	M	Q	K	Q	H	K	H	F	E	S	A	S	L	I	P
				D	D	P	T	L	L	L	T	V	A	G	M	V	P	F	K	P	Y	F	L	G	Q	K	E	A	P	C	P	R	V
				T	T	Y	Q	K	C	I	R	T	N	D	L	E	N	V	G	R	T	A	R	H	H	T	F	F	E	M	L	G	N
				F	S	F	G	N	Y	F	K	K	E	A	I	K	W	S	W	E	F	V	T	E	V	L	K	I	N	K	D	K	L
				W	V	T	V	F	T	T	D	D	E	A	E	K	I	W	I	E	E	C	N	F	P	K	E	R	I	V	R	M	G
				E	S	E	N	W	W	S	A	G	P	T	G	S	C	G	P	C	S	E	I	H	V	D	L	G	I	Q	Y	G	G
				D	E	N	S	K	I	G	D	E	G	T	D	N	R	F	I	E	I	W	N	L	V	F	T	E	W	N	R	M	E
				D	G	S	L	E	P	L	P	K	K	N	I	D	T	G	A	G	L	E	R	I	A	A	V	V	Q	G	K	T	N
				N	F	E	T	D	L	L	F	P	I	L	E	E	A	G	K	I	T	G	S	Q	Y	G	K	N	P	E	T	N	F
				S	L	K	V	I	T	D	H	A	R	A	V	T	F	L	V	N	D	G	V	I	P	S	N	E	G	R	G	Y	I
				L	R	R	I	L	R	R	A	V	R	H	G	R	L	L	G	Y	K	D	L	F	M	Y	K	M	V	D	K	V	V
				E	K	F	E	I	A	Y	P	D	L	R	K	N	V	E	N	I	R	K	I	V	K	I	E	E	E	K	F	S	N
				T	L	D	Q	G	I	Q	L	V	N	Q	E	I	D	N	L	L	T	N	G	K	N	K	L	D	G	E	I	S	F
				K	L	Y	D	T	Y	G	F	P	Y	E	L	T	E	E	I	A	E	E	R	G	V	I	V	L	R	E	E	F	E
				A	K	M	E	E	Q	K	E	K	A	R	S	A	R	E	V	V	M	E	K	G	Q	D	S	F	I	E	E	F	Y
				D	K	Y	G	V	T	E	F	T	G	Y	E	K	T	E	D	E	G	K	L	L	S	L	R	E	A	K	D	R	K
				Y	L	L	I	F	D	K	T	P	F	Y	A	E	S	G	G	Q	V	G	D	Q	G	K	I	Y	S	D	N	F	A
				G	K	V	L	D	V	Q	K	Q	K	D	I	F	I	H	T	V	K	L	E	K	G	M	P	E	E	N	K	T	Y
				K	L	E	V	D	V	V	K	R	L	D	T	A	K	N	H	T	A	T	H	L	L	H	K	A	L	R	E	I	V
				G	T	H	V	Q	Q	A	G	S	L	V	D	S	E	K	L	R	F	D	F	S	H	Y	E	A	L	T	E	E	Q
				L	S	K	I	E	D	I	V	N	K	K	I	R	E	G	I	E	V	V	V	S	H	H	S	I	E	E	A	K	K
				L	G	A	M	M	L	F	G	D	K	Y	G	D	V	V	R	V	V	D	V	H	G	F	S	T	E	L	C	G	G
				T	H	I	D	N	I	G	K	I	G	L	F	K	I	T	S	E	G	G	I	A	A	G	V	R	R	I	E	A	K
				T	G	Y	G	A	Y	L	V	E	K	E	E	A	D	I	L	K	N	I	E	Q	K	L	K	A	T	N	S	N	L
				V	E	K	V	E	K	N	L	E	T	L	K	D	T	E	K	E	L	E	I	L	K	Q	K	L	A	L	F	E	T
				K	A	A	I	S	G	M	E	E	I	G	G	V	K	V	L	I	A	A	F	K	D	K	S	T	E	D	L	R	T
				M	I	D	T	I	K	D	N	N	E	K	A	I	I	V	L	A	S	T	Q	D	K	L	A	F	A	V	G	V	T
				K	T	L	T	D	K	I	K	A	G	D	L	V	K	K	L	A	E	I	T	G	G	K	G	G	G	R	P	D	F
				A	Q	A	G	G	K	D	E	G	K	L	L	D	A	F	K	E	V	R	E	I	I	E	A	K	L	V			
				1				5					10					15					20					25					30
57	17.45%	99.0%	>sp|Q8RHI3|RL1_FUSNN 50S ribosomal protein L1 OS=Fusobacterium nucleatum subsp. nucleatum (strain ATCC 25586 / CIP 101130 / JCM 8532 / LMG 13131) OX=190304 GN=rplA PE=3 SV=1																														
				M	G	K	H	R	G	K	K	Y	L	E	V	A	K	L	V	E	I	G	K	L	Y	D	I	R	E	A	L	E	L
				V	Q	K	T	K	T	A	K	F	T	E	T	V	E	V	A	L	R	L	G	V	D	P	R	H	A	D	Q	Q	I
				R	G	T	V	V	L	P	H	G	T	G	K	T	V	K	I	L	A	I	T	S	G	E	N	I	E	K	A	L	A
				A	G	A	D	Y	A	G	A	E	E	Y	I	N	Q	I	Q	Q	G	W	L	D	F	D	L	V	I	A	T	P	D
				M	M	P	K	I	G	R	L	G	K	I	L	G	T	K	G	L	M	P	N	P	K	S	G	T	V	T	P	D	I
				A	A	A	V	S	E	F	K	K	G	K	L	A	F	R	V	D	K	L	G	S	I	H	A	P	I	G	K	V	D
				F	D	L	D	K	I	E	E	N	F	K	A	F	M	D	Q	I	I	R	L	K	P	A	S	S	K	G	Q	Y	L
				R	T	V	A	V	S	L	T	M	G	P	G	V	K	M	D	P	A	I	V	G	K	I	V	G					
				1				5					10					15					20					25					30
58	12.09%	99.0%	>tr|R9RBQ6|R9RBQ6_FUSNU Inosine-5'-monophosphate dehydrogenase OS=Fusobacterium nucleatum subsp. animalis 4_8 OX=469607 GN=guaB PE=3 SV=1																														
				M	M	N	G	K	I	V	K	E	G	I	T	F	D	D	V	L	L	I	P	A	K	S	N	V	L	P	N	E	V
				S	L	K	T	R	L	T	K	K	I	T	L	N	L	P	I	L	S	A	A	M	D	T	V	T	E	S	D	L	A
				I	A	L	A	R	Q	G	G	I	G	F	I	H	K	N	M	S	I	E	E	Q	A	A	E	V	D	R	V	K	R
				S	E	S	G	M	I	T	N	P	I	T	L	N	K	D	S	R	V	F	Q	A	E	E	L	M	S	R	Y	K	I
				S	G	L	P	V	I	E	D	D	G	K	L	I	G	I	I	T	N	R	D	I	K	Y	R	K	E	L	D	Q	P
				V	G	D	I	M	T	S	K	G	L	I	T	A	P	V	G	T	T	L	E	Q	A	K	E	I	L	L	A	N	R
				I	E	K	L	P	I	T	D	Q	N	G	Y	L	K	G	L	I	T	I	K	D	I	D	N	I	I	Q	Y	P	N
				A	C	K	D	N	L	G	K	L	R	C	G	A	A	V	G	I	A	H	D	T	I	E	R	V	R	A	L	V	K

				A	G	V	D	I	I	T	V	D	S	A	H	G	H	S	Q	G	V	I	N	M	I	K	E	I	K	K	N	F	P
				D	L	D	V	I	G	G	N	I	V	T	A	E	A	A	K	E	L	I	E	A	G	V	S	A	V	K	V	G	I
				G	P	G	S	I	C	T	T	R	V	V	A	G	V	G	V	P	Q	L	T	A	V	N	D	V	Y	E	Y	C	K
				D	K	N	I	G	V	I	A	D	G	G	I	K	L	S	G	D	I	V	K	A	L	A	A	G	G	D	C	V	M
				L	G	G	L	L	A	G	T	K	E	A	P	G	E	E	I	I	L	E	G	R	R	F	K	I	Y	V	G	M	G
				S	I	A	A	M	K	R	G	S	K	D	R	Y	F	Q	A	E	E	R	D	N	S	K	L	V	P	E	G	I	E
				G	R	I	A	Y	K	G	S	V	K	D	V	I	F	Q	L	A	G	G	I	R	A	G	M	G	Y	C	G	T	K
				T	I	K	D	L	Q	I	N	G	K	F	V	K	I	T	G	A	G	L	I	E	S	H	P	H	D	I	T	I	T
				K	E	A	P	N	Y	S	K																						
				1				5					10					15					20					25					30
59	9.00%	99.0%	>tr|R9RE94|R9RE94_FUSNU PTS systemN-acetylglucosamine-specific IIBC component OS=Fusobacterium nucleatum subsp. animalis 4_8 OX=469607 GN=HMPREF0409_01715 PE=4 SV=1																														
				M	F	G	Y	L	Q	K	I	G	K	A	L	M	V	P	V	A	V	L	P	A	A	A	I	M	L	G	I	G	Y
				W	I	D	P	T	G	W	G	A	N	S	Q	L	A	A	F	L	I	K	A	G	A	A	I	M	D	N	M	A	I
				L	F	A	V	G	V	A	Y	G	L	S	K	D	K	D	G	A	A	A	L	A	G	L	V	A	F	E	I	V	T
				T	L	L	S	T	G	A	V	S	Q	I	M	G	I	P	Q	E	E	V	H	A	A	F	G	K	I	N	N	Q	F
				I	G	I	L	C	G	V	I	S	G	E	L	Y	N	K	F	H	K	I	E	L	P	R	F	L	A	F	F	S	G
				K	R	F	V	P	I	I	T	S	V	V	M	I	V	V	S	F	I	L	T	Y	I	W	P	I	I	Y	G	A	L
				V	T	F	G	T	N	I	A	K	L	G	P	V	G	A	G	I	Y	G	F	F	N	R	L	L	I	P	V	G	L
				H	H	A	V	N	S	V	F	W	F	N	V	A	G	I	N	D	I	G	R	F	W	G	S	P	D	M	A	Y	A
				D	L	P	E	I	L	Q	G	T	Y	H	V	G	M	Y	Q	A	G	F	F	P	I	M	M	F	G	L	L	G	A
				C	A	A	F	I	Q	T	S	K	P	E	N	R	T	K	I	F	S	I	M	V	A	A	G	F	T	S	F	L	T
				G	V	T	E	P	I	E	F	A	F	M	F	V	A	P	V	L	Y	L	L	H	A	L	L	T	G	I	S	L	F
				L	A	A	S	F	N	W	M	A	G	F	S	F	S	G	G	F	I	D	F	F	L	S	L	K	N	P	N	A	N
				N	P	F	M	L	I	V	L	G	L	V	F	F	V	V	Y	Y	F	V	F	L	F	V	I	K	A	F	N	L	K
				T	P	G	R	E	E	S	E	E	E	K	A	E	A	I	K	V	K	T	T	N	S	E	L	A	E	T	L	V	G
				L	L	G	G	A	D	N	I	V	E	V	D	N	C	T	T	R	L	R	L	K	V	K	D	S	A	N	I	K	E
				S	E	I	K	K	L	V	P	G	V	L	K	P	S	K	E	S	V	Q	V	I	I	G	P	H	V	E	F	V	A
				T	E	L	K	R	I	L	G	K																					
				1				5					10					15					20					25					30
60	12.77%	99.0%	>tr|D5RC25|D5RC25_FUSN2 Glutaconate CoA-transferase subunit A OS=Fusobacterium nucleatum subsp. nucleatum (strain ATCC 23726 / VPI 4351) OX=525283 GN=gctA PE=4 SV=1																														
				M	S	K	I	M	S	L	H	D	A	I	A	K	Y	V	E	S	G	D	S	L	C	F	G	G	F	T	T	N	R
				K	P	Y	A	A	V	Y	E	I	I	R	Q	G	Q	T	D	F	I	G	Y	S	G	P	A	G	G	D	W	D	M
				L	I	G	C	G	R	I	K	A	F	I	N	C	Y	I	A	N	S	G	Y	T	N	V	C	R	R	F	R	D	A
				V	E	K	K	H	N	L	L	L	E	D	Y	S	Q	D	V	I	M	L	M	L	H	A	S	S	L	G	L	P	Y
				L	P	V	K	L	M	E	G	S	D	L	E	Y	K	W	G	I	S	A	E	I	R	K	T	I	P	K	L	P	D
				K	K	L	E	R	I	P	N	P	F	K	E	G	E	E	V	I	A	V	P	V	P	R	L	D	T	A	I	I	S
				V	Q	K	A	S	I	N	G	T	C	S	I	E	G	D	E	F	H	D	I	D	I	A	I	A	A	K	K	V	I
				V	I	A	E	E	I	V	T	E	E	E	I	R	R	D	P	S	K	N	S	I	P	Q	F	C	V	D	A	V	V
				H	V	P	Y	G	T	H	P	S	Q	L	Y	N	Y	Y	D	Y	D	A	D	F	Y	K	M	Y	D	K	V	T	K
				T	D	E	D	F	E	Q	F	I	K	E	W	V	I	D	V	K	D	H	E	G	Y	L	A	K	L	G	L	P	R
				V	S	K	L	K	V	V	P	G	F	Q	Y	A	A	K	L	V	K	D	G	E									
				1				5					10					15					20					25					30
61	20.65%	99.0%	>sp|Q8RIF8|RL2_FUSNN 50S ribosomal protein L2 OS=Fusobacterium nucleatum subsp. nucleatum (strain ATCC 25586 / CIP 101130 / JCM 8532 / LMG 13131) OX=190304 GN=rplB PE=3 SV=1																														
				M	A	I	R	K	M	K	P	I	T	N	G	T	R	H	M	S	R	L	V	N	D	E	L	D	K	V	R	P	E
				K	S	L	T	V	P	L	K	S	A	Y	G	R	D	N	Y	G	H	R	T	C	R	D	R	Q	K	G	H	K	R
				L	Y	R	I	I	D	F	K	R	N	K	L	D	V	P	A	R	V	A	T	I	E	Y	D	P	N	R	S	A	N
				I	A	L	L	F	Y	V	D	G	E	K	R	Y	I	L	A	P	K	G	L	K	K	G	D	I	V	S	A	G	S
				K	A	E	I	K	P	G	N	A	L	K	L	K	D	M	P	V	G	V	Q	I	H	N	I	E	L	Q	R	G	K
				G	G	Q	L	V	R	S	A	G	T	A	A	R	L	V	A	K	E	G	T	Y	C	H	V	E	L	P	S	G	E
				L	R	L	I	H	G	E	C	M	A	T	V	G	E	V	G	N	S	E	H	N	L	V	N	I	G	K	A	G	R
				A	R	H	M	G	K	R	P	H	V	R	G	A	V	M	N	P	V	D	H	P	H	G	G	G	E	G	K	N	S
				V	G	R	K	S	P	L	T	P	W	G	K	P	A	L	G	I	K	T	R	G	R	K	T	S	D	K	F	I	V
				R	R	R	N	E	K																								
				1				5					10					15					20					25					30
62	10.21%	99.0%	>sp|Q8RH11|SYS_FUSNN Serine--tRNA ligase OS=Fusobacterium nucleatum subsp. nucleatum (strain ATCC 25586 / CIP 101130 / JCM 8532 / LMG 13131) OX=190304 GN=serS PE=3 SV=2																														
				M	L	E	L	K	F	M	R	E	N	V	E	M	L	K	E	M	L	K	N	R	N	S	N	V	D	M	D	A	F
				V	E	L	D	S	K	R	R	E	V	L	S	E	V	E	N	L	K	R	E	R	N	N	A	S	A	E	I	A	N
				L	K	K	E	K	K	N	A	D	H	I	I	E	K	M	G	E	V	S	T	K	I	K	D	L	D	A	E	L	V
				E	I	D	E	K	I	K	D	I	Q	L	N	I	P	N	V	Y	H	P	S	T	P	I	G	P	D	E	D	Y	N
				L	E	I	R	K	W	G	I	P	K	K	F	D	F	E	P	K	S	H	W	D	I	G	E	D	L	G	I	L	D
				F	E	R	G	A	K	L	S	G	S	R	F	V	L	Y	R	G	A	A	A	R	L	E	R	A	I	I	N	F	M
				L	D	V	H	T	L	E	E	G	Y	T	E	H	I	T	P	F	M	V	K	A	E	V	C	E	G	T	G	Q	L
				P	K	F	E	E	D	M	Y	K	T	T	D	D	M	Y	L	I	S	T	S	E	I	T	M	T	N	I	H	R	K
				E	I	L	E	Q	A	E	L	P	K	Y	Y	T	A	Y	S	P	C	F	R	R	E	A	G	S	Y	G	K	D	V
				K	G	L	I	R	L	H	Q	F	N	K	V	E	M	V	K	I	T	D	A	E	S	S	Y	D	E	L	E	K	M
				V	N	N	A	E	T	I	L	Q	R	L	E	L	P	Y	R	V	I	Q	L	C	S	G	D	L	G	F	S	A	A
				K	T	Y	D	L	E	V	W	L	P	S	Q	N	K	Y	R	E	I	S	S	C	S	N	C	E	A	F	Q	A	R
				R	M	G	L	K	Y	R	V	P	N	G	S	E	F	C	H	T	L	N	G	S	G	L	A	V	G	R	T	L	V
				A	I	M	E	N	Y	Q	Q	E	D	G	S	F	L	V	P	K	V	L	I	P	Y	M	G	G	V	D	V	I	K
				K																													
				1				5					10					15					20					25					30
63	10.76%	99.0%	>tr|J8VN08|J8VN08_9FUSO DNA polymerase III subunit beta OS=Fusobacterium hwasookii ChDC F128 OX=1216362 GN=B437_01720 PE=3 SV=1																														
				M	H	I	K	I	N	R	Q	N	F	L	L	A	I	R	T	V	E	K	S	V	K	E	N	K	I	K	P	I	L
				S	C	I	Y	A	K	V	K	G	N	K	I	Y	F	T	G	T	N	L	D	T	T	I	K	T	S	I	D	V	N
				E	V	T	R	E	G	E	V	A	F	Y	Y	S	I	I	D	E	Y	L	K	E	I	K	D	E	F	V	I	L	R
				V	E	N	G	N	I	L	F	I	E	T	E	D	S	T	T	E	Y	D	V	F	N	P	E	D	Y	P	N	T	F
				E	N	V	I	L	N	E	N	N	F	K	F	E	M	P	S	Q	E	L	V	N	I	F	E	K	V	L	F	S	A
				D	T	P	D	N	I	A	M	N	C	I	R	I	E	S	I	A	K	Y	L	H	F	V	S	T	N	T	Y	R	L
				T	F	L	K	K	N	I	D	K	D	I	Q	D	F	S	V	S	V	P	A	D	T	I	S	S	L	I	K	I	I
				K	G	L	D	N	E	V	I	K	V	Y	K	E	D	A	H	L	Y	F	Q	Y	K	D	T	M	I	I	T	K	L
				I	E	L	R	F	P	N	Y	A	D	I	L	S	N	I	S	Y	D	K	K	L	F	I	N	N	E	K	L	T	N
				L	L	K	R	I	L	I	F	S	R	S	N	A	E	S	K	Y	S	S	T	Y	E	F	K	T	N	E	E	N	Q
				N	K	M	S	I	S	A	L	N	E	I	A	R	I	N	E	E	L	D	V	N	F	E	G	E	N	L	K	I	S
				L	N	S	K	Y	L	L	E	F	I	Q	N	I	P	K	E	K	E	L	V	L	E	F	M	Y	S	N	S	A	V
				K	V	Y	E	K	D	N	D	E	Y	I	Y	I	L	M	P	L	A	L	R	E									
				1				5					10					15					20					25					30
64	8.16%	99.0%	>tr|D5RDV7|D5RDV7_FUSN2 M18 family aminopeptidase OS=Fusobacterium nucleatum subsp. nucleatum (strain ATCC 23726 / VPI 4351) OX=525283 GN=HMPREF0397_1392 PE=3 SV=1																														
				M	E	K	L	K	L	A	K	H	L	I	N	F	I	D	E	S	P	S	N	Y	F	A	C	I	N	T	K	N	I
				L	N	D	K	G	F	I	E	L	F	E	T	E	E	W	K	L	K	K	G	G	K	Y	F	V	T	I	N	D	S
				G	I	I	A	F	T	I	G	S	E	K	I	S	K	S	G	Y	K	I	A	A	S	H	T	D	S	P	G	F	L
				I	K	P	S	P	E	I	N	R	K	G	F	N	I	L	N	T	E	V	Y	G	G	P	I	L	S	T	W	F	D
				R	P	L	S	F	S	G	R	V	F	V	E	S	D	N	A	L	K	P	K	K	Y	F	I	K	Y	D	K	D	L
				F	I	I	P	S	L	C	I	H	Q	N	R	G	V	N	D	G	M	A	I	N	A	Q	K	D	T	L	P	L	V
				T	I	T	D	E	N	E	K	F	S	L	K	K	L	L	A	K	Q	L	K	V	K	E	D	K	I	L	S	Y	D
				L	N	L	Y	S	R	E	K	G	C	L	L	G	A	K	G	E	F	I	S	V	G	R	L	D	N	L	A	A	L
				H	A	G	L	M	S	L	V	N	N	K	D	K	R	N	T	C	V	V	V	G	Y	D	N	E	E	I	G	S	N
				S	I	Q	G	A	D	S	P	T	L	K	N	I	L	E	R	I	S	N	A	M	K	L	S	F	E	E	H	Q	Q
				A	L	A	N	S	F	V	I	S	N	D	A	A	H	S	I	H	P	N	Y	L	E	K	S	D	P	T	N	E	P
				K	I	N	A	G	P	V	I	K	M	A	A	N	K	S	Y	I	T	D	G	Y	S	K	S	V	I	E	K	I	A
				K	D	F	K	I	P	I	Q	T	F	V	N	R	S	D	V	R	G	G	S	T	I	G	P	I	Q	Q	S	Q	I
				R	I	L	G	I	D	I	G	S	P	L	L	S	M	H	S	V	R	E	L	G	G	V	D	D	H	Y	N	L	Y
				R	L	I	S	E	F	F	K	I																					
				1				5					10					15					20					25					30
65	8.14%	99.0%	>tr|X7S8P8|X7S8P8_FUSNU Glutamate--tRNA ligase OS=Fusobacterium nucleatum 13_3C OX=1357398 GN=gltX PE=3 SV=1																														
				M	K	G	L	I	K	M	C	V	D	C	K	K	R	V	R	T	R	V	A	P	S	P	T	G	D	P	H	V	G
				T	A	Y	I	A	L	F	N	I	A	F	A	H	V	N	G	G	D	F	I	L	R	I	E	D	T	D	R	N	R
				Y	T	E	G	S	E	Q	M	I	F	D	A	L	K	W	L	D	L	D	Y	S	E	G	P	D	V	G	G	D	Y
				G	P	Y	R	Q	S	E	R	F	D	L	Y	G	K	Y	A	K	E	L	V	E	K	G	G	A	Y	Y	C	F	C
				D	H	E	R	L	E	N	L	R	E	R	Q	K	A	M	G	L	P	P	G	Y	D	G	H	C	R	S	L	S	K
				E	E	I	E	E	K	L	K	A	G	V	P	Y	V	I	R	L	K	M	P	Y	E	G	E	T	V	I	H	D	R
				L	R	G	D	V	V	F	E	N	S	K	I	D	D	Q	V	L	L	K	A	D	G	Y	P	T	Y	H	L	A	N
				I	V	D	D	H	L	M	G	I	T	H	V	I	R	A	E	E	W	I	P	S	T	P	K	H	I	Q	L	Y	K
				A	F	G	W	E	A	P	E	F	I	H	M	P	L	L	R	N	D	D	R	S	K	I	S	K	R	K	N	P	V
				S	L	I	W	Y	K	E	E	G	Y	L	K	E	G	L	V	N	F	L	G	L	M	G	Y	S	Y	G	D	G	Q
				E	I	F	T	L	Q	E	F	K	D	N	F	N	I	D	K	V	T	L	G	G	P	V	F	D	L	V	K	L	G
				W	V	N	N	Q	H	M	K	M	K	D	L	G	E	L	T	R	L	T	V	P	F	F	V	N	E	G	Y	L	E
				N	E	N	V	S	E	K	E	F	D	T	L	K	K	I	V	A	I	E	R	E	G	A	K	T	L	R	E	L	A
				K	N	S	K	F	F	F	V	D	E	F	S	L	P	E	L	K	E	D	M	D	K	K	E	R	K	S	V	E	K
				L	L	N	S	L	K	D	E	T	G	L	K	S	I	K	L	F	I	E	K	L	E	K	W	E	G	N	E	F	T
				A	E	E	A	K	D	L	L	H	S	L	L	D	D	L	Q	E	G	P	G	K	I	F	M	P	L	R	A	V	L
				T	G	A	T	K	G	A	D	L	Y	N	I	L	Y	V	I	G	K	E	R	A	L	K	R	I	Q	D	T	I	K
				K	Y	N	I	A	L																								
				1				5					10					15					20					25					30
66	5.64%	99.0%	>sp|Q8RI93|SECA_FUSNN Protein translocase subunit SecA OS=Fusobacterium nucleatum subsp. nucleatum (strain ATCC 25586 / CIP 101130 / JCM 8532 / LMG 13131) OX=190304 GN=secA PE=3 SV=1																														
				M	I	G	S	L	L	K	K	I	F	G	T	K	N	D	R	E	I	K	A	L	T	K	E	V	E	K	I	N	A
				L	E	S	E	Y	E	K	L	S	D	E	D	L	K	N	K	T	N	I	F	K	E	R	L	K	N	G	E	T	L
				D	D	I	L	V	E	A	F	A	T	V	R	E	A	S	K	R	V	L	G	L	R	H	Y	D	V	Q	L	I	G
				G	M	V	L	H	Q	G	K	I	T	E	M	K	T	G	E	G	K	T	L	V	A	T	C	P	V	Y	L	N	A
				L	A	G	H	G	V	H	V	I	T	V	N	D	Y	L	A	K	R	D	R	D	Q	M	S	R	L	Y	G	F	L
				G	L	S	S	G	V	I	L	N	G	L	P	T	D	Q	R	K	K	S	Y	N	S	D	I	T	Y	G	T	N	S
				E	F	G	F	D	Y	L	R	D	N	M	V	S	S	L	D	Q	K	V	Q	R	E	L	N	F	C	I	V	D	E
				V	D	S	I	L	I	D	E	A	R	T	P	L	I	I	S	G	A	A	E	D	K	I	K	W	Y	Q	I	S	F
				Q	V	V	S	M	L	N	R	S	Y	E	T	E	K	I	K	N	I	K	E	K	K	A	M	N	I	P	D	E	K

				W	G	D	Y	E	V	D	E	K	S	R	V	I	V	F	T	E	K	G	V	K	R	V	E	E	I	L	K	I	D
				N	L	Y	A	P	E	Y	V	E	L	T	H	F	L	N	Q	A	L	K	A	K	E	L	F	K	R	D	R	D	Y
				L	V	R	D	N	G	E	V	V	I	I	D	E	F	T	G	R	A	M	E	G	R	R	Y	S	D	G	L	H	Q
				A	I	E	A	K	E	G	V	K	I	A	S	E	N	Q	T	L	A	T	I	T	L	Q	N	Y	F	R	M	Y	K
				K	L	S	G	M	T	G	T	A	E	T	E	A	T	E	F	M	H	T	Y	G	L	E	V	V	V	I	P	T	N
				L	P	V	I	R	K	D	D	A	D	L	V	Y	K	T	K	K	E	K	I	N	S	I	I	D	R	I	Q	G	L
				Y	E	K	G	Q	P	V	L	V	G	T	I	S	I	K	S	S	E	E	L	S	E	L	L	K	K	R	K	I	P
				H	N	V	L	N	A	K	Y	H	A	K	E	A	E	I	V	A	Q	A	G	R	Y	K	A	V	T	I	A	T	N
				M	A	G	R	G	T	D	I	M	L	G	G	N	P	E	F	M	A	L	A	E	V	D	S	R	D	D	E	N	F
				P	E	V	F	A	K	Y	Q	E	Q	C	A	N	E	K	E	Q	V	L	A	L	G	G	L	F	I	L	G	T	E
				R	H	E	S	R	R	I	D	N	Q	L	R	G	R	S	G	R	Q	G	D	P	G	E	S	E	F	Y	L	S	L
				E	D	D	L	M	R	L	F	G	S	E	R	V	M	I	W	M	D	R	L	K	L	P	E	G	E	P	I	T	H
				R	M	I	N	S	A	I	E	K	A	Q	K	K	I	E	A	R	N	F	G	I	R	K	N	L	L	E	F	D	D
				V	M	N	K	Q	R	T	A	I	Y	E	S	R	N	E	A	L	A	I	D	N	L	K	D	R	I	L	G	M	L
				Q	R	N	I	T	E	K	V	Y	E	K	F	A	P	E	M	R	E	D	W	D	I	D	G	L	N	E	Y	L	K
				D	F	Y	V	Y	E	E	R	D	D	K	A	Y	L	R	S	T	K	E	E	Y	I	E	R	I	Y	N	A	L	V
				E	Q	Y	N	N	K	E	A	E	L	G	S	D	L	M	R	K	L	E	K	H	I	L	F	D	V	V	D	N	R
				W	R	G	H	L	K	S	L	D	A	L	R	E	S	I	Y	L	R	A	Y	G	Q	R	D	P	V	T	E	Y	K
				L	I	S	S	Q	I	F	E	E	M	I	A	T	I	Q	E	Q	A	T	S	F	L	F	K	V	V	V	N	T	E
				P	V	K	D	E	K	N	E	I	E	A	D	G	L	C	P	C	G	S	G	K	P	Y	E	K	C	C	G	R	
				1				5					10					15					20					25					30
67	9.43%	99.0%	>sp|Q8R600|EFTS_FUSNN Elongation factor Ts OS=Fusobacterium nucleatum subsp. nucleatum (strain ATCC 25586 / CIP 101130 / JCM 8532 / LMG 13131) OX=190304 GN=tsf PE=3 SV=2																														
				M	A	T	V	T	A	A	L	V	K	E	L	R	E	R	T	G	A	G	M	L	D	C	K	K	A	L	E	S	H
				D	G	D	I	E	K	S	I	D	Y	L	R	E	K	G	I	A	K	A	V	K	K	A	G	R	I	A	A	E	G
				L	I	F	D	E	A	T	P	D	H	K	K	A	V	I	L	E	F	N	S	E	T	D	F	V	A	K	N	E	E
				F	K	E	F	G	R	K	L	V	K	I	A	L	E	R	N	V	H	Q	L	E	E	L	N	E	A	Q	V	E	G
				D	K	K	V	S	E	A	L	T	D	L	I	A	K	I	G	E	N	M	S	L	R	R	L	A	V	V	V	A	K
				D	G	F	V	Q	T	Y	S	H	L	G	G	K	L	G	V	I	V	E	M	S	G	E	P	T	E	A	N	L	E
				K	A	K	N	I	A	M	H	V	A	A	M	D	P	K	Y	L	S	E	E	E	V	T	A	S	D	L	E	H	E
				K	E	I	A	R	K	Q	L	E	E	E	G	K	P	A	N	I	I	E	K	I	L	T	G	K	M	H	K	F	Y
				E	E	N	C	L	V	D	Q	I	Y	V	R	A	E	N	K	E	T	V	K	Q	Y	A	G	D	I	K	V	L	S
				F	E	R	F	K	V	G	D	G	I	E	K	R	E	E	D	F	A	A	E	V	A	A	Q	I	N	G			
				1				5					10					15					20					25					30
68	6.60%	99.0%	>tr|A0A101K6U9|A0A101K6U9_FUSNC Uncharacterized protein OS=Fusobacterium nucleatum subsp. nucleatum OX=76856 GN=RO03_08750 PE=4 SV=1																														
				M	Y	N	I	L	E	V	A	S	C	H	G	G	D	I	N	Y	L	Y	S	L	I	E	K	F	R	F	F	N	K
				N	E	N	F	G	I	K	F	Q	P	F	K	Y	D	L	I	A	T	K	D	Y	S	N	Y	S	I	Y	K	A	L
				F	F	N	K	N	E	W	K	D	I	I	Q	K	A	F	F	T	K	D	I	W	L	D	I	F	D	E	Y	S	L
				E	I	L	E	D	N	L	E	K	I	K	G	I	K	F	Q	T	S	I	L	D	N	L	I	I	F	N	R	L	K
				N	I	K	T	D	N	L	H	L	I	L	N	I	A	G	R	N	L	E	D	I	E	K	I	L	E	K	Y	S	N
				L	H	F	K	E	V	Y	L	E	V	G	F	Q	G	Y	P	T	S	L	E	D	C	G	V	S	K	I	S	I	L
				K	K	R	F	E	N	I	K	I	V	F	A	D	H	T	D	S	Q	T	E	E	A	T	I	L	P	I	L	V	G
				F	E	N	C	D	I	I	E	K	H	I	M	L	D	R	E	K	T	K	Y	D	F	Y	S	S	L	T	F	E	Q
				Y	E	K	F	I	E	M	Q	K	K	Y	F	K	L	K	E	Q	D	F	I	N	K	R	E	V	E	Y	F	K	K
				S	V	Q	I	P	V	L	N	K	D	K	K	A	G	E	L	I	D	I	E	N	D	F	E	F	R	R	T	D	S
				L	G	L	N	L	L	E	I	K	K	L	V	S	E	K	Y	I	L	A	S	D	K	K	K	G	E	T	I	T	E
				E	D	L	K	K	A	N	I	A	V	I	I	A	C	R	L	K	S	S	R	L	K	R	K	A	L	L	K	I	G
				T	L	S	S	I	E	M	C	I	K	N	V	L	K	F	E	D	T	N	S	V	V	L	A	T	S	I	T	E	E
				D	S	I	L	K	N	Y	T	Y	N	K	S	V	I	F	H	Q	G	D	P	D	D	V	I	Q	R	Y	I	D	I
				I	N	K	K	N	Y	D	V	I	V	R	V	T	G	D	C	P	Y	L	S	K	D	I	C	K	F	V	L	D	S
				H	F	E	K	G	A	E	Y	S	N	G	I	G	A	A	I	G	T	N	V	E	I	M	N	S	L	C	L	K	E
				I	K	K	Y	F	P	R	A	D	Y	S	E	Y	M	T	W	Y	F	Q	N	N	S	E	V	F	K	L	N	Y	V
				D	L	P	N	K	W	K	R	D	Y	R	L	T	L	D	Y	Q	E	D	L	D	L	F	N	K	V	E	E	Y	F
				S	K	N	N	I	N	Y	T	I	D	N	L	F	E	F	L	D	K	N	P	E	I	S	K	I	N	L	N	M	P
				V	R	Y	Y	V	D	Q	E	L	I	K	T	L	D	R	E	T	K	I	K	K									
				1				5					10					15					20					25					30
69	7.82%	99.0%	>tr|D5RBS6|D5RBS6_FUSN2 Uncharacterized protein OS=Fusobacterium nucleatum subsp. nucleatum (strain ATCC 23726 / VPI 4351) OX=525283 GN=HMPREF0397_0661 PE=4 SV=1																														
				M	S	I	R	K	F	R	K	K	M	K	P	F	I	I	I	L	T	V	V	F	I	L	S	L	A	Y	G	G	Y
				E	S	Y	R	T	S	R	A	N	K	K	A	Q	E	A	M	L	L	N	K	D	Y	I	Q	K	I	E	I	E	R
				A	K	Q	D	L	S	R	T	Y	S	D	R	V	D	K	E	L	V	D	I	L	A	F	N	D	V	I	D	K	N
				L	T	L	H	I	A	K	D	L	K	V	K	V	P	S	S	E	V	N	K	Q	Y	E	E	L	E	S	S	M	G
				D	K	E	Q	F	R	R	M	L	Q	V	R	G	L	T	K	D	S	L	K	N	Q	I	E	E	N	L	L	M	Q
				K	T	R	E	E	F	A	K	N	I	N	P	N	D	E	E	I	D	A	Y	M	A	L	Y	S	I	P	S	D	K
				R	E	D	A	V	N	L	Y	K	T	E	K	G	T	E	A	F	R	E	A	L	L	K	A	R	K	E	M	K	I
				K	D	L	A	P	E	Y	E	N	L	L	E	K	T	A	Y	E	E	E	G	F	T	I	T	N	L	D	L	A	R
				A	T	A	N	V	M	L	G	Q	K	I	S	K	E	D	A	E	K	Q	A	K	E	M	I	S	R	Q	I	K	M
				A	K	I	A	K	E	K	G	V	K	V	N	E	N	L	D	S	I	S	Q	F	Q	D	Y	Y	V	G	L	A	E
				K	V	R	D	E	V	K	P	T	D	D	E	L	M	K	F	F	N	A	N	R	E	K	Y	S	I	P	A	T	A
				D	A	K	L	V	F	I	S	V	K	S	A	K	E	D	D	D	L	A	K	E	K	A	E	K	L	L	S	E	L
				T	P	E	N	F	T	E	K	G	K	T	L	G	N	N	Q	D	I	I	Y	Q	D	L	G	T	F	G	T	K	A
				M	V	K	E	F	E	E	A	L	K	D	V	P	S	N	T	V	V	N	K	V	I	K	T	K	F	G	Y	H	V
				A	L	V	K	E	N	N	N	N	R	Q	W	G	V	E	H	I	L	I	V	P	Y	P	S	E	K	T	V	T	E
				K	L	E	K	L	N	K	I	K	A	D	I	E	A	G	T	L	A	L	N	D	K	I	D	E	D	A	I	Q	S
				F	D	A	K	G	I	T	P	D	G	I	I	P	D	F	V	Y	S	P	E	I	A	K	A	V	F	S	T	E	L
				N	K	V	G	I	I	N	P	N	K	A	T	I	I	I	F	Q	K	T	K	E	V	K	A	E	N	A	N	F	D
				K	S	K	E	Q	V	K	S	D	Y	V	N	K	K	V	A	E	Y	M	S	K	L	F							
				1				5					10					15					20					25					30
70	12.88%	99.0%	>sp|Q8RE44|RPOA_FUSNN DNA-directed RNA polymerase subunit alpha OS=Fusobacterium nucleatum subsp. nucleatum (strain ATCC 25586 / CIP 101130 / JCM 8532 / LMG 13131) OX=190304 GN=rpoA PE=3 SV=2																														
				M	L	K	I	E	K	Q	A	K	A	I	K	I	T	E	V	K	E	S	N	Y	K	G	Q	F	I	V	E	P	L
				Y	R	G	Y	G	N	T	L	G	N	A	L	R	R	V	L	L	S	S	I	P	G	A	A	I	K	G	M	R	I
				E	G	V	L	S	E	F	T	V	M	D	G	V	K	E	A	V	T	E	I	I	L	N	V	K	E	I	V	V	K
				A	E	S	S	G	E	R	R	M	S	L	S	I	K	G	P	K	V	V	K	A	A	D	I	V	A	D	I	G	L
				E	I	V	N	P	E	Q	V	I	C	T	V	T	T	D	R	T	L	D	I	E	F	I	V	D	T	G	E	G	F
				V	V	S	E	E	I	D	K	K	D	W	P	V	D	Y	I	A	V	D	A	I	Y	T	P	I	R	K	V	S	Y
				E	I	Q	D	T	M	F	G	R	M	T	D	F	D	K	L	T	L	N	V	E	T	D	G	S	I	E	I	R	D
				A	L	S	Y	A	V	E	L	L	K	L	H	L	D	P	F	L	E	I	G	N	K	M	E	N	L	R	D	D	I
				E	E	M	I	E	E	P	M	D	I	Q	V	I	D	D	K	S	H	D	M	K	I	E	E	L	D	L	T	V	R
				S	F	N	C	L	K	K	A	G	I	E	E	V	S	Q	L	A	S	L	S	L	N	E	L	L	K	I	K	N	L
				G	K	K	S	L	D	E	I	L	E	K	M	K	D	L	G	Y	D	L	E	K	N	G	S	P	E				
				1				5					10					15					20					25					30
71	8.21%	99.0%	>tr|D5RFQ4|D5RFQ4_FUSN2 Pyruvate kinase OS=Fusobacterium nucleatum subsp. nucleatum (strain ATCC 23726 / VPI 4351) OX=525283 GN=pyk PE=3 SV=1																														
				M	R	S	L	K	K	T	K	I	V	C	T	I	G	P	V	T	E	S	V	E	T	L	K	E	L	L	N	R	G
				M	N	V	M	R	L	N	F	S	H	G	D	Y	E	E	H	G	M	R	M	K	N	F	R	Q	A	M	S	E	T
				G	I	R	G	G	I	L	L	D	T	K	G	P	E	I	R	T	M	T	L	K	D	G	K	D	V	S	I	K	A
				G	Q	K	F	T	F	T	T	D	Q	S	V	V	G	D	N	E	R	V	A	V	T	Y	E	N	F	A	K	D	L
				K	V	G	N	M	V	L	V	D	D	G	L	L	E	L	D	V	V	E	I	K	G	N	E	V	I	C	I	A	R
				N	N	G	D	L	G	Q	K	K	G	I	N	L	P	N	V	S	V	N	L	P	A	L	S	E	K	D	I	E	D
				L	K	F	G	C	Q	N	N	V	D	F	V	A	A	S	F	I	R	K	A	D	D	V	R	Q	V	R	K	V	L
				R	E	N	G	G	E	R	I	Q	I	I	S	K	I	E	S	Q	E	G	L	D	N	F	D	E	I	L	A	E	S
				D	G	I	M	V	A	R	G	D	L	G	V	E	I	P	V	E	E	V	P	C	A	Q	K	M	M	I	R	K	C
				N	R	V	G	K	T	V	I	T	A	T	Q	M	L	D	S	M	I	K	N	P	R	P	T	R	A	E	A	N	D
				V	A	N	A	I	L	D	G	T	D	A	V	M	L	S	G	E	T	A	K	G	K	Y	P	L	A	A	V	E	V
				M	N	K	I	A	K	K	V	D	A	T	I	P	P	F	Y	I	E	G	V	I	N	K	N	D	I	T	S	A	V
				A	E	G	S	A	D	I	S	E	R	L	N	A	K	L	I	V	V	G	T	E	S	G	R	A	A	R	D	M	R
				R	Y	F	P	R	A	H	I	L	A	I	T	N	N	E	K	T	A	N	Q	L	V	L	S	R	G	I	I	S	Y
				V	D	A	S	P	K	T	L	E	E	F	F	V	V	A	E	S	V	A	K	K	L	N	L	V	K	N	N	D	I
				I	I	A	T	C	G	E	S	V	F	I	Q	G	T	T	N	S	I	K	V	I	Q	V	K	A					
				1				5					10					15					20					25					30
72	5.48%	99.0%	>tr|R9RAT0|R9RAT0_FUSNU Uncharacterized protein OS=Fusobacterium nucleatum subsp. animalis 4_8 OX=469607 GN=HMPREF0409_00958 PE=4 SV=1																														
				M	K	K	V	L	S	L	I	F	L	S	L	F	T	L	L	L	V	A	C	G	G	K	K	E	E	A	T	K	N
				E	G	E	T	K	K	E	A	R	V	I	K	V	T	T	K	F	V	D	D	E	Q	T	A	K	S	L	V	K	V
				V	E	A	I	N	K	R	S	N	G	S	L	E	L	Q	L	F	T	S	G	T	L	P	I	G	K	D	G	M	E
				Q	V	A	N	G	S	D	W	I	L	V	D	G	V	N	F	L	G	D	Y	V	P	D	Y	N	A	V	T	G	P
				M	L	Y	Q	S	F	D	E	Y	L	R	M	V	R	T	P	L	V	Q	D	L	N	A	Q	A	L	E	K	G	I
				K	V	L	S	L	D	W	L	F	G	F	R	N	I	E	A	K	K	A	I	K	T	P	E	D	M	K	G	L	K
				L	R	V	P	T	S	Q	L	Y	T	F	T	I	E	A	M	G	G	N	P	V	A	M	P	Y	P	D	T	Y	A
				A	L	Q	Q	G	V	I	D	G	L	E	G	S	I	L	S	Y	Y	G	T	K	Q	Y	E	N	V	K	E	Y	S
				L	T	R	H	L	L	G	V	S	A	V	C	I	S	K	K	C	W	D	S	L	T	D	E	E	R	T	I	I	Q
				E	E	F	D	K	G	A	E	D	N	L	T	E	T	K	K	L	E	D	E	Y	A	Q	K	L	K	D	N	G	V
				T	F	H	E	V	D	A	E	A	F	N	K	A	V	A	P	V	Y	E	K	F	P	K	W	T	P	G	I	Y	D
				K	I	M	E	N	L	T	Q	I	R	E	D	I	K	N	G	K													
				1				5					10					15					20					25					30
73	7.84%	96.2%	>tr|A0A1S1MKB1|A0A1S1MKB1_FUSNU Fe(3+) ions import ATP-binding protein FbpC OS=Fusobacterium nucleatum OX=851 GN=fbpC PE=3 SV=1																														
				M	S	V	N	I	K	I	E	N	A	Q	K	R	Y	G	D	N	I	I	I	E	N	L	S	L	D	I	K	Q	G
				E	F	F	T	L	L	G	P	S	G	C	G	K	T	T	L	L	R	M	I	A	G	F	N	S	I	E	K	G	D
				F	Y	F	N	E	K	R	I	N	D	L	D	P	A	K	R	N	I	G	M	V	F	Q	N	Y	A	I	F	P	H
				L	T	V	E	Q	N	V	E	F	G	L	K	N	R	K	V	S	K	E	E	M	K	T	E	T	E	K	F	L	K
				L	M	Q	I	D	E	Y	R	D	R	M	P	E	R	L	S	G	G	Q	Q	Q	R	V	A	L	A	R	A	L	V
				I	K	P	D	V	L	L	M	D	E	P	L	S	N	L	D	A	K	L	R	V	E	M	R	T	A	I	K	E	I
				Q	N	S	I	G	I	T	T	V	Y	V	T	H	D	Q	E	E	A	M	A	V	S	D	R	I	A	V	M	K	D
				G	E	I	Q	H	L	G	Q	P	K	D	I	Y	Q	R	P	A	N	L	F	V	A	T	F	I	G	K	T	N	V
				L	N	G	N	L	N	G	S	I	L	K	V	A	G	K	Y	D	I	T	L	N	N	I	K	D	K	N	I	K	G
				N	V	V	I	S	I	R	P	E	E	F	V	I	D	E	N	Q	I	K	D	G	I	K	A	F	I	D	S	S	V
				F	L	G	L	N	T	H	Y	F	A	H	L	E	S	G	E	K	I	E	I	V	Q	E	S	K	I	D	S	I	I
				P	K	G	A	E	V	Y	L	K	I	K	Q	D	K	I	N	V	F	T	E	D	G	S	K	N	I	L	D	G	V

				N	N	D	I	G	V	A	Y	A	K																				
				1				5					10					15					20					25					30
74	8.20%	99.0%	>sp|Q8RFY0|RECA_FUSNN Protein RecA OS=Fusobacterium nucleatum subsp. nucleatum (strain ATCC 25586 / CIP 101130 / JCM 8532 / LMG 13131) OX=190304 GN=recA PE=3 SV=2																														
				M	A	A	K	K	D	K	S	V	P	D	S	K	I	T	D	K	E	G	K	E	K	A	V	K	D	A	M	A	A
				I	T	K	G	F	G	S	G	L	I	M	K	L	G	E	K	S	S	M	N	V	E	S	I	P	T	G	S	I	N
				L	D	I	A	L	G	I	G	G	V	P	K	G	R	I	I	E	I	Y	G	A	E	S	S	G	K	T	T	L	A
				L	H	V	I	A	E	A	Q	K	Q	G	G	T	V	A	F	I	D	A	E	H	A	L	D	P	V	Y	A	K	A
				L	G	V	D	I	D	E	L	L	I	S	Q	P	D	Y	G	E	Q	A	L	E	I	A	D	T	L	V	R	S	G
				A	I	D	L	I	V	I	D	S	V	A	A	L	V	P	K	A	E	I	D	G	E	M	S	D	Q	Q	M	G	L
				Q	A	R	L	M	S	K	G	L	R	K	L	T	G	N	L	N	K	Y	K	T	T	M	I	F	I	N	Q	I	R
				E	K	I	G	V	T	Y	G	P	T	T	T	T	T	G	G	K	A	L	K	F	Y	S	S	V	R	M	E	V	K
				K	M	G	T	V	K	Q	G	D	D	P	I	G	S	E	V	I	V	K	V	T	K	N	K	V	A	P	P	F	K
				E	A	A	F	E	I	L	Y	G	K	G	I	S	K	V	G	E	I	I	D	A	A	V	A	K	D	V	I	V	K
				A	G	S	W	F	S	F	R	D	Q	S	I	G	Q	G	K	E	K	V	R	A	E	L	E	I	N	P	E	L	L
				A	Q	V	E	K	D	L	K	E	A	I	A	K	G	P	V	D	K	K	K	K	K	S	K	K	E	A	S	S	D
				D	T	D	D	E	N	L	E	I	D	D	A	I	D	E	N	N	D												
				1				5					10					15					20					25					30
75	10.51%	99.0%	>tr|Q8RGC9|Q8RGC9_FUSNN Iron(III)-binding protein OS=Fusobacterium nucleatum subsp. nucleatum (strain ATCC 25586 / CIP 101130 / JCM 8532 / LMG 13131) OX=190304 GN=FN0375 PE=4 SV=1																														
				M	K	K	F	I	K	F	L	L	M	S	M	G	M	I	F	M	L	V	A	C	G	G	D	K	E	K	T	E	A
				T	P	E	T	Q	G	S	N	E	L	V	I	Y	S	P	N	A	D	D	E	V	N	K	I	I	P	A	F	E	E
				A	T	G	I	K	V	I	L	Q	S	M	G	S	G	D	V	L	A	R	I	S	A	E	K	E	N	P	Q	A	D
				I	N	W	G	A	I	S	M	G	V	L	A	T	T	P	D	L	W	E	S	Y	T	S	E	N	E	K	N	V	P
				D	A	Y	K	N	T	T	G	F	F	T	N	Y	K	L	D	G	S	A	A	L	L	V	N	K	D	V	F	K	K
				L	G	L	D	P	E	K	F	N	G	Y	K	D	L	L	W	P	E	L	K	G	K	I	A	M	G	D	P	T	A
				S	S	S	A	I	A	E	L	T	N	M	L	L	V	M	G	E	K	P	Y	D	E	K	A	W	E	F	I	E	K
				F	I	G	Q	L	D	G	T	I	L	S	S	S	S	Q	I	Y	K	A	T	A	D	G	E	Y	A	V	G	V	T
				Y	E	N	P	A	V	T	L	L	Q	D	G	A	T	N	L	K	L	V	Y	P	E	E	G	S	V	W	L	P	G
				A	A	A	I	V	K	N	A	P	H	M	E	N	A	K	K	F	I	D	F	L	I	S	D	E	G	Q	K	V	V
				A	E	T	S	T	R	P	V	N	T	S	I	K	N	T	S	E	F	I	R	P	F	D	E	I	K	V	A	Y	E
				D	I	P	Y	C	A	E	H	R	K	E	W	Q	E	R	W	T	N	I	L	T	K								
				1				5					10					15					20					25					30
76	9.19%	98.5%	>tr|H1HG01|H1HG01_FUSNU Transcription termination/antitermination protein NusA OS=Fusobacterium nucleatum subsp. animalis F0419 OX=999414 GN=nusA PE=3 SV=1																														
				M	K	A	K	D	S	K	N	F	L	E	A	L	D	E	L	E	K	E	K	G	I	S	K	E	S	V	L	E	A
				I	E	L	A	L	L	A	A	Y	K	K	N	Y	G	E	D	E	N	V	E	V	T	V	D	R	E	N	G	D	I
				K	V	F	A	S	K	T	V	V	A	A	D	D	L	L	D	P	N	E	E	I	S	L	E	D	A	K	K	I	K
				K	R	V	K	I	G	D	I	L	K	F	E	V	D	C	E	N	F	R	R	N	A	V	Q	N	G	K	Q	I	V
				I	Q	K	V	R	E	A	E	R	E	H	I	F	N	K	F	K	E	R	E	D	S	I	V	T	G	I	I	R	R
				I	D	N	R	K	N	I	F	I	E	I	D	G	V	E	L	I	L	P	P	A	E	Q	S	V	S	D	I	Y	R
				V	G	E	R	I	K	V	Y	V	L	S	V	E	K	T	S	K	F	P	K	I	L	I	S	R	R	N	E	G	L
				L	R	K	L	F	E	I	E	I	P	E	I	T	S	G	I	I	E	I	K	S	V	A	R	E	A	G	S	R	A
				K	V	A	V	Y	S	A	V	P	N	I	D	T	I	G	A	C	I	G	Q	K	G	A	R	I	K	N	I	V	D
				E	L	N	G	E	R	I	D	I	V	E	W	K	P	V	V	E	E	F	V	S	A	V	L	S	P	A	V	V	E
				S	V	T	I	L	E	D	G	T	A	R	V	L	V	E	P	S	Q	L	S	L	A	I	G	K	N	G	Q	N	A
				R	L	A	A	R	L	T	G	M	R	V	D	I	K	V	I	D	K	D	K	E	T	L	K	E	G	E	D	E	
				1				5					10					15					20					25					30
77	9.18%	99.0%	>tr|J8VGK2|J8VGK2_9FUSO Uncharacterized protein OS=Fusobacterium hwasookii ChDC F128 OX=1216362 GN=B437_09730 PE=4 SV=1																														
				M	F	Y	I	P	F	F	V	L	L	V	I	L	I	A	I	V	M	F	K	A	V	K	I	V	P	E	S	Q	V
				Y	I	V	E	K	L	G	K	Y	Y	Q	S	L	S	S	G	L	S	F	I	N	P	F	F	D	K	V	S	R	I
				V	S	L	K	E	Q	V	V	D	F	D	P	Q	A	V	I	T	K	D	N	A	T	M	Q	I	D	T	V	V	Y
				F	Q	I	T	D	P	K	L	Y	T	Y	G	V	E	R	P	L	S	A	I	E	N	L	T	A	T	T	L	R	N
				I	I	G	D	M	T	V	D	E	T	L	T	S	R	D	I	I	N	T	K	M	R	Q	E	L	D	D	A	T	D
				P	W	G	I	K	V	N	R	V	E	L	K	S	I	L	P	P	N	D	I	R	I	A	M	E	K	E	M	K	A
				E	R	E	K	R	A	K	I	L	E	A	Q	A	T	R	E	S	A	I	L	V	A	E	G	E	K	Q	S	A	I
				L	R	A	E	A	E	K	E	V	K	I	K	E	A	E	G	R	A	Q	A	I	L	E	I	Q	K	A	E	A	E
				A	I	K	V	L	N	E	A	K	P	S	K	E	I	L	A	L	K	S	F	E	T	F	E	K	V	A	D	G	K
				S	T	K	I	L	I	P	S	E	I	Q	N	L	A	G	F	M	Q	A	I	K	E	I	K						
				1				5					10					15					20					25					30
78	7.77%	98.9%	>tr|Q8RIC2|Q8RIC2_FUSNN LPS biosynthesis protein WbpG OS=Fusobacterium nucleatum subsp. nucleatum (strain ATCC 25586 / CIP 101130 / JCM 8532 / LMG 13131) OX=190304 GN=FN1679 PE=4 SV=1																														
				M	K	Y	C	K	N	C	L	Q	P	D	T	R	P	G	I	K	F	N	E	D	G	V	C	Y	A	C	L	Y	E
				K	E	K	E	K	I	D	W	K	S	R	E	K	E	L	K	E	I	A	E	W	A	K	K	T	S	K	G	P	Y
				D	C	V	I	G	V	S	G	G	K	D	S	T	F	Q	A	I	Y	A	K	E	K	L	G	L	H	V	L	L	V
				N	G	E	P	D	Q	I	T	E	I	G	R	K	N	I	E	N	L	I	Q	K	G	F	D	I	I	K	L	R	P
				N	P	K	I	T	K	Q	L	V	K	T	A	F	L	K	Y	G	N	P	M	K	S	T	E	Y	P	L	W	T	S
				A	Y	I	I	A	D	K	F	D	I	P	L	I	I	Q	G	E	N	A	A	L	T	L	G	V	V	N	T	K	L
				G	V	D	G	N	A	M	N	V	D	G	G	N	T	L	N	G	C	N	A	S	D	W	V	E	D	G	L	P	L
				E	D	L	Y	M	Y	Q	F	P	D	K	E	K	M	A	K	K	G	I	K	A	I	W	L	Q	Y	Y	A	K	E
				W	S	Q	V	Y	N	A	D	F	S	I	A	R	G	L	L	G	R	T	V	E	D	L	H	D	L	G	R	Y	R
				R	Y	S	S	L	D	S	N	L	H	I	V	N	Q	M	L	K	Y	Y	K	F	G	F	G	F	A	T	D	E	A
				C	Y	D	I	R	E	G	R	L	T	R	D	D	A	I	W	L	I	N	E	Y	D	G	K	C	G	K	Q	Y	I
				D	E	F	C	K	Y	I	E	I	T	E	E	E	F	W	N	T	V	D	Q	Y	V	N	K	E	L	F	E	K	K
				D	G	K	W	I	P	K	F	K	V	G	K	N																	
				1				5					10					15					20					25					30
79	13.27%	99.0%	>sp|Q8RIF5|RL3_FUSNN 50S ribosomal protein L3 OS=Fusobacterium nucleatum subsp. nucleatum (strain ATCC 25586 / CIP 101130 / JCM 8532 / LMG 13131) OX=190304 GN=rplC PE=3 SV=1																														
				M	S	G	I	L	G	K	K	I	G	M	T	Q	I	F	E	D	G	K	F	V	P	V	T	V	V	E	A	G	P
				N	F	V	L	Q	K	K	T	E	E	K	D	G	Y	V	A	L	Q	L	G	F	D	E	K	K	E	K	N	T	T
				K	P	L	M	G	I	F	N	K	A	G	V	K	P	Q	R	F	V	K	E	L	E	V	E	S	V	D	G	Y	E
				L	G	Q	E	I	K	V	D	V	L	T	E	V	G	Y	V	D	I	T	G	T	S	K	G	K	G	T	S	G	V
				M	K	K	H	G	F	S	G	N	R	A	S	H	G	V	S	R	N	H	R	L	G	G	S	I	G	M	S	S	W
				P	G	K	V	L	K	G	K	K	M	A	G	Q	H	G	N	A	T	V	T	V	Q	N	L	K	V	V	K	V	D
				A	E	H	N	L	L	L	I	K	G	A	V	P	G	A	K	N	S	Y	L	V	I	K	P	A	V	K	K	V	I
				G																													
				1				5					10					15					20					25					30
80	5.71%	99.0%	>tr|Q8RE65|Q8RE65_FUSNN C4-dicarboxylate-binding protein OS=Fusobacterium nucleatum subsp. nucleatum (strain ATCC 25586 / CIP 101130 / JCM 8532 / LMG 13131) OX=190304 GN=FN1258 PE=1 SV=1																														
				M	A	L	V	A	C	G	G	K	K	E	E	A	T	K	E	S	G	D	A	K	Q	E	A	R	V	I	K	V	T
				T	K	F	V	D	D	E	Q	T	A	K	S	L	V	K	V	V	E	A	I	N	Q	R	S	N	G	T	L	E	L
				Q	L	F	T	S	G	T	L	P	I	G	K	D	G	M	E	Q	V	A	N	G	S	D	W	I	L	V	D	G	V
				N	F	L	G	D	Y	V	P	D	Y	N	A	V	T	G	P	M	L	Y	Q	S	F	E	E	Y	L	R	M	V	K
				T	P	L	V	Q	D	L	N	A	Q	A	L	E	K	G	I	K	V	L	S	L	D	W	L	F	G	F	R	N	I
				E	A	K	K	P	I	K	T	P	E	D	M	K	G	L	K	L	R	V	P	T	S	Q	L	Y	T	F	T	I	E
				A	M	G	G	N	P	V	A	M	P	Y	P	D	T	Y	A	A	L	Q	Q	G	V	I	D	G	L	E	G	S	I
				L	S	F	Y	G	T	K	Q	Y	E	N	V	K	E	Y	S	L	T	R	H	L	L	G	V	S	A	V	C	I	S
				K	K	C	W	D	S	L	T	D	E	Q	R	T	I	I	Q	E	E	F	D	K	G	A	L	D	N	L	T	E	T
				E	K	L	E	D	E	Y	A	Q	K	L	K	D	N	G	V	T	F	H	E	V	D	A	E	A	F	N	K	A	V
				A	P	V	Y	D	K	F	P	K	W	T	P	G	I	Y	D	K	I	M	E	N	L	T	Q	I	R	E	D	I	K
				N	G	K																											
				1				5					10					15					20					25					30
81	4.39%	99.0%	>sp|Q8RHB5|SYFB_FUSNN Phenylalanine--tRNA ligase beta subunit OS=Fusobacterium nucleatum subsp. nucleatum (strain ATCC 25586 / CIP 101130 / JCM 8532 / LMG 13131) OX=190304 GN=pheT PE=3 SV=1																														
				M	L	I	S	L	N	W	L	K	Q	Y	V	D	I	K	E	S	I	D	E	I	A	N	A	L	T	M	I	G	Q
				E	V	E	A	I	D	I	Q	G	K	D	L	D	N	V	V	I	G	Q	I	V	E	F	D	K	H	P	N	S	D
				R	L	T	L	L	K	V	N	V	G	G	E	E	P	L	Q	I	I	C	G	A	K	N	H	K	L	N	D	K	V
				V	V	A	K	I	G	A	V	L	P	G	N	F	K	I	K	K	S	K	I	R	D	V	E	S	Y	G	M	L	C
				S	E	A	E	L	G	F	T	K	E	S	E	G	I	I	I	L	P	E	D	A	P	I	G	T	E	Y	R	E	Y
				M	N	L	N	D	V	I	F	E	L	E	I	T	P	N	R	P	D	C	L	S	H	I	G	I	A	R	E	V	A
				A	Y	Y	N	R	K	V	K	Y	P	M	I	E	I	T	E	T	I	E	S	I	N	T	M	V	K	V	D	I	E
				D	K	D	R	C	K	R	Y	M	G	R	V	I	K	N	V	K	V	Q	E	S	P	D	W	L	K	S	R	I	R
				A	M	G	L	N	P	I	N	N	I	V	D	I	T	N	F	V	M	F	E	Y	N	Q	P	M	H	A	F	D	L
				D	K	L	E	G	N	I	T	I	R	A	A	K	K	N	E	E	I	T	T	L	D	G	V	D	R	V	L	K	N
				G	E	L	V	I	A	D	D	E	K	A	I	A	I	A	G	V	I	G	G	Q	N	T	Q	I	D	N	E	T	K
				N	I	F	V	E	V	A	Y	F	T	P	E	N	I	R	K	T	S	R	E	L	G	I	F	T	D	S	A	Y	R
				N	E	R	G	M	D	V	E	N	L	S	N	V	M	A	R	A	V	S	L	I	A	E	V	A	G	G	D	V	L
				S	E	V	I	D	K	Y	V	E	K	P	Q	R	A	E	I	S	L	N	L	E	K	L	N	K	F	I	G	K	N
				L	T	Y	D	E	V	G	K	I	L	T	H	L	D	I	E	L	K	P	L	G	E	G	T	M	L	L	I	P	P
				S	Y	R	A	D	L	T	R	P	A	D	I	Y	E	E	V	I	R	M	Y	G	F	D	N	I	E	A	K	I	P
				V	M	S	I	E	S	G	E	E	N	I	N	F	K	M	P	R	I	V	R	G	I	L	K	E	L	G	L	N	E
				V	I	N	Y	S	F	I	P	K	F	T	K	E	L	F	N	F	G	D	E	V	I	E	I	K	N	P	L	S	E
				D	M	A	I	M	R	P	T	L	L	Y	S	L	I	T	N	I	R	D	N	I	N	R	N	Q	T	D	L	K	L
				F	E	I	S	K	T	F	K	N	L	G	A	E	K	D	G	L	A	I	E	D	L	K	V	G	I	I	L	S	G
				R	E	D	K	N	L	W	N	Q	S	K	T	D	Y	N	F	Y	D	L	K	G	Y	L	E	F	L	L	E	R	L
				N	V	T	K	Y	S	L	T	R	L	E	N	S	N	F	H	P	G	A	S	A	E	I	K	I	G	E	D	I	I
				G	V	F	G	E	L	H	P	N	L	V	N	Y	F	G	I	K	R	E	K	L	F	F	A	E	L	N	L	T	K
				L	L	K	Y	I	K	I	K	V	N	Y	E	S	I	S	K	Y	P	E	V	L	R	D	L	A	I	T	L	D	R
				D	I	L	V	G	D	M	I	K	E	I	K	K	K	V	A	L	I	E	K	I	D	I	F	D	V	Y	S	G	D
				K	I	D	K	D	K	K	S	V	A	M	S	I	I	L	R	D	K	N	R	T	L	T	D	G	D	I	D	T	A
				M	N	T	I	L	E	L	I	K	D	K	Y	N	G	E	I	R	K												
				1				5					10					15					20					25					30
82	6.80%	98.2%	>tr|X7RVI8|X7RVI8_FUSNU Uncharacterized protein OS=Fusobacterium nucleatum 13_3C OX=1357398 GN=HMPREF2085_01916 PE=4 SV=1																														
				M	N	S	I	R	K	L	V	K	F	L	I	D	I	F	L	L	N	L	S	L	I	I	S	I	F	L	K	Y	D
				Q	I	Q	M	T	D	R	N	I	N	F	F	I	Y	Y	N	L	S	F	C	I	I	Y	F	I	L	K	I	Y	N
				N	S	W	R	F	S	G	I	S	E	Y	T	T	L	I	I	L	S	V	T	T	T	T	F	S	Y	I	L	R	E
				F	L	K	L	D	T	K	S	S	L	Y	F	E	T	L	I	I	F	T	F	L	L	I	L	S	R	F	L	M	F
				L	T	R	M	K	G	I	I	K	K	D	L	D	Q	E	N	V	L	I	Y	G	A	G	E	S	G	V	L	L	V
				K	E	S	S	I	N	P	N	F	P	Y	R	I	I	G	F	L	D	D	N	P	N	K	I	G	G	K	V	Y	G

				L	R	V	F	G	G	L	N	K	V	K	E	I	V	E	K	K	N	I	S	K	I	I	I	S	M	P	S	V	S
				Q	N	K	I	S	N	I	L	K	E	I	N	K	I	Q	D	L	S	V	K	I	L	P	N	V	D	N	L	I	E
				E	G	S	L	T	T	Q	L	R	N	I	K	L	E	D	L	L	G	R	E	E	I	K	I	N	T	K	E	V	M
				G	F	I	E	D	K	V	I	F	V	T	G	G	G	G	S	I	G	S	E	L	I	N	Q	I	A	K	Y	N	P
				K	K	I	I	N	I	E	I	N	E	N	A	S	Y	L	M	E	L	E	L	K	R	N	Y	P	F	L	D	Y	K
				T	E	I	A	S	V	R	D	S	E	K	L	D	I	L	F	N	K	Y	K	P	D	I	L	F	H	A	A	A	H
				K	H	V	P	L	M	E	N	N	P	E	E	A	I	K	N	N	I	F	G	T	K	N	I	A	E	C	C	L	K
				Y	K	L	E	S	V	V	L	I	S	T	D	K	A	V	N	P	T	N	V	M	G	A	T	K	R	V	C	E	M
				I	F	Q	K	Y	S	E	K	S	S	N	T	K	F	M	A	V	R	F	G	N	V	L	G	S	N	G	S	V	I
				P	I	F	S	K	L	I	E	E	G	K	N	L	T	L	T	H	K	D	I	I	R	Y	F	M	T	I	P	E	A
				A	Q	L	V	I	E	A	A	T	I	G	K	G	G	E	I	L	I	L	D	M	G	E	P	V	K	I	Y	D	L
				A	K	N	M	I	K	L	S	G	S	N	V	G	I	D	I	V	G	L	R	P	G	E	K	L	F	E	E	L	L
				Y	D	I	N	S	S	E	K	T	S	N	N	K	I	Y	I	T	N	M	E	N	E	K	V	Q	V	D	I	D	N
				Y	Y	T	I	L	K	D	L	I	K	E	N	D	V	I	G	M	R	R	T	L	A	N	I	I	G	T	F	K	G
				R	V	E																											
				1				5					10					15					20					25					30
83	3.87%	97.9%	>sp|Q8RG14|SYR_FUSNN Arginine--tRNA ligase OS=Fusobacterium nucleatum subsp. nucleatum (strain ATCC 25586 / CIP 101130 / JCM 8532 / LMG 13131) OX=190304 GN=argS PE=3 SV=1																														
				M	K	I	T	S	R	E	L	T	D	I	F	Q	K	H	V	E	N	L	F	P	N	K	E	L	K	P	V	E	I
				T	V	A	T	N	E	N	F	G	D	Y	Q	C	N	F	A	M	I	N	S	K	I	I	G	D	N	P	R	K	I
				A	E	E	V	K	N	N	F	P	Y	G	D	V	I	E	K	L	E	V	A	G	P	G	F	I	N	I	F	L	S
				D	K	Y	I	S	N	S	I	K	K	I	G	E	D	Y	D	F	S	F	L	N	R	K	G	K	V	I	I	D	F
				S	S	P	N	I	A	K	R	M	H	I	G	H	L	R	S	T	I	I	G	E	S	I	S	R	I	Y	R	F	L
				G	Y	D	V	V	A	D	N	H	I	G	D	W	G	T	Q	F	G	K	L	I	V	G	Y	R	N	W	L	D	K
				K	A	Y	K	K	N	A	I	E	E	L	E	R	V	Y	V	K	F	S	D	E	A	E	K	D	P	S	L	E	D
				L	A	R	A	E	L	K	K	V	Q	D	G	E	E	E	N	T	K	L	W	K	E	F	I	T	E	S	L	K	E
				Y	N	K	L	Y	K	R	L	D	V	H	F	D	T	Y	Y	G	E	S	F	Y	N	D	M	M	A	D	V	V	K
				E	L	V	D	K	K	I	A	V	D	D	D	G	A	K	V	V	F	F	D	E	K	D	N	L	F	P	C	I	V
				Q	K	K	D	G	A	Y	L	Y	S	T	S	D	I	A	T	V	K	F	R	K	N	T	Y	D	V	N	R	M	I
				Y	L	T	D	A	R	Q	Q	D	H	F	K	Q	F	F	K	I	T	D	M	L	G	W	N	I	E	K	Y	H	I
				W	F	G	I	I	R	F	A	D	G	I	L	S	T	R	K	G	N	V	I	K	L	E	E	L	L	D	E	A	H
				S	R	A	Y	D	V	V	N	E	K	N	P	N	L	S	E	G	E	K	Q	N	I	A	E	V	V	G	V	S	S
				V	K	Y	A	D	L	S	Q	N	K	Q	S	D	I	I	F	E	W	D	K	M	L	S	F	E	G	N	T	A	P
				Y	L	L	Y	T	Y	A	R	I	Q	S	I	L	R	K	V	A	E	L	N	I	G	L	N	E	N	I	E	I	K
				T	E	N	K	I	E	K	S	L	A	T	Y	L	L	A	F	P	I	S	V	L	K	A	G	E	T	F	K	P	N
				L	I	A	D	Y	L	Y	E	L	S	K	K	L	N	S	F	Y	N	N	C	P	I	L	N	Q	D	I	E	T	L
				K	S	R	A	L	L	I	K	K	T	G	E	V	L	K	E	G	L	E	L	L	G	I	P	I	L	N	K	M	
				1				5					10					15					20					25					30
84	6.35%	98.1%	>sp|Q8RFX6|SDHD_FUSNN Probable D-serine dehydratase OS=Fusobacterium nucleatum subsp. nucleatum (strain ATCC 25586 / CIP 101130 / JCM 8532 / LMG 13131) OX=190304 GN=dsdA PE=3 SV=1																														
				M	D	I	K	N	M	I	I	N	N	P	L	I	K	N	M	I	D	K	K	E	V	G	W	T	N	P	K	E	M
				N	Y	T	E	Y	E	K	K	L	P	L	K	D	Q	E	L	K	E	A	E	E	R	L	K	R	F	A	P	F	I
				K	K	V	F	P	E	T	E	E	T	Y	G	I	I	E	S	P	L	E	E	I	F	N	M	Q	K	E	L	E	K
				K	Y	H	T	E	I	L	G	K	L	Y	L	K	M	D	S	H	L	P	V	A	G	S	I	K	A	R	G	G	V
				Y	E	V	L	K	H	A	E	E	L	A	M	E	A	G	L	L	K	L	E	D	D	Y	S	I	L	A	D	K	K
				F	K	D	F	F	S	K	Y	K	I	Q	V	G	S	T	G	N	L	G	L	S	I	G	I	T	S	A	A	L	G
				F	Q	V	I	V	H	M	S	A	D	A	K	K	W	K	K	D	M	L	R	S	K	G	V	Q	V	I	E	Y	E
				S	D	Y	G	K	A	V	E	E	G	R	K	N	S	D	A	D	P	M	S	Y	F	V	D	D	E	K	S	M	N
				L	F	L	G	Y	T	V	A	A	S	R	I	K	K	Q	F	D	K	K	G	I	V	I	N	K	E	H	P	L	I
				V	Y	I	P	C	G	V	G	G	A	P	G	G	V	A	Y	G	L	K	R	I	F	K	E	N	V	Y	I	F	F
				V	E	P	V	L	A	P	C	M	L	L	G	M	Q	T	G	L	H	E	K	I	S	V	Y	D	V	G	I	H	G
				I	T	H	A	D	G	L	A	V	A	R	P	S	G	L	V	G	R	L	M	E	P	I	L	S	G	I	F	T	V
				D	D	Y	K	L	Y	D	Y	L	R	I	L	N	E	T	E	N	K	R	I	E	P	S	S	C	A	A	F	E	G
				V	V	S	L	L	K	Y	E	D	S	K	K	Y	I	E	N	R	I	G	K	N	I	N	N	V	Y	H	V	C	W
				A	T	G	G	K	M	V	P	Q	E	D	M	E	I	F	L	N	T	Y	L	K									
				1				5					10					15					20					25					30
85	6.67%	97.1%	>tr|A0A0M3UX89|A0A0M3UX89_FUSNC Uncharacterized protein OS=Fusobacterium nucleatum subsp. nucleatum OX=76856 GN=RO03_10820 PE=4 SV=1																														
				M	K	K	F	F	L	T	I	L	F	C	I	L	S	I	F	T	F	A	N	D	W	E	F	G	S	E	G	E	H
				I	I	P	L	K	G	S	N	M	S	I	K	K	E	K	I	T	L	K	L	T	P	D	G	M	L	V	N	V	K
				F	T	F	D	N	P	T	A	E	N	K	I	I	G	F	V	T	P	E	S	G	S	G	G	E	G	E	G	K	G
				N	R	K	P	E	P	L	N	I	K	S	F	K	T	T	V	N	G	K	E	V	K	S	N	V	E	L	L	S	K
				L	L	S	K	G	V	L	D	N	N	V	I	K	E	Y	T	E	K	E	K	N	F	Y	N	Y	V	Y	Y	F	N
				A	D	F	K	Q	G	E	N	V	V	E	H	N	Y	F	Y	T	G	S	Y	G	V	Y	E	R	D	F	S	Y	V
				V	T	T	I	S	K	W	K	N	K	T	V	E	D	F	E	I	E	V	Q	P	G	N	Y	F	V	K	L	P	Y
				S	F	W	K	N	N	N	K	I	N	W	E	I	V	G	K	G	K	M	V	T	I	A	P	T	K	K	P	N	D
				E	D	A	N	G	I	E	K	F	G	V	I	Y	L	K	L	D	N	G	F	V	R	Y	K	A	K	N	F	S	P
				T	D	D	F	Y	M	V	R	M	D	N	I	L	G	F	G	Y	E	F	P	E	G	K	V	Q	G	Y	K	F	K
				D	K	Y	F	D	I	A	A	R	G	N	S	Y	G	D	T	G	F	I	K	E	Y	Q	Q	D	L	N	D	K	D
				L	D	I	I	R	N	Y	P	Y	A	L	A	G	Y	D	F	D	K	K	D	L	K	D	Y	F	S	Q	F	I	W
				Y	S	P	V	S	K	N	V	K	I	D	S	D	S	N	N	I	I	K	A	V	D	E	I	N	T	K	R	K	K
				1				5					10					15					20					25					30
86	3.84%	97.9%	>sp|Q8RIA1|PNP_FUSNN Polyribonucleotide nucleotidyltransferase OS=Fusobacterium nucleatum subsp. nucleatum (strain ATCC 25586 / CIP 101130 / JCM 8532 / LMG 13131) OX=190304 GN=pnp PE=3 SV=1																														
				M	K	E	L	M	F	D	E	K	I	M	E	L	E	L	A	G	R	T	L	K	V	S	T	G	K	I	S	R	Q
				S	S	G	A	I	V	I	Q	Y	G	D	T	V	L	L	S	T	A	N	R	S	K	E	A	R	K	G	A	D	F
				F	P	L	T	V	D	Y	I	E	K	F	Y	S	S	G	K	F	P	G	G	F	N	K	R	E	G	R	P	S	T
				N	A	T	L	V	A	R	L	I	D	R	P	I	R	P	M	F	P	D	G	F	N	Y	D	V	H	I	V	N	T
				V	L	S	Y	D	E	I	N	I	P	D	Y	L	G	V	I	G	S	S	L	A	L	M	I	S	D	I	P	F	L
				G	P	V	A	S	V	I	V	G	Y	K	N	G	E	F	I	L	N	P	S	P	K	E	L	E	E	S	E	L	D
				L	I	V	A	G	T	K	E	A	V	N	M	V	E	A	G	A	K	E	L	D	E	E	T	M	L	K	A	I	M
				F	A	H	E	N	I	K	K	I	C	E	F	Q	E	E	F	S	K	L	Y	G	K	E	N	I	E	F	E	K	P
				E	V	L	T	L	V	K	D	F	I	D	T	N	G	Y	E	R	L	Q	Q	A	V	L	T	T	G	K	K	N	R
				E	D	A	V	D	S	L	E	E	E	L	M	E	K	F	I	Q	E	N	Y	P	D	V	P	E	E	E	L	P	E
				D	I	I	L	E	F	K	T	Y	Y	H	D	L	M	K	K	L	V	R	E	A	I	L	Y	H	K	H	R	V	D
				G	R	T	T	T	E	I	R	P	L	D	A	Q	I	N	V	L	P	I	P	H	G	S	A	L	F	T	R	G	E
				T	Q	S	L	A	I	T	T	L	G	T	K	E	D	E	Q	L	I	D	D	L	E	K	E	Y	Y	K	K	F	Y
				L	H	Y	N	F	P	P	Y	S	V	G	E	V	G	R	M	G	S	P	G	R	R	E	L	G	H	G	S	L	A
				E	R	A	L	S	Y	V	I	P	T	E	E	E	F	P	Y	T	I	R	V	V	S	E	I	T	E	S	N	G	S
				S	S	Q	A	S	I	C	G	G	S	L	S	L	M	S	A	G	V	P	I	K	E	H	V	A	G	I	A	M	G
				L	I	K	E	G	E	E	F	T	V	L	T	D	I	M	G	L	E	D	H	L	G	D	M	D	F	K	V	A	G
				T	K	S	G	I	T	A	L	Q	M	D	I	K	I	T	G	I	T	E	E	I	M	R	I	A	L	N	Q	A	H
				Q	A	R	I	Q	I	L	E	L	M	N	N	T	I	S	K	P	A	E	L	K	S	N	V	P	R	I	Q	Q	I
				T	I	P	K	D	K	I	A	A	L	I	G	P	G	G	K	N	I	K	G	I	I	E	Q	T	G	A	T	V	D
				I	T	D	D	G	L	V	S	V	F	A	K	D	A	D	T	L	E	K	T	L	K	L	V	D	S	F	V	R	E
				V	E	Y	N	E	V	Y	E	G	R	V	V	S	I	M	K	F	G	A	F	M	E	I	L	P	G	K	E	G	L
				L	H	I	S	E	I	S	P	E	R	V	E	K	V	E	D	V	L	S	V	G	D	V	F	K	V	R	V	I	S
				M	E	G	G	K	I	S	L	S	K	K	K	V																	
				1				5					10					15					20					25					30
87	3.06%	98.0%	>tr|D5REL8|D5REL8_FUSN2 Putative iron-sulfur cluster-binding protein OS=Fusobacterium nucleatum subsp. nucleatum (strain ATCC 23726 / VPI 4351) OX=525283 GN=HMPREF0397_1653 PE=4 SV=1																														
				M	A	S	E	D	L	K	K	E	I	R	F	A	L	E	N	A	T	L	G	R	T	L	G	N	F	C	K	T	Y
				P	A	R	R	E	K	S	Y	A	G	V	D	F	E	K	T	R	E	K	I	A	E	V	K	S	Y	A	A	E	H
				I	D	E	M	I	A	E	F	T	T	N	C	E	A	R	G	G	H	V	Y	H	A	K	S	T	E	D	A	M	E
				W	I	R	Q	L	V	K	E	K	G	V	K	T	I	V	K	S	K	S	M	A	S	E	E	I	K	M	N	H	V
				L	T	D	D	G	V	L	V	Q	E	T	D	L	G	E	F	I	I	A	L	E	G	N	T	P	V	H	M	V	M
				P	A	L	H	L	N	K	E	Q	V	A	D	L	F	T	D	Y	T	K	V	K	N	N	P	I	I	S	E	E	V
				K	T	A	R	R	V	M	R	D	K	F	T	H	A	D	M	G	V	S	G	A	N	V	A	V	A	E	T	G	T
				V	F	T	M	T	N	E	G	N	G	R	M	V	G	T	L	P	P	I	H	L	Y	V	F	G	I	E	K	F	V
				K	S	L	S	D	A	R	Y	I	F	K	A	L	P	R	N	G	T	A	Q	R	I	T	S	Y	I	S	M	Y	T
				G	A	C	E	V	T	T	N	K	E	T	D	E	K	C	K	K	D	F	Y	C	V	I	L	D	D	P	G	R	R
				E	I	L	A	E	P	D	F	R	E	I	F	N	C	I	R	C	G	A	C	L	D	V	C	P	A	F	A	L	V
				G	G	H	V	Y	G	S	N	V	Y	T	G	G	I	G	T	M	L	T	H	F	L	V	S	E	E	R	A	A	E
				I	Q	N	I	C	L	Q	C	G	R	C	N	E	V	C	G	G	G	L	H	I	S	D	M	I	M	K	L	R	E
				K	N	M	K	D	N	P	D	A	L	K	K	F	A	L	D	A	V	S	D	R	K	L	F	H	S	M	L	R	I
				A	S	V	A	Q	G	M	F	T	K	G	E	P	M	I	R	H	L	P	M	F	L	S	G	M	T	K	G	R	S
				F	P	A	I	A	Q	V	P	L	R	D	F	F	H	T	I	K	Q	D	V	K	N	P	K	G	T	V	A	I	F
				A	G	C	L	L	D	F	V	Y	T	D	L	A	R	A	V	V	A	N	M	N	S	I	G	Y	K	V	E	M	P
				L	G	Q	A	C	C	G	C	P	A	T	N	M	G	D	T	E	N	A	R	K	E	A	E	I	N	I	N	G	M
				E	S	E	K	Y	D	Y	V	V	S	A	C	P	S	C	T	H	Q	L	H	L	Y	P	T	F	F	E	E	G	T
				E	M	H	K	R	A	K	E	L	A	E	K	T	S	D	F	C	K	L	F	Y	E	L	G	G	M	S	E	T	G
				D	G	K	P	M	K	V	T	Y	H	D	S	C	H	L	K	R	S	L	K	V	S	K	E	Q	R	E	L	L	K
				N	T	K	G	V	E	F	V	E	M	N	D	C	D	N	C	C	G	F	G	G	S	Y	S	L	L	Y	P	E	I
				S	A	P	I	L	E	K	K	I	Q	N	I	K	E	S	G	A	D	V	V	A	L	D	C	P	G	C	L	M	Q
				I	K	G	G	L	D	A	R	G	I	D	D	I	K	V	K	H	T	A	E	I	I	A	E	K	R	G	L	I	
				1				5					10					15					20					25					30
88	3.60%	98.3%	>tr|U7TUD4|U7TUD4_FUSNU Uncharacterized protein OS=Fusobacterium nucleatum CTI-6 OX=1316587 GN=HMPREF1767_01582 PE=4 SV=1																														
				M	E	K	I	Y	K	I	V	A	E	E	L	K	I	P	V	D	K	V	E	N	T	I	K	L	L	D	D	G	A
				T	I	P	F	V	A	R	Y	R	K	E	I	T	G	N	L	D	E	V	Q	I	G	D	I	L	Q	K	V	E	Y
				L	R	N	L	E	E	R	K	E	E	V	I	R	L	I	E	E	Q	G	K	L	T	E	E	L	R	N	S	I	I
				E	A	K	I	L	Q	E	V	E	D	I	Y	F	P	Y	R	K	K	K	K	T	K	A	D	I	A	K	E	R	G
				L	E	P	L	A	E	K	F	Y	T	A	N	N	L	E	E	I	Q	N	L	A	K	D	F	I	T	E	E	V	P
				T	V	E	D	A	I	E	G	A	M	L	I	I	A	Q	N	I	S	E	K	A	E	Y	R	E	R	I	R	E	I
				Y	L	K	Y	S	I	I	E	S	K	A	S	K	K	A	A	E	L	D	E	K	K	V	Y	N	D	Y	Y	E	Y
				A	E	K	V	E	K	M	P	S	H	R	I	L	A	L	N	R	G	E	K	E	D	I	L	T	V	H	L	K	L
				E	D	S	D	R	E	R	I	E	N	M	I	L	R	E	F	S	K	N	D	L	V	S	T	Y	K	E	I	I	K
				D	S	L	D	R	L	I	V	P	S	I	E	R	E	V	R	N	A	L	T	E	R	A	E	I	E	S	I	A	V
				F	K	D	N	L	K	N	L	L	L	Q	A	P	L	K	E	K	N	V	L	A	L	D	P	G	Y	R	T	G	C
				K	V	A	V	I	D	K	Y	G	F	Y	K	E	N	T	V	F	F	L	V	E	A	M	H	N	P	K	Q	I	Q

<table>
<tr><td></td><td></td><td></td><td></td><td>D</td><td>A</td><td>R</td><td>D</td><td>K</td><td>F</td><td>L</td><td>K</td><td>L</td><td>V</td><td>K</td><td>K</td><td>Y</td><td>E</td><td>I</td><td>D</td><td>I</td><td>V</td><td>S</td><td>I</td><td>G</td><td>N</td><td>G</td><td>T</td><td>A</td><td>S</td><td>R</td><td>E</td><td>T</td><td>E</td></tr>
<tr><td></td><td></td><td></td><td></td><td>T</td><td>F</td><td>V</td><td>A</td><td>N</td><td>I</td><td>I</td><td>K</td><td>E</td><td>E</td><td>K</td><td>L</td><td>N</td><td>V</td><td>K</td><td>Y</td><td>L</td><td>I</td><td>V</td><td>N</td><td>E</td><td>A</td><td>G</td><td>A</td><td>S</td><td>V</td><td>Y</td><td>S</td><td>A</td><td>S</td></tr>
<tr><td></td><td></td><td></td><td></td><td>K</td><td>I</td><td>A</td><td>A</td><td>E</td><td>E</td><td>F</td><td>P</td><td>D</td><td>L</td><td>D</td><td>V</td><td>T</td><td>V</td><td>R</td><td>G</td><td>A</td><td>I</td><td>S</td><td>I</td><td>G</td><td>R</td><td>R</td><td>I</td><td>Q</td><td>D</td><td>P</td><td>L</td><td>A</td><td>E</td></tr>
<tr><td></td><td></td><td></td><td></td><td>L</td><td>V</td><td>K</td><td>I</td><td>D</td><td>P</td><td>K</td><td>S</td><td>I</td><td>G</td><td>V</td><td>G</td><td>M</td><td>Y</td><td>Q</td><td>H</td><td>D</td><td>V</td><td>N</td><td>Q</td><td>S</td><td>K</td><td>L</td><td>D</td><td>E</td><td>S</td><td>L</td><td>D</td><td>N</td><td>V</td></tr>
<tr><td></td><td></td><td></td><td></td><td>I</td><td>S</td><td>H</td><td>V</td><td>V</td><td>N</td><td>N</td><td>V</td><td>G</td><td>A</td><td>N</td><td>I</td><td>N</td><td>T</td><td>A</td><td>S</td><td>W</td><td>A</td><td>L</td><td>L</td><td>S</td><td>H</td><td>I</td><td>S</td><td>G</td><td>I</td><td>K</td><td>K</td><td>T</td><td>V</td></tr>
<tr><td></td><td></td><td></td><td></td><td>A</td><td>K</td><td>N</td><td>I</td><td>V</td><td>D</td><td>Y</td><td>R</td><td>K</td><td>E</td><td>N</td><td>G</td><td>N</td><td>F</td><td>K</td><td>N</td><td>R</td><td>K</td><td>E</td><td>I</td><td>L</td><td>K</td><td>V</td><td>K</td><td>G</td><td>V</td><td>G</td><td>P</td><td>K</td><td>A</td></tr>
<tr><td></td><td></td><td></td><td></td><td>Y</td><td>E</td><td>Q</td><td>M</td><td>A</td><td>G</td><td>F</td><td>L</td><td>V</td><td>I</td><td>P</td><td>E</td><td>G</td><td>E</td><td>N</td><td>I</td><td>L</td><td>D</td><td>N</td><td>T</td><td>V</td><td>I</td><td>H</td><td>P</td><td>E</td><td>S</td><td>Y</td><td>G</td><td>I</td><td>A</td></tr>
<tr><td></td><td></td><td></td><td></td><td>E</td><td>A</td><td>I</td><td>L</td><td>G</td><td>K</td><td>I</td><td>N</td><td>F</td><td>D</td><td>L</td><td>E</td><td>K</td><td>Y</td><td>N</td><td>N</td><td>E</td><td>L</td><td>D</td><td>V</td><td>A</td><td>R</td><td>E</td><td>R</td><td>L</td><td>K</td><td>S</td><td>F</td><td>D</td><td>Y</td></tr>
<tr><td></td><td></td><td></td><td></td><td>K</td><td>K</td><td>F</td><td>A</td><td>K</td><td>E</td><td>N</td><td>E</td><td>F</td><td>G</td><td>L</td><td>E</td><td>T</td><td>V</td><td>K</td><td>D</td><td>V</td><td>Y</td><td>E</td><td>A</td><td>L</td><td>L</td><td>K</td><td>D</td><td>R</td><td>R</td><td>D</td><td>P</td><td>R</td><td>D</td></tr>
<tr><td></td><td></td><td></td><td></td><td>D</td><td>F</td><td>E</td><td>K</td><td>P</td><td>L</td><td>L</td><td>K</td><td>S</td><td>D</td><td>I</td><td>L</td><td>N</td><td>I</td><td>D</td><td>N</td><td>L</td><td>E</td><td>V</td><td>G</td><td>M</td><td>E</td><td>L</td><td>E</td><td>G</td><td>T</td><td>V</td><td>R</td><td>N</td><td>V</td></tr>
<tr><td></td><td></td><td></td><td></td><td>V</td><td>K</td><td>F</td><td>G</td><td>A</td><td>F</td><td>I</td><td>D</td><td>I</td><td>G</td><td>L</td><td>K</td><td>N</td><td>D</td><td>A</td><td>L</td><td>L</td><td>H</td><td>I</td><td>S</td><td>E</td><td>I</td><td>S</td><td>D</td><td>K</td><td>Y</td><td>I</td><td>D</td><td>D</td><td>P</td></tr>
<tr><td></td><td></td><td></td><td></td><td>S</td><td>K</td><td>V</td><td>L</td><td>S</td><td>V</td><td>G</td><td>Q</td><td>I</td><td>I</td><td>K</td><td>V</td><td>K</td><td>I</td><td>K</td><td>D</td><td>V</td><td>D</td><td>K</td><td>D</td><td>R</td><td>G</td><td>R</td><td>V</td><td>G</td><td>L</td><td>T</td><td>R</td><td>K</td><td>G</td></tr>
<tr><td></td><td></td><td></td><td></td><td>Q</td><td>N</td><td colspan="28"></td></tr>
<tr><td></td><td></td><td></td><td></td><td>1</td><td></td><td></td><td></td><td>5</td><td></td><td></td><td></td><td></td><td>10</td><td></td><td></td><td></td><td></td><td>15</td><td></td><td></td><td></td><td></td><td>20</td><td></td><td></td><td></td><td></td><td>25</td><td></td><td></td><td></td><td></td><td>30</td></tr>
<tr><td colspan="34"></td></tr>
<tr><td>89</td><td>4.26%</td><td>98.7%</td><td colspan="31">>tr|A0A0M1VS21|A0A0M1VS21_FUSNV 1A family penicillin-binding protein OS=Fusobacterium nucleatum subsp. vincentii 4_1_13 OX=469606 GN=FSCG_00125 PE=4 SV=1</td></tr>
<tr><td></td><td></td><td></td><td></td><td>M</td><td>K</td><td>K</td><td>I</td><td>L</td><td>I</td><td>L</td><td>L</td><td>L</td><td>K</td><td>L</td><td>I</td><td>G</td><td>A</td><td>L</td><td>F</td><td>I</td><td>V</td><td>G</td><td>A</td><td>I</td><td>G</td><td>V</td><td>F</td><td>A</td><td>I</td><td>I</td><td>I</td><td>K</td><td>Y</td></tr>
<tr><td></td><td></td><td></td><td></td><td>R</td><td>L</td><td>E</td><td>L</td><td>P</td><td>N</td><td>I</td><td>Q</td><td>S</td><td>M</td><td>V</td><td>E</td><td>D</td><td>Y</td><td>K</td><td>P</td><td>Q</td><td>M</td><td>A</td><td>T</td><td>I</td><td>I</td><td>Y</td><td>D</td><td>K</td><td>N</td><td>N</td><td>N</td><td>V</td><td>V</td></tr>
<tr><td></td><td></td><td></td><td></td><td>D</td><td>T</td><td>L</td><td>S</td><td>V</td><td>E</td><td>S</td><td>R</td><td>E</td><td>I</td><td>A</td><td>K</td><td>L</td><td>E</td><td>D</td><td>I</td><td>S</td><td>P</td><td>Y</td><td>V</td><td>K</td><td>D</td><td>A</td><td>F</td><td>L</td><td>S</td><td>I</td><td>E</td><td>D</td><td>K</td></tr>
<tr><td></td><td></td><td></td><td></td><td>Q</td><td>F</td><td>Y</td><td>S</td><td>H</td><td>H</td><td>G</td><td>L</td><td>N</td><td>F</td><td>R</td><td>G</td><td>I</td><td>T</td><td>R</td><td>A</td><td>V</td><td>V</td><td>T</td><td>T</td><td>F</td><td>L</td><td>K</td><td>G</td><td>R</td><td>P</td><td>T</td><td>Q</td><td>G</td><td>G</td></tr>
<tr><td></td><td></td><td></td><td></td><td>S</td><td>S</td><td>I</td><td>T</td><td>Q</td><td>Q</td><td>L</td><td>A</td><td>K</td><td>N</td><td>A</td><td>F</td><td>L</td><td>T</td><td>P</td><td>E</td><td>K</td><td>T</td><td>F</td><td>S</td><td>R</td><td>K</td><td>V</td><td>K</td><td>E</td><td>A</td><td>I</td><td>L</td><td>T</td><td>Y</td></tr>
<tr><td></td><td></td><td></td><td></td><td>Q</td><td>I</td><td>E</td><td>R</td><td>T</td><td>Y</td><td>T</td><td>K</td><td>D</td><td>E</td><td>I</td><td>L</td><td>E</td><td>R</td><td>Y</td><td>L</td><td>N</td><td>E</td><td>I</td><td>Y</td><td>F</td><td>G</td><td>S</td><td>G</td><td>S</td><td>Y</td><td>G</td><td>I</td><td>R</td><td>N</td></tr>
<tr><td></td><td></td><td></td><td></td><td>A</td><td>A</td><td>E</td><td>Q</td><td>Y</td><td>F</td><td>K</td><td>K</td><td>D</td><td>V</td><td>K</td><td>D</td><td>L</td><td>N</td><td>I</td><td>A</td><td>E</td><td>A</td><td>A</td><td>L</td><td>L</td><td>A</td><td>G</td><td>I</td><td>P</td><td>N</td><td>R</td><td>P</td><td>T</td><td>K</td></tr>
<tr><td></td><td></td><td></td><td></td><td>Y</td><td>D</td><td>P</td><td>N</td><td>R</td><td>N</td><td>L</td><td>E</td><td>N</td><td>A</td><td>L</td><td>H</td><td>R</td><td>Q</td><td>K</td><td>I</td><td>I</td><td>L</td><td>K</td><td>E</td><td>M</td><td>Y</td><td>D</td><td>D</td><td>G</td><td>R</td><td>I</td><td>T</td><td>K</td><td>E</td></tr>
<tr><td></td><td></td><td></td><td></td><td>Q</td><td>Y</td><td>D</td><td>E</td><td>A</td><td>L</td><td>A</td><td>Y</td><td>K</td><td>F</td><td>E</td><td>L</td><td>E</td><td>N</td><td>E</td><td>D</td><td>N</td><td>V</td><td>K</td><td>N</td><td>V</td><td>P</td><td>A</td><td>N</td><td>T</td><td>S</td><td>I</td><td>I</td><td>Y</td><td>N</td></tr>
<tr><td></td><td></td><td></td><td></td><td>R</td><td>R</td><td>T</td><td>K</td><td>A</td><td>T</td><td>Y</td><td>K</td><td>N</td><td>P</td><td>E</td><td>L</td><td>T</td><td>T</td><td>I</td><td>V</td><td>E</td><td>D</td><td>Y</td><td>L</td><td>A</td><td>E</td><td>I</td><td>Y</td><td>D</td><td>E</td><td>E</td><td>Q</td><td>I</td><td>Y</td></tr>
<tr><td></td><td></td><td></td><td></td><td>T</td><td>S</td><td>G</td><td>L</td><td>K</td><td>I</td><td>Y</td><td>T</td><td>T</td><td>I</td><td>D</td><td>L</td><td>D</td><td>Y</td><td>Q</td><td>K</td><td>V</td><td>A</td><td>K</td><td>E</td><td>T</td><td>F</td><td>N</td><td>S</td><td>Y</td><td>P</td><td>Y</td><td>F</td><td>K</td><td>N</td></tr>
<tr><td></td><td></td><td></td><td></td><td>K</td><td>E</td><td>I</td><td>N</td><td>G</td><td>A</td><td>M</td><td>I</td><td>T</td><td>L</td><td>D</td><td>P</td><td>F</td><td>T</td><td>G</td><td>G</td><td>I</td><td>V</td><td>S</td><td>I</td><td>V</td><td>G</td><td>G</td><td>K</td><td>N</td><td>F</td><td>K</td><td>A</td><td>G</td><td>N</td></tr>
<tr><td></td><td></td><td></td><td></td><td>F</td><td>D</td><td>R</td><td>A</td><td>T</td><td>M</td><td>A</td><td>R</td><td>R</td><td>Q</td><td>L</td><td>G</td><td>S</td><td>S</td><td>F</td><td>K</td><td>P</td><td>F</td><td>V</td><td>Y</td><td>L</td><td>E</td><td>A</td><td>L</td><td>E</td><td>N</td><td>G</td><td>F</td><td>T</td><td>P</td></tr>
<tr><td></td><td></td><td></td><td></td><td>Y</td><td>S</td><td>V</td><td>V</td><td>V</td><td>N</td><td>D</td><td>F</td><td>V</td><td>A</td><td>F</td><td>G</td><td>K</td><td>W</td><td>A</td><td>P</td><td>K</td><td>N</td><td>F</td><td>D</td><td>G</td><td>R</td><td>Y</td><td>S</td><td>Y</td><td>N</td><td>S</td><td>T</td><td>L</td><td>V</td></tr>
<tr><td></td><td></td><td></td><td></td><td>N</td><td>S</td><td>L</td><td>N</td><td>L</td><td>S</td><td>L</td><td>N</td><td>V</td><td>P</td><td>A</td><td>V</td><td>K</td><td>L</td><td>L</td><td>D</td><td>A</td><td>I</td><td>T</td><td>V</td><td>D</td><td>T</td><td>F</td><td>K</td><td>E</td><td>A</td><td>I</td><td>G</td><td>D</td><td>N</td></tr>
<tr><td></td><td></td><td></td><td></td><td>V</td><td>K</td><td>L</td><td>S</td><td>S</td><td>E</td><td>V</td><td>K</td><td>D</td><td>L</td><td>T</td><td>A</td><td>A</td><td>L</td><td>G</td><td>S</td><td>V</td><td>D</td><td>S</td><td>T</td><td>P</td><td>V</td><td>N</td><td>V</td><td>A</td><td>A</td><td>N</td><td>F</td><td>S</td><td>I</td></tr>
<tr><td></td><td></td><td></td><td></td><td>F</td><td>V</td><td>N</td><td>G</td><td>G</td><td>Y</td><td>I</td><td>V</td><td>K</td><td>P</td><td>N</td><td>I</td><td>I</td><td>R</td><td>E</td><td>I</td><td>R</td><td>D</td><td>N</td><td>Q</td><td>D</td><td>I</td><td>L</td><td>I</td><td>Y</td><td>V</td><td>A</td><td>E</td><td>I</td><td>E</td></tr>
<tr><td></td><td></td><td></td><td></td><td>K</td><td>T</td><td>K</td><td>V</td><td>F</td><td>D</td><td>S</td><td>V</td><td>D</td><td>V</td><td>S</td><td>V</td><td>I</td><td>T</td><td>A</td><td>M</td><td>L</td><td>K</td><td>T</td><td>V</td><td>V</td><td>S</td><td>N</td><td>G</td><td>T</td><td>A</td><td>S</td><td>K</td><td>A</td><td>R</td></tr>
<tr><td></td><td></td><td></td><td></td><td>V</td><td>V</td><td>D</td><td>K</td><td>T</td><td>G</td><td>K</td><td>P</td><td>I</td><td>Q</td><td>Q</td><td>G</td><td>G</td><td>K</td><td>T</td><td>G</td><td>T</td><td>T</td><td>N</td><td>E</td><td>H</td><td>R</td><td>T</td><td>A</td><td>W</td><td>F</td><td>V</td><td>G</td><td>I</td><td>T</td></tr>
<tr><td></td><td></td><td></td><td></td><td>P</td><td>E</td><td>Y</td><td>V</td><td>T</td><td>A</td><td>C</td><td>Y</td><td>I</td><td>G</td><td>R</td><td>D</td><td>D</td><td>N</td><td>K</td><td>P</td><td>M</td><td>Y</td><td>G</td><td>K</td><td>M</td><td>T</td><td>G</td><td>G</td><td>S</td><td>A</td><td>V</td><td>A</td><td>P</td><td>M</td></tr>
<tr><td></td><td></td><td></td><td></td><td>W</td><td>S</td><td>K</td><td>Y</td><td>Y</td><td>Q</td><td>T</td><td>L</td><td>I</td><td>N</td><td>K</td><td>G</td><td>L</td><td>Y</td><td>T</td><td>P</td><td>G</td><td>K</td><td>F</td><td>E</td><td>F</td><td>L</td><td>E</td><td>N</td><td>Y</td><td>L</td><td>E</td><td>T</td><td>G</td><td>D</td></tr>
<tr><td></td><td></td><td></td><td></td><td>L</td><td>V</td><td>K</td><td>Q</td><td>N</td><td>I</td><td>D</td><td>I</td><td>Y</td><td>S</td><td>G</td><td>L</td><td>L</td><td>D</td><td>G</td><td>P</td><td>N</td><td>S</td><td>K</td><td>E</td><td>F</td><td>T</td><td>I</td><td>R</td><td>K</td><td>G</td><td>R</td><td>L</td><td>Q</td><td>V</td></tr>
<tr><td></td><td></td><td></td><td></td><td>E</td><td>S</td><td>A</td><td>G</td><td>K</td><td>Y</td><td>K</td><td>N</td><td>G</td><td>I</td><td>A</td><td>S</td><td>V</td><td>F</td><td>G</td><td>L</td><td>D</td><td>G</td><td>N</td><td>V</td><td>T</td><td>D</td><td>G</td><td>A</td><td>G</td><td>I</td><td>D</td><td>M</td><td>S</td><td>E</td></tr>
<tr><td></td><td></td><td></td><td></td><td>G</td><td>M</td><td>I</td><td>I</td><td>D</td><td>T</td><td>G</td><td>I</td><td>E</td><td>K</td><td>G</td><td>T</td><td>V</td><td>T</td><td>E</td><td>G</td><td>G</td><td>T</td><td>G</td><td>E</td><td>R</td><td>T</td><td>T</td><td>E</td><td>T</td><td>G</td><td>T</td><td>H</td><td>N</td><td>T</td></tr>
<tr><td></td><td></td><td></td><td></td><td>P</td><td>S</td><td>T</td><td>S</td><td>T</td><td>E</td><td>G</td><td>T</td><td>T</td><td>P</td><td>P</td><td>V</td><td>Q</td><td>N</td><td>N</td><td>N</td><td>S</td><td>T</td><td>N</td><td>K</td><td>D</td><td>G</td><td>D</td><td>S</td><td>L</td><td>T</td><td>N</td><td>R</td><td>L</td><td>L</td></tr>
<tr><td></td><td></td><td></td><td></td><td>G</td><td>D</td><td colspan="28"></td></tr>
<tr><td></td><td></td><td></td><td></td><td>1</td><td></td><td></td><td></td><td>5</td><td></td><td></td><td></td><td></td><td>10</td><td></td><td></td><td></td><td></td><td>15</td><td></td><td></td><td></td><td></td><td>20</td><td></td><td></td><td></td><td></td><td>25</td><td></td><td></td><td></td><td></td><td>30</td></tr>
<tr><td colspan="34"></td></tr>
<tr><td>90</td><td>5.01%</td><td>97.9%</td><td colspan="31">>tr|D5RDR4|D5RDR4_FUSN2 Pyridine nucleotide-disulfide oxidoreductase OS=Fusobacterium nucleatum subsp. nucleatum (strain ATCC 23726 / VPI 4351) OX=525283 GN=HMPREF0397_1349 PE=3 SV=1</td></tr>
<tr><td></td><td></td><td></td><td></td><td>M</td><td>E</td><td>K</td><td>I</td><td>Y</td><td>D</td><td>L</td><td>L</td><td>V</td><td>I</td><td>G</td><td>W</td><td>G</td><td>K</td><td>A</td><td>G</td><td>K</td><td>T</td><td>L</td><td>S</td><td>A</td><td>K</td><td>L</td><td>G</td><td>A</td><td>K</td><td>G</td><td>K</td><td>K</td><td>V</td></tr>
<tr><td></td><td></td><td></td><td></td><td>A</td><td>I</td><td>I</td><td>E</td><td>E</td><td>N</td><td>P</td><td>K</td><td>M</td><td>Y</td><td>G</td><td>G</td><td>T</td><td>C</td><td>I</td><td>N</td><td>V</td><td>G</td><td>C</td><td>L</td><td>P</td><td>T</td><td>K</td><td>S</td><td>L</td><td>V</td><td>H</td><td>S</td><td>A</td><td>K</td></tr>
<tr><td></td><td></td><td></td><td></td><td>I</td><td>L</td><td>A</td><td>E</td><td>V</td><td>K</td><td>K</td><td>Y</td><td>G</td><td>I</td><td>D</td><td>G</td><td>D</td><td>Y</td><td>S</td><td>F</td><td>K</td><td>N</td><td>N</td><td>F</td><td>F</td><td>K</td><td>E</td><td>A</td><td>M</td><td>K</td><td>K</td><td>K</td><td>E</td><td>E</td></tr>
<tr><td></td><td></td><td></td><td></td><td>M</td><td>T</td><td>I</td><td>K</td><td>L</td><td>R</td><td>N</td><td>K</td><td>N</td><td>F</td><td>G</td><td>L</td><td>L</td><td>D</td><td>T</td><td>N</td><td>E</td><td>N</td><td>V</td><td>D</td><td>I</td><td>Y</td><td>N</td><td>G</td><td>R</td><td>A</td><td>S</td><td>F</td><td>V</td><td>S</td></tr>
<tr><td></td><td></td><td></td><td></td><td>N</td><td>N</td><td>E</td><td>V</td><td>K</td><td>I</td><td>T</td><td>S</td><td>S</td><td>D</td><td>N</td><td>K</td><td>E</td><td>I</td><td>V</td><td>L</td><td>K</td><td>A</td><td>D</td><td>K</td><td>I</td><td>V</td><td>I</td><td>N</td><td>T</td><td>G</td><td>S</td><td>V</td><td>S</td><td>R</td></tr>
<tr><td></td><td></td><td></td><td></td><td>T</td><td>L</td><td>N</td><td>T</td><td>E</td><td>G</td><td>I</td><td>D</td><td>N</td><td>K</td><td>N</td><td>V</td><td>M</td><td>T</td><td>S</td><td>E</td><td>G</td><td>I</td><td>L</td><td>E</td><td>L</td><td>K</td><td>E</td><td>L</td><td>P</td><td>K</td><td>K</td><td>L</td><td>L</td><td>I</td></tr>
<tr><td></td><td></td><td></td><td></td><td>I</td><td>G</td><td>A</td><td>G</td><td>Y</td><td>I</td><td>G</td><td>L</td><td>E</td><td>F</td><td>A</td><td>S</td><td>Y</td><td>F</td><td>S</td><td>N</td><td>F</td><td>G</td><td>S</td><td>E</td><td>V</td><td>S</td><td>V</td><td>F</td><td>Q</td><td>F</td><td>D</td><td>D</td><td>A</td><td>F</td></tr>
<tr><td></td><td></td><td></td><td></td><td>L</td><td>A</td><td>R</td><td>E</td><td>D</td><td>E</td><td>D</td><td>E</td><td>T</td><td>K</td><td>I</td><td>I</td><td>K</td><td>E</td><td>I</td><td>L</td><td>E</td><td>N</td><td>K</td><td>G</td><td>V</td><td>K</td><td>F</td><td>F</td><td>F</td><td>N</td><td>T</td><td>S</td><td>V</td><td>K</td></tr>
<tr><td></td><td></td><td></td><td></td><td>K</td><td>F</td><td>E</td><td>D</td><td>L</td><td>G</td><td>D</td><td>S</td><td>V</td><td>K</td><td>A</td><td>I</td><td>C</td><td>M</td><td>K</td><td>D</td><td>G</td><td>Q</td><td>E</td><td>F</td><td>S</td><td>E</td><td>E</td><td>F</td><td>N</td><td>K</td><td>V</td><td>L</td><td>V</td><td>A</td></tr>
<tr><td></td><td></td><td></td><td></td><td>V</td><td>G</td><td>R</td><td>K</td><td>P</td><td>N</td><td>T</td><td>D</td><td>N</td><td>L</td><td>G</td><td>L</td><td>E</td><td>N</td><td>T</td><td>S</td><td>I</td><td>Q</td><td>L</td><td>G</td><td>K</td><td>F</td><td>G</td><td>E</td><td>I</td><td>L</td><td>V</td><td>D</td><td>D</td><td>Y</td></tr>
<tr><td></td><td></td><td></td><td></td><td>L</td><td>K</td><td>T</td><td>N</td><td>A</td><td>P</td><td>N</td><td>I</td><td>W</td><td>A</td><td>V</td><td>G</td><td>D</td><td>V</td><td>K</td><td>G</td><td>G</td><td>A</td><td>Q</td><td>F</td><td>T</td><td>Y</td><td>V</td><td>S</td><td>L</td><td>D</td><td>D</td><td>F</td><td>R</td><td>I</td></tr>
<tr><td></td><td></td><td></td><td></td><td>V</td><td>F</td><td>P</td><td>Q</td><td>I</td><td>L</td><td>G</td><td>E</td><td>N</td><td>N</td><td>R</td><td>R</td><td>K</td><td>L</td><td>S</td><td>D</td><td>R</td><td>V</td><td>L</td><td>I</td><td>P</td><td>T</td><td>S</td><td>T</td><td>F</td><td>I</td><td>D</td><td>P</td><td>P</td><td>Y</td></tr>
<tr><td></td><td></td><td></td><td></td><td>S</td><td>R</td><td>V</td><td>G</td><td>I</td><td>N</td><td>E</td><td>K</td><td>E</td><td>A</td><td>Q</td><td>R</td><td>L</td><td>G</td><td>I</td><td>N</td><td>Y</td><td>T</td><td>K</td><td>K</td><td>F</td><td>A</td><td>L</td><td>T</td><td>N</td><td>T</td><td>I</td><td>P</td><td>K</td><td>A</td></tr>
<tr><td></td><td></td><td></td><td></td><td>H</td><td>V</td><td>I</td><td>N</td><td>E</td><td>I</td><td>D</td><td>G</td><td>F</td><td>T</td><td>K</td><td>I</td><td>L</td><td>I</td><td>N</td><td>E</td><td>N</td><td>D</td><td>E</td><td>I</td><td>I</td><td>G</td><td>A</td><td>S</td><td>I</td><td>C</td><td>H</td><td>Y</td><td>E</td><td>S</td></tr>
<tr><td></td><td></td><td></td><td></td><td>H</td><td>E</td><td>M</td><td>I</td><td>N</td><td>L</td><td>L</td><td>A</td><td>L</td><td>A</td><td>I</td><td>N</td><td>Q</td><td>K</td><td>I</td><td>K</td><td>S</td><td>K</td><td>V</td><td>L</td><td>K</td><td>D</td><td>F</td><td>I</td><td>Y</td><td>T</td><td>H</td><td>P</td><td>I</td><td>F</td></tr>
<tr><td></td><td></td><td></td><td></td><td>I</td><td>E</td><td>S</td><td>L</td><td>N</td><td>D</td><td>I</td><td>L</td><td>G</td><td colspan="21"></td></tr>
<tr><td></td><td></td><td></td><td></td><td>1</td><td></td><td></td><td></td><td>5</td><td></td><td></td><td></td><td></td><td>10</td><td></td><td></td><td></td><td></td><td>15</td><td></td><td></td><td></td><td></td><td>20</td><td></td><td></td><td></td><td></td><td>25</td><td></td><td></td><td></td><td></td><td>30</td></tr>
<tr><td colspan="34"></td></tr>
<tr><td>91</td><td>2.03%</td><td>99.0%</td><td colspan="31">>tr|A0A0M1VWD5|A0A0M1VWD5_FUSNV Uncharacterized protein OS=Fusobacterium nucleatum subsp. vincentii 4_1_13 OX=469606 GN=FSCG_01708 PE=4 SV=1</td></tr>
<tr><td></td><td></td><td></td><td></td><td>M</td><td>R</td><td>K</td><td>Y</td><td>L</td><td>I</td><td>I</td><td>S</td><td>L</td><td>L</td><td>A</td><td>S</td><td>Y</td><td>S</td><td>I</td><td>V</td><td>F</td><td>A</td><td>G</td><td>E</td><td>S</td><td>E</td><td>D</td><td>F</td><td>K</td><td>A</td><td>V</td><td>D</td><td>N</td><td>L</td></tr>
<tr><td></td><td></td><td></td><td></td><td>Y</td><td>K</td><td>E</td><td>R</td><td>N</td><td>F</td><td>K</td><td>A</td><td>A</td><td>L</td><td>V</td><td>E</td><td>S</td><td>E</td><td>K</td><td>F</td><td>L</td><td>Q</td><td>K</td><td>Y</td><td>P</td><td>E</td><td>S</td><td>K</td><td>H</td><td>Q</td><td>K</td><td>S</td><td>M</td><td>R</td></tr>
<tr><td></td><td></td><td></td><td></td><td>D</td><td>K</td><td>V</td><td>A</td><td>K</td><td>I</td><td>Y</td><td>F</td><td>L</td><td>E</td><td>K</td><td>N</td><td>Y</td><td>K</td><td>K</td><td>A</td><td>E</td><td>E</td><td>I</td><td>F</td><td>K</td><td>K</td><td>L</td><td>Y</td><td>T</td><td>I</td><td>E</td><td>E</td><td>K</td><td>Q</td></tr>
<tr><td></td><td></td><td></td><td></td><td>S</td><td>Q</td><td>K</td><td>D</td><td>E</td><td>Y</td><td>A</td><td>S</td><td>Y</td><td>L</td><td>A</td><td>R</td><td>A</td><td>N</td><td>A</td><td>L</td><td>L</td><td>N</td><td>N</td><td>S</td><td>D</td><td>S</td><td>A</td><td>I</td><td>F</td><td>Y</td><td>A</td><td>R</td><td>E</td><td>I</td></tr>
<tr><td></td><td></td><td></td><td></td><td>K</td><td>D</td><td>K</td><td>K</td><td>T</td><td>F</td><td>Q</td><td>K</td><td>T</td><td>F</td><td>F</td><td>A</td><td>V</td><td>A</td><td>Q</td><td>N</td><td>F</td><td>L</td><td>A</td><td>K</td><td>E</td><td>N</td><td>N</td><td>E</td><td>A</td><td>A</td><td>Q</td><td>K</td><td>A</td><td>Y</td></tr>
<tr><td></td><td></td><td></td><td></td><td>K</td><td>E</td><td>I</td><td>I</td><td>D</td><td>N</td><td>K</td><td>Y</td><td>E</td><td>N</td><td>Y</td><td>K</td><td>E</td><td>S</td><td>M</td><td>M</td><td>G</td><td>L</td><td>G</td><td>I</td><td>V</td><td>Y</td><td>Y</td><td>N</td><td>L</td><td>K</td><td>D</td><td>Y</td><td>D</td><td>K</td></tr>
<tr><td></td><td></td><td></td><td></td><td>A</td><td>I</td><td>Y</td><td>W</td><td>L</td><td>S</td><td>E</td><td>F</td><td>S</td><td>K</td><td>E</td><td>M</td><td>P</td><td>K</td><td>E</td><td>N</td><td>K</td><td>E</td><td>M</td><td>V</td><td>S</td><td>Y</td><td>L</td><td>R</td><td>A</td><td>S</td><td>A</td><td>L</td><td>Y</td><td>R</td></tr>
<tr><td></td><td></td><td></td><td></td><td>K</td><td>G</td><td>N</td><td>T</td><td>D</td><td>E</td><td>A</td><td>I</td><td>S</td><td>R</td><td>F</td><td>E</td><td>E</td><td>L</td><td>A</td><td>N</td><td>I</td><td>E</td><td>P</td><td>S</td><td>T</td><td>E</td><td>Y</td><td>S</td><td>R</td><td>K</td><td>A</td><td>A</td><td>L</td><td>Y</td></tr>
<tr><td></td><td></td><td></td><td></td><td>L</td><td>I</td><td>E</td><td>I</td><td>Y</td><td>S</td><td>N</td><td>R</td><td>K</td><td>D</td><td>E</td><td>A</td><td>K</td><td>V</td><td>T</td><td>F</td><td>Y</td><td>L</td><td>N</td><td>R</td><td>I</td><td>K</td><td>G</td><td>T</td><td>K</td><td>E</td><td>Y</td><td>N</td><td>T</td><td>A</td></tr>
<tr><td></td><td></td><td></td><td></td><td>M</td><td>T</td><td>M</td><td>I</td><td>G</td><td>D</td><td>L</td><td>Y</td><td>V</td><td>T</td><td>K</td><td>E</td><td>N</td><td>Y</td><td>D</td><td>K</td><td>A</td><td>L</td><td>D</td><td>Y</td><td>Y</td><td>N</td><td>Q</td><td>S</td><td>N</td><td>D</td><td>K</td><td>N</td><td>N</td><td>P</td></tr>
<tr><td></td><td></td><td></td><td></td><td>K</td><td>L</td><td>I</td><td>Y</td><td>G</td><td>E</td><td>A</td><td>Y</td><td>S</td><td>L</td><td>Y</td><td>K</td><td>N</td><td>G</td><td>K</td><td>Y</td><td>E</td><td>E</td><td>A</td><td>L</td><td>K</td><td>K</td><td>F</td><td>Q</td><td>S</td><td>L</td><td>K</td><td>N</td><td>S</td><td>D</td></tr>
<tr><td></td><td></td><td></td><td></td><td>Y</td><td>Y</td><td>N</td><td>Q</td><td>S</td><td>I</td><td>Y</td><td>H</td><td>I</td><td>F</td><td>A</td><td>I</td><td>N</td><td>Y</td><td>K</td><td>L</td><td>K</td><td>N</td><td>F</td><td>D</td><td>E</td><td>I</td><td>I</td><td>R</td><td>D</td><td>R</td><td>E</td><td>I</td><td>I</td><td>R</td></tr>
<tr><td></td><td></td><td></td><td></td><td>K</td><td>V</td><td>V</td><td>V</td><td>S</td><td>Q</td><td>V</td><td>D</td><td>T</td><td>D</td><td>N</td><td>I</td><td>I</td><td>R</td><td>I</td><td>I</td><td>A</td><td>N</td><td>S</td><td>A</td><td>Y</td><td>Q</td><td>V</td><td>G</td><td>N</td><td>Y</td><td>K</td><td>L</td><td>A</td><td>K</td></tr>
<tr><td></td><td></td><td></td><td></td><td>D</td><td>Y</td><td>Y</td><td>G</td><td>R</td><td>L</td><td>F</td><td>A</td><td>V</td><td>S</td><td>P</td><td>D</td><td>K</td><td>E</td><td>N</td><td>L</td><td>F</td><td>R</td><td>V</td><td>I</td><td>L</td><td>L</td><td>D</td><td>S</td><td>Q</td><td>M</td><td>L</td><td>D</td><td>M</td><td>E</td></tr>
<tr><td></td><td></td><td></td><td></td><td>D</td><td>L</td><td>R</td><td>I</td><td>R</td><td>F</td><td>N</td><td>Q</td><td>Y</td><td>N</td><td>E</td><td>L</td><td>Y</td><td>S</td><td>N</td><td>D</td><td>T</td><td>E</td><td>F</td><td>K</td><td>K</td><td>D</td><td>V</td><td>Y</td><td>L</td><td>Y</td><td>T</td><td>G</td><td>D</td><td>A</td></tr>
<tr><td></td><td></td><td></td><td></td><td>Y</td><td>Y</td><td>K</td><td>A</td><td>G</td><td>D</td><td>P</td><td>E</td><td>R</td><td>A</td><td>E</td><td>Q</td><td>I</td><td>Y</td><td>K</td><td>T</td><td>Y</td><td>L</td><td>N</td><td>Q</td><td>Y</td><td>T</td><td>N</td><td>T</td><td>E</td><td>V</td><td>I</td><td>S</td><td>S</td><td>L</td></tr>
<tr><td></td><td></td><td></td><td></td><td>M</td><td>S</td><td>T</td><td>L</td><td>L</td><td>E</td><td>Q</td><td>K</td><td>K</td><td>Y</td><td>D</td><td>E</td><td>M</td><td>Q</td><td>Q</td><td>Y</td><td>L</td><td>S</td><td>L</td><td>A</td><td>Q</td><td>D</td><td>E</td><td>S</td><td>S</td><td>V</td><td>N</td><td>Y</td><td>L</td><td>K</td></tr>
<tr><td></td><td></td><td></td><td></td><td>G</td><td>I</td><td>A</td><td>A</td><td>M</td><td>G</td><td>L</td><td>G</td><td>K</td><td>Y</td><td>D</td><td>E</td><td>A</td><td>E</td><td>S</td><td>E</td><td>F</td><td>Q</td><td>K</td><td>V</td><td>L</td><td>A</td><td>S</td><td>G</td><td>D</td><td>Q</td><td>S</td><td>L</td><td>S</td><td>T</td></tr>
<tr><td></td><td></td><td></td><td></td><td>K</td><td>V</td><td>Y</td><td>L</td><td>N</td><td>R</td><td>V</td><td>R</td><td>N</td><td>Y</td><td>F</td><td>L</td><td>A</td><td>E</td><td>R</td><td>Y</td><td>N</td><td>E</td><td>A</td><td>V</td><td>Q</td><td>A</td><td>G</td><td>E</td><td>Q</td><td>Y</td><td>L</td><td>S</td><td>K</td><td>L</td></tr>
<tr><td></td><td></td><td></td><td></td><td>S</td><td>P</td><td>D</td><td>K</td><td>D</td><td>K</td><td>V</td><td>I</td><td>Y</td><td>S</td><td>E</td><td>M</td><td>L</td><td>D</td><td>K</td><td>I</td><td>G</td><td>L</td><td>S</td><td>Y</td><td>F</td><td>R</td><td>L</td><td>G</td><td>K</td><td>Y</td><td>N</td><td>Q</td><td>A</td><td>R</td></tr>
<tr><td></td><td></td><td></td><td></td><td>A</td><td>Y</td><td>Y</td><td>S</td><td>K</td><td>I</td><td>A</td><td>S</td><td>I</td><td>K</td><td>G</td><td>Y</td><td>E</td><td>V</td><td>Y</td><td>G</td><td>K</td><td>F</td><td>Q</td><td>I</td><td>A</td><td>D</td><td>S</td><td>Y</td><td>Y</td><td>N</td><td>E</td><td>K</td><td>N</td><td>Y</td></tr>
<tr><td></td><td></td><td></td><td></td><td>E</td><td>K</td><td>A</td><td>A</td><td>S</td><td>L</td><td>Y</td><td>K</td><td>E</td><td>V</td><td>Y</td><td>N</td><td>Q</td><td>F</td><td>G</td><td>E</td><td>T</td><td>F</td><td>Y</td><td>G</td><td>E</td><td>Q</td><td>A</td><td>Y</td><td>Y</td><td>K</td><td>Y</td><td>I</td><td>M</td><td>T</td></tr>
<tr><td></td><td></td><td></td><td></td><td>L</td><td>S</td><td>L</td><td>T</td><td>G</td><td>N</td><td>T</td><td>D</td><td>A</td><td>F</td><td>E</td><td>R</td><td>E</td><td>K</td><td>D</td><td>N</td><td>F</td><td>M</td><td>K</td><td>V</td><td>Y</td><td>P</td><td>N</td><td>S</td><td>N</td><td>L</td><td>R</td><td>G</td><td>T</td><td>I</td></tr>
<tr><td></td><td></td><td></td><td></td><td>T</td><td>N</td><td>L</td><td>T</td><td>T</td><td>N</td><td>M</td><td>Y</td><td>I</td><td>E</td><td>S</td><td>G</td><td>D</td><td>T</td><td>D</td><td>K</td><td>A</td><td>I</td><td>E</td><td>S</td><td>L</td><td>N</td><td>N</td><td>S</td><td>S</td><td>A</td><td>N</td><td>T</td><td>S</td><td>D</td></tr>
<tr><td></td><td></td><td></td><td></td><td>I</td><td>S</td><td>V</td><td>K</td><td>E</td><td>N</td><td>N</td><td>T</td><td>A</td><td>K</td><td>I</td><td>I</td><td>T</td><td>L</td><td>K</td><td>L</td><td>Q</td><td>K</td><td>K</td><td>D</td><td>Y</td><td>K</td><td>D</td><td>I</td><td>E</td><td>K</td><td>Y</td><td>I</td><td>A</td><td>E</td></tr>
<tr><td></td><td></td><td></td><td></td><td>L</td><td>P</td><td>T</td><td>E</td><td>E</td><td>E</td><td>R</td><td>V</td><td>Y</td><td>Y</td><td>S</td><td>A</td><td>Q</td><td>Y</td><td>Y</td><td>A</td><td>V</td><td>K</td><td>K</td><td>D</td><td>P</td><td>K</td><td>V</td><td>V</td><td>K</td><td>E</td><td>Y</td><td>E</td><td>I</td><td>L</td></tr>
<tr><td></td><td></td><td></td><td></td><td>L</td><td>Q</td><td>Y</td><td>D</td><td>K</td><td>Y</td><td>K</td><td>A</td><td>Y</td><td>A</td><td>S</td><td>K</td><td>G</td><td>L</td><td>G</td><td>D</td><td>Y</td><td>Y</td><td>F</td><td>D</td><td>K</td><td>K</td><td>D</td><td>L</td><td>S</td><td>K</td><td>A</td><td>K</td><td>K</td><td>Y</td></tr>
<tr><td></td><td></td><td></td><td></td><td>Y</td><td>G</td><td>T</td><td>Y</td><td>A</td><td>T</td><td>V</td><td>N</td><td>K</td><td>N</td><td>P</td><td>D</td><td>E</td><td>Y</td><td>I</td><td>L</td><td>Y</td><td>R</td><td>L</td><td>G</td><td>Q</td><td>A</td><td>N</td><td>E</td><td>K</td><td>E</td><td>N</td><td>N</td><td>L</td><td>K</td></tr>
<tr><td></td><td></td><td></td><td></td><td>M</td><td>A</td><td>L</td><td>T</td><td>D</td><td>Y</td><td>K</td><td>A</td><td>V</td><td>Y</td><td>S</td><td>K</td><td>N</td><td>G</td><td>K</td><td>L</td><td>A</td><td>N</td><td>D</td><td>A</td><td>M</td><td>L</td><td>R</td><td>A</td><td>A</td><td>E</td><td>I</td><td>Y</td><td>D</td><td>K</td></tr>
<tr><td></td><td></td><td></td><td></td><td>Q</td><td>E</td><td>N</td><td>V</td><td>A</td><td>E</td><td>A</td><td>E</td><td>K</td><td>L</td><td>F</td><td>T</td><td>K</td><td>L</td><td>Y</td><td>A</td><td>V</td><td>K</td><td>N</td><td>N</td><td>K</td><td>D</td><td>L</td><td>K</td><td>A</td><td>Y</td><td>S</td><td>I</td><td>E</td><td>K</td></tr>
<tr><td></td><td></td><td></td><td></td><td>L</td><td>I</td><td>Y</td><td>Y</td><td>K</td><td>L</td><td>V</td><td>K</td><td>N</td><td>N</td><td>T</td><td>K</td><td>E</td><td>A</td><td>K</td><td>K</td><td>L</td><td>Y</td><td>D</td><td>E</td><td>L</td><td>K</td><td>K</td><td>L</td><td>D</td><td>A</td><td>K</td><td>R</td><td>A</td><td>E</td></tr>
<tr><td></td><td></td><td></td><td></td><td>K</td><td>F</td><td>K</td><td>A</td><td>Y</td><td>F</td><td colspan="24"></td></tr>
<tr><td></td><td></td><td></td><td></td><td>1</td><td></td><td></td><td></td><td>5</td><td></td><td></td><td></td><td></td><td>10</td><td></td><td></td><td></td><td></td><td>15</td><td></td><td></td><td></td><td></td><td>20</td><td></td><td></td><td></td><td></td><td>25</td><td></td><td></td><td></td><td></td><td>30</td></tr>
<tr><td colspan="34"></td></tr>
<tr><td>92</td><td>26.00%</td><td>89.4%</td><td colspan="31">>tr|Q7M144|Q7M144_FUSNU Major outer membrane protein (Fragment) OS=Fusobacterium nucleatum OX=851 PE=1 SV=1</td></tr>
<tr><td></td><td></td><td></td><td></td><td>K</td><td>E</td><td>V</td><td>M</td><td>P</td><td>A</td><td>P</td><td>T</td><td>P</td><td>A</td><td>P</td><td>E</td><td>K</td><td>V</td><td>V</td><td>E</td><td>Y</td><td>V</td><td>E</td><td>K</td><td>P</td><td>V</td><td>I</td><td>V</td><td>Y</td><td>A</td><td>X</td><td>E</td><td>V</td><td>A</td></tr>
<tr><td></td><td></td><td></td><td></td><td>P</td><td>A</td><td>W</td><td>R</td><td>P</td><td>N</td><td>G</td><td>S</td><td>V</td><td>D</td><td>V</td><td>Q</td><td>Y</td><td>K</td><td>W</td><td>Y</td><td>G</td><td>E</td><td>V</td><td>E</td><td colspan="10"></td></tr>
<tr><td></td><td></td><td></td><td></td><td>1</td><td></td><td></td><td></td><td>5</td><td></td><td></td><td></td><td></td><td>10</td><td></td><td></td><td></td><td></td><td>15</td><td></td><td></td><td></td><td></td><td>20</td><td></td><td></td><td></td><td></td><td>25</td><td></td><td></td><td></td><td></td><td>30</td></tr>
<tr><td colspan="34"></td></tr>
<tr><td>93</td><td>7.18%</td><td>98.7%</td><td colspan="31">>tr|Q8RDT9|Q8RDT9_FUSNN Threonine dehydratase OS=Fusobacterium nucleatum subsp. nucleatum (strain ATCC 25586 / CIP 101130 / JCM 8532 / LMG 13131) OX=190304 GN=FN1411 PE=4 SV=1</td></tr>
<tr><td></td><td></td><td></td><td></td><td>M</td><td>A</td><td>K</td><td>L</td><td>E</td><td>A</td><td>F</td><td>I</td><td>K</td><td>A</td><td>K</td><td>E</td><td>K</td><td>L</td><td>S</td><td>K</td><td>V</td><td>L</td><td>L</td><td>E</td><td>T</td><td>Q</td><td>L</td><td>I</td><td>Y</td><td>S</td><td>P</td><td>I</td><td>F</td><td>S</td></tr>
<tr><td></td><td></td><td></td><td></td><td>K</td><td>E</td><td>S</td><td>G</td><td>N</td><td>K</td><td>V</td><td>F</td><td>I</td><td>K</td><td>P</td><td>E</td><td>N</td><td>L</td><td>Q</td><td>K</td><td>T</td><td>G</td><td>S</td><td>F</td><td>K</td><td>I</td><td>R</td><td>G</td><td>A</td><td>Y</td><td>N</td><td>K</td><td>I</td><td>S</td></tr>
<tr><td></td><td></td><td></td><td></td><td>N</td><td>L</td><td>T</td><td>D</td><td>A</td><td>E</td><td>K</td><td>K</td><td>R</td><td>G</td><td>V</td><td>I</td><td>A</td><td>S</td><td>S</td><td>A</td><td>G</td><td>N</td><td>H</td><td>A</td><td>Q</td><td>G</td><td>V</td><td>A</td><td>Y</td><td>G</td><td>A</td><td>K</td><td>E</td><td>S</td></tr>
<tr><td></td><td></td><td></td><td></td><td>G</td><td>I</td><td>K</td><td>A</td><td>V</td><td>I</td><td>V</td><td>M</td><td>P</td><td>K</td><td>S</td><td>T</td><td>P</td><td>L</td><td>I</td><td>K</td><td>V</td><td>E</td><td>S</td><td>T</td><td>K</td><td>Q</td><td>Y</td><td>G</td><td>A</td><td>E</td><td>V</td><td>I</td><td>L</td><td>H</td></tr>
<tr><td></td><td></td><td></td><td></td><td>G</td><td>D</td><td>V</td><td>Y</td><td>D</td><td>D</td><td>A</td><td>F</td><td>K</td><td>K</td><td>A</td><td>K</td><td>E</td><td>L</td><td>E</td><td>E</td><td>K</td><td>E</td><td>G</td><td>Y</td><td>V</td><td>F</td><td>V</td><td>H</td><td>P</td><td>F</td><td>N</td><td>D</td><td>E</td><td>D</td></tr>
<tr><td></td><td></td><td></td><td></td><td>V</td><td>L</td><td>D</td><td>G</td><td>Q</td><td>G</td><td>T</td><td>I</td><td>A</td><td>L</td><td>E</td><td>I</td><td>L</td><td>E</td><td>E</td><td>L</td><td>P</td><td>E</td><td>T</td><td>D</td><td>I</td><td>I</td><td>L</td><td>V</td><td>P</td><td>I</td><td>G</td><td>G</td><td>G</td><td>G</td></tr>
<tr><td></td><td></td><td></td><td></td><td>L</td><td>I</td><td>S</td><td>G</td><td>I</td><td>A</td><td>C</td><td>A</td><td>A</td><td>K</td><td>I</td><td>L</td><td>K</td><td>P</td><td>E</td><td>I</td><td>K</td><td>I</td><td>I</td><td>G</td><td>V</td><td>E</td><td>P</td><td>E</td><td>G</td><td>A</td><td>A</td><td>S</td><td>A</td><td>Y</td></tr>
<tr><td></td><td></td><td></td><td></td><td>E</td><td>A</td><td>I</td><td>K</td><td>E</td><td>N</td><td>K</td><td>V</td><td>V</td><td>E</td><td>L</td><td>K</td><td>E</td><td>A</td><td>N</td><td>T</td><td>I</td><td>A</td><td>D</td><td>G</td><td>T</td><td>A</td><td>V</td><td>K</td><td>K</td><td>I</td><td>G</td><td>D</td><td>L</td><td>N</td></tr>
<tr><td></td><td></td><td></td><td></td><td>F</td><td>E</td><td>Y</td><td>I</td><td>K</td><td>K</td><td>Y</td><td>V</td><td>D</td><td>E</td><td>I</td><td>I</td><td>T</td><td>V</td><td>S</td><td>D</td><td>Y</td><td>E</td><td>L</td><td>M</td><td>E</td><td>A</td><td>F</td><td>L</td><td>L</td><td>L</td><td>V</td><td>E</td><td>K</td><td>H</td></tr>
<tr><td></td><td></td><td></td><td></td><td>K</td><td>I</td><td>I</td><td>A</td><td>E</td><td>N</td><td>S</td><td>G</td><td>I</td><td>L</td><td>S</td><td>I</td><td>A</td><td>A</td><td>T</td><td>K</td><td>K</td><td>L</td><td>K</td><td>E</td><td>K</td><td>N</td><td>K</td><td>K</td><td>V</td><td>V</td><td>S</td><td>V</td><td>I</td><td>S</td></tr>
<tr><td></td><td></td><td></td><td></td><td>G</td><td>G</td><td>N</td><td>I</td><td>D</td><td>V</td><td>L</td><td>M</td><td>I</td><td>S</td><td>S</td><td>M</td><td>I</td><td>N</td><td>K</td><td>G</td><td>L</td><td>I</td><td>R</td><td>R</td><td>D</td><td>R</td><td>I</td><td>F</td><td>N</td><td>F</td><td>T</td><td>V</td><td>S</td><td>I</td></tr>
<tr><td></td><td></td><td></td><td></td><td>P</td><td>D</td><td>K</td><td>P</td><td>G</td><td>E</td><td>L</td><td>A</td><td>K</td><td>V</td><td>V</td><td>D</td><td>L</td><td>I</td><td>A</td><td>E</td><td>Q</td><td>G</td><td>A</td><td>N</td><td>V</td><td>I</td><td>K</td><td>L</td><td>E</td><td>H</td><td>N</td><td>Q</td><td>F</td><td>K</td></tr>
<tr><td></td><td></td><td></td><td></td><td>N</td><td>L</td><td>S</td><td>R</td><td>F</td><td>K</td><td>D</td><td>I</td><td>E</td><td>L</td><td>Q</td><td>I</td><td>T</td><td>V</td><td>E</td><td>T</td><td>N</td><td>G</td><td>S</td><td>E</td><td>H</td><td>V</td><td>Q</td><td>N</td><td>L</td><td>T</td><td>Q</td><td>A</td><td>F</td><td>E</td></tr>
<tr><td></td><td></td><td></td><td></td><td>E</td><td>K</td><td>G</td><td>Y</td><td>E</td><td>I</td><td>V</td><td>R</td><td>I</td><td>K</td><td>S</td><td>K</td><td>I</td><td>N</td><td colspan="16"></td></tr>
<tr><td></td><td></td><td></td><td></td><td>1</td><td></td><td></td><td></td><td>5</td><td></td><td></td><td></td><td></td><td>10</td><td></td><td></td><td></td><td></td><td>15</td><td></td><td></td><td></td><td></td><td>20</td><td></td><td></td><td></td><td></td><td>25</td><td></td><td></td><td></td><td></td><td>30</td></tr>
<tr><td colspan="34"></td></tr>
<tr><td>94</td><td>1.18%</td><td>99.0%</td><td colspan="31">>tr|A0A0M1VWL0|A0A0M1VWL0_FUSNV Uncharacterized protein OS=Fusobacterium nucleatum subsp. vincentii 4_1_13 OX=469606 GN=FSCG_01767 PE=4 SV=1</td></tr>
<tr><td></td><td></td><td></td><td></td><td>M</td><td>K</td><td>N</td><td>N</td><td>L</td><td>S</td><td>K</td><td>V</td><td>E</td><td>K</td><td>D</td><td>L</td><td>R</td><td>S</td><td>I</td><td>A</td><td>R</td><td>R</td><td>F</td><td>K</td><td>T</td><td>V</td><td>R</td><td>Y</td><td>S</td><td>I</td><td>G</td><td>L</td><td>A</td><td>V</td></tr>
<tr><td></td><td></td><td></td><td></td><td>L</td><td>F</td><td>L</td><td>M</td><td>L</td><td>G</td><td>I</td><td>N</td><td>A</td><td>F</td><td>S</td><td>E</td><td>E</td><td>T</td><td>V</td><td>T</td><td>R</td><td>E</td><td>I</td><td>I</td><td>Q</td><td>N</td><td>S</td><td>V</td><td>G</td><td>N</td><td>L</td><td>Q</td><td>S</td><td>K</td></tr>
<tr><td></td><td></td><td></td><td></td><td>I</td><td>E</td><td>T</td><td>L</td><td>K</td><td>V</td><td>E</td><td>N</td><td>E</td><td>K</td><td>A</td><td>L</td><td>K</td><td>G</td><td>L</td><td>R</td><td>L</td><td>E</td><td>L</td><td>V</td><td>Q</td><td>L</td><td>M</td><td>E</td><td>Q</td><td>G</td><td>N</td><td>Q</td><td>V</td><td>V</td></tr>
<tr><td></td><td></td><td></td><td></td><td>K</td><td>S</td><td>P</td><td>W</td><td>S</td><td>S</td><td>W</td><td>Q</td><td>F</td><td>G</td><td>L</td><td>N</td><td>Y</td><td>M</td><td>Y</td><td>S</td><td>R</td><td>W</td><td>G</td><td>G</td><td>A</td><td>Y</td><td>K</td><td>G</td><td>R</td><td>G</td><td>D</td><td>K</td><td>K</td><td>E</td></tr>
<tr><td></td><td></td><td></td><td></td><td>K</td><td>Y</td><td>P</td><td>Y</td><td>E</td><td>G</td><td>I</td><td>F</td><td>A</td><td>R</td><td>S</td><td>N</td><td>D</td><td>L</td><td>F</td><td>L</td><td>R</td><td>S</td><td>I</td><td>S</td><td>P</td><td>D</td><td>S</td><td>D</td><td>F</td><td>Y</td><td>E</td><td>K</td><td>Y</td><td>T</td></tr>
<tr><td></td><td></td><td></td><td></td><td>A</td><td>A</td><td>S</td><td>K</td><td>E</td><td>R</td><td>L</td><td>K</td><td>N</td><td>S</td><td>A</td><td>T</td><td>T</td><td>S</td><td>D</td><td>R</td><td>K</td><td>R</td><td>Q</td><td>G</td><td>L</td><td>R</td><td>D</td><td>S</td><td>S</td><td>Y</td><td>G</td><td>L</td><td>E</td><td>N</td></tr>
<tr><td></td><td></td><td></td><td></td><td>T</td><td>L</td><td>N</td><td>Q</td><td>Q</td><td>E</td><td>P</td><td>I</td><td>V</td><td>Q</td><td>I</td><td>E</td><td>L</td><td>G</td><td>A</td><td>S</td><td>V</td><td>R</td><td>P</td><td>R</td><td>E</td><td>I</td><td>V</td><td>K</td><td>S</td><td>P</td><td>V</td><td>N</td><td>V</td><td>T</td></tr>
<tr><td></td><td></td><td></td><td></td><td>A</td><td>P</td><td>R</td><td>I</td><td>T</td><td>V</td><td>S</td><td>P</td><td>V</td><td>T</td><td>P</td><td>F</td><td>S</td><td>K</td><td>P</td><td>S</td><td>A</td><td>P</td><td>S</td><td>E</td><td>P</td><td>T</td><td>A</td><td>P</td><td>T</td><td>I</td><td>D</td><td>I</td><td>K</td><td>G</td></tr>
<tr><td></td><td></td><td></td><td></td><td>F</td><td>D</td><td>P</td><td>A</td><td>A</td><td>P</td><td>D</td><td>V</td><td>T</td><td>A</td><td>P</td><td>D</td><td>L</td><td>P</td><td>I</td><td>A</td><td>P</td><td>T</td><td>F</td><td>N</td><td>I</td><td>Q</td><td>L</td><td>G</td><td>S</td><td>Y</td><td>R</td><td>N</td><td>Y</td><td>M</td></tr>
<tr><td></td><td></td><td></td><td></td><td>E</td><td>Q</td><td>N</td><td>F</td><td>L</td><td>H</td><td>D</td><td>P</td><td>D</td><td>G</td><td>G</td><td>R</td><td>F</td><td>S</td><td>G</td><td>D</td><td>G</td><td>K</td><td>S</td><td>Y</td><td>D</td><td>T</td><td>S</td><td>E</td><td>N</td><td>K</td><td>T</td><td>V</td><td>N</td><td>D</td></tr>
<tr><td></td><td></td><td></td><td></td><td>T</td><td>D</td><td>L</td><td>G</td><td>I</td><td>T</td><td>P</td><td>T</td><td>V</td><td>I</td><td>Y</td><td>A</td><td>W</td><td>A</td><td>T</td><td>P</td><td>S</td><td>R</td><td>Y</td><td>V</td><td>R</td><td>G</td><td>A</td><td>K</td><td>F</td><td>D</td><td>S</td><td>A</td><td>L</td><td>L</td></tr>
<tr><td></td><td></td><td></td><td></td><td>K</td><td>A</td><td>Y</td><td>F</td><td>D</td><td>Y</td><td>T</td><td>N</td><td>K</td><td>N</td><td>H</td><td>G</td><td>N</td><td>G</td><td>G</td><td>G</td><td>T</td><td>L</td><td>T</td><td>V</td><td>E</td><td>G</td><td>N</td><td>L</td><td>T</td><td>I</td><td>D</td><td>S</td><td>I</td><td>N</td></tr>
<tr><td></td><td></td><td></td><td></td><td>P</td><td>L</td><td>T</td><td>D</td><td>R</td><td>Q</td><td>K</td><td>E</td><td>N</td><td>E</td><td>T</td><td>N</td><td>A</td><td>G</td><td>R</td><td>A</td><td>H</td><td>N</td><td>A</td><td>Q</td><td>P</td><td>F</td><td>L</td><td>I</td><td>G</td><td>G</td><td>S</td><td>R</td><td>V</td><td>A</td></tr>
<tr><td></td><td></td><td></td><td></td><td>T</td><td>L</td><td>D</td><td>N</td><td>A</td><td>R</td><td>G</td><td>G</td><td>A</td><td>T</td><td>I</td><td>K</td><td>N</td><td>R</td><td>A</td><td>T</td><td>I</td><td>N</td><td>M</td><td>V</td><td>G</td><td>P</td><td>L</td><td>V</td><td>V</td><td>G</td><td>Y</td><td>E</td><td>I</td><td>Q</td></tr>
<tr><td></td><td></td><td></td><td></td><td>N</td><td>D</td><td>N</td><td>D</td><td>A</td><td>G</td><td>S</td><td>D</td><td>K</td><td>R</td><td>E</td><td>V</td><td>I</td><td>N</td><td>E</td><td>G</td><td>T</td><td>L</td><td>T</td><td>D</td><td>D</td><td>K</td><td>E</td><td>K</td><td>D</td><td>L</td><td>D</td><td>D</td><td>I</td><td>G</td></tr>
<tr><td></td><td></td><td></td><td></td><td>G</td><td>L</td><td>K</td><td>K</td><td>G</td><td>Q</td><td>I</td><td>G</td><td>G</td><td>G</td><td>K</td><td>N</td><td>T</td><td>L</td><td>S</td><td>D</td><td>I</td><td>I</td><td>E</td><td>L</td><td>T</td><td>R</td><td>P</td><td>Q</td><td>N</td><td>L</td><td>G</td><td>N</td><td>D</td><td>K</td></tr>
<tr><td></td><td></td><td></td><td></td><td>I</td><td>T</td><td>V</td><td>T</td><td>R</td><td>T</td><td>R</td><td>D</td><td>I</td><td>L</td><td>N</td><td>N</td><td>D</td><td>G</td><td>T</td><td>I</td><td>K</td><td>E</td><td>K</td><td>G</td><td>G</td><td>Y</td><td>T</td><td>G</td><td>Y</td><td>K</td><td>I</td><td>G</td><td>M</td><td>I</td></tr>
<tr><td></td><td></td><td></td><td></td><td>L</td><td>T</td><td>Q</td><td>E</td><td>L</td><td>D</td><td>T</td><td>P</td><td>N</td><td>P</td><td>G</td><td>N</td><td>N</td><td>Y</td><td>Y</td><td>R</td><td>L</td><td>I</td><td>N</td><td>D</td><td>G</td><td>K</td><td>I</td><td>S</td><td>F</td><td>M</td><td>G</td><td>R</td><td>N</td><td>S</td></tr>
<tr><td></td><td></td><td></td><td></td><td>I</td><td>G</td><td>I</td><td>Q</td><td>V</td><td>Y</td><td>A</td><td>K</td><td>P</td><td>D</td><td>N</td><td>T</td><td>P</td><td>S</td><td>T</td><td>I</td><td>I</td><td>D</td><td>V</td><td>I</td><td>N</td><td>K</td><td>E</td><td>N</td><td>G</td><td>N</td><td>G</td><td>A</td><td>G</td><td>E</td></tr>
<tr><td></td><td></td><td></td><td></td><td>I</td><td>T</td><td>L</td><td>G</td><td>G</td><td>I</td><td>E</td><td>S</td><td>Y</td><td>G</td><td>L</td><td>K</td><td>L</td><td>S</td><td>S</td><td>R</td><td>I</td><td>L</td><td>R</td><td>E</td><td>A</td><td>N</td><td>G</td><td>R</td><td>K</td><td>S</td><td>V</td><td>F</td><td>E</td><td>N</td></tr>
</table>

R	G	K	I	N	I	K	G	G	D	G	S	E	N	S	L	S	S	G	M	A	V	L	E	D	T	T	M	M	G
S	N	F	A	I	R	A	Y	K	G	M	V	V	N	K	G	T	I	N	V	S	G	G	K	G	N	T	G	M	I
L	K	V	D	A	E	D	D	I	T	N	D	T	N	G	I	I	N	V	S	G	T	A	N	I	G	M	R	V	D
K	G	A	V	P	T	G	A	S	G	T	P	E	A	I	N	N	G	T	I	N	V	S	G	S	A	T	K	A	K
D	G	N	I	G	M	V	A	H	K	Q	A	K	A	I	N	N	K	H	I	T	F	A	S	G	T	K	Y	G	T
G	L	L	A	K	G	A	G	A	K	I	E	N	K	G	G	N	A	K	I	T	G	S	G	L	E	R	T	I	G
M	S	A	L	Q	G	T	T	G	L	N	S	G	T	I	D	L	S	G	N	R	V	T	G	V	Y	N	E	G	T
F	N	M	T	G	G	L	L	K	A	S	G	N	Q	S	I	S	L	Y	S	K	G	S	S	S	T	T	N	I	T
G	G	T	I	A	A	G	D	K	A	V	G	L	Y	A	D	G	S	T	I	G	I	S	N	P	T	K	L	E	A
H	N	G	G	L	M	F	Y	N	Y	A	S	N	D	P	T	N	P	S	G	R	F	N	L	T	G	N	V	E	G
D	I	K	S	G	G	T	A	F	Y	F	K	G	A	A	S	N	T	A	S	F	L	N	Q	M	F	N	K	G	Q
A	S	S	T	G	K	L	K	L	K	L	E	D	G	A	T	L	F	V	L	D	S	P	G	G	A	P	I	K	L
S	T	V	R	T	L	G	T	A	L	G	D	K	V	D	I	S	R	T	T	S	N	K	Y	K	A	Y	T	V	Y
K	G	S	L	E	I	D	E	A	V	N	L	D	N	E	T	D	N	F	Y	K	M	D	F	T	A	S	N	V	T
I	N	E	N	I	N	V	T	G	S	K	A	G	K	V	I	I	A	Q	A	N	Y	N	G	A	T	S	S	S	T
I	K	V	T	N	K	G	K	I	D	Y	S	G	N	K	S	T	A	L	A	T	D	F	G	Q	V	T	N	E	T
S	G	T	I	R	M	S	G	D	N	S	I	G	L	Y	G	A	A	N	S	I	V	T	N	K	G	T	I	E	M
G	K	A	G	V	G	I	W	G	A	N	K	L	S	D	K	Y	A	N	R	N	I	N	I	V	N	S	G	T	I
R	G	I	S	G	E	E	G	I	F	G	I	Y	A	K	N	S	H	A	G	A	T	S	N	I	S	H	S	G	N
I	D	L	S	Q	A	K	K	S	T	G	I	F	M	T	K	G	T	L	N	S	S	G	N	I	S	V	N	E	G
S	V	G	V	N	A	E	D	S	T	V	N	V	N	G	G	T	H	T	I	G	A	N	S	I	G	F	N	L	K
G	N	S	S	K	L	L	A	N	S	G	N	I	S	I	T	G	K	G	S	V	A	Y	L	F	E	G	V	N	L
T	S	G	T	N	F	K	D	N	L	T	L	T	A	T	N	G	Y	T	Y	I	N	L	T	N	S	V	L	N	Y
K	N	Q	K	T	I	N	N	D	D	T	I	F	I	N	S	K	D	S	T	V	N	L	L	E	G	N	D	I	S
S	T	K	N	K	V	V	G	V	Y	S	E	G	G	A	I	S	N	A	G	K	I	T	L	M	G	N	G	S	S
A	L	Y	S	K	G	S	G	T	I	N	G	A	P	G	K	I	T	I	G	A	N	G	S	G	I	Y	V	V	N
A	G	S	T	G	S	N	Y	G	E	I	T	I	G	A	G	S	V	G	M	R	A	E	N	G	R	I	K	N	Y
S	T	G	K	I	S	S	T	A	E	K	A	T	G	M	S	Q	S	G	N	E	N	L	E	N	E	G	T	I	T
L	T	G	N	Q	S	V	G	M	H	S	E	G	V	T	A	A	G	H	Q	M	I	N	K	G	I	V	T	V	G
H	S	A	T	A	T	S	P	S	I	G	M	Y	A	A	N	T	D	K	T	T	I	I	N	N	G	K	V	I	A
G	N	K	S	T	G	I	Y	G	G	N	V	T	L	N	N	N	S	E	T	S	A	G	N	G	G	I	G	V	Y
S	K	G	G	T	V	D	I	K	E	N	A	K	I	S	V	G	D	T	L	G	D	K	Q	E	G	V	G	V	Y
L	A	G	N	N	Q	T	L	N	S	D	T	D	N	L	T	I	G	K	G	S	F	G	Y	V	M	T	G	Q	G
N	T	V	R	T	G	K	A	G	T	T	R	M	I	T	L	T	H	N	S	I	F	M	Y	S	A	D	R	T	G
T	A	V	N	Y	N	N	L	R	S	T	G	D	L	N	Y	G	I	Y	A	S	G	R	V	D	N	R	G	T	I
D	F	S	Q	G	I	G	N	I	G	A	Y	S	Y	T	K	G	A	T	T	T	P	N	S	I	R	N	Y	G	T
I	N	V	S	K	S	D	L	Q	T	N	P	D	D	R	K	Y	G	I	G	M	A	A	G	Y	S	E	E	S	P
A	G	S	G	R	K	V	T	R	G	I	G	S	I	E	N	H	G	L	I	R	V	T	T	P	D	S	I	G	M
Y	A	T	G	K	G	S	R	I	Y	N	G	P	T	G	R	I	E	L	S	G	R	K	R	N	I	G	I	F	A
E	N	G	A	E	V	V	N	E	G	T	I	T	T	V	G	S	G	N	V	G	Q	I	G	I	G	I	T	S	G
A	T	L	I	N	R	G	N	I	H	V	N	A	A	R	G	Y	G	L	F	V	A	G	G	I	V	K	N	Y	G
N	I	T	V	A	G	G	A	Q	K	T	K	E	V	S	A	A	D	T	S	K	A	L	G	D	E	G	L	D	R
V	G	I	K	S	P	A	G	A	S	K	G	T	I	T	S	N	G	K	V	K	R	P	T	I	V	R	A	I	P
N	R	K	P	S	E	I	P	K	S	S	I	G	M	Y	L	D	T	S	G	I	N	Y	T	K	P	I	N	N	V
G	A	L	A	G	L	K	Q	G	D	L	I	V	G	T	E	A	A	D	Y	T	N	S	K	Y	I	Q	L	G	Q
D	I	I	K	P	Y	N	E	M	I	R	K	S	G	I	E	K	W	S	I	Y	S	A	S	L	T	W	M	A	S
I	T	Q	L	P	D	Y	T	I	R	N	A	Y	L	V	K	I	P	Y	T	V	F	A	G	D	K	K	T	T	R
D	T	Y	N	F	T	D	G	L	E	Q	R	Y	G	V	E	G	L	N	S	R	E	K	E	L	F	K	K	L	N
K	I	G	N	N	E	R	I	L	L	Q	Q	A	F	D	E	M	M	G	H	Q	Y	A	N	V	Q	Q	R	I	Y
A	T	G	Q	I	L	D	K	E	F	D	Y	L	R	D	E	W	K	T	A	S	K	D	S	N	K	M	K	I	F
G	T	K	G	E	Y	K	T	D	T	A	G	V	I	D	Y	K	N	E	A	Y	G	M	A	Y	V	H	E	N	E
D	I	K	L	G	K	G	I	G	W	Y	T	G	I	V	D	N	T	F	K	F	K	D	I	G	R	S	K	E	E
Q	I	Q	A	K	V	G	L	L	K	S	I	P	F	D	D	N	N	S	L	N	W	T	I	S	G	D	I	F	V
G	Y	N	K	M	H	R	K	Y	L	V	V	N	E	I	F	N	A	R	S	K	Y	Y	T	Y	G	V	G	I	K
N	K	I	S	K	D	F	R	L	S	E	D	F	S	L	V	P	Y	G	S	L	N	L	E	Y	G	R	V	N	K
I	K	E	K	V	G	E	I	R	L	E	V	K	E	N	Y	Y	V	S	V	N	P	E	I	G	A	E	L	T	Y
K	H	L	L	A	S	R	K	T	F	R	V	G	L	G	I	A	Y	E	N	E	L	G	K	V	A	N	G	K	N
K	A	R	V	A	Y	T	N	A	D	W	F	N	I	R	G	E	K	E	D	R	K	G	N	I	K	F	D	L	N
I	G	L	D	N	Q	R	V	G	V	T	A	N	A	G	Y	D	T	K	G	H	N	V	R	G	G	L	G	L	R
V	I	F																											
1				5					10					15					20					25					30

95 7.83% 99.0% >sp|Q8RI55|ENO_FUSNN Enolase OS=Fusobacterium nucleatum subsp. nucleatum (strain ATCC 25586 / CIP 101130 / JCM 8532 / LMG 13131) OX=190304 GN=eno PE=3 SV=1

M	T	G	I	V	E	V	I	G	R	E	I	L	D	S	R	G	N	P	T	V	E	V	D	V	I	L	E	C	G
A	K	G	R	A	A	V	P	S	G	A	S	T	G	S	H	E	A	V	E	L	R	D	E	D	K	G	R	Y	L
G	K	G	V	L	K	A	V	N	N	V	N	T	E	I	R	E	A	L	L	G	M	D	A	L	N	Q	V	E	I
D	K	V	M	L	E	L	D	R	T	P	N	K	G	R	L	G	A	N	A	I	L	G	V	S	L	A	V	A	K
A	A	A	E	A	L	G	Q	P	L	Y	K	Y	L	G	G	V	N	S	K	E	L	P	L	P	M	M	N	I	L
N	G	G	A	H	A	D	S	A	V	D	L	Q	E	F	M	I	Q	P	V	G	A	K	S	F	R	E	A	M	Q
M	G	A	E	V	F	H	H	L	G	K	I	L	K	A	N	G	D	S	T	N	V	G	N	E	G	G	Y	A	P
S	K	I	Q	G	T	E	G	A	L	A	L	I	S	E	A	V	K	A	A	G	Y	E	L	G	K	D	I	T	F
A	L	D	A	A	S	S	E	F	C	K	E	V	N	G	K	Y	E	Y	H	F	K	R	E	G	G	V	V	R	T
T	D	E	M	I	K	W	Y	E	E	L	I	N	K	Y	P	I	V	S	I	E	D	G	L	G	E	D	D	W	D
G	W	V	K	L	T	K	A	I	G	D	R	V	Q	I	V	G	D	D	L	F	V	T	N	T	E	R	L	K	K
G	I	E	L	G	A	G	N	S	I	L	I	K	L	N	Q	I	G	S	L	T	E	T	L	D	A	I	E	M	A
K	R	A	G	Y	T	A	V	V	S	H	R	S	G	E	T	E	D	A	T	I	A	D	V	A	V	A	T	N	A
G	Q	I	K	T	G	S	T	S	R	T	D	R	M	A	K	Y	N	Q	L	L	R	I	E	E	E	L	G	S	V
A	Q	Y	N	G	R	D	V	F	Y	N	I	K	K																
1				5					10					15					20					25					30

96 5.91% 95.7% >tr|R9RAW2|R9RAW2_FUSNU Amino acid carrier protein OS=Fusobacterium nucleatum subsp. animalis 4_8 OX=469607 GN=HMPREF0409_00094 PE=3 SV=1

M	D	F	F	N	Y	I	I	G	Q	I	N	T	F	L	W	S	Y	V	L	I	V	L	L	L	L	S	G	L	F
Y	T	L	R	T	G	F	A	Q	G	R	L	L	G	D	M	V	A	L	I	T	G	K	L	S	S	L	R	D	G
E	K	K	I	A	G	Q	V	T	G	F	Q	A	F	C	I	A	V	A	S	H	V	G	T	G	N	L	A	G	V
A	I	A	V	S	I	G	G	P	G	A	L	F	W	M	W	I	I	A	L	L	G	A	A	T	S	M	I	E	N
T	L	A	Q	T	Y	K	V	K	D	G	K	G	G	F	R	G	G	P	S	Y	Y	M	E	K	A	L	G	Q	K
T	L	G	Y	I	F	S	V	I	V	I	L	T	F	A	F	I	F	N	T	V	Q	A	N	T	I	A	K	A	F
E	T	S	F	A	S	F	G	M	N	S	Y	I	A	A	I	I	L	A	A	L	T	A	L	V	I	F	G	G	L
H	R	I	A	N	V	V	S	L	M	V	P	I	M	A	I	A	Y	V	V	V	A	V	I	V	L	A	L	N	I
T	H	I	P	R	L	F	L	S	I	I	E	A	A	F	G	V	K	Q	A	V	G	G	A	I	G	V	A	M	L
Q	G	I	K	R	G	L	Y	S	N	E	A	G	M	G	S	A	P	N	A	A	T	S	N	V	S	H	P	V	
K	Q	G	L	L	Q	A	F	G	V	F	V	D	T	I	L	I	C	S	A	T	G	F	I	V	L	L	Y	P	E
Y	N	A	G	T	D	T	G	I	Q	L	T	Q	F	A	L	S	Y	S	V	G	A	W	G	A	H	F	I	T	L
C	I	F	L	F	A	F	S	S	L	I	G	N	Y	Y	Y	G	E	A	N	L	E	F	L	T	K	S	K	S	S
M	F	I	F	R	V	L	T	V	V	F	V	F	L	G	S	I	A	S	L	G	L	V	W	D	M	A	D	V	F
M	G	I	M	A	L	M	N	I	I	V	I	A	I	L	S	P	K	A	V	A	I	I	K	D	Y	I	K	Q	R
K	E	G	K	N	P	V	F	R	A	K	D	I	P	G	L	E	N	T	E	C	W	D	D						
1				5					10					15					20					25					30

97 7.60% 98.9% >sp|Q8RFY7|GSA_FUSNN Glutamate-1-semialdehyde 2,1-aminomutase OS=Fusobacterium nucleatum subsp. nucleatum (strain ATCC 25586 / CIP 101130 / JCM 8532 / LMG 13131) OX=190304 GN=hemL PE=3 SV=1

M	V	F	K	N	S	I	D	L	Y	K	K	A	V	E	L	I	P	G	G	V	N	S	P	V	R	A	F	K	S
V	N	R	E	A	P	I	F	I	K	K	G	Q	G	A	K	I	W	D	E	D	D	N	E	Y	I	D	Y	I	C
S	W	G	P	L	I	L	G	H	N	H	P	K	V	I	E	E	V	K	K	I	I	E	N	G	S	S	Y	G	L
P	T	K	Y	E	V	D	L	A	E	L	I	V	D	I	V	P	S	I	E	K	V	R	L	T	T	S	G	T	E
A	T	M	S	A	V	R	L	A	R	A	Y	T	Q	R	N	K	I	L	K	F	E	G	C	Y	H	G	H	S	D
A	L	L	V	K	S	G	S	G	L	L	T	E	G	Y	Q	D	S	N	G	I	T	D	G	V	L	K	D	T	L
T	L	P	F	G	D	I	E	K	V	K	E	I	L	K	N	K	D	V	A	C	V	I	V	E	P	I	P	A	N
M	G	L	I	E	T	H	K	E	F	L	Q	G	L	G	K	V	T	E	K	T	G	T	I	L	I	F	D	E	V
I	S	G	F	R	L	A	L	G	G	A	Q	E	F	F	G	I	T	P	D	L	T	T	L	G	K	I	I	G	G
G	Y	P	V	G	A	F	G	G	K	K	E	I	M	D	L	V	A	P	V	G	R	V	Y	H	A	G	T	L	S
G	N	P	I	A	S	K	A	G	F	A	T	I	S	Y	L	K	E	N	P	N	I	Y	K	E	L	E	E	K	T
N	Y	L	I	D	N	I	E	I	L	A	K	K	Y	S	V	N	V	C	V	N	S	M	G	S	L	F	T	I	F
F	V	D	I	D	K	V	E	N	L	E	D	S	L	K	S	N	T	E	N	F	S	I	Y	F	N	T	M	L	E
N	G	I	V	I	P	P	S	Q	F	E	A	H	F	L	S	M	A	H	T	K	K	E	L	N	R	T	L	E	V
I	E	M	A	F	K	K	I	G	E	K	S	G	K																
1				5					10					15					20					25					30

98 2.46% 98.8% >sp|Q8RG86|CARB_FUSNN Carbamoyl-phosphate synthase large chain OS=Fusobacterium nucleatum subsp. nucleatum (strain ATCC 25586 / CIP 101130 / JCM 8532 / LMG 13131) OX=190304 GN=carB PE=3 SV=2

M	P	K	R	K	D	I	K	T	I	L	V	I	G	S	G	P	I	I	I	G	Q	A	A	E	F	D	Y	A	G
T	Q	A	C	L	S	L	R	E	E	G	Y	E	V	I	L	V	N	S	N	P	A	T	I	M	T	D	K	E	I
A	D	K	V	Y	I	E	P	L	T	V	E	F	L	S	K	I	I	R	K	E	K	P	D	A	L	L	P	T	L
G	G	Q	V	A	L	N	L	A	V	S	L	H	E	S	G	I	L	D	E	C	G	V	E	I	L	G	T	K	L
T	S	I	K	Q	A	E	D	R	E	L	F	R	D	L	M	N	E	L	N	E	P	V	P	D	S	A	I	V	H
T	L	E	E	A	E	N	F	V	K	E	I	D	Y	P	V	I	V	R	P	A	F	T	M	G	G	T	G	G	G
I	C	Y	N	E	E	D	L	H	E	I	V	P	N	G	L	N	Y	S	P	V	H	Q	C	L	L	E	K	S	I
A	G	Y	K	E	I	E	Y	E	V	M	R	D	S	N	D	T	A	I	V	V	C	N	M	E	N	I	D	P	V
G	I	H	T	G	D	S	I	V	V	A	P	S	Q	T	L	T	D	R	E	H	H	M	L	R	D	V	S	L	K
I	I	R	A	L	K	I	E	G	G	C	N	V	Q	I	A	L	D	P	N	S	F	K	Y	Y	I	I	E	V	N
P	R	V	S	R	S	S	A	L	A	S	K	A	T	G	Y	P	I	A	K	I	A	A	K	I	A	V	G	M	T
L	D	E	I	I	N	P	V	T	K	S	S	Y	A	C	F	E	P	A	I	D	Y	V	V	T	K	I	P	R	F
P	F	D	K	F	G	D	G	D	R	Y	L	G	T	Q	M	K	A	T	G	E	V	M	A	I	G	R	T	L	E
E	S	L	L	K	A	I	R	S	L	E	Y	G	V	H	H	L	G	L	P	N	G	E	E	F	S	L	E	K	I
I	K	R	I	K	L	A	G	D	E	R	L	F	F	I	G	E	A	L	R	R	D	V	S	I	E	E	I	H	E
Y	T	K	I	D	L	F	F	L	N	K	M	K	N	I	I	D	L	E	H	L	L	K	D	N	K	G	N	I	E
L	L	R	K	V	K	T	F	G	F	S	D	R	V	I	A	H	R	W	E	M	T	E	P	E	I	T	E	L	R
H	K	H	N	I	R	P	V	Y	K	M	V	D	T	C	A	A	E	F	D	S	N	T	P	Y	F	Y	S	T	Y
E	F	E	N	E	S	T	R	S	D	K	E	K	I	V	V	L	G	S	G	P	I	R	I	G	Q	G	I	E	F
D	Y	A	T	V	H	A	I	M	A	I	K	K	L	G	Y	E	A	I	V	I	N	N	N	P	E	T	V	S	T
D	F	S	I	S	D	K	L	Y	F	E	P	L	T	Q	E	D	V	M	E	I	L	D	L	E	K	P	L	G	V
V	V	Q	F	G	G	Q	T	A	I	N	L	A	D	K	L	V	K	N	G	I	Q	I	L	G	S	S	L	D	S
I	D	T	A	E	D	R	D	R	F	E	K	L	L	I	G	L	K	I	P	Q	P	L	G	K	T	A	F	D	V

				E	T	A	L	K	N	A	N	E	I	G	Y	P	V	L	V	R	P	S	Y	V	L	G	G	R	A	M	E	I	V
				Y	N	D	E	D	L	T	K	Y	M	E	K	A	V	H	I	N	P	D	H	P	V	L	I	D	R	Y	L	I	G
				K	E	I	E	V	D	A	I	S	D	G	E	N	T	F	I	P	G	I	M	E	H	I	E	R	A	G	V	H	S
				G	D	S	I	S	I	Y	P	P	Q	S	L	S	E	K	E	I	E	T	L	I	D	Y	T	K	K	L	A	S	G
				L	E	V	K	G	L	I	N	I	Q	Y	V	V	S	K	G	E	I	Y	V	L	E	V	N	P	R	A	S	R	T
				V	P	F	L	S	K	V	T	G	V	P	V	A	N	I	A	M	Q	C	I	L	G	K	K	L	K	D	L	G	F
				T	K	D	I	A	D	I	G	N	F	V	S	V	K	V	P	V	F	S	F	Q	K	L	K	N	V	D	T	T	L
				G	P	E	M	K	S	T	G	E	V	I	G	T	D	V	N	L	Q	K	A	L	Y	K	G	L	T	A	A	G	I
				K	I	K	D	Y	G	R	V	L	F	T	I	D	D	K	N	K	E	A	A	L	N	L	A	K	G	F	S	D	V
				G	F	S	I	L	T	T	E	G	T	G	I	Y	F	E	E	Y	G	L	K	V	K	K	V	G	K	I	D	N	S
				D	Y	S	V	L	D	A	I	Q	N	G	D	V	D	I	V	I	N	T	T	T	K	G	K	S	S	E	K	D	G
				F	R	I	R	R	K	A	T	E	Y	G	V	I	C	F	T	S	L	D	T	A	N	A	L	L	R	V	I	E	S
				M	S	F	R	V	Q	T	L																						
				1				5					10					15					20					25					30
99	7.08%	98.1%	>tr|R9RA98|R9RA98_FUSNU Uncharacterized protein OS=Fusobacterium nucleatum subsp. animalis 4_8 OX=469607 GN=HMPREF0409_01185 PE=4 SV=1																														
				M	D	L	K	E	K	V	L	E	Y	K	D	E	V	V	K	E	I	Q	N	A	V	R	V	K	S	V	K	E	A
				P	L	P	G	M	P	F	G	E	G	P	A	K	A	L	D	H	F	M	N	L	A	K	K	L	G	F	K	T	E
				K	F	D	N	Y	A	M	H	I	D	M	G	E	G	K	E	T	L	G	I	L	A	H	V	D	V	V	P	E	G
				D	N	W	T	Y	P	P	Y	S	G	T	I	A	D	G	K	I	Y	G	R	G	T	L	D	D	K	G	P	A	I
				I	S	L	F	A	M	K	A	I	A	D	S	G	I	K	L	N	K	K	I	R	M	I	L	G	A	D	E	E	S
				G	S	A	C	L	K	Y	Y	F	G	E	L	K	M	P	Y	P	D	I	A	F	T	P	D	S	S	F	P	V	T
				Y	A	E	K	G	S	V	R	V	K	I	K	K	K	F	S	T	L	K	D	V	V	I	K	G	G	N	A	F	N
				S	V	P	N	E	A	N	G	V	I	P	V	D	M	L	G	E	V	K	N	K	N	K	V	E	F	V	K	E	G
				N	V	Y	K	I	F	S	A	G	I	P	A	H	G	A	H	P	E	K	G	Y	N	A	I	S	A	L	F	E	V
				L	K	D	I	E	V	K	N	E	E	L	K	G	L	V	A	F	F	D	K	F	I	K	M	E	T	D	G	K	S
				F	G	V	K	C	T	D	G	E	T	G	D	L	T	L	N	L	G	K	I	N	L	E	N	N	E	L	E	I	W
				I	D	M	R	V	P	V	K	V	K	N	E	Q	I	I	E	T	I	K	K	N	T	E	D	Y	G	Y	E	F	L
				L	H	S	N	T	Q	P	L	Y	V	A	K	D	S	F	L	V	S	T	L	M	N	I	Y	K	E	L	T	G	D
				N	A	A	Q	P	V	A	I	G	G	G	T	Y	A	K	Y	A	K	N	A	V	A	F	G	A	L	L	P	D	Q
				E	D	R	M	H	Q	R	D	E	Y	L	E	I	S	K	I	D	K	L	L	Q	I	Y	V	E	A	I	Y	R	L
				A	K																												
				1				5					10					15					20					25					30
100	2.04%	95.2%	>sp|Q8R5Z1|IF2_FUSNN Translation initiation factor IF-2 OS=Fusobacterium nucleatum subsp. nucleatum (strain ATCC 25586 / CIP 101130 / JCM 8532 / LMG 13131) OX=190304 GN=infB PE=3 SV=1																														
				M	K	V	R	V	H	E	L	A	K	K	Y	E	I	K	N	K	E	F	L	E	I	L	K	K	D	I	G	I	T
				V	T	S	H	L	S	N	L	D	E	D	Q	V	N	K	I	D	D	Y	F	A	K	M	N	M	L	K	V	E	T
				V	E	P	V	K	V	H	K	E	K	K	E	E	K	P	I	R	K	I	M	D	E	D	E	N	D	E	G	E	G
				Y	S	Q	K	N	N	K	K	A	K	F	Q	Q	T	K	N	K	K	N	N	N	I	T	F	E	E	D	G	N	S
				H	K	N	K	G	K	K	K	K	G	R	R	T	D	F	I	L	K	T	V	E	A	T	P	D	V	V	E	E	D
				G	I	K	I	I	K	F	R	G	E	L	T	L	G	D	F	A	E	K	L	G	V	N	S	A	E	I	I	K	K
				L	F	L	K	G	Q	M	L	T	I	N	S	P	I	T	L	S	M	A	E	D	L	A	A	D	Y	D	V	L	I
				E	E	E	Q	E	V	E	L	D	F	G	E	K	F	D	L	E	I	E	D	K	A	A	D	L	K	E	R	P	P
				V	I	T	I	M	G	H	V	D	H	G	K	T	S	L	L	D	A	I	R	T	T	N	V	V	G	G	E	A	G
				G	I	T	Q	K	I	G	A	Y	Q	V	E	R	D	G	K	R	I	T	F	I	D	T	P	G	H	E	A	F	T
				D	M	R	A	R	G	A	Q	V	T	D	I	A	I	L	V	V	A	A	D	D	G	V	M	P	Q	T	V	E	A
				I	S	H	A	K	V	A	K	V	P	I	I	V	A	V	N	K	I	D	K	P	E	A	N	P	M	K	V	K	Q
				E	L	M	E	H	G	L	V	S	A	E	W	G	G	D	V	E	F	V	E	V	S	A	K	Q	K	I	N	L	D
				G	L	L	D	T	I	L	I	T	A	E	I	L	E	L	K	G	N	N	K	K	R	A	K	G	V	V	L	E	S
				R	L	D	P	K	I	G	P	I	A	D	I	L	V	Q	E	G	T	L	K	I	G	D	V	I	V	A	G	E	V
				Q	G	K	V	K	A	L	L	N	D	K	G	E	R	V	N	N	A	T	V	S	Q	P	V	E	V	I	G	F	N
				N	V	P	D	A	G	D	T	M	Y	V	I	Q	N	E	Q	H	A	K	R	I	V	E	E	V	R	K	E	R	K
				I	Q	E	T	T	K	K	T	I	S	L	E	S	L	S	D	Q	F	K	H	E	D	L	K	E	L	N	L	I	L
				R	A	D	S	K	G	S	V	D	A	L	R	D	S	L	L	K	L	S	N	D	E	V	A	V	S	I	I	Q	A
				A	S	G	A	I	T	E	S	D	V	K	L	A	E	A	A	G	A	I	I	I	G	Y	N	V	R	P	T	T	K
				A	L	K	E	A	E	V	S	K	V	E	I	R	T	S	G	I	I	Y	H	I	T	E	D	I	E	K	A	L	A
				G	M	L	E	P	E	Y	R	E	E	Y	L	G	R	I	E	I	K	K	V	F	K	V	S	K	V	G	N	I	A
				G	C	I	V	I	D	G	K	V	K	N	D	S	N	I	R	I	L	R	D	N	V	V	I	Y	E	G	K	L	A
				S	L	K	R	F	K	D	D	A	K	E	V	V	A	G	Q	E	C	G	L	G	V	E	N	F	N	D	I	K	D
				G	D	V	V	E	A	F	E	M	V	E	V	K	R	T	L	K													
				1				5					10					15					20					25					30
101	1.33%	83.9%	>tr|Q8REY5|Q8REY5_FUSNN DNA helicase OS=Fusobacterium nucleatum subsp. nucleatum (strain ATCC 25586 / CIP 101130 / JCM 8532 / LMG 13131) OX=190304 GN=FN0949 PE=4 SV=1																														
				M	D	N	K	N	N	L	S	Y	Q	K	I	L	Q	V	E	K	L	P	F	L	K	P	L	A	I	D	E	E	L
				L	E	W	I	S	G	D	W	G	D	Y	K	S	S	V	K	L	L	S	E	K	I	I	K	E	N	N	S	L	K
				V	A	N	I	S	D	E	E	K	E	I	L	E	K	L	L	K	D	R	Q	L	W	I	E	E	Q	K	K	I	E
				N	V	R	K	L	F	D	V	L	Y	S	K	Y	L	S	L	D	R	D	S	D	T	L	E	L	L	V	G	N	G
				I	V	K	V	P	N	E	D	I	C	Y	P	V	L	L	K	K	V	N	F	S	L	D	A	E	R	N	L	I	T
				I	V	D	S	S	D	D	D	F	I	T	Q	E	L	Y	L	N	F	L	A	E	V	E	N	V	N	L	D	N	V
				F	K	L	G	D	K	I	I	E	N	N	I	H	P	I	S	K	N	D	V	I	K	D	F	F	R	E	F	I	H
				N	L	N	P	R	A	E	F	I	E	D	K	K	I	S	D	E	D	N	I	I	T	I	E	W	K	P	I	L	F
				I	R	K	K	D	D	G	K	I	E	A	I	N	N	I	I	K	D	I	E	E	G	G	E	V	P	G	Y	L	T
				E	L	V	G	I	I	E	N	D	K	K	E	I	E	D	I	P	D	I	L	F	T	K	E	T	N	N	E	Q	V
				E	I	I	K	N	I	Y	S	H	K	A	V	V	V	Q	G	P	P	G	T	G	K	T	H	T	I	A	N	L	L
				G	H	F	L	A	E	G	K	N	V	L	I	T	S	Q	T	R	K	A	L	E	V	L	K	E	K	I	P	N	E
				I	Q	D	L	C	I	S	M	L	D	D	D	S	S	D	L	G	N	S	V	E	S	I	S	E	K	L	G	Y	L
				N	L	E	K	L	K	N	E	Y	E	E	I	E	R	N	R	N	D	L	K	E	D	I	K	N	I	K	R	K	I
				F	N	I	K	Y	Q	E	S	K	P	I	I	Y	N	N	E	S	I	S	L	K	E	A	G	E	F	L	R	K	N
				E	R	E	L	D	K	I	P	G	I	V	S	S	G	V	L	C	P	V	N	N	E	E	L	E	F	L	K	T	G
				Y	K	K	S	V	S	K	E	E	E	K	E	I	E	L	G	L	N	K	I	S	D	F	W	S	L	E	E	F	E
				G	M	L	K	A	K	K	E	F	K	S	E	I	E	L	L	L	E	N	K	K	Y	H	I	N	D	E	I	L	Y
				I	D	D	N	I	V	I	D	L	K	K	F	K	N	Y	T	N	I	D	N	I	I	P	E	E	L	K	L	I	E
				D	W	K	K	D	V	C	I	A	G	T	E	N	S	G	D	R	K	I	W	L	D	F	I	K	D	I	R	R	L
				Y	E	L	T	N	N	T	K	D	K	F	F	K	K	D	I	V	Y	K	D	I	D	V	S	T	A	K	K	L	I
				I	A	L	K	D	G	L	E	K	P	G	L	F	F	K	H	K	L	R	K	A	K	K	E	I	A	D	R	V	T
				I	N	K	R	I	L	E	I	P	Y	D	C	D	V	A	L	E	Y	T	S	L	A	E	L	E	E	N	T	K	N
				S	W	K	L	L	M	T	G	N	T	L	M	D	K	E	S	N	N	K	N	F	F	K	Q	L	Y	S	Y	A	D
				Q	M	E	Y	L	L	N	W	Y	D	K	E	R	K	I	F	L	N	R	V	E	N	A	G	F	E	R	L	D	I
				S	K	K	E	G	S	P	I	Y	V	D	E	I	N	Q	I	F	D	F	I	P	K	L	E	E	L	I	T	I	G
				K	V	G	L	K	Y	S	E	I	D	K	K	R	T	E	Y	L	E	K	I	E	G	I	I	K	E	N	S	F	L
				G	S	E	I	K	N	A	I	E	K	E	N	T	E	K	Y	S	E	T	L	K	K	L	E	V	L	A	G	K	E
				E	L	Y	R	K	H	K	N	L	L	K	N	I	K	A	V	A	N	L	W	A	D	E	L	E	K	G	L	F	N
				E	K	V	E	N	I	Y	N	A	W	R	Y	K	Q	I	S	Q	T	L	K	E	L	I	E	K	P	Y	E	S	L
				Q	E	D	I	L	E	K	S	E	E	L	N	K	L	T	A	E	L	V	T	K	K	T	W	Y	N	I	V	K	F
				I	E	E	K	D	N	L	A	I	S	Q	A	L	R	G	W	K	Q	T	I	Q	K	I	G	K	G	T	G	K	N
				T	V	L	Y	K	K	H	A	K	E	K	M	L	L	C	Q	K	V	V	P	A	W	I	M	P	L	N	K	V	F
				D	T	L	N	P	V	E	N	K	F	D	I	V	I	V	D	E	A	S	Q	S	D	I	S	S	L	I	L	L	Y
				M	A	K	K	I	I	V	V	G	D	D	K	Q	V	S	P	S	D	V	G	V	N	I	D	K	I	N	M	F	R
				R	K	Y	I	K	G	N	V	A	N	D	D	L	Y	G	V	R	A	S	L	Y	S	I	V	S	T	T	F	Q	P
				I	S	L	R	E	H	F	R	S	V	P	E	I	I	G	Y	S	N	K	T	S	Y	D	N	Q	I	L	P	L	R
				D	S	N	S	S	I	L	K	P	A	I	V	E	Y	K	V	D	G	K	R	D	E	K	N	K	V	N	K	V	E
				A	E	T	I	V	T	L	I	E	A	C	L	D	M	K	E	Y	K	N	S	S	F	G	V	I	S	L	L	G	D
				E	Q	A	E	L	I	Q	N	L	I	V	K	R	I	P	A	I	E	I	E	N	H	K	I	L	C	G	N	P	A
				S	F	Q	G	D	E	R	D	V	M	F	I	S	L	V	D	S	S	E	E	N	K	N	L	R	L	V	G	E	G
				V	E	G	A	T	R	K	R	Y	N	V	A	I	S	R	A	K	D	Q	L	W	L	V	H	S	I	D	K	N	S
				L	K	E	G	D	L	R	K	E	L	F	E	Y	I	N	S	V	K	E	N	T	F	E	K	I	I	N	E	N	T
				V	I	S	D	F	E	N	E	I	T	K	H	L	L	E	R	N	Y	T	V	K	Q	Q	W	K	V	G	S	Y	D
				I	D	I	V	A	I	Y	G	D	K	K	I	A	I	E	C	D	G	K	N	L	N	H	S	Q	E	K	I	I	A
				N	L	A	E	Q	E	V	L	E	R	C	G	W	E	F	V	R	V	R	A	S	Q	Y	F	R	N	P	D	K	A
				I	K	E	L	I	L	Q	L	E	D	K	G	I	Y	P	N	N	K	E	N	H	S	N	G	N	E	L	L	N	N
				I	K	S	K	A	L	E	L	M	E	K	Y	E	E	N															
				1				5					10					15					20					25					30
102	10.60%	91.4%	>tr|X7RY36|X7RY36_FUSNU Uncharacterized protein OS=Fusobacterium nucleatum 13_3C OX=1357398 GN=HMPREF2085_01772 PE=4 SV=1																														
				M	R	Q	K	I	V	S	M	E	E	A	I	S	H	I	K	D	G	M	T	V	H	F	G	G	F	L	A	C	G
				T	A	E	S	V	V	T	A	L	V	E	K	G	V	K	D	L	T	I	V	C	N	D	T	G	F	V	D	K	G
				V	G	R	L	V	V	N	N	Q	V	K	K	V	I	A	S	H	I	G	T	N	A	E	T	G	K	K	M	H	D
				G	T	M	E	V	E	L	V	P	Q	G	T	L	A	E	R	V	R	A	A	G	Y	G	L	G	G	I	L	T	P
				T	G	L	G	T	I	V	Q	E	G	K	Q	I	I	N	V	D	G	K	D	Y	L	L	E	K	P	I	K	A	D
				V	A	V	L	F	G	T	K	V	D	E	L	G	N	V	I	C	E	K	T	T	K	N	F	N	P	L	M	A	M
				A	A	D	T	V	I	V	E	A	L	E	I	V	P	A	G	S	L	S	P	E	H	L	D	I	S	R	I	F	I
				D	Y	I	V	K	S	K																							
				1				5					10					15					20					25					30
103	4.43%	90.7%	>sp|Q8RHM2|KPRS_FUSNN Ribose-phosphate pyrophosphokinase OS=Fusobacterium nucleatum subsp. nucleatum (strain ATCC 25586 / CIP 101130 / JCM 8532 / LMG 13131) OX=190304 GN=prs PE=3 SV=1																														
				M	I	N	F	N	N	V	K	I	F	S	G	N	S	N	L	E	L	A	K	K	I	A	E	K	A	G	L	Q	L
				G	K	A	E	I	Q	R	F	K	D	G	E	V	Y	I	E	I	E	E	T	V	R	G	R	D	V	F	V	V	Q
				S	T	S	E	P	V	N	E	N	L	M	E	L	L	I	F	V	D	A	L	K	R	A	S	A	K	T	I	N	V
				I	I	P	Y	Y	G	Y	A	R	Q	D	R	K	S	K	P	R	E	P	I	T	S	K	L	V	A	N	L	L	T
				T	A	G	V	N	R	V	V	A	M	D	L	H	A	D	Q	I	Q	G	F	F	D	I	P	L	D	H	M	Q	A
				L	P	L	M	A	R	Y	F	K	E	K	G	F	K	G	D	E	V	V	V	V	S	P	D	V	G	G	V	K	R
				A	R	K	L	A	E	K	L	D	C	K	I	A	I	I	D	K	R	R	P	K	P	N	M	S	E	V	M	N	L
				I	G	E	V	E	G	K	I	A	I	F	I	D	D	M	I	D	T	A	G	T	I	T	N	G	A	D	A	I	A
				Q	R	G	A	K	E	V	Y	A	C	C	T	H	A	V	F	S	D	P	A	I	E	R	L	E	K	S	V	L	K
				E	I	V	I	T	D	S	I	A	L	P	E	R	K	K	I	D	K	I	K	I	L	S	V	D	S	V	F	A	N
				A	I	D	R	I	T	N	N	Q	S	V	S	E	L	F	N														
				1				5					10					15					20					25					30
104	3.05%	94.9%	>tr|R9RBQ7|R9RBQ7_FUSNU Translation elongation factor G OS=Fusobacterium nucleatum subsp. animalis 4_8 OX=469607 GN=HMPREF0409_01714 PE=4 SV=1																														
				M	K	V	F	T	T	E	N	I	R	N	I	S	L	L	G	H	R	G	S	G	K	T	T	L	V	E	S	I	L
				Y	V	K	G	Y	I	K	R	K	G	D	V	E	N	G	T	T	V	S	D	F	D	K	E	E	I	R	R	I	F

S	I	N	T	S	L	I	S	V	E	Q	N	D	V	K	**L**	**N**	**F**	**L**	**D**	**T**	**P**	**G**	**Y**	**F**	**D**	**F**	**V**	**G**	**E**
**V**	**V**	**S**	**A**	**L**	**R**	V	S	A	S	A	V	L	V	L	D	A	T	A	G	V	E	V	G	T	E	K	A	W	K
L	L	E	E	R	K	L	P	R	I	I	F	V	N	K	M	D	K	G	Y	V	N	Y	P	K	L	L	A	E	L
K	E	K	F	G	K	K	I	A	P	F	C	I	P	I	G	E	K	E	E	F	K	G	F	V	N	V	V	D	M
V	G	R	V	F	D	G	K	E	C	V	D	T	P	I	P	K	D	I	D	V	S	E	V	R	N	L	L	F	E
A	I	A	E	T	D	E	V	L	M	D	K	Y	F	A	G	E	E	F	T	Q	E	E	I	V	K	G	L	H	K
G	V	V	N	G	D	I	V	P	V	M	V	G	S	A	Q	Q	N	I	G	I	H	T	L	L	N	Y	L	E	L
Y	M	P	C	P	T	E	L	F	S	G	Q	R	I	G	E	D	P	T	T	Q	Q	E	K	V	V	K	I	S	S
E	N	P	F	S	A	I	V	F	K	T	L	V	D	P	F	I	G	K	I	S	F	F	K	V	N	S	G	V	I
K	K	E	T	E	V	F	N	P	K	K	N	K	K	E	R	I	A	Q	I	I	T	M	Q	G	N	K	Q	I	E
L	E	E	L	H	A	G	D	I	G	A	T	T	K	L	Q	F	T	Q	T	G	D	T	L	C	D	K	N	F	P
V	V	F	N	K	I	R	F	P	K	P	N	I	Y	S	G	V	V	P	A	N	K	N	D	D	E	K	L	S	T
A	L	Q	R	V	M	E	E	D	P	T	F	V	M	S	R	N	Y	E	T	K	Q	L	L	I	G	G	Q	G	E
K	H	L	Y	I	I	L	C	K	I	K	N	K	F	G	V	H	A	E	L	Q	D	V	I	V	S	Y	R	E	T
I	L	G	K	A	E	V	E	G	K	H	K	K	Q	S	G	G	A	G	Q	Y	G	D	V	F	I	R	F	E	H
S	D	K	D	F	E	F	V	D	E	I	K	G	G	V	V	P	R	N	Y	I	P	A	V	E	K	G	L	I	E
A	K	E	K	G	V	L	A	G	Y	P	V	I	N	F	K	A	T	L	Y	D	G	S	Y	H	P	V	D	S	N
D	L	S	F	K	L	A	A	I	L	A	F	K	A	G	M	E	K	A	K	P	V	L	L	E	P	F	V	R	M
E	I	R	I	P	E	E	Y	M	G	D	V	M	G	D	L	N	K	R	R	G	R	V	L	G	M	D	H	T	E
T	G	E	Q	L	L	L	A	E	V	P	E	A	E	I	L	K	Y	S	I	D	L	R	A	L	T	Q	G	R	G
E	F	E	Y	E	F	V	R	Y	E	E	V	P	E	N	I	S	K	R	I	I	E	E	R	N	K	D	K		
1				5					10					15					20					25					30

105 4.46% 86.6% >tr|A0A117MWD9|A0A117MWD9_FUSNC UDP-N-acetylglucosamine 4,6-dehydratase (Inverting) OS=Fusobacterium nucleatum subsp. nucleatum OX=76856 GN=RO03_08765 PE=4 SV=1

M	L	N	N	S	T	I	L	I	T	G	G	T	G	S	F	G	N	R	F	I	E	R	I	L	S	E	Y	N	P
K	K	I	I	I	Y	S	R	D	E	F	K	Q	D	L	M	K	K	N	F	V	M	K	Y	G	V	E	K	T	K
K	L	R	F	F	I	G	D	V	R	D	K	E	R	L	Y	R	**A**	**F**	**D**	**G**	**V**	**D**	**Y**	**V**	**I**	**H**	**A**	**A**	**A**
**M**	**K**	Q	V	P	A	C	E	Y	N	P	F	E	A	I	K	T	N	I	H	G	A	E	N	V	I	E	A	A	I
D	R	G	V	K	K	V	V	A	L	S	T	D	K	A	V	N	P	I	N	L	Y	G	G	T	K	L	V	S	D
K	L	F	I	S	A	N	A	Y	S	G	E	K	G	T	I	F	S	V	V	R	Y	G	N	V	A	G	S	R	G
S	V	I	P	F	F	K	Q	L	L	S	Q	G	K	T	E	L	P	I	T	D	I	H	M	T	R	F	W	M	V
L	D	D	A	V	N	L	V	L	K	A	L	K	E	S	K	G	G	E	T	F	V	F	K	N	P	S	F	L	I
T	E	L	A	K	A	L	N	P	N	G	I	I	K	E	V	G	I	R	E	G	E	K	I	H	E	V	M	I	T
R	D	D	A	K	Y	T	Y	D	Y	E	D	Y	Y	V	I	Y	P	N	F	E	W	W	N	K	E	K	I	K	S
G	G	K	L	I	P	E	N	W	D	Y	N	S	G	T	N	D	W	W	L	D	A	K	E	L	E	K	R	I	A
K	L	D	I	K	Y																								
1				5					10					15					20					25					30

106 19.28% 94.5% >sp|Q8RIG4|RS17_FUSNN 30S ribosomal protein S17 OS=Fusobacterium nucleatum subsp. nucleatum (strain ATCC 25586 / CIP 101130 / JCM 8532 / LMG 13131) OX=190304 GN=rpsQ PE=3 SV=1

M	R	N	E	R	K	V	R	E	G	I	V	V	S	D	K	M	Q	K	**T**	**I**	**V**	**V**	**A**	**I**	**E**	**T**	**M**	**I**	**L**
**H**	**P**	**I**	**Y**	**K**	K	R	V	K	R	T	T	K	F	K	A	H	D	E	E	N	V	A	Q	V	G	D	K	V	K
I	M	E	T	R	R	L	S	K	D	K	N	W	R	L	V	E	I	I	E	K	A	R							
1				5					10					15					20					25					30

107 9.62% 90.2% >sp|Q8RIM0|RS7_FUSNN 30S ribosomal protein S7 OS=Fusobacterium nucleatum subsp. nucleatum (strain ATCC 25586 / CIP 101130 / JCM 8532 / LMG 13131) OX=190304 GN=rpsG PE=3 SV=1

M	S	R	R	R	A	A	V	K	R	D	V	L	P	D	S	R	Y	S	D	K	V	V	T	K	V	I	N	S	I
M	L	D	G	K	K	**S**	**I**	**A**	**E**	**G**	**I**	**F**	**Y**	**S**	**A**	**M**	**D**	**L**	**I**	**K**	E	K	T	G	Q	E	G	Y	D
I	F	K	Q	A	L	E	N	I	K	P	Q	I	E	V	R	S	R	R	I	G	G	A	T	Y	Q	V	P	V	E
V	K	A	D	R	Q	Q	T	L	A	I	R	W	L	T	T	Y	T	R	A	R	K	E	Y	G	M	I	E	K	L
A	A	E	L	I	A	A	A	N	N	E	G	A	T	I	K	K	K	E	D	T	Y	K	M	A	E	A	N	R	A
F	A	H	Y	R	V																								
1				5					10					15					20					25					30

108 2.51% 95.7% >sp|Q8RHT8|AMPA_FUSNN Probable cytosol aminopeptidase OS=Fusobacterium nucleatum subsp. nucleatum (strain ATCC 25586 / CIP 101130 / JCM 8532 / LMG 13131) OX=190304 GN=pepA PE=3 SV=1

M	S	F	Q	C	V	K	K	Y	E	D	S	Y	D	K	Y	V	L	A	A	T	S	E	K	V	V	L	P	D	Y
L	D	K	E	S	K	K	I	A	E	T	I	I	K	K	N	K	F	T	A	K	A	S	E	K	I	S	M	T	L
V	N	K	K	K	V	I	E	F	I	I	I	G	L	G	E	K	K	K	L	D	A	K	N	T	R	Q	Y	L	F
D	G	L	K	N	I	I	G	K	V	L	F	S	F	D	N	K	D	L	D	N	I	D	I	L	A	E	V	V	E
H	I	N	Y	K	F	D	K	Y	F	S	K	K	K	E	E	F	L	E	V	S	Y	L	T	D	K	K	V	P	K
L	I	E	G	Y	E	L	A	K	I	S	N	I	V	K	D	L	V	N	E	Q	A	E	V	L	N	P	K	E	L
A	D	R	A	T	K	L	G	K	K	F	G	F	D	V	E	I	L	D	E	K	K	A	Q	K	L	G	M	N	A
Y	L	S	V	A	R	A	A	H	H	R	P	Y	V	I	V	M	R	Y	K	G	N	A	K	S	K	Y	T	F	G
L	V	G	K	G	L	T	Y	D	T	G	G	L	S	L	K	P	T	D	S	M	L	T	M	R	C	D	M	G	G
A	A	T	M	I	G	A	M	C	S	V	A	K	M	K	L	K	K	N	V	T	C	V	V	A	A	C	E	N	S
I	G	P	N	A	Y	R	P	G	D	I	L	T	A	M	N	G	K	T	I	E	V	T	N	T	D	A	E	G	R
**L**	**T**	**L**	**A**	**D**	**A**	**L**	**T**	**Y**	**I**	**V**	**R**	K	E	K	V	N	E	V	I	D	A	A	T	L	T	G	A	I	M
V	A	L	G	E	D	V	T	G	V	F	T	N	D	E	K	M	A	R	K	V	I	D	A	S	E	N	W	N	E
Y	F	W	Q	M	P	M	F	D	L	Y	K	K	N	L	K	S	S	Y	A	D	M	Q	N	T	G	V	R	W	G
G	S	T	N	A	A	K	F	L	E	E	F	I	D	D	T	K	W	V	H	L	D	I	A	G	T	A	W	A	S
G	A	N	P	Y	Y	S	Q	K	G	A	T	G	Q	V	F	R	T	V	Y	S	Y	I	K	D	N	K	N		
1				5					10					15					20					25					30

109 4.51% 96.0% >tr|R9RBA8|R9RBA8_FUSNU Sodium/glutamate symporter OS=Fusobacterium nucleatum subsp. animalis 4_8 OX=469607 GN=HMPREF0409_00791 PE=3 SV=1

M	F	E	Y	Q	L	N	M	A	E	T	V	G	F	A	I	I	L	L	L	L	G	R	W	I	K	K	K	V	N
F	F	E	K	F	F	I	P	A	P	V	I	G	G	T	L	F	S	I	I	L	L	I	G	H	Q	T	E	T	F
T	F	T	F	N	D	D	I	K	N	L	L	M	I	A	F	F	T	T	V	G	F	S	A	S	L	K	I	L	K
K	G	G	V	G	V	A	L	F	L	L	A	A	V	I	L	V	I	I	Q	D	I	V	G	P	V	L	A	K	A
L	G	I	N	P	L	L	G	L	A	A	G	S	I	P	L	T	G	G	H	G	T	S	G	A	F	G	P	Y	L
E	D	L	G	A	T	G	A	T	V	V	A	V	A	S	A	T	Y	G	L	I	A	G	C	L	I	G	G	P	I
G	R	R	L	M	I	K	N	N	L	K	P	T	E	N	K	**A**	**G**	**V**	**D**	**D**	**S**	**I**	**L**	**G**	**S**	**T**	**T**	**E**	**V**
**T**	**E**	**E**	**R**	L	F	S	A	V	V	Y	I	G	I	A	M	G	I	G	A	T	I	T	L	I	L	G	N	H	G
I	K	F	P	A	Y	L	M	G	M	V	V	A	A	I	I	R	N	I	L	D	F	N	Q	K	Q	L	P	F	T
E	I	G	I	V	G	N	I	S	L	S	L	F	L	S	M	A	L	M	S	M	K	L	W	Q	L	I	D	L	A
V	P	L	I	V	I	L	L	V	Q	T	V	V	M	G	L	F	A	Y	F	V	T	F	N	I	M	G	R	D	Y
D	A	A	V	M	S	T	G	H	C	G	F	G	L	G	A	T	P	N	A	M	A	N	M	E	T	F	T	T	A
N	G	P	S	V	K	A	F	F	I	I	P	I	V	G	S	L	F	I	D	F	I	N	A	G	V	I	Q	A	F
A	T	W	I	V	K	N	F	M																					
1				5					10					15					20					25					30

110 3.37% 94.5% >tr|Q8RGG4|Q8RGG4_FUSNN Aminotransferase OS=Fusobacterium nucleatum subsp. nucleatum (strain ATCC 25586 / CIP 101130 / JCM 8532 / LMG 13131) OX=190304 GN=FN0334 PE=3 SV=1

M	L	A	K	R	Y	T	G	K	K	L	V	D	N	I	F	A	T	S	K	K	A	K	Q	A	I	V	K	F	G
K	E	N	V	I	N	A	T	I	G	S	L	Y	N	E	D	E	K	L	A	V	Y	D	V	V	E	S	V	Y	R
N	L	P	P	E	D	L	Y	A	Y	A	T	N	V	I	G	E	D	D	Y	L	E	E	V	I	K	A	V	F	F
D	D	Y	K	E	A	L	K	E	L	H	I	A	S	I	A	T	T	G	G	T	G	A	I	S	N	T	V	K	N
Y	M	D	T	G	D	K	V	L	L	P	N	W	M	W	G	T	Y	K	N	I	V	I	E	N	G	G	K	I	E
T	Y	Q	L	F	N	E	N	G	D	F	N	F	E	D	F	K	N	K	V	L	E	L	A	K	I	Q	K	N	V
V	L	I	L	N	E	P	S	H	N	P	T	G	F	R	**M**	**T**	**Y**	**E**	**E**	**W**	**V**	**N**	**L**	**L**	**D**	**F**	**F**	**K**	S
I	K	D	T	N	I	I	V	I	R	D	V	A	Y	F	E	Y	D	D	R	G	E	E	E	T	K	K	L	R	K
L	L	I	G	M	P	K	N	I	L	F	M	Y	A	F	S	L	S	K	S	L	S	I	Y	G	M	R	I	G	A
Q	I	A	V	S	S	D	E	E	V	I	Q	E	F	K	D	A	L	P	F	S	C	R	T	T	W	S	N	I	P
K	G	G	M	K	L	F	A	T	I	M	K	N	P	E	L	K	A	N	F	L	K	E	K	Q	G	Y	M	N	L
L	N	E	R	A	N	I	F	L	K	E	A	K	E	E	N	L	K	V	L	P	Y	K	S	G	F	F	V	T	V
P	V	G	E	T	V	D	K	I	I	E	D	L	E	S	K	N	I	F	V	I	K	F	D	T	G	I	R	I	G
L	C	S	V	P	K	R	K	I	K	G	L	A	K	K	I	K	D	S	I	N	K	F	K	N					
1				5					10					15					20					25					30

111 4.67% 96.0% >tr|Q8RFS0|Q8RFS0_FUSNN DNA polymerase III, beta chain OS=Fusobacterium nucleatum subsp. nucleatum (strain ATCC 25586 / CIP 101130 / JCM 8532 / LMG 13131) OX=190304 GN=FN0617 PE=3 SV=1

M	K	F	S	I	N	R	Q	K	T	I	E	I	I	G	E	Y	S	N	I	L	K	D	N	P	V	K	P	S	L
A	G	L	F	I	Q	A	K	N	N	Q	V	V	F	K	G	A	N	T	E	I	E	L	I	R	Y	A	N	C	E
I	E	S	E	G	Q	V	L	I	K	P	A	L	L	L	E	Y	I	K	L	L	E	G	E	N	I	N	F	E	K
K	D	G	Y	L	I	V	N	N	A	E	F	S	I	L	D	D	N	T	Y	P	E	L	T	E	I	I	P	I	V
I	A	S	E	N	T	V	K	F	T	M	S	L	E	K	V	K	**F**	**L**	**T**	**N**	**S**	**S**	**A**	**S**	**T**	**D**	**T**	**L**	**F**
**N**	**S**	**I**	**K**	M	I	F	K	D	N	V	L	E	L	V	S	T	D	S	F	R	L	I	Y	M	K	K	E	L	N
N	M	I	N	K	D	V	L	V	P	G	D	S	I	A	V	L	Y	K	I	F	K	D	L	D	E	E	F	S	L
A	A	S	D	D	K	L	I	V	T	W	K	D	A	Y	F	T	C	K	L	L	S	L	S	F	P	D	F	R	P
L	I	N	N	T	N	H	D	K	R	F	E	F	N	R	D	E	L	N	L	A	L	K	K	V	I	S	V	T	K
N	S	S	D	S	K	N	V	A	T	F	N	F	K	G	N	Q	L	L	I	S	G	V	S	A	N	A	K	I	N
Q	K	V	N	M	I	K	T	G	E	D	L	K	L	G	M	N	C	K	Y	I	K	D	F	I	D	N	V	D	K
N	I	I	I	D	A	T	N	S	S	S	M	L	R	F	M	E	E	G	N	E	N	Y	I	Y	L	I	M	P	V
N	I	R	V																										
1				5					10					15					20					25					30

112 4.46% 86.3% >tr|Q8RG28|Q8RG28_FUSNN Alanine racemase OS=Fusobacterium nucleatum subsp. nucleatum (strain ATCC 25586 / CIP 101130 / JCM 8532 / LMG 13131) OX=190304 GN=FN0491 PE=3 SV=1

M	N	T	S	F	Y	V	S	L	D	K	N	A	L	Y	H	N	I	E	Y	L	R	E	Y	K	Q	K	E	L	L
P	V	I	K	A	N	A	Y	G	H	N	Y	L	L	I	A	K	A	L	Y	D	F	N	I	K	T	W	A	A	A
R	F	S	E	A	I	T	I	T	E	Y	M	M	N	N	F	S	I	N	D	F	K	I	L	V	F	E	S	L	D
D	D	Y	S	F	L	E	K	Y	P	Q	I	C	P	T	I	N	S	I	K	D	L	K	N	A	L	A	N	N	I
P	I	D	R	L	S	L	K	I	D	F	G	F	G	R	N	G	I	K	T	E	E	V	D	E	L	K	N	L	I
K	F	N	S	L	K	F	L	G	I	F	S	H	L	F	S	S	T	Y	T	D	G	L	E	V	I	R	K	F	T
E	V	V	N	K	L	G	K	D	N	F	E	M	V	H	L	Q	N	A	A	G	I	Y	N	Y	N	V	E	V	V
T	H	I	R	T	G	M	L	T	Y	G	L	Q	E	A	G	F	Y	D	H	D	L	K	P	V	F	T	G	L	I
G	Y	V	D	S	V	R	Y	V	N	E	L	D	Y	V	A	Y	E	D	L	T	S	I	S	P	K	T	K	K	I
A	K	I	K	I	G	Y	G	D	G	F	P	K	A	N	N	K	T	T	C	L	I	K	K	K	E	Y	V	I	S
Q	V	T	M	D	N	T	F	I	E	V	D	D	R	V	N	V	G	D	K	V	H	L	Y	H	R	P	N	E	M
K	T	R	**T**	**G**	**L**	**S**	**M**	**L**	**E**	**A**	**L**	**I**	**A**	**L**	**S**	**P**	**L**	**R**	V	K	R	I	F	E	G	E	E	N	
1				5					10					15					20					25					30

113 4.47% 87.6% >sp|Q8RFZ9|RLMN_FUSNN Probable dual-specificity RNA methyltransferase RlmN OS=Fusobacterium nucleatum subsp. nucleatum (strain ATCC 25586 / CIP 101130 / JCM 8532 / LMG 13131) OX=190304 GN=rlmN PE=3 SV=1

M	K	N	E	K	I	N	I	L	N	L	T	Q	E	E	L	T	E	L	L	V	S	L	G	L	K	K	F	Y	G
K	E	V	F	I	W	L	H	K	K	I	T	R	S	F	D	E	M	T	N	L	S	L	K	D	R	E	I	L	K

				E	K	T	Y	I	P	F	F	N	L	L	K	Y	Q	V	S	K	I	D	K	T	E	K	F	L	F	E	L	E	D
				G	G	T	I	E	T	V	L	L	R	H	K	D	S	K	N	K	E	I	R	N	T	L	C	V	S	S	Q	V	G
				C	P	V	K	C	S	F	C	A	T	G	Q	S	G	Y	M	R	N	L	S	V	S	E	I	L	N	Q	I	Y	T
				V	E	R	R	L	R	K	K	G	E	N	L	N	N	L	V	F	M	G	M	G	E	P	L	L	N	I	D	N	L
				S	K	A	L	S	I	I	S	N	E	N	G	I	N	I	S	K	R	K	I	T	I	S	T	S	G	V	V	S	G
				I	E	K	I	L	L	D	K	I	P	I	E	L	A	I	S	L	H	S	A	I	N	E	K	R	D	K	I	I	P
				I	N	K	N	F	P	L	E	D	L	S	A	V	L	I	E	Y	Q	K	Q	T	K	R	R	I	T	F	E	Y	I
				L	I	D	N	F	N	I	S	E	T	D	A	N	A	L	A	D	F	I	H	Q	F	D	H	V	V	N	L	I	P
				Y	N	E	V	E	G	A	E	H	T	R	P	S	V	K	K	I	N	K	F	Y	I	Y	L	K	N	V	R	K	V
				N	V	T	L	R	Q	E	K	G	S	D	I	D	G	A	C	G	Q	L	R	Q	R	N	K	K	G	D	N		
				1				5					10					15					20					25					30
114	3.06%	93.2%	>tr|Q8RHY2|Q8RHY2_FUSNN Short-chain fatty acids transporter OS=Fusobacterium nucleatum subsp. nucleatum (strain ATCC 25586 / CIP 101130 / JCM 8532 / LMG 13131) OX=190304 GN=FN1858 PE=4 SV=1																														
				M	E	S	V	K	E	R	K	G	I	F	K	R	F	T	S	M	C	V	R	V	M	E	R	W	L	P	D	P	F
				I	F	C	A	L	L	T	F	I	V	F	I	G	A	I	F	L	T	K	A	T	P	L	Q	V	V	G	F	W	A
				D	G	F	W	S	L	L	S	F	S	M	Q	M	A	L	V	L	V	T	G	H	T	L	A	S	S	R	L	F	K
				K	M	L	S	T	F	A	S	G	I	K	G	P	K	Q	A	I	L	I	V	S	I	V	S	G	I	A	C	A	L
				N	W	G	F	G	L	V	I	G	A	L	F	A	K	E	I	A	K	K	V	K	G	V	D	Y	R	L	L	I	A
				S	A	Y	T	G	F	L	V	W	H	G	G	L	S	G	S	I	P	L	Q	L	A	S	G	G	E	G	L	A	K
				Q	T	A	G	V	V	T	E	A	I	P	T	S	Q	T	L	F	S	P	M	N	I	F	I	I	V	G	L	L	I
				I	V	P	L	L	N	M	A	M	F	P	S	K	D	E	I	V	E	V	D	P	K	L	L	V	E	P	E	E	V
				V	M	D	T	S	K	M	T	P	A	E	R	V	E	N	S	S	A	V	S	I	L	L	S	I	M	G	F	V	Y
				I	G	Y	Y	L	K	T	K	G	F	A	L	N	L	N	L	V	N	F	I	F	L	F	L	G	I	L	L	H	G
				T	P	R	R	Y	L	N	A	L	A	E	A	T	K	G	A	G	G	I	L	L	Q	F	P	F	Y	A	G	I	M
				G	I	M	V	G	A	D	A	D	G	M	S	L	A	K	L	M	S	N	F	F	V	S	I	S	T	E	K	T	F
				P	V	F	S	F	I	S	A	G	V	V	N	F	F	V	P	S	G	G	G	Q	W	A	V	Q	A	P	I	V	M
				P	A	G	Q	A	I	G	V	S	A	A	K	S	A	M	A	I	A	W	G	D	A	W	T	N	M	I	Q	P	F
				W	A	L	P	A	L	G	I	A	G	L	G	A	K	D	I	M	G	Y	C	L	I	V	T	V	V	S	G	I	F
				I	C	T	G	F	L	L	F																						
				1				5					10					15					20					25					30
115	2.39%	87.2%	>tr|J8V6J9|J8V6J9_9FUSO Glutaredoxin-like protein OS=Fusobacterium hwasookii ChDC F128 OX=1216362 GN=B437_04006 PE=4 SV=1																														
				M	E	R	I	Y	D	M	I	V	I	G	G	G	P	A	G	L	S	A	G	I	Y	G	G	R	A	K	L	D	V
				L	V	I	E	K	E	N	K	G	G	Q	I	S	L	T	S	E	V	V	N	Y	P	G	I	L	E	I	S	G	S
				E	F	M	T	Q	T	R	K	Q	A	Q	G	F	G	V	N	F	V	Q	E	E	V	V	G	M	D	F	T	Q	K
				I	K	T	I	K	T	N	N	A	E	Y	K	T	L	S	V	V	I	A	T	G	A	A	P	R	K	L	G	F	P
				G	E	K	E	F	T	G	R	G	V	A	Y	C	A	T	C	D	G	E	F	F	T	G	M	D	I	F	V	I	G
				A	G	F	A	A	A	E	E	A	M	F	L	T	K	Y	G	K	S	V	T	I	I	A	R	E	P	D	F	T	C
				A	K	S	I	G	D	K	V	K	A	H	P	K	I	T	V	K	F	N	T	E	L	T	E	L	T	G	D	V	K
				P	T	A	A	K	F	K	N	N	V	T	G	E	V	T	E	Y	K	A	K	V	G	Q	T	F	G	V	F	V	F
				V	G	Y	A	P	S	S	E	I	F	K	G	H	I	E	I	G	Q	G	G	F	I	P	T	N	E	D	L	M	T
				N	V	D	G	V	F	A	V	G	D	I	R	P	K	R	L	R	Q	V	V	T	A	V	A	D	G	A	I	A	A
				T	S	I	E	K	Y	V	H	D	L	R	D	E	L	G	I	Q	K	E	E	K	E	E	E	K	T	T	S	V	A
				T	E	Q	E	H	F	L	D	D	D	L	R	Q	Q	L	V	A	V	V	D	R	F	E	N	P	V	E	I	V	V
				F	K	D	P	N	N	E	E	S	V	N	I	E	N	A	V	K	D	I	A	S	I	S	P	E	K	L	K	F	S
				S	Y	N	E	G	E	N	K	E	L	E	A	K	V	K	I	T	R	T	P	T	I	A	V	L	D	K	D	G	N
				Y	S	G	L	K	Y	S	S	L	P	S	G	H	E	L	N	S	F	I	L	G	L	Y	N	V	A	G	P	G	Q
				K	V	A	A	E	S	L	E	K	I	E	K	I	N	K	P	V	N	I	K	I	G	I	S	L	S	C	T	K	C
				P	K	T	V	Q	A	T	Q	R	I	A	T	L	N	K	N	V	E	M	E	M	I	N	I	F	T	F	Q	D	F
				K	N	R	Y	D	I	M	S	V	P	A	I	I	V	D	D	Q	H	I	Y	F	G	E	K	T	V	E	D	V	L
				E	I	I	N	K																									
				1				5					10					15					20					25					30
116	5.13%	95.7%	>tr|F7L086|F7L086_FUSNU Iron(III)-binding protein OS=Fusobacterium nucleatum subsp. animalis 11_3_2 OX=457403 GN=HMPREF0401_01257 PE=4 SV=1																														
				M	K	K	K	F	F	W	L	V	L	S	L	M	T	L	F	L	V	A	C	G	G	G	E	K	K	E	E	A	T
				D	S	N	A	N	T	E	I	T	G	K	I	V	I	Y	T	S	M	Y	E	D	I	I	D	N	V	S	E	K	L
				K	K	E	F	P	N	L	E	V	E	F	F	Q	G	G	T	G	T	L	Q	S	K	I	I	A	E	L	Q	A	N
				K	L	G	C	D	M	L	M	V	A	E	P	S	Y	S	L	E	L	K	E	K	G	I	L	H	P	Y	I	S	K
				N	A	E	N	L	A	L	D	Y	D	K	E	G	Y	W	Y	P	V	R	L	L	N	M	V	L	A	Y	N	P	D
				K	Y	K	K	E	D	L	A	L	T	F	E	D	F	A	K	R	E	D	L	K	G	K	I	S	I	P	D	P	L
				K	S	G	T	A	L	A	A	V	S	A	L	S	D	K	Y	G	E	E	Y	F	Q	N	L	A	K	L	K	V	V
				V	E	S	G	S	V	A	V	T	K	L	E	T	G	E	A	A	E	I	M	I	L	E	E	S	I	L	K	K	R
				E	E	E	N	S	T	L	E	V	I	Y	P	E	D	G	I	I	S	I	P	S	T	I	M	T	V	K	E	D	M
				S	A	N	K	N	I	K	A	A	E	A	L	T	D	W	F	L	S	P	A	G	Q	E	A	I	V	E	G	W	M
				H	S	V	L	K	N	P	E	K	A	P	Y	D	A	K	A	T	A	E	I	L	K	S	S	M	P	I	N	W	E
				K	T	Y	K	D	R	E	E	L	R	K	M	F	E	K	F	I	T	K	A	N									
				1				5					10					15					20					25					30
117	3.77%	88.6%	>sp|Q8RED8|LDH_FUSNN L-lactate dehydrogenase OS=Fusobacterium nucleatum subsp. nucleatum (strain ATCC 25586 / CIP 101130 / JCM 8532 / LMG 13131) OX=190304 GN=ldh PE=3 SV=1																														
				M	L	Q	T	R	K	V	G	I	V	G	I	G	H	V	G	S	H	C	A	L	S	M	L	L	Q	G	V	C	D
				E	M	V	L	M	D	I	I	P	E	K	A	K	A	H	A	I	D	C	M	D	T	I	S	F	L	P	H	R	A
				I	I	R	D	G	G	I	Q	E	L	S	K	M	D	V	I	V	I	S	V	G	S	L	T	K	N	E	Q	R	L
				E	E	L	K	G	S	L	E	A	I	K	S	F	V	P	D	V	V	K	A	G	F	N	G	I	F	V	T	I	T
				N	P	V	D	I	V	T	Y	F	V	R	E	L	S	G	F	P	K	N	R	V	I	G	T	G	T	G	L	D	S
				A	R	L	K	R	I	L	S	E	V	T	N	I	D	S	Q	V	I	Q	A	Y	M	L	G	E	H	G	D	T	Q
				I	A	N	F	S	S	A	T	I	Q	G	V	P	F	L	D	Y	M	K	T	H	P	E	Q	F	K	G	V	E	L
				S	V	L	E	K	Q	V	V	R	T	A	W	D	I	I	S	G	K	N	C	T	E	F	G	I	G	C	T	C	S
				N	L	V	K	A	I	F	H	N	E	R	R	V	L	P	C	S	A	Y	L	N	G	E	Y	G	H	S	G	F	Y
				T	G	V	P	A	I	I	G	S	N	G	V	E	E	I	L	E	L	P	L	D	E	R	E	R	K	G	F	E	D
				A	C	A	V	M	K	K	Y	I	E	V	G	K	S	Y	K	I	V												
				1				5					10					15					20					25					30
118	6.38%	91.5%	>tr|A0A0M1VVU6|A0A0M1VVU6_FUSNV Uncharacterized protein OS=Fusobacterium nucleatum subsp. vincentii 4_1_13 OX=469606 GN=FSCG_01469 PE=4 SV=1																														
				M	D	E	K	F	W	E	K	I	N	T	Y	G	E	N	G	E	F	D	K	V	V	K	E	I	K	K	L	P	E
				D	K	L	D	I	E	I	I	N	V	L	G	R	S	Y	M	N	L	G	D	Y	E	N	A	L	D	T	Y	L	S
				Y	I	G	K	D	K	E	D	V	T	N	A	D	I	W	L	Y	S	E	C	G	W	L	C	N	E	V	G	D	Y
				E	Q	G	L	K	Y	L	Q	E	S	E	K	L	G	R	D	D	E	W	L	N	T	E	M	G	Q	C	L	G	R
				L	E	R	A	E	E	G	I	V	R	L	K	K	S	L	K	L	I	E	T	E	A	P	E	N	I	N	E	K	I
				F	I	N	S	E	I	G	Y	L	Y	G	F	L	E	N	S	E	E	A	L	K	Y	F	Y	I	A	K	D	L	G
				R	N	D	D	W	I	Y	M	H	L	W	F	N	L	E	R	S	K	G	K	E	E	A	L	K	Y	F	E	N	E
				V	K	A	N	D	K	N	A	V	I	W	A	S	L	G	Q	I	Y	M	N	F	F	E	N	Y	E	G	S	E	K
				A	F	K	K	A	F	G	L	S	G	D	G	L	Y	L	Y	N	R	G	I	A	L	R	M	F	G	R	Y	K	E
				A	V	E	V	L	L	Q	S	R	K	I	S	V	Q	E	G	D	V	T	D	G	E	D	L	D	L	V	R	C	Y
				I	A	L	K	D	K	E	N	A	E	K	Y	L	E	Y	A	K	E	G	I	E	N	V	P	E	E	H	V	D	E
				F	E	N	T	L	K	E	L	E	D	L	I	A	K	I															
				1				5					10					15					20					25					30
119	3.31%	90.2%	>tr|A0A0X3Y1L1|A0A0X3Y1L1_FUSNC Uncharacterized protein OS=Fusobacterium nucleatum subsp. nucleatum OX=76856 GN=RO03_04420 PE=4 SV=1																														
				M	K	K	L	L	L	L	T	A	I	L	S	S	G	L	Y	A	A	S	I	D	H	I	Q	T	Y	S	P	D	Y
				L	A	N	Q	A	Q	T	G	M	I	N	G	V	S	P	Y	Y	N	P	A	A	L	G	R	L	E	K	G	K	Y
				L	H	A	G	F	Q	F	A	H	G	H	E	K	M	S	Y	K	G	K	E	H	K	A	N	L	N	Q	A	I	P
				N	I	A	L	T	F	V	D	D	K	G	A	T	F	F	N	F	G	G	L	A	G	G	G	K	L	R	Y	D	G
				V	S	G	V	D	V	L	T	D	L	A	Q	F	K	P	L	G	I	Y	D	K	G	S	S	L	T	G	S	N	K
				Y	E	Q	I	T	L	G	R	A	F	N	I	D	D	K	L	S	F	S	V	A	A	R	V	V	H	G	T	R	R
				L	N	G	N	L	N	I	G	A	N	P	T	A	Q	Y	K	Q	E	K	A	Q	Q	V	A	Q	E	V	S	K	A
				V	D	A	A	T	Q	G	K	G	L	S	A	A	Q	I	A	A	I	K	T	K	K	T	N	E	A	L	A	K	L
				Q	Q	R	V	N	D	L	S	Q	N	G	L	S	G	D	I	D	S	K	R	E	A	W	G	Y	G	F	Q	L	G
				V	N	Y	K	V	N	D	K	L	N	L	A	A	R	Y	D	S	R	I	K	M	N	F	K	A	K	G	H	E	H
				Q	L	E	T	T	D	I	L	K	Q	T	I	G	L	S	T	F	Y	P	Q	Y	T	I	N	S	K	I	R	R	D
				L	P	A	I	L	S	V	G	A	S	Y	K	V	A	D	N	Y	L	V	S	T	T	A	N	Y	Y	F	N	H	Q
				A	K	M	D	R	V	T	T	F	G	E	H	E	H	G	R	D	Y	K	N	G	W	E	I	A	V	G	N	E	Y
				K	L	N	D	K	F	T	L	I	G	S	L	N	Y	A	N	T	G	A	K	T	A	S	Y	N	D	T	E	Y	A
				L	N	S	F	T	L	G	G	G	I	R	Y	Q	Y	D	E	S	L	A	I	T	A	S	V	A	H	F	I	Y	Q
				G	A	E	G	N	F	K	E	K	Y	G	V	T	E	N	Q	K	Y	K	K	E	I	T	A	V	G	L	S	V	T
				K	K	F																											
				1				5					10					15					20					25					30
120	3.85%	91.6%	>sp|Q8RG52|SYK_FUSNN Lysine--tRNA ligase OS=Fusobacterium nucleatum subsp. nucleatum (strain ATCC 25586 / CIP 101130 / JCM 8532 / LMG 13131) OX=190304 GN=lysS PE=3 SV=1																														
				M	E	K	Y	F	D	R	L	E	K	E	P	L	I	A	E	R	W	K	K	I	E	E	L	E	S	Y	G	I	K
				P	F	G	K	K	Y	D	K	Q	I	M	I	G	D	I	L	K	H	K	P	E	E	N	L	K	F	K	T	A	G
				R	I	M	S	L	R	G	K	G	K	V	Y	F	A	H	I	E	D	Q	S	G	K	I	Q	I	Y	I	K	K	D
				E	L	G	E	E	Q	F	D	H	I	V	K	M	L	N	V	G	D	I	I	G	L	E	G	E	L	F	I	T	H
				T	E	E	L	T	L	R	V	K	S	I	A	L	L	T	K	N	V	R	S	L	P	E	K	Y	H	G	L	T	D
				V	E	I	R	Y	R	K	R	Y	V	D	L	I	M	N	P	E	V	R	E	T	F	I	K	R	T	K	I	I	K
				A	I	R	K	Y	L	D	D	R	G	F	L	E	V	E	T	P	I	M	H	P	I	L	G	G	A	A	A	K	P
				F	I	T	H	H	N	T	L	N	I	D	L	F	L	R	I	A	P	E	L	Y	L	K	K	L	I	V	G	G	F
				E	R	V	Y	D	L	N	R	N	F	R	N	E	G	I	S	T	R	H	N	P	E	F	T	M	V	E	L	Y	Q
				A	H	A	D	F	N	D	M	M	D	L	C	E	G	I	I	S	S	V	C	Q	E	I	N	G	T	T	D	I	E
				Y	D	G	V	Q	L	S	L	K	N	F	N	R	V	H	M	V	D	M	I	K	E	V	T	G	V	D	F	W	K
				E	M	T	F	E	E	A	K	K	L	A	K	E	H	H	V	E	V	A	D	H	M	N	S	V	G	H	I	I	N
				E	F	F	E	Q	K	C	E	E	K	V	I	Q	P	T	F	V	Y	G	H	P	V	E	I	S	P	L	A	K	R
				N	E	D	N	P	N	F	T	D	R	F	E	L	F	I	N	K	R	E	Y	A	N	A	F	T	E	L	N	D	P
				A	D	Q	R	G	R	F	E	A	Q	V	E	E	A	M	R	G	N	E	E	A	T	P	E	I	D	E	S	F	V
				E	A	L	E	Y	G	L	P	P	T	G	G	M	G	I	G	I	D	R	L	V	M	L	L	T	G	A	P	S	I
				R	D	V	I	L	F	P	Q	M	K	P	R	D																	
				1				5					10					15					20					25					30
121	2.54%	90.6%	>tr|D5RD76|D5RD76_FUSN2 AbgT transporter family OS=Fusobacterium nucleatum subsp. nucleatum (strain ATCC 23726 / VPI 4351) OX=525283 GN=ydaH PE=4 SV=1																														
				M	E	K	E	K	K	K	G	I	Q	R	F	L	D	F	V	E	R	G	G	N	K	L	P	H	P	L	T	L	F
				W	L	F	C	L	V	I	A	I	I	S	A	I	A	A	A	S	G	T	S	V	T	Y	E	A	F	N	R	K	E
				G	I	I	Q	E	M	T	L	S	I	K	S	L	L	N	A	E	G	I	R	Y	I	F	S	S	M	V	K	N	F

			TGFAPLGTVLVALIGIGVAEGSGLMGATMK
			KVVTATPKRLLTSIVVLAGVMSNIASDAGY
			VVLIPLGAVIFLSFGRHPIAGLAAAFAGVS
			GGFSANLLLSTTDPLLSGITTEAAKLLNPD
			YFVNPASNYYFMCASTILITVMGTFITEKI
			IEPRLGEYKGEVLVDHNELTAQEKKALRCA
			GVSVIIFCIVMGILTIPANAILRVDGTLKQ
			WTHDGLVPALMMFFLIPGIVYGKVAGTIKN
			DKDVAKMMGSSLATMGGYLALAFAASQFVA
			FFSYTNLGTYVAVKGADFLQSIGLTGLPLI
			IIFVLVAAFINLFMGSASAKWAIMAPIFVP
			MLMRLGYTPEFTQLAYRIGDSSTNIITPLM
			TYFAMIVAFMQKYDKESGMGTLISVMLPYS
			VAFLIGWTIFLMIWFITGLPIGVEGAIHLA
			GM
			1 5 10 15 20 25 30
122	3.50%	92.2%	>tr|I3PCU1|I3PCU1_FUSNC DNA topoisomerase (ATP-hydrolyzing) (Fragment) OS=Fusobacterium nucleatum subsp. nucleatum OX=76856 GN=gyrB PE=4 SV=1
			DEALAGYCDKIEVKILPENIIEVMDNGRGI
			PTDIHPKYGKSAMEIVLTILHAGGKFENDN
			YKVSGGLHGVGVSVVNALSEWLEVEVRKNG
			VVHYQKYHRGKPEEDVKVIGSCDESEHGTI
			VRFKADGEIFETLIYNYFTLSNRLKELAYL
			NKGLTIILSDLRKEEKKEETYKFDGGILDF
			LNEIVKEDTTIIEKPFYVSSEQDNVGVDVT
			FTYTTSQNEVIYSFVNNINTHEGGTHVQGF
			RTALTKVINDVGKAQGLLKDKDGKLMGNDI
			REGVVGIVSTKIPQPQFEGQTKGKLGNSEV
			SGIVNTIVSSSLKIFLEDNPNITKIIIEKI
			LNSKKAREAAQKARELVLRKSVLEVGSLPG
			KLADCTSKKAEECEIFIVEGDSAGGSAKQG
			RDRYNQAILPLRGKIINVEKAGLHKSLESS
			EIRAMVTAFGTSIGETFDIAKLRYGKIILM
			TDADVDGAHIRTLILTFLYRYMKDLITEGN
			IYIACPPLYKVSSGKQIIYAYNDLELKNIL
			GQMNQDNKKYTIQRYKGLGEMNPEQLWETT
			MNP
			1 5 10 15 20 25 30
123	2.77%	92.6%	>tr|D5RE27|D5RE27_FUSN2 Flavocytochrome c OS=Fusobacterium nucleatum subsp. nucleatum (strain ATCC 23726 / VPI 4351) OX=525283 GN=HMPREF0397_1462 PE=4 SV=1
			MEETMRKNFFGKLFGLTLLMFTLLFSGASA
			EVYEGTGLGYDKNGIILDVEITNNKIVDIK
			VKRAKESDFATPAIQEIAKKVIATQSLDVD
			GISGASLTSEGTKEAIEEAVSKSGVTLTAV
			AVQNTKAVELPKEADVVVIGAGGAGLTSAI
			AAHEKGAKVILIEKTELLGGNTNYATAGLN
			AAGTKIQEKLGEKDSPELFYEDTMKGGKNK
			NNKELVKVLANNSSAIVDWLIERGADLSEL
			TSTGGQSAKRTHRPTGGSAVGPNIISALSK
			TAENEKIDIRKGTKAIALVKGKNRIVGVKV
			KEADGKEYTIKAKAVIVATGGFGANAKMVE
			KYNPKLKGFGSTNSPAIVGDGIVMVEKVGG
			ALVDMNEIQTHPTVVYKKTNMITEAVRGEG
			AILVNKDGKRFIDELETRDVVSKAILSQNG
			KSAFLIFDEGIRTKLKAADGYVKKGFAVEG
			TLEEIAAKIGTDAKTLEATLNKYNEAVKNK
			VDSEFNKKNLPKELTGTKYYAIEISPAVHH
			TMGGVRINTNAEVLGKNGRPIKGLYAAGEV
			TGGIHGANRIGGNAVADITIFGKIAGENAA
			TYSKSVK
			1 5 10 15 20 25 30
124	2.57%	93.1%	>tr|R9RCR9|R9RCR9_FUSNU Uncharacterized protein OS=Fusobacterium nucleatum subsp. animalis 4_8 OX=469607 GN=HMPREF0409_00160 PE=4 SV=1
			MEINKRIEKARKVMEKYKVDAYIITSSDYH
			QSEYIDDYFKGREYLSGFTGSAGVLVIFKD
			EACLWTDGRYHIQAENQLKGSEIKLFKQGN
			LGVPTYKEYIVSKLAENSKIGIDAKILLSS
			DINEILSKKKYKIIDFDLLAEVWDKRKALP
			NEKIFILEDKYTGKAYKEKVKEIRKVLKEK
			GADYNIISSLDDIAWIYNFRGDDVQHNPVA
			LSFTVISEKKASLYINKNKLNEEAKKYFKD
			NKVEVKEYFEFFEDIKKLKGNILVDFNKIS
			YAIYEAITKNNLINSMNPSTYLKAHKNETE
			IANTKDIHIQDGVAMVKFMYWLKNNYKKEN
			ITEFSAEESINSLREKIEGYIDLSFSTISA
			FGKNAAMMHYSAPEKKSAKIEDGVYLLDSG
			GTYLKGTTDITRTFFLGKVGKQEKIDNTLV
			LKGMLALSRAKFLFGATGTNLDILARQFLW
			NVGIDYKCGTGHGVGHILNVHEGPHGIRFQ
			YNPQRLEVGMIVTNEPGAYIEGSHGIRIEN
			ELLVKEFCETEHGKFLEFETITYAPIDLDG
			IVKTLLTKEEKQQLNEYHLEVYEKLSPYLN
			KKEKEFLKEYTKSI
			1 5 10 15 20 25 30
125	7.63%	89.1%	>tr|D5RDX3|D5RDX3_FUSN2 Cell shape determining protein, MreB/Mrl family OS=Fusobacterium nucleatum subsp. nucleatum (strain ATCC 23726 / VPI 4351) OX=525283 GN=ppx PE=4 SV=1
			MMRCKVMKKFMGKLLGIFSDDLGIDLGTSN
			TLICMKNKGIILREPSVVAISTKTKEIFEV
			GEKAKHMIGRTPSTYETIRPLRNGVIADYE
			VTEKMLRSFYKRIKSGTLLNKPRVIICVPA
			GITQVEKRAVMEVTREAGAREAYLIEEPMA
			AAIGVGINIFEPEGSMVVDIGGGTSELAVV
			SLGGVVKKSSFRVAGDRFDTAIVDYVRQKH
			NLLIGEKSAEDIKIKIGTVSPEEEDMEIEV
			SGKYVLNGLPKDITLTSSELIDTLSTLVQE
			IIEEIRVVFEKTPPELAADIKKRGIYISGG
			GALLRGIDKKIAAGLNLKVTISEDPLNAVI
			NGIGVLLNNFSLYSKVLVSTETEY
			1 5 10 15 20 25 30
**Recombinant BL21 (DE3) 40kDa**	**overage (Percen**	**obability (Percer**	**Sequences and Coverage**
1	70.92%	99.0%	>tr|Q47903|Q47903_FUSNC Porin OS=Fusobacterium nucleatum subsp. nucleatum OX=76856 GN=fomA PE=4 SV=1
			MKKLALVLGSLLVVGSVASAKEVMPAPTPA
			PKKVVEYVEKPVIVYRDREVAPAWRPNGSV
			DVQYRWYGNVENRTPKKEDPASPWLGDNVN
			AGRLQTLTKVNFTEKQTLEIRTRNYHTLMN
			PKDSQAADDQVRVRHFYKFGKLGSSKIDVT
			SRLEYKKNNGDAGRKQAEASVLFDFADYIY
			SNNFFKADKFGFRLGYQHKWAGHNSGVVGQ
			PFNKGTQDNYFINFESEYTLPWGFSAELNA
			YNYYNVHNKKFATYNKGNKKSQFYGEIEAY
			LYQHTPLYKTNNVELSFDFEGGYDPYTWHQ
			YKVVSAKDSNKYEVYMLPTLQVSYKPTDFV
			KLYAAAGAEYRNWAVTAESKAKNWRWQPTA
			WAGMKVTF
			1 5 10 15 20 25 30
2	67.40%	99.0%	>tr|A0A140NAN5|A0A140NAN5_ECOBD Porin Gram-negative type OS=Escherichia coli (strain B / BL21-DE3) OX=469008 GN=ECBD_2666 PE=3 SV=1
			MMKRNILAVIVPALLVAGTANAAEIYNKDG
			NKVDLYGKAVGLHYFSKGNGENSYGGNGDM
			TYARLGFKGETQINSDLTGYGQWEYNFQGN
			NSEGADAQTGNKTRLAFAGLKYADVGSFDY
			GRNYGVVYDALGYTDMLPEFGGDTAYSDDF
			FVGRVGGVATYRNSNFFGLVDGLNFAVQYL
			GKNERDTARRSNGDGVGGSISYEYEGFGIV
			GAYGAADRTNLQEAQPLGNGKKAEQWATGL
			KYDANNIYLAANYGETRNATPITNKFTNTS
			GFANKTQDVLLVAQYQFDFGLRPSIAYTKS
			KAKDVEGIGDVDLVNYFEVGATYYFNKNMS
			TYVDYIINQIDSDNKLGVGSDDTVAVGIVY
			QF
			1 5 10 15 20 25 30
3	66.93%	99.0%	>tr|A0A140N8E1|A0A140N8E1_ECOBD Phosphoglycerate kinase OS=Escherichia coli (strain B / BL21-DE3) OX=469008 GN=pgk PE=3 SV=1
			MSVIKMTDLDLAGKRVFIRADLNVPVKDGK
			VTSDARIRASLPTIELALKQGAKVMVTSHL
			GRPTEGEYNEEFSLLPVVNYLKDKLSNPVR
			LVKDYLDGVDVAEGELVVLENVRFNKGEKK
			DDETLSKKYAALCDVFVMDAFGTAHRAQAS

			T H G I G K F A D V A C A G P L L A A E L D A L G K A L K E
			P A R P M V A I V G G S K V S T K L T V L D S L S K I A D Q
			L I V G G G I A N T F I A A Q G H D V G K S L Y E A D L V D
			E A K R L L T T C N I P V P S D V R V A T E F S E T A P A T
			L K S V N D V K A D E Q I L D I G D A S A Q E L A E I L K N
			A K T I L W N G P V G V F E F P N F R K G T E I V A N A I A
			D S E A F S I A G G G D T L A A I D L F G I A D K I S Y I S
			T G G G A F L E F V E G K V L P A V A M L E E R A K K
			1 5 10 15 20 25 30
4	54.10%	99.0%	>tr|A0A140N2U0|A0A140N2U0_ECOBD DNA-directed RNA polymerase subunit alpha OS=Escherichia coli (strain B / BL21-DE3) OX=469008 GN=rpoA PE=3 SV=1
			M Q G S V T E F L K P R L V D I E Q V S S T H A K V T L E P
			L E R G F G H T L G N A L R R I L L S S M P G C A V T E V E
			I D G V L H E Y S T K E G V Q E D I L E I L L N L K G L A V
			R V Q G K D E V I L T L N K S G I G P V T A A D I T H D G D
			V E I V K P Q H V I C H L T D E N A S I S M R I K V Q R G R
			G Y V P A S T R I H S E E D E R P I G R L L V D A C Y S P V
			E R I A Y N V E A A R V E Q R T D L D K L V I E M E T N G T
			I D P E E A I R R A A T I L A E Q L E A F V D L R D V R Q P
			E V K E E K P E F D P I L L R P V D D L E L T V R S A N C L
			K A E A I H Y I G D L V Q R T E V E L L K T P N L G K K S L
			T E I K D V L A S R G L S L G M R L E N W P P A S I A D E
			1 5 10 15 20 25 30
5	54.55%	99.0%	>tr|A0A140ND68|A0A140ND68_ECOBD Aminotransferase OS=Escherichia coli (strain B / BL21-DE3) OX=469008 GN=ECBD_2667 PE=3 SV=1
			M F E N I T A A P A D P I L G L A D L F R A D E R P G K I N
			L G I G V Y K D E T G K T P V L T S V K K A E Q Y L L E N E
			T T K N Y L G I D G I P E F G R C T Q E L L F G K G S A L I
			N D K R A R T A Q T P G G T G A L R V A A D F L A K N T S V
			K R V W V S N P S W P N H K S V F N S A G L E V R E Y A Y Y
			D A E N H T L D F D A L I N S L N E A Q A G D V V L F H G C
			C H N P T G I D P T L E Q W Q T L A Q L S V E K G W L P L F
			D F A Y Q G F A R G L E E D A E G L R A F A A M H K E L I V
			A S S Y S K N F G L Y N E R V G A C T L V A A D S E T V D R
			A F S Q M K A A I R A N Y S N P P A H G A S V V A T I L S N
			D A L R A I W E Q E L T D M R Q R I Q R M R Q L F V N T L Q
			E K G A N R D F S F I I K Q N G M F S F S G L T K E Q V L R
			L R E E F G V Y A V A S G R V N V A G M T P D N M A P L C E
			A I V A V L
			1 5 10 15 20 25 30
6	43.73%	99.0%	>tr|A0A140N821|A0A140N821_ECOBD Fructose-bisphosphate aldolase OS=Escherichia coli (strain B / BL21-DE3) OX=469008 GN=ECBD_0813 PE=3 SV=1
			M S K I F D F V K P G V I T G D D V Q K V F Q V A K E N N F
			A L P A V N C V G T D S I N A V L E T A A K V K A P V I V Q
			F S N G G A S F I A G K G V K S D V P Q G A A I L G A I S G
			A H H V H Q M A E H Y G V P V I L H T D H C A K K L L P W I
			D G L L D A G E K H F A A T G K P L F S S H M I D L S E E S
			L Q E N I E I C S K Y L E R M S K I G M T L E I E L G C T G
			G E E D G V D N S H M D A S A L Y T Q P E D V D Y A Y T E L
			S K I S P R F T I A A S F G N V H G V Y K P G N V V L T P T
			I L R D S Q E Y V S K K H N L P H N S L N F V F H G G S G S
			T A Q E I K D S V S Y G V V K M N I D T D T Q W A T W E G V
			L N Y Y K A N E A Y L Q G Q L G N P K G E D Q P N K K Y Y D
			P R V W L R A G Q T S M I A R L E K A F Q E L N A I D V L
			1 5 10 15 20 25 30
7	58.66%	99.0%	>tr|A0A140N802|A0A140N802_ECOBD Glycerophosphoryl diester phosphodiesterase OS=Escherichia coli (strain B / BL21-DE3) OX=469008 GN=ECBD_1421 PE=4 SV=1
			M K L T L K N L S M A I M M S T I V M G S S A M A A D S N E
			K I V I A H R G A S G Y L P E H T L P A K A M A Y A Q G A D
			Y L E Q D L V M T K D D N L V V L H D H Y L D R V T D V A D
			R F P D R A R K D G R Y Y A I D F T L D E I K S L K F T E G
			F D I E N G K K V Q T Y P G R F P M G K S D F R V H T F E E
			E I E F V Q G L N H S T G K N I G I Y P E I K A P W F H H Q
			E G K D I A A K T L E V L K K Y G Y T G K D D K V Y L Q C F
			D A D E L K R I K N E L E P K M G M D L N L V Q L I A Y T D
			W N E T Q Q K Q P D G S W V N Y N Y D W M F K P G A M K Q V
			A E Y A D G I G P D Y H M L I E E T S Q P G N I K L T G M V
			Q D A Q Q N K L V V H P Y T V R S D K L P E Y T T D V N Q L
			Y D A L Y N K A G V N G L F T D F P D K A V K F L N K E
			1 5 10 15 20 25 30
8	51.52%	99.0%	>tr|A0A140N6W0|A0A140N6W0_ECOBD Elongation factor Tu OS=Escherichia coli (strain B / BL21-DE3) OX=469008 GN=tuf PE=1 SV=1
			M S K E K F E R T K P H V N V G T I G H V D H G K T T L T A
			A I T T V L A K T Y G G A A R A F D Q I D N A P E E K A R G
			I T I N T S H V E Y D T P T R H Y A H V D C P G H A D Y V K
			N M I T G A A Q M D G A I L V V A A T D G P M P Q T R E H I
			L L G R Q V G V P Y I I V F L N K C D M V D D E E L L E L V
			E M E V R E L L S Q Y D F P G D D T P I V R G S A L K A L E
			G D A E W E A K I L E L A G F L D S Y I P E P E R A I D K P
			F L L P I E D V F S I S G R G T V V T G R V E R G I I K V G
			E E V E I V G I K E T Q K S T C T G V E M F R K L L D E G R
			A G E N V G V L L R G I K R E E I E R G Q V L A K P G T I K
			P H T K F E S E V Y I L S K D E G G R H T P F F K G Y R P Q
			F Y F R T T D V T G T I E L P E G V E M V M P G D N I K M V
			V T L I H P I A M D D G L R F A I R E G G R T V G A G V V A
			K V L G
			1 5 10 15 20 25 30
9	28.90%	99.0%	>tr|A0A140N870|A0A140N870_ECOBD Alcohol dehydrogenase GroES domain protein OS=Escherichia coli (strain B / BL21-DE3) OX=469008 GN=ECBD_1566 PE=3 SV=1
			M K S V V N D T D G I V R V A E S V I P E I K H Q D E V R V
			K I A S S G L C G S D L P R I F K N G A H Y Y P I T L G H E
			F S G Y I D A V G S G V D D L H P G D A V A C V P L L P C F
			T C P E C L K G F Y S Q C A K Y D F I G S R R D G G F A E Y
			I V V K R K N V F A L P T D M P I E D G A F I E P I T V G L
			H A F H L A Q G C E N K N V I I I G A G T I G L L A I Q C A
			V A L G A K S V T A I D I S S E K L A L A K S F G A M Q T F
			N S S E M S A P Q M Q S V L R E L R F N Q L I L E T A G V P
			Q T V E L A V E I A G P H A Q L A L V G T L H Q D L H L T S
			A T F G K I L R K E L T V I G S W M N Y S S P W P G Q E W E
			T A S R L L T E R K L S L E P L I A H R G S F E S F A Q A V
			R D I A R N A M P G K V L L I P
			1 5 10 15 20 25 30
10	35.68%	99.0%	>tr|A0A140N487|A0A140N487_ECOBD 2-amino-3-ketobutyrate coenzyme A ligase OS=Escherichia coli (strain B / BL21-DE3) OX=469008 GN=kbl PE=3 SV=1
			M R G E F Y Q Q L T N D L E T A R A E G L F K E E R I I T S
			A Q Q A D I T V A D G S H V I N F C A N N Y L G L A N H P D
			L I A A A K A G M D S H G F G M A S V R F I C G T Q D S H K
			E L E Q K L A A F L G M E D A I L Y S S C F D A N G G L F E
			T L L G A E D A I I S D A L N H A S I I D G V R L C K A K R
			Y R Y A N N D M Q E L E A R L K E A R E A G A R H V L I A T
			D G V F S M D G V I A N L K G V C D L A D K Y D A L V M V D
			D S H A V G F V G E N G R G S H E Y C D V M G R V D I I T G
			T L G K A L G G A S G G Y T A A R K E V V E W L R Q R S R P
			Y L F S N S L A P A I V A A S I K V L E M V E A G S E L R D
			R L W A N A R Q F R G Q M S A A G F T L A G A D H A I I P V
			M L G D A V V A Q K F A R E L Q K E G I Y V T G F F Y P V V
			P K G Q A R I R T Q M S A A H T P E Q I T R A V E A F T R I
			G K Q L G V I A
			1 5 10 15 20 25 30
11	55.61%	99.0%	>tr|A0A140NFM6|A0A140NFM6_ECOBD Cell division protein FtsZ OS=Escherichia coli (strain B / BL21-DE3) OX=469008 GN=ftsZ PE=3 SV=1
			M F E P M E L T N D A V I K V I G V G G G G G N A V E H M V
			R E R I E G V E F F A V N T D A Q A L R K T A V G Q T I Q I
			G S G I T K G L G A G A N P E V G R N A A D E D R D A L R A
			A L E G A D M V F I A A G M G G G T G T G A A P V V A E V A
			K D L G I L T V A V V T K P F N F E G K K R M A F A E Q G I
			T E L S K H V D S L I T I P N D K L L K V L G R G I S L L D
			A F G A A N D V L K G A V Q G I A E L I T R P G L M N V D F
			A D V R T V M S E M G Y A M M G S G V A S G E D R A E E A A
			E M A I S S P L L E D I D L S G A R G V L V N I T A G F D L
			R L D E F E T V G N T I R A F A S D N A T V V I G T S L D P
			D M N D E L R V T V V A T G I G M D K R P E I T L V T N K Q
			V Q Q P V M D R Y Q Q H G M A P L T Q E Q K P V A K V V N D
			N A P Q T A K E P D Y L D I P A F L R K Q A D
			1 5 10 15 20 25 30
12	45.36%	99.0%	>tr|A0A140NBF4|A0A140NBF4_ECOBD Succinate--CoA ligase [ADP-forming] subunit beta OS=Escherichia coli (strain B / BL21-DE3) OX=469008 GN=sucC PE=3 SV=1
			M N L H E Y Q A K Q L F A R Y G L P A P V G Y A C T T P R E
			A E E A A S K I G A G P W V V K C Q V H A G G R G K A G G V
			K V V N S K E D I R A F A E N W L G K R L V T Y Q T D A N G
			Q P V N Q I L V E A A T D I A K E L Y L G A V V D R S S R R

				V	V	F	M	A	S	T	E	G	G	V	E	I	E	K	V	A	E	E	T	P	H	L	I	H	K	V	A	L	D
				P	L	T	G	P	M	P	Y	Q	G	R	E	L	A	F	K	L	G	L	E	G	K	L	V	Q	Q	F	T	K	I
				F	M	G	L	A	T	I	F	L	E	R	D	L	A	L	I	E	I	N	P	L	V	I	T	K	Q	G	D	L	I
				C	L	D	G	K	L	G	A	D	G	N	A	L	F	R	Q	P	D	L	R	E	M	R	D	Q	S	Q	E	D	P
				R	E	A	Q	A	A	Q	W	E	L	N	Y	V	A	L	D	G	N	I	G	C	M	V	N	G	A	G	L	A	M
				G	T	M	D	I	V	K	L	H	G	G	E	P	A	N	F	L	D	V	G	G	G	A	T	K	E	R	V	T	E
				A	F	K	I	I	L	S	D	D	K	V	K	A	V	L	V	N	I	F	G	G	I	V	R	C	D	L	I	A	D
				G	I	I	G	A	V	A	E	V	G	V	N	V	P	V	V	V	R	L	E	G	N	N	A	E	L	G	A	K	K
				L	A	D	S	G	L	N	I	I	A	A	K	G	L	T	D	A	A	Q	Q	V	V	A	A	V	E	G	K		
				1				5					10					15					20					25					30
13	49.17%	99.0%	>tr|A0A140NDI4|A0A140NDI4_ECOBD Phosphoserine aminotransferase OS=Escherichia coli (strain B / BL21-DE3) OX=469008 GN=serC PE=3 SV=1																														
				M	A	Q	I	F	N	F	S	S	G	P	A	M	L	P	A	E	V	L	K	Q	A	Q	Q	E	L	R	D	W	N
				G	L	G	T	S	V	M	E	V	S	H	R	G	K	E	F	I	Q	V	A	E	E	A	E	K	D	F	R	D	L
				L	N	V	P	S	N	Y	K	V	L	F	C	H	G	G	G	R	G	Q	F	A	A	V	P	L	N	I	L	G	D
				K	T	T	A	D	Y	V	D	A	G	Y	W	A	A	S	A	I	K	E	A	K	K	Y	C	T	P	N	V	F	D
				A	K	V	T	V	D	G	L	R	A	V	K	P	M	R	E	W	Q	L	S	D	N	A	A	Y	M	H	Y	C	P
				N	E	T	I	D	G	I	A	I	D	E	T	P	D	F	G	K	D	V	V	V	A	A	D	F	S	S	T	I	L
				S	R	P	I	D	V	S	R	Y	G	V	I	Y	A	G	A	Q	K	N	I	G	P	A	G	L	T	I	V	I	V
				R	E	D	L	L	G	K	A	N	I	A	C	P	S	I	L	D	Y	S	I	L	N	D	N	G	S	M	F	N	T
				P	P	T	F	A	W	Y	L	S	G	L	V	F	K	W	L	K	A	N	G	G	V	A	E	M	D	K	I	N	Q
				Q	K	A	E	L	L	Y	G	V	I	D	N	S	D	F	Y	R	N	D	V	A	K	A	N	R	S	R	M	N	V
				P	F	Q	L	A	D	S	A	L	D	K	L	F	L	E	E	S	F	A	A	G	L	H	A	L	K	G	H	R	V
				V	G	G	M	R	A	S	I	Y	N	A	M	P	L	E	G	V	K	A	L	T	D	F	M	V	E	F	E	R	R
				H	G																												
				1				5					10					15					20					25					30
14	40.69%	99.0%	>tr|A0A140NBN8|A0A140NBN8_ECOBD Beta-lactamase OS=Escherichia coli (strain B / BL21-DE3) OX=469008 GN=ECBD_3019 PE=3 SV=1																														
				M	N	T	I	F	S	A	R	I	M	K	R	L	A	L	T	T	A	L	C	T	A	F	I	S	A	A	H	A	D
				D	L	N	I	K	T	M	I	P	G	V	P	Q	I	D	A	E	S	Y	I	L	I	D	Y	N	S	G	K	V	L
				A	E	Q	N	A	D	V	R	R	D	P	A	S	L	T	K	M	M	T	S	Y	V	I	G	Q	A	M	K	A	G
				K	F	K	E	T	D	L	V	T	I	G	N	D	A	W	A	T	G	N	P	V	F	K	G	S	S	L	M	F	L
				K	P	G	M	Q	V	P	V	S	Q	L	I	R	G	I	N	L	Q	S	G	N	D	A	C	V	A	M	A	D	F
				A	A	G	S	Q	D	A	F	V	G	L	M	N	S	Y	V	N	A	L	G	L	K	N	T	H	F	Q	T	V	H
				G	L	D	A	D	G	Q	Y	S	S	A	R	D	M	A	L	I	G	Q	A	L	I	R	D	V	P	N	E	Y	S
				I	Y	K	E	K	E	F	T	F	N	G	I	R	Q	L	N	R	N	G	L	L	W	D	N	S	L	N	V	D	G
				I	K	T	G	H	T	D	K	A	G	Y	N	L	V	A	S	A	T	E	G	Q	M	R	L	I	S	A	V	M	G
				G	R	T	F	K	G	R	E	A	E	S	K	K	L	L	T	W	G	F	R	F	F	E	T	V	N	P	L	K	V
				G	K	E	F	A	S	E	P	V	W	F	G	D	S	D	R	A	S	L	G	V	D	K	D	V	Y	L	T	I	P
				R	G	R	M	K	D	L	K	A	S	Y	V	L	N	S	S	E	L	H	A	P	L	Q	K	N	Q	V	V	G	T
				I	N	F	Q	L	D	G	K	T	I	E	Q	R	P	L	V	V	L	Q	E	I	P	E	G	N	F	F	G	K	I
				I	D	Y	I	K	L	M	F	H	H	W	F	G																	
				1				5					10					15					20					25					30
15	49.68%	99.0%	>tr|A0A140N6M4|A0A140N6M4_ECOBD ADP-L-glycero-D-manno-heptose-6-epimerase OS=Escherichia coli (strain B / BL21-DE3) OX=469008 GN=hldD PE=3 SV=1																														
				M	I	I	V	T	G	G	A	G	F	I	G	S	N	I	V	K	A	L	N	D	K	G	I	T	D	I	L	V	V
				D	N	L	K	D	G	T	K	F	V	N	L	V	D	L	N	I	A	D	Y	M	D	K	E	D	F	L	I	Q	I
				M	A	G	E	E	F	G	D	V	E	A	I	F	H	E	G	A	C	S	S	T	T	E	W	D	G	K	Y	M	M
				D	N	N	Y	Q	Y	S	K	E	L	L	H	Y	C	L	E	R	E	I	P	F	L	Y	A	S	S	A	A	T	Y
				G	G	R	T	S	D	F	I	E	S	R	E	Y	E	K	P	L	N	V	Y	G	Y	S	K	F	L	F	D	E	Y
				V	R	Q	I	L	P	E	A	N	S	Q	I	V	G	F	R	Y	F	N	V	Y	G	P	R	E	G	H	K	G	S
				M	A	S	V	A	F	H	L	N	T	Q	L	N	N	G	E	S	P	K	L	F	E	G	S	E	N	F	K	R	D
				F	V	Y	V	G	D	V	A	D	V	N	L	W	F	L	E	N	G	V	S	G	I	F	N	L	G	T	G	R	A
				E	S	F	Q	A	V	A	D	A	T	L	A	Y	H	K	K	G	Q	I	E	Y	I	P	F	P	D	K	L	K	G
				R	Y	Q	A	F	T	Q	A	D	L	T	N	L	R	A	A	G	Y	D	K	P	F	K	T	V	A	E	G	V	T
				E	Y	M	A	W	L	N	R	D	A																				
				1				5					10					15					20					25					30
16	23.04%	99.0%	>tr|A0A140NFV3|A0A140NFV3_ECOBD Chaperone protein DnaK OS=Escherichia coli (strain B / BL21-DE3) OX=469008 GN=dnaK PE=2 SV=1																														
				M	G	K	I	I	G	I	D	L	G	T	T	N	S	C	V	A	I	M	D	G	T	T	P	R	V	L	E	N	A
				E	G	D	R	T	T	P	S	I	I	A	Y	T	Q	D	G	E	T	L	V	G	Q	P	A	K	R	Q	A	V	T
				N	P	Q	N	T	L	F	A	I	K	R	L	I	G	R	R	F	Q	D	E	E	V	Q	R	D	V	S	I	M	P
				F	K	I	I	A	A	D	N	G	D	A	W	V	E	V	K	G	Q	K	M	A	P	P	Q	I	S	A	E	V	L
				K	K	M	K	K	T	A	E	D	Y	L	G	E	P	V	T	E	A	V	I	T	V	P	A	Y	F	N	D	A	Q
				R	Q	A	T	K	D	A	G	R	I	A	G	L	E	V	K	R	I	I	N	E	P	T	A	A	A	L	A	Y	G
				L	D	K	G	T	G	N	R	T	I	A	V	Y	D	L	G	G	G	T	F	D	I	S	I	I	E	I	D	E	V
				D	G	E	K	T	F	E	V	L	A	T	N	G	D	T	H	L	G	G	E	D	F	D	S	R	L	I	N	Y	L
				V	E	E	F	K	K	D	Q	G	I	D	L	R	N	D	P	L	A	M	Q	R	L	K	E	A	A	E	K	A	K
				I	E	L	S	S	A	Q	Q	T	D	V	N	L	P	Y	I	T	A	D	A	T	G	P	K	H	M	N	I	K	V
				T	R	A	K	L	E	S	L	V	E	D	L	V	N	R	S	I	E	P	L	K	V	A	L	Q	D	A	G	L	S
				V	S	D	I	D	D	V	I	L	V	G	G	Q	T	R	M	P	M	V	Q	K	K	V	A	E	F	F	G	K	E
				P	R	K	D	V	N	P	D	E	A	V	A	I	G	A	A	V	Q	G	G	V	L	T	G	D	V	K	D	V	L
				L	L	D	V	T	P	L	S	L	G	I	E	T	M	G	G	V	M	T	T	L	I	A	K	N	T	T	I	P	T
				K	H	S	Q	V	F	S	T	A	E	D	N	Q	S	A	V	T	I	H	V	L	Q	G	E	R	K	R	A	A	D
				N	K	S	L	G	Q	F	N	L	D	G	I	N	P	A	P	R	G	M	P	Q	I	E	V	T	F	D	I	D	A
				D	G	I	L	H	V	S	A	K	D	K	N	S	G	K	E	Q	K	I	T	I	K	A	S	S	G	L	N	E	D
				E	I	Q	K	M	V	R	D	A	E	A	N	A	E	A	D	R	K	F	E	E	L	V	Q	T	R	N	Q	G	D
				H	L	L	H	S	T	R	K	Q	V	E	E	A	G	D	K	L	P	A	D	D	K	T	A	I	E	S	A	L	T
				A	L	E	T	A	L	K	G	E	D	K	A	A	I	E	A	K	M	Q	E	L	A	Q	V	S	Q	K	L	M	E
				I	A	Q	Q	Q	H	A	Q	Q	Q	T	A	G	A	D	A	S	A	N	N	A	K	D	D	D	V	V	D	A	E
				F	E	E	V	K	D	K	K																						
				1				5					10					15					20					25					30
17	37.37%	99.0%	>tr|A0A140N4P3|A0A140N4P3_ECOBD 4-hydroxy-3-methylbut-2-en-1-yl diphosphate synthase (flavodoxin) OS=Escherichia coli (strain B / BL21-DE3) OX=469008 GN=ispG PE=3 SV=1																														
				M	H	N	Q	A	P	I	Q	R	R	K	S	T	R	I	Y	V	G	N	V	P	I	G	D	G	A	P	I	A	V
				Q	S	M	T	N	T	R	T	T	D	V	E	A	T	V	N	Q	I	K	A	L	E	R	V	G	A	D	I	V	R
				V	S	V	P	T	M	D	A	A	E	A	F	K	L	I	K	Q	Q	V	N	V	P	L	V	A	D	I	H	F	D
				Y	R	I	A	L	K	V	A	E	Y	G	V	D	C	L	R	I	N	P	G	N	I	G	N	E	E	R	I	R	M
				V	V	D	C	A	R	D	K	N	I	P	I	R	I	G	V	N	A	G	S	L	E	K	D	L	Q	E	K	Y	G
				E	P	T	P	Q	A	L	L	E	S	A	M	R	H	V	D	H	L	D	R	L	N	F	D	Q	F	K	V	S	V
				K	A	S	D	V	F	L	A	V	E	S	Y	R	L	L	A	K	Q	I	D	Q	P	L	H	L	G	I	T	E	A
				G	G	A	R	S	G	A	V	K	S	A	I	G	L	G	L	L	L	S	E	G	I	G	D	T	L	R	V	S	L
				A	A	D	P	V	E	E	I	K	V	G	F	D	I	L	K	S	L	R	I	R	S	R	G	I	N	F	I	A	C
				P	T	C	S	R	Q	E	F	D	V	I	G	T	V	N	A	L	E	Q	R	L	E	D	I	I	T	P	M	D	V
				S	I	I	G	C	V	V	N	G	P	G	E	A	L	V	S	T	L	G	V	T	G	G	N	K	K	S	G	L	Y
				E	D	G	V	R	K	D	R	L	D	N	N	D	M	I	D	Q	L	E	A	R	I	R	A	K	A	S	Q	L	D
				E	A	R	R	I	D	V	Q	Q	V	E	K																		
				1				5					10					15					20					25					30
18	23.46%	99.0%	>tr|A0A140NB75|A0A140NB75_ECOBD Delta-aminolevulinic acid dehydratase OS=Escherichia coli (strain B / BL21-DE3) OX=469008 GN=ECBD_3292 PE=3 SV=1																														
				M	T	D	L	I	Q	R	P	R	R	L	R	K	S	P	A	L	R	A	M	F	E	E	T	T	L	S	L	N	D
				L	V	L	P	I	F	V	E	E	E	I	D	D	Y	K	A	V	E	A	M	P	G	V	M	R	I	P	E	K	H
				L	A	R	E	I	E	R	I	A	N	A	G	I	R	S	V	M	T	F	G	I	S	H	H	T	D	E	T	G	S
				D	A	W	R	E	D	G	L	V	A	R	M	S	R	I	C	K	Q	T	V	P	E	M	I	V	M	S	D	T	C
				F	C	E	Y	T	S	H	G	H	C	G	V	L	C	E	H	G	V	D	N	D	A	T	L	E	N	L	G	K	Q
				A	V	V	A	A	A	A	G	A	D	F	I	A	P	S	A	A	M	D	G	Q	V	Q	A	I	R	Q	A	L	D
				A	A	G	F	K	D	T	A	I	M	S	Y	S	T	K	F	A	S	S	F	Y	G	P	F	R	E	A	A	G	S
				A	L	K	G	D	R	K	S	Y	Q	M	N	P	M	N	R	R	E	A	I	R	E	S	L	L	D	E	A	Q	G
				A	D	C	L	M	V	K	P	A	G	A	Y	L	D	I	V	R	E	L	R	E	R	T	E	L	P	I	G	A	Y
				Q	V	S	G	E	Y	A	M	I	K	F	A	A	L	A	G	A	I	D	E	E	K	V	V	L	E	S	L	G	S
				I	K	R	A	G	A	D	L	I	F	S	Y	F	A	M	D	L	A	E	K	K	I	L	R						
				1				5					10					15					20					25					30
19	29.09%	99.0%	>tr|A0A140N769|A0A140N769_ECOBD Tartrate dehydrogenase OS=Escherichia coli (strain B / BL21-DE3) OX=469008 GN=ECBD_1842 PE=4 SV=1																														
				M	M	K	T	M	R	I	A	A	I	P	G	D	G	I	G	K	E	V	L	P	E	G	I	R	V	L	Q	A	A
				A	E	R	W	G	F	A	L	S	F	E	Q	M	E	W	A	S	C	E	Y	Y	S	H	H	G	K	M	M	P	D
				D	W	H	E	Q	L	S	R	F	D	A	I	Y	F	G	A	V	G	W	P	D	T	V	P	D	H	I	S	L	W
				G	S	L	L	K	F	R	R	E	F	D	Q	Y	V	N	L	R	P	V	R	L	F	P	G	V	P	C	P	L	A
				G	K	Q	P	G	D	I	D	F	Y	V	V	R	E	N	T	E	G	E	Y	S	S	L	G	G	R	V	N	E	G
				T	E	H	E	V	V	I	Q	E	S	V	F	T	R	R	G	V	D	R	I	L	R	Y	A	F	E	L	A	Q	S
				R	P	R	K	T	L	T	S	A	T	K	S	N	G	L	A	I	S	M	P	Y	W	D	E	R	V	E	A	M	A
				E	N	Y	P	E	I	R	W	D	K	Q	H	I	D	I	L	C	A	R	F	V	M	Q	P	E	R	F	D	V	V
				V	A	S	N	L	F	G	D	I	L	S	D	L	G	P	A	C	I	G	T	I	G	I	A	P	S	A	N	L	N
				P	E	R	T	F	P	S	L	F	E	P	V	H	G	S	A	P	D	I	Y	G	K	N	I	A	N	P	I	A	T
				I	W	A	G	A	M	M	L	D	F	L	G	N	G	D	E	R	F	Q	Q	A	H	N	G	I	L	A	A	I	E
				E	V	I	A	H	G	P	K	T	P	D	M	K	G	N	A	T	T	P	Q	V	A	D	A	I	C	K	I	I	L
				R																													
				1				5					10					15					20					25					30
20	29.58%	99.0%	>tr|A0A140NDH1|A0A140NDH1_ECOBD Glutamate-1-semialdehyde 21-aminomutase OS=Escherichia coli (strain B / BL21-DE3) OX=469008 GN=hemL PE=3 SV=1																														
				M	S	K	S	E	N	L	Y	S	A	A	R	E	L	I	P	G	G	V	N	S	P	V	R	A	F	T	G	V	G
				G	T	P	L	F	I	E	K	A	D	G	A	Y	L	Y	D	V	D	G	K	A	Y	I	D	Y	V	G	S	W	G
				P	M	V	L	G	H	N	H	P	A	I	R	N	A	V	I	E	A	A	E	R	G	L	S	F	G	A	P	T	E
				M	E	V	K	M	A	Q	L	V	T	E	L	V	P	T	M	D	M	V	R	M	V	N	S	G	T	E	A	T	M
				S	A	I	R	L	A	R	G	F	T	G	R	D	K	I	I	K	F	E	G	C	Y	H	G	H	A	D	C	L	L
				V	K	A	G	S	G	A	L	T	L	G	Q	P	N	S	P	G	V	P	A	D	F	A	K	H	T	L	T	C	T
				Y	N	D	L	A	S	V	R	A	A	F	E	Q	Y	P	Q	E	I	A	C	I	I	V	E	P	V	A	G	N	M
				N	C	V	P	P	L	P	E	F	L	P	G	L	R	A	L	C	D	E	F	G	A	L	L	I	I	D	E	V	M
				T	G	F	R	V	A	L	A	G	A	Q	D	Y	Y	G	V	E	P	D	L	T	C	L	G	K	I	I	G	G	G
				M	P	V	G	A	F	G	G	R	R	D	V	M	D	A	L	A	P	T	G	P	V	Y	Q	A	G	T	L	S	G
				N	P	I	A	M	A	A	G	F	A	C	L	N	E	V	A	Q	P	G	V	H	E	T	L	D	E	L	T	S	R

				L	A	E	G	L	L	E	A	A	E	E	A	G	I	P	L	V	V	N	H	V	G	G	M	F	G	I	F	F	T
				D	A	E	S	V	T	C	Y	Q	D	V	M	A	C	D	V	E	R	F	K	R	F	F	H	M	M	L	D	E	G
				V	Y	L	A	P	S	A	F	E	A	G	F	M	S	V	A	H	S	M	E	D	I	N	N	T	I	D	A	A	R
				R	V	F	A	K	L																								
				1				5					10					15					20					25					30
21	29.25%	99.0%	>tr|A0A140NAC4|A0A140NAC4_ECOBD PhoH family protein OS=Escherichia coli (strain B / BL21-DE3) OX=469008 GN=ECBD_2991 PE=4 SV=1																														
				M	F	S	F	R	R	H	T	D	K	R	N	G	L	N	I	D	T	R	E	I	T	L	E	P	A	D	N	A	R
				L	L	S	L	C	G	P	F	D	D	N	I	K	Q	L	E	R	R	L	G	I	E	I	N	R	R	D	N	H	F
				K	L	T	G	R	P	I	C	V	T	A	A	A	D	I	L	R	S	L	Y	V	D	T	A	P	M	R	G	Q	I
				Q	D	I	E	P	E	Q	I	H	L	A	I	K	E	A	R	V	L	E	Q	S	A	E	S	V	P	E	Y	G	K
				A	V	N	I	K	T	K	R	G	V	I	K	P	R	T	P	N	Q	A	Q	Y	I	A	N	I	L	D	H	D	I
				T	F	G	V	G	P	A	G	T	G	K	T	Y	L	A	V	A	A	A	V	D	A	L	E	R	Q	E	I	R	R
				I	L	L	T	R	P	A	V	E	A	G	E	K	L	G	F	L	P	G	D	L	S	Q	K	V	D	P	Y	L	R
				P	L	Y	D	A	L	F	E	M	L	G	F	E	K	V	E	K	L	I	E	R	N	V	I	E	V	A	P	L	A
				Y	M	R	G	R	T	L	N	D	A	F	I	I	L	D	E	S	Q	N	T	T	I	E	Q	M	K	M	F	L	T
				R	I	G	F	N	S	K	A	V	I	T	G	D	V	T	Q	I	D	L	P	R	N	T	K	S	G	L	R	H	A
				I	E	V	L	A	D	V	E	E	I	S	F	N	F	F	H	S	E	D	V	V	R	H	P	V	V	A	R	I	V
				N	A	Y	E	A	W	E	E	A	E	Q	K	R	K	A	A	L	A	A	E	R	K	R	E	E	Q	E	Q	K	
				1				5					10					15					20					25					30
22	24.79%	99.0%	>tr|A0A140NB96|A0A140NB96_ECOBD Transcriptional regulatorLacI family OS=Escherichia coli (strain B / BL21-DE3) OX=469008 GN=ECBD_2908 PE=4 SV=1																														
				M	V	N	V	K	P	V	T	L	Y	D	V	A	E	Y	A	G	V	S	Y	Q	T	V	S	R	V	V	N	Q	A
				S	H	V	S	A	K	T	R	E	K	V	E	A	A	M	A	E	L	N	Y	I	P	N	R	V	A	Q	Q	L	A
				G	K	Q	S	L	L	I	G	V	A	T	S	S	L	A	L	H	A	P	S	Q	I	V	A	A	I	K	S	R	A
				D	Q	L	G	A	S	V	V	V	S	M	V	E	R	S	G	V	E	A	C	K	A	A	V	H	N	L	L	A	Q
				R	V	S	G	L	I	I	N	Y	P	L	D	D	Q	D	A	I	A	V	E	A	A	C	T	N	V	P	A	L	F
				L	D	V	S	D	Q	T	P	I	N	S	I	I	F	S	H	E	D	G	T	R	L	G	V	E	H	L	V	A	L
				G	H	Q	Q	I	A	L	L	A	G	P	L	S	S	V	S	A	R	L	R	L	A	G	W	H	K	Y	L	T	R
				N	Q	I	Q	P	I	A	E	R	E	G	D	W	S	A	M	S	G	F	Q	Q	T	M	Q	M	L	N	E	G	I
				V	P	T	A	M	L	V	A	N	D	Q	M	A	L	G	A	M	R	A	I	T	E	S	G	L	R	V	G	A	D
				I	S	V	V	G	Y	D	D	T	E	D	S	S	C	Y	I	P	P	L	T	T	I	K	Q	D	F	R	L	L	G
				Q	T	S	V	D	R	L	L	Q	L	S	Q	G	Q	A	V	K	G	N	Q	L	L	P	V	S	L	V	K	R	K
				T	T	L	A	P	N	T	Q	T	A	S	P	R	A	L	A	D	S	L	M	Q	L	A	R	Q	V	S	R	L	E
				S	G	Q																											
				1				5					10					15					20					25					30
23	23.67%	99.0%	>tr|A0A140NFZ9|A0A140NFZ9_ECOBD Chaperone protein DnaJ OS=Escherichia coli (strain B / BL21-DE3) OX=469008 GN=dnaJ PE=3 SV=1																														
				M	A	K	Q	D	Y	Y	E	I	L	G	V	S	K	T	A	E	E	H	E	I	K	K	A	Y	K	R	L	A	M
				K	Y	H	P	D	R	N	Q	G	D	K	E	A	E	A	K	F	K	E	I	K	E	A	Y	E	V	L	T	D	S
				Q	K	R	A	A	Y	D	Q	Y	G	H	A	A	F	E	Q	G	G	M	G	G	G	G	F	G	G	G	A	D	F
				S	D	I	F	G	D	V	F	G	D	I	F	G	G	G	R	G	R	Q	R	A	A	R	G	A	D	L	R	Y	N
				M	E	L	T	L	E	E	A	V	R	G	V	T	K	E	I	R	I	P	T	L	E	E	C	D	V	C	H	G	S
				G	A	K	P	G	T	Q	P	Q	T	C	P	T	C	H	G	S	G	Q	V	Q	M	R	Q	G	F	F	A	V	Q
				Q	T	C	P	H	C	Q	G	R	G	T	L	I	K	D	P	C	N	K	C	H	G	H	G	R	V	E	R	S	K
				T	L	S	V	K	I	P	A	G	V	D	T	G	D	R	I	R	L	A	G	E	G	E	A	G	E	H	G	A	P
				A	G	D	L	Y	V	Q	V	Q	V	K	Q	H	P	I	F	E	R	E	G	N	N	L	Y	C	E	V	P	I	N
				F	A	M	A	A	L	G	G	E	I	E	V	P	T	L	D	G	R	V	K	L	K	V	P	G	E	T	Q	T	G
				K	L	F	R	M	R	G	K	G	V	K	S	V	R	G	G	A	Q	G	D	L	L	C	R	V	V	V	E	T	P
				V	G	L	N	E	K	Q	K	Q	L	L	Q	E	L	Q	E	S	F	G	G	P	T	G	E	H	N	S	P	R	S
				K	S	F	F	D	G	V	K	K	F	F	D	D	L	T	R														
				1				5					10					15					20					25					30
24	22.15%	99.0%	>tr|A0A140NBS3|A0A140NBS3_ECOBD Dihydrolipoyl dehydrogenase OS=Escherichia coli (strain B / BL21-DE3) OX=469008 GN=ECBD_3503 PE=3 SV=1																														
				M	S	T	E	I	K	T	Q	V	V	V	L	G	A	G	P	A	G	Y	S	A	A	F	R	C	A	D	L	G	L
				E	T	V	I	V	E	R	Y	N	T	L	G	G	V	C	L	N	V	G	C	I	P	S	K	A	L	L	H	V	A
				K	V	I	E	E	A	K	A	L	A	E	H	G	I	V	F	G	E	P	K	T	D	I	D	K	I	R	T	W	K
				E	K	V	I	N	Q	L	T	G	G	L	A	G	M	A	K	G	R	K	V	K	V	V	N	G	L	G	K	F	T
				G	A	N	T	L	E	V	E	G	E	N	G	K	T	V	I	N	F	D	N	A	I	I	A	A	G	S	R	P	I
				Q	L	P	F	I	P	H	E	D	P	R	I	W	D	S	T	D	A	L	E	L	K	E	V	P	E	R	L	L	V
				M	G	G	G	I	I	G	L	E	M	G	T	V	Y	H	A	L	G	S	Q	I	D	V	V	E	M	F	D	Q	V
				I	P	A	A	D	K	D	I	V	K	V	F	T	K	R	I	S	K	K	F	N	L	M	L	E	T	K	V	T	A
				V	E	A	K	E	D	G	I	Y	V	T	M	E	G	K	K	A	P	A	E	P	Q	R	Y	D	A	V	L	V	A
				I	G	R	V	P	N	G	K	N	L	D	A	G	K	A	G	V	E	V	D	D	R	G	F	I	R	V	D	K	Q
				L	R	T	N	V	P	H	I	F	A	I	G	D	I	V	G	Q	P	M	L	A	H	K	G	V	H	E	G	H	V
				A	A	E	V	I	A	G	K	K	H	Y	F	D	P	K	V	I	P	S	I	A	Y	T	E	P	E	V	A	W	V
				G	L	T	E	K	E	A	K	E	K	G	I	S	Y	E	T	A	T	F	P	W	A	A	S	G	R	A	I	A	S
				D	C	A	D	G	M	T	K	L	I	F	D	K	E	S	H	R	V	I	G	G	A	I	V	G	T	N	G	G	E
				L	L	G	E	I	G	L	A	I	E	M	G	C	D	A	E	D	I	A	L	T	I	H	A	H	P	T	L	H	E
				S	V	G	L	A	A	E	V	F	E	G	S	I	T	D	L	P	N	P	K	A	K	K	K						
				1				5					10					15					20					25					30
25	34.15%	99.0%	>tr|C5W6Z1|C5W6Z1_ECOBD UDP-4-amino-4-deoxy-L-arabinose--oxoglutarate aminotransferase OS=Escherichia coli (strain B / BL21-DE3) GN=arnB PE=3 SV=1MAEGKAMSEFLPFSRPAMGVEELAAVKEVLESGWITTGPKNQALEQAFCQLTGNQHAIAV																														
				S	S	A	T	A	G	M	H	I	T	L	M	A	L	K	I	G	K	G	D	E	V	I	T	P	S	L	T	W	V
				S	T	L	N	M	I	S	L	L	G	A	T	P	V	M	V	D	V	D	R	D	T	L	M	V	T	P	E	A	I
				E	S	A	I	T	P	R	T	K	A	I	I	P	V	H	Y	A	G	A	P	A	D	I	D	A	I	R	A	I	G
				E	R	Y	G	I	A	V	I	E	D	A	A	H	A	V	G	T	Y	Y	K	G	R	H	I	G	A	K	G	T	A
				I	F	S	F	H	A	I	K	N	I	T	C	A	E	G	G	L	I	V	T	D	N	E	N	L	A	R	Q	L	R
				M	L	K	F	H	G	L	G	V	D	A	Y	D	R	Q	T	W	G	R	A	P	Q	A	E	V	L	T	P	G	Y
				K	Y	N	L	T	D	I	N	A	A	I	A	L	T	Q	L	V	K	L	E	H	L	N	T	R	R	R	E	I	A
				Q	Q	Y	Q	Q	A	L	A	A	L	P	F	Q	P	L	S	L	P	A	W	P	H	V	H	A	W	H	L	F	I
				I	R	V	D	E	Q	R	C	G	I	S	R	D	A	L	M	E	A	L	K	E	R	G	I	G	T	G	L	H	F
				R	A	A	H	T	Q	K	Y	Y	R	E	R	F	P	T	L	S	L	P	N	T	E	W	N	S	E	R	I	C	S
				L	P	L	F	P	D	M	T	T	A	D	A	D	H	V	I	T	A	L	Q	Q	L	A	G	Q					
				1				5					10					15					20					25					30
26	19.12%	99.0%	>tr|A0A140SS73|A0A140SS73_ECOBD Glycerol kinase OS=Escherichia coli (strain B / BL21-DE3) OX=469008 GN=glpK PE=3 SV=1																														
				M	T	E	K	K	Y	I	V	A	L	D	Q	G	T	T	S	S	R	A	V	V	M	D	H	D	A	N	I	I	S
				V	S	Q	R	E	F	E	Q	I	Y	P	K	P	G	W	V	E	H	D	P	M	E	I	W	A	T	Q	S	S	T
				L	V	E	V	L	A	K	A	D	I	S	S	D	Q	I	A	A	I	G	I	T	N	Q	R	E	T	T	I	V	W
				E	K	E	T	G	K	P	I	Y	N	A	I	V	W	Q	C	R	R	T	A	E	I	C	E	H	L	K	R	D	G
				L	E	D	Y	I	R	S	N	T	G	L	V	I	D	P	Y	F	S	G	T	K	V	K	W	I	L	D	H	V	E
				G	S	R	E	R	A	R	R	G	E	L	L	F	G	T	V	D	T	W	L	I	W	K	M	T	Q	G	R	V	H
				V	T	D	Y	T	N	A	S	R	T	M	L	F	N	I	H	T	L	D	W	D	D	K	M	L	E	V	L	D	I
				P	R	E	M	L	P	E	V	R	R	S	S	E	V	Y	G	Q	T	N	I	G	G	K	G	G	T	R	I	P	I
				S	G	I	A	G	D	Q	Q	A	A	L	F	G	Q	L	C	V	K	E	G	M	A	K	N	T	Y	G	T	G	C
				F	M	L	M	N	T	G	E	K	A	V	K	S	E	N	G	L	L	T	T	I	A	C	G	P	T	G	E	V	N
				Y	A	L	E	G	A	V	F	M	A	G	A	S	I	Q	W	L	R	D	E	M	K	L	I	N	D	A	Y	D	S
				E	Y	F	A	T	K	V	Q	N	T	N	G	V	Y	V	V	P	A	F	T	G	L	G	A	P	Y	W	D	P	Y
				A	R	G	A	I	F	G	L	T	R	G	V	N	A	N	H	I	I	R	A	T	L	E	S	I	A	Y	Q	T	R
				D	V	L	E	A	M	Q	A	D	S	G	I	R	L	H	A	L	R	V	D	G	G	A	V	A	N	N	F	L	M
				Q	F	Q	S	D	I	L	G	T	R	V	E	R	P	E	V	R	E	V	T	A	L	G	A	A	Y	L	A	G	L
				A	V	G	F	W	Q	N	L	D	E	L	Q	E	K	A	V	I	E	R	E	F	R	P	G	I	E	T	T	E	R
				N	Y	R	Y	A	G	W	K	K	A	V	K	R	A	M	A	W	E	E	H	D	E								
				1				5					10					15					20					25					30
27	28.24%	99.0%	>tr|A0A140N582|A0A140N582_ECOBD Cell shape determining proteinMreB/Mrl family OS=Escherichia coli (strain B / BL21-DE3) OX=469008 GN=ECBD_0496 PE=4 SV=1																														
				M	L	K	K	F	R	G	M	F	S	N	D	L	S	I	D	L	G	T	A	N	T	L	I	Y	V	K	G	Q	G
				I	V	L	N	E	P	S	V	V	A	I	R	Q	D	R	A	G	S	P	K	S	V	A	A	V	G	H	D	A	K
				Q	M	L	G	R	T	P	G	N	I	A	A	I	R	P	M	K	D	G	V	I	A	D	F	F	V	T	E	K	M
				L	Q	H	F	I	K	Q	V	H	S	N	S	F	M	R	P	S	P	R	V	L	V	C	V	P	V	G	A	T	Q
				V	E	R	R	A	I	R	E	S	A	Q	G	A	G	A	R	E	V	F	L	I	E	E	P	M	A	A	A	I	G
				A	G	L	P	V	S	E	A	T	G	S	M	V	V	D	I	G	G	G	T	T	E	V	A	V	I	S	L	N	G
				V	V	Y	S	S	S	V	R	I	G	G	D	R	F	D	E	A	I	I	N	Y	V	R	R	N	Y	G	S	L	I
				G	E	A	T	A	E	R	I	K	H	E	I	G	S	A	Y	P	G	D	E	V	R	E	I	E	V	R	G	R	N
				L	A	E	G	V	P	R	G	F	T	L	N	S	N	E	I	L	E	A	L	Q	E	P	L	T	G	I	V	S	A
				V	M	V	A	L	E	Q	C	P	P	E	L	A	S	D	I	S	E	R	G	M	V	L	T	G	G	G	A	L	L
				R	N	L	D	R	L	L	M	E	E	T	G	I	P	V	V	V	A	E	D	P	L	T	C	V	A	R	G	G	G
				K	A	L	E	M	I	D	M	H	G	G	D	L	F	S	E	E													
				1				5					10					15					20					25					30
28	28.45%	99.0%	>tr|A0A140N1Y4|A0A140N1Y4_ECOBD L-threonine 3-dehydrogenase OS=Escherichia coli (strain B / BL21-DE3) OX=469008 GN=tdh PE=3 SV=1																														
				M	K	A	L	S	K	L	K	A	E	E	G	I	W	M	T	D	V	P	V	P	E	L	G	H	N	D	L	L	I
				K	I	R	K	T	A	I	C	G	T	D	V	H	I	Y	N	W	D	E	W	S	Q	K	T	I	P	V	P	M	V
				V	G	H	E	Y	V	G	E	V	V	G	I	G	Q	E	V	K	G	F	K	I	G	D	R	V	S	G	E	G	H
				I	T	C	G	H	C	R	N	C	R	G	G	R	T	H	L	C	R	N	T	I	G	V	G	V	N	R	P	G	C
				F	A	E	Y	L	V	I	P	A	F	N	A	F	K	I	P	D	N	I	S	D	D	L	A	S	I	F	D	P	F
				G	N	A	V	H	T	A	L	S	F	D	L	V	G	E	D	V	L	V	S	G	A	G	P	I	G	I	M	A	A
				A	V	A	K	H	V	G	A	R	N	V	V	I	T	D	V	N	E	Y	R	L	E	L	A	R	K	M	G	I	T
				R	A	V	N	V	A	K	E	N	L	N	D	V	M	A	E	L	G	M	T	E	G	F	D	V	G	L	E	M	S
				G	A	P	P	A	F	R	T	M	L	D	T	M	N	H	G	G	R	I	A	M	L	G	I	P	P	S	D	M	S
				I	D	W	T	K	V	I	F	K	G	L	F	I	K	G	I	Y	G	R	E	M	F	E	T	W	Y	K	M	A	A
				L	I	Q	S	G	L	D	L	S	P	I	I	T	H	R	F	S	I	D	D	F	Q	K	G	F	D	A	M	R	S
				G	Q	S	G	K	V	I	L	S	W	D																			
				1				5					10					15					20					25					30
29	25.32%	99.0%	>tr|A0A140N6Z1|A0A140N6Z1_ECOBD Mannose-6-phosphate isomerase OS=Escherichia coli (strain B / BL21-DE3) OX=469008 GN=ECBD_2033 PE=3 SV=1																														
				M	Q	K	L	I	N	S	V	Q	N	Y	A	W	G	S	K	T	A	L	T	E	L	Y	G	M	E	N	P	S	S
				Q	P	M	A	E	L	W	M	G	A	H	P	K	S	S	S	R	V	Q	N	A	A	G	D	I	V	S	L	R	D
				V	I	E	S	D	K	S	T	L	L	G	E	A	V	A	K	R	F	G	E	L	P	F	L	F	K	V	L	C	A

A	Q	P	L	S	I	Q	V	H	P	N	K	H	N	S	E	I	G	F	A	K	E	N	A	A	G	I	P	M	D
A	A	E	R	N	Y	K	D	P	N	H	K	P	E	L	V	F	A	L	T	P	F	L	A	M	N	A	F	R	E
F	S	E	I	V	S	L	L	Q	P	V	A	G	A	H	P	A	I	A	H	F	L	Q	Q	P	D	A	E	R	L
S	E	L	F	A	S	L	L	N	M	Q	G	E	E	K	S	H	A	L	A	I	L	K	S	A	L	D	S	Q	Q
G	E	P	W	Q	T	I	R	L	I	S	E	F	Y	P	E	D	S	G	L	F	S	P	L	L	L	N	V	V	K
L	N	P	G	E	A	M	F	L	F	A	E	T	P	H	A	Y	L	Q	G	V	A	L	E	V	M	A	N	S	D
N	V	L	R	A	G	L	T	P	K	Y	I	D	I	P	E	L	V	A	N	V	K	F	E	A	K	P	A	N	Q
L	L	T	Q	P	V	K	Q	G	A	E	L	D	F	P	I	P	V	D	D	F	A	F	S	L	H	D	L	S	D
K	E	T	T	I	S	Q	Q	S	A	A	I	L	F	C	V	E	G	D	A	T	L	W	K	G	S	Q	Q	L	Q
L	K	P	G	E	S	A	F	I	A	A	N	E	S	P	V	T	V	K	G	H	G	R	L	A	R	V	Y	N	K
L																													
1				5					10					15					20					25					30

30 | 19.44% | 99.0% | >tr|Q8RFC6|Q8RFC6_FUSNN Electron transfer flavoprotein alpha-subunit OS=Fusobacterium nucleatum subsp. nucleatum (strain ATCC 25586 / CIP 101130 / JCM 8532 / LMG 13131) OX=190304 GN=FN0785 PE=4 SV=1

M	N	L	N	D	Y	K	G	I	L	V	F	A	E	Q	R	D	G	I	L	Q	N	V	G	L	E	L	L	G	K
A	T	E	L	A	Y	E	I	N	K	Q	I	A	L	K	D	A	G	D	E	L	A	D	F	A	G	K	K	E	A
A	I	K	S	V	D	V	A	A	A	T	L	E	E	D	D	Q	E	L	K	N	K	V	A	D	I	K	K	N	N
P	D	A	A	K	V	T	A	L	L	V	G	H	N	I	K	N	L	A	Q	D	L	I	K	A	G	A	D	K	V
L	V	V	D	K	P	E	L	E	V	Y	D	T	E	A	Y	A	Q	V	L	T	A	T	I	N	D	Q	K	P	E
I	V	L	F	G	A	T	T	L	G	R	D	L	A	P	R	V	S	S	R	I	A	T	G	L	T	A	D	C	T
K	L	E	L	L	K	D	K	E	R	Q	L	G	M	T	R	P	A	F	G	G	N	L	M	A	T	I	V	S	P
D	H	R	P	Q	M	A	T	V	R	P	G	V	M	K	K	L	P	K	S	D	D	R	T	G	E	V	V	E	F
P	V	T	L	D	T	S	K	M	K	V	K	L	L	K	V	V	K	E	G	G	N	K	V	D	I	S	E	A	K
I	L	V	S	G	G	R	G	V	G	S	K	Q	N	F	E	L	L	E	D	L	A	T	E	I	G	G	I	V	S
S	S	R	A	Q	V	D	A	G	N	M	P	H	D	R	Q	V	G	Q	T	G	K	T	V	R	P	E	V	Y	F
A	C	G	I	S	G	A	I	Q	H	V	A	G	M	E	E	S	E	F	I	I	A	I	N	K	D	R	F	A	P
I	F	S	V	A	D	L	G	I	V	G	D	L	H	K	I	L	P	I	L	T	E	E	I	K	K	Y	K	A	T
K																													
1				5					10					15					20					25					30

31 | 21.67% | 99.0% | >tr|A0A140N7B0|A0A140N7B0_ECOBD Acetylornithine/succinyldiaminopimelate aminotransferase OS=Escherichia coli (strain B / BL21-DE3) OX=469008 GN=argD PE=3 SV=1

M	A	I	E	Q	T	A	I	T	R	A	T	F	D	E	V	I	L	P	I	Y	A	P	A	E	F	I	P	V	K
G	Q	G	S	R	I	W	D	Q	Q	G	K	E	Y	V	D	F	A	G	G	I	A	V	T	A	L	G	H	C	H
P	A	L	V	N	A	L	K	T	Q	G	E	T	L	W	H	I	S	N	V	F	T	N	E	P	A	L	R	L	G
R	K	L	I	E	A	T	F	A	E	R	V	V	F	M	N	S	G	T	E	A	N	E	T	A	F	K	L	A	R
H	Y	A	C	V	R	H	S	P	F	K	T	K	I	I	A	F	H	N	A	F	H	G	R	S	L	F	T	V	S
V	G	G	Q	P	K	Y	S	D	G	F	G	P	K	P	A	D	I	I	H	V	P	F	N	D	L	H	A	V	K
A	V	M	D	D	H	T	C	A	V	V	V	E	P	I	Q	G	E	G	G	V	T	A	A	T	P	E	F	L	Q
G	L	R	E	L	C	D	Q	H	Q	A	L	L	V	F	D	E	V	Q	C	G	M	G	R	T	G	D	L	F	A
Y	M	H	Y	G	V	T	P	D	I	L	T	S	A	K	A	L	G	G	G	F	P	I	S	A	M	L	T	T	A
E	I	A	S	A	F	H	P	G	S	H	G	S	T	Y	G	G	N	P	L	A	C	A	V	A	G	A	A	F	D
I	I	N	T	P	E	V	L	E	G	I	Q	A	K	R	Q	R	F	V	D	H	L	Q	K	I	D	Q	Q	Y	D
V	F	S	D	I	R	G	M	G	L	L	I	G	A	E	L	K	P	Q	Y	K	G	R	A	R	D	F	L	Y	A
G	A	E	A	G	V	M	V	L	N	A	G	P	D	V	M	R	F	A	P	S	L	V	V	E	D	A	D	I	D
E	G	M	Q	R	F	A	H	A	V	A	K	V	V	G	A														
1				5					10					15					20					25					30

32 | 26.36% | 99.0% | >tr|A0A140N5B5|A0A140N5B5_ECOBD Iron-containing alcohol dehydrogenase OS=Escherichia coli (strain B / BL21-DE3) OX=469008 GN=ECBD_0729 PE=4 SV=1

M	N	N	F	N	L	H	T	P	T	R	I	L	F	G	K	G	A	I	A	G	L	R	E	Q	I	P	H	D	A
R	V	L	I	T	Y	G	G	G	S	V	K	K	T	G	V	L	D	Q	V	L	D	A	L	K	G	M	D	V	L
E	F	G	G	I	E	P	N	P	A	Y	E	T	L	M	N	A	V	K	L	V	R	E	Q	K	V	T	F	L	L
A	V	G	G	G	S	V	L	D	G	T	K	F	I	A	A	A	A	N	Y	P	E	N	I	D	P	W	H	I	L
Q	T	G	G	K	E	I	K	S	A	I	P	M	G	C	V	L	T	L	P	A	T	G	S	E	S	N	A	G	A
V	I	S	R	K	T	T	G	D	K	Q	A	F	H	S	A	H	V	Q	P	V	F	A	V	L	D	P	V	Y	T
Y	T	L	P	P	R	Q	V	A	N	G	V	V	D	A	F	V	H	T	V	E	Q	Y	V	T	K	P	V	D	A
K	I	Q	D	R	F	A	E	G	I	L	L	T	L	I	E	D	G	P	K	A	L	K	E	P	E	N	Y	D	V
R	A	N	V	M	W	A	A	T	Q	A	L	N	G	L	I	G	A	G	V	P	Q	D	W	A	T	H	M	L	G
H	E	L	T	A	M	H	G	L	D	H	A	Q	T	L	A	I	V	L	P	A	L	W	N	E	K	R	D	T	K
R	A	K	L	L	Q	Y	A	E	R	V	W	N	I	T	E	G	S	D	D	E	R	I	D	A	A	I	A	A	T
R	N	F	F	E	Q	L	G	V	P	T	H	L	S	D	Y	G	L	D	G	S	S	I	P	A	L	L	K	K	L
E	E	H	G	M	T	Q	L	G	E	N	H	D	I	T	L	D	V	S	R	R	I	Y	E	A	A	R			
1				5					10					15					20					25					30

33 | 15.66% | 99.0% | >tr|A0A140N8G3|A0A140N8G3_ECOBD Aminomethyltransferase OS=Escherichia coli (strain B / BL21-DE3) OX=469008 GN=gcvT PE=3 SV=1

M	A	Q	Q	T	P	L	Y	E	Q	H	T	L	C	G	A	R	M	V	D	F	H	G	W	M	M	P	L	H	Y
G	S	Q	I	D	E	H	H	A	V	R	T	D	A	G	M	F	D	V	S	H	M	T	I	V	D	L	R	G	S
R	T	R	E	F	L	R	Y	L	L	A	N	D	V	A	K	L	T	K	S	G	K	A	L	Y	S	G	M	L	N
A	S	G	G	V	I	D	D	L	I	V	Y	Y	F	T	E	D	F	F	R	L	V	V	N	S	A	T	R	E	K
D	L	S	W	I	T	Q	H	A	E	P	F	G	I	E	I	T	V	R	D	D	L	S	M	I	A	V	Q	G	P
N	A	Q	A	K	A	A	T	L	F	N	D	A	Q	R	Q	A	V	E	G	M	K	P	F	F	G	V	Q	A	G
D	L	F	I	A	T	T	G	Y	T	G	E	A	G	Y	E	I	A	L	P	N	E	K	A	A	D	F	W	R	A
L	V	E	A	G	V	K	P	C	G	L	G	A	R	D	T	L	R	L	E	A	G	M	N	L	Y	G	Q	E	M
D	E	T	I	S	P	L	A	A	N	M	G	W	T	I	A	W	E	P	A	D	R	D	F	I	G	R	E	A	L
E	V	Q	R	E	H	G	T	E	K	L	V	G	L	V	M	T	E	K	G	V	L	R	N	E	L	P	V	R	F
T	D	A	Q	G	N	Q	H	E	G	I	I	T	S	G	T	F	S	P	T	L	G	Y	S	I	A	L	A	R	V
P	E	G	I	G	E	T	A	I	V	Q	I	R	N	R	E	M	P	V	K	V	T	K	P	V	F	V	R	N	G
K	A	V	A																										
1				5					10					15					20					25					30

34 | 25.00% | 99.0% | >tr|A0A140NGE1|A0A140NGE1_ECOBD Fructose-16-bisphosphatase class 1 OS=Escherichia coli (strain B / BL21-DE3) OX=469008 GN=fbp PE=3 SV=1

M	K	T	L	G	E	F	I	V	E	K	Q	H	E	F	S	H	A	T	G	E	L	T	A	L	L	S	A	I	K
L	G	A	K	I	I	H	R	D	I	N	K	A	G	L	V	D	I	L	G	A	S	G	A	E	N	V	Q	G	E
V	Q	Q	K	L	D	L	F	A	N	E	K	L	K	A	A	L	K	A	R	D	I	V	A	G	I	A	S	E	E
E	D	E	I	V	V	F	E	G	C	E	H	A	K	Y	V	V	L	M	D	P	L	D	G	S	S	N	I	D	V
N	V	S	V	G	T	I	F	S	I	Y	R	R	V	T	P	V	G	T	P	V	T	E	E	D	F	L	Q	P	G
N	K	Q	V	A	A	G	Y	V	V	Y	G	S	S	T	M	L	V	Y	T	T	G	C	G	V	H	A	F	T	Y
D	P	S	L	G	V	F	C	L	C	Q	E	R	M	R	F	P	E	K	G	K	T	Y	S	I	N	E	G	N	Y
I	K	F	P	N	G	V	K	K	Y	I	K	F	C	Q	E	E	D	K	S	T	N	R	P	Y	T	S	R	Y	I
G	S	L	V	A	D	F	H	R	N	L	L	K	G	G	I	Y	L	Y	P	S	T	A	S	H	P	D	G	K	L
R	L	L	Y	E	C	N	P	M	A	F	L	A	E	Q	A	G	G	K	A	S	D	G	K	E	R	I	L	D	I
I	P	E	T	L	H	Q	R	R	S	F	F	V	G	N	D	H	M	V	E	D	V	E	R	F	I	R	E	F	P
D	A																												
1				5					10					15					20					25					30

35 | 32.02% | 99.0% | >tr|A0A140NA20|A0A140NA20_ECOBD Dihydroxyacetone kinaseDhaK subunit OS=Escherichia coli (strain B / BL21-DE3) OX=469008 GN=ECBD_2422 PE=4 SV=1

M	K	K	L	I	N	D	V	Q	D	V	L	D	E	Q	L	A	G	L	A	K	A	H	P	S	L	T	L	H	Q
D	P	V	Y	V	T	R	A	D	A	P	V	A	G	K	V	A	L	L	S	G	G	G	S	G	H	E	P	M	H
C	G	Y	I	G	Q	G	M	L	S	G	A	C	P	G	E	I	F	T	S	P	T	P	D	K	I	F	E	C	A
M	Q	V	D	G	G	E	G	V	L	L	I	I	K	N	Y	T	G	D	I	L	N	F	E	T	A	T	E	L	L
H	D	S	G	V	K	V	T	T	V	V	I	D	D	D	V	A	V	K	D	S	L	Y	T	A	G	R	R	G	V
A	N	T	V	L	I	E	K	L	V	G	A	A	A	E	R	G	D	S	L	D	A	C	A	E	L	G	R	K	L
N	N	Q	G	H	S	I	G	I	A	L	G	A	C	T	V	P	A	A	G	K	P	S	F	T	L	A	D	N	E
M	E	F	G	V	G	I	H	G	E	P	G	I	D	R	R	P	F	S	S	L	D	Q	T	V	D	E	M	F	D
T	L	L	V	N	G	S	Y	H	R	T	L	R	F	W	D	Y	Q	Q	G	S	W	Q	E	E	Q	Q	T	K	Q
P	L	Q	S	G	D	R	V	I	A	L	V	N	N	L	G	A	T	P	L	S	E	L	Y	G	V	Y	N	R	L
T	T	R	C	Q	Q	A	G	L	T	I	E	R	N	L	I	G	A	Y	C	T	S	L	D	M	T	G	F	S	I
T	L	L	K	V	D	D	E	T	L	A	L	W	D	A	P	V	H	T	P	A	L	N	W	G	K				
1				5					10					15					20					25					30

36 | 28.92% | 99.0% | >tr|A0A140N7T7|A0A140N7T7_ECOBD DegT/DnrJ/EryC1/StrS aminotransferase OS=Escherichia coli (strain B / BL21-DE3) OX=469008 GN=ECBD_1619 PE=3 SV=1

M	N	D	K	T	I	P	V	T	Q	P	S	L	P	E	L	A	E	F	M	P	Y	L	E	K	I	W	K	N	K
W	L	T	N	N	G	P	F	H	Q	E	L	E	E	K	L	C	E	F	L	G	V	Q	H	I	S	L	F	N	N
A	T	I	A	L	I	T	A	L	Q	A	L	R	I	T	G	E	V	I	T	T	P	Y	S	F	V	A	T	S	H
A	I	L	W	N	G	L	T	P	V	F	V	D	I	E	N	D	G	Y	N	I	D	Y	R	K	I	E	Q	A	I
T	P	K	T	S	A	I	L	P	V	H	C	Y	S	T	P	C	E	V	E	E	I	Q	K	I	A	D	N	Y	G
L	K	V	I	Y	D	A	A	H	A	F	G	V	N	F	K	G	Q	S	L	L	N	Y	G	D	L	S	I	L	S
F	H	A	T	K	V	F	N	T	F	E	G	G	A	I	I	S	P	D	A	K	T	K	L	R	I	D	R	L	K
N	F	G	I	A	D	E	L	T	V	T	A	P	G	I	N	G	K	M	S	E	I	N	A	A	F	G	L	V	Q
L	K	H	I	E	G	S	I	S	K	R	K	I	I	D	S	L	Y	R	N	L	L	K	G	T	P	G	I	T	I
F	P	G	N	I	N	A	N	S	N	Y	S	Y	F	P	I	L	I	D	D	G	F	H	M	S	R	D	Q	A	Y
E	L	L	K	K	N	N	I	L	S	R	K	Y	F	Y	P	L	I	S	N	M	P	M	Y	R	G	L	I	S	A
S	V	D	N	L	P	I	A	N	S	V	A	D	K	V	L	C	L	P	I	Y	T	D	L	N	E	E	I	V	V
K	I	T	K	L	L	L	G	K	M																				
1				5					10					15					20					25					30

37 | 28.88% | 99.0% | >tr|C5WA11|C5WA11_ECOBD Aspartate-semialdehyde dehydrogenase OS=Escherichia coli (strain B / BL21-DE3) GN=asd PE=3 SV=1

M	K	N	V	G	F	I	G	W	R	G	M	V	G	S	V	L	M	Q	R	M	V	E	E	R	D	F	D	A	I
R	P	V	Y	F	S	T	S	Q	L	G	Q	A	A	P	S	F	G	G	T	T	G	T	L	Q	D	A	F	D	L
E	A	L	K	A	L	D	I	I	V	T	C	Q	G	G	D	Y	T	N	E	I	Y	P	K	L	R	E	S	G	W
Q	G	Y	W	I	D	A	A	S	S	L	R	M	K	D	D	A	I	I	I	L	D	P	V	N	Q	D	V	I	T
D	G	L	N	N	G	I	R	T	F	V	G	G	N	C	T	V	S	L	M	L	M	S	L	G	G	L	F	A	N
D	L	V	D	W	V	S	V	A	T	Y	Q	A	A	S	G	G	G	A	R	H	M	R	E	L	L	T	Q	M	G
H	L	Y	G	H	V	A	D	E	L	A	T	P	S	S	A	I	L	D	I	E	R	K	V	T	T	L	T	R	S
G	E	L	P	V	D	N	F	G	V	P	L	A	G	S	L	I	P	W	I	D	K	Q	L	D	N	G	Q	S	R
E	E	W	K	G	Q	A	E	T	N	K	I	L	N	T	S	S	V	I	P	V	D	G	L	C	V	R	V	G	A
L	R	C	H	S	Q	A	F	T	I	K	L	K	K	D	V	S	I	P	T	V	E	E	L	L	A	A	H	N	P
W	A	K	V	V	P	N	D	R	E	I	T	M	R	E	L	T	P	A	A	V	T	G	T	L	T	T	P	V	G
R	L	R	K	L	N	M	G	P	E	F	L	S	A	F	T	V	G	D	Q	L	L	W	G	A	A	E	P	L	R
R	M	L	R	Q	L	A																							
1				5					10					15					20					25					30

38	20.54%	99.0%	>tr|A0A140N9D5|A0A140N9D5_ECOBD Cysteine desulfurase IscS OS=Escherichia coli (strain B / BL21-DE3) OX=469008 GN=iscS PE=3 SV=1
			M K L P I Y L D Y S A T T P V D P R V A E K M M Q F M T M D
			G T F G N P A S R S H R F G W Q A E E A V D I A R N Q I A D
			L V G A D P R E I V F T S G A T E S D N L A I K G A A N F Y
			Q K K G K H I I T S K T E H K A V L D T C R Q L E R E G F E
			V T Y L A P Q R N G I I D L K E L E A A M R D D T I L V S I
			M H V N N E I G V V Q D I A A I G E M C R A R G I I Y H V D
			A T Q S V G K L P I D L S Q L K V D L M S F S G H K I Y G P
			K G I G A L Y V R R K P R V R I E A Q M H G G G H E R G M R
			S G T L P V H Q I V G M G E A Y R I A K E E M A T E M E R L
			R G L R N R L W N G I K D I E E V Y L N G D L E H G A P N I
			L N V S F N Y V E G E S L I M A L K D L A V S S G S A C T S
			A S L E P S Y V L R A L G L N D E L A H S S I R F S L G R F
			T T E E E I D Y T I E L V R K S I G R L R D L S P L W E M Y
			K Q G V D L N S I E W A H H
			1 5 10 15 20 25 30
39	17.88%	99.0%	>tr|A0A140NEW5|A0A140NEW5_ECOBD Aminotransferase OS=Escherichia coli (strain B / BL21-DE3) OX=469008 GN=ECBD_3979 PE=3 SV=1
			M F Q K V D A Y A G D P I L T L M E R F K E D P R S D K V N
			L S I G L Y Y N E D G I I P Q L Q A V A E A E A R L N A Q P
			H G A S L Y L P M E G L N S Y R H A I A P L L F G A D H P V
			L K Q Q R V A T I Q T L G G S G A L K V G A D F L K R Y F P
			E S G V W V S D P T W E N H V A I F A G A G F E V S T Y P W
			Y D E A T N G V R F N D L L A T L K T L P A R S I V L L H P
			C C H N P T G A D L T N D Q W D A V I E I L K A R E L I P F
			L D I A Y Q G F G A G M E E D A Y A I R A I A S A G L P A L
			V S N S F S K I F S L Y G E R V G G L S V M C E D A E A A G
			R V L G Q L K A T V R R N Y S S P P N F G A Q V V A A V L N
			D E A L K A S W L A E V E E M R T R I L A M R Q E L V K V L
			S T E M P E R N F D Y L L N Q R G M F S Y T G L S A A Q V D
			R L R E E F G V Y L I A S G R M C V A G L N T A N V Q R V A
			K A F A A V M
			1 5 10 15 20 25 30
40	25.64%	99.0%	>tr|A0A140NE48|A0A140NE48_ECOBD Isoaspartyl dipeptidase OS=Escherichia coli (strain B / BL21-DE3) OX=469008 GN=ECBD_3703 PE=3 SV=1
			M I D Y T A A G F T L L Q G A H L Y A P E D R G I C D V L V
			A N G K I I A V A S N I P S D I V P N C T V V D L S G Q I L
			C P G F I D Q H V H L I G G G G E A G P T T R T P E V A L S
			R L T E A G V T S V V G L L G T D S I S R H P E S L L A K T
			R A L N E E G I S A W M L T G A Y H V P S R T I T G S V E K
			D V A I I D R V I G V K C A I S D H R S A A P D V Y H L A N
			M A A E S R V G G L L G G K P G V T V F H M G D S K K A L Q
			P I Y D L L E N C D V P I S K L L P T H V N R N V P L F E Q
			A L E F A R K G G T I D I T S S I D E P V A P A E G I A R A
			V Q A G I P L A R V T L S S D G N G S Q P F F D D E G N L T
			H I G V A G F E T L L E T V Q V L V K D Y D F S I S D A L R
			P L T S S V A G F L N L T G K G E I L P G N D A D L L V M T
			P E L R I E Q V Y A R G K L M V K D G K A C V K G T F E T A
			1 5 10 15 20 25 30
41	17.85%	99.0%	>tr|X7S5D3|X7S5D3_FUSNU Uncharacterized protein OS=Fusobacterium nucleatum 13_3C OX=1357398 GN=HMPREF2085_00804 PE=3 SV=1
			M E F N V P K T H E L F R Q M I R E F V E K E V K P I A A E
			V D E N E R F P M E T V E K M A K I G I M G I P I P K E Y G
			G A G G D N L M Y A M A V E E L S K A C A T T G V I V S A H
			T S L G T W P I L K F G N E K Q K Q K Y L P K M A S G E W I
			G A F G L T E P N A G T D A A G Q Q T M A V Q D P E T G E W
			I L N G A K I F I T N A G Y A H V Y V V F A M T D K S K G L
			K G I S A F I V E A N T P G F S I G K K E M K L G I R G S A
			T C E L I F E N C R I P K E N L L G D K G K G F K I A M M T
			L D G G R I G I A S Q A L G I A A G A L D E A I N Y A K E R
			K Q F G R S L A Q F Q N T Q F Q I A N L D V K V E A A R L L
			V Y K A A W R E S N N L P Y S L D A A R A K L F A A E T A M
			E V T T K A V Q I F G G Y G Y T R E Y P V E R M M R D A K I
			T E I Y E G T S E V Q R M V I A A N I I K
			1 5 10 15 20 25 30
42	17.56%	99.0%	>tr|A0A140N4Y8|A0A140N4Y8_ECOBD Protein RecA OS=Escherichia coli (strain B / BL21-DE3) OX=469008 GN=recA PE=3 SV=1
			M A I D E N K Q K A L A A A L G Q I E K Q F G K G S I M R L
			G E D R S M D V E T I S T G S L S L D I A L G A G G L P M G
			R I V E I Y G P E S S G K T T L T L Q V I A A A Q R E G K T
			C A F I D A E H A L D P I Y A R K L G V D I D N L L C S Q P
			D T G E Q A L E I C D A L A R S G A V D V I V V D S V A A L
			T P K A E I E G E I G D S H M G L A A R M M S Q A M R K L A
			G N L K Q S N T L L I F I N Q I R M K I G V M F G N P E T T
			T G G N A L K F Y A S V R L D I R R I G A V K E G E N V V G
			S E T R V K V V K N K I A A P F K Q A E F Q I L Y G E G I N
			F Y G E L V D L G V K E K L I E K A G A W Y S Y K G E K I G
			Q G K A N A T A W L K D N P E T A K E I E K K V R E L L L S
			N P N S T P D F S V D D S E G V A E T N E D F
			1 5 10 15 20 25 30
43	20.43%	99.0%	>tr|A0A140NHS0|A0A140NHS0_ECOBD ATP synthase subunit beta OS=Escherichia coli (strain B / BL21-DE3) OX=469008 GN=atpD PE=3 SV=1
			M A T G K I V Q V I G A V V D V E F P Q D A V P R V Y D A L
			E V Q N G N E R L V L E V Q Q Q L G G G I V R T I A M G S S
			D G L R R G L D V K D L E H P I E V P V G K A T L G R I M N
			V L G E P V D M K G E I G E E E R W A I H R A A P S Y E E L
			S N S Q E L L E T G I K V I D L M C P F A K G G K V G L F G
			G A G V G K T V N M M E L I R N I A I E H S G Y S V F A G V
			G E R T R E G N D F Y H E M T D S N V I D K V S L V Y G Q M
			N E P P G N R L R V A L T G L T M A E K F R D E G R D V L L
			F V D N I Y R Y T L A G T E V S A L L G R M P S A V G Y Q P
			T L A E E M G V L Q E R I T S T K T G S I T S V Q A V Y V P
			A D D L T D P S P A T T F A H L D A T V V L S R Q I A S L G
			I Y P A V D P L D S T S R Q L D P L V V G Q E H Y D T A R G
			V Q S I L Q R Y Q E L K D I I A I L G M D E L S E E D K L V
			V A R A R K I Q R F L S Q P F F V A E V F T G S P G K Y V S
			L K D T I R G F K G I M E G E Y D H L P E Q A F Y M V G S I
			E E A V E K A K K L
			1 5 10 15 20 25 30
44	6.97%	99.0%	>tr|A0A140NBE7|A0A140NBE7_ECOBD Formate acetyltransferase OS=Escherichia coli (strain B / BL21-DE3) OX=469008 GN=ECBD_2692 PE=4 SV=1
			M S E L N E K L A T A W E G F T K G D W Q N E V N V R D F I
			Q K N Y T P Y E G D E S F L A G A T E A T T T L W D K V M E
			G V K L E N R T H A P V D F D T A V A S T I T S H D A G Y I
			N K Q L E K I V G L Q T E A P L K R A L I P F G G I K M I E
			G S C K A Y N R E L D P M I K K I F T E Y R K T H N Q G V F
			D V Y T P D I L R C R K S G V L T G L P D A Y G R G R I I G
			D Y R R V A L Y G I D Y L M K D K L A Q F T S L Q A D L E N
			G V N L E Q T I R L R E E I A E Q H R A L G Q M K E M A A K
			Y G Y D I S G P A T N A Q E A I Q W T Y F G Y L A A V K S Q
			N G A A M S F G R T S T F L D V Y I E R D L K A G K I T E Q
			E A Q E M V D H L V M K L R M V R F L R T P E Y D E L F S G
			D P I W A T E S I G G M G L D G R T L V T K N S F R F L N T
			L Y T M G P S P E P N M T I L W S E K L P L N F K K F A A K
			V S I D T S S L Q Y E N D D L M R P D F N N D D Y A I A C C
			V S P M I V G K Q M Q F F G A R A N L A K T M L Y A I N G G
			V D E K L K M Q V G P K S E P I K G D V L N Y D E V M E R M
			D H F M D W L A K Q Y I T A L N I I H Y M H D K Y S Y E A S
			L M A L H D R D V I R T M A C G I A G L S V A A D S L S A I
			K Y A K V K P I R D E D G L A I D F E I E G E Y P Q F G N N
			D P R V D D L A V D L V E R F M K K I Q K L H T Y R D A I P
			T Q S V L T I T S N V V Y G K K T G N T P D G R R A G A P F
			G P G A N P M H G R D Q K G A V A S L T S V A K L P F A Y A
			K D G I S Y T F S I V P N A L G K D D E V R K T N L A G L M
			D G Y F H H E A S I E G G Q H L N V N V M N R E M L L D A M
			E N P E K Y P Q L T I R V S G Y A V R F N S L T K E Q Q Q D
			V I T R T F T Q S M
			1 5 10 15 20 25 30
45	8.23%	99.0%	>tr|A0A140NDL0|A0A140NDL0_ECOBD Pyruvate dehydrogenase E1 component OS=Escherichia coli (strain B / BL21-DE3) OX=469008 GN=ECBD_3505 PE=4 SV=1
			M S E R F P N D V D P I E T R D W L Q A I E S V I R E E G V
			E R A Q Y L I D Q L L A E A R K G G V N V A A G T G I S N Y
			I N T I P V E E Q P E Y P G N L E L E R R I R S A I R W N A
			I M T V L R A S K K D L E L G G H M A S F Q S S A T I Y D V
			C F N H F F R A R N E Q D G G D L V Y F Q G H I S P G V Y A
			R A F L E G R L T Q E Q L D N F R Q E V H G N G L S S Y P H
			P K L M P E F W Q F P T V S M G L G P I G A I Y Q A K F L K
			Y L E H R G L K D T S K Q T V Y A F L G D G E M D E P E S K
			G A I T I A T R E K L D N L V F V I N C N L Q R L D G P V T
			G N G K I I N E L E G I F E G A G W N V I K V M W G S R W D

E	L	L	R	K	D	T	S	G	K	L	I	Q	L	M	N	E	T	V	D	G	D	Y	Q	T	F	K	S	K	D
G	A	Y	V	R	E	H	F	F	G	K	Y	P	E	T	A	A	L	V	A	D	W	T	D	E	Q	I	W	A	L
N	R	G	G	H	D	P	K	K	I	Y	A	A	F	K	K	A	Q	E	T	K	G	K	A	T	V	I	L	A	H
T	I	K	G	Y	G	M	G	D	A	A	E	G	K	N	I	A	H	Q	V	K	K	M	N	M	D	G	V	R	H
I	R	D	R	F	N	V	P	V	S	D	T	D	I	E	K	L	P	Y	I	T	F	P	E	G	S	E	E	H	T
Y	L	H	A	Q	R	Q	K	L	H	G	Y	L	P	S	R	Q	P	N	F	T	E	K	L	E	L	P	S	L	Q
D	F	G	A	L	L	E	E	Q	S	K	E	I	S	T	T	I	A	F	V	R	A	L	N	V	M	L	K	N	K
S	I	K	D	R	L	V	P	I	I	A	D	E	A	R	T	F	G	M	E	G	L	F	R	Q	I	G	I	Y	S
P	N	G	Q	Q	Y	T	P	Q	D	R	E	Q	V	A	Y	Y	K	E	D	E	K	G	Q	I	L	Q	E	G	I
N	E	L	G	A	G	C	S	W	L	A	A	A	T	S	Y	S	T	N	N	L	P	M	I	P	F	Y	I	Y	Y
S	M	F	G	F	Q	R	I	G	D	L	C	W	A	A	G	D	Q	Q	A	R	G	F	L	I	G	G	T	S	G
R	T	T	L	N	G	E	G	L	Q	H	E	D	G	H	S	H	I	Q	S	L	T	I	P	N	C	I	S	Y	D
P	A	Y	A	Y	E	V	A	V	I	M	H	D	G	L	E	R	M	Y	G	E	K	Q	E	N	V	Y	Y	Y	I
T	T	L	N	E	N	Y	H	M	P	A	M	P	E	G	A	E	E	G	I	R	K	G	I	Y	K	L	E	T	I
E	G	S	K	G	K	V	Q	L	L	G	S	G	S	I	L	R	H	V	R	E	A	A	E	I	L	A	K	D	Y
G	V	G	S	D	V	Y	S	V	T	S	F	T	E	L	A	R	D	G	Q	D	C	E	R	W	N	M	L	H	P
L	E	T	P	R	V	P	Y	I	A	Q	V	M	N	D	A	P	A	V	A	S	T	D	Y	M	K	L	F	A	E
Q	V	R	T	Y	V	P	A	D	D	Y	R	V	L	G	T	D	G	F	G	R	S	D	S	R	E	N	L	R	H
H	F	E	V	D	A	S	Y	V	V	V	A	A	L	G	E	L	A	K	R	G	E	I	D	K	K	V	V	A	D
A	I	A	K	F	N	I	D	A	D	K	V	N	P	R	L	A													
1				5					10					15					20					25					30

46 26.49% 99.0% >tr|A0A140N8N3|A0A140N8N3_ECOBD UTP--glucose-1-phosphate uridylyltransferase OS=Escherichia coli (strain B / BL21-DE3) OX=469008 GN=ECBD_2386 PE=3 SV=1

M	A	A	I	N	T	K	V	K	K	A	V	I	P	V	A	G	L	G	T	R	M	L	P	A	T	K	A	I	P
K	E	M	L	P	L	V	D	K	P	L	I	Q	Y	V	V	N	E	C	I	A	A	G	I	T	E	I	V	L	V
T	H	S	S	K	N	S	I	E	N	H	F	D	T	S	F	E	L	E	A	M	L	E	K	R	V	K	R	Q	L
L	D	E	V	Q	S	I	C	P	P	H	V	T	I	M	Q	V	R	Q	G	L	A	K	G	L	G	H	A	V	L
C	A	H	P	V	V	G	D	E	P	V	A	V	I	L	P	D	V	I	L	D	E	Y	E	S	D	L	S	Q	D
N	L	A	E	M	I	R	R	F	D	E	T	G	H	S	Q	I	M	V	E	P	V	A	D	V	T	A	Y	G	V
V	D	C	K	G	V	E	L	A	P	G	E	S	V	P	M	V	G	V	V	E	K	P	K	A	D	V	A	P	S
N	L	A	I	V	G	R	Y	V	L	S	A	D	I	W	P	L	L	A	K	T	P	P	G	A	G	D	E	I	Q
L	T	D	A	I	D	M	L	I	E	K	E	T	V	E	A	Y	H	M	K	G	K	S	H	D	C	G	N	K	L
G	Y	M	Q	A	F	V	E	Y	G	I	R	H	N	T	L	G	T	E	F	K	A	W	L	E	E	E	M	G	I
K	K																												
1				5					10					15					20					25					30

47 15.26% 99.0% >tr|A0A140NAF9|A0A140NAF9_ECOBD Lipoyl synthase OS=Escherichia coli (strain B / BL21-DE3) OX=469008 GN=lipA PE=3 SV=1

M	S	K	P	I	V	M	E	R	G	V	K	Y	R	D	A	D	K	M	A	L	I	P	V	K	N	V	A	T	E
R	E	A	L	L	R	K	P	E	W	M	K	I	K	L	P	A	D	S	T	R	I	Q	G	I	K	A	A	M	R
K	N	G	L	H	S	V	C	E	E	A	S	C	P	N	L	A	E	C	F	N	H	G	T	A	T	F	M	I	L
G	A	I	C	T	R	R	C	P	F	C	D	V	A	H	G	R	P	V	A	P	D	A	N	E	P	V	K	L	A
Q	T	I	A	D	M	A	L	R	Y	V	V	I	T	S	V	D	R	D	D	L	R	D	G	G	A	Q	H	F	A
D	C	I	T	A	I	R	E	K	S	P	Q	I	K	I	E	T	L	V	P	D	F	R	G	R	M	D	R	A	L
D	I	L	T	A	T	P	P	D	V	F	N	H	N	L	E	N	V	P	R	I	Y	R	Q	V	R	P	G	A	D
Y	N	W	S	L	K	L	L	E	R	F	K	E	A	H	P	E	I	P	T	K	S	G	L	M	V	G	L	G	E
T	N	E	E	I	I	E	V	M	R	D	L	R	R	H	G	V	T	M	L	T	L	G	Q	Y	L	Q	P	S	R
H	H	L	P	V	Q	R	Y	V	S	P	D	E	F	D	E	M	K	A	E	A	L	A	M	G	F	T	H	A	A
C	G	P	F	V	R	S	S	Y	H	A	D	L	Q	A	K	G	M	E	V	K									
1				5					10					15					20					25					30

48 15.99% 99.0% >sp|Q8R603|EFTU_FUSNN Elongation factor Tu OS=Fusobacterium nucleatum subsp. nucleatum (strain ATCC 25586 / CIP 101130 / JCM 8532 / LMG 13131) OX=190304 GN=tuf PE=3 SV=1

M	A	K	E	K	Y	E	R	S	K	P	H	V	N	I	G	T	I	G	H	V	D	H	G	K	T	T	T	T	A
A	I	S	K	V	L	S	D	K	G	W	A	S	K	V	D	F	D	Q	I	D	A	A	P	E	E	K	E	R	G
I	T	I	N	T	A	H	I	E	Y	E	T	E	K	R	H	Y	A	H	V	D	C	P	G	H	A	D	Y	V	K
N	M	I	T	G	A	A	Q	M	D	G	A	I	L	V	V	S	A	A	D	G	P	M	P	Q	T	R	E	H	I
L	L	S	R	Q	V	G	V	P	Y	I	V	V	Y	L	N	K	S	D	M	V	E	D	E	E	L	L	E	L	V
E	M	E	V	R	E	L	L	T	E	Y	G	F	P	G	D	D	I	P	V	I	R	G	S	S	L	G	A	L	N
G	E	E	K	W	V	E	K	I	L	E	L	M	E	A	V	D	N	Y	I	P	T	P	E	R	A	V	D	Q	P
F	L	M	P	I	E	D	V	F	T	I	T	G	R	G	T	V	V	T	G	R	V	E	R	G	V	I	K	V	G
E	E	I	E	I	V	G	I	K	P	T	T	K	T	T	C	T	G	V	E	M	F	R	K	L	L	D	Q	G	Q
A	G	D	N	I	G	V	L	L	R	G	T	K	K	E	E	V	E	R	G	Q	V	L	A	K	P	G	S	I	H
P	H	T	N	F	K	G	E	V	Y	V	L	T	K	D	E	G	G	R	H	T	P	F	F	T	G	Y	R	P	Q
F	Y	F	R	T	T	D	I	T	G	A	V	T	L	P	D	G	V	E	M	V	M	P	G	D	N	I	T	M	T
V	E	L	I	H	P	I	A	M	E	Q	G	L	R	F	A	I	R	E	G	G	R	T	V	A	S	G	V	V	S
E	I	T	K																										
1				5					10					15					20					25					30

49 16.67% 99.0% >tr|A0A140NBE2|A0A140NBE2_ECOBD N-methyl-L-tryptophan oxidase OS=Escherichia coli (strain B / BL21-DE3) OX=469008 GN=solA PE=3 SV=1

M	K	Y	D	L	I	I	I	G	S	G	S	V	G	A	A	A	G	Y	Y	A	T	R	A	G	L	N	V	L	M
T	D	A	H	M	P	P	H	Q	H	G	S	H	H	G	D	T	R	L	I	R	H	A	Y	G	E	G	E	K	Y
V	P	L	V	L	R	A	Q	T	L	W	D	E	L	S	R	H	N	E	E	D	P	I	F	V	R	S	G	V	I
N	L	G	P	A	D	S	T	F	L	A	N	V	A	H	S	A	E	Q	W	Q	L	N	V	E	K	L	D	A	Q
G	I	M	A	R	W	P	E	I	R	V	P	D	N	Y	I	G	L	F	E	T	D	S	G	F	L	R	S	E	L
A	I	K	T	W	I	Q	L	A	K	E	A	G	C	A	Q	L	F	N	C	P	V	T	A	I	R	H	D	D	D
G	V	T	I	E	T	A	D	G	E	Y	Q	A	K	K	A	I	V	C	A	G	T	W	V	K	D	L	L	P	E
L	P	V	Q	P	V	R	K	V	F	A	W	Y	Q	A	D	G	R	Y	S	V	K	N	K	F	P	A	F	T	G
E	L	P	N	G	D	Q	Y	Y	G	F	P	A	E	N	D	A	L	K	I	G	K	H	N	G	G	Q	V	I	H
S	A	D	E	R	V	P	F	A	E	V	A	S	D	G	S	E	A	F	P	F	L	R	N	V	L	P	G	I	G
C	C	L	Y	G	A	A	C	T	Y	D	N	S	P	D	E	D	F	I	I	D	T	L	P	G	H	D	N	T	L
L	I	T	G	L	S	G	H	G	F	K	F	A	S	V	L	G	E	I	A	A	D	F	A	Q	D	K	K	S	D
F	D	L	T	P	F	R	L	S	R	F	Q																		
1				5					10					15					20					25					30

50 22.41% 99.0% >tr|A0A140NI62|A0A140NI62_ECOBD ECA polysaccharide chain length modulation protein OS=Escherichia coli (strain B / BL21-DE3) OX=469008 GN=wzzE PE=3 SV=1

M	T	Q	P	M	P	G	K	P	A	E	D	A	E	N	E	L	D	I	R	G	L	F	R	T	L	W	A	G	K
L	W	I	I	G	M	G	L	A	F	A	L	I	A	L	A	Y	T	F	F	A	R	Q	E	W	S	S	T	A	I
T	D	R	P	T	V	N	M	L	G	G	Y	Y	S	Q	Q	Q	F	L	R	N	L	D	V	R	S	N	M	A	S
A	D	Q	P	S	V	M	D	E	A	Y	K	E	F	V	M	Q	L	A	S	W	D	T	R	R	E	F	W	L	Q
T	D	Y	Y	K	Q	R	M	V	G	N	S	K	A	D	A	A	L	L	D	E	M	I	N	N	I	Q	F	I	P
G	D	F	T	R	A	V	N	D	S	V	K	L	I	A	E	T	A	P	D	A	N	N	L	L	R	Q	Y	V	A
F	A	S	Q	R	A	A	S	H	L	N	D	E	L	K	G	A	W	A	A	R	T	I	Q	M	K	A	Q	V	K
R	Q	E	E	V	A	K	A	I	Y	D	R	R	M	N	S	I	E	Q	A	L	K	I	A	E	Q	H	N	I	S
R	S	A	T	D	V	P	A	E	E	L	P	D	S	E	M	F	L	L	G	R	P	M	L	Q	A	R	L	E	N
L	Q	A	V	G	P	A	F	D	L	D	Y	D	Q	N	R	A	M	L	N	T	L	N	V	G	P	T	L	D	P
R	F	Q	T	Y	R	Y	L	R	T	P	E	E	P	V	K	R	D	S	P	R	R	A	F	L	M	I	M	W	G
I	V	G	G	L	I	G	A	G	V	A	L	T	R	R	C	S	K												
1				5					10					15					20					25					30

51 5.57% 99.0% >tr|A0A140NDI2|A0A140NDI2_ECOBD Beta-galactosidase OS=Escherichia coli (strain B / BL21-DE3) OX=469008 GN=lacZ PE=3 SV=1

M	T	M	I	T	D	S	L	A	V	V	L	Q	R	R	D	W	E	N	P	G	V	T	Q	L	N	R	L	A	A
H	P	P	F	A	S	W	R	N	S	E	E	A	R	T	D	R	P	S	Q	Q	L	R	S	L	N	G	E	W	R
F	A	W	F	P	A	P	E	A	V	P	E	S	W	L	E	C	D	L	P	E	A	D	T	V	V	V	P	S	N
W	Q	M	H	G	Y	D	A	P	I	Y	T	N	V	T	Y	P	I	T	V	N	P	P	F	V	P	T	E	N	P
T	G	C	Y	S	L	T	F	N	V	D	E	S	W	L	Q	E	G	Q	T	R	I	I	F	D	G	V	N	S	A
F	H	L	W	C	N	G	R	W	V	G	Y	G	Q	D	S	R	L	P	S	E	F	D	L	S	A	F	L	R	A
G	E	N	R	L	A	V	M	V	L	R	W	S	D	G	S	Y	L	E	D	Q	D	M	W	R	M	S	G	I	F
R	D	V	S	L	L	H	K	P	T	T	Q	I	S	D	F	H	V	A	T	R	F	N	D	D	F	S	R	A	V
L	E	A	E	V	Q	M	C	G	E	L	R	D	Y	L	R	V	T	V	S	L	W	Q	G	E	T	Q	V	A	S
G	T	A	P	F	G	G	E	I	I	D	E	R	G	G	Y	A	D	R	V	T	L	R	L	N	V	E	N	P	K
L	W	S	A	E	I	P	N	L	Y	R	A	V	V	E	L	H	T	A	D	G	T	L	I	E	A	E	A	C	D
V	G	F	R	E	V	R	I	E	N	G	L	L	L	L	N	G	K	P	L	L	I	R	G	V	N	R	H	E	H
H	P	L	H	G	Q	V	M	D	E	Q	T	M	V	Q	D	I	L	L	M	K	Q	N	N	F	N	A	V	R	C
S	H	Y	P	N	H	P	L	W	Y	T	L	C	D	R	Y	G	L	Y	V	V	D	E	A	N	I	E	T	H	G
M	V	P	M	N	R	L	T	D	D	P	R	W	L	P	A	M	S	E	R	V	T	R	M	V	Q	R	D	R	N
H	P	S	V	I	I	W	S	L	G	N	E	S	G	H	G	A	N	H	D	A	L	Y	R	W	I	K	S	V	D
P	S	R	P	V	Q	Y	E	G	G	G	A	D	T	T	A	T	D	I	I	C	P	M	Y	A	R	V	D	E	D
Q	P	F	P	A	V	P	K	W	S	I	K	K	W	L	S	L	P	G	E	T	R	P	L	I	L	C	E	Y	A
H	A	M	G	N	S	L	G	G	F	A	K	Y	W	Q	A	F	R	Q	Y	P	R	L	Q	G	G	F	V	W	D
W	V	D	Q	S	L	I	K	Y	D	E	N	G	N	P	W	S	A	Y	G	G	D	F	G	D	T	P	N	D	R
Q	F	C	M	N	G	L	V	F	A	D	R	T	P	H	P	A	L	T	E	A	K	H	Q	Q	Q	F	F	Q	F
R	L	S	G	Q	T	I	E	V	T	S	E	Y	L	F	R	H	S	D	N	E	L	L	H	W	M	V	A	L	D
G	K	P	L	A	S	G	E	V	P	L	D	V	A	P	Q	G	K	Q	L	I	E	L	P	E	L	P	Q	P	E
S	A	G	Q	L	W	L	T	V	R	V	V	Q	P	N	A	T	A	W	S	E	A	G	H	I	S	A	W	Q	Q
W	R	L	A	E	N	L	S	V	T	L	P	A	A	S	H	A	I	P	H	L	T	T	S	E	M	D	F	C	I
E	L	G	N	K	R	W	Q	F	N	R	Q	S	G	F	L	S	Q	M	W	I	G	D	K	K	Q	L	L	T	P
L	R	D	Q	F	T	R	A	P	L	D	N	D	I	G	V	S	E	A	T	R	I	D	P	N	A	W	V	E	R
W	K	A	A	G	H	Y	Q	A	E	A	A	L	L	Q	C	T	A	D	T	L	A	D	A	V	L	I	T	T	A
H	A	W	Q	H	Q	G	K	T	L	F	I	S	R	K	T	Y	R	I	D	G	S	G	Q	M	A	I	T	V	D
V	E	V	A	S	D	T	P	H	P	A	R	I	G	L	N	C	Q	L	A	Q	V	A	E	R	V	N	W	L	G
L	G	P	Q	E	N	Y	P	D	R	L	T	A	A	C	F	D	R	W	D	L	P	L	S	D	M	Y	T	P	Y
V	F	P	S	E	N	G	L	R	C	G	T	R	E	L	N	Y	G	P	H	Q	W	R	G	D	F	Q	F	N	I
S	R	Y	S	Q	Q	Q	L	M	E	T	S	H	R	H	L	L	H	A	E	E	G	T	W	L	N	I	D	G	F
H	M	G	I	G	G	D	D	S	W	S	P	S	V	S	A	E	F	Q	L	S	A	G	R	Y	H	Y	Q	L	V
W	C	Q	K																										
1				5					10					15					20					25					30

52 14.02% 99.0% >tr|A0A140N5T6|A0A140N5T6_ECOBD Erythronate-4-phosphate dehydrogenase OS=Escherichia coli (strain B / BL21-DE3) OX=469008 GN=pdxB PE=3 SV=1

M	K	I	L	V	D	E	N	M	P	Y	A	R	D	L	F	S	R	L	G	E	V	T	A	V	P	G	R	P	I
P	V	A	Q	L	A	D	A	D	A	L	M	V	R	S	V	T	K	V	N	E	S	L	L	A	G	K	P	I	K
F	V	G	T	A	T	A	G	T	D	H	V	D	E	A	W	L	K	Q	A	G	I	G	F	S	A	A	P	G	C
N	A	I	A	V	V	E	Y	V	F	S	S	L	L	M	L	A	E	R	D	G	F	S	L	Y	D	R	T	V	G

				I	V	G	V	G	N	V	G	R	R	L	Q	A	R	L	E	A	L	G	I	K	T	L	L	C	D	P	P	R	A
				D	R	G	D	E	G	D	F	R	S	L	D	E	L	V	Q	R	A	D	I	L	T	F	H	T	P	L	F	K	D
				G	P	Y	K	T	L	H	L	A	D	E	K	L	I	R	S	L	K	P	G	A	I	L	I	N	A	C	R	G	A
				V	V	D	N	T	A	L	L	T	C	L	N	E	G	Q	K	L	S	V	V	L	D	V	W	E	G	E	P	E	L
				N	V	E	L	L	K	K	V	D	I	G	T	P	H	I	A	G	Y	T	L	E	G	K	A	R	G	T	T	Q	V
				F	E	A	Y	S	K	F	I	G	H	E	Q	H	V	A	L	D	T	L	L	P	A	P	E	F	G	R	I	T	L
				H	G	P	L	D	Q	P	T	L	K	R	L	V	H	L	V	Y	D	V	R	R	D	D	A	P	L	R	K	V	A
				G	I	P	G	E	F	D	K	L	R	K	N	Y	L	E	R	R	E	W	S	S	L	Y	V	I	C	D	D	A	S
				A	A	S	L	L	C	K	L	G	F	N	A	V	H	H	P	A	R												
				1				5					10					15					20					25					30
53	11.97%	99.0%	>tr|A0A140NFM9|A0A140NFM9_ECOBD UDP-N-acetylglucosamine 2-epimerase OS=Escherichia coli (strain B / BL21-DE3) OX=469008 GN=wecB PE=3 SV=1																														
				M	K	V	L	T	V	F	G	T	R	P	E	A	I	K	M	A	P	L	V	H	A	L	A	K	D	P	F	F	E
				A	K	V	C	V	T	A	Q	H	R	E	M	L	D	Q	V	L	K	L	F	S	I	V	P	D	Y	D	L	N	I
				M	Q	P	G	Q	G	L	T	E	I	T	C	R	I	L	E	G	L	K	P	I	L	A	E	F	K	P	D	V	V
				L	V	H	G	D	T	T	T	T	L	A	T	S	L	A	A	F	Y	Q	R	I	P	V	G	H	V	E	A	G	L
				R	T	G	D	L	Y	S	P	W	P	E	E	A	N	R	T	L	T	G	H	L	A	M	Y	H	F	S	P	T	E
				T	S	R	Q	N	L	L	R	E	N	V	A	D	S	R	I	F	I	T	G	N	T	V	I	D	A	L	L	W	V
				R	D	Q	V	M	S	S	D	T	L	R	S	E	L	A	A	N	Y	P	F	I	D	P	D	K	K	M	I	L	V
				T	G	H	R	R	E	S	F	G	R	G	F	E	E	I	C	H	A	L	A	D	I	A	T	T	H	Q	D	I	Q
				I	V	Y	P	V	H	L	N	P	N	V	R	E	P	V	N	R	I	L	G	H	V	K	N	V	I	L	I	D	P
				Q	E	Y	L	P	F	V	W	L	M	N	H	A	W	L	I	L	T	D	S	G	G	I	Q	E	E	A	P	S	L
				G	K	P	V	L	V	M	R	D	T	T	E	R	P	E	A	V	T	A	G	T	V	R	L	V	G	T	D	K	Q
				R	I	V	E	E	V	T	R	L	L	K	D	E	N	E	Y	Q	A	M	S	R	A	H	N	P	Y	G	D	G	Q
				A	C	S	R	I	L	E	A	L	K	N	N	R	I	S	L														
				1				5					10					15					20					25					30
54	16.33%	99.0%	>tr|A0A140NDZ5|A0A140NDZ5_ECOBD Ribosomal RNA small subunit methyltransferase C OS=Escherichia coli (strain B / BL21-DE3) OX=469008 GN=rsmC PE=3 SV=1																														
				M	S	A	F	T	P	A	S	E	V	L	L	R	H	S	D	D	F	E	Q	S	R	I	L	F	A	G	D	L	Q
				D	D	L	P	A	R	L	D	T	A	A	S	R	A	H	T	Q	Q	F	H	H	W	Q	V	L	S	R	Q	M	G
				D	N	A	R	F	S	L	V	A	T	A	D	D	V	A	D	C	D	T	L	I	Y	Y	W	P	K	N	K	P	E
				A	Q	F	Q	L	M	N	L	L	S	L	L	P	V	G	T	D	I	F	V	V	G	E	N	R	S	G	V	R	S
				A	E	Q	M	L	A	D	Y	A	P	L	N	K	V	D	S	A	R	R	C	G	L	Y	F	G	R	L	E	K	Q
				P	V	F	D	A	E	K	F	W	G	E	Y	S	V	D	G	L	T	V	K	T	L	P	G	V	F	S	R	D	G
				L	D	V	G	S	Q	L	L	L	S	T	L	T	P	H	T	K	G	K	V	L	D	V	G	C	G	A	G	V	L
				S	V	A	F	A	R	H	S	P	K	I	R	L	T	L	C	D	V	S	A	P	A	V	E	A	S	R	A	T	L
				A	A	N	G	V	E	G	E	V	F	A	S	N	V	F	S	E	V	K	G	R	F	D	M	I	I	S	N	P	P
				F	H	D	G	M	Q	T	S	L	D	A	A	Q	T	L	I	R	G	A	V	R	H	L	N	S	G	G	E	L	R
				I	V	A	N	A	F	L	P	Y	P	D	V	L	D	E	T	F	G	F	H	E	V	I	A	Q	T	G	R	F	K
				V	Y	R	A	I	M	T	R	Q	A	K	K	G																	
				1				5					10					15					20					25					30
55	15.31%	99.0%	>tr|A0A140N9F5|A0A140N9F5_ECOBD Outer membrane protein assembly factor BamB OS=Escherichia coli (strain B / BL21-DE3) OX=469008 GN=bamB PE=3 SV=1																														
				M	Q	L	R	K	L	L	L	P	G	L	L	S	V	T	L	L	S	G	C	S	L	F	N	S	E	E	D	V	V
				K	M	S	P	L	P	T	V	E	N	Q	F	T	P	T	T	A	W	S	T	S	V	G	S	G	I	G	N	F	Y
				S	N	L	H	P	A	L	A	D	N	V	V	Y	A	A	D	R	A	G	L	V	K	A	L	N	A	D	D	G	K
				E	I	W	S	V	S	L	A	E	K	D	G	W	F	S	K	E	P	A	L	L	S	G	G	V	T	V	S	G	G
				H	V	Y	I	G	S	E	K	A	Q	V	Y	A	L	N	T	S	D	G	T	V	A	W	Q	T	K	V	A	G	E
				A	L	S	R	P	V	V	S	D	G	L	V	L	I	H	T	S	N	G	Q	L	Q	A	L	N	E	A	D	G	A
				V	K	W	T	V	N	L	D	M	P	S	L	S	L	R	G	E	S	A	P	A	T	A	F	G	A	A	V	V	G
				G	D	N	G	R	V	S	A	V	L	M	E	Q	G	Q	M	I	W	Q	Q	R	I	S	Q	A	T	G	S	T	E
				I	D	R	L	S	D	V	D	T	T	P	V	V	V	N	G	V	V	F	A	L	A	Y	N	G	N	L	T	A	L
				D	L	R	S	G	Q	I	M	W	K	R	E	L	G	S	V	N	D	F	I	V	D	G	N	R	I	Y	L	V	D
				Q	N	D	R	V	M	A	L	T	I	D	G	G	V	T	L	W	T	Q	S	D	L	L	H	R	L	L	T	S	P
				V	L	Y	N	G	N	L	V	V	G	D	S	E	G	Y	L	H	W	I	N	V	E	D	G	R	F	V	A	Q	Q
				K	V	D	S	S	G	F	Q	T	E	P	V	A	A	D	G	K	L	L	I	Q	A	K	D	G	T	V	Y	S	I
				T	R																												
				1				5					10					15					20					25					30
56	16.95%	99.0%	>tr|A0A140NF01|A0A140NF01_ECOBD Transcription termination factor Rho OS=Escherichia coli (strain B / BL21-DE3) OX=469008 GN=rho PE=3 SV=1																														
				M	N	L	T	E	L	K	N	T	P	V	S	E	L	I	T	L	G	E	N	M	G	L	E	N	L	A	R	M	R
				K	Q	D	I	I	F	A	I	L	K	Q	H	A	K	S	G	E	D	I	F	G	D	G	V	L	E	I	L	Q	D
				G	F	G	F	L	R	S	A	D	S	S	Y	L	A	G	P	D	D	I	Y	V	S	L	S	Q	I	R	R	F	N
				L	R	T	G	D	T	I	S	G	K	I	R	P	P	K	E	G	E	R	Y	F	A	L	L	K	V	N	E	V	N
				F	D	K	P	E	N	A	R	N	K	I	L	F	E	N	L	T	P	L	H	A	N	S	R	L	R	M	E	R	G
				N	G	S	T	E	D	L	T	A	R	V	L	D	L	A	S	P	I	G	R	G	Q	R	G	L	I	V	A	P	P
				K	A	G	K	T	M	L	L	Q	N	I	A	Q	S	I	A	Y	N	H	P	D	C	V	L	M	V	L	L	I	D
				E	R	P	E	E	V	T	E	M	Q	R	L	V	K	G	E	V	V	A	S	T	F	D	E	P	A	S	R	H	V
				Q	V	A	E	M	V	I	E	K	A	K	R	L	V	E	H	K	K	D	V	I	I	L	L	D	S	I	T	R	L
				A	R	A	Y	N	T	V	V	P	A	S	G	K	V	L	T	G	G	V	D	A	N	A	L	H	R	P	K	R	F
				F	G	A	A	R	N	V	E	E	G	G	S	L	T	I	I	A	T	A	L	I	D	T	G	S	K	M	D	E	V
				I	Y	E	E	F	K	G	T	G	N	M	E	L	H	L	S	R	K	I	A	E	K	R	V	F	P	A	I	D	Y
				N	R	S	G	T	R	K	E	E	L	L	T	T	Q	E	E	L	Q	K	M	W	I	L	R	K	I	I	H	P	M
				G	E	I	D	A	M	E	F	L	I	N	K	L	A	M	T	K	T	N	D	D	F	F	E	M	M	K	R	S	
				1				5					10					15					20					25					30
57	12.96%	99.0%	>tr|A0A140N6G0|A0A140N6G0_ECOBD Enolase OS=Escherichia coli (strain B / BL21-DE3) OX=469008 GN=eno PE=3 SV=1																														
				M	S	K	I	V	K	I	I	G	R	E	I	I	D	S	R	G	N	P	T	V	E	A	E	V	H	L	E	G	G
				F	V	G	M	A	A	A	P	S	G	A	S	T	G	S	R	E	A	L	E	L	R	D	G	D	K	S	R	F	L
				G	K	G	V	T	K	A	V	A	A	V	N	G	P	I	A	Q	A	L	I	G	K	D	A	K	D	Q	A	G	I
				D	K	I	M	I	D	L	D	G	T	E	N	K	S	K	F	G	A	N	A	I	L	A	V	S	L	A	N	A	K
				A	A	A	A	A	K	G	M	P	L	Y	E	H	I	A	E	L	N	G	T	P	G	K	Y	S	M	P	V	P	M
				M	N	I	I	N	G	G	E	H	A	D	N	N	V	D	I	Q	E	F	M	I	Q	P	V	G	A	K	T	V	K
				E	A	I	R	M	G	S	E	V	F	H	H	L	A	K	V	L	K	A	K	G	M	N	T	A	V	G	D	E	G
				G	Y	A	P	N	L	G	S	N	A	E	A	L	A	V	I	A	E	A	V	K	A	A	G	Y	E	L	G	K	D
				I	T	L	A	M	D	C	A	A	S	E	F	Y	K	D	G	K	Y	V	L	A	G	E	G	N	K	A	F	T	S
				E	E	F	T	H	F	L	E	E	L	T	K	Q	Y	P	I	V	S	I	E	D	G	L	D	E	S	D	W	D	G
				F	A	Y	Q	T	K	V	L	G	D	K	I	Q	L	V	G	D	D	L	F	V	T	N	T	K	I	L	K	E	G
				I	E	K	G	I	A	N	S	I	L	I	K	F	N	Q	I	G	S	L	T	E	T	L	A	A	I	K	M	A	K
				D	A	G	Y	T	A	V	I	S	H	R	S	G	E	T	E	D	A	T	I	A	D	L	A	V	G	T	A	A	G
				Q	I	K	T	G	S	M	S	R	S	D	R	V	A	K	Y	N	Q	L	I	R	I	E	E	A	L	G	E	K	A
				P	Y	N	G	R	K	E	I	K	G	Q	A																		
				1				5					10					15					20					25					30
58	15.50%	99.0%	>tr|A0A140N7M5|A0A140N7M5_ECOBD Acetate kinase OS=Escherichia coli (strain B / BL21-DE3) OX=469008 GN=ackA PE=3 SV=1																														
				M	S	S	K	L	V	L	V	L	N	C	G	S	S	S	L	K	F	A	I	I	D	A	V	N	G	E	E	Y	L
				S	G	L	A	E	C	F	H	L	P	E	A	R	I	K	W	K	M	D	G	N	K	Q	E	A	A	L	G	A	G
				A	A	H	S	E	A	L	N	F	I	V	N	T	I	L	A	Q	K	P	E	L	S	A	Q	L	T	A	I	G	H
				R	I	V	H	G	G	E	K	Y	T	S	S	V	V	I	D	E	S	V	I	Q	G	I	K	D	A	A	S	F	A
				P	L	H	N	P	A	H	L	I	G	I	E	E	A	L	K	S	F	P	Q	L	K	D	K	N	V	A	V	F	D
				T	A	F	H	Q	T	M	P	E	E	S	Y	L	Y	A	L	P	Y	N	L	Y	K	E	H	G	I	R	R	Y	G
				A	H	G	T	S	H	F	Y	V	T	Q	E	A	A	K	M	L	N	K	P	V	E	E	L	N	I	I	T	C	H
				L	G	N	G	G	S	V	S	A	I	R	N	G	K	C	V	D	T	S	M	G	L	T	P	L	E	G	L	V	M
				G	T	R	S	G	D	I	D	P	A	I	I	F	H	L	H	D	T	L	G	M	S	V	D	A	I	N	K	L	L
				T	K	E	S	G	L	L	G	L	T	E	V	T	S	D	C	R	Y	V	E	D	N	Y	A	T	K	E	D	A	K
				R	A	M	D	V	Y	C	H	R	L	A	K	Y	I	G	A	Y	T	A	L	M	D	G	R	L	D	A	V	V	F
				T	G	G	I	G	E	N	A	A	M	V	R	E	L	S	L	G	K	L	G	V	L	G	F	E	V	D	H	E	R
				N	L	A	A	R	F	G	K	S	G	F	I	N	K	E	G	T	R	P	A	V	V	I	P	T	N	E	E	L	V
				I	A	Q	D	A	S	R	L	T	A																				
				1				5					10					15					20					25					30
59	5.47%	99.0%	>tr|A0A140NH65|A0A140NH65_ECOBD 60 kDa chaperonin OS=Escherichia coli (strain B / BL21-DE3) OX=469008 GN=groL PE=3 SV=1																														
				M	A	A	K	D	V	K	F	G	N	D	A	R	V	K	M	L	R	G	V	N	V	L	A	D	A	V	K	V	T
				L	G	P	K	G	R	N	V	V	L	D	K	S	F	G	A	P	T	I	T	K	D	G	V	S	V	A	R	E	I
				E	L	E	D	K	F	E	N	M	G	A	Q	M	V	K	E	V	A	S	K	A	N	D	A	A	G	D	G	T	T
				T	A	T	V	L	A	Q	A	I	I	T	E	G	L	K	A	V	A	A	G	M	N	P	M	D	L	K	R	G	I
				D	K	A	V	T	A	A	V	E	E	L	K	A	L	S	V	P	C	S	D	S	K	A	I	A	Q	V	G	T	I
				S	A	N	S	D	E	T	V	G	K	L	I	A	E	A	M	D	K	V	G	K	E	G	V	I	T	V	E	D	G
				T	G	L	Q	D	E	L	D	V	V	E	G	M	Q	F	D	R	G	Y	L	S	P	Y	F	I	N	K	P	E	T
				G	A	V	E	L	E	S	P	F	I	L	L	A	D	K	K	I	S	N	I	R	E	M	L	P	V	L	E	A	V
				A	K	A	G	K	P	L	L	I	I	A	E	D	V	E	G	E	A	L	A	T	L	V	V	N	T	M	R	G	I
				V	K	V	A	A	V	K	A	P	G	F	G	D	R	R	K	A	M	L	Q	D	I	A	T	L	T	G	G	T	V
				I	S	E	E	I	G	M	E	L	E	K	A	T	L	E	D	L	G	Q	A	K	R	V	V	I	N	K	D	T	T
				T	I	I	D	G	V	G	E	E	A	A	I	Q	G	R	V	A	Q	I	R	Q	Q	I	E	E	A	T	S	D	Y
				D	R	E	K	L	Q	E	R	V	A	K	L	A	G	G	V	A	V	I	K	V	G	A	A	T	E	V	E	M	K
				E	K	K	A	R	V	E	D	A	L	H	A	T	R	A	A	V	E	E	G	V	V	A	G	G	G	V	A	L	I
				R	V	A	S	K	L	A	D	L	R	G	Q	N	E	D	Q	N	V	G	I	K	V	A	L	R	A	M	E	A	P
				L	R	Q	I	V	L	N	C	G	E	E	P	S	V	V	A	N	T	V	K	G	G	D	G	N	Y	G	Y	N	A
				A	T	E	E	Y	G	N	M	I	D	M	G	I	L	D	P	T	K	V	T	R	S	A	L	Q	Y	A	A	S	V
				A	G	L	M	I	T	T	E	C	M	V	T	D	L	P	K	N	D	A	A	D	L	G	A	A	G	G	M	G	G
				M	G	G	M	G	G	M	M																						
				1				5					10					15					20					25					30
60	10.25%	98.3%	>tr|A0A140N8V0|A0A140N8V0_ECOBD dTDP-glucose 46-dehydratase OS=Escherichia coli (strain B / BL21-DE3) OX=469008 GN=ECBD_1614 PE=3 SV=1																														
				M	K	I	L	V	T	G	G	A	G	F	I	G	S	A	V	V	R	H	I	I	N	N	T	Q	D	S	V	V	N
				V	D	K	L	T	Y	A	G	N	L	E	S	L	A	E	I	S	D	S	E	R	Y	S	F	E	H	A	D	I	C
				D	A	E	A	M	A	R	I	F	A	Q	H	Q	P	D	A	V	M	H	L	A	A	E	S	H	V	D	R	S	I
				T	G	P	A	A	F	I	E	T	N	I	V	G	T	Y	V	L	L	E	A	A	R	N	Y	W	S	G	L	D	D
				E	K	K	K	N	F	R	F	H	H	I	S	T	D	E	V	Y	G	D	L	P	H	P	D	E	V	N	S	N	E
				T	L	Q	L	F	T	E	T	T	A	Y	A	P	S	S	P	Y	S	A	S	K	A	S	S	D	H	L	V	R	A
				W	K	R	T	Y	G	L	P	T	I	V	T	N	C	S	N	N	Y	G	P	Y	H	F	P	E	K	L	I	P	L

			V I L N A L E G K A L P I Y G K G D Q I R D W L Y V E D H A
			R A L Y T V V T E G K A G E T Y N I G G H N E K K N I D V V
			F T I C D L L D E I V P K E K S Y R E Q I T Y V A D R P G H
			D R R Y A I D A D K I S R E L G W K P Q E T F E S G I R K T
			V E W Y L A N T N W V E N V K S G A Y Q S W I E Q N Y E G R
			Q
			1 5 10 15 20 25 30
61	8.68%	99.0%	>tr|C5W8R2|C5W8R2_ECOBD Ybl130 OS=Escherichia coli (strain B / BL21-DE3) GN=ybl130 PE=4 SV=1
			M F F R D M I K K R R G D T I K D S N N K K Q P D K S E K V
			N I V S K T P K L A E K D K K K N I T N N L A K N E P K M I
			T T N I R L F T K I N H L I H T G E G I N C S F H I E Q W I
			G E F I A S N E R F A L L I R D I N L Y N W A I T Q Y P N V
			D I V Y A K S A I A V E Q V L N Q L P Y L K A I Y Y L S N T
			G N L I H T L R Y N I Y Q H I F L G H G D S D K A A S A H K
			F F R V Y D E I W V A G Q A H I D R F K N A G F N V E H I K
			F V K V G R P S L K Q I V K Q S V S N N N V T E H I I K T L
			T Y F P T W E G A F E E N N Y S S A R L S P I F L S E L Q Q
			K L N C K I V A K Y H P L T G S R D K T L E T I Q D A L Q N
			S F I D R S C M L S I A N K S E P I P N L V V N S D L F I C
			D I S A V V S E C I S S L S P I F I Y I P E D R N V I T S G
			S D M H Y S D Y A Y T F S N I E Q L M Q L I E N I I I N G N
			D H L A E A R Y K A L D Y L L S V N E T V N D A F L S E L K
			R I S S T V S E K E N K R E F V I Q
			1 5 10 15 20 25 30
62	9.09%	99.0%	>tr|A0A140NE28|A0A140NE28_ECOBD Glycoside hydrolase family 4 OS=Escherichia coli (strain B / BL21-DE3) OX=469008 GN=ECBD_3912 PE=3 SV=1
			M M T A P K I T F I G A G S T I F V K N I L G D V F H R E A
			L K T A H I A L M D I D P T R L E E S H I V V R K L M D S A
			G A S G K I T C H T Q Q K E A L Q D A D F V V V A F Q I G G
			Y E P C T V T D F E V C K R H G L E Q T I A D T L G P G G I
			M R A L R T I P H L W Q I C E D M T E V C P D A T M L N Y V
			N P M A M N T W A M Y A R Y P H I K Q V G L C H S V Q G T A
			E E L A R D L N I D P A T L R Y R C A G I N H M A F Y L E L
			E R K T A D G S Y V N L Y P E L L A A H D A G Q A P K P N I
			H G N T R C Q N I V R Y E M F K K L G Y F V T E S S E H F A
			E Y T P W F I K P G R E D L I E R Y K V P L D E Y P K R C V
			E Q L A N W H K E L E E Y K N A S R I D I K P S R E Y A S T
			I M N A I W T G E P S V I Y G N V R N D G L I D N L P Q G C
			C V E V A C L V D A N G I Q P T K V G T L P S H L A A L M Q
			T N I N V Q T L L T E A I L T E N R D R V Y H A A M M D P H
			T A A V L G I D E I Y A L V D D L I A A H G D W L P G W L H
			R
			1 5 10 15 20 25 30
63	8.90%	99.0%	>tr|A0A140NC10|A0A140NC10_ECOBD Citrate synthase OS=Escherichia coli (strain B / BL21-DE3) OX=469008 GN=ECBD_2941 PE=3 SV=1
			M A D T K A K L T L N G D T A V E L D V L K G T L G Q D V I
			D I R T L G S K G V F T F D P G F T S T A S C E S K I T F I
			D G D E G I L L H R G F P I D Q L A T D S N Y L E V C Y I L
			L N G E K P T Q E Q Y D E F K T T V T R H T M I H E Q I T R
			L F H A F R R D S H P M A V M C G I T G A L A A F Y H D S L
			D V N N P R H R E I A A F R L L S K M P T M A A M C Y K Y S
			I G Q P F V Y P R N D L S Y A G N F L N M M F S T P C E P Y
			E V N P I L E R A M D R I L I L H A D H E Q N A S T S T V R
			T A G S S G A N P F A C I A A G I A S L W G P A H G G A N E
			A A L K M L E E I S S V K H I P E F V R R A K D K N D S F R
			L M G F G H R V Y K N Y D P R A T V M R E T C H E V L K E L
			G T K D D L L E V A M E L E N I A L N D P Y F I E K K L Y P
			N V D F Y S G I I L K A M G I P S S M F T V I F A M A R T V
			G W I A H W S E M H S D G M K I A R P R Q L Y T G Y E K R D
			F K S D I K R
			1 5 10 15 20 25 30
64	13.82%	99.0%	>tr|A0A140NAQ0|A0A140NAQ0_ECOBD Iron-sulfur cluster carrier protein OS=Escherichia coli (strain B / BL21-DE3) OX=469008 GN=ECBD_1545 PE=3 SV=1
			M N E Q S Q A K S P E A L R A M V A G T L A N F Q H P T L K
			H N L T T L K A L H H V A W M D D T L H V E L V M P F V W H
			S A F E E L K E Q C S A E L L R I T G A K A I D W K L S H N
			I A T L K R V K N Q P G I N G V K N I I A V S S G K G G V G
			K S S T A V N L A L A L A A E G A K V G I L D A D I Y G P S
			I P T M L G A E N Q R P T S P D G T H M A P I M S H G L A T
			N S I G Y L V T D D N A M V W R G P M A S K A L M Q M L Q E
			T L W P D L D Y L V L D M P P G T G D I Q L T L A Q N I P V
			T G A V V V T T P Q D I A L I D A K K G I V M F E K V E V P
			V L G I V E N M S V H I C S N C G H H E P I F G T G G A E K
			L A E K Y H T Q L L G Q M P L H I S L R E D L D K G T P T V
			I S R P E S E F T A I Y R Q L A D R V A A Q L Y W Q G E V I
			P G E I S F R A V
			1 5 10 15 20 25 30
65	12.18%	99.0%	>tr|A0A140N8E4|A0A140N8E4_ECOBD UPF0283 membrane protein YcjF OS=Escherichia coli (strain B / BL21-DE3) OX=469008 GN=ycjF PE=3 SV=1
			M T E P L K P R I D F D G P L E V D Q N P K F R A Q Q T F D
			E N Q A Q N F A P A T L D E A Q E E E G Q V E A V M D A A L
			R P K R S L W R K M V M G G L A L F G A S V V G Q G V Q W T
			M N A W Q T Q D W V A L G G C A A G A L I I G A G V G S V V
			T E W R R L W R L R Q R A H E R D E A R D L L H S H G T G K
			G R A F C E K L A Q Q A G I D Q S H P A L Q R W Y A S I H E
			T Q N D R E V V S L Y A H L V Q P V L D A Q A R R E I S R S
			A A E S T L M I A V S P L A L V D M A F I A W R N L R L I N
			R I A T L Y G I E L G Y Y S R L R L F K L V L L N I A F A G
			A S E L V R E V G M D W M S Q D L A A R L S T R A A Q G I G
			A G L L T A R L G I K A M E L C R P L P W I D D D K P R L G
			D F R R Q L I G Q V K E T L Q K G K T P S E K
			1 5 10 15 20 25 30
66	10.95%	99.0%	>tr|A0A140N6E5|A0A140N6E5_ECOBD D-tagatose-16-bisphosphate aldolase subunit GatZ OS=Escherichia coli (strain B / BL21-DE3) OX=469008 GN=gatZ PE=3 SV=1
			M K T L I A R H K A G E H I G I C S V C S A H P L V I E A A
			L A F D R N S T R K V L I E A T S N Q V N Q F G G Y T G M T
			P A D F R E F V F T I A D K V G F A R E R I I L G G D H L G
			P N C W Q Q E N A D A A M E K S V E L V K A Y V R A G F S K
			I H L D A S M S C A G D P I P L A P E T V A E R A A V L C F
			A A E S V A T D C Q R E Q L S Y V I G T E V P V P G G E A S
			A I Q S V H I T Q V E D A A N T L R T H Q K A F I A R G L T
			E A L T R V I A I V V Q P G V E F D H S N I I H Y Q P Q E A
			Q P L A Q W I E N T R M V Y E A H S T D Y Q T R A A Y W E L
			V R D H F A I L K V G P A L T F A L R E A I F A L A Q I E Q
			E L T A P E N R S G C L A V I E E V M L D E P Q Y W K K Y Y
			R T G F N D S L L D I R Y S L S D R I R Y Y W P H S R I K N
			S V E T M M L N L E G V D I P L G M I S Q Y L P K Q F E R I
			Q S G E L S A I P H Q L I M D K I Y D V L R A Y R Y G C A E
			1 5 10 15 20 25 30
67	10.92%	99.0%	>tr|A0A140ND46|A0A140ND46_ECOBD Dihydroorotase OS=Escherichia coli (strain B / BL21-DE3) OX=469008 GN=pyrC PE=3 SV=1
			M T A P S Q V L K I R R P D D W H L H L R D G D M L K T V V
			P Y T S E I Y G R A I V M P N L A P P V T T V E A A V A Y R
			Q R I L D A V P A G H D F T P L M T C Y L T D S L D P N E L
			E R G F N E G V F T A A K L Y P A N A T T N S S H G V T S V
			D A I M P V L E R M E K I G M P L L V H G E V T H A D I D I
			F D R E A R F I E S V M E P L R Q R L T A L K V V F E H I T
			T K D A A D Y V R D G N E R L A A T I T P Q H L M F N R N H
			M L V G G V R P H L Y C L P I L K R N I H Q Q A L R E L V A
			S G F N R V F L G T D S A P H A R H R K E S S C G C A G C F
			N A P T A L G S Y A T V F E E M N A L Q H F E A F C S V N G
			P Q F Y G L P V N D T F I E L V R E E Q Q V A E S I A L T D
			D T L V P F L A G E T V R W S V K Q
			1 5 10 15 20 25 30
68	13.60%	99.0%	>tr|A0A140N7T1|A0A140N7T1_ECOBD Succinyl-diaminopimelate desuccinylase OS=Escherichia coli (strain B / BL21-DE3) OX=469008 GN=dapE PE=3 SV=1
			M S C P V I E L T Q Q L I R R P S L S P D D A G C Q A L L I
			E R L Q A I G F T V E R M D F A D T Q N F W A W R G Q G E T
			L A F A G H T D V V P P G D A D R W I N P P F E P T I R D G
			M L F G R G A A D M K G S L A A M V V A A E R F V A Q H P N
			H T G R L A F L I T S D E E A S A H N G T V K V V E A L M A
			R N E R L D Y C L V G E P S S I E V V G D V V K N G R R G S
			L T C N L T I H G V Q G H V A Y P H L A D N P V H R A A P F
			L N E L V A I E W D Q G N E F F P A T S M Q I A N I Q A G T
			G S N N V I P G E L F V Q F N F R F S T E L T D E M I K A Q
			V L A L L E K H Q L R Y T V D W W L S G Q P F L T A R G K L
			V D A V V N A V E H Y N E I K P Q L L T T G G T S D G R F I
			A R M G A Q V V E L G P V N A T I H K I N E C V N A A D L Q
			L L A R M Y Q R I M E Q L V A

				1				5					10					15					20					25					30
69	11.85%	99.0%	>tr|A0A140N3Y7|A0A140N3Y7_ECOBD Aldo/keto reductase OS=Escherichia coli (strain B / BL21-DE3) OX=469008 GN=ECBD_0890 PE=4 SV=1																														
				M	Q	Y	H	R	I	P	H	S	S	L	E	V	S	T	L	G	L	G	T	M	T	F	G	E	Q	N	S	E	A
				D	A	H	A	Q	L	D	Y	A	V	A	Q	G	I	N	L	I	D	V	A	E	M	Y	P	V	P	P	R	P	E
				T	Q	G	L	T	E	T	Y	V	G	N	W	L	A	K	H	G	S	R	E	K	L	I	I	A	S	K	V	S	G
				P	S	R	N	N	D	K	G	I	R	P	D	Q	A	L	D	R	K	N	I	R	E	A	L	H	D	S	L	K	R
				L	Q	T	D	Y	L	D	L	Y	Q	V	H	W	P	Q	R	P	T	N	C	F	G	K	L	G	Y	S	W	T	D
				S	A	P	A	V	S	L	L	D	T	L	D	A	L	A	E	Y	Q	R	A	G	K	I	R	Y	I	G	V	S	N
				E	T	A	F	G	V	M	R	Y	L	H	L	A	D	K	H	D	L	P	R	I	V	T	I	Q	N	P	Y	S	L
				L	N	R	S	F	E	V	G	L	A	E	V	S	Q	Y	E	G	V	E	L	L	A	Y	S	C	L	G	F	G	T
				L	T	G	K	Y	L	N	G	A	K	P	A	G	A	R	N	T	L	F	S	R	F	T	R	Y	S	G	E	Q	T
				Q	K	A	V	A	A	Y	V	D	I	A	R	R	H	G	L	D	P	A	Q	M	A	L	A	F	V	R	R	Q	P
				F	V	A	S	T	L	L	G	A	T	T	M	D	Q	L	K	T	N	I	E	S	L	H	L	E	L	S	E	D	V
				L	A	E	I	E	A	V	H	Q	V	Y	T	Y	P	A	P														
				1				5					10					15					20					25					30
70	6.64%	99.0%	>tr|A0A140NBA5|A0A140NBA5_ECOBD 30S ribosomal protein S1 OS=Escherichia coli (strain B / BL21-DE3) OX=469008 GN=ECBD_2684 PE=3 SV=1																														
				M	T	E	S	F	A	Q	L	F	E	E	S	L	K	E	I	E	T	R	P	G	S	I	V	R	G	V	V	V	A
				I	D	K	D	V	V	L	V	D	A	G	L	K	S	E	S	A	I	P	A	E	Q	F	K	N	A	Q	G	E	L
				E	I	Q	V	G	D	E	V	D	V	A	L	D	A	V	E	D	G	F	G	E	T	L	L	S	R	E	K	A	K
				R	H	E	A	W	I	T	L	E	K	A	Y	E	D	A	E	T	V	T	G	V	I	N	G	K	V	K	G	G	F
				T	V	E	L	N	G	I	R	A	F	L	P	G	S	L	V	D	V	R	P	V	R	D	T	L	H	L	E	G	K
				E	L	E	F	K	V	I	K	L	D	Q	K	R	N	N	V	V	V	S	R	R	A	V	I	E	S	E	N	S	A
				E	R	D	Q	L	L	E	N	L	Q	E	G	M	E	V	K	G	I	V	K	N	L	T	D	Y	G	A	F	V	D
				L	G	G	V	D	G	L	L	H	I	T	D	M	A	W	K	R	V	K	H	P	S	E	I	V	N	V	G	D	E
				I	T	V	K	V	L	K	F	D	R	E	R	T	R	V	S	L	G	L	K	Q	L	G	E	D	P	W	V	A	I
				A	K	R	Y	P	E	G	T	K	L	T	G	R	V	T	N	L	T	D	Y	G	C	F	V	E	I	E	E	G	V
				E	G	L	V	H	V	S	E	M	D	W	T	N	K	N	I	H	P	S	K	V	V	N	V	G	D	V	V	E	V
				M	V	L	D	I	D	E	E	R	R	R	I	S	L	G	L	K	Q	C	K	A	N	P	W	Q	Q	F	A	E	T
				H	N	K	G	D	R	V	E	G	K	I	K	S	I	T	D	F	G	I	F	I	G	L	D	G	G	I	D	G	L
				V	H	L	S	D	I	S	W	N	V	A	G	E	E	A	V	R	E	Y	K	K	G	D	E	I	A	A	V	V	L
				Q	V	D	A	E	R	E	R	I	S	L	G	V	K	Q	L	A	E	D	P	F	N	N	W	V	A	L	N	K	K
				G	A	I	V	T	G	K	V	T	A	V	D	A	K	G	A	T	V	E	L	A	D	G	V	E	G	Y	L	R	A
				S	E	A	S	R	D	R	V	E	D	A	T	L	V	L	S	V	G	D	E	V	E	A	K	F	T	G	V	D	R
				K	N	R	A	I	S	L	S	V	R	A	K	D	E	A	D	E	K	D	A	I	A	T	V	N	K	Q	E	D	A
				N	F	S	N	N	A	M	A	E	A	F	K	A	A	K	G	E													
				1				5					10					15					20					25					30
71	15.92%	99.0%	>tr|D5RE94|D5RE94_FUSN2 Acetyl-CoA C-acetyltransferase OS=Fusobacterium nucleatum subsp. nucleatum (strain ATCC 23726 / VPI 4351) OX=525283 GN=atoB PE=3 SV=1																														
				M	S	K	V	Y	V	V	A	A	K	R	S	A	I	G	S	F	L	G	T	L	S	P	L	K	P	G	D	L	G
				A	Q	I	V	K	N	I	L	E	E	T	K	V	D	P	A	N	I	D	E	V	I	V	G	N	V	L	S	A	G
				Q	A	Q	G	V	G	R	Q	V	A	I	R	A	G	I	P	Y	E	V	P	A	Y	S	V	N	I	I	C	G	S
				G	M	K	S	V	I	T	A	F	S	N	I	K	A	G	E	A	D	L	V	I	A	G	G	T	E	S	M	S	G
				A	G	F	I	L	P	G	A	I	R	G	G	H	K	M	A	D	L	T	M	K	D	H	M	I	L	D	A	L	T
				D	A	Y	H	N	I	H	M	G	I	T	A	E	N	I	A	E	R	Y	G	I	T	R	E	E	Q	D	A	F	A
				L	D	S	Q	L	K	A	I	A	A	V	D	S	G	R	F	K	D	E	I	A	P	V	V	I	P	N	K	K	G
				D	I	I	F	D	T	D	E	Y	P	N	R	K	T	D	A	E	K	L	A	K	L	K	P	A	F	K	K	D	G
				S	V	T	A	G	N	A	S	G	L	N	D	G	A	S	F	L	M	L	A	S	E	E	A	V	K	K	Y	N	L
				K	P	L	V	E	I	V	A	T	G	T	G	G	V	D	P	L	V	M	G	M	G	P	V	P	A	I	R	K	A
				F	N	K	T	D	L	K	L	K	D	M	E	L	I	E	L	N	E	A	F	A	A	Q	S	L	G	V	I	K	E
				L	C	K	E	H	G	V	T	P	E	W	I	K	E	R	T	N	V	N	G	G	A	I	A	L	G	H	P	V	G
				A	S	G	N	R	I	T	V	T	L	I	Y	E	M	K	K	R	G	V	E	Y	G	L	A	S	L	C	I	G	G
				G	M	G	T	A	L	I	L	K	N	V	K																		
				1				5					10					15					20					25					30
72	15.27%	99.0%	>tr|A0A140NGB9|A0A140NGB9_ECOBD Ornithine carbamoyltransferase OS=Escherichia coli (strain B / BL21-DE3) OX=469008 GN=argI PE=3 SV=1																														
				M	S	G	F	Y	H	K	H	F	L	K	L	L	D	F	T	P	A	E	L	N	S	L	L	Q	L	A	A	K	L
				K	A	D	K	K	S	G	K	E	E	A	K	L	T	G	K	N	I	A	L	I	F	E	K	D	S	T	R	T	R
				C	S	F	E	V	A	A	Y	D	Q	G	A	R	V	T	Y	L	G	P	S	G	S	Q	I	G	H	K	E	S	I
				K	D	T	A	R	V	L	G	R	M	Y	D	G	I	Q	Y	R	G	Y	G	Q	E	I	V	E	T	L	A	E	Y
				A	G	V	P	V	W	N	G	L	T	N	E	F	H	P	T	Q	L	L	A	D	L	L	T	M	Q	E	H	L	P
				G	K	A	F	N	E	M	T	L	V	Y	A	G	D	A	R	N	N	M	G	N	S	M	L	E	A	A	A	L	T
				G	L	D	L	R	L	V	A	P	Q	A	C	W	P	E	A	A	L	V	T	E	C	R	A	L	A	Q	Q	N	G
				G	N	I	T	L	T	E	D	V	A	K	G	V	E	G	A	D	F	I	Y	T	D	V	W	V	S	M	G	E	A
				K	E	K	W	A	E	R	I	A	L	L	R	D	Y	Q	V	N	S	K	M	M	Q	L	T	G	N	P	E	V	K
				F	L	H	C	L	P	A	F	H	D	D	Q	T	T	L	G	K	K	M	A	E	E	F	G	L	H	G	G	M	E
				V	T	D	E	V	F	E	S	A	A	S	I	V	F	D	Q	A	E	N	R	M	H	T	I	K	A	V	M	V	A
				T	L	S	K																										
				1				5					10					15					20					25					30
73	17.47%	99.0%	>tr|A0A140N2J1|A0A140N2J1_ECOBD 33 kDa chaperonin OS=Escherichia coli (strain B / BL21-DE3) OX=469008 GN=hslO PE=3 SV=1																														
				M	P	Q	H	D	Q	L	H	R	Y	L	F	E	N	F	A	V	R	G	E	L	V	T	V	S	E	T	L	Q	Q
				I	L	E	N	H	D	Y	P	Q	P	V	K	N	V	L	A	E	L	L	V	A	T	S	L	L	T	A	T	L	K
				F	D	G	D	I	T	V	Q	L	Q	G	D	G	P	M	N	L	A	V	I	N	G	N	N	N	Q	Q	M	R	G
				V	A	R	V	Q	G	E	I	P	E	N	A	D	L	K	T	L	V	G	N	G	Y	V	V	I	T	I	T	P	S
				E	G	E	R	Y	Q	G	V	V	G	L	E	G	D	T	L	A	A	C	L	E	D	Y	F	M	R	S	E	Q	L
				P	T	R	L	F	I	R	T	G	D	V	D	G	K	P	A	A	G	G	M	L	L	Q	V	M	P	A	Q	N	A
				Q	Q	D	D	F	D	H	L	A	T	L	T	E	T	I	K	T	E	E	L	L	T	L	P	A	N	E	V	L	W
				R	L	Y	H	E	E	E	V	T	V	Y	D	P	Q	D	V	E	F	K	C	T	C	S	R	E	R	C	A	D	A
				L	K	T	L	P	D	E	E	V	D	S	I	L	A	E	D	G	E	I	D	M	H	C	D	Y	C	G	N	H	Y
				L	F	N	A	M	D	I	A	E	I	R	N	N	A	S	P	A	D	P	Q	V	H								
				1				5					10					15					20					25					30
74	12.12%	99.0%	>tr|A0A140NFJ6|A0A140NFJ6_ECOBD Nicotinate-nucleotide pyrophosphorylase OS=Escherichia coli (strain B / BL21-DE3) OX=469008 GN=ECBD_3510 PE=3 SV=1																														
				M	P	P	R	R	Y	N	P	D	T	R	R	D	E	L	L	E	R	I	N	L	D	I	P	G	A	V	A	Q	A
				L	R	E	D	L	G	G	T	V	D	A	N	N	D	I	T	A	K	L	L	P	E	N	S	R	S	H	A	T	V
				I	T	R	E	N	G	V	F	C	G	K	R	W	V	E	E	V	F	I	Q	L	A	G	D	D	V	T	I	I	W
				H	V	D	D	G	D	V	I	N	A	N	Q	S	L	F	E	L	E	G	P	S	R	V	L	L	T	G	E	R	T
				A	L	N	F	V	Q	T	L	S	G	V	A	S	K	V	R	H	Y	V	E	L	L	E	G	T	N	T	Q	L	L
				D	T	R	K	T	L	P	G	L	R	S	A	L	K	Y	A	V	L	C	G	G	G	A	N	H	R	L	G	L	S
				D	A	F	L	I	K	E	N	H	I	I	A	S	G	S	V	R	Q	A	V	E	K	A	S	W	L	H	P	D	A
				P	V	E	V	E	V	E	N	L	E	E	L	D	E	A	L	K	A	G	A	D	I	I	M	L	D	N	F	E	T
				E	Q	M	R	E	A	V	K	R	T	N	G	K	A	L	L	E	V	S	G	N	V	T	D	K	T	L	R	E	F
				A	E	T	G	V	D	F	I	S	V	G	A	L	T	K	H	V	Q	A	L	D	L	S	M	R	F	R			
				1				5					10					15					20					25					30
75	5.37%	99.0%	>tr|A0A140N6A5|A0A140N6A5_ECOBD Chaperone protein ClpB OS=Escherichia coli (strain B / BL21-DE3) OX=469008 GN=clpB PE=3 SV=1																														
				M	R	L	D	R	L	T	N	K	F	Q	L	A	L	A	D	A	Q	S	L	A	L	G	H	D	N	Q	F	I	E
				P	L	H	L	M	S	A	L	L	N	Q	E	G	G	S	V	S	P	L	L	T	S	A	G	I	N	A	G	Q	L
				R	T	D	I	N	Q	A	L	N	R	L	P	Q	V	E	G	T	G	G	D	V	Q	P	S	Q	D	L	V	R	V
				L	N	L	C	D	K	L	A	Q	K	R	G	D	N	F	I	S	S	E	L	F	V	L	A	A	L	E	S	R	G
				T	L	A	D	I	L	K	A	A	G	A	T	T	A	N	I	T	Q	A	I	E	Q	M	R	G	G	E	S	V	N
				D	Q	G	A	E	D	Q	R	Q	A	L	K	K	Y	T	I	D	L	T	E	R	A	E	Q	G	K	L	D	P	V
				I	G	R	D	E	E	I	R	R	T	I	Q	V	L	Q	R	R	T	K	N	N	P	V	L	I	G	E	P	G	V
				G	K	T	A	I	V	E	G	L	A	Q	R	I	I	N	G	E	V	P	E	G	L	K	G	R	R	V	L	A	L
				D	M	G	A	L	V	A	G	A	K	Y	R	G	E	F	E	E	R	L	K	G	V	L	N	D	L	A	K	Q	E
				G	N	V	I	L	F	I	D	E	L	H	T	M	V	G	A	G	K	A	D	G	A	M	D	A	G	N	M	L	K
				P	A	L	A	R	G	E	L	H	C	V	G	A	T	T	L	D	E	Y	R	Q	Y	I	E	K	D	A	A	L	E
				R	R	F	Q	K	V	F	V	A	E	P	S	V	E	D	T	I	A	I	L	R	G	L	K	E	R	Y	E	L	H
				H	H	V	Q	I	T	D	P	A	I	V	A	A	A	T	L	S	H	R	Y	I	A	D	R	Q	L	P	D	K	A
				I	D	L	I	D	E	A	A	S	S	I	R	M	Q	I	D	S	K	P	E	E	L	D	R	L	D	R	R	I	I
				Q	L	K	L	E	Q	Q	A	L	M	K	E	S	D	E	A	S	K	K	R	L	D	M	L	N	E	E	L	S	D
				K	E	R	Q	Y	S	E	L	E	E	E	W	K	A	E	K	A	S	L	S	G	T	Q	T	I	K	A	E	L	E
				Q	A	K	I	A	I	E	Q	A	R	R	V	G	D	L	A	R	M	S	E	L	Q	Y	G	K	I	P	E	L	E
				K	Q	L	E	A	A	T	Q	L	E	G	K	T	M	R	L	L	R	N	K	V	T	D	A	E	I	A	E	V	L
				A	R	W	T	G	I	P	V	S	R	M	M	E	S	E	R	E	K	L	L	R	M	E	Q	E	L	H	H	R	V
				I	G	Q	N	E	A	V	D	A	V	S	N	A	I	R	R	S	R	A	G	L	A	D	P	N	R	P	I	G	S
				F	L	F	L	G	P	T	G	V	G	K	T	E	L	C	K	A	L	A	N	F	M	F	D	S	D	E	A	M	V
				R	I	D	M	S	E	F	M	E	K	H	S	V	S	R	L	V	G	A	P	P	G	Y	V	G	Y	E	E	G	G
				Y	L	T	E	A	V	R	R	R	P	Y	S	V	I	L	L	D	E	V	E	K	A	H	P	D	V	F	N	I	L
				L	Q	V	L	D	D	G	R	L	T	D	G	Q	G	R	T	V	D	F	R	N	T	V	V	I	M	T	S	N	L
				G	S	D	L	I	Q	E	R	F	G	E	L	D	Y	A	H	M	K	E	L	V	L	G	V	V	S	H	N	F	R
				P	E	F	I	N	R	I	D	E	V	V	V	F	H	P	L	G	E	Q	H	I	A	S	I	A	Q	I	Q	L	K
				R	L	Y	K	R	L	E	E	R	G	Y	E	I	H	I	S	D	E	A	L	K	L	L	S	E	N	G	Y	D	P
				V	Y	G	A	R	P	L	K	R	A	I	Q	Q	Q	I	E	N	P	L	A	Q	Q	I	L	S	G	E	L	V	P
				G	K	V	I	R	L	E	V	N	E	D	R	I	V	A	V	Q													
				1				5					10					15					20					25					30
76	4.83%	98.3%	>tr|A0A140N7C7|A0A140N7C7_ECOBD Elongation factor G OS=Escherichia coli (strain B / BL21-DE3) OX=469008 GN=fusA PE=3 SV=1																														
				M	A	R	T	T	P	I	A	R	Y	R	N	I	G	I	S	A	H	I	D	A	G	K	T	T	T	T	E	R	I
				L	F	Y	T	G	V	N	H	K	I	G	E	V	H	D	G	A	A	T	M	D	W	M	E	Q	E	Q	E	R	G
				I	T	I	T	S	A	A	T	T	A	F	W	S	G	M	A	K	Q	Y	E	P	H	R	I	N	I	I	D	T	P
				G	H	V	D	F	T	I	E	V	E	R	S	M	R	V	L	D	G	A	V	M	V	Y	C	A	V	G	G	V	Q
				P	Q	S	E	T	V	W	R	Q	A	N	K	Y	K	V	P	R	I	A	F	V	N	K	M	D	R	M	G	A	N
				F	L	K	V	V	N	Q	I	K	T	R	L	G	A	N	P	V	P	L	Q	L	A	I	G	A	E	E	H	F	T
				G	V	V	D	L	V	K	M	K	A	I	N	W	N	D	A	D	Q	G	V	T	F	E	Y	E	D	I	P	A	D
				M	V	E	L	A	N	E	W	H	Q	N	L	I	E	S	A	A	E	A	S	E	E	L	M	E	K	Y	L	G	G
				E	E	L	T	E	A	E	I	K	G	A	L	R	Q	R	V	L	N	N	E	I	I	L	V	T	C	G	S	A	F
				K	N	K	G	V	Q	A	M	L	D	A	V	I	D	Y	L	P	S	P	V	D	V	P	A	I	N	G	I	L	D
				D	G	K	D	T	P	A	E	R	H	A	S	D	D	E	P	F	S	A	L	A	F	K	I	A	T	D	P	F	V

				G N L T F F R V Y S G V V N S G D T V L N S V K A A R E R F
				G R I V Q M H A N K R E E I K E V R A G D I A A A I G L K D
				V T T G D T L C D P D A P I I L E R M E F P E P V I S I A V
				E P K T K A D Q E K M G L A L G R L A K E D P S F R V W T D
				E E S N Q T I I A G M G E L H L D I I V D R M K R E F N V E
				A N V G K P Q V A Y R E T I R Q K V T D V E G K H A K Q S G
				G R G Q Y G H V V I D M Y P L E P G S N P K G Y E F I N D I
				K G G V I P G E Y I P A V D K G I Q E Q L K A G P L A G Y P
				V V D M G I R L H F G S Y H D V D S S E L A F K L A A S I A
				F K E G F K K A K P V L L E P I M K V E V E T P E E N T G D
				V I G D L S R R R G M L K G Q E S E V T G V K I H A E V P L
				S E M F G Y A T Q L R S L T K G R A S Y T M E F L K Y D E A
				P S N V A Q A V I E A R G K
				1 5 10 15 20 25 30
77	11.31%	99.0%	>tr|A0A140SSA1|A0A140SSA1_ECOBD HemY protein OS=Escherichia coli (strain B / BL21-DE3) OX=469008 GN=ECBD_4240 PE=4 SV=1	
				M L K V L L L F V L L I A G I V V G P M I A G H Q G Y V L I
				Q T D N Y N I E T S V T G L A I I L I L A M V V L F A I E W
				L L R R I F R T G A H T R G W F V G R K R R R A R K Q T E Q
				A L L K L A E G D Y Q Q V E K L M A K N A D H A E Q P V V N
				Y L L A A E A A Q Q R G D E A R A N Q H L E R A A E L A G N
				D T I P V E I T R V R L Q L A R N E N H A A R H G V D K L L
				E V T P R H P E V L R L A E Q A Y I R T G A W S S L L D I I
				P S M A K A H V G D E E H R A M L E Q Q A W I G L M D Q A R
				A D N G S E G L R N W W K N Q S R K T R H Q V A L Q V A M A
				E H L I E C D D H D T A Q Q I I I D G L K R Q Y D D R L L L
				P I P R L K T N N P E Q L E K V L R Q Q I K N V G D R P L L
				W S T L G Q S L M K H G E W Q E A S L A F R A A L K Q R P D
				A Y D Y A W L A D A L D R L H K P E E A A A M R R D G L M L
				T L Q N N P P Q
				1 5 10 15 20 25 30
78	8.61%	98.8%	>tr|Q072Q4|Q072Q4_ECOBD Uncharacterized protein OS=Escherichia coli (strain B / BL21-DE3) OX=469008 PE=4 SV=1	
				M F F R D M I K K R R G D T I K D S N N K K Q P D K S E K V
				N I V S K T P K L A E K D K K K N I T N N L A K N E P K M I
				T T N I R L F T K I N H L I H T G G G I N C S F H I E Q W I
				G E F I A S N E R F A L L I R D I N L Y N W A I T Q Y P N V
				D I V Y A K S A I A V E Q V L N Q L P Y L K A I Y Y L S N T
				G N L I H T L R Y N I Y Q H I F L G H G D S D K A A S A H K
				F F R V Y D E I W V A G Q A H I D R F K N A G F N V E H I K
				F V K V G R P S L K Q I V K Q S V S N N N V T E H I I K T L
				T Y F P T W E G A F E E N N Y S S A R L S P I F L S E L R Q
				K L N C K I V A K Y H P L T G S R D K T L E T I Q D A L Q I
				I S
				1 5 10 15 20 25 30
79	9.14%	98.9%	>tr|A0A140N836|A0A140N836_ECOBD Chorismate synthase OS=Escherichia coli (strain B / BL21-DE3) OX=469008 GN=aroC PE=3 SV=1	
				M A G N T I G Q L F R V T T F G E S H G L A L G C I V D G V
				P P G I P L T E A D L Q H D L D R R R P G T S R Y T T Q R R
				E P D Q V K I L S G V F E G V T T G T S I G L L I E N T D Q
				R S Q D Y S A I K D V F R P G H A D Y T Y E Q K Y G L R D Y
				R G G G R S S A R E T A M R V A A G A I A K K Y L A E K F G
				I E I R G C L T Q M G D I P L E I K D W S Q V E Q N P F F C
				P D P D K I D A L D E L M R A L K K E G D S I G A K V T V V
				A S G V P A G L G E P V F D R L D A D I A H A L M S I N A V
				K G V E I G D G F D V V A L R G S Q N R D E I T K D G F Q S
				N H A G G I L G G I S S G Q Q I I A H M A L K P T S S I T V
				P G R T I N R F G E E V E M I T K G R H D P C V G I R A V P
				I A E A M L A I V L M D H L L R Q R A Q N A D V K T D I P R
				W
				1 5 10 15 20 25 30
80	5.33%	98.9%	>tr|A0A140SSA2|A0A140SSA2_ECOBD Glutamine synthetase OS=Escherichia coli (strain B / BL21-DE3) OX=469008 GN=ECBD_4157 PE=3 SV=1	
				M S A E H V L T M L N E H E V K F V D L R F T D T K G K E Q
				H V T I P A H Q V N A E F F E E G K M F D G S S I G G W K G
				I N E S D M V L M P D A S T A V I D P F F A D S T L I I R C
				D I L E P G T L Q G Y D R D P R S I A K R A E D Y L R S T G
				I A D T V L F G P E P E F F L F D D I R F G S S I S G S H V
				A I D D I E G A W N S S T Q Y E G G N K G H R P A V K G G Y
				F P V P P V D S A Q D I R S E M C L V M E Q M G L V V E A H
				H H E V A T A G Q N E V A T R F N T M T K K A D E I Q I Y K
				Y V V H N V A H R F G K T A T F M P K P M F G D N G S G M H
				C H M S L S K N G V N L F A G D K Y A G L S E Q A L Y Y I G
				G V I K H A K A I N A L A N P T T N S Y K R L V P G Y E A P
				V M L A Y S A R N R S A S I R I P V V S S P K A R R I E V R
				F P D P A A N P Y L C F A A L L M A G L D G I K N K I H P G
				E A M D K N L Y D L P P E E A K E I P Q V A G S L E E A L N
				E L D L D R E F L K A G G V F T D E A I D A Y I A L R R E E
				D D R V R M T P H P V E F E L Y Y S V
				1 5 10 15 20 25 30
81	3.79%	97.6%	>tr|C5WA02|C5WA02_ECOBD Glycerol 3-phosphate dehydrogenaseaerobic OS=Escherichia coli (strain B / BL21-DE3) GN=glpD PE=3 SV=1	
				M E T K D L I V I G G G I N G A G I A A D A A G R G L S V L
				M L E A Q D L A C A T S S A S S K L I H G G L R Y L E H Y E
				F R L V S E A L A E R E V L L K M A P H I A F P M R F R L P
				H R P H L R P A W M I R I G L F M Y D H L G K R T S L P G S
				T G L R F G A N S V L K P E I K R G F E Y S D C W V D D A R
				L V L A N A Q M V V R K G G E V L T R T R A T S A R R E N G
				L W I V E A E D I D T G K K Y S W Q A R G L V N A T G P W V
				K Q F F D D G M H L P S P Y G I R L I K G S H I V V P R V H
				T Q K Q A Y I L Q N E D K R I V F V I P W M D E F S I I G T
				T D V E Y K G D P K A V K I E E S E I N Y L L N V Y N T H F
				K K Q L S R D D I V W T Y S G V R P L C D D E S D S P Q A I
				T R D Y T L D I H D E N G K A P L L S V F G G K L T T Y R K
				L A E H A L E K L T P Y Y Q G I G P A W T K E S V L P G G A
				I E G D R D D Y A A R L R R R Y P F L T E S L A R H Y A R T
				Y G S N S E L L L G N A G T V S D L G E D F G H E F Y E A E
				L K Y L V D H E W V R R A D D A L W R R T K Q G M W L N A D
				Q Q S R V S Q W L V E Y T Q Q K L S L A S
				1 5 10 15 20 25 30
82	7.61%	97.5%	>tr|A0A140NAY3|A0A140NAY3_ECOBD Histidine biosynthesis bifunctional protein HisB OS=Escherichia coli (strain B / BL21-DE3) OX=469008 GN=hisB PE=3 SV=1	
				M S Q K Y L F I D R D G T L I S E P P S D F Q V D R F D K L
				A F E P G V I P E L L K L Q K A G Y K L V M I T N Q D G L G
				T Q S F P Q A D F D G P H N L M M Q I F T S Q G V Q F D E V
				L I C P H L P A D E C D C R K P K V K L V E R Y L A E Q A M
				D R A N S Y V I G D R A T D I Q L A E N M G I N G L R Y D R
				E I L N W P M I G E Q L T K R D R Y A H V V R N T K E T Q I
				D V Q V W L D R E G G S K I N T G V G F F D H M L D Q I A T
				H G G F R M E I N V K G D L Y I D D H H T V E D T G L A L G
				E A L K I A L G D K R G I C R F G F V L P M D E C L A R C A
				L D I S G R P H L E Y K A E F T Y Q R V G D L S T E M I E H
				F F R S L S Y T M G V T L H L K T K G K N D H H R V E S L F
				K A F G R T L R Q A I R V E G D T L P S S K G V L
				1 5 10 15 20 25 30
83	8.65%	97.5%	>tr|A0A140N6I5|A0A140N6I5_ECOBD Selenidewater dikinase OS=Escherichia coli (strain B / BL21-DE3) OX=469008 GN=selD PE=3 SV=1	
				M S E N S I R L T Q Y S H G A G C G C K I S P K V L E T I L
				H S E Q A K F V D P N L L V G N E T R D D A A V Y D L G N G
				T S V I S T T D F F M P I V D N P F D F G R I A A T N A I S
				D I F A M G G K P I M A I A I L G W P I N K L S P E I A R E
				V T E G G R Y A C R Q A G I A L A G G H S I D A P E P I F G
				L A V T G I V P T E R V K K N S T A Q A G C K L F L T K P L
				G I G V L T T A E K K S L L K P D H Q G L A T E V M C R M N
				I A G A S F A N I E G V K A M T D V T G F G L L G H L S E M
				C Q G A G V Q A R V D Y E A I P K L P G V E E Y I K L G A V
				P G G T E R N F A S Y G H L M G E M P R E V R D L L C D P Q
				T S G G L L L A V M P E A E N E V K A T A A E F G I E L T A
				I G E L V P A R G G R A M V E I R
				1 5 10 15 20 25 30
84	3.79%	97.6%	>tr|A0A140N6N1|A0A140N6N1_ECOBD Glycerol-3-phosphate dehydrogenase OS=Escherichia coli (strain B / BL21-DE3) OX=469008 GN=ECBD_0316 PE=3 SV=1	
				M E T K D L I V I G G G I N G A G I A A D A A G R G L S V L
				M L E A Q D L A C A T S S A S S K L I H G G L R Y L E H Y E
				F R L V S E A L A E R E V L L K M A P H I A F P M R F R L P
				H R P H L R P A W M I R I G L F M Y D H L G K R T S L P G S
				T G L R F G A N S V L K P E I K R G F E Y S D C W V D D A R
				L V L A N A Q M V V R K G G E V L T R T R A T S A R R E N G

				L	W	I	V	E	A	E	D	I	D	T	G	K	K	Y	S	W	Q	A	R	G	L	V	N	A	T	G	P	W	V
				K	Q	F	F	D	D	G	M	H	L	P	S	P	Y	G	I	R	L	I	K	G	S	H	I	V	V	P	R	V	H
				T	Q	K	Q	A	Y	I	L	Q	N	E	D	K	R	I	V	F	V	I	P	W	M	D	E	F	S	I	I	G	T
				T	D	V	E	Y	K	G	D	P	K	A	V	K	I	E	E	S	E	I	N	Y	L	L	N	V	Y	N	T	H	F
				K	K	Q	L	S	R	D	D	I	V	W	T	Y	S	G	V	R	P	L	C	D	D	E	S	D	S	P	Q	A	I
				T	R	D	Y	T	L	D	I	H	D	E	N	G	K	A	P	L	L	S	V	F	G	G	K	L	T	T	Y	R	K
				L	A	E	H	A	L	E	K	L	T	P	Y	Y	Q	G	I	G	P	A	W	T	K	E	S	V	L	P	G	G	A
				I	E	G	D	R	D	D	Y	A	A	R	L	R	R	R	Y	P	F	L	T	E	S	L	A	R	H	Y	A	R	T
				Y	G	S	N	S	E	L	L	L	G	N	A	G	T	V	S	D	L	G	E	D	F	G	H	E	F	Y	E	A	E
				L	K	Y	L	V	D	H	E	W	V	R	R	A	D	D	A	L	W	R	R	T	K	Q	G	M	W	L	N	A	D
				Q	Q	S	R	V	S	Q	W	L	V	E	Y	T	Q	Q	R	L	S	L	A	S									
				1				5					10					15					20					25					30
85	8.96%	99.0%	>tr|R9RA39|R9RA39_FUSNU Glyceraldehyde-3-phosphate dehydrogenase OS=Fusobacterium nucleatum subsp. animalis 4_8 OX=469607 GN=HMPREF0409_00492 PE=3 SV=1																														
				M	A	V	K	V	A	I	N	G	F	G	R	I	G	R	L	A	L	R	V	M	S	K	N	K	D	F	D	V	V
				A	I	N	D	L	T	D	A	K	T	L	A	H	L	F	K	Y	D	S	A	Q	G	R	F	D	G	T	I	E	V
				T	D	D	G	F	I	V	N	G	D	S	I	K	V	F	A	K	A	N	P	E	E	L	P	W	G	D	L	G	V
				D	V	V	L	E	C	T	G	F	F	T	S	K	E	K	A	E	A	H	I	K	A	G	A	K	K	V	V	I	S
				A	P	A	T	G	D	L	K	T	V	V	Y	N	V	N	D	N	I	L	D	G	T	E	T	V	I	S	G	A	S
				C	T	T	N	C	L	A	P	M	A	K	V	L	N	D	K	F	G	I	V	E	G	L	M	T	T	I	H	A	Y
				T	N	D	Q	N	T	L	D	A	P	H	K	K	G	D	L	R	R	A	R	A	A	A	E	N	I	V	P	N	T
				T	G	A	A	K	A	I	G	L	V	I	P	E	L	K	G	K	L	D	G	A	A	Q	R	V	P	V	I	T	G
				S	I	T	E	L	V	T	V	L	G	K	D	V	T	V	D	E	V	N	A	A	M	K	A	A	S	N	E	S	F
				G	Y	T	E	E	P	L	V	S	S	D	I	I	G	I	S	F	G	S	L	F	D	A	T	Q	T	K	V	L	T
				V	D	G	K	Q	L	V	K	T	V	A	W	Y	D	N	E	M	S	Y	T	S	Q	L	I	R	T	L	K	K	F
				V	E	I	S	K																									
				1				5					10					15					20					25					30
86	9.46%	99.0%	>tr|A0A140N964|A0A140N964_ECOBD tRNA pseudouridine synthase D OS=Escherichia coli (strain B / BL21-DE3) OX=469008 GN=truD PE=3 SV=1																														
				M	I	E	F	D	N	L	T	Y	L	H	G	K	P	Q	G	T	G	L	L	K	A	N	P	E	D	F	V	V	V
				E	D	L	G	F	E	P	D	G	E	G	E	H	I	L	V	R	I	L	K	N	G	C	N	T	R	F	V	A	D
				A	L	A	K	F	L	K	I	H	A	R	E	V	S	F	A	G	Q	K	D	K	H	A	V	T	E	Q	W	L	C
				A	R	V	P	G	K	E	M	P	D	L	S	A	F	Q	L	E	G	C	Q	V	L	E	Y	A	R	H	K	R	K
				L	R	L	G	A	L	K	G	N	A	F	T	L	V	L	R	E	V	S	N	R	D	D	V	E	Q	R	L	I	D
				I	C	V	K	G	V	P	N	Y	F	G	A	Q	R	F	G	I	G	G	S	N	L	Q	G	A	Q	R	W	A	Q
				T	N	T	P	V	R	D	R	N	K	R	S	F	W	L	S	A	A	R	S	A	L	F	N	Q	I	V	A	E	R
				L	K	K	A	D	V	N	Q	V	V	D	G	D	A	L	Q	L	A	G	R	G	S	W	F	V	A	T	T	E	E
				L	A	E	L	Q	R	R	V	N	D	K	E	L	M	I	T	A	A	L	P	G	S	G	E	W	G	T	Q	R	E
				A	L	A	F	E	Q	A	A	V	A	A	E	T	E	L	Q	A	L	L	V	R	E	K	V	E	A	A	R	R	A
				M	L	L	Y	P	Q	Q	L	S	W	N	W	W	D	D	V	T	V	E	I	R	F	W	L	P	A	G	S	F	A
				T	S	V	V	R	E	L	I	N	T	T	G	D	Y	A	H	I	A	E											
				1				5					10					15					20					25					30
87	5.35%	98.4%	>tr|A0A140NE14|A0A140NE14_ECOBD Lysine--tRNA ligase OS=Escherichia coli (strain B / BL21-DE3) OX=469008 GN=lysS PE=3 SV=1																														
				M	S	E	Q	E	T	R	G	A	N	E	A	I	D	F	N	D	E	L	R	N	R	R	E	K	L	A	A	L	R
				Q	Q	G	V	A	F	P	N	D	F	R	R	D	H	T	S	E	Q	L	H	E	E	F	D	A	K	D	N	Q	E
				L	E	S	L	N	I	E	V	S	V	A	G	R	M	M	T	R	R	I	M	G	K	A	S	F	V	T	L	Q	D
				V	G	G	R	I	Q	L	Y	V	A	R	D	S	L	P	E	G	V	Y	N	D	Q	F	K	K	W	D	L	G	D
				I	I	G	A	R	G	T	L	F	K	T	Q	T	G	E	L	S	I	H	C	T	E	L	R	L	L	T	K	A	L
				R	P	L	P	D	K	F	H	G	L	Q	D	Q	E	V	R	Y	R	Q	R	Y	L	D	L	I	A	N	D	K	S
				R	Q	T	F	V	V	R	S	K	I	L	A	A	I	R	Q	F	M	V	A	R	G	F	M	E	V	E	T	P	M
				M	Q	V	I	P	G	G	A	S	A	R	P	F	I	T	H	H	N	A	L	D	L	D	M	Y	L	R	I	A	P
				E	L	Y	L	K	R	L	V	V	G	G	F	E	R	V	F	E	I	N	R	N	F	R	N	E	G	I	S	V	R
				H	N	P	E	F	T	M	M	E	L	Y	M	A	Y	A	D	Y	H	D	L	I	E	L	T	E	S	L	F	R	T
				L	A	Q	E	V	L	G	T	T	K	V	T	Y	G	E	H	V	F	D	F	G	K	P	F	E	K	L	T	M	R
				E	A	I	K	K	Y	R	P	E	T	D	M	A	D	L	D	N	F	D	A	A	K	A	L	A	E	S	I	G	I
				T	V	E	K	S	W	G	L	G	R	I	V	T	E	I	F	D	E	V	A	E	A	H	L	I	Q	P	T	F	I
				T	E	Y	P	A	E	V	S	P	L	A	R	R	N	D	V	N	P	E	I	T	D	R	F	E	F	F	I	G	G
				R	E	I	G	N	G	F	S	E	L	N	D	A	E	D	Q	A	E	R	F	Q	E	Q	V	N	A	K	A	A	G
				D	D	E	A	M	F	Y	D	E	D	Y	V	T	A	L	E	Y	G	L	P	P	T	A	G	L	G	I	G	I	D
				R	M	I	M	L	F	T	N	S	H	T	I	R	D	V	I	L	F	P	A	M	R	P	Q	K					
				1				5					10					15					20					25					30
88	10.94%	98.8%	>tr|A0A140NBY5|A0A140NBY5_ECOBD 4-hydroxythreonine-4-phosphate dehydrogenase OS=Escherichia coli (strain B / BL21-DE3) OX=469008 GN=pdxA PE=3 SV=1																														
				M	V	K	T	Q	R	V	V	I	T	P	G	E	P	A	G	I	G	P	D	L	V	V	Q	L	A	Q	R	E	W
				P	V	E	L	V	V	C	A	D	A	T	L	L	T	N	R	A	A	M	L	G	L	P	L	T	L	R	P	Y	S
				P	N	S	P	A	Q	P	Q	T	A	G	T	L	T	L	L	P	V	A	L	R	A	P	V	T	A	G	Q	L	A
				V	E	N	G	H	Y	V	V	E	T	L	A	R	A	C	D	G	C	L	N	G	E	F	A	A	L	I	T	G	P
				V	H	K	G	V	I	N	D	A	G	I	P	F	T	G	H	T	E	F	F	E	E	R	S	Q	A	K	K	V	V
				M	M	L	A	T	E	E	L	R	V	A	L	A	T	T	H	L	P	L	R	D	I	A	D	A	I	T	P	A	L
				L	H	E	V	I	A	I	L	H	H	D	L	R	T	K	F	G	I	A	E	P	R	I	L	V	C	G	L	N	P
				H	A	G	E	G	G	H	M	G	T	E	E	I	D	T	I	I	P	V	L	N	E	L	R	A	Q	G	M	K	L
				N	G	P	L	P	A	D	T	L	F	Q	P	K	Y	L	D	N	A	D	A	V	L	A	M	Y	H	D	Q	G	L
				P	V	L	K	Y	Q	G	F	G	R	G	V	N	I	T	L	G	L	P	F	I	R	T	S	V	D	H	G	T	A
				L	E	L	A	G	R	G	K	A	D	V	G	S	F	I	T	A	L	N	L	A	I	K	M	I	V	N	T	Q	
				1				5					10					15					20					25					30
89	4.33%	99.0%	>tr|A0A140ND61|A0A140ND61_ECOBD Chaperone protein HtpG OS=Escherichia coli (strain B / BL21-DE3) OX=469008 GN=htpG PE=3 SV=1																														
				M	K	G	Q	E	T	R	G	F	Q	S	E	V	K	Q	L	L	H	L	M	I	H	S	L	Y	S	N	K	E	I
				F	L	R	E	L	I	S	N	A	S	D	A	A	D	K	L	R	F	R	A	L	S	N	P	D	L	Y	E	G	D
				G	E	L	R	V	R	V	S	F	D	K	D	K	R	T	L	T	I	S	D	N	G	V	G	M	T	R	D	E	V
				I	D	H	L	G	T	I	A	K	S	G	T	K	S	F	L	E	S	L	G	S	D	Q	A	K	D	S	Q	L	I
				G	Q	F	G	V	G	F	Y	S	A	F	I	V	A	D	K	V	T	V	R	T	R	A	A	G	E	K	P	E	N
				G	V	F	W	E	S	A	G	E	G	E	Y	T	V	A	D	I	T	K	E	D	R	G	T	E	I	T	L	H	L
				R	E	G	E	D	E	F	L	D	D	W	R	V	R	S	I	I	S	K	Y	S	D	H	I	A	L	P	V	E	I
				E	K	R	E	E	K	D	G	E	T	V	I	S	W	E	K	I	N	K	A	Q	A	L	W	T	R	N	K	S	E
				I	T	D	E	E	Y	K	E	F	Y	K	H	I	A	H	D	F	N	D	P	L	T	W	S	H	N	R	V	E	G
				K	Q	E	Y	T	S	L	L	Y	I	P	S	Q	A	P	W	D	M	W	N	R	D	H	K	H	G	L	K	L	Y
				V	Q	R	V	F	I	M	D	D	A	E	Q	F	M	P	N	Y	L	R	F	V	R	G	L	I	D	S	S	D	L
				P	L	N	V	S	R	E	I	L	Q	D	S	T	V	T	R	N	L	R	N	A	L	T	K	R	V	L	Q	M	L
				E	K	L	A	K	D	D	A	E	K	Y	Q	T	F	W	Q	Q	F	G	L	V	L	K	E	G	P	A	E	D	F
				A	N	Q	E	A	I	A	K	L	L	R	F	A	S	T	H	T	D	S	S	A	Q	T	V	S	L	E	D	Y	V
				S	R	M	K	E	G	Q	E	K	I	Y	Y	I	T	A	D	S	Y	A	A	A	K	S	S	P	H	L	E	L	L
				R	K	K	G	I	E	V	L	L	L	S	D	R	I	D	E	W	M	M	N	Y	L	T	E	F	D	G	K	P	F
				Q	S	V	S	K	V	D	E	S	L	E	K	L	A	D	E	V	D	E	S	A	K	E	A	E	K	A	L	T	P
				F	I	D	R	V	K	A	L	L	G	E	R	V	K	D	V	R	L	T	H	R	L	T	D	T	P	A	I	V	S
				T	D	A	D	E	M	S	T	Q	M	A	K	L	F	A	A	A	G	Q	K	V	P	E	V	K	Y	I	F	E	L
				N	P	D	H	V	L	V	K	R	A	A	D	T	E	D	E	A	K	F	S	E	W	V	E	L	L	L	D	Q	A
				L	L	A	E	R	G	T	L	E	D	P	N	L	F	I	R	R	M	N	Q	L	L	V	S						
				1				5					10					15					20					25					30
90	5.26%	99.0%	>tr|A0A140ND72|A0A140ND72_ECOBD ATP synthase subunit alpha OS=Escherichia coli (strain B / BL21-DE3) OX=469008 GN=atpA PE=3 SV=1																														
				M	Q	L	N	S	T	E	I	S	E	L	I	K	Q	R	I	A	Q	F	N	V	V	S	E	A	H	N	E	G	T
				I	V	S	V	S	D	G	V	I	R	I	H	G	L	A	D	C	M	Q	G	E	M	I	S	L	P	G	N	R	Y
				A	I	A	L	N	L	E	R	D	S	V	G	A	V	V	M	G	P	Y	A	D	L	A	E	G	M	K	V	K	C
				T	G	R	I	L	E	V	P	V	G	R	G	L	L	G	R	V	V	N	T	L	G	A	P	I	D	G	K	G	P
				L	D	H	D	G	F	S	A	V	E	A	I	A	P	G	V	I	E	R	Q	S	V	D	Q	P	V	Q	T	G	Y
				K	A	V	D	S	M	I	P	I	G	R	G	Q	R	E	L	I	I	G	D	R	Q	T	G	K	T	A	L	A	I
				D	A	I	I	N	Q	R	D	S	G	I	K	C	I	Y	V	A	I	G	Q	K	A	S	T	I	S	N	V	V	R
				K	L	E	E	H	G	A	L	A	N	T	I	V	V	V	A	T	A	S	E	S	A	A	L	Q	Y	L	A	P	Y
				A	G	C	A	M	G	E	Y	F	R	D	R	G	E	D	A	L	I	I	Y	D	D	L	S	K	Q	A	V	A	Y
				R	Q	I	S	L	L	L	R	R	P	P	G	R	E	A	F	P	G	D	V	F	Y	L	H	S	R	L	L	E	R
				A	A	R	V	N	A	E	Y	V	E	A	F	T	K	G	E	V	K	G	K	T	G	S	L	T	A	L	P	I	I
				E	T	Q	A	G	D	V	S	A	F	V	P	T	N	V	I	S	I	T	D	G	Q	I	F	L	E	T	N	L	F
				N	A	G	I	R	P	A	V	N	P	G	I	S	V	S	R	V	G	G	A	A	Q	T	K	I	M	K	K	L	S
				G	G	I	R	T	A	L	A	Q	Y	R	E	L	A	A	F	S	Q	F	A	S	D	L	D	D	A	T	R	K	Q
				L	D	H	G	Q	K	V	T	E	L	L	K	Q	K	Q	Y	A	P	M	S	V	A	Q	Q	S	L	V	L	F	A
				A	E	R	G	Y	L	A	D	V	E	L	S	K	I	G	S	F	E	A	A	L	L	A	Y	V	D	R	D	H	A
				P	L	M	Q	E	I	N	Q	T	G	G	Y	N	D	E	I	E	G	K	L	K	G	I	L	D	S	F	K	A	T
				Q	S	W																											
				1				5					10					15					20					25					30

91	8.29%	99.0%	>tr|A0A140N784|A0A140N784_ECOBD 3-dehydroquinate synthase OS=Escherichia coli (strain B / BL21-DE3) OX=469008 GN=aroB PE=3 SV=1																														
				M	E	R	I	V	V	T	L	G	E	R	S	Y	P	I	T	I	A	S	G	L	F	N	E	P	A	S	F	L	P
				L	K	S	G	E	Q	V	M	L	V	T	N	E	T	L	A	P	L	Y	L	D	K	V	R	G	V	L	E	Q	A
				G	V	N	V	D	S	V	I	L	P	D	G	E	Q	Y	K	S	L	A	V	L	D	T	V	F	T	A	L	L	Q
				K	P	H	G	R	D	T	T	L	V	A	L	G	G	G	V	V	G	D	L	T	G	F	A	A	A	S	Y	Q	R
				G	V	R	F	I	Q	V	P	T	T	L	L	S	Q	V	D	S	S	V	G	G	K	T	A	V	N	H	P	L	G
				K	N	M	I	G	A	F	Y	Q	P	A	S	V	V	V	D	L	D	C	L	K	T	L	P	P	R	E	L	A	S
				G	L	A	E	V	I	K	Y	G	I	I	L	D	G	A	F	F	N	W	L	E	E	N	L	D	A	L	L	R	L
				D	G	P	A	M	A	Y	C	I	R	R	C	C	E	L	K	A	E	V	V	A	A	D	E	R	E	T	G	L	R
				A	L	L	N	L	G	H	T	F	G	H	A	I	E	A	E	M	G	Y	G	N	W	L	H	G	E	A	V	A	A
				G	M	V	M	A	A	R	T	S	E	R	L	G	Q	F	S	S	A	E	T	Q	R	I	I	T	L	L	K	R	A
				G	L	P	V	N	G	P	R	E	M	S	A	Q	A	Y	L	P	H	M	L	R	D	K	K	V	L	A	G	E	I
				R	L	I	L	P	L	A	I	G	K	S	E	V	R	S	G	V	S	H	E	L	V	L	N	A	I	A	D	C	Q
				S	A																												
				1				5					10					15					20					25					30
92	8.48%	99.0%	>tr|A0A140ND59|A0A140ND59_ECOBD Aspartate--ammonia ligase OS=Escherichia coli (strain B / BL21-DE3) OX=469008 GN=asnA PE=3 SV=1																														
				M	K	T	A	Y	I	A	K	Q	R	Q	I	S	F	V	K	S	H	F	S	R	Q	L	E	E	R	L	G	L	I
				E	V	Q	A	P	I	L	S	R	V	G	D	G	T	Q	D	N	L	S	G	C	E	K	A	V	Q	V	K	V	K
				A	L	P	D	A	Q	F	E	V	V	H	S	L	A	K	W	K	R	Q	T	L	G	Q	H	D	F	S	A	G	E
				G	L	Y	T	H	M	K	A	L	R	P	D	E	D	R	L	S	P	L	H	S	V	Y	V	D	Q	W	D	W	E
				R	V	M	G	D	G	E	R	Q	F	S	T	L	K	S	T	V	E	A	I	W	A	G	I	K	A	T	E	A	A
				V	S	E	E	F	G	L	A	P	F	L	P	D	Q	I	H	F	V	H	S	Q	E	L	L	S	R	Y	P	D	L
				D	A	K	G	R	E	R	A	I	A	K	D	L	G	A	V	F	L	V	G	I	G	G	K	L	S	D	G	H	C
				H	D	V	R	A	P	D	Y	D	D	W	S	T	P	S	E	L	G	H	A	G	L	N	G	D	I	L	V	W	N
				P	V	L	E	D	A	F	E	L	S	S	M	G	I	R	V	D	A	D	T	L	K	H	Q	L	A	L	T	G	D
				E	D	R	L	Q	L	E	W	H	Q	A	L	L	R	G	E	M	P	Q	T	I	G	G	G	I	G	Q	S	R	L
				T	M	L	L	L	Q	L	P	H	I	G	Q	V	Q	C	G	V	W	P	A	A	V	R	E	N	V	P	S	L	L
				1				5					10					15					20					25					30
93	9.42%	99.0%	>tr|A0A140N9W3|A0A140N9W3_ECOBD Malate/L-lactate dehydrogenase OS=Escherichia coli (strain B / BL21-DE3) OX=469008 GN=ECBD_2822 PE=3 SV=1																														
				M	E	S	G	H	R	F	D	A	Q	T	L	H	S	F	I	Q	A	V	F	R	Q	M	G	S	E	E	Q	E	A
				K	L	V	A	D	H	L	I	A	A	N	L	A	G	H	D	S	H	G	I	G	M	I	P	S	Y	V	R	S	W
				S	Q	G	H	L	Q	I	N	H	H	A	K	T	V	K	E	A	G	A	A	V	T	L	D	G	D	R	A	F	G
				Q	V	A	A	H	E	A	M	A	L	G	I	E	K	A	H	Q	H	G	I	A	A	V	A	L	H	N	S	H	H
				I	G	R	I	G	Y	W	A	E	Q	C	A	A	A	G	F	V	S	I	H	F	V	S	V	V	G	I	P	M	V
				A	P	F	H	G	R	D	S	R	F	G	T	N	P	F	C	V	V	F	P	R	K	D	N	F	P	L	L	L	D
				Y	A	T	S	A	I	A	F	G	K	T	R	V	A	W	H	K	G	V	P	V	P	P	G	C	L	I	D	V	N
				G	V	P	T	T	N	P	A	V	M	Q	E	S	P	L	G	S	L	L	T	F	A	E	H	K	G	Y	A	L	A
				A	M	C	E	I	L	G	G	A	L	S	G	G	K	T	T	H	Q	E	T	L	Q	T	S	P	D	A	I	L	N
				C	M	T	T	I	I	I	N	P	E	L	F	G	A	P	D	C	N	A	Q	T	E	A	F	A	E	W	V	K	A
				S	P	H	D	D	D	K	P	I	L	L	P	G	E	W	E	V	N	T	R	R	E	R	Q	K	Q	G	I	P	L
				D	A	G	S	W	Q	A	I	C	D	A	A	R	Q	I	G	M	P	E	E	T	L	Q	A	F	C	Q	Q	L	A
				S																													
				1				5					10					15					20					25					30
94	2.09%	85.0%	>tr|A0A140NA93|A0A140NA93_ECOBD Integral membrane protein TerC OS=Escherichia coli (strain B / BL21-DE3) OX=469008 GN=ECBD_1592 PE=4 SV=1																														
				M	E	W	I	A	D	P	S	I	W	A	G	L	I	T	L	I	V	I	E	L	V	L	G	I	D	N	L	V	F
				I	A	I	L	A	E	K	L	P	P	K	Q	R	D	R	A	R	V	T	G	L	L	L	A	M	L	M	R	L	L
				L	L	A	S	I	S	W	L	V	T	L	T	Q	P	L	F	S	F	R	S	F	T	F	S	A	R	D	L	I	M
				L	F	G	G	F	F	L	L	F	K	A	T	M	E	L	N	E	R	L	E	G	K	D	S	N	N	P	T	Q	R
				K	G	A	K	F	W	G	V	V	T	Q	I	V	V	L	D	A	I	F	S	L	D	S	V	I	T	A	V	G	M
				V	D	H	L	L	V	M	M	A	A	V	V	I	A	I	S	L	M	L	M	A	S	K	P	L	T	Q	F	V	N
				S	H	P	T	I	V	I	L	C	L	S	F	L	L	M	I	G	F	S	L	V	A	E	G	F	G	F	V	I	P
				K	G	Y	L	Y	A	A	I	G	F	S	V	M	I	E	A	L	N	Q	L	A	I	F	N	R	R	R	F	L	S
				A	N	Q	T	L	R	Q	R	T	T	E	A	V	M	R	L	L	S	G	Q	K	E	D	A	E	L	D	A	E	T
				A	S	M	L	V	D	H	G	N	Q	Q	I	F	N	P	Q	E	R	R	M	I	E	R	V	L	N	L	N	Q	R
				T	V	S	S	I	M	T	S	R	H	D	I	E	H	I	D	L	N	A	P	E	D	E	I	R	Q	L	L	E	R
				N	Q	H	T	R	L	V	V	T	D	G	D	D	A	E	D	L	L	G	V	V	H	V	I	D	L	L	Q	Q	S
				L	R	G	E	P	L	N	L	R	V	L	I	R	Q	P	L	V	F	P	E	T	L	P	L	L	P	A	L	E	Q
				F	R	N	A	R	T	H	F	A	F	V	V	D	E	F	G	S	V	E	G	I	V	T	L	S	D	V	T	E	T
				I	A	G	N	L	P	N	E	V	E	E	I	D	A	R	H	D	I	Q	K	N	A	D	G	S	W	T	A	N	G
				H	M	P	L	E	D	L	V	Q	Y	V	P	L	P	L	D	E	K	R	E	Y	H	T	I	A	G	L	L	M	E
				Y	L	Q	R	I	P	K	P	G	E	E	V	Q	V	G	D	Y	L	L	K	T	L	Q	V	E	S	H	R	V	Q
				K	V	Q	I	I	P	L	H	K	D	G	E	L	E	Y	E	V													
				1				5					10					15					20					25					30
95	8.99%	98.1%	>tr|A0A140NGW4|A0A140NGW4_ECOBD tRNA-dihydrouridine(20/20a) synthase OS=Escherichia coli (strain B / BL21-DE3) OX=469008 GN=dusA PE=3 SV=1																														
				M	H	G	N	S	E	M	Q	K	I	N	Q	T	S	A	M	P	E	K	T	D	V	H	W	S	G	R	F	S	V
				A	P	M	L	D	W	T	D	R	H	C	R	Y	F	L	R	L	L	S	R	N	T	L	L	Y	T	E	M	V	T
				T	G	A	I	I	H	G	K	G	D	Y	L	A	Y	S	E	E	E	H	P	V	A	L	Q	L	G	G	S	D	P
				A	A	L	A	Q	C	A	K	L	A	E	A	R	G	Y	D	E	I	N	L	N	V	G	C	P	S	D	R	V	Q
				N	G	M	F	G	A	C	L	M	G	N	A	Q	L	V	A	D	C	V	K	A	M	R	D	V	V	S	I	P	V
				T	V	K	T	R	I	G	I	D	D	Q	D	S	Y	E	F	L	C	D	F	I	N	T	V	S	G	K	G	E	C
				E	M	F	I	I	H	A	R	K	A	W	L	S	G	L	S	P	K	E	N	R	E	I	P	P	L	D	Y	P	R
				V	Y	Q	L	K	R	D	F	P	H	L	T	M	S	I	N	G	G	I	K	S	L	E	E	A	K	A	H	L	Q
				H	M	D	G	V	M	V	G	R	E	A	Y	Q	N	P	G	I	L	A	A	V	D	R	E	I	F	G	S	S	D
				I	D	A	D	P	V	A	V	V	R	A	M	Y	P	Y	I	E	R	E	L	S	Q	G	T	Y	L	G	H	I	T
				R	H	M	L	G	L	F	Q	G	I	P	G	A	R	Q	W	R	R	Y	L	S	E	N	A	H	K	A	G	A	D
				I	N	V	L	E	H	A	L	K	L	V	A	D	K	R															
				1				5					10					15					20					25					30
96	10.67%	99.0%	>tr|A0A140N7X0|A0A140N7X0_ECOBD Arabinose 5-phosphate isomerase OS=Escherichia coli (strain B / BL21-DE3) OX=469008 GN=ECBD_0545 PE=3 SV=1																														
				M	S	H	V	E	L	Q	P	G	F	D	F	Q	Q	A	G	K	E	V	L	A	I	E	R	E	C	L	A	E	L
				D	Q	Y	I	N	Q	N	F	T	L	A	C	E	K	M	F	W	C	K	G	K	V	V	V	M	G	M	G	K	S
				G	H	I	G	R	K	M	A	A	T	F	A	S	T	G	T	P	S	F	F	V	H	P	G	E	A	A	H	G	D
				L	G	M	V	T	P	Q	D	V	V	I	A	I	S	N	S	G	E	S	S	E	I	T	A	L	I	P	V	L	K
				R	L	H	V	P	L	I	C	I	T	G	R	P	E	S	S	M	A	R	A	A	D	V	H	L	C	V	K	V	A
				K	E	A	C	P	L	G	L	A	P	T	S	S	T	T	A	T	L	V	M	G	D	A	L	A	V	A	L	L	K
				A	R	G	F	T	A	E	D	F	A	L	S	H	P	G	G	A	L	G	R	K	L	L	L	R	V	N	D	I	M
				H	T	G	D	E	I	P	H	V	K	K	T	A	S	L	R	D	A	L	L	E	V	T	R	K	N	L	G	M	T
				V	I	C	D	D	N	M	M	I	E	G	I	F	T	D	G	D	L	R	R	V	F	D	M	G	V	D	V	R	Q
				L	S	I	A	D	V	M	T	P	G	G	I	R	V	R	P	G	I	L	A	V	E	A	L	N	L	M	Q	S	R
				H	I	T	S	V	M	V	A	D	G	D	H	L	L	G	V	L	H	M	H	D	L	L	R	A	G	V	V		
				1				5					10					15					20					25					30
97	10.65%	99.0%	>tr|A0A140NA92|A0A140NA92_ECOBD 3-oxoacyl-[acyl-carrier-protein] synthase 2 OS=Escherichia coli (strain B / BL21-DE3) OX=469008 GN=ECBD_2506 PE=3 SV=1																														
				M	S	K	R	R	V	V	V	T	G	L	G	M	L	S	P	V	G	N	T	V	E	S	T	W	K	A	L	L	A
				G	Q	S	G	I	S	L	I	D	H	F	D	T	S	A	Y	A	T	K	F	A	G	L	V	K	D	F	N	C	E
				D	I	I	S	R	K	E	Q	R	K	M	D	A	F	I	Q	Y	G	I	V	A	G	V	Q	A	M	Q	D	S	G
				L	E	I	T	E	E	N	A	T	R	I	G	A	A	I	G	S	G	I	G	G	L	G	L	I	E	E	N	H	T
				S	L	M	N	G	G	P	R	K	I	S	P	F	F	V	P	S	T	I	V	N	M	V	A	G	H	L	T	I	M
				Y	G	L	R	G	P	S	I	S	I	A	T	A	C	T	S	G	V	H	N	I	G	H	A	A	R	I	I	A	Y
				G	D	A	D	V	M	V	A	G	G	A	E	K	A	S	T	P	L	G	V	G	G	F	G	A	A	R	A	L	S
				T	R	N	D	N	P	Q	A	A	S	R	P	W	D	K	E	R	D	G	F	V	L	G	D	G	A	G	M	L	V
				L	E	E	Y	E	H	A	K	K	R	G	A	K	I	Y	A	E	L	V	G	F	G	M	S	S	D	A	Y	H	M
				T	S	P	P	E	N	G	A	G	A	A	L	A	M	A	N	A	L	R	D	A	G	I	E	A	S	Q	I	G	Y
				V	N	A	H	G	T	S	T	P	A	G	D	K	A	E	A	Q	A	V	K	T	I	F	G	E	A	A	S	R	V
				L	V	S	S	T	K	S	M	T	G	H	L	L	G	A	A	G	A	V	E	S	I	Y	S	I	L	A	L	R	D
				Q	A	V	P	P	T	I	N	L	D	N	P	D	E	G	C	D	L	D	F	V	P	H	E	A	R	Q	V	S	G
				M	E	Y	T	L	C	N	S	F	G	F	G	G	T	N	G	S	L	I	F	K	K	I							
				1				5					10					15					20					25					30
98	8.02%	93.9%	>tr|A0A140N7J9|A0A140N7J9_ECOBD Biotin carboxylase OS=Escherichia coli (strain B / BL21-DE3) OX=469008 GN=ECBD_0489 PE=4 SV=1																														
				M	L	D	K	I	V	I	A	N	R	G	E	I	A	L	R	I	L	R	A	C	K	E	L	G	I	K	T	V	A
				V	H	S	S	A	D	R	D	L	K	H	V	L	L	A	D	E	T	V	C	I	G	P	A	P	S	V	K	S	Y
				L	N	I	P	A	I	I	S	A	A	E	I	T	G	A	V	A	I	H	P	G	Y	G	F	L	S	E	N	A	N
				F	A	E	Q	V	E	R	S	G	F	I	F	I	G	P	K	A	E	T	I	R	L	M	G	D	K	V	S	A	I
				A	A	M	K	K	A	G	V	P	C	V	P	G	S	D	G	P	L	G	D	D	M	D	K	N	R	A	I	A	K
				R	I	G	Y	P	V	I	I	K	A	S	G	G	G	G	G	R	G	M	R	V	V	R	G	D	A	E	L	A	Q
				S	I	S	M	T	R	A	E	A	K	A	A	F	S	N	D	M	V	Y	M	E	K	Y	L	E	N	P	R	H	V
				E	I	Q	V	L	A	D	G	Q	G	N	A	I	Y	L	A	E	R	D	C	S	M	Q	R	R	H	Q	K	V	V
				E	E	A	P	A	P	G	I	T	P	E	L	R	R	Y	I	G	E	R	C	A	K	A	C	V	D	I	G	Y	R
				G	A	G	T	F	E	F	L	F	E	N	G	E	F	Y	F	I	E	M	N	T	R	I	Q	V	E	H	P	V	T
				E	M	I	T	G	V	D	L	I	K	E	Q	L	R	I	A	A	G	Q	P	L	S	I	K	Q	E	E	V	H	V
				R	G	H	A	V	E	C	R	I	N	A	E	D	P	N	T	F	L	P	S	P	G	K	I	T	R	F	H	A	P
				G	G	F	G	V	R	W	E	S	H	I	Y	A	G	Y	T	V	P	P	Y	Y	D	S	M	I	G	K	L	I	C
				Y	G	E	N	R	D	V	A	I	A	R	M	K	N	A	L	Q	E	L	I	I	D	G	I	K	T	N	V	D	L
				Q	I	R	I	M	N	D	E	N	F	Q	H	G	G	T	N	I	H	Y	L	E	K	K	L	G	L	Q	E	K	
				1				5					10					15					20					25					30
99	7.59%	97.6%	>tr|A0A140N9A3|A0A140N9A3_ECOBD Cyclopropane-fatty-acyl-phospholipid synthase OS=Escherichia coli (strain B / BL21-DE3) OX=469008 GN=ECBD_1982 PE=4 SV=1																														
				M	S	S	S	C	I	E	E	V	S	V	P	D	D	N	W	Y	R	I	A	N	E	L	L	S	R	A	G	I	A
				I	N	G	S	A	P	A	D	I	R	V	K	N	P	D	F	F	K	R	V	L	Q	E	G	S	L	G	L	G	E
				S	Y	M	D	G	W	W	E	C	D	R	L	D	M	F	F	S	K	V	L	R	A	G	L	E	N	Q	L	P	H
				H	F	K	D	T	L	R	I	A	G	A	R	L	F	N	L	Q	S	K	K	R	A	W	I	V	G	K	E	H	Y
				D	L	G	N	D	L	F	S	R	M	L	D	P	F	M	Q	Y	S	C	A	Y	W	K	D	A	D	N	L	E	S
				A	Q	Q	A	K	L	K	M	I	C	E	K	L	Q	L	K	P	G	M	R	V	L	D	I	G	C	G	W	G	G
				L	A	H	Y	M	A	S	N	Y	D	V	S	V	V	G	V	T	I	S	A	E	Q	Q	K	M	A	Q	E	R	C

				E G L D V T I L L Q D Y R D L N D Q F D R I V S V G M F E H
				V G P K N Y D T Y F A V V D R N L K P E G I F L L H T I G S
				K K T D L N V D P W I N K Y I F P N G C L P S V R Q I A Q S
				S E P H F V M E D W H N F G A D Y D T T L M A W Y E R F L A
				A W P E I A D N Y S E R F K R M F T Y Y L N A C A G A F R A
				R D I Q L W Q V V F S R G V E N G L R V A R
				1 5 10 15 20 25 30
100	4.63%	92.6%	>tr|A0A140SS69|A0A140SS69_ECOBD Iron-containing alcohol dehydrogenase OS=Escherichia coli (strain B / BL21-DE3) OX=469008 GN=ECBD_4078 PE=4 SV=1	
				M D R I I Q S P G K Y I Q G A D V I N R L G E Y L K P L A E
				R W L V V G D K F V L G F A Q S T V E K S F K D A G L V V E
				I A P F G G E C S Q N E I D R L R G I A E T A Q C G A I L G
				I G G G K T L D T A K A L A H F M G V P V A I A P T I A S T
				D A P C S A L S V I Y T D E G E F D R Y L L L P N N P N M V
				I V D T K I V A G A P A R L L A A G I G D A L A T W F E A R
				A C S R S G A T T M A G G K C T Q A A L A L A E L C Y N T L
				L E E G E K A M L A A E Q H V V T P A L E R V I E A N T Y L
				S G V G F E S G G L A A A H A V H N G L T A I P D A H H Y Y
				H G E K V A F G T L T Q L V L E N A P V E E I E T V A A L S
				H A V G L P I T L A Q L D I K E D V P A K M R I V A E A A C
				A E G E T I H N M P G G A T P D Q V Y A A L L V A D Q Y G Q
				R F L Q E W E
				1 5 10 15 20 25 30
101	5.73%	98.6%	>tr|A0A140N9S7|A0A140N9S7_ECOBD Anaerobic glycerol-3-phosphate dehydrogenase subunit B OS=Escherichia coli (strain B / BL21-DE3) OX=469008 GN=glpB PE=3 SV=1	
				M R F D T V I M G G G L A G L L C G L Q L Q K H G L R C A I
				V T R G Q S A L H F S S G S L D L L S H L P D G Q P V A D I
				H S G L E S L R Q Q A P A H P Y S L L G P Q R V L D L A C Q
				A Q A L I A E S G A Q L Q G S V E L A H Q R I T P L G T L R
				S T W L S S P E V P V W P L P A K K I C V V G I S G L M D F
				Q A H L A A A S L R E L D L S V E T A E I E L P E L D V L R
				N N A T E F R A V N I A R F L D N E E N W P L L L D A L I P
				V A N T C E M I L M P A C F G L A D D K L W R W L N E K L P
				C S L M L L P T L P P S V L G I R L Q N Q L Q R Q F V H Q G
				G V W M P G D E V K K V T C K N G V V N E I W T R N H A D I
				P L R P R F A V L A S G S F F S G G L V A E R N G I R E P I
				L G L D V L Q T A T R G E W Y K G D F F A P Q P W Q Q F G V
				T T D E T L R P S Q A G Q T I E N L F A I G S V L G G F D P
				I A Q G C G G G V C A V S A L H A A Q Q I A Q R A G G Q Q
				1 5 10 15 20 25 30
102	6.20%	80.7%	>tr|A0A140N6I7|A0A140N6I7_ECOBD SseB family protein OS=Escherichia coli (strain B / BL21-DE3) OX=469008 GN=ECBD_1162 PE=4 SV=1	
				M S E T K N E L E D L L E K A A T E P A H R P A F F R T L L
				E S T V W V P G T A A Q G E A V V E D S A L D L Q H W E K E
				D G T S V I P F F T S L E A L Q Q A V E D E Q A F V V M P V
				R T L F E M T L G E T L F L N A K L P T G K E F M P R E I S
				L L I G E E G N P L S S Q E I L E G G E S L I L S E V A E P
				P A Q M I D S L T T L F K T I K P V K R A F I C S I K E N E
				E A Q P N L L I G I E A D G D I E E I I Q A T G S V A T D T
				L P G D E P I D I C Q V K K G E K G I S H F I T E H I A P F
				Y E R R W G G F L R D F K Q N R I I
				1 5 10 15 20 25 30
103	10.37%	99.0%	>tr|A0A140N9Z8|A0A140N9Z8_ECOBD PhosphocarrierHPr family OS=Escherichia coli (strain B / BL21-DE3) OX=469008 GN=ECBD_1489 PE=4 SV=1	
				M F Q L S V Q D I H P G E K A G D K E E A I R Q V A A A L V
				Q A G N V A E G Y V N G M L A R E Q Q T S T F L G N G I A I
				P H G T T D T R D Q V L K T G V Q V F Q F P E G V T W G D S
				Q V A Y V A I G I A A S S D E H L G L L R Q L T H V L S D D
				S V A E Q L K S A T T A E E L R A L L M G E K Q S E Q L K L
				D N E M L T L D I V A S D L L T L Q A L N A A R L K E A G A
				V D A T F V T K A I N E Q P L N L G Q G I W L S D S A E G N
				L R S A I A V S R A A N A F D V D G E T A A M L V S V A M N
				D D Q P I A V L K R L A D L L L D N K A D R L M K A D A A T
				L L A L L T S D D A P T D D V L S A E F V V R N E H G L H A
				R P G T M L V N T I K Q F N S D I T V T N L D G T G K P A N
				G R S L M K V V A L G V K K G H R L R F T A Q G A D A E Q A
				L K A I G D A I A A G L G E G A
				1 5 10 15 20 25 30
104	4.49%	86.0%	>tr|A0A140NEK4|A0A140NEK4_ECOBD Modulator of FtsH protease HflC OS=Escherichia coli (strain B / BL21-DE3) OX=469008 GN=ECBD_3859 PE=3 SV=1	
				M R K S V I A I I I V L V V L Y M S V F V V K E G E R G I
				T L R F G K V L R D D D N K P L V Y E P G L H F K I P F I E
				T V K M L D A R I Q T M D N Q A D R F V T K E K K D L I V D
				S Y I K W R I S D F S R Y Y L A T G G G D I S Q A E V L L K
				R K F S D R L R S E I G R L D V K D I V T D S R G R L T L E
				V R D A L N S G S A G T E D E V T T P A A D N A I A E A A E
				R V T A E T K G K V P V I N P N S M A A L G I E V V D V R I
				K Q I N L P T E V S E A I Y N R M R A E R E A V A R R H R S
				Q G Q E E A E K L R A T A D Y E V T R T L A E A E R Q G R I
				M R G E G D A E A A K L F A D A F S K D P D F Y A F I R S L
				R A Y E N S F S G N Q D V M V M S P D S D F F R Y M K T P T
				S A T R
				1 5 10 15 20 25 30
105	4.81%	83.5%	>tr|A0A140NAN3|A0A140NAN3_ECOBD Isocitrate dehydrogenase [NADP] OS=Escherichia coli (strain B / BL21-DE3) OX=469008 GN=ECBD_2463 PE=4 SV=1	
				M E S K V V V P A Q G K K I T L Q N G K L N V P E N P I I P
				Y I E G D G I G V D V T P A M L K V V D A A V E K A Y K G E
				R K I S W M E I Y T G E K S T Q V Y G Q D V W L P A E T L D
				L I R E Y R V A I K G P L T T P V G G G I R S L N V A L R Q
				E L D L Y I C L R P V R Y Y Q G T P S P V K H P E L T D M V
				I F R E N S E D I Y A G I E W K A D S A D A E K V I K F L R
				E E M G V K K I R F P E H C G I G I K P C S E E G T K R L V
				R A A I E Y A I A N D R D S V T L V H K G N I M K F T E G A
				F K D W G Y Q L A R E E F G G E L I D G G P W L K V K N P N
				T G K E I V I K D V I A D A F L Q Q I L L R P A E Y D V I A
				C M N L N G D Y I S D A L A A Q V G G I G I A P G A N I G D
				E C A L F E A T H G T A P K Y A G Q D K V N P G S I I L S A
				E M M L R H M G W T E A A D L I V K G M E G A I N A K T V T
				Y D F E R L M E G A K L L K C S E F G D A I I K N M
				1 5 10 15 20 25 30
106	6.89%	82.7%	>tr|A0A140NH47|A0A140NH47_ECOBD N-acetyl-gamma-glutamyl-phosphate reductase OS=Escherichia coli (strain B / BL21-DE3) OX=469008 GN=argC PE=3 SV=1	
				M L N T L I V G A S G Y A G A E L V T Y V N R H P H M N I T
				A L T V S A Q S N D A G K L I S D L H P Q L K G I V D L P L
				Q P M S D I S E F S P G V D V V F L A T A H E V S H D L A P
				Q F L E A G C V V F D L S G A F R V N D A T F Y E K Y Y G F
				T H Q Y P E L L E Q A A Y G L A E W C G N K L K E A N L I A
				V P G C Y P T A A Q L A L K P L I D A D L L D L N Q W P V I
				N A T S G V S G A G R K A A I S N S F C E V S L Q P Y G V F
				T H R H Q P E I A T H L G A D V I F T P H L G N F P R G I L
				E T I T C R L K S G V T Q A Q V A Q V L Q Q A Y A H K P L V
				R L Y D K G V P A L K N V V G L P F C D I G F A V Q G E H L
				I I V A T E D N L L K G A A A Q A V Q C A N I R F G Y A E T
				Q S L I
				1 5 10 15 20 25 30
107	4.20%	91.4%	>tr|A0A140NBE6|A0A140NBE6_ECOBD Threonine aldolase OS=Escherichia coli (strain B / BL21-DE3) OX=469008 GN=ECBD_2724 PE=4 SV=1	
				M I D L R S D T V T R P S R A M L E A M M A A P V G D D V Y
				G D D P T V N A L Q D Y A A E L S G K E A A I F L P T G T Q
				A N L V A L L S H C E R G E E Y I V G Q A A H N Y L F E A G
				G A A V L G S I Q P Q P I D A A A D G T L P L D K V A M K I
				K P D D I H F A R T K L L S L E N T H N G K M L P R E Y L K
				E A W E F T R E R N L A L H V D G A R I F N A V V A Y G C E
				L K E I T Q Y C D S F T I C L S K G L G T P V G S L L V G N
				R D Y I K R A I R W R K M A G G G M R Q S G I L A A A G M Y
				A L K N N V A R L Q E D H D N A A W M A E Q L R E A G A D V
				M R Q D T N M L F V R V G E E N A A A L G E Y M K A R N V L
				I N A S P I V R L V T H L D V S R E Q L A E V A A H W R A F
				L A R
				1 5 10 15 20 25 30
108	2.20%	93.6%	>tr|A0A140N996|A0A140N996_ECOBD Aspartate--tRNA ligase OS=Escherichia coli (strain B / BL21-DE3) OX=469008 GN=aspS PE=3 SV=1	
				M R T E Y C G Q L R L S H V G Q Q V T L C G W V N R R R D L
				G S L I F I D M R D R E G I V Q V F F D P D R A D A L K L A
				S E L R N E F C I Q V T G T V R A R D E K N I N R D M A T G
				E I E V L A S S L T I I N R A D V L P L D S N H V N T E E A
				R L K Y R Y L D L R R P E M A Q R L K T R A K I T S L V R R
				F M D D H G F L D I E T P M L T K A T P E G A R D Y L V P S
				R V H K G K F Y A L P Q S P Q L F K Q L L M M S G F D R Y Y
				Q I V K C F R D E D L R A D R Q P E F T Q I D V E T S F M T
				A P Q V R E V M E A L V R H L W L E V K G V D L G D F P V M

				T	F	A	E	A	E	R	R	Y	G	S	D	K	P	D	L	R	N	P	M	E	L	T	D	V	A	D	L	L	K
				S	V	E	F	A	V	F	A	G	P	A	N	D	P	K	G	R	V	A	A	L	R	V	P	G	G	A	S	L	T
				R	K	Q	I	D	E	Y	G	N	F	V	K	I	Y	G	A	K	G	L	A	Y	I	K	V	N	E	R	A	K	G
				L	E	G	I	N	S	P	V	A	K	F	L	N	A	E	I	I	E	D	I	L	D	R	T	A	A	Q	D	G	D
				M	I	F	F	G	A	D	N	K	K	I	V	A	D	A	M	G	A	L	R	L	K	V	G	K	D	L	G	L	T
				D	E	S	K	W	A	P	L	W	V	I	D	F	P	M	F	E	D	D	G	E	G	G	L	T	A	M	H	H	P
				F	T	S	P	K	D	M	T	A	A	E	L	K	A	A	P	E	N	A	V	A	N	A	Y	D	M	V	I	N	G
				Y	E	V	G	G	G	S	V	R	I	H	N	G	D	M	Q	Q	T	V	F	G	I	L	G	I	N	E	E	E	Q
				R	E	K	F	G	F	L	L	D	A	L	K	Y	G	T	P	P	H	A	G	L	A	F	G	L	D	R	L	T	M
				L	L	T	G	T	D	N	I	R	D	V	I	A	F	P	K	T	T	A	A	A	C	L	M	T	E	A	P	S	F
				A	N	P	T	A	L	A	E	L	S	I	Q	V	V	K	K	A	E	N	N										
				1				5					10					15					20					25					30
109	5.74%	94.0%	>tr|C5WA15|C5WA15_ECOBD DNA-binding transcriptional repressor OS=Escherichia coli (strain B / BL21-DE3) GN=gntR PE=4 SV=1																														
				M	K	K	K	R	P	V	L	Q	D	V	A	D	R	V	G	V	T	K	M	T	V	S	R	F	L	R	N	P	E
				Q	V	S	V	A	L	R	G	K	I	A	A	A	L	D	E	L	G	Y	I	P	N	R	A	P	D	I	L	S	N
				A	T	S	R	A	I	G	V	L	L	P	S	L	T	N	Q	V	F	A	E	V	L	R	G	I	E	S	V	T	D
				A	H	G	Y	Q	T	M	L	A	H	Y	G	Y	K	P	E	M	E	Q	E	R	L	E	S	M	L	S	W	N	I
				D	G	L	I	L	T	E	R	T	H	T	P	R	T	L	K	M	I	E	V	A	G	I	P	V	V	E	L	M	D
				S	K	S	P	C	L	D	I	A	V	G	F	D	N	F	E	A	A	R	Q	M	T	T	A	I	I	A	R	G	H
				R	H	I	A	Y	L	G	A	R	L	D	E	R	T	I	I	K	Q	K	G	Y	E	Q	A	M	L	D	A	D	L
				V	P	Y	S	V	M	V	E	Q	S	S	S	Y	S	S	G	I	E	L	I	R	Q	A	R	R	E	Y	P	Q	L
				D	G	V	F	C	T	N	D	D	L	A	V	G	A	A	F	E	C	Q	R	L	G	L	K	V	P	D	D	M	A
				I	A	G	F	H	G	H	D	I	G	Q	V	M	E	P	R	L	A	S	V	L	T	P	R	E	R	M	G	S	I
				G	A	E	R	L	L	A	R	I	R	G	E	S	V	T	P	K	M	L	D	L	G	F	T	L	S	P	G	G	S
				I																													
				1				5					10					15					20					25					30
110	4.96%	84.4%	>tr|A0A140SS84|A0A140SS84_ECOBD Acetylornithine deacetylase OS=Escherichia coli (strain B / BL21-DE3) OX=469008 GN=argE PE=3 SV=1																														
				M	K	N	K	L	P	P	F	I	E	I	Y	R	A	L	I	A	T	P	S	I	S	A	T	E	E	A	L	D	Q
				S	N	A	D	L	I	T	L	L	A	D	W	F	K	D	L	G	F	N	V	E	V	Q	P	V	P	G	T	R	N
				K	F	N	M	L	A	S	I	G	Q	G	A	G	G	L	L	L	A	G	H	T	D	T	V	P	F	D	D	G	R
				W	T	R	D	P	F	T	L	T	E	H	D	G	K	L	Y	G	L	G	T	A	D	M	K	G	F	F	A	F	I
				L	D	A	L	R	D	V	D	V	T	K	L	K	K	P	L	Y	I	L	A	T	A	D	E	E	T	S	M	A	G
				A	R	Y	F	A	E	T	T	A	L	R	P	D	C	A	I	I	G	E	P	T	S	L	Q	P	V	R	A	H	K
				G	H	I	S	N	A	I	R	I	Q	G	Q	S	G	H	S	S	D	P	A	R	G	V	N	A	I	E	L	M	H
				D	A	I	G	H	I	L	Q	L	R	D	N	L	K	E	R	Y	H	Y	E	A	F	T	V	P	Y	P	T	L	N
				L	G	H	I	H	G	G	D	A	S	N	R	I	C	A	C	C	E	L	H	M	D	I	R	P	L	P	G	M	T
				L	N	E	L	N	G	L	L	N	D	A	L	A	P	V	S	E	R	W	P	G	R	L	T	V	D	E	L	H	P
				P	I	P	G	Y	E	C	P	P	N	H	Q	L	V	E	V	V	E	K	L	L	G	A	K	T	E	V	V	N	Y
				C	T	E	A	P	F	I	Q	T	L	C	P	T	L	V	L	G	P	G	S	I	N	Q	A	H	Q	P	D	E	Y
				L	E	T	R	F	I	K	P	T	R	E	L	I	I	Q	V	I	H	H	F	C	W	H							
				1				5					10					15					20					25					30
111	5.65%	93.9%	>tr|A0A140N9G0|A0A140N9G0_ECOBD Dihydroorotate dehydrogenase (quinone) OS=Escherichia coli (strain B / BL21-DE3) OX=469008 GN=pyrD PE=3 SV=1																														
				M	Y	Y	P	F	V	R	K	A	L	F	Q	L	D	P	E	R	A	H	E	F	T	F	Q	Q	L	R	R	I	T
				G	T	P	F	E	A	L	V	R	Q	K	V	P	A	K	P	V	N	C	M	G	L	T	F	K	N	P	L	G	L
				A	A	G	L	D	K	D	G	E	C	I	D	A	L	G	A	M	G	F	G	S	I	E	I	G	T	V	T	P	R
				P	Q	P	G	N	D	K	P	R	L	F	R	L	V	D	A	E	G	L	I	N	R	M	G	F	N	N	L	G	V
				D	N	L	V	E	N	V	K	K	A	H	Y	D	G	V	L	G	I	N	I	G	K	N	K	D	T	P	V	E	Q
				G	K	D	D	Y	L	I	C	M	E	K	I	Y	A	Y	A	G	Y	I	A	I	N	I	S	S	P	N	T	P	G
				L	R	T	L	Q	Y	G	E	A	L	D	D	L	L	T	A	I	K	N	K	Q	N	D	L	Q	A	M	H	H	K
				Y	V	P	I	A	V	K	I	A	P	D	L	S	E	E	E	L	I	Q	V	A	D	S	L	V	R	H	N	I	D
				G	V	I	A	T	N	T	T	L	D	R	S	L	V	Q	G	M	K	N	C	D	Q	T	G	G	L	S	G	R	P
				L	Q	L	K	S	T	E	I	I	R	R	L	S	L	E	L	N	G	R	L	P	I	I	G	V	G	G	I	D	S
				V	I	A	A	R	E	K	I	A	A	G	A	S	L	V	Q	I	Y	S	G	F	I	F	K	G	P	P	L	I	K
				E	I	V	T	H	I																								
				1				5					10					15					20					25					30
112	3.24%	87.0%	>tr|A0A140NAT9|A0A140NAT9_ECOBD Flagellar hook protein FlgE OS=Escherichia coli (strain B / BL21-DE3) OX=469008 GN=ECBD_2524 PE=3 SV=1																														
				M	A	F	S	Q	A	V	S	G	L	N	A	A	A	T	N	L	D	V	I	G	N	N	I	A	N	S	A	T	Y
				G	F	K	S	G	T	A	S	F	A	D	M	F	A	G	S	K	V	G	L	G	V	K	V	A	G	I	T	Q	D
				F	T	D	G	T	T	T	N	T	G	R	G	L	D	V	A	I	S	Q	N	G	F	F	R	L	V	D	S	N	G
				S	V	F	Y	S	R	N	G	Q	F	K	L	D	E	N	R	N	L	V	N	M	Q	G	L	Q	L	T	G	Y	P
				A	T	G	T	P	P	T	I	Q	Q	G	A	N	P	T	N	I	S	I	P	N	T	L	M	A	A	K	T	T	T
				T	A	S	M	Q	I	N	L	N	S	S	D	P	L	P	T	V	T	P	F	S	A	S	N	A	D	S	Y	N	K
				K	G	S	V	T	V	F	D	S	Q	G	N	A	H	D	M	S	V	Y	F	V	K	T	G	D	N	N	W	Q	V
				Y	T	Q	D	S	S	D	P	T	G	T	A	E	P	A	M	K	L	V	F	N	A	N	G	V	L	T	S	N	P
				T	E	N	I	T	T	G	A	I	N	G	A	D	P	A	T	F	S	L	S	F	L	N	S	M	Q	Q	N	T	G
				A	N	N	I	V	A	T	T	Q	N	G	Y	K	P	G	D	L	V	S	Y	Q	I	N	D	D	G	T	V	V	G
				N	Y	S	N	E	Q	T	Q	L	L	G	Q	I	V	L	A	N	F	A	N	N	E	G	L	A	S	E	G	D	N
				V	W	S	A	T	Q	S	S	G	V	A	L	L	G	T	A	G	T	G	N	F	G	T	L	T	N	G	A	L	E
				A	S	N	V	D	L	S	K	E	L	V	N	M	I	V	A	Q	R	N	Y	Q	S	N	A	Q	T	I	K	T	Q
				D	Q	I	L	N	T	L	V	N	L	R																			
				1				5					10					15					20					25					30
113	2.88%	95.8%	>tr|A0A140N8X9|A0A140N8X9_ECOBD Serine hydroxymethyltransferase OS=Escherichia coli (strain B / BL21-DE3) OX=469008 GN=glyA PE=3 SV=1																														
				M	L	K	R	E	M	N	I	A	D	Y	D	A	E	L	W	Q	A	M	E	Q	E	K	V	R	Q	E	E	H	I
				E	L	I	A	S	E	N	Y	T	S	P	R	V	M	Q	A	Q	G	S	Q	L	T	N	K	Y	A	E	G	Y	P
				G	K	R	Y	Y	G	G	C	E	Y	V	D	I	V	E	Q	L	A	I	D	R	A	K	E	L	F	G	A	D	Y
				A	N	V	Q	P	H	S	G	S	Q	A	N	F	A	V	Y	T	A	L	L	E	P	G	D	T	V	L	G	M	N
				L	A	H	G	G	H	L	T	H	G	S	P	V	N	F	S	G	K	L	Y	N	I	V	P	Y	G	I	D	A	T
				G	H	I	D	Y	A	D	L	E	K	Q	A	K	E	H	K	P	K	M	I	I	G	G	F	S	A	Y	S	G	V
				V	D	W	A	K	M	R	E	I	A	D	S	I	G	A	Y	L	F	V	D	M	A	H	V	A	G	L	V	A	A
				G	V	Y	P	N	P	V	P	H	A	H	V	V	T	T	T	T	H	K	T	L	A	G	P	R	G	G	L	I	L
				A	K	G	G	S	E	E	L	Y	K	K	L	N	S	A	V	F	P	G	G	Q	G	G	P	L	M	H	V	I	A
				G	K	A	V	A	L	K	E	A	M	E	P	E	F	K	T	Y	Q	Q	Q	V	A	K	N	A	K	A	M	V	E
				V	F	L	E	R	G	Y	K	V	V	S	G	G	T	D	N	H	L	F	L	V	D	L	V	D	K	N	L	T	G
				K	E	A	D	A	A	L	G	R	A	N	I	T	V	N	K	N	S	V	P	N	D	P	K	S	P	F	V	T	S
				G	I	R	V	G	T	P	A	I	T	R	R	G	F	K	E	A	E	A	K	E	L	A	G	W	M	C	D	V	L
				D	S	I	N	D	E	A	V	I	E	R	I	K	G	K	V	L	D	I	C	A	R	Y	P	V	Y	A			
				1				5					10					15					20					25					30
114	3.57%	89.7%	>tr|C5W9C5|C5W9C5_ECOBD ATP-dependent zinc metalloprotease FtsH OS=Escherichia coli (strain B / BL21-DE3) GN=ftsH PE=3 SV=1																														
				M	A	K	N	L	I	L	W	L	V	I	A	V	V	L	M	S	V	F	Q	S	F	G	P	S	E	S	N	G	R
				K	V	D	Y	S	T	F	L	Q	E	V	N	N	D	Q	V	R	E	A	R	I	N	G	R	E	I	N	V	T	K
				K	D	S	N	R	Y	T	T	Y	I	P	V	Q	D	P	K	L	L	D	N	L	L	T	K	N	V	K	V	V	G
				E	P	P	E	E	P	S	L	L	A	S	I	F	I	S	W	F	P	M	L	L	L	I	G	V	W	I	F	F	M
				R	Q	M	Q	G	G	G	G	K	G	A	M	S	F	G	K	S	K	A	R	M	L	T	E	D	Q	I	K	T	T
				F	A	D	V	A	G	C	D	E	A	K	E	E	V	A	E	L	V	E	Y	L	R	E	P	S	R	F	Q	K	L
				G	G	K	I	P	K	G	V	L	M	V	G	P	P	G	T	G	K	T	L	L	A	K	A	I	A	G	E	A	K
				V	P	F	F	T	I	S	G	S	D	F	V	E	M	F	V	G	V	G	A	S	R	V	R	D	M	F	E	Q	A
				K	K	A	A	P	C	I	I	F	I	D	E	I	D	A	V	G	R	Q	R	G	A	G	L	G	G	G	H	D	E
				R	E	Q	T	L	N	Q	M	L	V	E	M	D	G	F	E	G	N	E	G	I	I	V	I	A	A	T	N	R	P
				D	V	L	D	P	A	L	L	R	P	G	R	F	D	R	Q	V	V	V	G	L	P	D	V	R	G	R	E	Q	I
				L	K	V	H	M	R	R	V	P	L	A	P	D	I	D	A	A	I	I	A	R	G	T	P	G	F	S	G	A	D
				L	A	N	L	V	N	E	A	A	L	F	A	A	R	G	N	K	R	V	V	S	M	V	E	F	E	K	A	K	D
				K	I	M	M	G	A	E	R	R	S	M	V	M	T	E	A	Q	K	E	S	T	A	Y	H	E	A	G	H	A	I
				I	G	R	L	V	P	E	H	D	P	V	H	K	V	T	I	I	P	R	G	R	A	L	G	V	T	F	F	L	P
				E	G	D	A	I	S	A	S	R	Q	K	L	E	S	Q	I	S	T	L	Y	G	G	R	L	A	E	E	I	I	Y
				G	P	E	H	V	S	T	G	A	S	N	D	I	K	V	A	T	N	L	A	R	N	M	V	T	Q	W	G	F	S
				E	K	L	G	P	L	L	Y	A	E	E	E	G	E	V	F	L	G	R	S	V	A	K	A	K	H	M	S	D	E
				T	A	R	I	I	D	Q	E	V	K	A	L	I	E	R	N	Y	N	R	A	R	Q	L	L	T	D	N	M	D	I
				L	H	A	M	K	D	A	L	M	K	Y	E	T	I	D	A	P	Q	I	D	D	L	M	A	R	R	D	V	R	P
				P	A	G	W	E	E	P	G	A	S	N	N	S	G	D	N	G	S	P	K	A	P	R	P	V	D	E	P	R	T
				P	N	P	G	N	T	M	S	E	Q	L	G	D	K																
				1				5					10					15					20					25					30
115	2.42%	92.8%	>tr|A0A140N587|A0A140N587_ECOBD Bifunctional polymyxin resistance protein ArnA OS=Escherichia coli (strain B / BL21-DE3) OX=469008 GN=arnA PE=3 SV=1																														
				M	K	T	V	V	F	A	Y	H	D	M	G	C	L	G	I	E	A	L	L	A	A	G	Y	E	I	S	A	I	F
				T	H	T	D	N	P	G	E	K	A	F	Y	G	S	V	A	R	L	A	A	E	R	G	I	P	V	Y	A	P	D
				N	V	N	H	P	L	W	V	E	R	I	A	Q	L	S	P	D	V	I	F	S	F	Y	Y	R	H	L	I	Y	D
				E	I	L	Q	L	A	P	A	G	A	F	N	L	H	G	S	L	L	P	K	Y	R	G	R	A	P	L	N	W	V
				L	V	N	G	E	T	E	T	G	V	T	L	H	R	M	V	K	R	A	D	A	G	A	I	V	A	Q	L	R	I
				A	I	A	P	D	D	I	A	I	T	L	H	H	K	L	C	H	A	A	R	Q	L	L	E	Q	T	L	P	A	I
				K	H	G	N	I	L	E	I	A	Q	R	E	N	E	A	T	C	F	G	R	R	T	P	D	D	S	F	L	E	W
				H	K	P	A	S	V	L	H	N	M	V	R	A	V	A	D	P	W	P	G	A	F	S	Y	V	G	N	Q	K	F
				T	V	W	S	S	R	V	H	P	H	A	S	K	A	Q	P	G	S	V	I	S	V	A	P	L	L	I	A	C	G
				D	G	A	L	E	I	V	T	G	Q	A	G	D	G	I	T	M	Q	G	S	Q	L	A	Q	T	L	G	L	V	Q
				G	S	R	L	N	S	Q	P	A	C	T	A	R	R	R	T	R	V	L	I	L	G	V	N	G	F	I	G	N	H
				L	T	E	R	L	L	R	E	D	H	Y	E	V	Y	G	L	D	I	G	S	D	A	I	S	R	F	L	N	H	P
				H	F	H	F	V	E	G	D	I	S	I	H	S	E	W	I	E	Y	H	V	K	K	C	D	V	V	L	P	L	V
				A	I	A	T	P	I	E	Y	T	R	N	P	L	R	V	F	E	L	D	F	E	E	N	L	R	I	I	R	Y	C
				V	K	Y	R	K	R	I	I	F	P	S	T	S	E	V	Y	G	M	C	S	D	K	Y	F	D	E	D	H	S	N
				L	I	V	G	P	V	N	K	P	R	W	I	Y	S	V	S	K	Q	L	L	D	R	V	I	W	A	Y	G	E	K
				E	G	L	Q	F	T	L	F	R	P	F	N	W	M	G	P	R	L	D	N	L	N	A	A	R	I	G	S	S	R
				A	I	T	Q	L	I	L	N	L	V	E	G	S	P	I	K	L	I	D	G	G	K	Q	K	R	C	F	T	D	I
				R	D	G	I	E	A	L	Y	R	I	I	E	N	A	G	N	R	C	D	G	E	I	I	N	I	G	N	P	E	N
				E	A	S	I	E	E	L	G	E	M	L	L	A	S	F	E	K	H	P	L	R	H	H	F	P	P	F	A	G	F
				R	V	V	E	S	S	S	Y	Y	G	K	G	Y	Q	D	V	E	H	R	K	P	S	I	R	N	A	H	R	C	L
				D	W	E	P	K	I	D	M	Q	E	T	I	D	E	T	L	D	F	F	L	R	T	V	D	L	T	D	K	P	S

				1 5 10 15 20 25 30
116	4.09%	86.4%	>tr|A0A140NEB0|A0A140NEB0_ECOBD Ubiquinone biosynthesis hydroxylaseUbiH/UbiF/VisC/COQ6 family OS=Escherichia coli (strain B / BL21-DE3) OX=469008 GN=ECBD_2989 PE=4 SV=1	
				MTNQPTEIAIVGGGMVGGALALGLAQHGFA
				VTVIEHAEPAPFVADSQPDVRISAISAASV
				SLLKGLGVWDAVQAMRCHPYRRLETWEWET
				AHVVFDAAELKLPLLGYMVENTVLQQALWQ
				ALEAHPKVTLRVPGSLIALHRHDDLQELEL
				KGGEVIRAKLVIGADGANSQVRQMAGIGVH
				AWQYAQSCMLISVQCENDPGDSTWQQFTPD
				GPRAFLPLFDNWASLVWYDSPARIRQLQNM
				NMAQLQAEIAKHFPSRLGYVTPLAAGAFPL
				TRRHALQYVQPGLALVGDAAHTIHPLAGQG
				VNLGYRDVDALIDVLVNARSYGEAWASYPI
				LKRYQMRRMADNFIMQSGMDLFYAGFSNNL
				PPLRFMRNLGLMAAERAGVLKRQALKYALG
				L
				1 5 10 15 20 25 30
117	5.30%	92.1%	>tr|R9RD46|R9RD46_FUSNU Glutamate formiminotransferase OS=Fusobacterium nucleatum subsp. animalis 4_8 OX=469607 GN=HMPREF0409_00839 PE=4 SV=1	
				MAKIVECIPNYSEGKDLAKIERIVAPYKNN
				PKIKLLSVEPDANYNRTVVTVLGDPDEVKK
				AVIESIGIATKEIDMNKHKGEHKRMGATDV
				VPFLPIQEMTTEECNEISREVGKAVWEKFK
				LPVFLYESTATAPNRVSLPDIRKGEYEGMA
				EKLKQPEWAPDFGERAPHPTAGVTAIGCRM
				PLIAFNINLATTDMSIPKEIAKDIRFSSGG
				FRFIQAGPAEILDKGFVQVTMNIKDYTKNP
				IYRIMETVKMEAKRWGVKVTGCEIIGATPF
				ASLTDSLKYYLACDGIKDDVDAMSMEKVVE
				LMIKYLGLTDFDVKKVLEANI
				1 5 10 15 20 25 30
118	3.27%	87.3%	>tr|A0A140ND70|A0A140ND70_ECOBD Efflux transporterRND familyMFP subunit OS=Escherichia coli (strain B / BL21-DE3) OX=469008 GN=ECBD_3193 PE=3 SV=1	
				MNKNRGFTPLAVVLMLSGSLALTGCDDKQA
				QQGGQQMPAVGVVTVKTEPLQITTELPGRT
				SAYRIAEVRPQVSGIILKRNFKEGSDIEAG
				VSLYQIDPATYQATYDSAKGDLAKAQAAAN
				IAQLTVNRYQKLLGTQYISKQEYDQALADA
				QQANAAVTAAKAAVETARINLAYTKVTSPI
				SGRIGKSNVTEGALVQNGQATALATVQQLD
				PIYVDVTQSSNDFLRLKQELANGTLKQENG
				KAKVSLITSDGIKFPQDGTLEFSDVTVDQT
				TGSITLRAIFPNPDHTLLPGMFVRARLEEG
				LNPNAILVPQQGVTRTPRGDATVLVVGADD
				KVETRPIVASQAIGDKWLVTEGLKAGDRVV
				ISGLQKVRPGVQVKAQEVTADNNQQAASGA
				QPEQSKS
				1 5 10 15 20 25 30
119	4.34%	94.5%	>tr|A0A140N5K0|A0A140N5K0_ECOBD Ubiquinone biosynthesis hydroxylaseUbiH/UbiF/VisC/COQ6 family OS=Escherichia coli (strain B / BL21-DE3) OX=469008 GN=ECBD_0830 PE=4 SV=1	
				MSVIIVGGGMAGATLALAISRLSHGALPVH
				LIEATAPESHAHPGFDGRAIALAAGTCQQL
				ARIGVWQSLADCATAITTVHVSDRGHAGFV
				TLAAEDYQLAALGQVVELHNVGQRLFALLR
				KAPGVTLHCPDRVANVARTQSHVEVTLESG
				ETLTGRVLVAADGTHSALATACGVDWQQEP
				YEQLAVIANVATSVAHEGRAFERFTQHGPL
				AMLPMSDGRCSLVWCHPLERREEVLSWSDE
				KFCRELQSAFGWRLGKITHAGKRSAYPLAL
				TRAARAITHRTVLVGNAAQTLHPIAGQGFN
				LGMRDVMSLAETLTQAQERGEDMGDYGVLC
				RYQQRRQSDREATIGVTDSLVHLFANRWAP
				LVVGRNIGLMTMELFTPARDVLAQRTLGWV
				AR
				1 5 10 15 20 25 30
120	1.33%	87.6%	>tr|A0A140ND17|A0A140ND17_ECOBD Protein translocase subunit SecA OS=Escherichia coli (strain B / BL21-DE3) OX=469008 GN=secA PE=2 SV=1	
				MLIKLLTKVFGSRNDRTLRRMRKVVNIINA
				MEPEMEKLSDEELKGKTAEFRARLEKGEVL
				ENLIPEAFAVVREASKRVFGMRHFDVQLLG
				GMVLNERCIAEMRTGEGKTLTATLPAYLNA
				LTGKGVHVVTVNDYLAQRDAENNRPLFEFL
				GLTVGINLPGMPAPAKREAYAADITYGTNN
				EYGFDYLRDNMAFSPEERVQRKLHYALVDE
				VDSILIDEARTPLIISGPAEDSSEMYKRVN
				KIIPHLIRQEKEDSETFQGEGHFSVDEKSR
				QVNLTERGLVLIEELLVKEGIMDEGESLYS
				PANIMLMHHVTAALRAHALFTRDVDYIVKD
				GEVIIVDEHTGRTMQGRRWSDGLHQAVEAK
				EGVQIQNENQTLASITFQNYFRLYEKLAGM
				TGTADTEAFEFSSIYKLDTVVVPTNRPMIR
				KDLPDLVYMTEAEKIQAIIEDIKERTAKGQ
				PVLVGTISIEKSELVSNELTKAGIKHNVLN
				AKFHANEAAIVAQAGYPAAVTIATNMAGRG
				TDIVLGGSWQAEVAALENPTAEQIEKIKAD
				WQVRHDAVLEAGGLHIIGTERHESRRIDNQ
				LRGRSGRQGDAGSSRFYLSMEDALMRIFAS
				DRVSGMMRKLGMKPGEAIEHPWVTKAIANA
				QRKVESRNFDIRKQLLEYDDVANDQRRAIY
				SQRNELLDVSDVSETINSIREDVFKATIDA
				YIPPQSLEEMWDIPGLQERLKNDFDLDLPI
				AEWLDKEPELHEETLRERILAQSIEVYQRK
				EEVVGAEMMRHFEKGVMLQTLDSLWKEHLA
				AMDYLRQGIHLRGYAQKDPKQEYKRESFSM
				FAAMLESLKYEVISTLSKVQVRMPEEVEEL
				EQQRRMEAERLAQMQQLSHQDDDSAAAAAL
				AAQTGERKVGRNDPCPCGSGKKYKQCHGRL
				Q
				1 5 10 15 20 25 30
121	3.67%	88.1%	>tr|U7U1B1|U7U1B1_FUSNU Uncharacterized protein OS=Fusobacterium nucleatum CTI-6 OX=1316587 GN=HMPREF1767_00107 PE=4 SV=1	
				MVNKIEESYNKCLNLFKEGKRDTEEYRKEL
				ENVIDLAKDNNEFKLCYFNAKFRLAQFYNE
				KHKYDLSKKHFLELINDKNIEEFKLDAIIH
				HAYNLRILKKYDEATFWYEKLSELSTSKYY
				DEAVLEGLAKCATMVNNFEKERENYHILLS
				SCLNKEDFKNLAEKILNLKSQLLSTIDEKQ
				KEKISAEIIYLNDDLDTAYYKLIDIKMKIA
				KSYFNEKKYEDCRKEVQTIFEFLEHSISDM
				QDYAITNANMLLGKTYFEEANFEKAREYFK
				PIANTPKEDKYYKYMISDIHAARNFLTKMK
				1 5 10 15 20 25 30
122	2.02%	91.0%	>tr|A0A140N6F4|A0A140N6F4_ECOBD Flavohemoprotein OS=Escherichia coli (strain B / BL21-DE3) OX=469008 GN=hmp PE=3 SV=1	
				MLDAQTIATVKATIPLLVETGPKLTAHFYD
				RMFTHNPELKEIFNMSNQRNGDQREALFNA
				IAAYASNIENLPALLPAVEKIAQKHTSFQI
				KPEQYNIVGEHLLATLDEMFSPGQEVLDAW
				GKAYGVLANVFINREAEIYNENASKAGGWE
				GTRDFRIVAKTPRSALITSFELEPVDGGAV
				AEYRPGQYLGVWLKPEGFPHQEIRQYSLTR
				KPDGKGYRIAVKREEGGQVSNWLHNHANVG
				DVVKLVAPAGDFFMAVADDTPVTLISAGVG
				QTPMLAMLDTLAKAGHTAQVNWFHAAENGD
				VHAFADEVKELGQSLPRFTAHTWYRQPSEA
				DRAKGQFDSEGLMDLSKLEGAFSDPTMQFY
				LCGPVGFMQFTAKQLVDLGVKQENIHYECF
				GPHKVL
				1 5 10 15 20 25 30
123	5.81%	93.2%	>tr|A0A140N3Q3|A0A140N3Q3_ECOBD L-lactate dehydrogenase OS=Escherichia coli (strain B / BL21-DE3) OX=469008 GN=lldD PE=3 SV=1	
				MIISAASDYRAAAQRILPPFLFHYMDGGAY
				SEYTLRRNVEDLSEVALRQRILKNMSDLSL
				ETTLFNEKLSMPVALAPVGLCGMYARRGEV
				QAAKAADAHGIPFTLSTVSVCPIEEVAPAI
				KRPMWFQLYVLRDRGFMRNALERAKAAGCS
				TLVFTVDMPTPGARYRDAHSGMSGPNAAMR
				RYLQAVTHPQWAWDVGLNGRPHDLGNISAY

				L	G	K	P	T	G	L	E	D	Y	I	G	W	L	G	N	N	F	D	P	S	I	S	W	K	D	L	E	W	I
				R	D	F	W	D	G	P	M	V	I	K	G	I	L	D	P	E	D	A	R	D	A	V	R	F	G	A	D	G	I
				V	V	S	N	H	G	G	R	Q	L	D	G	V	L	S	S	A	R	A	L	P	A	I	A	D	A	V	K	G	D
				I	A	I	L	A	D	S	G	I	R	N	G	L	D	V	V	R	M	I	A	L	G	A	D	T	V	L	L	G	R
				A	F	L	Y	A	L	A	T	A	G	Q	A	G	V	A	N	L	L	N	L	I	E	K	E	M	K	V	A	M	T
				L	T	G	A	K	S	I	S	E	I	T	Q	D	S	L	V	Q	G	L	G	K	E	L	P	A	A	L	A	P	M
				A	K	G	N	A	A																								
				1				5					10					15					20					25					30
124	2.91%	95.4%	>tr|A0A140NB56|A0A140NB56_ECOBD Glucose-1-phosphatase OS=Escherichia coli (strain B / BL21-DE3) OX=469008 GN=ECBD_2592 PE=4 SV=1																														
				M	N	K	T	L	I	T	A	T	V	A	G	I	V	L	L	A	S	N	A	Q	A	Q	T	V	P	E	G	Y	Q
				L	Q	Q	V	L	M	M	S	R	H	N	L	R	A	P	L	A	N	N	G	S	V	L	E	Q	S	T	P	N	K
				W	P	E	W	D	V	P	G	G	Q	L	T	T	K	G	G	V	L	E	V	Y	M	G	H	Y	M	R	E	W	L
				A	E	Q	G	M	V	K	S	G	E	C	P	P	P	D	T	V	Y	A	Y	A	N	S	L	Q	R	T	V	A	T
				A	Q	F	F	I	T	G	A	F	P	G	C	D	I	P	V	H	H	Q	E	K	M	G	T	M	D	P	T	F	N
				P	V	I	T	D	D	S	A	A	F	S	E	K	A	V	A	A	M	E	K	E	L	S	K	L	Q	L	T	D	S
				Y	Q	L	L	E	K	I	V	N	Y	K	D	S	P	A	C	K	E	K	Q	Q	C	S	L	V	D	G	K	N	T
				F	S	A	K	Y	Q	Q	E	P	G	V	S	G	P	L	K	V	G	N	S	L	V	D	A	F	T	L	Q	Y	Y
				E	G	F	P	M	D	Q	V	A	W	G	E	I	K	S	D	Q	Q	W	K	V	L	S	K	L	K	N	G	Y	Q
				D	S	L	F	T	S	P	E	V	A	R	N	V	A	K	P	L	V	S	Y	I	D	K	A	L	V	T	D	R	T
				S	A	P	K	I	T	V	L	V	G	H	D	S	N	I	A	S	L	L	T	A	L	D	F	K	P	Y	Q	L	H
				D	Q	N	E	R	T	P	I	G	G	K	I	V	F	Q	R	W	H	D	S	K	A	N	R	D	L	M	K	I	E
				Y	V	Y	Q	S	A	E	Q	L	R	N	A	D	A	L	T	L	Q	A	P	A	Q	R	V	T	L	E	L	S	G
				C	P	I	D	A	N	G	F	C	P	M	D	K	F	D	S	V	L	N	E	A	V	K							
				1				5					10					15					20					25					30
125	3.71%	87.2%	>tr|A0A140NAH0|A0A140NAH0_ECOBD Fructose-bisphosphate aldolase OS=Escherichia coli (strain B / BL21-DE3) OX=469008 GN=ECBD_1560 PE=4 SV=1																														
				M	T	D	I	A	Q	L	L	G	K	D	A	D	N	L	L	Q	H	R	C	M	T	I	P	S	D	Q	L	Y	L
				P	G	H	D	Y	V	D	R	V	M	I	D	N	N	R	P	P	A	V	L	R	N	M	Q	T	L	Y	N	T	G
				R	L	A	G	T	G	Y	L	S	I	L	P	V	D	Q	G	V	E	H	S	A	G	A	S	F	A	A	N	P	L
				Y	F	D	P	K	N	I	V	E	L	A	I	E	A	G	C	N	C	V	A	S	T	Y	G	V	L	A	S	V	S
				R	R	Y	A	H	R	I	P	F	L	V	K	L	N	H	N	E	T	L	S	Y	P	N	T	Y	D	Q	T	L	Y
				A	S	V	E	Q	A	F	N	M	G	A	V	A	V	G	A	T	I	Y	F	G	S	E	E	S	R	R	Q	I	E
				E	I	S	A	A	F	E	R	A	H	E	L	G	M	V	T	V	L	W	A	Y	L	R	N	S	A	F	K	K	D
				G	V	D	Y	H	V	S	A	D	L	T	G	Q	A	N	H	L	A	A	T	I	G	A	D	I	V	K	Q	K	M
				A	E	N	N	G	G	Y	K	A	I	N	Y	G	Y	T	D	D	R	V	Y	S	K	L	T	S	E	N	P	I	D
				L	V	R	Y	Q	L	A	N	C	Y	M	G	R	A	G	L	I	N	S	G	G	A	A	G	G	E	T	D	L	S
				D	A	V	R	T	A	V	I	N	K	R	A	G	G	M	G	L	I	L	G	R	K	A	F	K	K	S	M	A	D
				G	V	K	L	I	N	A	V	Q	D	V	Y	L	D	S	K	I	T	I	A										
				1				5					10					15					20					25					30
126	4.11%	90.0%	>tr|A0A140N6V3|A0A140N6V3_ECOBD NADH:flavin oxidoreductase/NADH oxidase OS=Escherichia coli (strain B / BL21-DE3) OX=469008 GN=ECBD_1993 PE=4 SV=1																														
				M	S	S	E	K	L	Y	S	P	L	K	V	G	A	I	T	A	A	N	R	I	F	M	A	P	L	T	R	L	R
				S	I	E	P	G	D	I	P	T	P	L	M	A	E	Y	Y	R	Q	R	A	S	A	G	L	I	I	S	E	A	T
				Q	I	S	A	Q	A	K	G	Y	A	G	A	P	G	I	H	S	P	E	Q	I	A	A	W	K	K	I	T	A	G
				V	H	A	E	N	G	H	M	A	V	Q	L	W	H	T	G	R	I	S	H	A	S	L	Q	P	G	G	Q	A	P
				V	A	P	S	A	L	S	A	G	T	R	T	S	L	R	D	E	N	G	Q	A	I	R	V	E	T	S	M	P	R
				A	L	E	L	E	E	I	P	G	I	V	N	D	F	R	Q	A	I	A	N	A	R	E	A	G	F	D	L	V	E
				L	H	S	A	H	G	Y	L	L	H	Q	F	L	S	P	S	S	N	H	R	T	D	Q	Y	G	G	S	V	E	N
				R	A	R	L	V	L	E	V	V	D	A	G	I	E	E	W	G	A	D	R	I	G	I	R	V	S	P	I	G	T
				F	Q	N	T	D	N	G	P	N	E	E	A	D	A	L	Y	L	I	E	Q	L	G	K	R	G	I	A	Y	L	H
				M	S	E	P	D	W	A	G	G	E	P	Y	T	D	A	F	R	E	K	V	R	A	R	F	H	G	P	I	I	G
				A	G	A	Y	T	V	E	K	A	E	T	L	I	G	K	G	L	I	D	A	V	A	F	G	R	D	W	I	A	N
				P	D	L	V	A	R	L	Q	R	K	A	D	L	N	P	Q	R	A	E	S	F	Y	G	G	G	A	E	G	Y	T
				D	Y	P	T	L																									
				1				5					10					15					20					25					30
127	3.05%	94.2%	>tr|A0A140NE56|A0A140NE56_ECOBD Mannonate dehydratase OS=Escherichia coli (strain B / BL21-DE3) OX=469008 GN=uxuA PE=3 SV=1																														
				M	E	Q	T	W	R	W	Y	G	P	N	D	P	V	S	L	A	D	V	R	Q	A	G	A	T	G	V	V	T	A
				L	H	H	I	P	N	G	E	V	W	S	V	E	E	I	L	K	R	K	A	I	I	E	D	A	G	L	V	W	S
				V	V	E	S	V	P	I	H	E	D	I	K	T	H	T	G	N	Y	E	Q	W	I	A	N	Y	Q	Q	T	L	R
				N	L	A	Q	C	G	I	R	T	V	C	Y	N	F	M	P	V	L	D	W	T	R	T	D	L	E	Y	V	L	P
				D	G	S	K	A	L	R	F	D	Q	I	E	F	A	A	F	E	M	H	I	L	K	R	P	G	A	E	A	D	Y
				T	E	E	E	I	A	Q	A	A	E	R	F	A	T	M	S	D	E	D	K	A	R	L	T	R	N	I	I	A	G
				L	P	G	A	E	E	G	Y	T	L	D	Q	F	R	K	H	L	E	L	Y	K	D	I	D	K	A	K	L	R	E
				N	F	A	V	F	L	K	A	I	I	P	V	A	E	E	V	G	V	R	M	A	V	H	P	D	D	P	P	R	P
				I	L	G	L	P	R	I	V	S	T	I	E	D	M	Q	W	M	V	D	T	V	N	S	M	A	N	G	F	T	M
				C	T	G	S	Y	G	V	R	A	D	N	D	L	V	D	M	I	K	Q	F	G	P	R	I	Y	F	T	H	L	R
				S	T	M	R	E	D	N	P	K	T	F	H	E	A	A	H	L	N	G	D	V	D	M	Y	E	V	V	K	A	I
				V	E	E	E	H	R	R	K	A	E	G	K	E	D	L	I	P	M	R	P	D	H	G	H	Q	M	L	D	D	L
				K	K	K	T	N	P	G	Y	S	A	I	G	R	L	K	G	L	A	E	V	R	G	V	E	L	A	I	Q	R	A
				F	F	S	R																										
				1				5					10					15					20					25					30
128	5.18%	91.6%	>tr|A0A140NAM8|A0A140NAM8_ECOBD Glutamate 5-kinase OS=Escherichia coli (strain B / BL21-DE3) OX=469008 GN=proB PE=3 SV=1																														
				M	S	D	S	Q	T	L	V	V	K	L	G	T	S	V	L	T	G	G	S	R	R	L	N	R	A	H	I	V	E
				L	V	R	Q	C	A	Q	L	H	A	A	G	H	R	I	V	I	V	T	S	G	A	I	A	A	G	R	E	H	L
				G	Y	P	E	L	P	A	T	I	A	S	K	Q	L	L	A	A	V	G	Q	S	R	L	I	Q	L	W	E	Q	L
				F	S	I	Y	G	I	H	V	G	Q	M	L	L	T	R	A	D	M	E	D	R	E	R	F	L	N	A	R	D	T
				L	R	A	L	L	D	N	N	I	V	P	V	I	N	E	N	D	A	V	A	T	A	E	I	K	V	G	D	N	D
				N	L	S	A	L	A	A	I	L	A	G	A	D	K	L	L	L	L	T	D	Q	K	G	L	Y	T	A	D	P	R
				S	N	P	Q	A	E	L	I	K	D	V	Y	G	I	D	D	A	L	R	A	I	A	G	D	S	V	S	G	L	G
				T	G	G	M	S	T	K	L	Q	A	A	D	V	A	C	R	A	G	I	D	T	I	I	A	A	G	S	K	P	G
				V	I	G	D	V	M	E	G	I	S	V	G	T	L	F	H	A	Q	A	T	P	L	E	N	R	K	R	W	I	F
				G	A	P	P	A	G	E	I	T	V	D	E	G	A	T	A	A	I	L	E	R	G	S	S	L	L	P	K	G	I
				K	S	V	T	G	N	F	S	R	G	E	V	I	R	I	C	N	L	E	G	R	D	I	A	H	G	V	S	R	Y
				N	S	D	A	L	R	R	I	A	G	H	H	S	Q	E	I	D	A	I	L	G	Y	E	Y	G	P	V	A	V	H
				R	D	D	M	I	T	R																							
				1				5					10					15					20					25					30
129	4.49%	94.1%	>tr|A0A140N5Z1|A0A140N5Z1_ECOBD Peptidase M23 OS=Escherichia coli (strain B / BL21-DE3) OX=469008 GN=ECBD_0982 PE=4 SV=1																														
				M	S	A	G	S	P	K	F	T	V	R	R	I	A	A	L	S	L	V	S	L	W	L	A	G	C	S	D	T	S
				N	P	P	A	P	V	S	S	V	N	G	N	A	P	A	N	T	N	S	G	M	L	I	T	P	P	P	K	M	G
				T	T	S	T	A	Q	Q	P	Q	I	Q	P	V	Q	Q	P	Q	I	Q	A	T	Q	Q	P	Q	I	Q	P	V	Q
				P	V	A	Q	Q	P	V	Q	M	E	N	G	R	I	V	Y	N	R	Q	Y	G	N	I	P	K	G	S	Y	S	G
				S	T	Y	T	V	K	K	G	D	T	L	F	Y	I	A	W	I	T	G	N	D	F	R	D	L	A	Q	R	N	N
				I	Q	A	P	Y	A	L	N	V	G	Q	T	L	Q	V	G	N	A	S	G	T	P	I	T	G	G	N	A	I	T
				Q	A	D	A	A	E	Q	G	V	V	I	K	P	A	Q	N	S	T	V	A	V	A	S	Q	P	T	I	T	Y	S
				E	S	S	G	E	Q	S	A	N	K	M	L	P	N	N	K	P	A	A	T	T	V	T	A	P	V	T	V	P	T
				A	S	T	T	E	P	T	V	S	S	T	S	T	S	T	P	I	S	T	W	R	W	P	T	E	G	K	V	I	E
				T	F	G	A	S	E	G	G	N	K	G	I	D	I	A	G	S	K	G	Q	A	I	I	A	T	A	D	G	R	V
				V	Y	A	G	N	A	L	R	G	Y	G	N	L	I	I	I	K	H	N	D	D	Y	L	S	A	Y	A	H	N	D
				T	M	L	V	R	E	Q	Q	E	V	K	A	G	Q	K	I	A	T	M	G	S	T	G	T	S	S	T	R	L	H
				F	E	I	R	Y	K	G	K	S	V	N	P	L	R	Y	L	P	Q	R											
				1				5					10					15					20					25					30
130	3.18%	89.9%	>tr|A0A140N9E5|A0A140N9E5_ECOBD Glutamate--tRNA ligase OS=Escherichia coli (strain B / BL21-DE3) OX=469008 GN=gltX PE=3 SV=1																														
				M	K	I	K	T	R	F	A	P	S	P	T	G	Y	L	H	V	G	G	A	R	T	A	L	Y	S	W	L	F	A
				R	N	H	G	G	E	F	V	L	R	I	E	D	T	D	L	E	R	S	T	P	E	A	I	E	A	I	M	D	G
				M	N	W	L	S	L	E	W	D	E	G	P	Y	Y	Q	T	K	R	F	D	R	Y	N	A	V	I	D	Q	M	L
				E	E	G	T	A	Y	K	C	Y	C	S	K	E	R	L	E	A	L	R	E	E	Q	M	A	K	G	E	K	P	R
				Y	D	G	R	C	R	H	S	H	E	H	H	A	D	D	E	P	C	V	V	R	F	A	N	P	Q	E	G	S	V
				V	F	D	D	Q	I	R	G	P	I	E	F	S	N	Q	E	L	D	D	L	I	I	R	R	T	D	G	S	P	T
				Y	N	F	C	V	V	V	D	D	W	D	M	E	I	T	H	V	I	R	G	E	D	H	I	N	N	T	P	R	Q
				I	N	I	L	K	A	L	K	A	P	V	P	V	Y	A	H	V	S	M	I	N	G	D	D	G	K	K	L	S	K
				R	H	G	A	V	S	V	M	Q	Y	R	D	D	G	Y	L	P	E	A	L	L	N	Y	L	V	R	L	G	W	S
				H	G	D	Q	E	I	F	T	R	E	E	M	I	K	Y	F	T	L	N	A	V	S	K	S	A	S	A	F	N	T
				D	K	L	L	W	L	N	H	H	Y	I	N	A	L	P	P	E	Y	V	A	T	H	L	Q	W	H	I	E	Q	E
				N	I	D	T	R	N	G	P	Q	L	A	D	L	V	K	L	L	G	E	R	C	K	T	L	K	E	M	A	Q	S
				C	R	Y	F	Y	E	D	F	A	E	F	D	A	D	A	A	K	K	H	L	R	P	V	A	R	Q	P	L	E	V
				V	R	D	K	L	A	A	I	T	D	W	T	A	E	N	V	H	H	A	I	Q	A	T	A	D	E	L	E	V	G
				M	G	K	V	G	M	P	L	R	V	A	V	T	G	A	G	Q	S	P	A	L	D	V	T	V	H	A	I	G	K
				T	R	S	I	E	R	I	N	K	A	L	D	F	I	A	E	R	E	N	Q	Q									
				1				5					10					15					20					25					30
131	2.35%	92.4%	>tr|A0A140N7V6|A0A140N7V6_ECOBD D-tagatose-16-bisphosphate aldolase subunit KbaZ OS=Escherichia coli (strain B / BL21-DE3) OX=469008 GN=kbaZ PE=3 SV=1																														
				M	K	H	L	T	E	M	V	R	Q	H	K	A	G	K	T	N	A	I	Y	A	V	C	S	A	H	P	L	V	L
				E	A	A	I	R	Y	A	S	A	N	Q	T	P	L	L	I	E	A	T	S	N	Q	V	D	Q	F	G	G	Y	T
				G	M	T	P	A	D	F	R	G	F	V	C	Q	L	A	D	S	L	N	F	P	Q	D	A	L	I	L	G	G	D
				H	L	G	P	N	R	W	Q	N	L	P	A	A	Q	A	M	A	N	A	D	D	L	I	K	S	Y	V	A	A	G
				F	K	K	I	H	L	D	C	S	M	S	C	Q	D	D	P	I	P	L	T	D	D	I	V	A	E	R	A	A	R
				L	A	K	V	A	E	E	T	C	L	E	H	F	G	E	A	D	L	E	Y	V	I	G	T	E	V	P	V	P	G
				G	A	H	E	T	L	S	E	L	A	V	T	T	P	D	A	A	R	A	T	L	E	A	H	R	H	A	F	E	K
				Q	G	L	N	A	I	W	P	R	I	I	A	L	V	V	Q	P	G	V	E	F	D	H	T	N	V	I	D	Y	Q
				P	A	K	A	S	A	L	S	Q	M	V	E	N	Y	E	T	L	I	F	E	A	H	S	T	D	Y	Q	T	P	Q
				S	L	R	Q	L	V	I	D	H	F	A	I	L	K	V	G	P	A	L	T	F	A	L	R	E	A	L	F	S	L
				A	A	I	E	E	E	L	V	P	A	K	A	C	S	G	L	R	Q	V	L	E	D	V	M	L	D	R	P	E	Y
				W	Q	S	H	Y	H	G	D	G	N	A	R	R	L	A	R	G	Y	S	Y	S	D	R	V	R	Y	Y	W	P	D
				S	Q	I	D	D	A	F	A	H	L	V	R	N	L	A	D	S	P	I	P	L	P	L	I	S	Q	Y	L	P	L
				Q	Y	V	K	V	R	S	G	E	L	Q	P	T	P	R	E	L	I	I	N	H	I	Q	D	I	L	A	Q	Y	H
				T	A	C	E	G	Q																								

				1				5					10					15					20					25					30
132	5.69%	90.8%	>tr|A0A140N6W2|A0A140N6W2_ECOBD Anhydro-N-acetylmuramic acid kinase OS=Escherichia coli (strain B / BL21-DE3) OX=469008 GN=anmK PE=3 SV=1																														
				M	K	S	G	R	F	I	G	V	M	S	G	T	S	L	D	G	V	D	V	V	L	A	T	I	D	E	H	R	V
				A	Q	L	A	S	L	S	W	P	I	P	V	S	L	K	Q	A	V	L	D	I	C	Q	G	Q	Q	L	T	L	S
				Q	F	G	Q	L	D	T	Q	L	G	R	L	F	A	D	A	V	N	A	L	L	K	E	Q	N	L	Q	A	R	D
				I	V	A	I	G	C	H	G	Q	T	V	W	H	E	P	T	G	V	A	P	H	T	L	Q	I	G	D	N	N	Q
				I	V	A	R	T	G	I	T	V	V	G	D	F	R	R	R	D	I	A	L	G	G	Q	G	A	P	L	V	P	A
				F	H	H	A	L	L	A	H	P	T	E	R	R	M	V	L	N	I	G	G	I	A	N	L	S	L	L	I	P	G
				Q	P	V	G	G	Y	D	T	G	P	G	N	M	L	M	D	A	W	I	W	R	Q	A	G	K	P	Y	D	K	D
				A	E	W	A	R	A	G	K	V	I	L	P	L	L	Q	N	M	L	S	D	P	Y	F	S	Q	P	A	P	K	S
				T	G	R	E	Y	F	N	Y	G	W	L	E	R	H	L	R	H	F	P	G	V	D	P	R	D	V	Q	A	T	L
				A	E	L	T	A	V	T	I	S	E	Q	V	L	L	S	G	G	C	E	R	L	M	V	C	G	G	G	S	R	N
				P	L	L	M	A	R	L	A	A	L	L	P	G	T	E	V	T	T	T	D	A	V	G	I	S	G	D	D	M	E
				A	L	A	F	A	W	L	A	W	R	T	L	A	G	L	P	G	N	L	P	S	V	T	G	A	S	Q	E	T	V
				L	G	A	I	F	P	A	N	P																					
				1				5					10					15					20					25					30
133	3.66%	93.9%	>tr|A0A140NDX6|A0A140NDX6_ECOBD dTDP-glucose 46-dehydratase OS=Escherichia coli (strain B / BL21-DE3) OX=469008 GN=ECBD_4251 PE=3 SV=1																														
				M	R	K	I	L	I	T	G	G	A	G	F	I	G	S	A	L	V	R	Y	I	I	N	E	T	S	D	A	V	V
				V	V	D	K	L	T	Y	A	G	N	L	M	S	L	A	P	V	A	Q	S	E	R	F	A	F	E	K	V	D	I
				C	D	R	A	E	L	A	R	V	F	T	E	H	Q	P	D	C	V	M	H	L	A	A	E	S	H	V	D	R	S
				I	D	G	P	A	A	F	I	E	T	N	I	V	G	T	Y	T	L	L	E	A	A	R	A	Y	W	N	A	L	T
				E	D	K	K	S	A	F	R	F	H	H	I	S	T	D	E	V	Y	G	D	L	H	S	T	D	D	F	F	T	E
				T	T	P	Y	A	P	S	S	P	Y	S	A	S	K	A	S	S	D	H	L	V	R	A	W	L	R	T	Y	G	L
				P	T	L	I	T	N	C	S	N	N	Y	G	P	Y	H	F	P	E	K	L	I	P	L	M	I	L	N	A	L	A
				G	K	S	L	P	V	Y	G	N	G	Q	Q	I	R	D	W	L	Y	V	E	D	H	A	R	A	L	Y	C	V	A
				T	T	G	K	V	G	E	T	Y	N	I	G	G	H	N	E	R	K	N	L	D	V	V	E	T	I	C	E	L	L
				E	E	L	A	P	N	K	P	H	G	V	V	H	Y	S	D	L	I	T	F	V	A	D	R	P	G	H	D	L	R
				Y	A	I	D	A	S	K	I	A	R	E	L	G	W	L	P	Q	E	T	F	E	S	G	M	R	K	T	V	Q	W
				Y	L	A	N	E	S	W	W	K	Q	V	Q	D	G	S	Y	Q	G	E	R	L	G	L	K	G					
				1				5					10					15					20					25					30
134	4.24%	91.5%	>tr|A0A140NH17|A0A140NH17_ECOBD Uroporphyrinogen decarboxylase OS=Escherichia coli (strain B / BL21-DE3) OX=469008 GN=hemE PE=3 SV=1																														
				M	T	E	L	K	N	D	R	Y	L	R	A	L	L	R	Q	P	V	D	V	T	P	V	W	M	M	R	Q	A	G
				R	Y	L	P	E	Y	K	A	T	R	A	Q	A	G	D	F	M	S	L	C	K	N	A	E	L	A	C	E	V	T
				L	Q	P	L	R	R	Y	P	L	D	A	A	I	L	F	S	D	I	L	T	V	P	D	A	M	G	L	G	L	Y
				F	E	A	G	E	G	P	R	F	T	S	P	V	T	C	K	A	D	V	D	K	L	P	I	P	D	P	E	D	E
				L	G	Y	V	M	N	A	V	R	T	I	R	R	E	L	K	G	E	V	P	L	I	G	F	S	G	S	P	W	T
				L	A	T	Y	M	V	E	G	G	S	S	K	A	F	T	V	I	K	K	M	M	Y	A	D	P	Q	A	L	H	A
				L	L	D	K	L	A	K	S	V	T	L	Y	L	N	A	Q	I	K	A	G	A	Q	A	V	M	I	F	D	T	W
				G	G	V	L	T	G	R	D	Y	Q	Q	F	S	L	Y	Y	M	H	K	I	V	D	G	L	L	R	E	N	D	G
				R	R	V	P	V	T	L	F	T	K	G	G	G	Q	W	L	E	A	M	A	E	T	G	C	D	A	L	G	L	D
				W	T	T	D	I	A	D	A	R	R	R	V	G	N	K	V	A	L	Q	G	N	M	D	P	S	M	L	Y	A	P
				P	A	R	I	E	E	E	V	A	T	I	L	A	G	F	G	H	G	E	G	H	V	F	N	L	G	H	G	I	H
				Q	D	V	P	P	E	H	A	G	V	F	V	E	A	V	H	R	L	S	E	Q	Y	H	R						
				1				5					10					15					20					25					30
135	3.81%	91.1%	>tr|A0A140NFJ1|A0A140NFJ1_ECOBD UDP-3-O-(3-hydroxymyristoyl)glucosamine N-acyltransferase OS=Escherichia coli (strain B / BL21-DE3) OX=469008 GN=lpxD PE=3 SV=1																														
				M	P	S	I	R	L	A	D	L	A	Q	Q	L	D	A	E	L	H	G	D	G	D	I	V	I	T	G	V	A	S
				M	Q	S	A	Q	T	G	H	I	T	F	M	V	N	P	K	Y	R	E	H	L	G	L	C	Q	A	S	A	V	V
				M	T	Q	D	D	L	P	F	A	K	S	A	A	L	V	V	K	N	P	Y	L	T	Y	A	R	M	A	Q	I	L
				D	T	T	P	Q	P	A	Q	N	I	A	P	S	A	V	I	G	A	T	A	K	L	G	N	N	V	S	I	G	A
				N	A	V	I	E	S	G	V	E	L	G	D	N	V	I	I	G	A	G	C	F	V	G	K	N	S	K	I	G	A
				G	S	R	L	W	A	N	V	T	I	Y	H	E	I	Q	I	G	Q	N	C	L	I	Q	S	G	T	V	V	G	A
				D	G	F	G	Y	A	N	D	R	G	N	W	V	K	I	P	Q	I	G	R	V	I	I	G	D	R	V	E	I	G
				A	C	T	T	I	D	R	G	A	L	D	D	T	I	I	G	N	G	V	I	I	D	N	Q	C	Q	I	A	H	N
				V	V	I	G	D	N	T	A	V	A	G	G	V	I	M	A	G	S	L	K	I	G	R	Y	C	M	I	G	G	A
				S	V	I	N	G	H	M	E	I	C	D	K	V	T	V	T	G	M	G	M	V	M	R	P	I	T	E	P	G	V
				Y	S	S	G	I	P	L	Q	P	N	K	V	W	R	K	T	A	A	L	V	M	N	I	D	D	M	S	K	R	L
				K	S	L	E	R	K	V	N	Q	Q	D																			
				1				5					10					15					20					25					30
136	3.76%	95.2%	>tr|A0A140N877|A0A140N877_ECOBD Hydrogenase (NiFe) small subunit HydA OS=Escherichia coli (strain B / BL21-DE3) OX=469008 GN=ECBD_0741 PE=4 SV=1																														
				M	T	G	D	N	T	L	I	H	S	H	G	I	N	R	R	D	F	M	K	L	C	A	A	L	A	A	T	M	G
				L	S	S	K	A	A	A	E	M	A	E	S	V	T	N	P	Q	R	P	P	V	I	W	I	G	A	Q	E	C	T
				G	C	T	E	S	L	L	R	A	T	H	P	T	V	E	N	L	V	L	E	T	I	S	L	E	Y	H	E	V	L
				S	A	A	F	G	H	Q	V	E	E	N	K	H	N	A	L	E	K	Y	K	G	Q	Y	V	L	V	V	D	G	S
				I	P	L	K	D	N	G	I	Y	C	M	V	A	G	E	P	I	V	D	H	I	R	K	A	A	E	G	A	A	A
				I	I	A	I	G	S	C	S	A	W	G	G	V	A	A	A	G	V	N	P	T	G	A	V	S	L	Q	E	V	L
				P	G	K	T	V	I	N	I	P	G	C	P	P	N	P	H	N	F	L	A	T	V	A	H	I	I	T	Y	G	K
				P	P	K	L	D	D	K	N	R	P	T	F	A	Y	G	R	L	I	H	E	H	C	E	R	R	P	H	F	D	A
				G	R	F	A	K	E	F	G	D	E	G	H	R	E	G	W	C	L	Y	H	L	G	C	K	G	P	E	T	Y	G
				N	C	S	T	L	Q	F	C	D	V	G	G	V	W	P	V	A	I	G	H	P	C	Y	G	C	N	E	E	G	I
				G	F	H	K	G	I	H	Q	L	A	N	V	E	N	Q	T	P	R	S	Q	K	P	D	V	N	A	K	E	G	G
				N	V	S	A	G	A	I	G	L	L	G	G	V	V	G	L	V	A	G	V	S	V	M	A	V	R	E	L	G	R
				Q	Q	K	K	D	N	A	D	S	R	G	E																		
				1				5					10					15					20					25					30
137	4.73%	91.0%	>tr|A0A140N478|A0A140N478_ECOBD Lipopolysaccharide 3-alpha-galactosyltransferase OS=Escherichia coli (strain B / BL21-DE3) OX=469008 GN=ECBD_0097 PE=4 SV=1																														
				M	S	A	H	Y	F	N	P	Q	E	M	I	N	K	T	I	I	F	D	E	R	P	A	A	S	V	A	S	S	F
				H	V	A	Y	G	I	D	K	N	F	L	F	G	C	G	V	S	I	T	S	V	L	L	H	N	N	D	V	S	F
				V	F	H	V	F	I	D	D	I	P	E	A	D	I	Q	R	L	A	Q	L	A	K	S	Y	R	T	C	I	Q	I
				H	L	V	N	C	E	R	L	K	A	L	P	T	T	K	N	W	S	I	A	M	Y	F	R	F	V	I	A	D	Y
				F	I	D	Q	Q	D	K	I	L	Y	L	D	A	D	I	A	C	Q	G	N	L	K	P	L	I	T	M	D	L	A
				N	N	V	A	A	V	V	T	E	R	D	A	N	W	W	S	L	R	G	Q	S	L	Q	C	N	E	L	E	K	G
				Y	F	N	S	G	V	L	L	I	N	T	L	A	W	A	Q	E	S	V	S	A	K	A	M	S	M	L	A	D	K
				A	I	V	S	R	L	T	Y	M	D	Q	D	I	L	N	L	I	L	L	G	K	V	K	F	I	D	A	K	Y	N
				T	Q	F	S	L	N	Y	E	L	K	K	S	F	V	C	P	I	N	D	E	T	V	L	I	H	Y	V	G	P	T
				K	P	W	H	Y	W	A	G	Y	P	S	A	Q	P	F	I	K	A	K	E	A	S	P	W	K	N	E	P	L	M
				R	P	V	N	S	N	Y	A	R	Y	C	A	K	H	N	F	K	Q	N	K	P	I	N	G	I	M	N	Y	I	Y
				Y	F	Y	L	K	I	I	K																						
				1				5					10					15					20					25					30
138	2.86%	91.4%	>tr|A0A140NG83|A0A140NG83_ECOBD UDP-N-acetyl-D-mannosamine dehydrogenase OS=Escherichia coli (strain B / BL21-DE3) OX=469008 GN=wecC PE=3 SV=1																														
				M	S	F	A	T	I	S	V	I	G	L	G	Y	I	G	L	P	T	A	A	A	F	A	S	R	Q	K	Q	V	I
				G	V	D	I	N	Q	H	A	V	D	T	I	N	R	G	E	I	H	I	V	E	P	D	L	A	S	V	V	K	T
				A	V	E	G	G	F	L	R	A	S	T	T	P	V	E	A	D	A	W	L	I	A	V	P	T	P	F	K	G	D
				H	E	P	D	M	T	Y	V	E	S	A	A	R	S	I	A	P	V	L	K	K	G	A	L	V	I	L	E	S	T
				S	P	V	G	S	T	E	K	M	A	E	W	L	A	E	M	R	P	D	L	T	F	P	Q	Q	V	G	E	Q	A
				D	V	N	I	A	Y	C	P	E	R	V	L	P	G	Q	V	M	V	E	L	I	K	N	D	R	V	I	G	G	M
				T	P	V	C	S	A	R	A	S	E	L	Y	K	I	F	L	E	G	E	C	V	V	T	N	S	R	T	A	E	M
				C	K	L	T	E	N	S	F	R	D	V	N	I	A	F	A	N	E	L	S	L	I	C	A	D	Q	G	I	N	V
				W	E	L	I	R	L	A	N	R	H	P	R	V	N	I	L	Q	P	G	P	G	V	G	G	H	C	I	A	V	D
				P	W	F	I	V	A	Q	N	P	Q	Q	A	R	L	I	R	T	A	R	E	V	N	D	H	K	P	F	W	V	I
				D	Q	V	K	A	A	V	A	D	C	L	A	A	T	D	K	R	A	S	E	L	K	I	A	C	F	G	L	A	F
				K	P	N	I	D	D	L	R	E	S	P	A	M	E	I	A	E	L	I	A	Q	W	H	S	G	E	T	L	V	V
				E	P	N	I	H	Q	L	P	K	K	L	T	G	L	C	T	L	A	Q	L	D	E	A	L	A	T	A	D	V	L
				V	M	L	V	D	H	S	Q	F	K	V	I	N	G	D	N	V	H	Q	Q	Y	V	V	D	A	K	G	V	W	R
				1				5					10					15					20					25					30
139	2.69%	88.5%	>tr|A0A140NGE4|A0A140NGE4_ECOBD Oxidoreductase domain protein OS=Escherichia coli (strain B / BL21-DE3) OX=469008 GN=ECBD_3762 PE=4 SV=1																														
				M	I	N	Y	G	V	V	G	V	G	Y	F	G	A	E	L	A	R	F	M	N	M	H	D	N	A	K	I	T	C
				V	Y	D	P	E	N	G	E	N	I	A	R	E	L	Q	C	I	N	M	S	S	L	D	A	L	V	S	S	K	L
				V	D	C	V	I	V	A	T	P	N	Y	L	H	K	E	P	V	I	K	A	A	K	N	K	K	H	V	F	C	E
				K	P	I	A	L	S	Y	E	D	C	V	D	M	V	K	A	C	K	E	A	G	V	T	F	M	A	G	H	I	M
				N	F	F	N	G	V	Q	Y	A	R	K	L	I	K	E	G	V	I	G	E	I	L	S	C	H	T	K	R	N	G
				W	E	N	K	Q	E	R	L	S	W	K	K	M	K	E	Q	S	G	G	H	L	Y	H	H	I	H	E	L	D	C
				V	Q	H	L	L	G	E	I	P	E	T	V	T	M	I	G	G	N	L	A	H	S	G	P	G	F	G	N	E	D
				D	M	L	F	M	T	L	E	F	P	S	G	K	L	A	T	L	E	W	G	S	A	F	N	W	P	E	H	Y	V
				I	I	N	G	T	K	G	S	I	K	I	D	M	Q	E	T	A	G	S	L	R	I	G	G	Q	T	K	H	F	L
				V	H	E	T	Q	E	E	D	D	D	R	R	K	G	N	M	T	S	E	M	D	G	A	I	A	Y	G	H	P	G
				K	K	T	P	L	W	L	A	S	L	I	R	K	E	T	L	F	L	H	N	I	L	C	G	A	K	P	E	E	D
				Y	I	D	L	L	N	G	E	A	A	M	S	A	I	A	T	A	D	A	A	T	L	S	R	S	Q	D	R	K	V
				K	I	S	E	I	I	K	H	T	S	V	M																		
				1				5					10					15					20					25					30
140	3.49%	96.7%	>tr|A0A140NAV7|A0A140NAV7_ECOBD Cupin 4 family protein OS=Escherichia coli (strain B / BL21-DE3) OX=469008 GN=ECBD_2471 PE=4 SV=1																														
				M	E	Y	Q	L	T	L	N	W	P	D	F	L	E	R	H	W	Q	K	R	P	V	V	L	K	R	G	F	N	N
				F	I	D	P	I	S	P	D	E	L	A	G	L	A	M	E	S	E	V	D	S	R	L	V	S	H	Q	D	G	K
				W	Q	V	S	H	G	P	F	E	S	Y	D	H	L	G	E	T	N	W	S	L	L	V	Q	A	V	N	H	W	H
				E	P	T	A	A	L	M	R	P	F	R	E	L	P	D	W	R	I	D	D	L	M	I	S	F	S	V	P	G	G
				G	V	G	P	H	L	D	Q	Y	D	V	F	I	I	Q	G	T	G	R	R	R	W	R	V	G	E	K	L	Q	M
				K	Q	H	C	P	H	P	D	L	L	Q	V	D	P	F	E	A	I	I	D	E	E	L	E	P	G	D	I	L	Y
				I	P	P	G	F	P	H	E	G	Y	A	L	E	N	A	M	N	Y	S	V	G	F	R	A	P	N	T	R	E	L
				I	S	G	F	A	D	Y	V	L	Q	R	E	L	G	G	N	Y	Y	S	D	P	D	V	P	P	R	A	H	P	A
				D	V	L	P	Q	E	M	D	K	L	R	E	M	M	L	E	L	I	N	Q	P	E	H	F	K	Q	W	F	G	E
				F	I	S	Q	S	R	H	E	L	D	I	A	P	P	E	P	P	Y	Q	P	D	E	I	Y	D	A	L	K	Q	G
				D	V	L	V	R	L	G	G	L	R	V	L	R	I	G	D	D	V	Y	A	N	G	E	K	I	D	S	P	H	R
				P	A	L	D	A	L	A	S	N	I	A	L	T	A	E	N	F	G	D	A	L	E	D	P	S	F	L	A	M	L

			A A L V N S G Y W F F E G
			1 5 10 15 20 25 30
141	3.46%	90.6%	>tr|A0A140NHM2|A0A140NHM2_ECOBD dTDP-4-amino-46-dideoxygalactose transaminase OS=Escherichia coli (strain B / BL21-DE3) OX=469008 GN=wecE PE=3 SV=1
			M I P F N A P P V V G T E L D Y M Q S A M G S G K L C G D G
			G F T R R C Q Q W L E Q R F G S A K V L L T P S C T A S L E
			M A A L L L D I Q P G D E V I M P S Y T F V S T A N A F V L
			R G A K I V F V D V R P D T M N I D E T L I E A A I T D K T
			R V I V P V H Y A G V A C E M D T I M A L A K K H N L F V V
			E D A A Q G V M S T Y K G R A L G T I G H I G C F S F H E T
			K N Y T A G G E G G A T L I N D K A L I E R A E I I R E K G
			T N R S Q F F R G Q V D K Y T W R D I G S S Y L M S D L Q A
			A Y L W A Q L E A A D R I N Q Q R L A L W Q N Y Y D A L A P
			L A K A G R I E L P S I P D G C V Q N A H M F Y I K L R D I
			D D R S A L I N F L K E A E I M A V F H Y I P L H G C P A G
			E H F G E F H G E D R Y T T K E S E R L L R L P L F Y N L S
			P V N Q R T V I A T L L N Y F S
			1 5 10 15 20 25 30
142	2.88%	94.0%	>tr|A0A140N666|A0A140N666_ECOBD Aminotransferase class I and II OS=Escherichia coli (strain B / BL21-DE3) OX=469008 GN=ECBD_0162 PE=4 SV=1
			M T F S L F G D K F T R H S G I T L L M E D L N D G L R T P
			G A I M L G G G N P A Q I P E M Q D Y F Q T L L T D M L E S
			G K A T D A L C N Y D G P Q G K T E L L T L L A G M L R E K
			L G W D I E P Q N I A L T N G S Q S A F F Y L F N L F A G R
			R A D G R V K K V L F P L A P E Y I G Y A D A G L E E D L F
			V S A R P N I E L L P E G Q F K Y H V D F E H L H I G E E T
			G M I C V S R P T N P T G N V I T D E E L L K L D A L A N Q
			H G I P L V I D N A Y G V P F P G I I F S E A R P L W N P N
			I V L C M S L S K L G L P G S R C G I I I A N E K I I T A I
			T N M N G I I S L A P G G I G P A M M C E M I K R N D L L R
			L S E T V I K P F Y Y Q R V Q E T I A I I R R Y L P E N R C
			L I H K P E G A I F L W L W F K D L P I T T E Q L Y Q R L K
			A R G V L M V P G H N F F P G L D K P W P H T H Q C M R M N
			Y V P E P E K I E A G V K I L A E E I E R A W A E S H
			1 5 10 15 20 25 30
143	4.06%	87.0%	>tr|A0A140N852|A0A140N852_ECOBD Alcohol dehydrogenase zinc-binding domain protein OS=Escherichia coli (strain B / BL21-DE3) OX=469008 GN=ECBD_2190 PE=4 SV=1
			M G Q Q K Q R N R R W V L A S R P H G A P V P E N F R L E E
			D D V A T P G E G Q V L L R T V Y L S L D P Y M R G R M S D
			E P S Y S P P V D I G G V M V G G T V S R V V E S N H P D Y
			Q P G D W V L G Y S G W Q D Y D I S S G D D L V K L G D H P
			Q N P S W S L G V L G M P G F T A Y M G L L D I G Q P K E G
			E T L V V A A A T G P V G A T V G Q I G K L K G C R V V G V
			A G G A E K C R H A I E V L G F D V C L D H H A D D F A E Q
			L A K A C P K G I D I Y Y E N V G G K V F D A V L P L L N T
			S A R I P V C G L V C S Y N A T E L P P G P D R L P L L M A
			T V L K K R I R L Q G F I I A Q D Y G H R I H E F Q K E M G
			Q W V K E D K I H Y R E D I T D G L E N A P Q T F I G L L K
			G K N F G K V V I R V A G D D
			1 5 10 15 20 25 30
144	3.10%	95.4%	>tr|A0A140NAS1|A0A140NAS1_ECOBD PTS systemgalactitol-specific IIC subunit OS=Escherichia coli (strain B / BL21-DE3) OX=469008 GN=ECBD_1565 PE=4 SV=1
			M F S E V M R Y I L D L G P T V M L P I V I I I F S K I L G
			M K A G D C F K A G L H I G I G F V G I G L V I G L M L D S
			I G P A A K A M A E N F D L N L H V V D V G W P G S S P M T
			W A S Q I A L V A I P I A I L V N V A M L L T R M T R V V N
			V D I W N I W H M T F T G A L L H L A T G S W M I G M A G V
			V I H A A F V Y K L G D W F A R D T R N F F E L E G I A I P
			H G T S A Y M G P I A V L V D A I I E K I P G V N R I K F S
			A D D I Q R K F G P F G E P V T V G F V M G L I I G I L A G
			Y D V K G V L Q L A V K T A A V M L L M P R V I K P I M D G
			L T P I A K Q A R S R L Q A K F G G Q E F L I G L D P A L L
			L G H T A V V S A S L I F I P L T I L I A V C V P G N Q V L
			P F G D L A T I G F F V A M A V A V H R G N L F R T L I S G
			V I I M S I T L W I A T Q T I G L H T Q L A A N A G A L K A
			G G M V A S M D Q G G S P I T W L L I Q V F S P Q N I P G F
			I I I G A I Y L T G I F M T W R R A R G F I K Q E K V V L A
			E
			1 5 10 15 20 25 30
145	3.66%	88.4%	>tr|R9RAU6|R9RAU6_FUSNU Benzoyl-CoA reductasesubunit C OS=Fusobacterium nucleatum subsp. animalis 4_8 OX=469607 GN=HMPREF0409_01242 PE=4 SV=1
			M A E I K E L L E Q F K Y Y A E N P R K Q L D K Y L A E G K
			K A V G I F P Y Y A P E E I V Y A G G M V P F G V W G G Q G
			P I E K A K D Y F P T F Y Y S L A L R C L E M A L D G T L D
			G L S A S M L T T L D D T L R P L S Q N Y K V S A G K K I P
			M I F L N H A Q H R K E D F G K Q Y N A K I F N N A K E E L
			E K I C N V K I T D E N L K K A F K V Y N E N R E E K R R F
			I K L A A K H P Q S V K A S D R S N V L K S S Y F M L K D E
			H T D L L R I L N Q Q L E A L P E E Q W D G V R V V T S G I
			I T D N P G L L E V F D N Y K V C V V A D D V A H E S R A L
			K V D I D L S I A D P M L A L A D Q F A R M D E D P L L Y D
			P D I I K R P K Y V L D L V K E N N A D G C L L F M M N F N
			D T E E M E Y P S L K Q A F D E A K V P L I K M G Y D Q Q M
			V D F G Q V K T Q L E T F N E L V Q L S R F
			1 5 10 15 20 25 30
146	3.75%	91.1%	>tr|A0A140N536|A0A140N536_ECOBD Signal recognition particle protein OS=Escherichia coli (strain B / BL21-DE3) OX=469008 GN=ffh PE=3 SV=1
			M F D N L T D R L S R T L R N I S G R G R L T E D N V K D T
			L R E V R M A L L E A D V A L P V V R E F I N R V K E K A V
			G H E V N K S L T P G Q E F V K I V R N E L V A A M G E E N
			Q T L N L A A Q P P A V V L M A G L Q G A G K T T S V G K L
			G K F L R E K H K K K V L V V S A D V Y R P A A I K Q L E T
			L A E Q V G V D F F P S D V G Q K P V D I V N A A L K E A K
			L K F Y D V L L V D T A G R L H V D E A M M D E I K Q V H A
			S I N P V E T L F V V D A M T G Q D A A N T A K A F N E A L
			P L T G V V L T K V D G D A R G G A A L S I R H I T G K P I
			K F L G V G E K T E A L E P F H P D R I A S R I L G M G D V
			L S L I E D I E S K V D R A Q A E K L A S K L K K G D G F D
			L N D F L E Q L R Q M K N M G G M A S L M G K L P G M G Q I
			P D N V K S Q M D D K V L V R M E A I I N S M T M K E R A K
			P E I I K G S R K R R I A A G C G M Q V Q D V N R L L K Q F
			D D M Q R M M K K M K K G G M A K M M R S M K G M M P P G F
			P G R
			1 5 10 15 20 25 30
147	3.30%	86.1%	>tr|A0A140NBN7|A0A140NBN7_ECOBD Tyrosine--tRNA ligase OS=Escherichia coli (strain B / BL21-DE3) OX=469008 GN=tyrS PE=1 SV=1
			M A S S N L I K Q L Q E R G L V A Q V T D E E A L A E R L A
			Q G P I A L Y C G F D P T A D S L H L G H L V P L L C L K R
			F Q Q A G H K P V A L V G G A T G L I G D P S F K A A E R K
			L N T E E T V Q E W V D K I R K Q V A P F L D F D C G E N S
			A I A A N N Y D W F G N M N V L T F L R D I G K H F S V N Q
			M I N K E A V K Q R L N R E D Q G I S F T E F S Y N L L Q G
			Y D F A C L N K Q Y G V V L Q I G G S D Q W G N I T S G I D
			L T R R L H Q N Q V F G L T V P L I T K A D G T K F G K T E
			G G A V W L D P K K T S P Y K F Y Q F W I N T A D A D V Y R
			F L K F F T F M S I E E I N A L E E E D K N S G K A P R A Q
			Y V L A E Q V T R L V H G E E G L Q A A K R I T E C L F S G
			S L S A L S E A D F E Q L A Q D G V P M V E M E K G A D L M
			Q A L V D S E L Q P S R G Q A R K T I A S N A I T I N G E K
			Q S D P E Y F F K E E E R L F G R F T L L R R G K K N Y C L
			I C W K
			1 5 10 15 20 25 30
